# Advanced Catalysts for Olefin Synthesis: Copper(II) Quinoline-Fused Oxazolidines in Alcohol Dehydration

**DOI:** 10.1021/acs.joc.5c02669

**Published:** 2025-12-31

**Authors:** Aurodeep Panda, Ruilin Zhang, William W. Brennessel, William D. Jones

**Affiliations:** Department of Chemistry, 6927 University of Rochester, Rochester, New York 14627, United States

## Abstract

The new copper­(II) catalyst [Cu­(^Q^FOX)­(CH_3_CN)]­[OTf]_2_ has been synthesized and found to be active for alcohol dehydration to give olefins at 120 °C with a 1% catalyst loading (^Q^FOX = di-2-quinolinyl-fused-oxazolidine). Benzylic, allylic, and tertiary alcohols are all dehydrated. In some cases, ethers are observed to be produced, but these are re-entrained into the catalytic cycle. Unactivated secondary alcohols are also dehydrated. Similar catalysts employing Mn^II^, Fe^II^, Co^II^, and Ni^II^ were also synthesized but showed reduced dehydration activity compared to the copper­(II) derivative. A Cu^I^ analog was also prepared but did not show improved dehydration activity.

## Graphical abstract

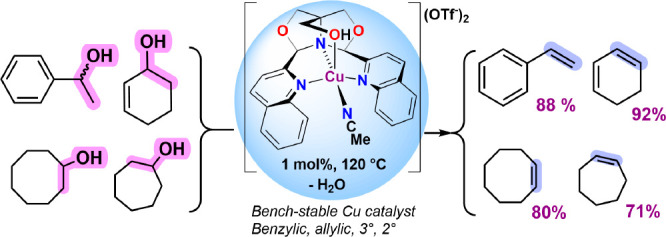

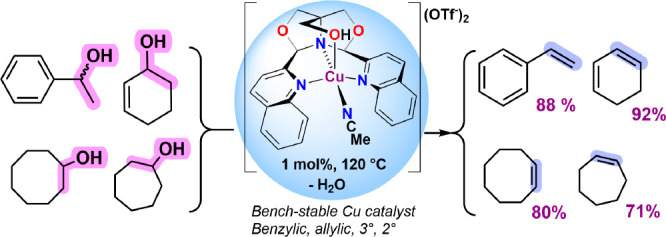

## Introduction

Olefins are important industrial building blocks that are predominantly obtained from petroleum. Due to the overexploitation of petroleum for the production of fossil fuels, it is necessary to explore sustainable routes to olefin production. Among these, alcohol dehydration is a simple, sustainable pathway to access different types of alkenes. Current industrial processes for alkene production, as well as literature reports, are predominantly based on numerous zirconia,^1^ alumina,^2^ or zeolite-based heterogeneous catalysts,^3^ while homogeneous catalyst systems are few and are based on Brønsted acids.^4^ These systems are corrosive to equipment and generate copious amounts of waste. Coupled with these issues, the elimination of water has a competing nucleophilic substitution reaction to generate ethers rather than olefins, making the development of olefin-selective catalytic systems challenging.^5^

Espenson and Zhu, in 1996, reported that methyltrioxorhenium (MTO) catalyzed the dehydration of several alcohols to yield the corresponding ethers in good yields.^6^ The alkenes were obtained in lower yields, showing the preference of the system for the nucleophilic substitution product. Klein-Gebbink and coworkers followed up by using suspended Re_2_O_7_ in toluene as an excellent catalyst for the dehydration of alcohols, including 1-phenylethanol to styrene, without the use of a Brønsted acid.^7^ They also reported a bulky Cp^tBu3^ReO_3_ catalyst that was active for diol dehydration.^8^ Initial reports of using nonprecious metal catalysts was demonstrated by Laali et al., who used 10 mol % Cu­(OTf)_2_ to dehydrate various primary, secondary, and tertiary alcohols at ∼160 °C to their corresponding olefins in yields ranging from 30 to 92%.^9^ Another report by Hoffman and coworkers showed that anhydrous CuSO_4_ could dehydrate neat alcohols to their corresponding olefins (120–160 °C), with styrene being produced in 65% yield at 120 °C.^10^

In 2021, our group demonstrated that Fe­(^py^FOX)­(OTf)_2_ can dehydrate 1-phenylethanol to styrene in up to 74% yield at moderate temperatures.^11^ (^py^FOX = fused meso-dipyridylbis-oxazolidine) This led us to investigate other metal complexes, and in 2024, we reported a highly active Cu­(^py^FOX)­(OTf)_2_ catalyst that dehydrated 1-phenylethanol to styrene in >95% yield, along with other activated alcohols such as benzylic, tertiary, and allylic alcohols.^12^ Secondary alcohols were not dehydrated. Here, we report a new fused-oxazolidine-based ligand with quinoline side arms that provides unique steric and electronic properties. Several corresponding metal complexes were synthesized, and it was found that the copper-quinoline FOX catalyst can dehydrate secondary alcohols such as cycloheptanol and cyclooctanol in good yields while retaining its kinetic reactivity similar to its precursor copper-pyridine-FOX catalyst.

## Results and Discussion

### Syntheses of Quinoline-Based Fused Oxazolidine (^Q^FOX) Ligands

The synthesis of quinoline-based FOX ligands followed a synthetic sequence similar to that of the pyridine-FOX ligands, as shown in Scheme 1. Reaction of tris­(hydroxymethyl)­aminomethane and 2-quinolinecarboxaldehyde in the presence of a catalytic amount of acetic acid gave access to the racemic isomer (*rac-* ^Q^FOX). The racemic isomer was then isomerized to the corresponding *meso*-quinoline FOX (*meso-* ^Q^FOX) in the presence of catalytic AlCl_3_. Both the *rac-* and the *meso-* isomer were characterized by single-crystal X-ray crystallography (Figure 1).

**Scheme 1:**
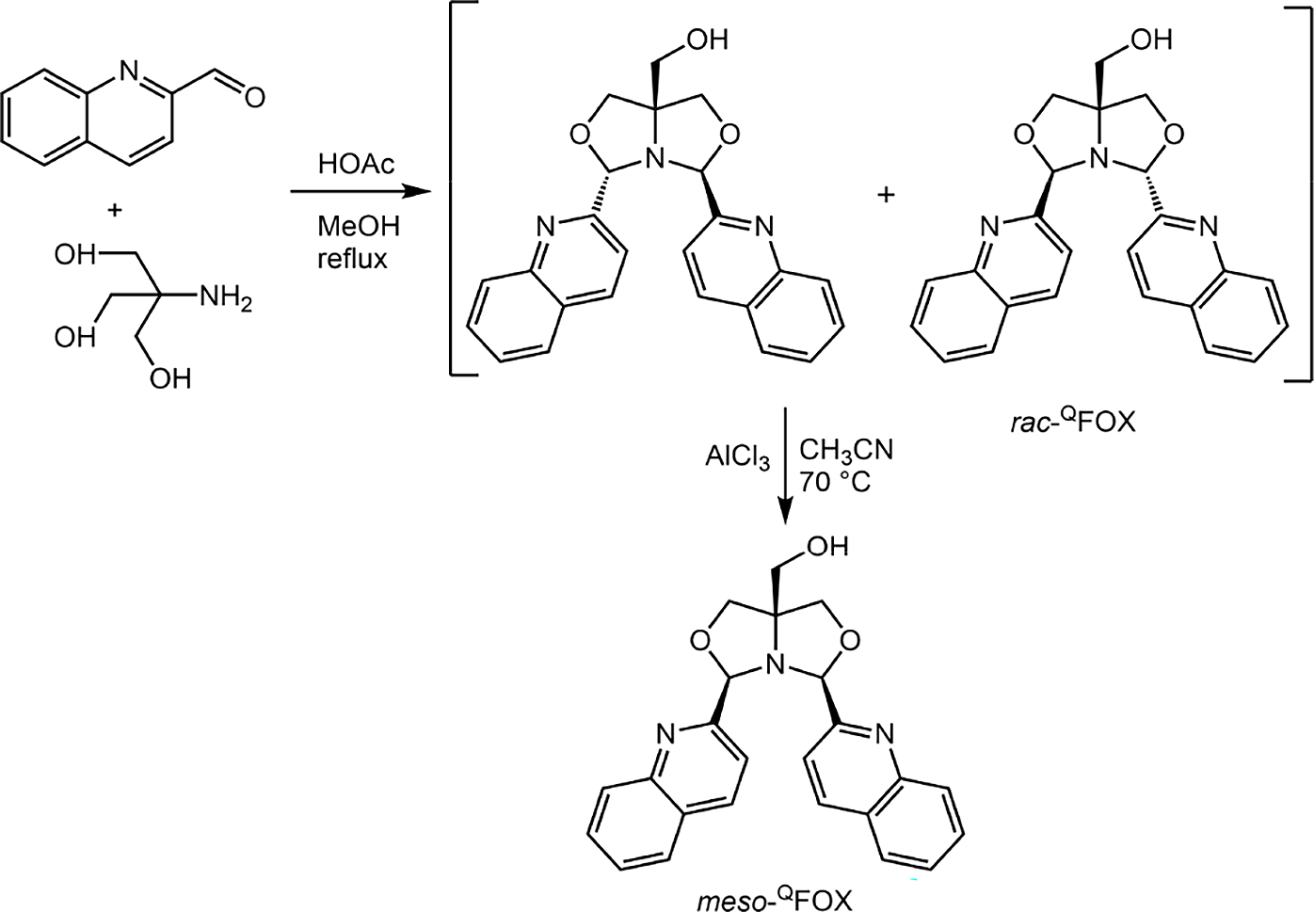
Synthesis of Quinoline FOX (^Q^FOX) Ligand

**Figure 1:**
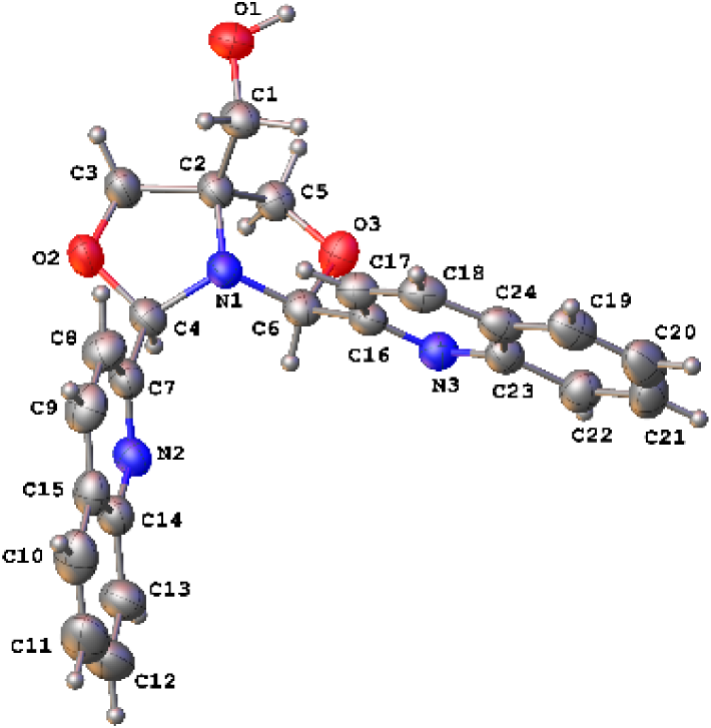
Structure of the *meso*-^Q^FOX ligand. Ellipsoids are shown at the 50% probability level.

### Preparation of First-Row (^Q^FOX)­M^II^ Complexes

In previous attempts to synthesize metal triflate complexes via a direct reaction between M­(OTf)_2_ and FOX derivatives, crystallization of the crude reaction product in Et_2_O/MeCN led to the isolation of a mixture of the desired product contaminated with M­(OTf)_2_. An alternative synthetic route was implemented to avoid contamination (Scheme 2). In this synthetic route, the reaction of MBr_2_ with ^Q^FOX produced M­(^Q^FOX)­Br_2_ (M = Mn, Fe, Co, Ni, Cu, **1a**–**e**), which could be easily isolated as it formed as a precipitate in MeCN. Moreover, further reaction with AgOTf was more efficient due to the precipitation of AgBr. The resulting M­(^Q^FOX)­(OTf)_2_ complexes (**2a**–**e**) were isolated from the solution by crystallization (see Supporting Information).

**Scheme 2:**
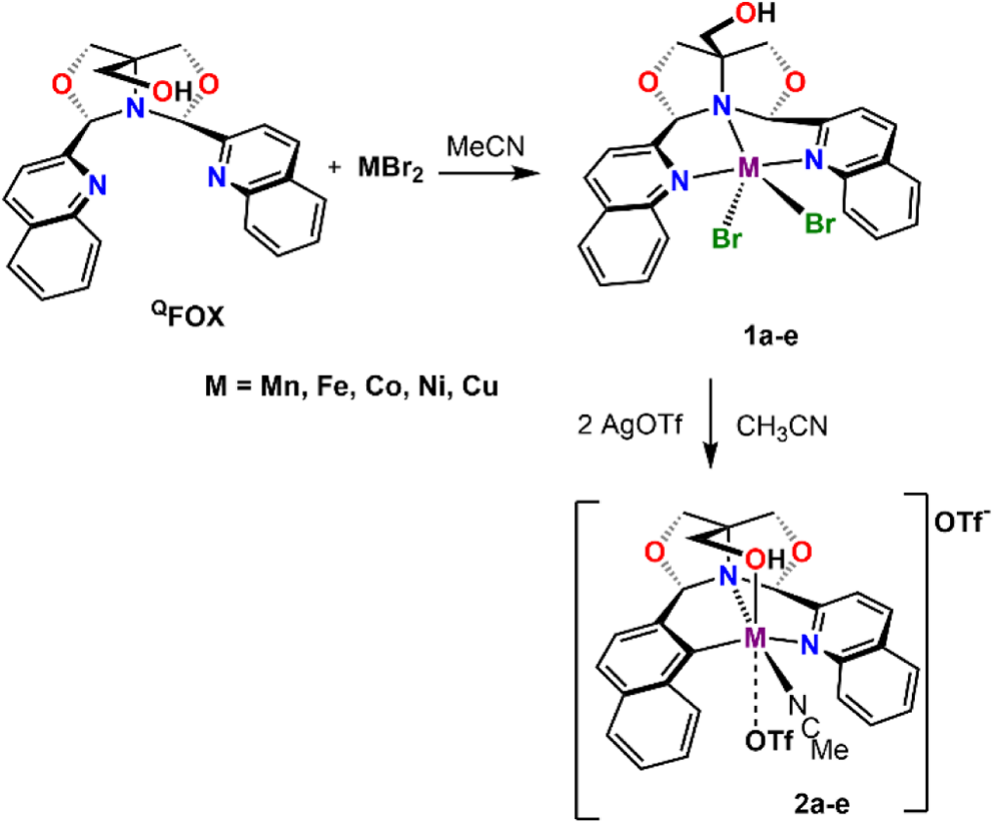
Synthesis of Metal­(II) Triflate Complexes

Magnetic moments for compounds **2a**–**e** were determined using the Evans method.^13^ The compounds were dissolved in methanol containing trifluorotoluene, with a capillary containing methanol/trifluorotoluene, and the separation of the ^19^F singlets was used for the determination of μ_eff_. Values are given in Table 1. All complexes are high-spin M­(II), with the appropriate number of aligned electrons.

**Table 1:** Magnetic Moments for **2a**–**f** (Evans Method)

Cmpd	μ_eff_	# unpaired electrons
**2a** (Mn^II^)	6.21	5
**2b** (Fe^II^)	5.44	4
**2c** (Co^II^)	5.02	3
**2d** (Ni^II^)	3.31	2
**2e** (Cu^II^)	2.00	1

Single-crystal X-ray structures were obtained for eight compounds: compounds **1a**, **1c**, **1d**, and **2a**–**e**. The structures of dibromides **1** all have a κ^3^-*NNN*-^Q^FOX ligand with a dangling CH_2_OH group. They are trigonal bipyramidal with apical quinolines. Triflate complexes **2a**–**2d** are octahedral with a κ^4^-^Q^FOX ligand, while **2e** (M = Cu) is square pyramidal. The structure of **2a** shows two triflates coordinated to manganese, while **2b**–**2d** have one triflate (trans to OH) and one acetonitrile (trans to tertiary nitrogen) bound to the metal. The structure of complex **2e** is representative and is shown in Figure 2. The copper complex has two outer-sphere triflate ions and a bound acetonitrile, with the site opposite the hydroxymethyl vacant (O4 is 2.58 Å from Cu). These coordination changes are expected because, as one moves across the first row, there is a periodic decrease in the metal ionic radius, which limits the binding of the bulky triflate ions (2 to 1 to 0). This is reflected in the metal–ligand bond lengths, which show a general contraction across the row (Table 2). The size of [Cu­(^Q^FOX)­(MeCN)]^2+^ is too small to accommodate a bound triflate coordinated to the metal center. Furthermore, **2e** has a 19-electron count and does not require additional ligation. The two quinoline groups are very nearly coplanar in the copper complex (6.3°). Also, the copper–nitrogen distances are significantly smaller than in the other complexes, indicating higher Lewis acidity. Our previous studies showed that ^py^FOX compounds are good Lewis acid dehydration catalysts, with Cu­(II) showing superior activity.^12^ Hence, copper complex **2e** is the most Lewis acidic and has good potential as a dehydration catalyst.

**Figure 2:**
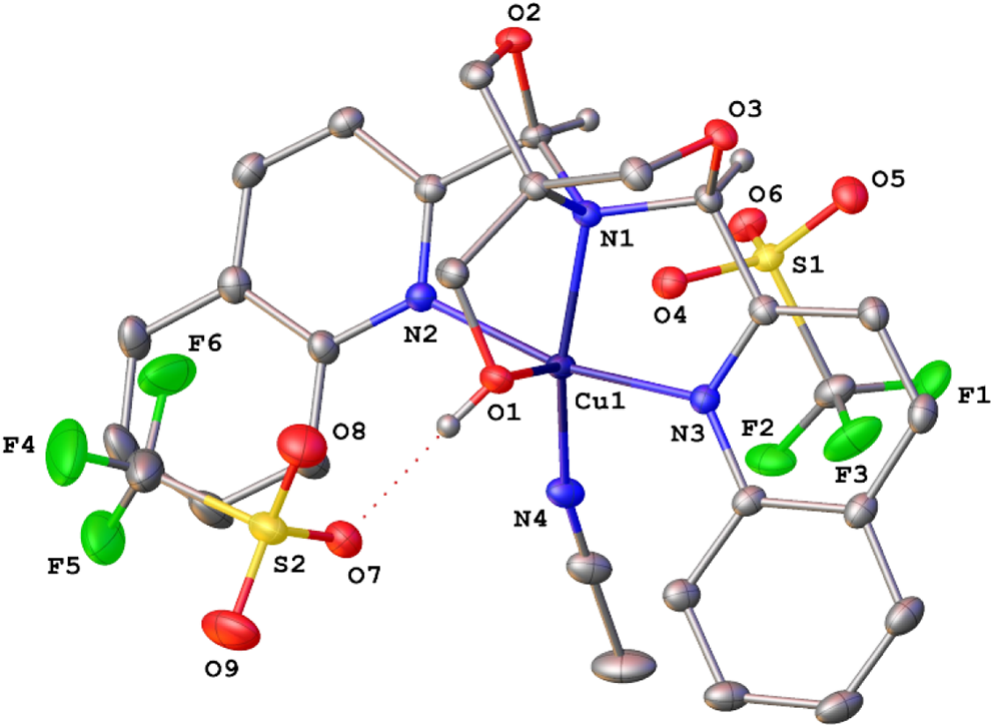
X-ray structure of [Cu­(^Q^FOX)­(MeCN)]­[OTf]_2_ (**2e**). Ellipsoids are shown at the 50% probability level.

**Table 2:** Metal-Ligand Bond Distances for Compounds **2a–e**

Cmpd	M(1)-O(1)^a^	M(1)-O(4)^b^	M(1)-N(1)^c^	M(1)-N(2)	M(1)-N(3)	M(1)-N(4)^d^	*Q*–*Q* angle,^e^ deg
**2a**	2.2841(10)	2.1098(10)	2.2089(11)	2.2771(11)	2.2196(11)	2.2699(11)^d^	12.1
**2b**	2.1395(13)	2.1490(12)	2.1946(14)	2.2079(14)	2.1875(15)	2.1509(15)	1.5
**2c**	2.1080(12)	2.1854(11)	2.1497(13)	2.1332(13)	2.1589(13)	2.1035(14)	28.8
**2d**	2.0660(19)	2.1320(19)	2.037(2)	2.145(2)	2.108(2)	2.011(2)	22.9
**2e**	2.3724(12)	(2.579)^f^	2.0151(13)	1.9999(14)	1.9898(13)	1.9857(14)	6.3

a O­(1) = hydroxymethyl oxygen.

b O­(4) = triflate oxygen.

c N­(1) = tertiary nitrogen.

d N­(4) = acetonitrile nitrogen, except in **2a**, where triflate replaces CH_3_CN.

e Angle between planes of quinoline rings.

f Weakly interacting triflate in **2e**.

A Cu­(I) analog of **2e** was synthesized by reaction of ^Q^FOX with CuBr, followed by AgOTf. A single-crystal X-ray structure of the compound (**2f**) showed κ^3^-*NNN* coordination with a coordinated MeCN ligand and a dangling CH_2_OH group. The metal center is 0.94 Å above the NNN plane, with the acetonitrile occupying an axial position (Figure 3). The geometry of **2f** lies somewhere between tetrahedral and trigonal pyramidal, closer to *T*_d_ (τ_4_ = 0.80).^14^

**Figure 3:**
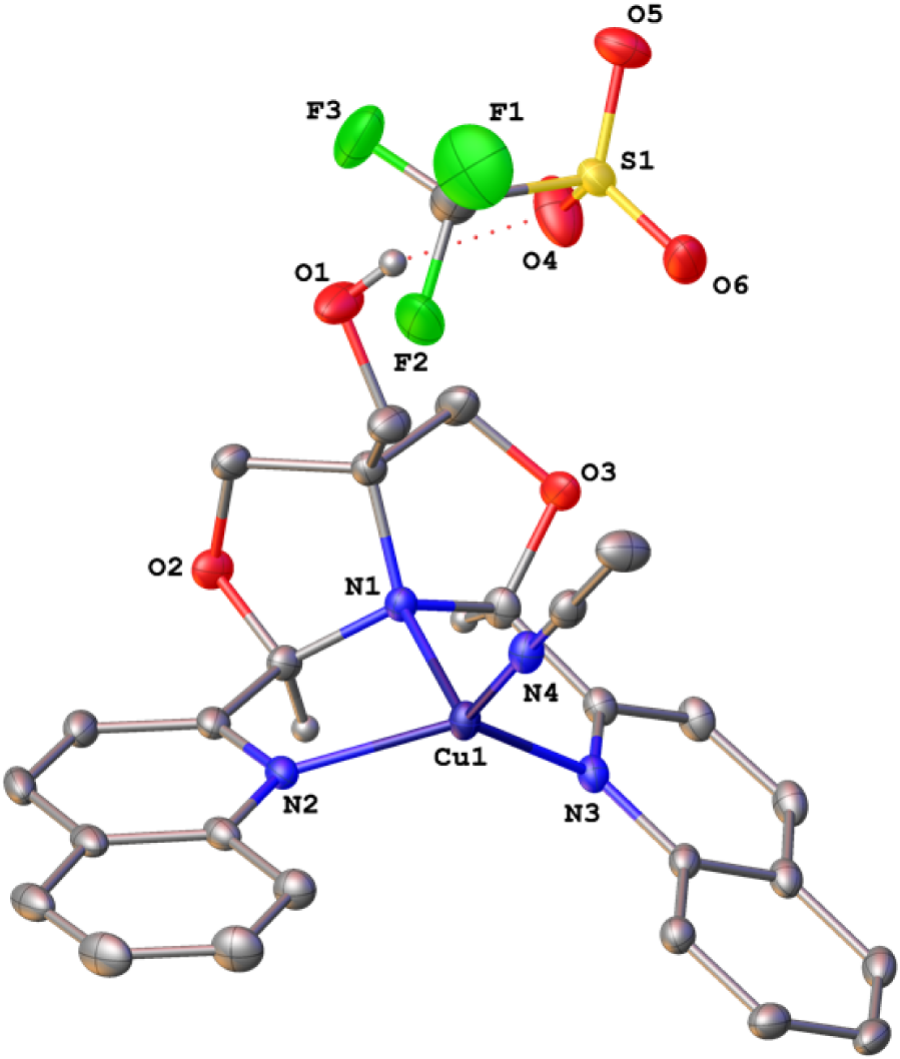
X-ray structure of [Cu­(^Q^FOX)­(CH_3_CN))]­[OTf] (**2f**). Ellipsoids are shown at the 50% probability level.

### Dehydration with ^Q^FOX Bis-Triflate Complexes

Initial screening of the different metal ^Q^FOX bis-triflate complexes under conditions similar to those of the ^py^FOX catalysts revealed strong solvent-dependent effects (Scheme 3). Table 3 shows the dehydration reactions of 1-phenylethanol in toluene solvent, in which iron, nickel, and copper ^Q^FOX catalysts showed >75% consumption of the starting material. For the iron and copper catalysts, the product distribution was dominated by α-methylbenzyl ethers (**E**) instead of styrene (**S**). The formation of **E**, although not the primary target, was shown to be reversible with the reported iron and copper catalysts.^11,12^ It was thus expected that the formation of **E** was reversible in this similar catalytic system and can be converted into **S** under appropriate reaction conditions. Very little of the styrene dimer **D** is observed with M­(^Q^FOX) catalysts, in contrast to Fe­(^py^FOX) and Cu­(^py^FOX) catalysts.

**Scheme 3:**
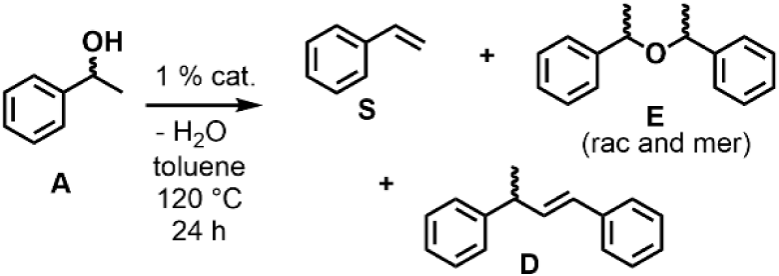
Reaction Conditions for Dehydration of 1-Phenylethanol

**Table 3:** Dehydration of 1-Phenylethanol in Toluene using Different [M­(^Q^FOX)]­(OTf)_2_ Catalysts^a^

	% Composition				
catalyst	M	**A**	**S**	**E**	**D**
**2a**	Mn^II^	91	1	7	<1%
**2b**	Fe^II^	12	16	63	<1%
**2c**	Co^II^	68	4	29	<1%
**2d**	Ni^II^	3	38	25	<1%
**2e**	Cu^II^	28	12	57	<1%

a Reactions were carried out at 120 °C in toluene with 1% catalyst, 0.83 M 1-phenylethanol, for 24 h. About 9% acetophenone is seen in these reactions; about 3% acetophenone was present in the starting material.

Moreover, this reactivity of the copper ^Q^FOX was observed only in *ortho*-dichlorobenzene (ODCB) solvent, which is in stark contrast to the copper ^py^FOX complex. Other solvents, including THF, acetonitrile, DMSO, and dioxane, showed little reaction of phenylethanol. It was noticed that the catalyst was partially soluble in ODCB initially. Upon addition of alcohol and heating, the reaction mixture became homogeneous. The catalyst was reisolated at the end of the reaction, and crystallization showed it to be the aquo-substituted complex [Cu­(^Q^FOX)­(H_2_O)]­[OTf]_2_, **3** (Figure 4).

**Figure 4:**
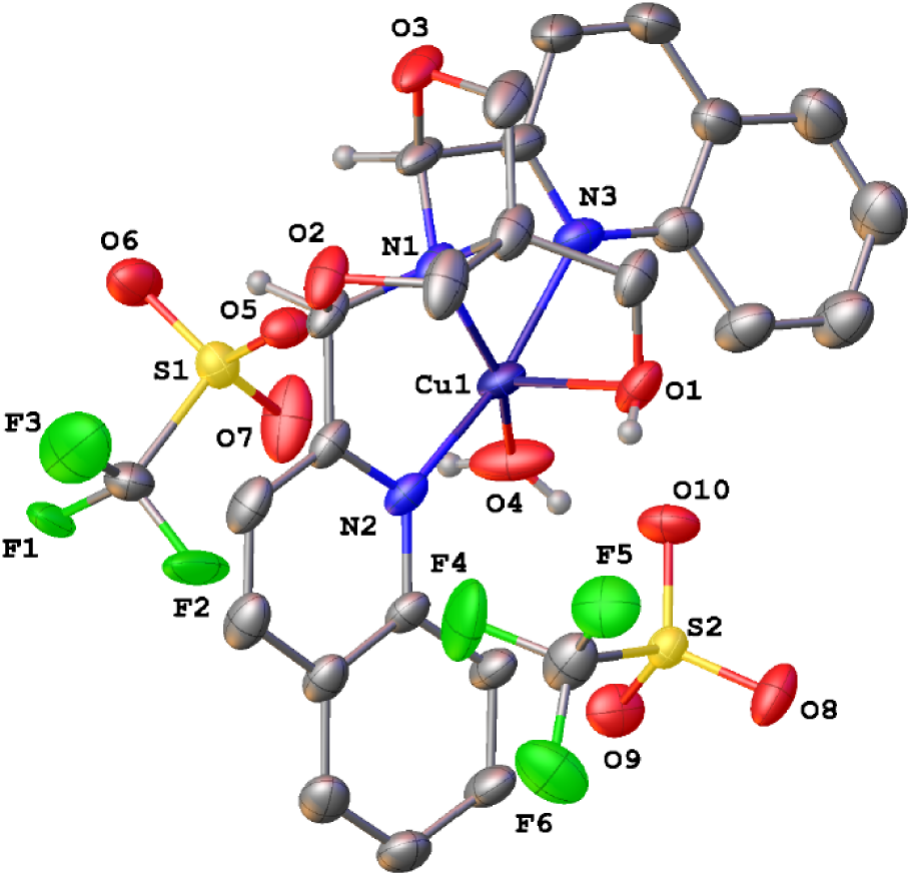
X-ray structure of [Cu­(^Q^FOX)­(H_2_O))]­[OTf]_2_ (**3**). Ellipsoids are shown at the 50% probability level.

In order to find the most active catalyst for the dehydration of 1-phenylethanol, all of the catalysts were screened under various reaction conditions. It was found that copper catalyst **2e** gave the highest yield of **S** (84%), with negligible amounts of ethers after 24 h (Table 4). The Cu^I^ catalyst **2f** showed reduced dehydration activity compared to the Cu^II^ catalyst **2e**.^15^

**Table 4:** Dehydration of 1-Phenylethanol in *o*-Dichlorobenzene using Different M­(^Q^FOX)]­(OTf)_2_ Catalysts^a^

	% Composition				
catalyst	M^n+^	**A**	**S**	**E**	**D**
**2a**	Mn^II^	87	5	10	<1%
**2b**	Fe^II^	80	7	12	<1%
**2c**	Co^II^	68	10	7	<1%
**2d**	Ni^II^	100	0	0	<1%
**2e**	Cu^II^	0	84	1	<1%
**2f**	Cu^I^	37	3	58	<1%

a Reactions were carried out at 120 °C in ODCB with 1% catalyst, for 24 h, 0.83 M 1-phenylethanol. About 9% acetophenone is seen in these reactions; about 3% acetophenone was present in the starting material.

Investigation of the distribution of species of the reaction at 120 °C was carried out by monitoring via ^1^H NMR spectroscopy using 0.55 M 1-phenylethanol and 1% **2e** in ODCB-*d*_4_ in sealed NMR tubes.^16^ Astonishingly, only styrene and trace amounts of styrene dimer were observed after 13 h of reaction. Monitoring the reaction periodically showed that the α-methylbenzyl ethers were formed (along with styrene) but were converted to styrene within 6 h. At 8 h, the reaction was complete with an 88% yield of styrene. Further heating led to styrene dimerization and further polymerization to polystyrene (Figure 5).

**Figure 5:**
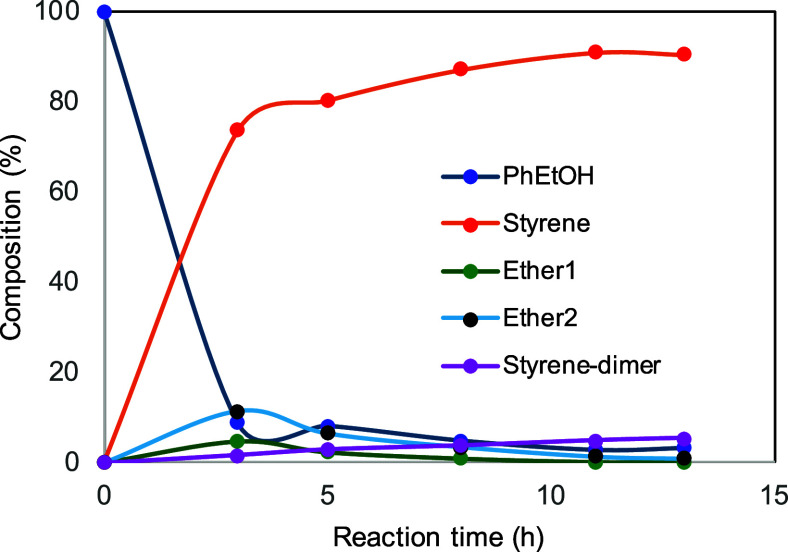
Distribution of species by ^1^H NMR spectroscopy in ODCB-*d*_4_, 0.59 M 1-phenylethanol, 1% **2e**, at 120 °C.

To investigate further the reactivity differences in the new ^Q^FOX system, the distribution of species for the dehydration of 1-phenylpropanol was examined. Similar to the 1-phenylethanol kinetics, the initial phase of the reaction showed the formation of the two types of α-methylbenzyl ethers, which later are re-entrained to give the styrenes.^17^ After 18 h, 82% of the more stable trans-β-methylstyrene and 7% of the less stable cis-β-methylstyrene, along with some polystyrene, were observed (Figure 6). Traces (<4%) of styrene dimer were observed at 18 h. Additionally, *tert*-butyl alcohol was dehydrated to isobutylene in 84% yield after 48 h without the formation of any di-*tert*-butyl ether, as determined by ^13^C NMR spectroscopy.

**Figure 6:**
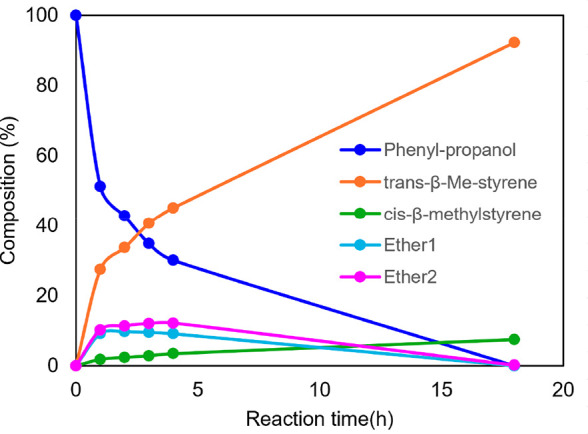
Plot of ^1^H NMR data for conversion of 1-phenylpropanol (0.55 M) to β-methylstyrenes in toluene-*d*_8_ at short reaction times. Traces of styrene dimer were observed as byproducts of the reaction after 18 h. *T* = 120 °C.

### Comparative Reactivities: Copper-^Q^FOX vs -^py^FOX

Our previous studies with the [(^py^FOX)­Cu­(CH_3_CN)]­(OTf)_2_ complex^12^ showed that it is an excellent catalyst for dehydration of tertiary, benzylic, and allylic alcohols, but that it was unable to dehydrate secondary or primary alcohols. Toluene was the optimal solvent. Hence, with the copper ^Q^FOX catalyst, we wanted to investigate the role of sterics and electronics on any changes in reactivity. Comparisons of the two catalysts at the same reaction times and temperatures were made with several substrates (Scheme 4). With **2e** as catalyst, ODCB proved to be the optimal solvent, and it was observed that the reaction of cyclopentanol and cyclohexanol for 48 h led to consumption of 65% of the starting material, yielding 41% of cyclopentene and 36% of cyclohexene, respectively. Interestingly, it was observed that cycloheptanol and cyclooctanol were dehydrated completely after 48 h, yielding 71% cycloheptene and 80% cyclooctene, respectively. This reactivity, which is partially dependent on the introduction of the quinoline moiety, was not observed in the corresponding copper pyridine-FOX catalyst, where these unactivated secondary alcohols showed no significant reactivity. Several other comparisons are shown in Scheme 4. 1-Methylcyclohexanol was dehydrated to 1-methylcyclohexene in 90% yield, with none of the exocyclic double-bond product methylenecyclohexene observed. Similarly, 2-cyclohexen-1-ol was dehydrated selectively to 1,3-hexadiene in 92% yield. Allylic alcohols are readily dehydrated with catalyst **2e**. In contrast to the 1-phenylethanol substrate, no ethers or olefin dimers were seen with any of these substrates.

**Scheme 4:**
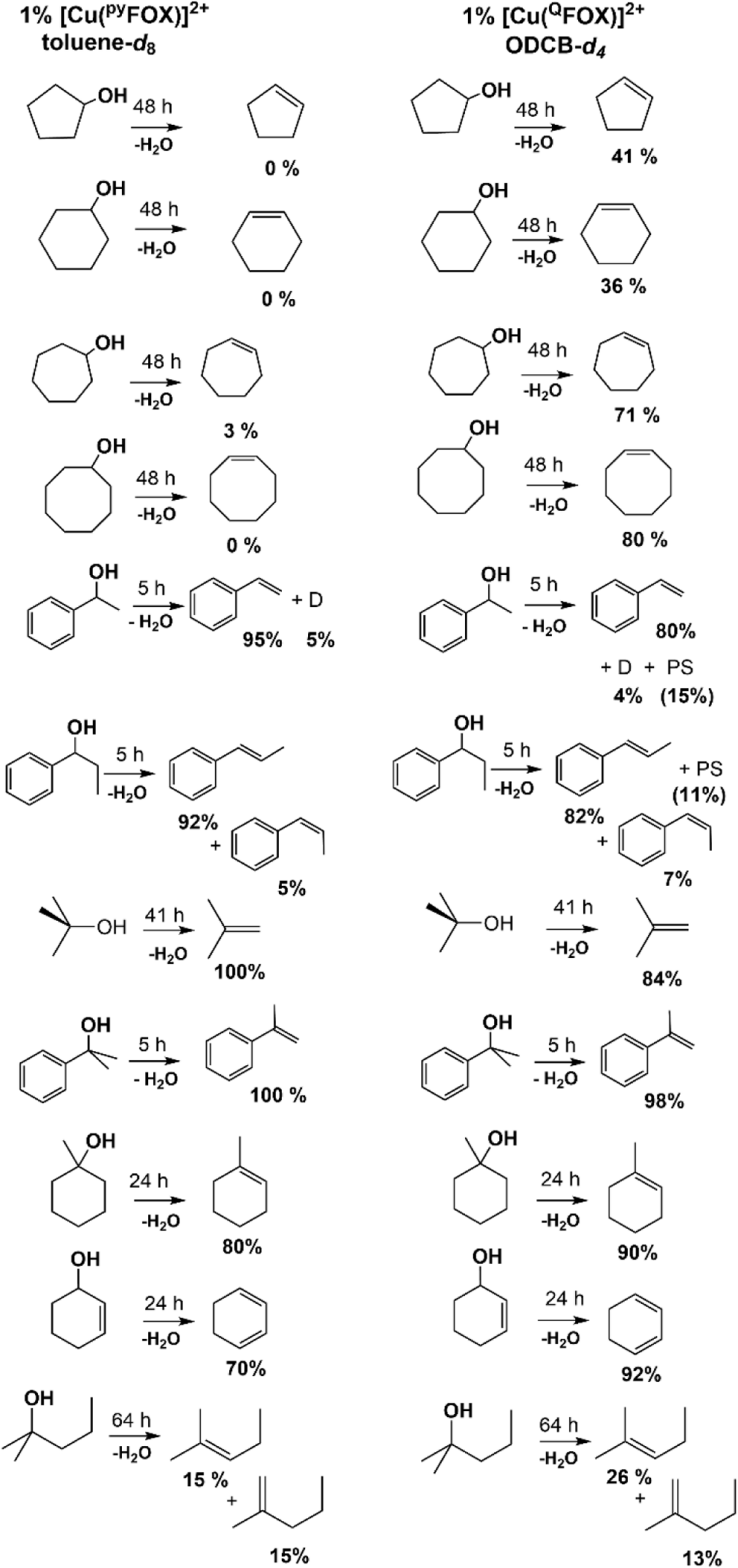
Dehydration of Alcohols at 120 °C: ^py^FOX vs ^Q^FOX

The mechanism for these dehydrations follows that proposed for the [(^py^FOX)­Cu­(CH_3_CN)]­(OTf)_2_ complex (Scheme 5).^12^ Replacement of the acetonitrile in **2e** by alcohol (i) is followed by dissociation of the carbonium ion (ii) and formation of a copper hydroxide species. The carbonium ion can transfer a β-hydrogen to this hydroxide, generating the aquo complex **3** and the corresponding olefin (iii). Complex **3** then undergoes exchange of water for an alcohol, and the cycle continues. Alternatively, in the case of R = phenyl, the carbonium ion can react with more alcohol to give the α-methylbenzyl ethers plus **3** (iv), or react with styrene to give dimer plus **3** (v). The higher reactivity of the Cu­(^Q^FOX) catalyst compared to the Cu­(^py^FOX) catalyst could be due to a lower-energy LUMO in the quinoline ligand, which increases the Lewis acidity of copper through π-backbonding. Catalyst **2e**, with its bis-quinoline ligation, forms a cavity in which the carbonium ion is formed and subsequently reacts. This may contribute to the differing effects of solvent on the reactivity of the (^Q^FOX)Cu vs (^py^FOX)Cu catalysts.

**Scheme 5:**
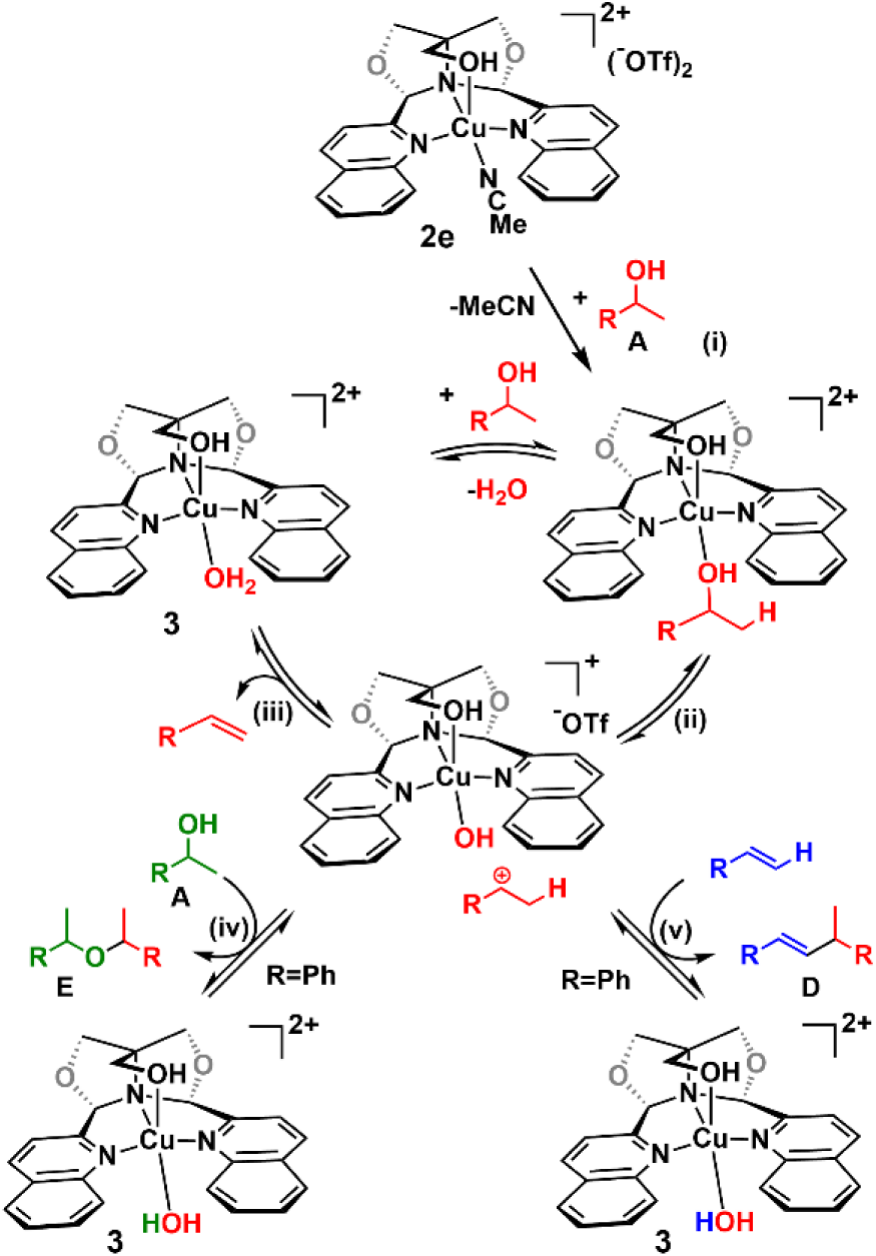
Proposed Dehydration Mechanism

## Conclusions

The new catalyst [(^Q^FOX)­Cu­(CH_3_CN)]­(OTf)_2_ (**2e**) has been found to be very active for alcohol dehydration. Styrene is formed from 1-phenylethanol, and tertiary and allylic alcohols are readily dehydrated. In contrast to the analogous ^py^FOX catalyst, **2e** also dehydrates unactivated secondary alcohols. The higher reactivity of this catalyst compared to that of the ^py^FOX catalyst indicates increased Lewis acidity in the ^Q^FOX catalysts. Additional studies are underway to determine what other reactions the intermediate carbonium ions might undergo.

## Experimental Section

All commercial reagents were used as received without further purification. All air- and moisture-sensitive manipulations were performed using standard Schlenk techniques or in an inert atmosphere of purified nitrogen. Diethyl ether, toluene, and acetonitrile were used directly from an Innovative Technology, Inc. PS-MD-6 solvent purification system.

Elemental compositions were determined by using a PerkinElmer 2400 Series II analyzer. Nuclear magnetic resonance data were recorded on Bruker Avance 400 and Avance 500 spectrometers or a Jeol 500 spectrometer. GC–MS data were recorded on a Hewlett-Packard 5890 GC Series II instrument equipped with a Hewlett-Packard 5970 Series mass-selective detector. Quantitative GC was performed on a Shimadzu GC-2010 instrument equipped with a flame ionization detector and an autosampler. A Rigaku Synergy-S diffractometer with dual PhotonJet-S microfocus X-ray sources (Cu Kα, Mo Kα) and a HyPix-6000HE HPC detector was used for crystallographic structure determinations. NMR spectra and chromatograms are provided in the Supporting Information.

μ_eff_ values for complexes **2a**–**e** were determined using the Evans method. A known mass of the compound was dissolved in CH_3_OH containing trifluorotoluene and placed in an NMR tube along with a sealed capillary containing trifluorotoluene/methanol. The shift in the CF_3_ group ^19^F resonance was measured and used to determine μ_eff_.

### Synthesis of Racemic Quinoline FOX (*rac*-^Q^FOX) Ligand

To a clean, dry 250 mL round-bottom flask, tris­(hydroxymethyl)­aminomethane (5.0 g, 41.3 mmol) and quinoline-2-carbaldehyde (13.0 g, 124 mmol) were dissolved in methanol (80 mL). Acetic acid (236 μL) was added, and the stirred solution was refluxed for 2 h and then kept at room temperature overnight. Methanol was then removed under reduced pressure. The crude product was then recrystallized from hot methanol layered with ether to afford 14.1 g of product as a white crystalline solid (86%).

^1^H NMR (400 MHz, DMSO-*d*_6_) δ 8.31 (d, *J* = 8.5 Hz, 1H), 8.17 (d, *J* = 8.5 Hz, 1H), 7.84 (d, *J* = 8.1 Hz, 1H), 7.78–7.69 (m, 2H), 7.63 (d, *J* = 8.6 Hz, 1H), 7.43–7.22 (m, 4H), 7.09 (d, *J* = 8.4 Hz, 1H), 6.83 (d, *J* = 8.3 Hz, 1H), 5.63 (s, 1H), 5.24 (t, *J* = 5.4 Hz, 1H), 5.16 (s, 1H), 4.37 (d, *J* = 8.7 Hz, 1H), 4.19 (d, *J* = 8.7 Hz, 1H), 3.85 (dd, *J* = 17.8, 8.8 Hz, 2H), and 3.75 (ddd, *J* = 34.6, 10.8, 5.6 Hz, 2H).

^13^C­{^1^H} NMR (101 MHz, DMSO-*d*_6_) δ: 160.3, 155.6, 147.8, 146.8, 137.7, 136.9, 130.2, 129.7, 129.3, 129.2, 128.7, 128.6, 128.4, 127.7, 127.3, 121.1, 120.3, 95.5, 94.8, 75.9, 75.3, 73.6, 66.3 (1 C obscured).

### Synthesis of Meso Quinoline FOX (*meso*-^Q^FOX) Ligand

In a nitrogen atmosphere, a 250 mL round-bottom flask was charged with *rac-* ^Q^FOX (10.0 g, 35.4 mmol) and anhydrous AlCl_3_ (334 mg, 3.54 mmol) in 150 mL of CH_3_CN. The stirred solution was heated to 60 °C for 2 h and kept at room temperature overnight. All solvent was removed under reduced pressure. The crude product was extracted with CH_2_Cl_2_ (3 × 20 mL). Insoluble impurities were removed by filtration. Silica (∼10 g) was added to the solution to absorb the product. The slurry was dried under vacuum, and the product was isolated through column chromatography (MeOH/CH_2_Cl_2_, 0:100 to 2:98). Further recrystallization from hot ethyl acetate layered with hexanes afforded 5.13 g of product (51%) as a colorless crystalline solid.

^1^H NMR (500 MHz, DMSO-*d*_6_) δ: 8.28 (d, *J* = 8.6 Hz, 2H), 7.89 (d, *J* = 8.5 Hz, 4H), 7.69 (t, *J* = 7.6 Hz, 2H), 7.60 (d, *J* = 8.5 Hz, 2H), 7.54 (t, *J* = 7.6 Hz, 2H), 5.81 (s, 2H), 5.22 (t, *J* = 5.4 Hz, 1H), 4.21 (d, *J* = 8.7 Hz, 2H), 3.93 (d, *J* = 8.7 Hz, 2H), 3.48 (d, *J* = 5.4 Hz, 2H).

^13^C­{^1^H} NMR (101 MHz, DMSO-*d*_6_) δ: 159.9, 146.9, 137.2, 130.1, 129.1, 128.2, 127.8, 127.1, 119.6, 98.3, 75.4, 73.0, 65.7.

Anal. Calcd (found) for C_24_H_21_N_3_O_3_: 72.17(71.70) %C, 5.30(5.27) %H, and 10.52(10.33) %N.

### General Procedure for the Synthesis of M­(^Q^FOX)­Br_2_

The procedure used here to prepare **1e** is representative of the other metals. To a clean, dry 20 mL scintillation vial in a nitrogen-filled glovebox, ^Q^FOX (100 mg, 0.33 mmol) and CuBr_2_ (82 mg, 0.37 mmol) were dissolved in 5 mL of anhydrous acetonitrile. The reaction mixture was stirred for 30 min to precipitate a pale blue solid. The solid was collected by filtration, washed with anhydrous diethyl ether (6 mL), and dried in vacuo to afford a pale blue solid **1e** (148 mg, 0.284 mmol, 84%). Single crystals of the product were obtained by layering a saturated methanol solution of the complex with diethyl ether on the benchtop. The compounds are all paramagnetic, and NMR data were not used for characterization. EAs were obtained for the crude, unrecrystallized products.

**1a:** 0.191 g, 94% yield. Anal. Calcd (found) for C_24_H_21_Br_2_Mn_3_N_3_O_3_: 46.93 (46.83) %C, 3.45 (3.17) %H, 6.84 (7.92) %N.

**1b:** 0.139 g, 70% yield. Anal. Calcd (found) for C_24_H_21_Br_2_FeN_3_O_3_: 46.86 (45.08) %C, 3.44 (3.35) %H, 6.83 (6.61) %N.

**1c:** 0.159 g, 80% yield. Anal. Calcd (found) for C_24_H_21_Br_2_CoN_3_O_3_: 46.63 (46.80) %C, 3.42 (3.23) %H, 6.80 (7.95) %N.

**1d:** 0.141 g, 71% yield. Anal. Calcd (found) for C_24_H_21_Br_2_NiN_3_O_3_: 46.65 (45.01) %C, 3.43 (3.58) %H, 6.80 (7.79) %N.

**1e:** 168 g, 84% yield. Anal. Calcd (found) for C_24_H_21_Br_2_CuN_3_O_3_: 46.28 (46.22) %C, 3.40 (3.31) %H, 6.75 (6.78) %N.

### General Procedure for the Synthesis of [M­(^Q^FOX)­(MeCN)]­[OTf]_2_

The procedure used here to prepare **2e** is representative of the other metals. To a clean, dry 20 mL scintillation vial in a nitrogen-filled glovebox, Cu­(^Q^FOX)­Br_2_ (100 mg, 0.16 mmol) and AgOTf (98.3 mg, 0.38 mmol) were dissolved in 5 mL of anhydrous acetonitrile. The reaction mixture was stirred for 30 min to obtain a clear, blue solution with the precipitation of a yellow solid (AgBr). The yellow solid was discarded, and the blue solution was reduced in vacuo and layered with anhydrous diethyl ether to afford single crystals of **2e** (107 mg, 0.15 mmol, 80%). The compounds are all paramagnetic, and NMR data were not of use for characterization.

**2a**: 0.053 g, 43% yield. [(^Q^FOX)­Mn­(OTf)_2_]­(NCMe) Anal. Calcd (found) for C_26_H_21_F_6_N_3_MnO_9_S_2_: 41.50 (40.99) %C, 2.81 (2.78) %H, 5.58 (5.38) %N. μ_eff_ = 6.21.

**2b**: 0.099 g, 75% yield. [(^Q^FOX)­Fe­(NCMe)­(OTf)]­[OTf]. Anal. Calcd (found) for C_28_H_24_F_6_FeN_4_O_9_S_2_: 42.33(42.85) %C, 3.05 (3.45) %H, 7.05 (6.89) %N. μ_eff_ = 5.44

**2c**: 0.106 g, 82% yield. [(^Q^FOX)­Co­(NCMe)­(OTf)]­[OTf]. Anal. Calcd (found) for C_28_H_24_F_6_N_4_CoO_9_S_2_: 42.17 (42.26) %C, 3.03 (2.82) %H, 7.02 (7.89) %N. μ_eff_ = 5.02

**2d**: 0.067 g, 48% yield. [(^Q^FOX)­Ni­(NCMe)­(OTf)]­[OTf]-(CH_3_CN). Anal. Calcd (found) for C_30_H_27_F_6_N_5_NiO_9_S_2_: 42.98 (42.59) %C, 3.25 (3.12) %H, 8.35 (7.56) %N. μ_eff_ = 3.31

**2e**: 0.106 g, 80% yield. [(^Q^FOX)­Cu­(NCMe)]­[OTf]_2_. Anal. Calcd (found) for C_28_H_24_CuF_6_N_4_O_9_S_2_: 41.92 (41.44) %C, 3.02 (2.84) %H, 6.98 (6.56) %N. μ_eff_ = 2.00

### Synthesis of [Cu­(QFOX)­(MeCN)]Br (**1f**)

To a clean, dry 20 mL scintillation vial in a nitrogen-filled glovebox, ^Q^FOX (152 mg, 0.5 mmol) was reacted with anhydrous CuBr (72 mg, 0.5 mmol) dissolved in 5 mL of anhydrous acetonitrile. The reaction mixture was stirred for 2 h to obtain a yellow solid, which was collected by filtration, washed with diethyl ether, and dried in vacuo to afford a yellow solid (240 mg, 82%).

### Synthesis of [Cu­(QFOX)­(MeCN)]­[OTf] (**2f**)

To a clean, dry 20 mL scintillation vial in a nitrogen-filled glovebox, **[Cu­(** ^**Q**^ **FOX)­(MeCN)]­Br** (100 mg, 0.17 mmol) was reacted with anhydrous AgOTf (44 mg, 0.171 mmol) dissolved in 5 mL of anhydrous acetonitrile. The reaction mixture was stirred for 2 h to obtain a yellow solution with precipitation of AgBr. The yellow solution was then reduced in vacuo and layered with anhydrous diethyl ether to afford orange crystals of **2f** (87 mg, 0.133 mmol, 78%).

### Dehydration Studies with Cu Compounds

To a clean, dry 5 mL Schlenk tube containing 1 mol % of catalyst **2e** (5.8 mg, 0.0083 mmol) and 1 mL of anhydrous toluene (or ODCB), 0.83 mmol (0.1 mL) of 1-phenylethanol was added under a positive pressure of nitrogen. The tube was sealed by using a Teflon stopper. It was then heated in an aluminum heating block at 120 °C for 24 h and subsequently cooled in an ice bath. The contents were then filtered through a 3.5 cm plug of silica and washed with diethyl ether. The filtered contents were transferred to a vial, and 40 μL of *n*-decane was added as an internal standard. 50 μL of this solution was diluted with 1 mL of diethyl ether and then analyzed by GC.

A crystal of the catalyst was isolated at the end of the reaction, and a single-crystal X-ray structure of **3** was obtained, showing a bound water molecule and an outer-sphere triflate.

The same reaction procedure was used for the reactions of other alcohols and with the different catalysts **2a**–**d**. The substrates t-butanol, 2-phenylisopropanol, and 2-methyl-2-pentanol were examined by using NMR spectroscopy. See Supporting Information for GC traces and spectra.

Caution! Heating a closed sample above the boiling point of the solvent or substrate can result in elevated pressure within the vessel. Adequate precautions, such as a blast shield, should be used to protect against potential explosions.

### Kinetic Study of 1-Phenylethanol and Other Alcohols

To a clean, dry J. Young NMR tube containing 1 mol % of catalyst **2** (2.9 mg, 0.0041 mmol) and 0.7 mL of toluene-*d*_8_ was added 0.05 mL (0.414 mmol) of 1-phenylethanol under a nitrogen atmosphere in a glovebag. The tube was then sealed with a Teflon stopper and heated in an aluminum heating block at 120 °C. ^1^H NMR spectra were recorded at various time points to monitor the progress of the reaction. Some experiments were also conducted in sealed NMR tubes.

## Supplementary Material



## Data Availability

The data underlying this study are available in the published article and its online Supporting Information.

## Notes

NMR spectra, tables of distribution of species for reactions, and GC traces and NMR data for reactions in Scheme 4 (PDF); X-ray structure reports for *meso*-^Q^FOX, *rac*-^Q^FOX, **1a**, **1c**, **1d**, **2a–f**, and **3** (2 polymorphs) (PDF); CIF files for X-ray structures *meso*-^Q^FOX, *rac*-^Q^FOX, **1a**, **1c**, **1d**, **2a–f**, **3** (2 polymorphs), **1d’**, and **2d’** (CIF); the X-ray data are also available from the Cambridge Structural Database (CCDC #2504808–2504809, 2493761–2493771). Differently solvated derivatives of **1d** and **2d** are also reported, [Ni­(^Q^FOX)­(CH_3_CN)­Br]Br (**1d’**) and [Ni­(^Q^FOX)­(μ-OTf)]_2_(OTf)_2_, (**2d’**) (CCDC #2497051–2497052) (PDF)
