# Supplementary material for: Advanced Catalysts for Olefin Synthesis: Copper(II) Quinoline-Fused Oxazolidines in Alcohol Dehydration
Source: J Org Chem. 2025 Dec 31;91(2):1136–43. doi: 10.1021/acs.joc.5c02669 (PMC12814556; doi:10.1021/acs.joc.5c02669)
Supplement: Supplementary file 1 [file jo5c02669_si_001.pdf]

Supporting Information

Advanced Catalysts for Olefin Synthesis: Copper(II) Quinoline-Fused Oxazolidines in Alcohol Dehydration

Aurodeep Panda, Ruilin Zhang, William W. Brennessel, and William D. Jones*

Department of Chemistry, University of Rochester, Rochester, NY 14627

Figure S-1. ^{1}H NMR spectrum of *rac*-^{Q}FOX in DMSO-d_6	SI-2
Figure S-2. ^{13}C{^{1}H} NMR spectrum of *rac*-^{Q}FOX in DMSO-d_6	SI-3
Figure S-3. ^{1}H NMR spectrum of *meso*-^{Q}FOX in DMSO-d_6	SI-4
Figure S-4. ^{13}C{^{1}H} NMR spectrum of *meso*-^{Q}FOX in DMSO-d_6	SI-5
Figure S-5. ^{1}H NMR spectrum of α-methylbenzyl ethers	SI-6
Figure S-6. ^{19}F{^{1}H} spectrum of **2a** (Evan's method)	SI-7
Figure S-7. ^{19}F{^{1}H} spectrum of **2b** (Evan's method)	SI-8
Figure S-8. ^{19}F{^{1}H} spectrum of **2c** (Evan's method)	SI-9
Figure S-9. ^{19}F{^{1}H} spectrum of **2d** (Evan's method)	SI-10
Figure S-10. ^{19}F{^{1}H} spectrum of **2e** (Evan's method)	SI-11
Figure S-11. Kinetic data for the dehydration of 1-phenylethanol in ODCB-d_4 using **2e**	SI-12
Figure S-12. Kinetic data for the dehydration of 1-phenylpropanol in toluene-d_8 using **2e**	SI-13
Figures S-13 to S-22. GC chromatograms for reactions in Scheme 4	SI-14
Figures S-23 to S-38. NMR spectra for reactions in Scheme 4	SI-24
Figures S-39 to S-48. Paramagnetic NMR spectra of **1a** – **2e**	SI-40
Screening Reactions:Dehydration of 1-phenylethanol catalyzed by bis-triflate complexes	SI-50
Isolation and Structures of **1a-e**, **1d'**, **2a-e**, and **2d'**	SI-51
X-ray structure determination of *rac*-^{Q}FOX	SI-53
X-ray structure determination of *meso*-^{Q}FOX	SI-68
X-ray structure determination of Mn(^{Q}FOX)Br_2, **1a**	SI-90
X-ray structure determination of Co(^{Q}FOX)Br_2, **1c**	SI-104
X-ray structure determination of Ni(^{Q}FOX)Br_2, **1d**	SI-117
X-ray structure determination of Mn(^{Q}FOX)$(OTf)_2(CH_3CN)$, **2a**	SI-132
X-ray structure determination of Fe(^{Q}FOX)$(CH_3CN)(OTf)_2$, **2b**	SI-151
X-ray structure determination of Co(^{Q}FOX)$(CH_3CN)(OTf)_2$, **2c**	SI-173
X-ray structure determination of Ni(^{Q}FOX)$(CH_3CN)(OTf)_2$, **2d**	SI-190
X-ray structure determination of Cu(^{Q}FOX)$(CH_3CN)(OTf)_2$, **2e**	SI-208
X-ray structure determination of Cu(^{Q}FOX)$(H_2O)(OTf)_2$, **2f**	SI-225
X-ray structure determination of Cu(^{Q}FOX)$(CH_3CN)(OTf)$, **3** (1st polymorph)	SI-246
X-ray structure determination of Cu(^{Q}FOX)$(CH_3CN)(OTf)$, **3** (2nd polymorph)	SI-263
X-ray structure determination of [Ni(^{Q}FOX)(CH_3CN)Br]Br, **1d'**	SI-279
X-ray structure determination of [Ni(^{Q}FOX)(OTf)]$_2(OTf)_2$, **2d'**	SI-295

Figure S-1. ^{1}H NMR spectrum of *rac*-^{Q}FOX in DMSO-d_6

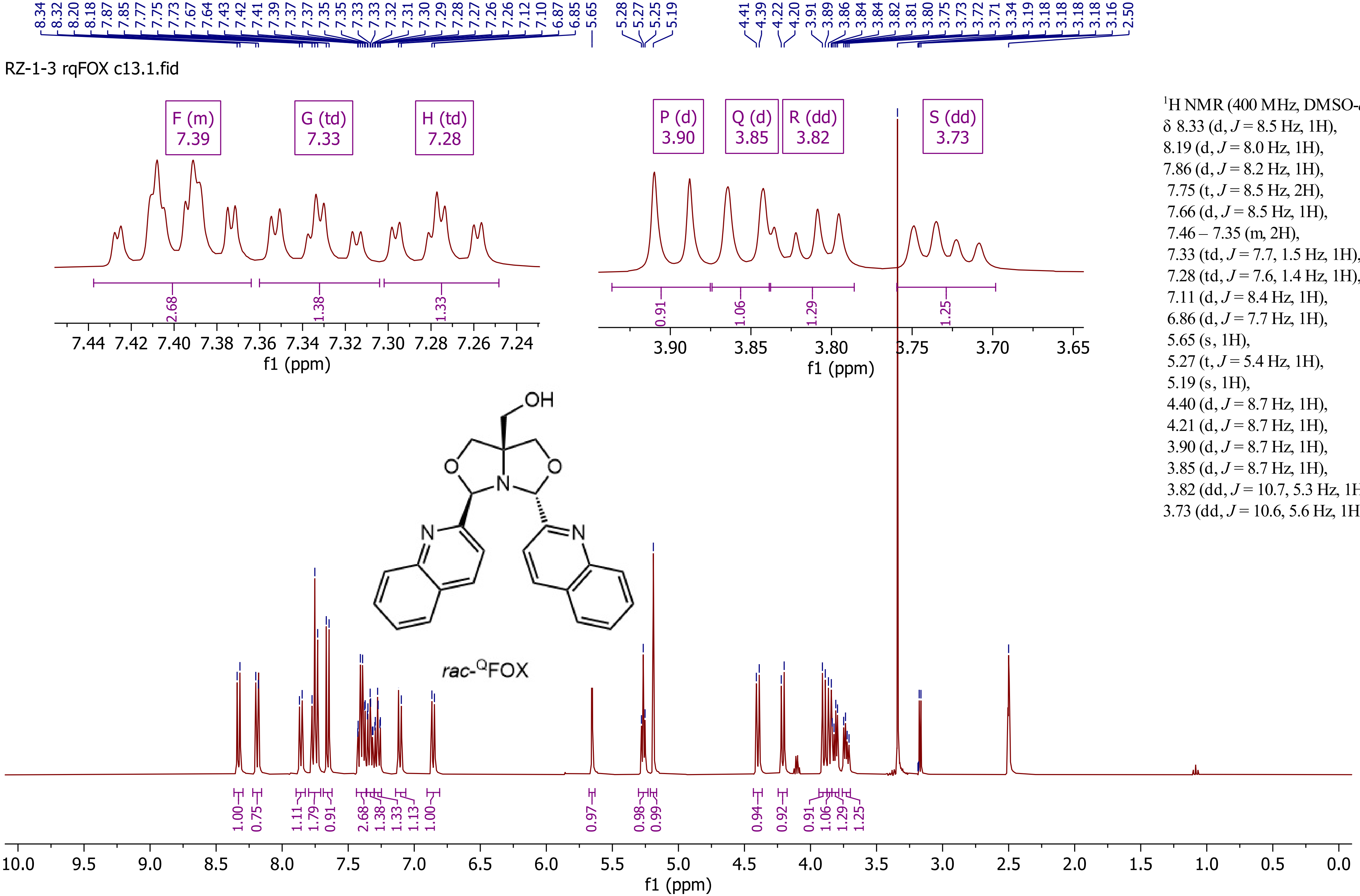

^{1}H NMR (400 MHz, DMSO-d_6:) δ 8.33 (d, J = 8.5 Hz, 1H), 8.19 (d, J = 8.0 Hz, 1H), 7.86 (d, J = 8.2 Hz, 1H), 7.75 (t, J = 8.5 Hz, 2H), 7.66 (d, J = 8.5 Hz, 1H), 7.46 – 7.35 (m, 2H), 7.33 (td, J = 7.7, 1.5 Hz, 1H), 7.28 (td, J = 7.6, 1.4 Hz, 1H), 7.11 (d, J = 8.4 Hz, 1H), 6.86 (d, J = 7.7 Hz, 1H), 5.65 (s, 1H), 5.27 (t, J = 5.4 Hz, 1H), 5.19 (s, 1H), 4.40 (d, J = 8.7 Hz, 1H), 4.21 (d, J = 8.7 Hz, 1H), 3.90 (d, J = 8.7 Hz, 1H), 3.85 (d, J = 8.7 Hz, 1H), 3.82 (dd, J = 10.7, 5.3 Hz, 1H), 3.73 (dd, J = 10.6, 5.6 Hz, 1H).

Figure S-2. $^{13}C\{^{1}H\}$ NMR spectrum of *rac*-^{Q}FOX in DMSO-d_6

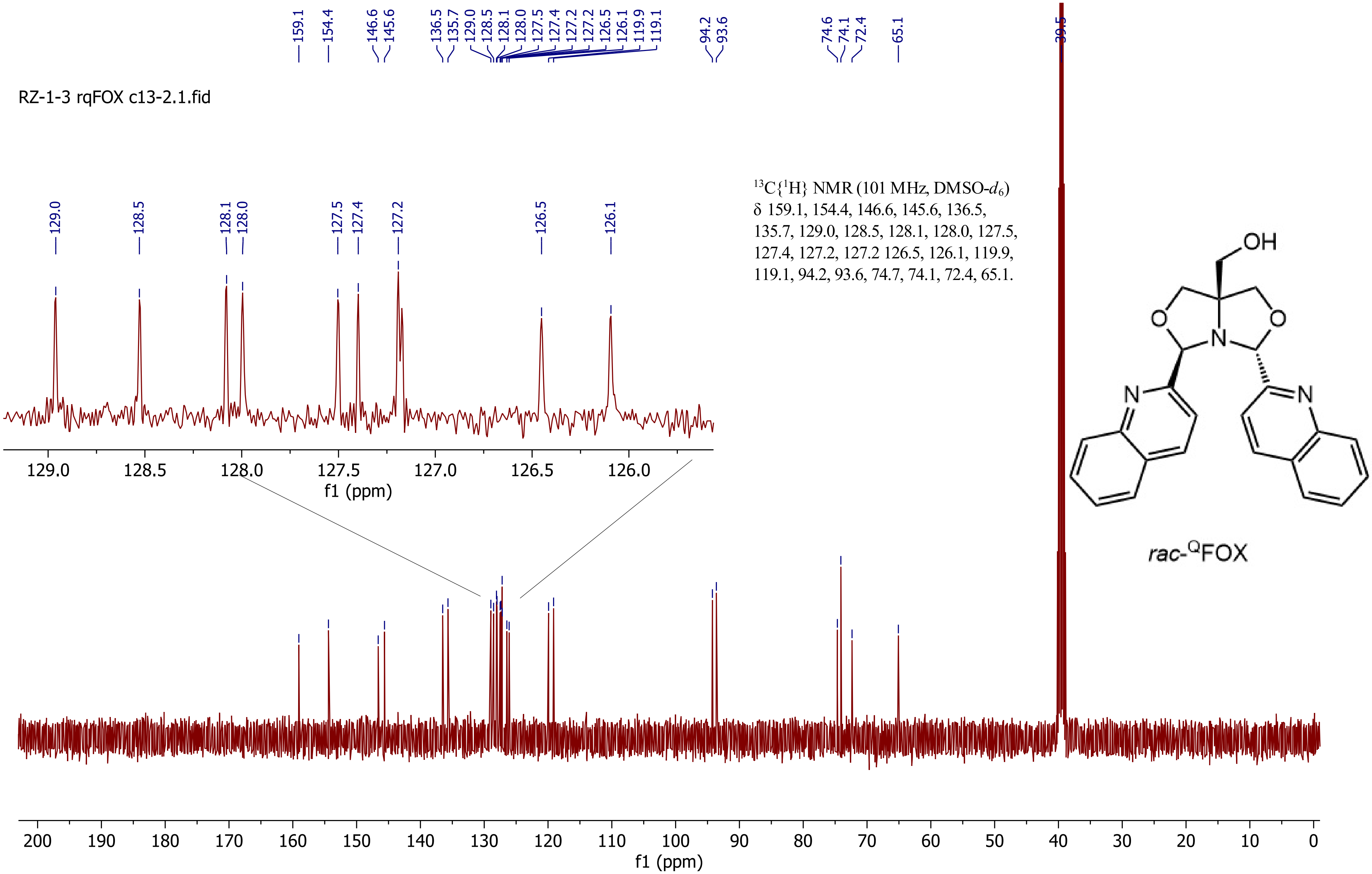

$^{13}C\{^{1}H\}$ NMR (101 MHz, DMSO-d_6) δ 159.1, 154.4, 146.6, 145.6, 136.5, 135.7, 129.0, 128.5, 128.1, 128.0, 127.5, 127.4, 127.2, 127.2 126.5, 126.1, 119.9, 119.1, 94.2, 93.6, 74.7, 74.1, 72.4, 65.1.

rac-^{Q}FOX

Figure S-3. ^{1}H NMR spectrum of *meso*-^{Q}FOX in DMSO-d_6

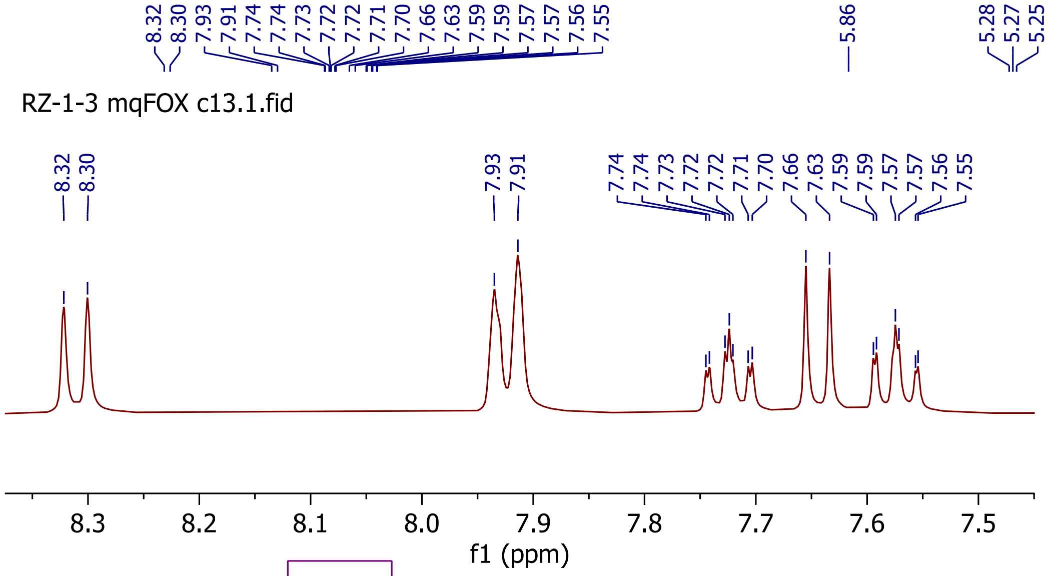

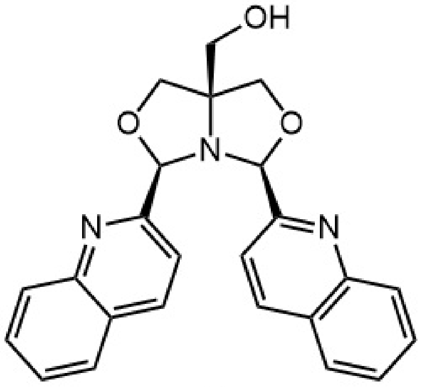

^{1}H NMR (400 MHz, DMSO-d_6): δ 8.31 (d, J = 8.5 Hz, 1H), 7.92 (d, J = 8.4 Hz, 2H), 7.72 (ddd, J = 8.3, 6.9, 1.4 Hz, 1H), 7.64 (d, J = 8.5 Hz, 1H), 7.57 (td, J = 7.4, 1.0 Hz, 1H), 5.86 (s, 1H), 5.27 (t, J = 5.4 Hz, 1H), 4.26 (d, J = 8.6 Hz, 1H), 3.97 (d, J = 8.7 Hz, 1H), 3.53 (d, J = 5.4 Hz, 1H).

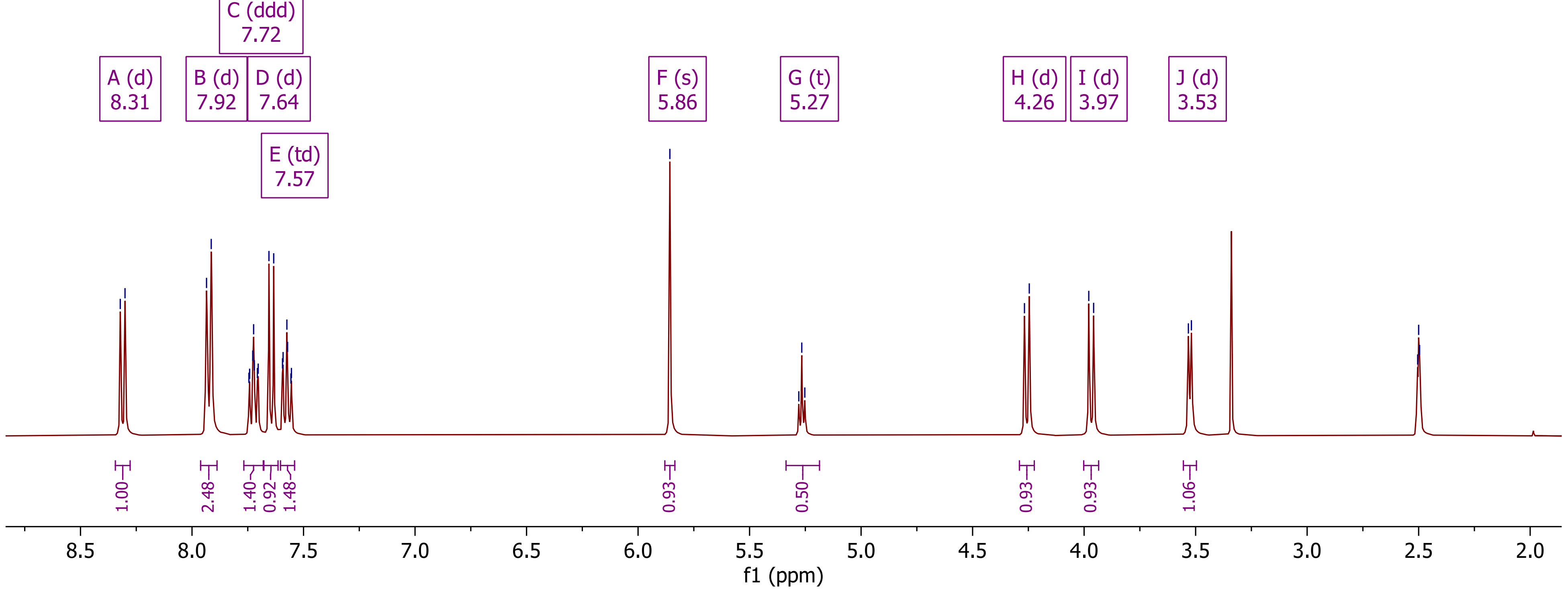

Figure S-4. $^{13}C\{^1H\}$ NMR spectrum of *meso*-^{Q}FOX in DMSO-d_6

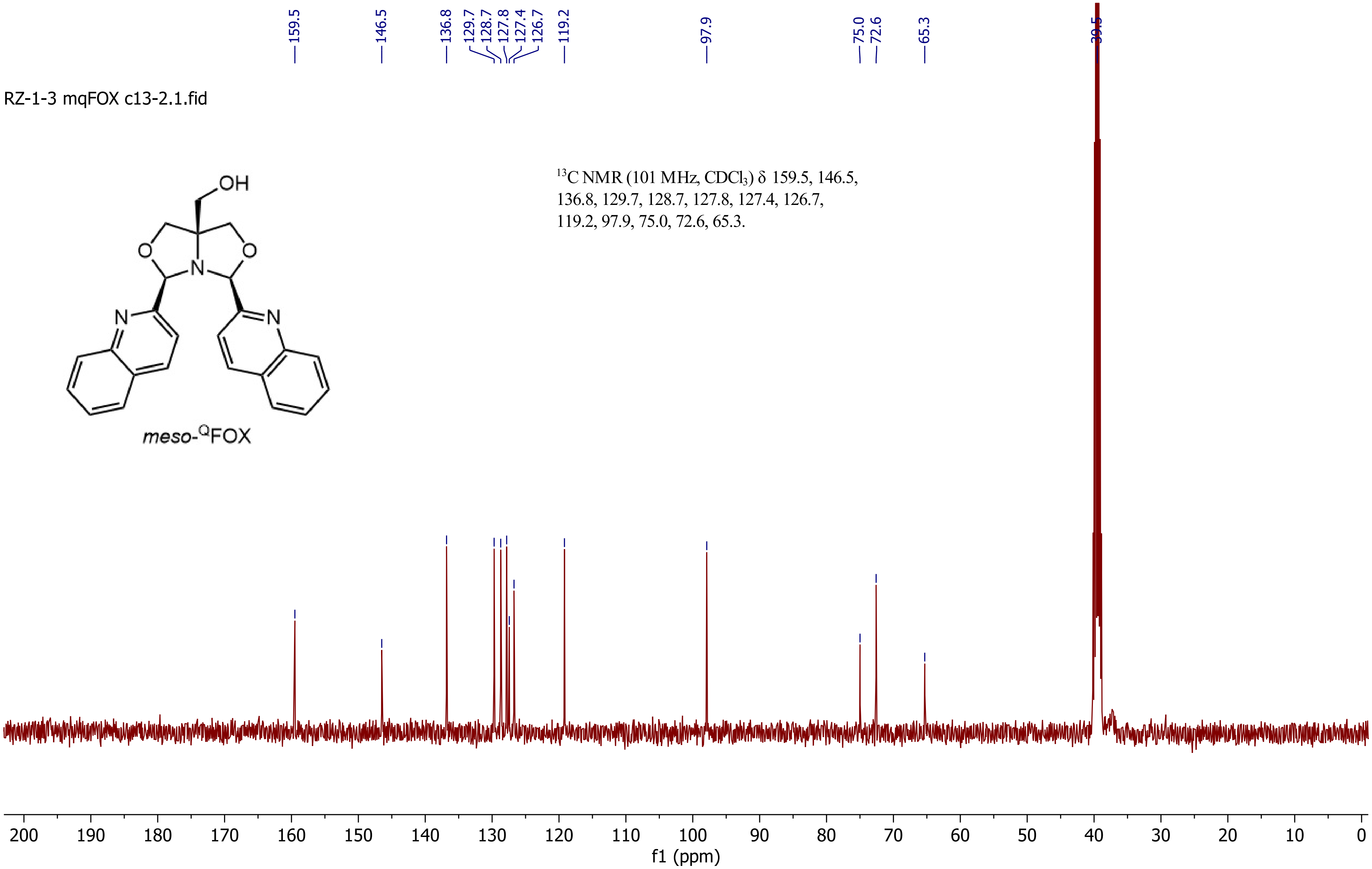

Figure S-5. ^{1}H NMR spectrum of α-methylbenzyl ethers

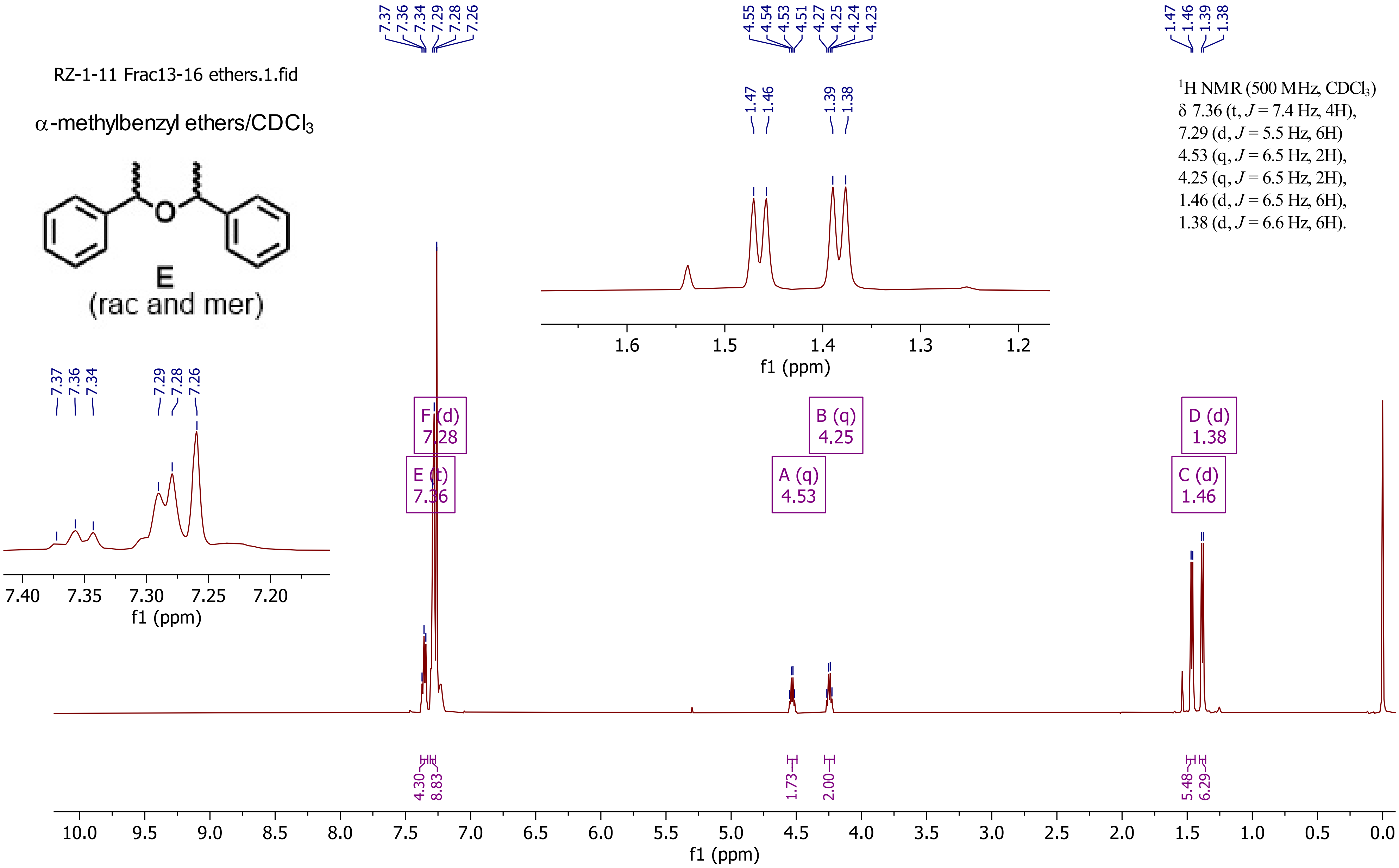

^{1}H NMR (500 MHz, $CDCl_3$) δ 7.36 (t, J = 7.4 Hz, 4H), 7.29 (d, J = 5.5 Hz, 6H) 4.53 (q, J = 6.5 Hz, 2H), 4.25 (q, J = 6.5 Hz, 2H), 1.46 (d, J = 6.5 Hz, 6H), 1.38 (d, J = 6.6 Hz, 6H).

Figure S-6. $^{19}F\{^1H\}$ spectrum of **2a** (Evan's method)

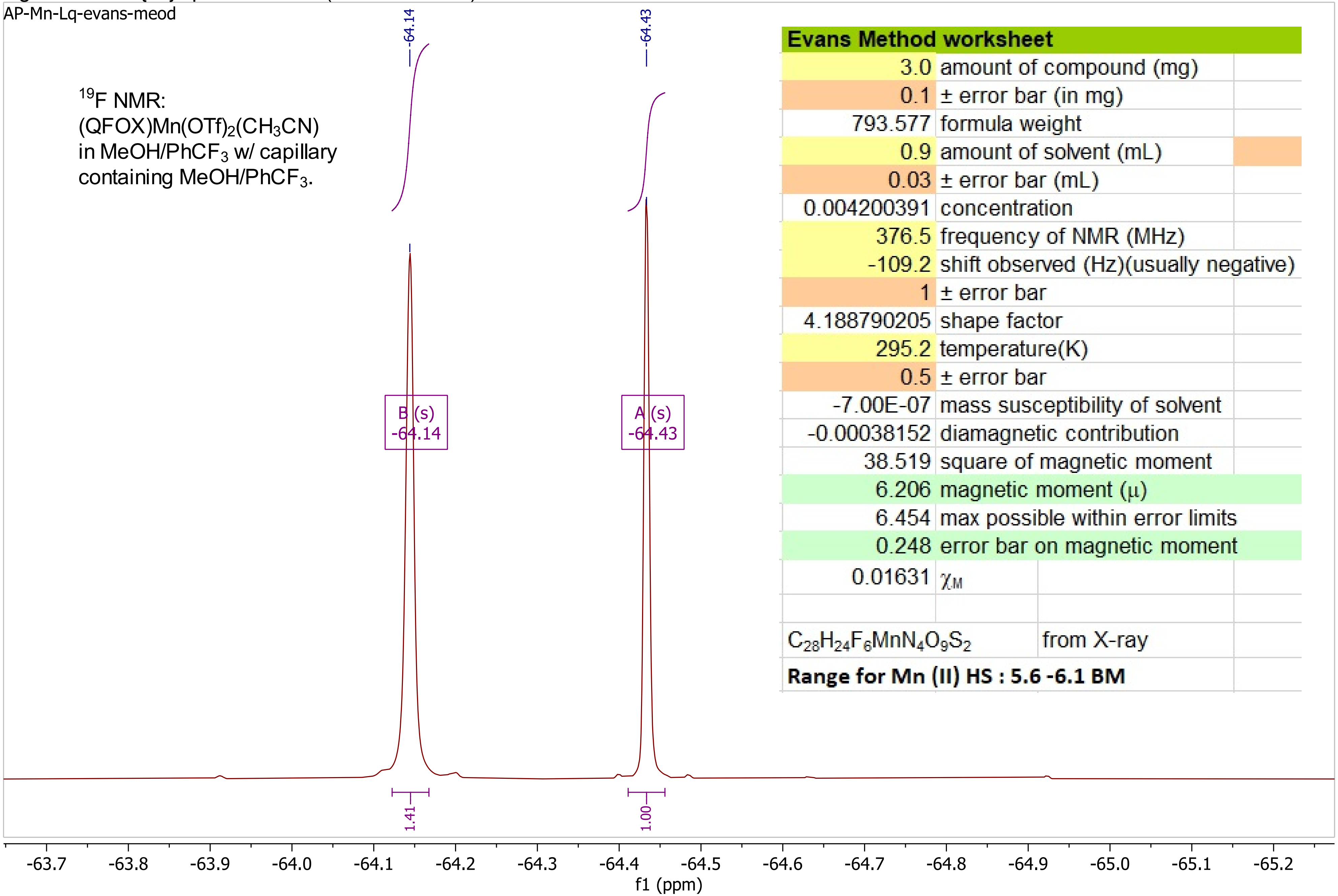

Evans Method worksheet		
3.0	amount of compound (mg)	
0.1	± error bar (in mg)	
793.577	formula weight	
0.9	amount of solvent (mL)	
0.03	± error bar (mL)	
0.004200391	concentration	
376.5	frequency of NMR (MHz)	
-109.2	shift observed (Hz)(usually negative)	
1	± error bar	
4.188790205	shape factor	
295.2	temperature(K)	
0.5	± error bar	
-7.00E-07	mass susceptibility of solvent	
-0.00038152	diamagnetic contribution	
38.519	square of magnetic moment	
6.206	magnetic moment (μ)	
6.454	max possible within error limits	
0.248	error bar on magnetic moment	
0.01631	χ_M	
$C_{28}H_{24}F_6MnN_4O_9S_2$	from X-ray	
**Range for Mn (II) HS : 5.6 -6.1 BM**		

Figure S-7. $^{19}F\{^1H\}$ spectrum of **2b** (Evan's method)

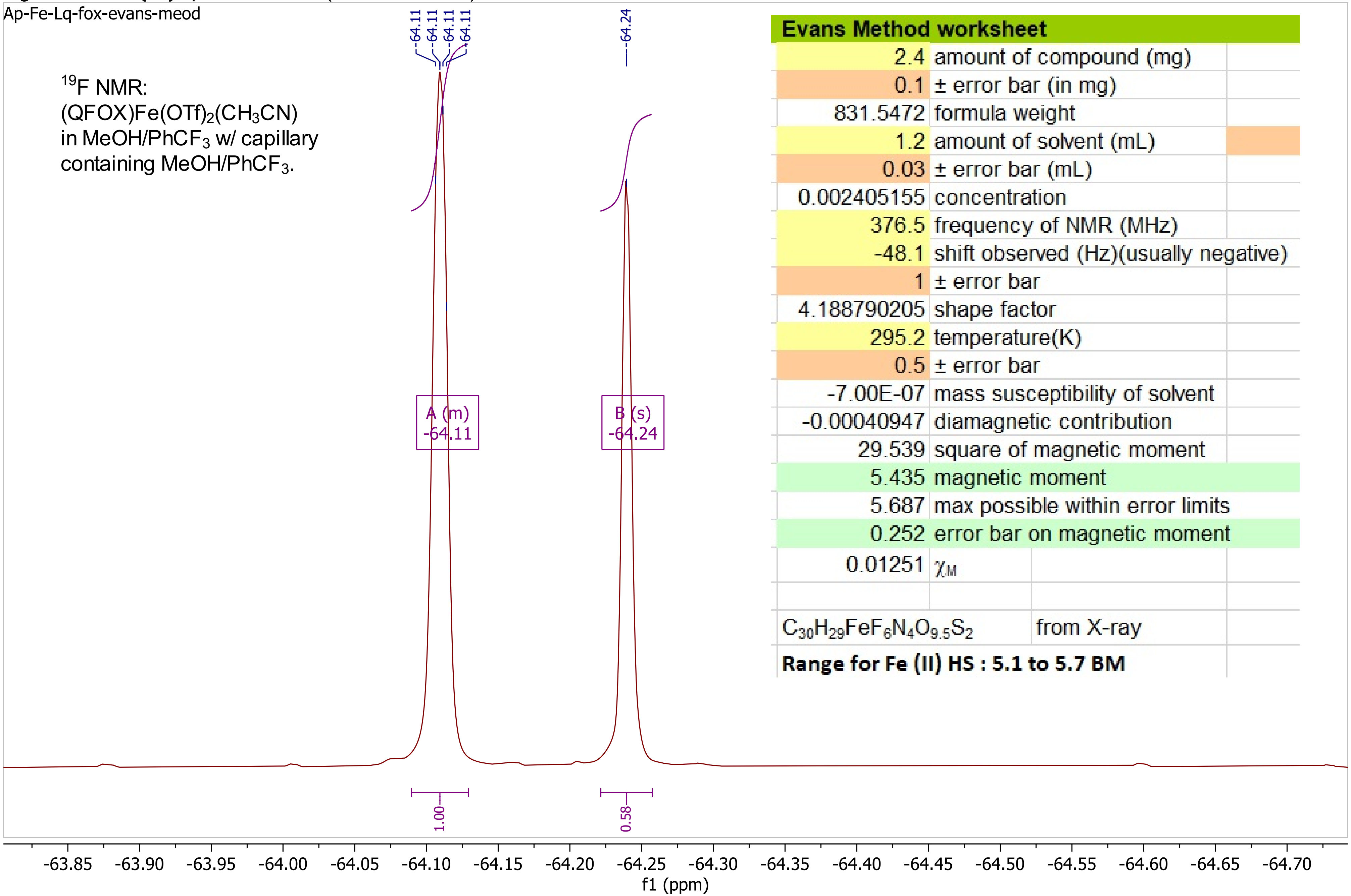

Evans Method worksheet		
2.4	amount of compound (mg)	
0.1	± error bar (in mg)	
831.5472	formula weight	
1.2	amount of solvent (mL)	
0.03	± error bar (mL)	
0.002405155	concentration	
376.5	frequency of NMR (MHz)	
-48.1	shift observed (Hz)(usually negative)	
1	± error bar	
4.188790205	shape factor	
295.2	temperature(K)	
0.5	± error bar	
-7.00E-07	mass susceptibility of solvent	
-0.00040947	diamagnetic contribution	
29.539	square of magnetic moment	
5.435	magnetic moment	
5.687	max possible within error limits	
0.252	error bar on magnetic moment	
0.01251	χ_M	
$C_{30}H_{29}FeF_6N_4O_{9.5}S_2$		from X-ray
**Range for Fe (II) HS : 5.1 to 5.7 BM**		

Figure S-8. $^{19}F\{^{1}H\}$ spectrum of **2c** (Evan's method)

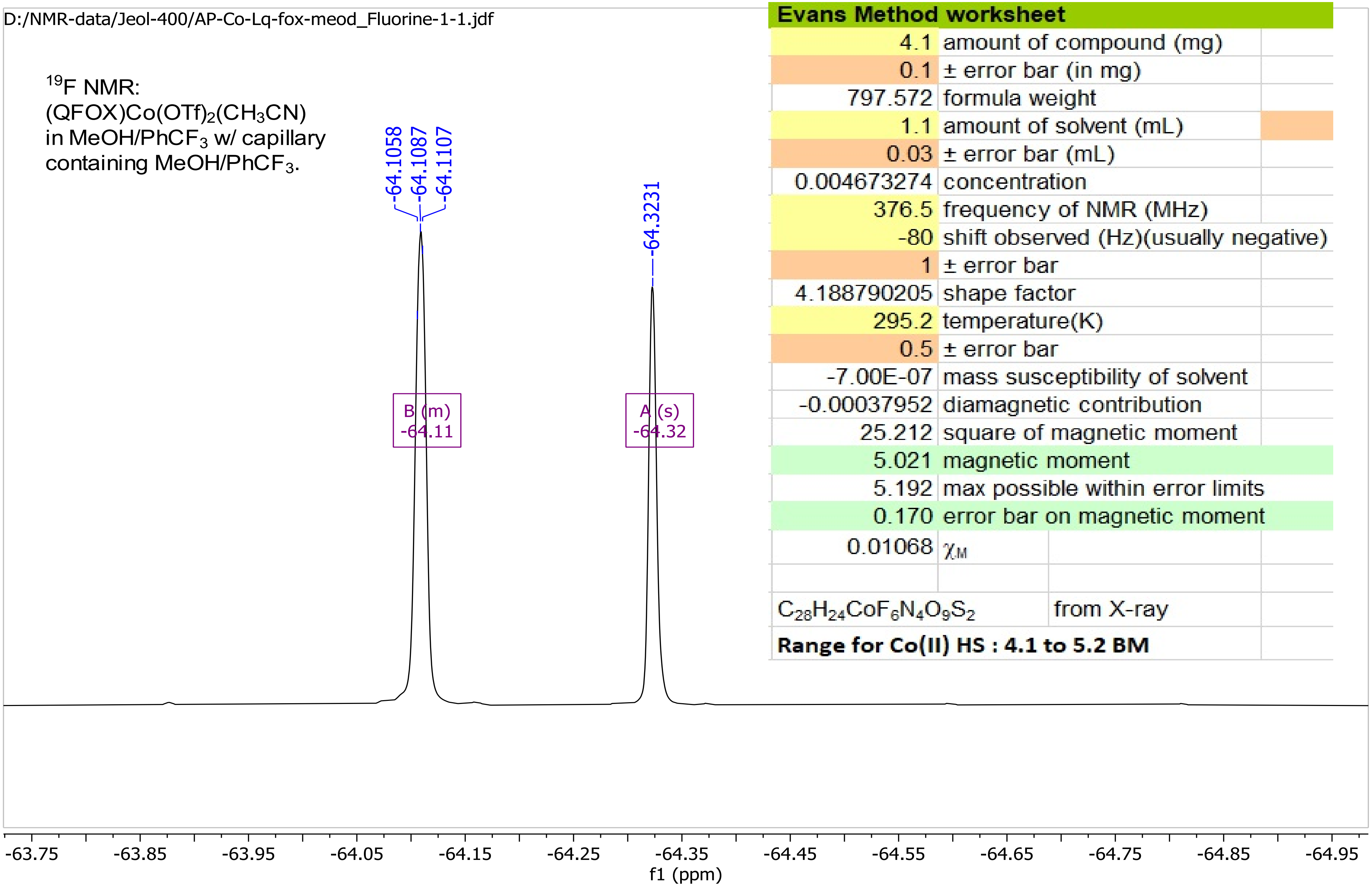

Evans Method worksheet		
4.1	amount of compound (mg)	
0.1	± error bar (in mg)	
797.572	formula weight	
1.1	amount of solvent (mL)	
0.03	± error bar (mL)	
0.004673274	concentration	
376.5	frequency of NMR (MHz)	
-80	shift observed (Hz)(usually negative)	
1	± error bar	
4.188790205	shape factor	
295.2	temperature(K)	
0.5	± error bar	
-7.00E-07	mass susceptibility of solvent	
-0.00037952	diamagnetic contribution	
25.212	square of magnetic moment	
5.021	magnetic moment	
5.192	max possible within error limits	
0.170	error bar on magnetic moment	
0.01068	χ_M	
$C_{28}H_{24}CoF_6N_4O_9S_2$		from X-ray
**Range for Co(II) HS : 4.1 to 5.2 BM**		

Figure S-9. $^{19}F\{^{1}H\}$ spectrum of **2d** (Evan's method)

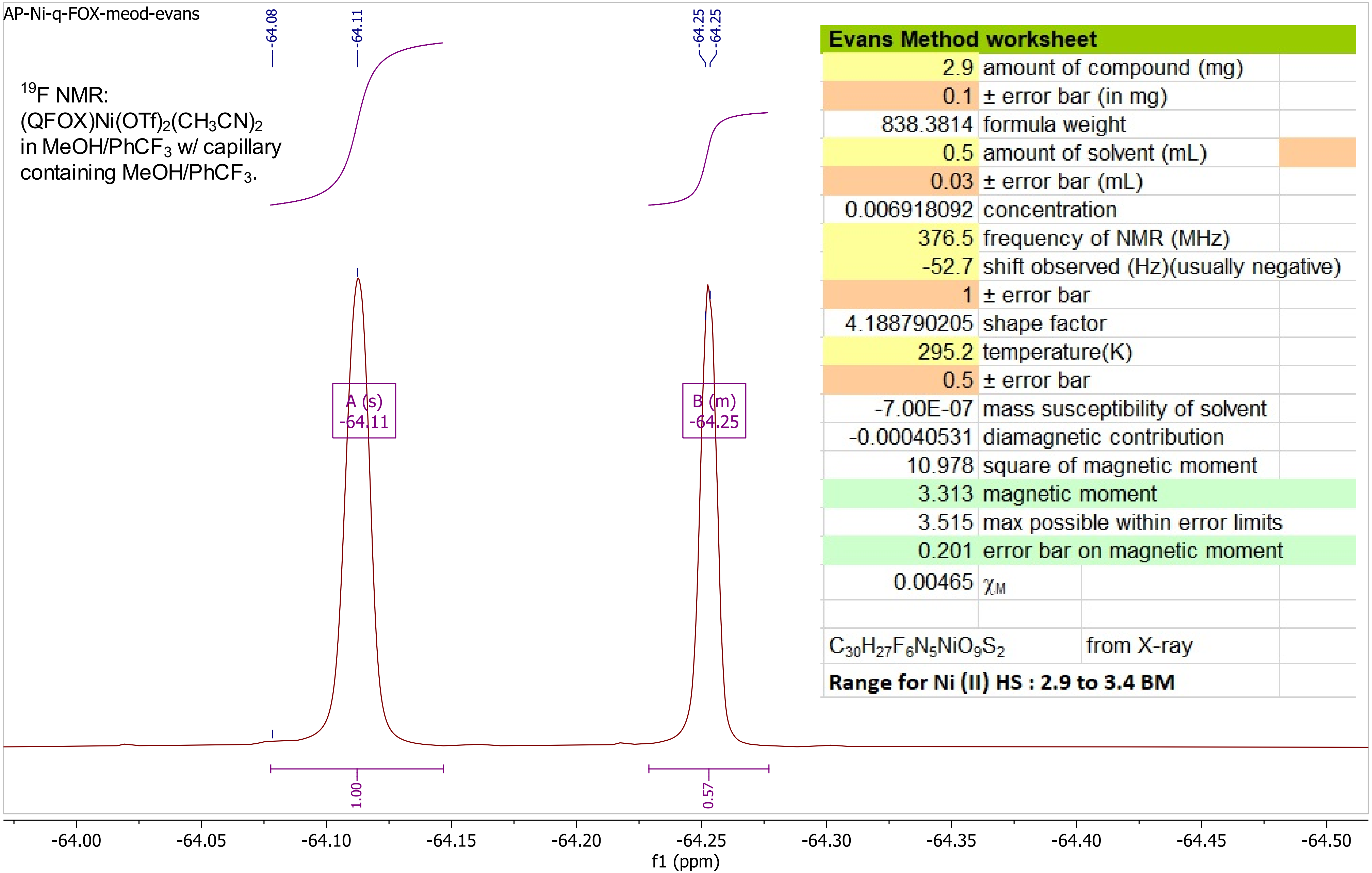

**Evans Method worksheet**		
2.9	amount of compound (mg)	
0.1	± error bar (in mg)	
838.3814	formula weight	
0.5	amount of solvent (mL)	
0.03	± error bar (mL)	
0.006918092	concentration	
376.5	frequency of NMR (MHz)	
-52.7	shift observed (Hz)(usually negative)	
1	± error bar	
4.188790205	shape factor	
295.2	temperature(K)	
0.5	± error bar	
-7.00E-07	mass susceptibility of solvent	
-0.00040531	diamagnetic contribution	
10.978	square of magnetic moment	
3.313	magnetic moment	
3.515	max possible within error limits	
0.201	error bar on magnetic moment	
0.00465	χ_M	
$C_{30}H_{27}F_6N_5NiO_9S_2$		from X-ray
**Range for Ni (II) HS : 2.9 to 3.4 BM**		

Figure S-10. $^{19}F\{^1H\}$ spectrum of **2e** (Evan's method)

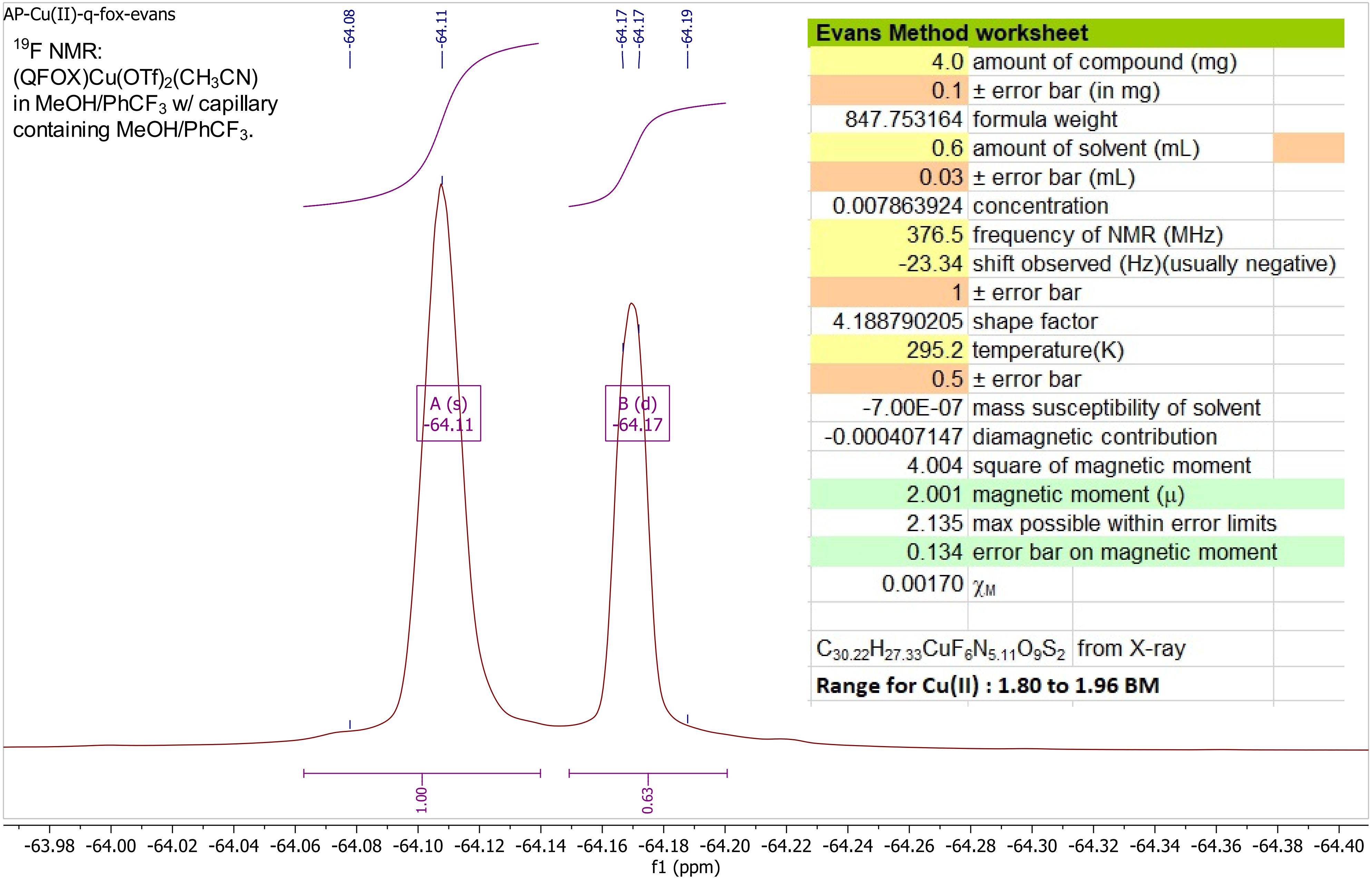

Evans Method worksheet		
4.0	amount of compound (mg)	
0.1	± error bar (in mg)	
847.753164	formula weight	
0.6	amount of solvent (mL)	
0.03	± error bar (mL)	
0.007863924	concentration	
376.5	frequency of NMR (MHz)	
-23.34	shift observed (Hz)(usually negative)	
1	± error bar	
4.188790205	shape factor	
295.2	temperature(K)	
0.5	± error bar	
-7.00E-07	mass susceptibility of solvent	
-0.000407147	diamagnetic contribution	
4.004	square of magnetic moment	
2.001	magnetic moment (μ)	
2.135	max possible within error limits	
0.134	error bar on magnetic moment	
0.00170	χ_M	
$C_{30.22}H_{27.33}CuF_6N_{5.11}O_9S_2$	from X-ray	
**Range for Cu(II) : 1.80 to 1.96 BM**		

Dehydration of 1-phenylethanol (0.59 M) at 120 °C in ODCB-d_4. (1% 2e)

By ^{1}H NMR	Areas	δ 4.75	6.55,6.50	4.43	4.15	6.28	WeightedAreas		(2H)	(2H)	(2H)		Percentages of total					
time, h	TMS	PhEtOH	Styrene	Ether1	Ether2	S-dimer	PhEtOH	Styrene	Ether1	Ether2	S-dimer	Sum	PhEtOH	Styrene	Ether1	Ether2	S-dimer	Sum
0	100	7.95	0	0	0	0	7.95	0	0	0	0	7.95	100	0	0	0	0	100
3	100	0.52	4.4	0.54	1.37	0.20	0.52	4.4	0.27	0.685	0.1	5.975	8.70	73.64	4.52	11.46	1.67	100
5	100	0.46	4.5	0.25	0.73	0.32	0.46	4.5	0.125	0.365	0.16	5.61	8.20	80.21	2.23	6.51	2.85	100
8	100	0.39	6.91	0.14	0.55	0.59	0.39	6.91	0.07	0.275	0.295	7.94	4.91	87.03	0.88	3.46	3.72	100
11	100	0.18	5.68	0.02	0.17	0.60	0.18	5.68	0.01	0.085	0.3	6.255	2.88	90.81	0.16	1.36	4.80	100
13	100	0.2	5.46	0.02	0.11	0.64	0.2	5.46	0.01	0.055	0.32	6.045	3.31	90.32	0.17	0.91	5.29	100

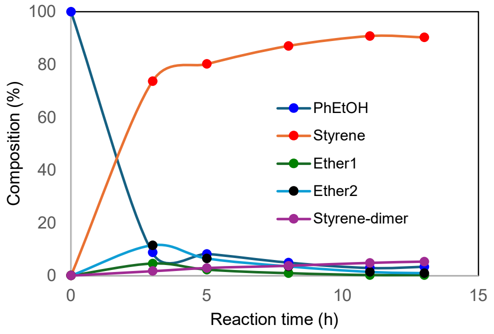

Dehydration of 1-phenylpropanol (0.55 M) at 120 °C in toluene-d_8 (1% 2e)

By ^{1}H NMR	Areas	1H@4.47	6.27,6.06	6.35,5.67	2H@4.28	2H@3.96	2H	WtdAreas			(2H)	(2H)	(2H)		Percentages of total						
time, h	TMS	PhPrOH	trans-ene	cis-ene	Ether1	Ether2	S-dimer	PhPrOH	trans-ene	cis-ene	Ether1	Ether2	S-dimer	Sum	PhEtOH	trans-ene	cis-ene	Ether1	Ether2	S-dimer	Sum
0	100	15.57	0	0	0	0	0	15.57	0	0	0	0	0	15.57	100	0	0	0	0	0	100
1	100	6.89	3.70	0.25	2.48	2.76	0	6.89	3.7	0.25	1.24	1.38	0	13.46	51.19	27.49	1.857355	9.21	10.25	0.00	100
2	100	5.89	4.64	0.33	2.68	3.14	0	5.89	4.64	0.33	1.34	1.57	0	13.77	42.77	33.70	2.396514	9.73	11.40	0.00	100
3	100	3.87	4.52	0.32	2.12	2.68	0	3.87	4.52	0.32	1.06	1.34	0	11.11	34.83	40.68	2.880288	9.54	12.06	0.00	100
4	100	3.46	5.20	0.40	2.11	2.80	0	3.46	5.2	0.4	1.055	1.4	0	11.515	30.05	45.16	3.47373	9.16	12.16	0.00	100
18	100	0	12.90	1.05	0	0.05	0	0	12.9	1.05	0	0.025	0	13.975	0.00	92.31	7.513417	0.00	0.18	0.00	100

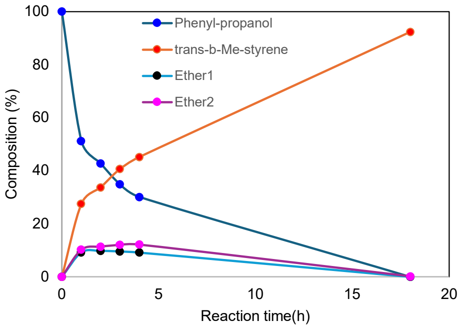

Figure S13. Dehydration of cycloheptanol (0.83 M), 1% Cu^{py}FOX, toluene, 120 °C, 48 h (Data for some dehydrations were reported previously in reference 12.)

SHIMADZU LabSolutions **Analysis Report**

<Sample Information>

Sample Name	: AP-04-132A-cyheptol-48h		
Sample ID	: AP-04-132A-cyheptol-48h		
Data Filename	: AP-04-132A-cyheptol-48h.gcd		
Method Filename	: Alcohols (FID ONLY)-45 min run.gcm		
Batch Filename	:		
Vial #	: 1	Sample Type	: Unknown
Injection Volume	: 1 uL		
Date Acquired	: 3/12/2025 9:10:44 AM	Acquired by	: wdjgroup
Date Processed	: 3/12/2025 9:42:16 AM	Processed by	: wdjgroup

<Chromatogram>

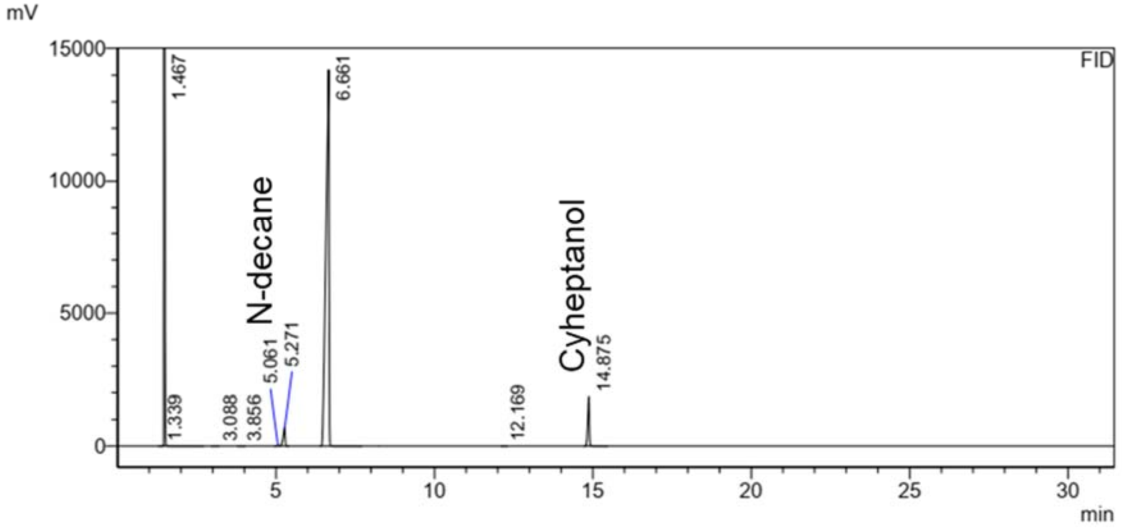

<Peak Table>

FID

Peak#	Ret. Time	Area	Height	Conc.	Unit	Mark	Name
1	1.339	16685	12643	0.000			
2	1.467	96563297	56054353	0.000		SV	
3	3.088	22471	6024	0.000			
4	3.856	10383	2840	0.000			
5	5.061	114810	28919	0.000			
6	5.271	3053432	676957	0.000	ppm	V	n-decane
7	6.661	95107994	14162795	0.000		S	
8	12.169	20493	7252	0.000			
9	14.875	6566384	1846135	-0.000	ppm		cyheptanol
Total		201475949	72797919				

Figure S-14 Dehydration of cyclooctanol (0.83 M), 1% $Cu^{py}FOX$, toluene, 120 °C, 48 h

SHIMADZU LabSolutions

Analysis Report

<Sample Information>

Sample Name : AP-04-132B-cyheptol-48h
Sample ID : AP-04-132B-cyheptol-48h
Data Filename : AP-04-132B-cyheptol-48h.gcd
Method Filename : Alcohols (FID ONLY)-45 min run.gcm
Batch Filename :
Vial # : 1   Sample Type : Unknown
Injection Volume : 1 uL
Date Acquired : 3/12/2025 9:47:16 AM   Acquired by : wdjgroup
Date Processed : 3/12/2025 10:14:26 AM   Processed by : wdjgroup

<Chromatogram>

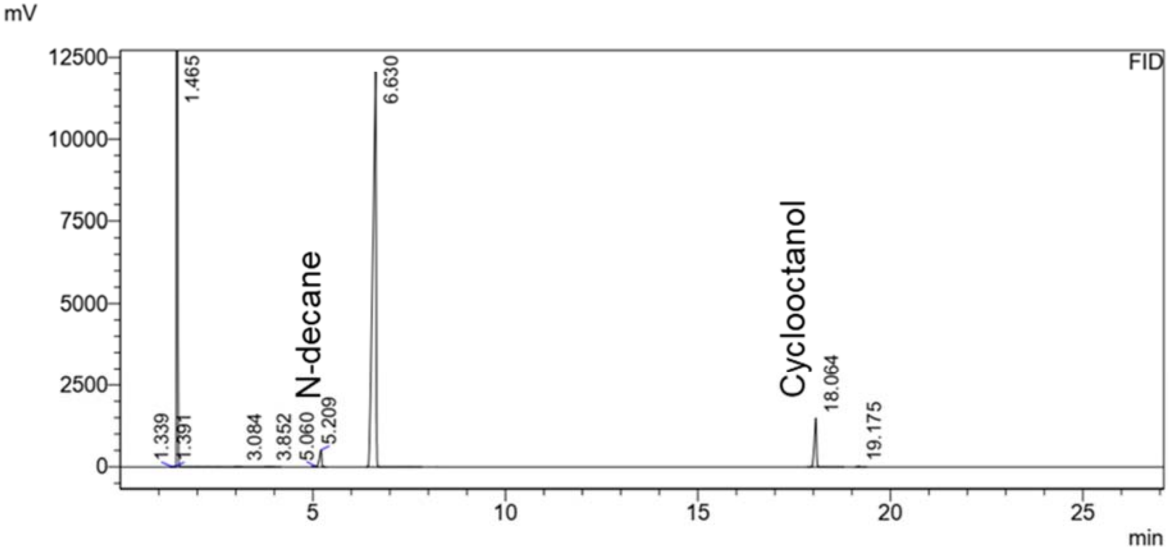

<Peak Table>

FID

Peak#	Ret. Time	Area	Height	Conc.	Unit	Mark	Name
1	1.339	18364	14000	0.000		V	
2	1.391	10551	7463	0.000		V	
3	1.465	110031525	63793952	0.000		SV	
4	3.084	15218	3073	0.000		TV	
5	3.852	12255	3386	0.000		T	
6	5.060	114239	30791	0.000			
7	5.209	2108711	514044	0.000	ppm	V	n-decane
8	6.630	71098053	12004661	0.000		SV	
9	18.064	5749490	1484964	-0.000	ppm	S	cy-octanol
10	19.175	73631	22128	0.000			
Total		189232037	77878462				

Figure S-15. Dehydration of cyclooctanol (0.83 M), 1% Cu^{Q}FOX, ODCB, 120 °C, 48 h

SHIMADZU LabSolutions **Analysis Report**

<Sample Information>

Sample Name	: AP-04-131B-cyoctol-24h		
Sample ID	: AP-04-131B-cyoctol-24h		
Data Filename	: AP-04-131B-cyoctol-24h.gcd		
Method Filename	: Alcohols (FID ONLY)-45 min run.gcm		
Batch Filename	:		
Vial #	: 2	Sample Type	: Unknown
Injection Volume	: 1 uL		
Date Acquired	: 3/4/2025 11:37:08 AM	Acquired by	: wdjgroup
Date Processed	: 3/4/2025 12:23:32 PM	Processed by	: wdjgroup

<Chromatogram>

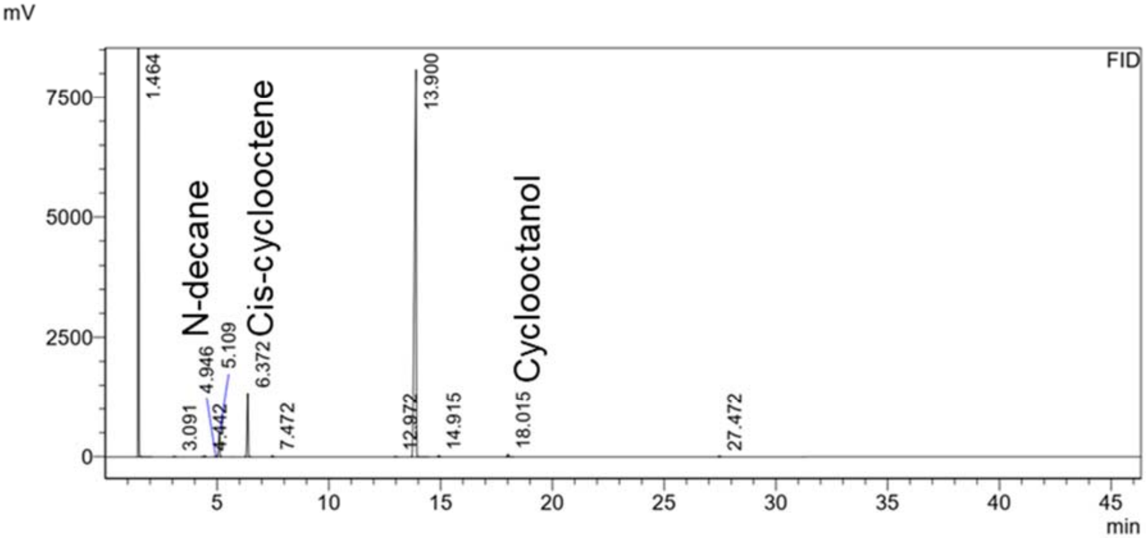

<Peak Table>

FID

Peak#	Ret. Time	Area	Height	Conc.	Unit	Mark	Name
1	1.464	117881154	66945864	0.000		SV	
2	3.091	30703	11908	0.000			
3	4.442	104806	25820	0.000		V	
4	4.946	33078	8691	0.000			
5	5.109	1894351	508394	0.000	ppm	V	n-decane
6	6.372	4487328	1333289	-0.000	ppm	S	cis-cyoctene
7	7.472	73639	29581	0.000		V	
8	12.972	22979	9832	0.000		S	
9	13.900	45490075	8073422	0.000		SV	
10	14.915	85323	30257	0.000		V	
11	18.015	190825	55690	-0.000	ppm	SV	cy-octanol
12	27.472	66195	19703	0.000		V	
Total		170360457	77052451				

Figure S-16. Dehydration of cycloheptanol (0.83 M), 1% Cu^{Q}FOX, ODCB, 120 °C, 48 h

SHIMADZU LabSolutions **Analysis Report**

<Sample Information>

Sample Name	: AP-04-131A-cyheptol-24h		
Sample ID	: AP-04-131A-cyheptol-24h		
Data Filename	: AP-04-131A-cyheptol-24h.gcd		
Method Filename	: Alcohols (FID ONLY)-45 min run.gcm		
Batch Filename	:		
Vial #	: 1	Sample Type	: Unknown
Injection Volume	: 1 uL		
Date Acquired	: 3/4/2025 10:43:26 AM	Acquired by	: wdjgroup
Date Processed	: 3/4/2025 11:29:49 AM	Processed by	: wdjgroup

<Chromatogram>

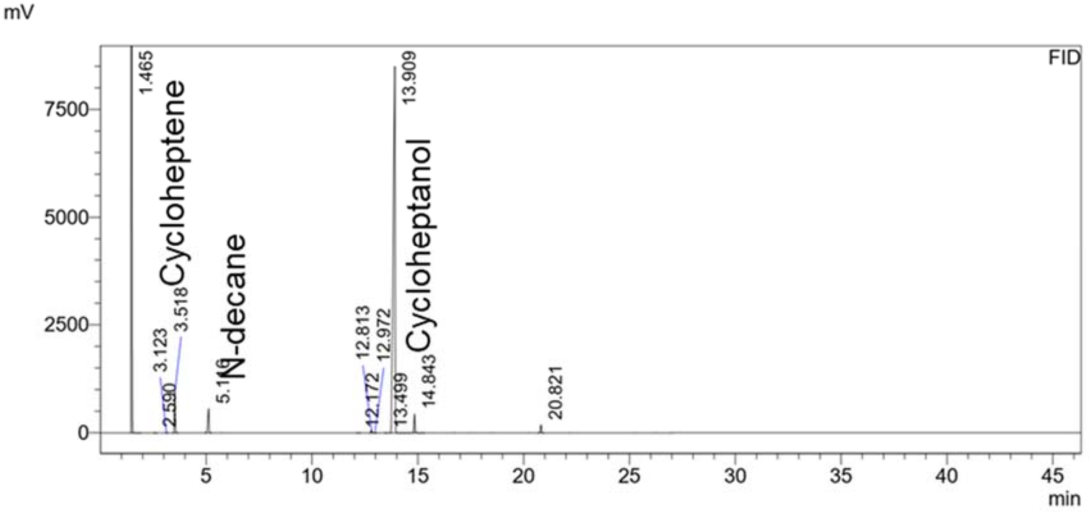

<Peak Table>

FID

Peak#	Ret. Time	Area	Height	Conc.	Unit	Mark	Name
1	1.465	114075207	64674911	0.000		V	
2	2.590	20099	8678	0.000			
3	3.123	14858	4903	0.000		V	
4	3.518	2646558	940804	-0.000	ppm		Cy-heptene
5	5.116	2112750	559725	0.000	ppm		n-decane
6	12.172	12601	4490	0.000			
7	12.813	191277	63909	0.000			
8	12.972	25757	11174	0.000		V	
9	13.499	12444	3576	0.000		V	
10	13.909	50869280	8487331	0.000		SV	
11	14.843	1396192	436236	-0.000	ppm	S	cy-heptanol
12	20.821	618954	187162	0.000			
Total		171995977	75382898				

Figure S-17. Dehydration of 1-Me-1-cyclohexanol (0.83 M), 1% Cu^{Q}FOX, ODCB, 120 °C, 24 h

SHIMADZU LabSolutions **Analysis Report**

<Sample Information>

Sample Name	: AP-04-097-B-24h		
Sample ID	: AP-04-097-B-24h		
Data Filename	: AP-04-097-B-24h.gcd		
Method Filename	: Alcohols (FID ONLY)-35 min run.gcm		
Batch Filename	:		
Vial #	: 1	Sample Type	: Unknown
Injection Volume	: 1 uL		
Date Acquired	: 10/4/2024 10:42:34 AM	Acquired by	: wdjgroup
Date Processed	: 10/4/2024 11:21:12 AM	Processed by	: wdjgroup

<Chromatogram>

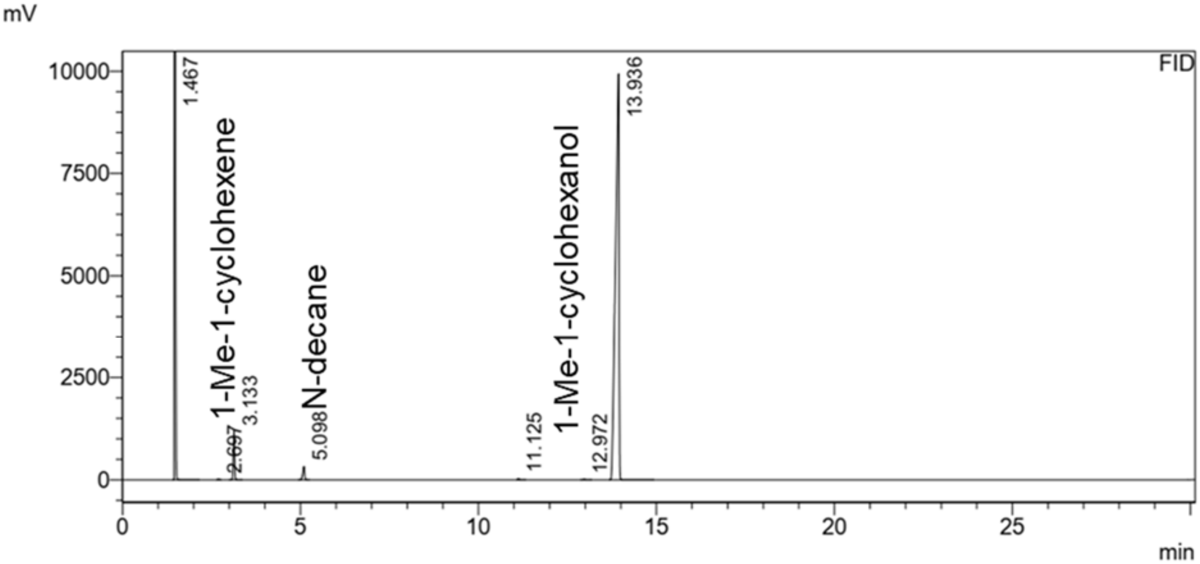

<Peak Table>

FID

Peak#	Ret. Time	Area	Height	Conc.	Unit	Mark	Name
1	1.467	82972599	49698714	0.000		SV	
2	2.697	31331	13378	-0.000	ppm	V	methylenecyhexane
3	3.133	2936832	1186756	-0.000	ppm		1-Me-1-cyhexene
4	5.098	1153176	326549	0.000	ppm		ndecane
5	11.125	63215	32725	0.000		V	
6	12.972	39912	15950	-0.000	ppm		Cyhexanol
7	13.936	66832596	9910914	0.000		S	
Total		154029661	61184987				

Figure S-18. Dehydration of cyclohex-2-en-1-ol (0.83 M), 1% Cu^{Q}FOX, ODCB, 120 °C, 24 h

SHIMADZU LabSolutions

Analysis Report

<Sample Information>

Sample Name	: AP-04-116A-cyclohex-2-en-1-ol		
Sample ID	: AP-04-116A-cyclohex-2-en-1-ol		
Data Filename	: AP-04-116A-cyclohex-2-en-1-ol.gcd		
Method Filename	: Alcohols (FID ONLY)-45 min run.gcm		
Batch Filename	:		
Vial #	: 1	Sample Type	: Unknown
Injection Volume	: 1 uL		
Date Acquired	: 12/3/2024 10:31:31 AM	Acquired by	: wdjgroup
Date Processed	: 12/3/2024 11:50:20 AM	Processed by	: wdjgroup

<Chromatogram>

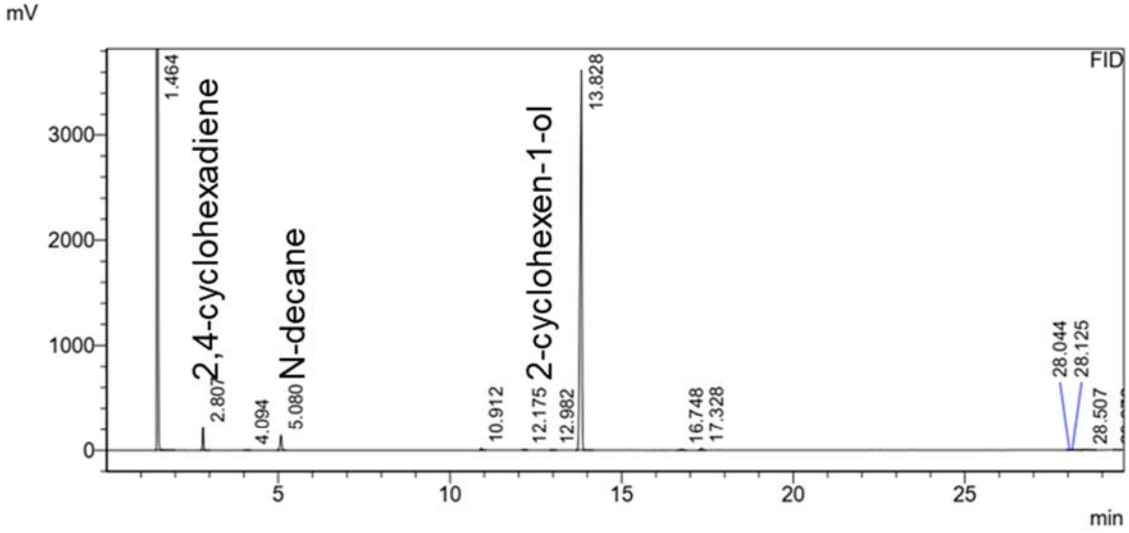

<Peak Table>

FID

Peak#	Ret. Time	Area	Height	Conc.	Unit	Mark	Name
1	1.464	122872705	69837115	0.000		V	
2	2.807	474138	216706	-0.000	ppm		2,4-cyclohexadiene
3	4.094	12645	4361	0.000		V	
4	5.080	475253	143304	0.000	ppm		n-decane
5	10.912	33673	17004	0.000			
6	12.175	25315	8387	0.000			
7	12.982	13613	4456	-0.000	ppm		2-cyclohexene-1-ol
8	13.828	13048675	3579616	0.000			
9	16.748	32270	10144	0.000			
10	17.328	55581	18743	0.000			
11	28.044	10310	2045	0.000		V	
12	28.125	24997	3522	0.000		V	
13	28.507	56622	5581	0.000		V	
14	29.372	26573	4335	0.000			
Total		137162371	73855319				

Figure S-19. Dehydration of cyclohexanol (0.83 M), 1% $Cu^{Q}FOX$, ODCB, 120 °C, 48 h

SHIMADZU LabSolutions **Analysis Report**

<Sample Information>

Sample Name	: AP-04-137A-cyhexol-48h		
Sample ID	: AP-04-137A-cyhexol-48h		
Data Filename	: AP-04-137A-cyhexol-48h.gcd		
Method Filename	: Alcohols (FID ONLY)-45 min run.gcm		
Batch Filename	:		
Vial #	: 1	Sample Type	: Unknown
Injection Volume	: 1 uL		
Date Acquired	: 4/10/2025 1:08:36 PM	Acquired by	: wdjgroup
Date Processed	: 4/11/2025 2:58:30 PM	Processed by	: wdjgroup

<Chromatogram>

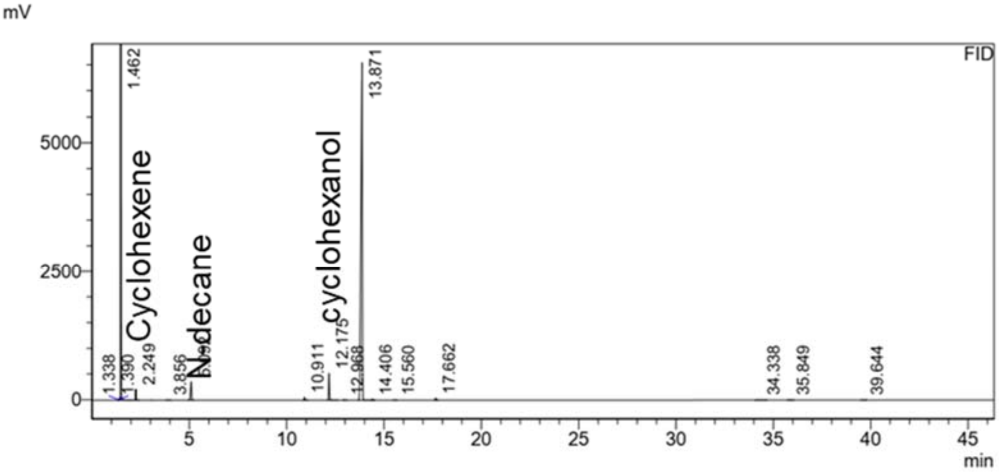

<Peak Table>

FID

Peak#	Ret. Time	Area	Height	Conc.	Unit	Mark	Name
1	1.338	21291	15767	0.000			
2	1.390	15749	11276	0.000		V	
3	1.462	127783705	71686794	0.000		SV	
4	2.249	399156	208346	-0.000	ppm	S	cyhexene
5	3.856	12674	3398	0.000			
6	5.092	1252447	357665	0.000	ppm		n-decane
7	10.911	111739	56562	0.000		V	
8	12.175	1364659	519488	-0.000	ppm	S	cyhexanol
9	12.968	17182	7449	0.000			
10	13.871	32214015	6532728	0.000		S	
11	14.406	39629	14674	0.000		T	
12	15.560	10257	3017	0.000			
13	17.662	110192	37845	0.000			
14	34.338	14202	821	0.000		V	
15	35.849	11111	2005	0.000		S	
16	39.644	10139	1634	0.000			
Total		163388147	79459465				

Figure S-20. Dehydration of cyclopentanol (0.83 M), 1% Cu^{Q}FOX, ODCB, 120 °C, 48 h

SHIMADZU LabSolutions **Analysis Report**

<Sample Information>

Sample Name	: AP-04-140B-48h-cypentol-q-fox		
Sample ID	: AP-04-140B-48h-cypentol-q-fox		
Data Filename	: AP-04-140B-48h-cypentol-q-fox.gcd		
Method Filename	: Alcohols (FID ONLY)-45 min run.gcm		
Batch Filename	:		
Vial #	: 1	Sample Type	: Unknown
Injection Volume	: 1 uL		
Date Acquired	: 4/14/2025 10:28:52 AM	Acquired by	: wdjgroup
Date Processed	: 10/17/2025 11:55:14 AM	Processed by	: wdjgroup

<Chromatogram>

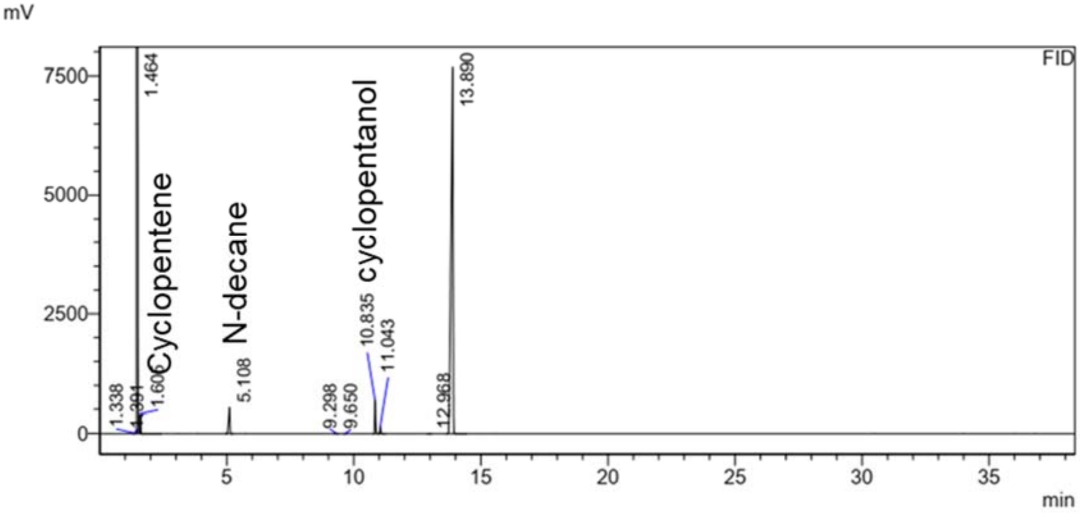

<Peak Table>

FID

Peak#	Ret. Time	Area	Height	Conc.	Unit	Mark	Name
1	1.338	20458	16335	0.000			
2	1.391	22403	13847	0.000		V	
3	1.464	121085592	68072525	0.000		SV	
4	1.605	574552	399772	-0.000	ppm	T	cypentene
5	5.108	2024816	538875	0.000	ppm		n-decane
6	9.298	27776	13827	0.000			
7	9.650	10508	5471	0.000			
8	10.835	1656526	675564	-0.000	ppm		cypentanol
9	11.043	371167	157638	0.000		V	
10	12.968	21724	9433	0.000			
11	13.890	41693499	7658825	0.000		S	
Total		167509022	77562113				

Figure S-21. Dehydration of cyclohexanol (0.83 M), 1% Cu^{py}FOX, toluene, 120 °C, 48 h

SHIMADZU LabSolutions

Analysis Report

<Sample Information>

Sample Name	: AP-04-141A-48h-cyhexol-py-fox		
Sample ID	: AP-04-141A-48h-cyhexol-py-fox		
Data Filename	: AP-04-141A-48h-cyhexol-py-fox.gcd		
Method Filename	: Alcohols (FID ONLY)-45 min run.gcm		
Batch Filename	:		
Vial #	: 1	Sample Type	: Unknown
Injection Volume	: 1 uL		
Date Acquired	: 4/14/2025 11:21:56 AM	Acquired by	: wdjgroup
Date Processed	: 4/14/2025 11:52:41 AM	Processed by	: wdjgroup

<Chromatogram>

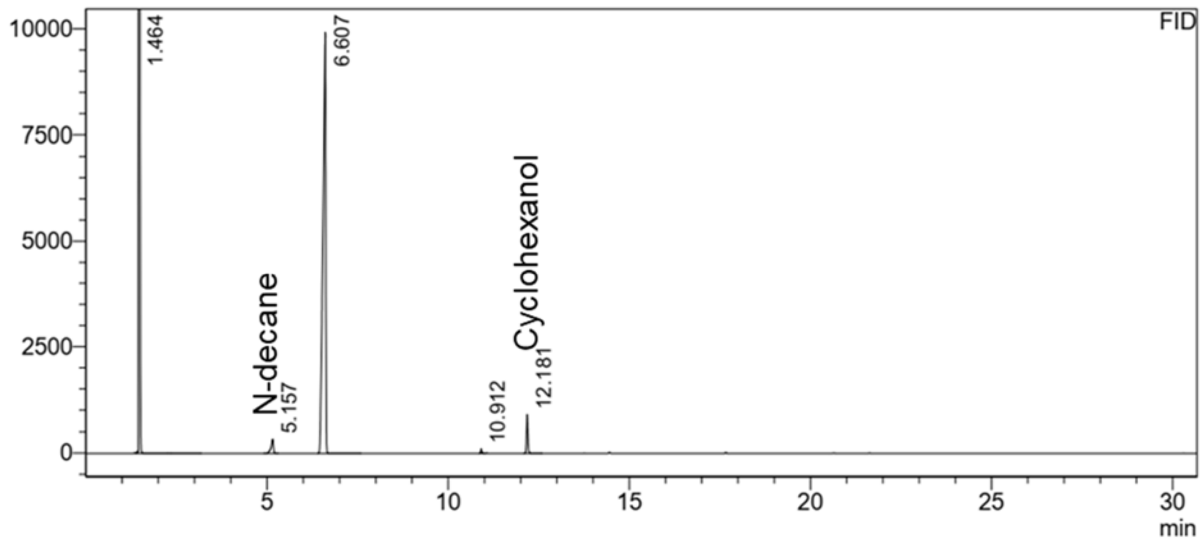

<Peak Table>

FID

Peak#	Ret. Time	Area	Height	Conc.	Unit	Mark	Name
1	1.464	119377702	67429607	0.000		SV	
2	5.157	1496221	335953	0.000	ppm		n-decane
3	6.607	51664330	9883612	0.000		S	
4	10.912	195830	99514	0.000			
5	12.181	2288720	892203	-0.000	ppm		Cy-hexanol
Total		175022803	78640889				

Figure S-22. Dehydration of cyclopentanol (0.83 M), 1% Cu^{py}FOX, toluene, 120 °C, 48 h

SHIMADZU LabSolutions **Analysis Report**

<Sample Information>

Sample Name	: AP-04-156B-cypentol-pyfox-48h		
Sample ID	: AP-04-156B-cypentol-pyfox-48h		
Data Filename	: AP-04-156B-cypentol-pyfox-48h001.gcd		
Method Filename	: Alcohols (FID ONLY)-33min-high-steep-rise run.gcm		
Batch Filename	:		
Vial #	: 1	Sample Type	: Unknown
Injection Volume	: 1 uL		
Date Acquired	: 10/17/2025 4:53:32 PM	Acquired by	: chemadmin
Date Processed	: 10/17/2025 5:51:56 PM	Processed by	: chemadmin

<Chromatogram>

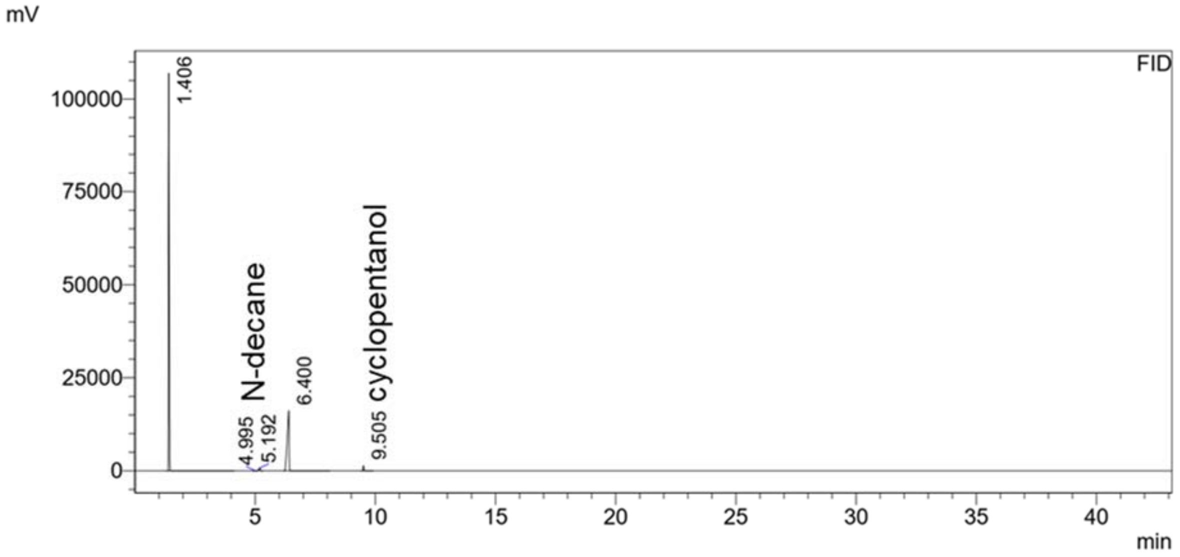

<Peak Table>

FID

Peak#	Ret. Time	Area	Height	Conc.	Unit	Mark	Name
1	1.406	212147007	105090397	0.000		SV	
2	4.995	102641	25024	0.000			
3	5.192	3221688	734141	0.000	ppm	V	n-decane
4	6.400	85660352	16135046	0.000		S	
5	9.505	3353645	1466333	-0.000	ppm		cy-pentanol
Total		304485334	123450941				

Figure S-23. ^{1}H-NMR Dehydration of tert-butanol (0.83 M), 1% Cu^{py}FOX, toluene-d_8, 120 °C, 0 h

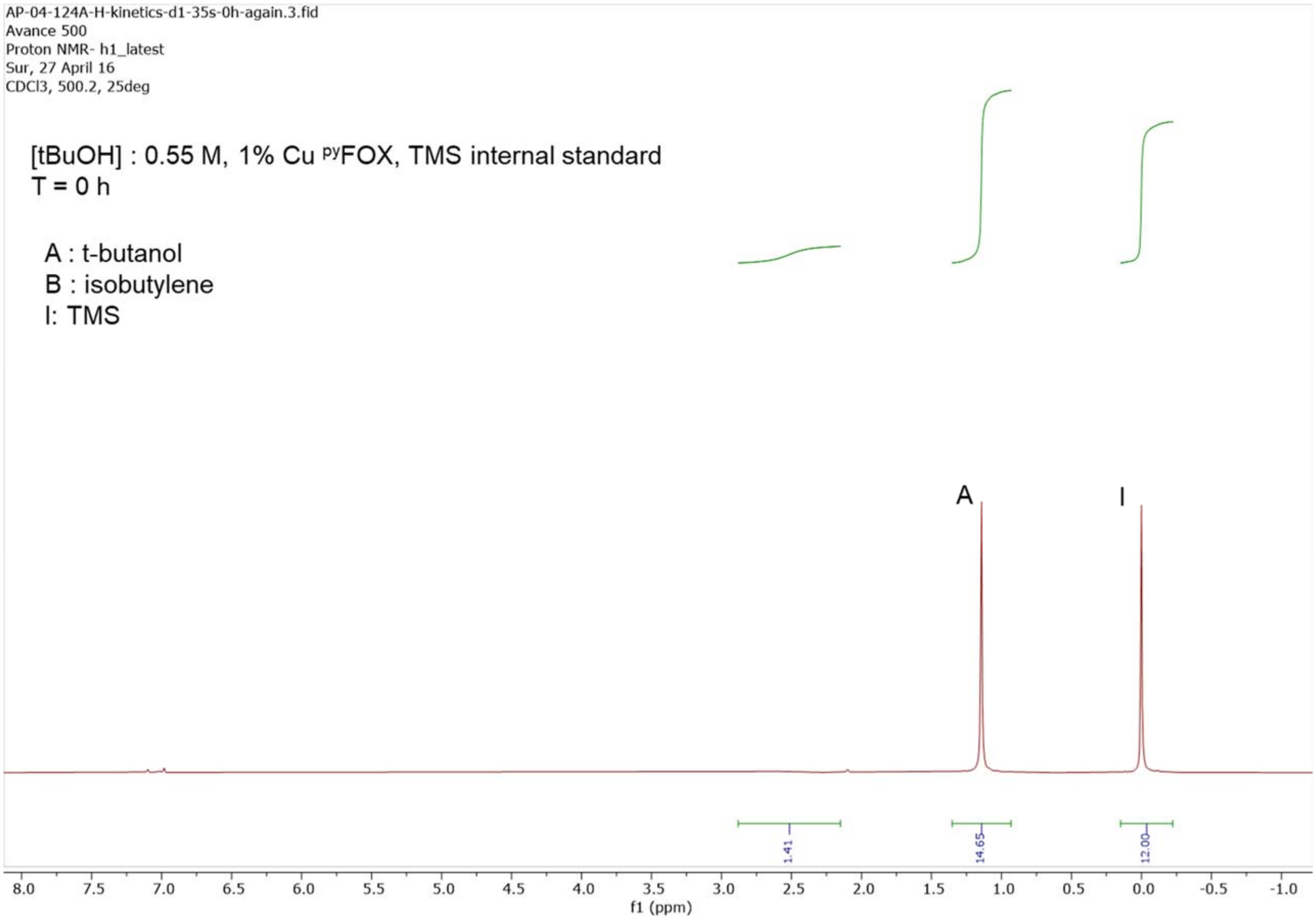

Figure S-24. ^{1}H-NMR Dehydration of tert-butanol (0.83 M), 1% Cu^{py}FOX, toluene-d_8, 120 °C, 41 h

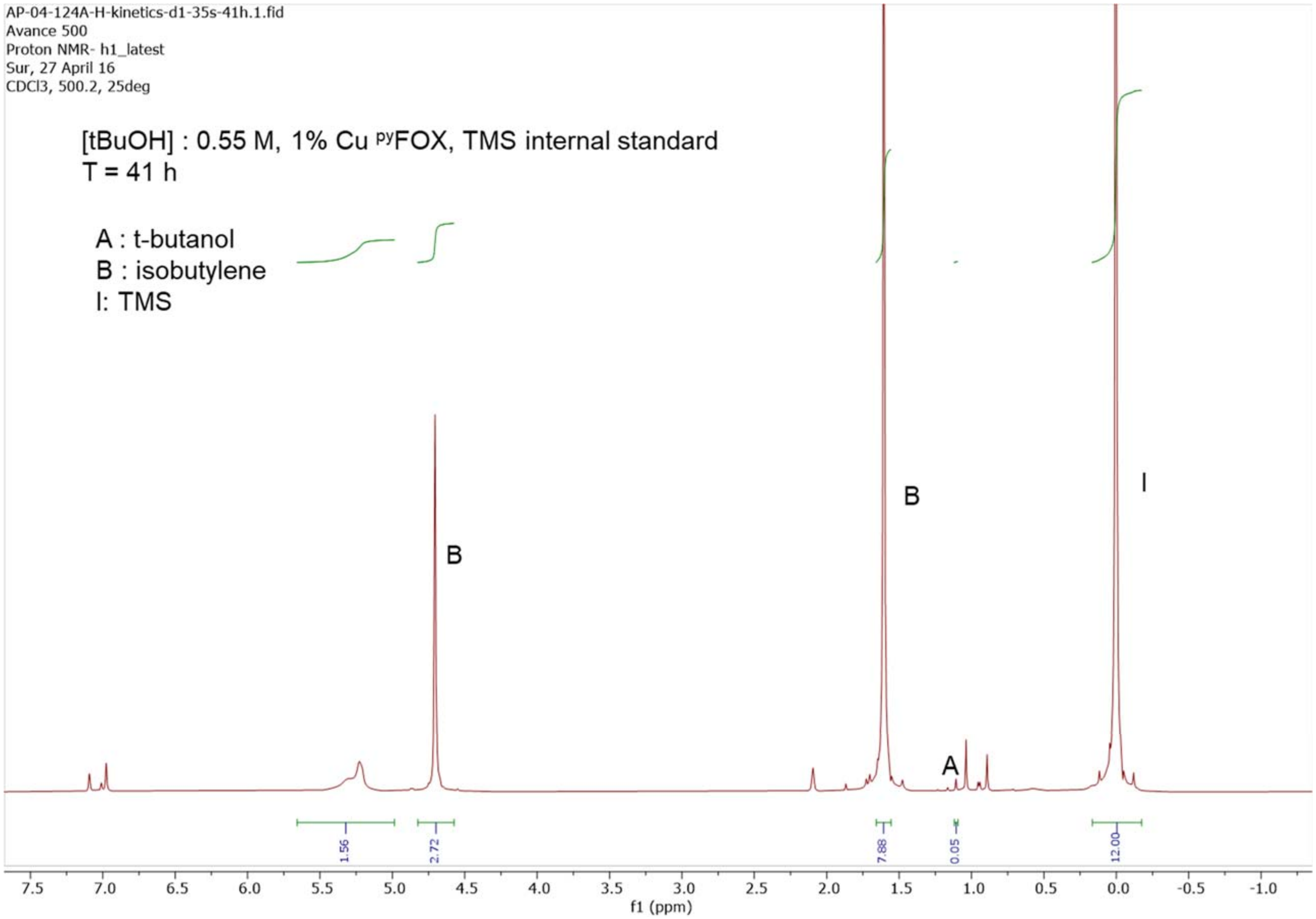

Figure S-25. ^{1}H-NMR Dehydration of tert-butanol (0.83 M), 1% Cu^{py}FOX, toluene-d_8, 120 °C, 19 h

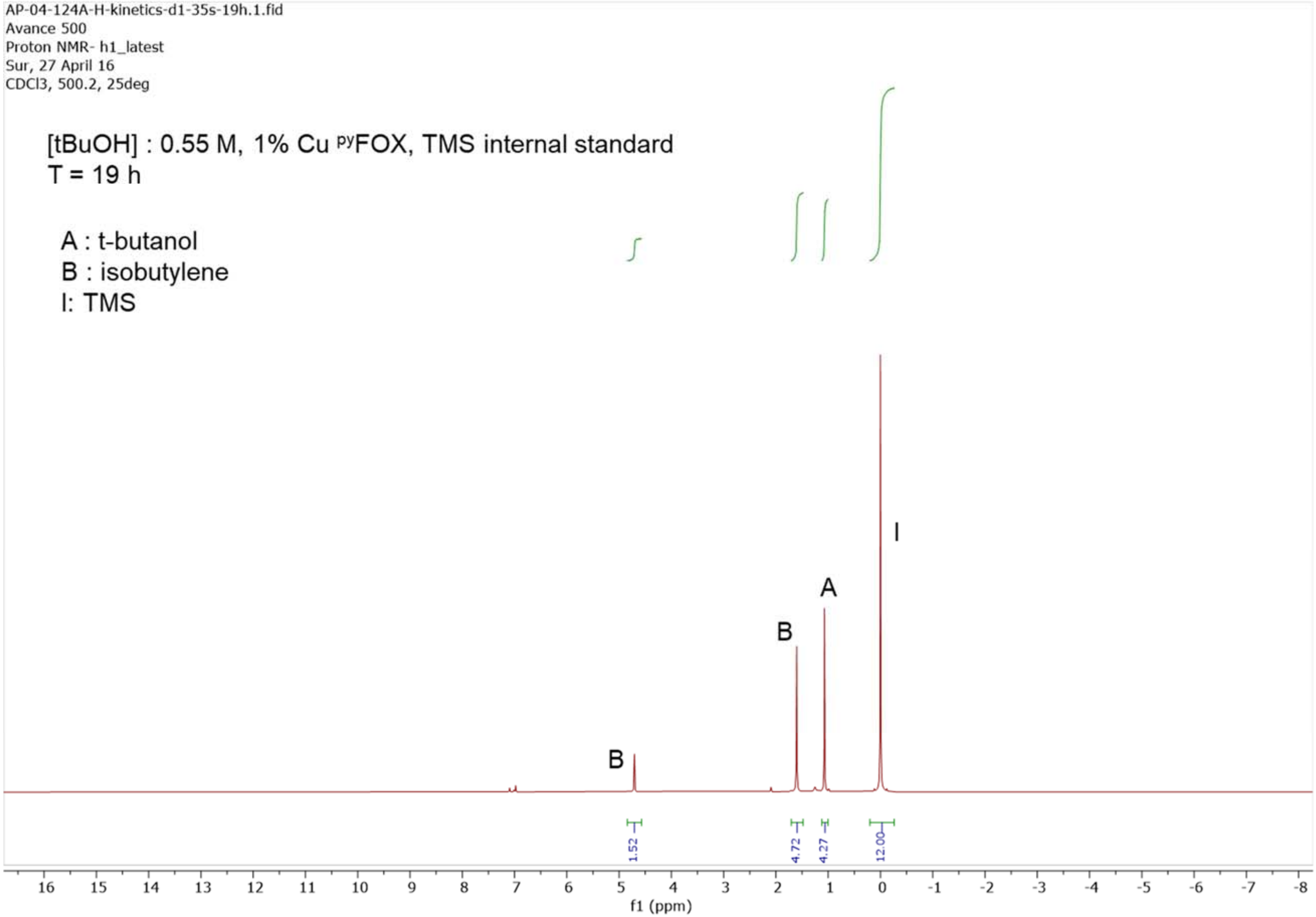

Figure S-26. ^{13}C-NMR Dehydration of tert-butanol (0.83 M), 1% Cu^{py}FOX, toluene-d$_8$, 120 °C, 19 h

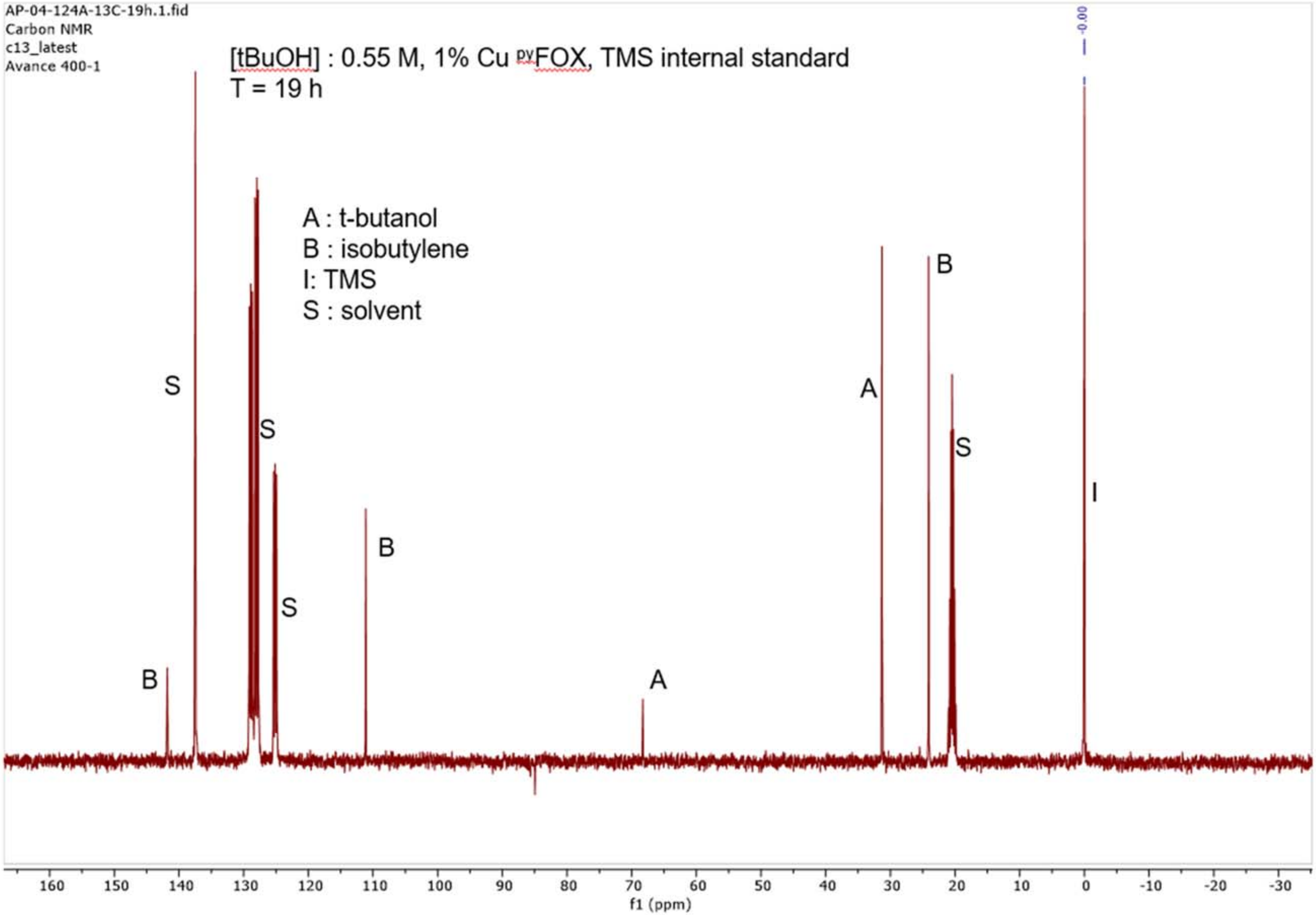

Figure S-27. ^{1}H-NMR Dehydration of tert-butanol (0.83 M), 1% Cu ^{Q}FOX, ODCB-d_4, 120 °C, 0 h

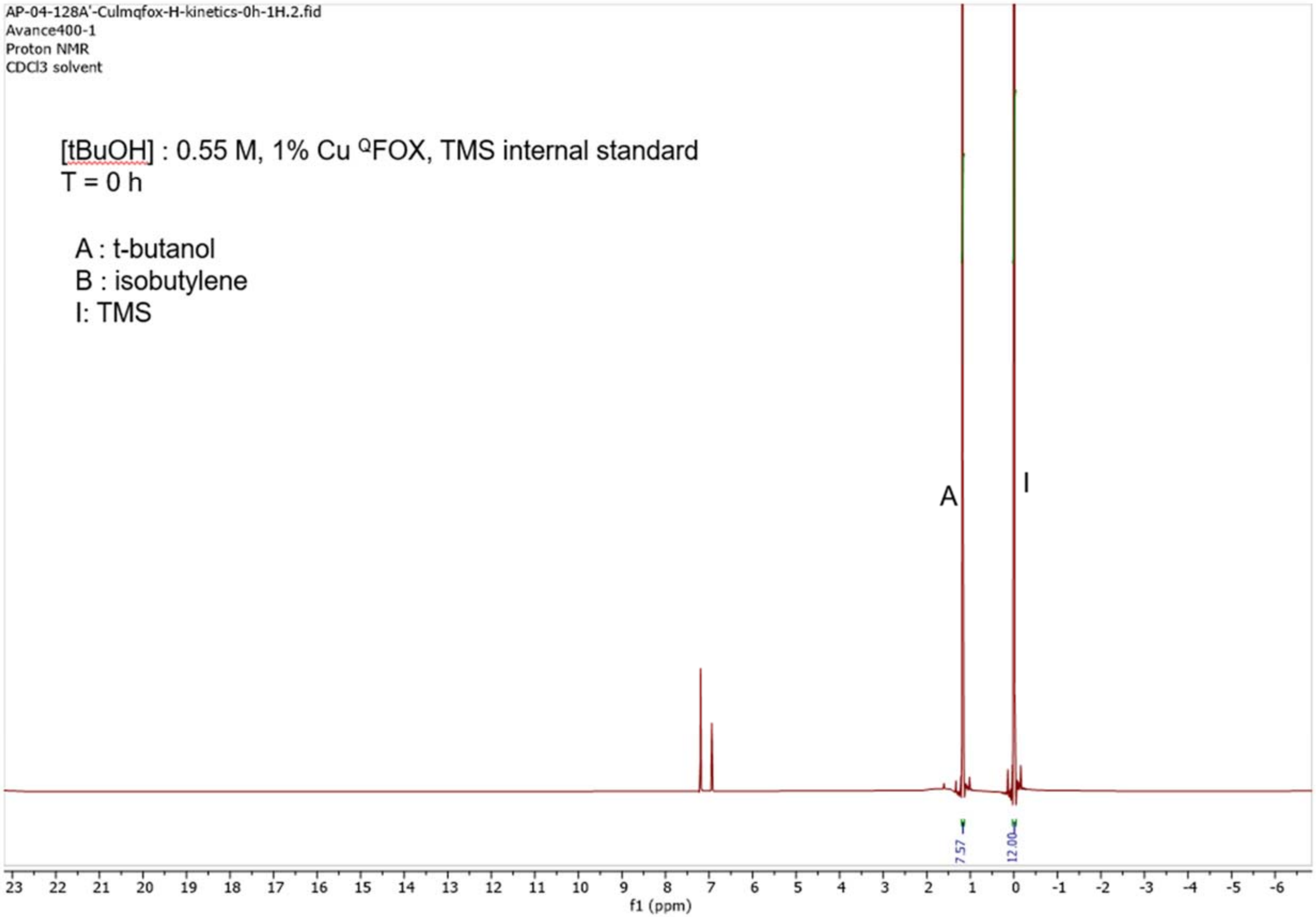

Figure S-28. ^{1}H-NMR Dehydration of tert-butanol (0.83 M), 1% Cu ^{Q}FOX, ODCB-d_4, 120 °C, 41 h

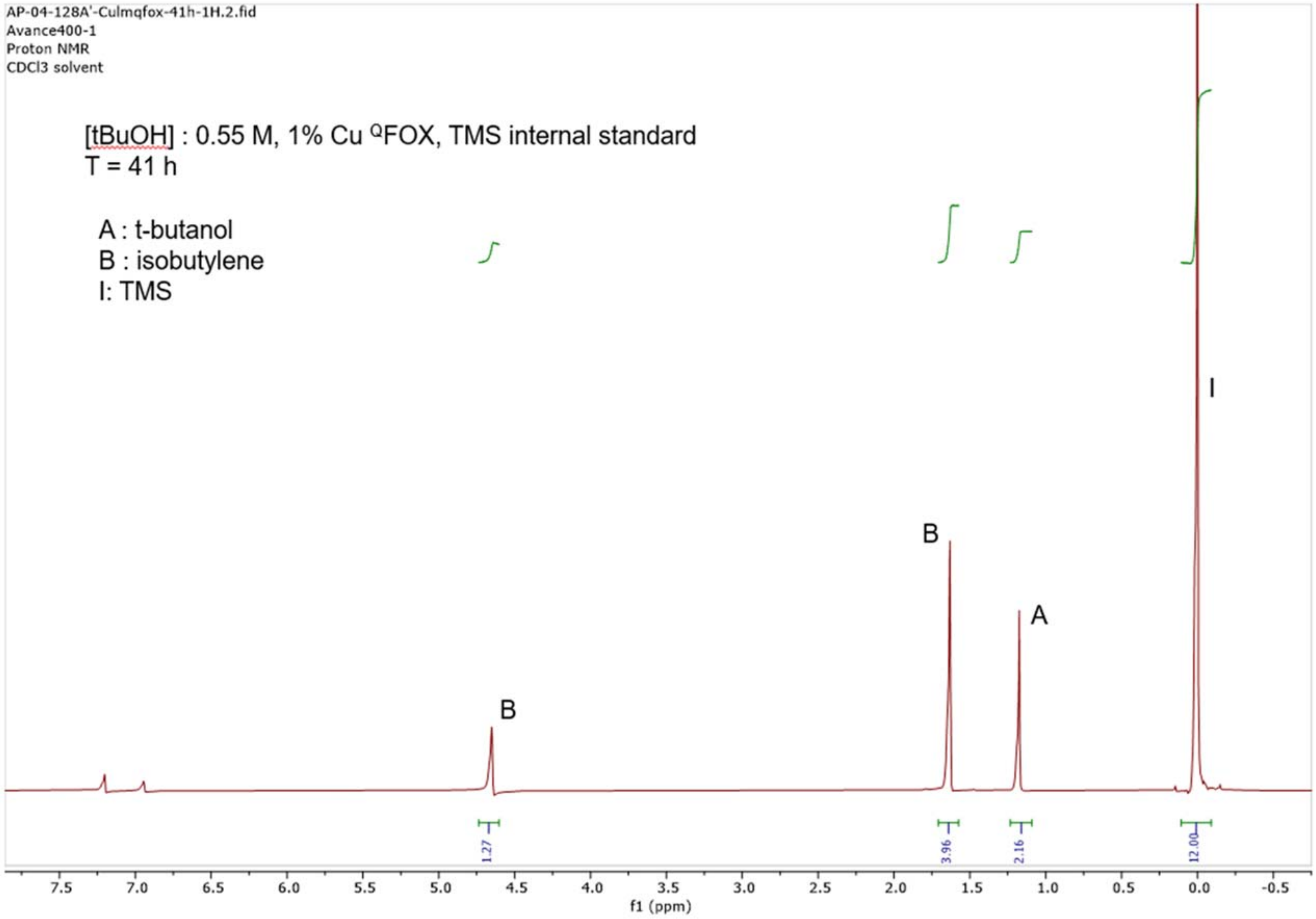

Figure S-29. ^{1}H-NMR Dehydration of tert-butanol (0.83 M), 1% Cu ^{Q}FOX, ODCB-d_4, 120 °C, 23 h

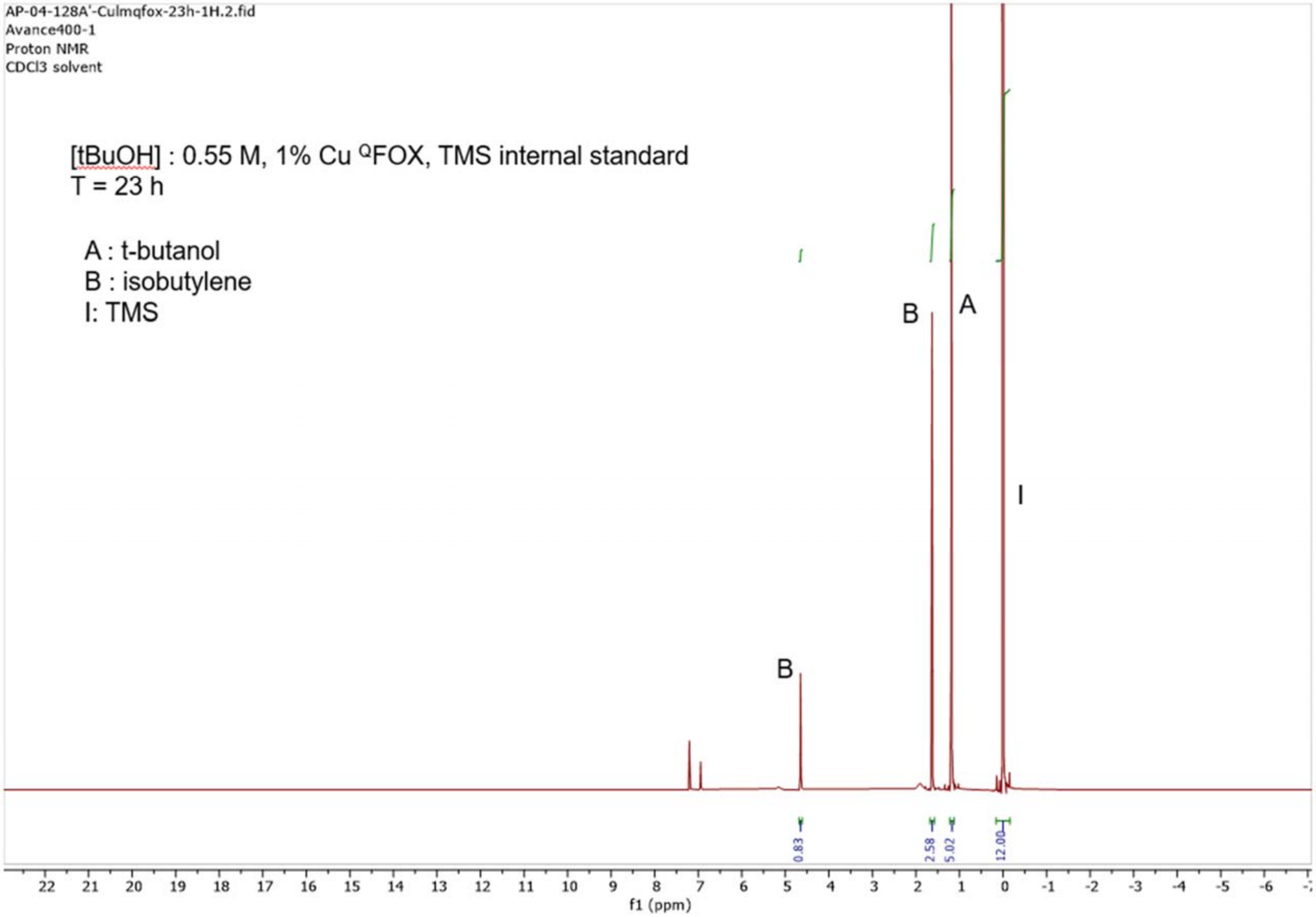

Figure S-30. ^{13}C-NMR Dehydration of tert-butanol (0.83 M), 1% Cu^{Q}FOX, ODCB-d$_4$, 120 °C, 23 h

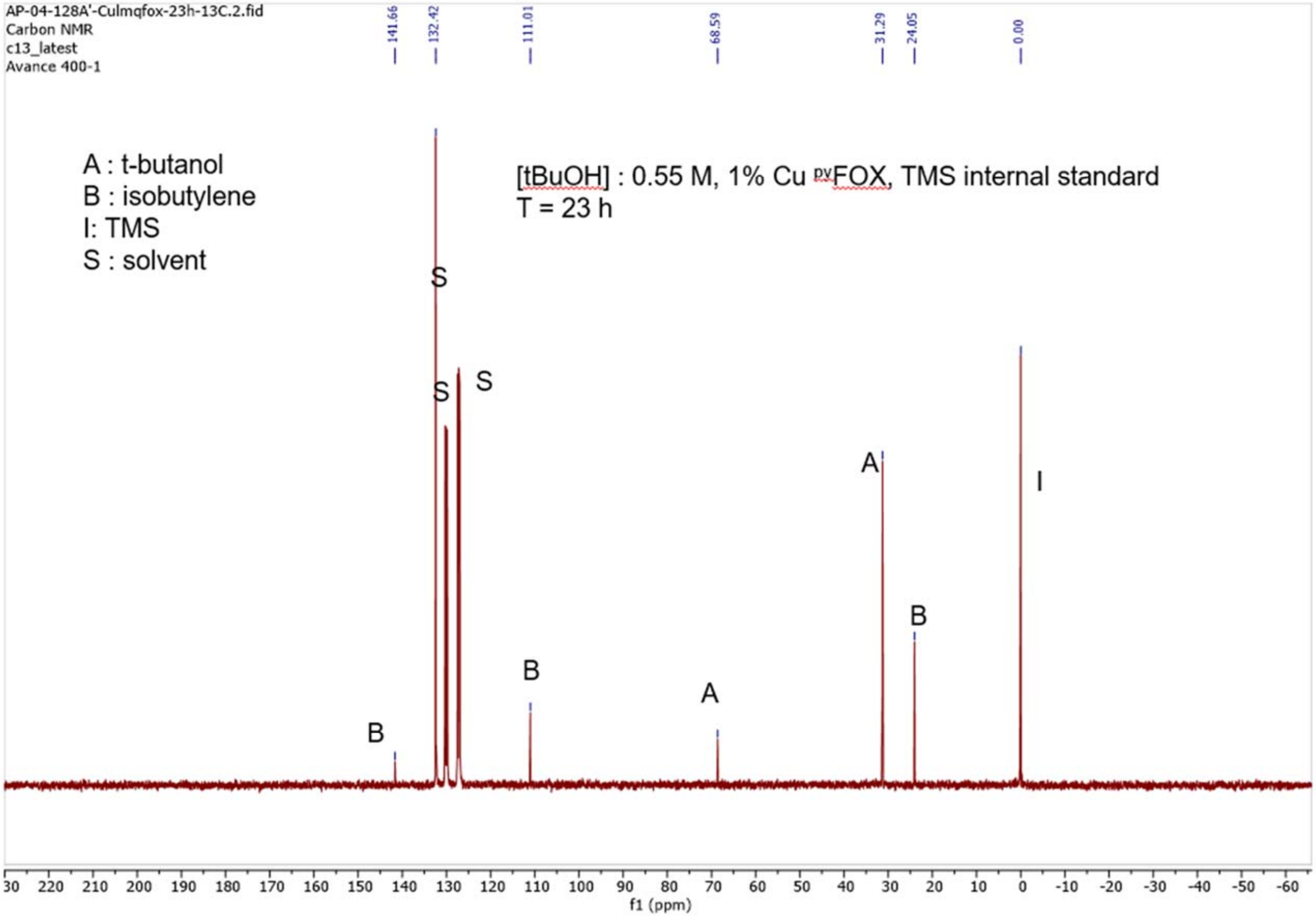

Figure S-31. ^{1}H-NMR Dehydration of 2-phenylisopropanol (0.83 M), 1% Cu^{Q}FOX, ODCB-d$_4$, 120 °C, 0 h

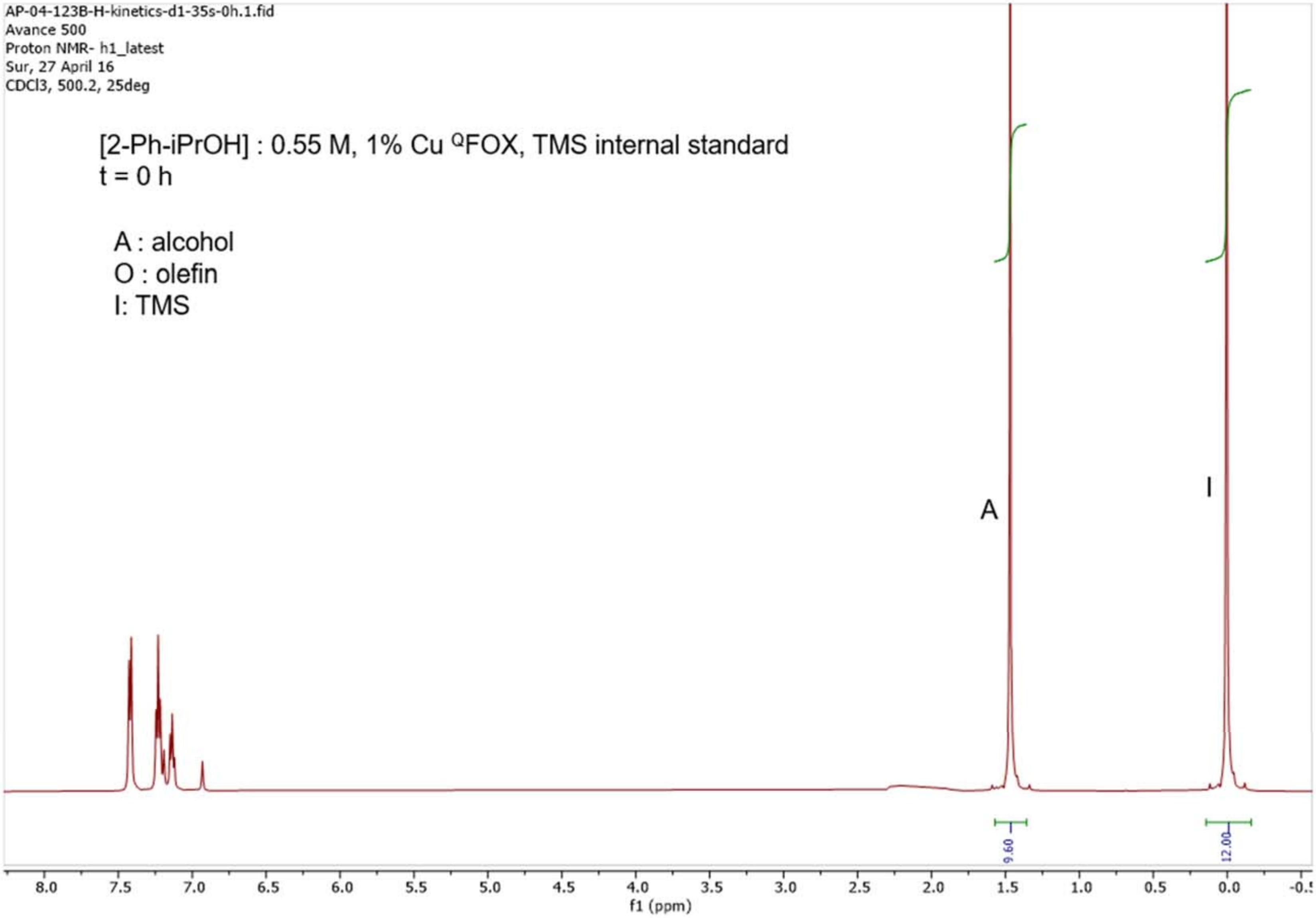

Figure S-32. ^{1}H-NMR Dehydration of 2-phenylisopropanol (0.83 M), 1% Cu^{Q}FOX, ODCB-d_4, 120 °C, 5 h

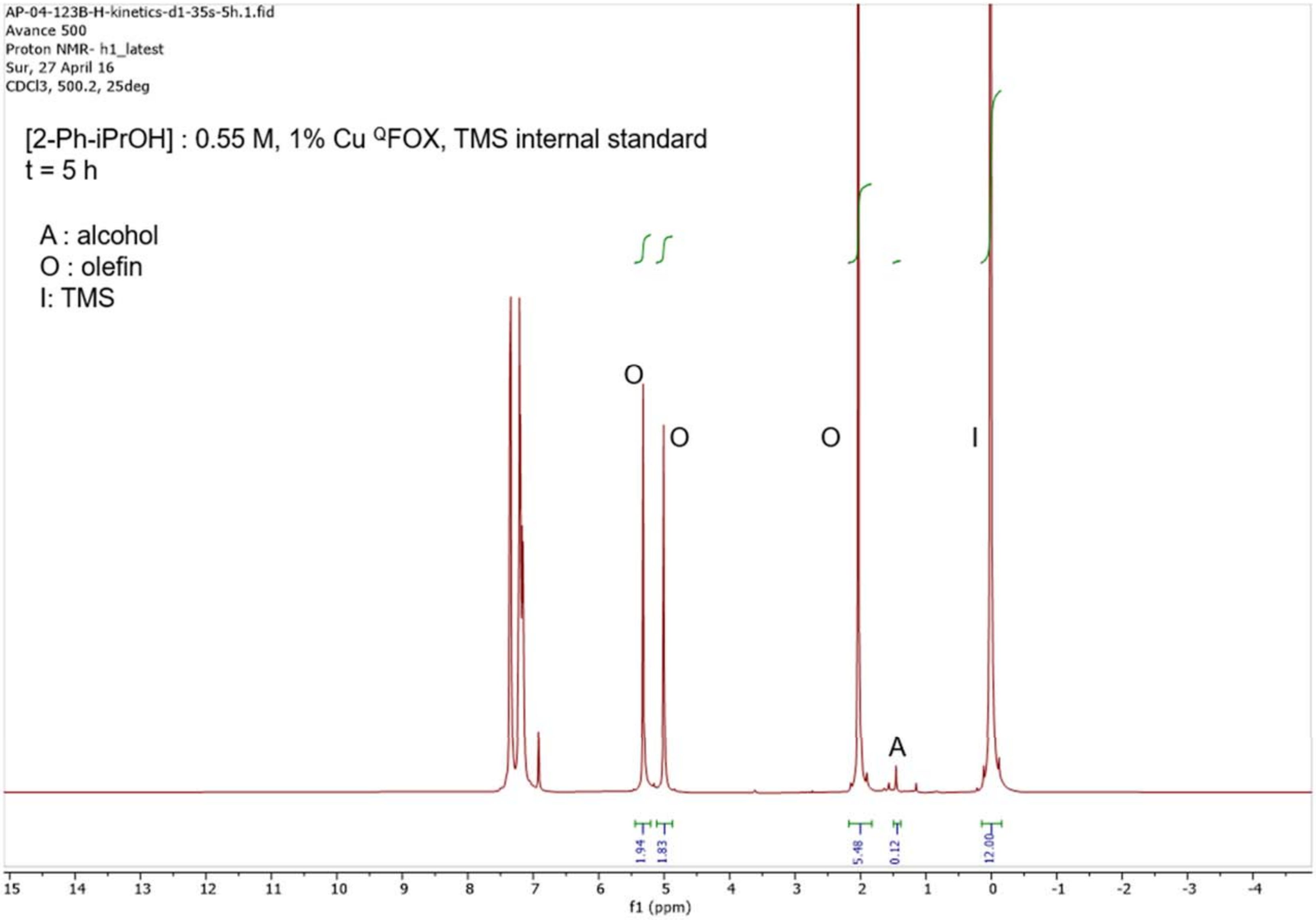

Figure S-33. ^{1}H-NMR Dehydration of 2-phenylisopropanol (0.83 M), 1% Cu^{py}FOX, toluene-d_8, 120 °C, 0 h

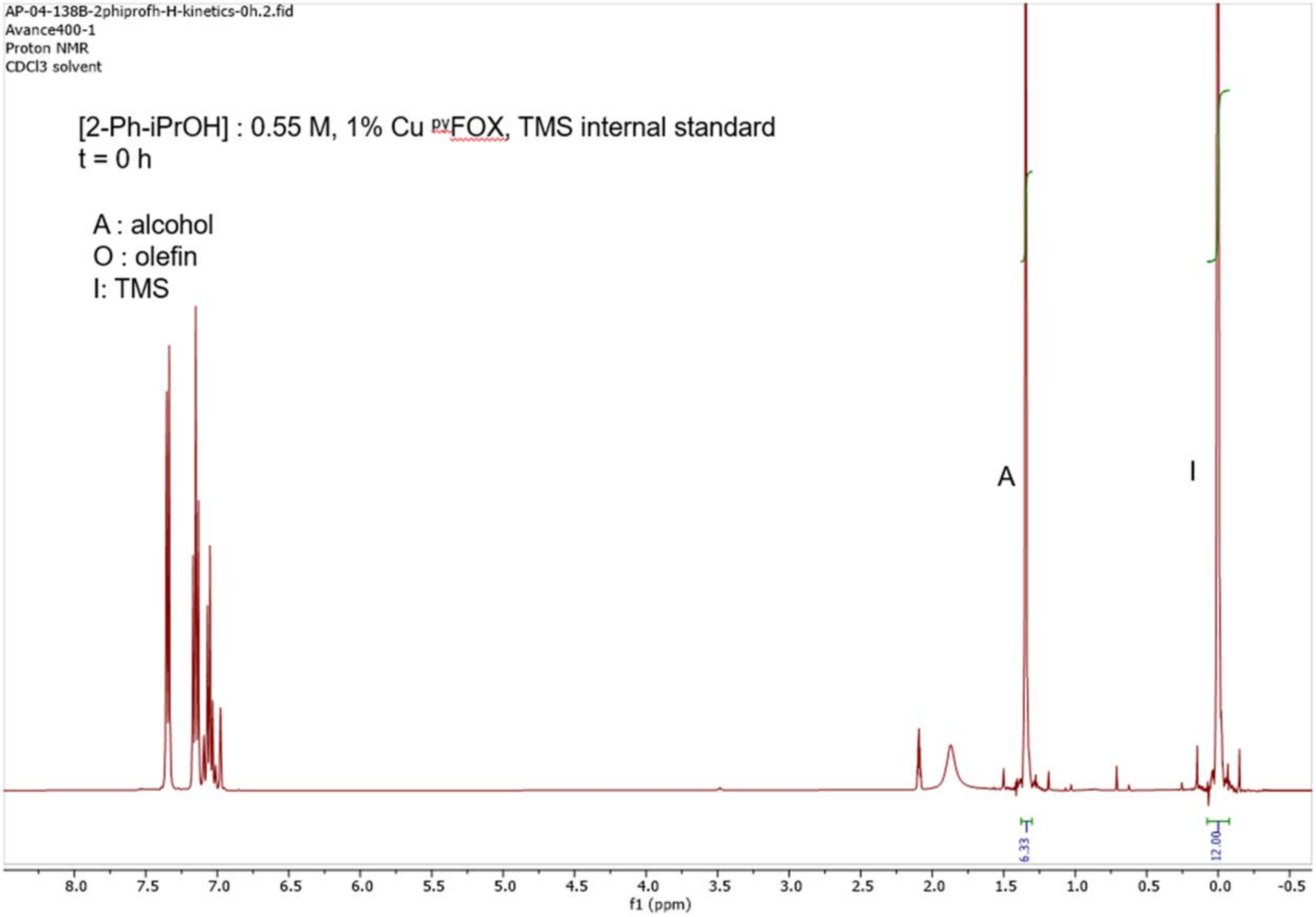

Figure S-34. ^{1}H-NMR Dehydration of 2-phenylisopropanol (0.83 M), 1% Cu^{py}FOX, toluene-d_8, 120 °C, 5 h

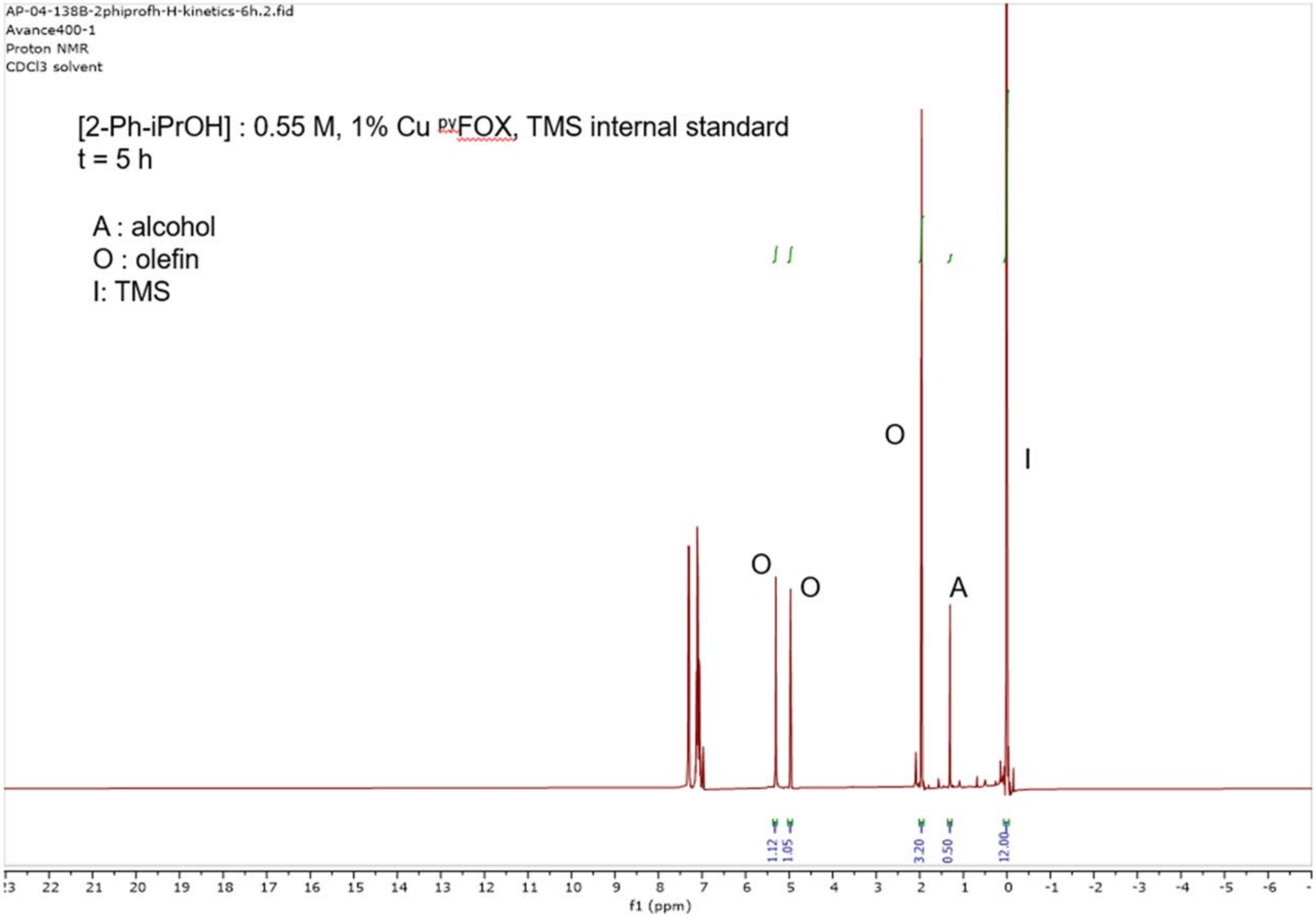

Figure S-35. ^{1}H-NMR Dehydration of 2-Me-2-pentanol (0.83 M), 1% Cu^{py}FOX, toluene-d$_8$, 120 °C, 0 h

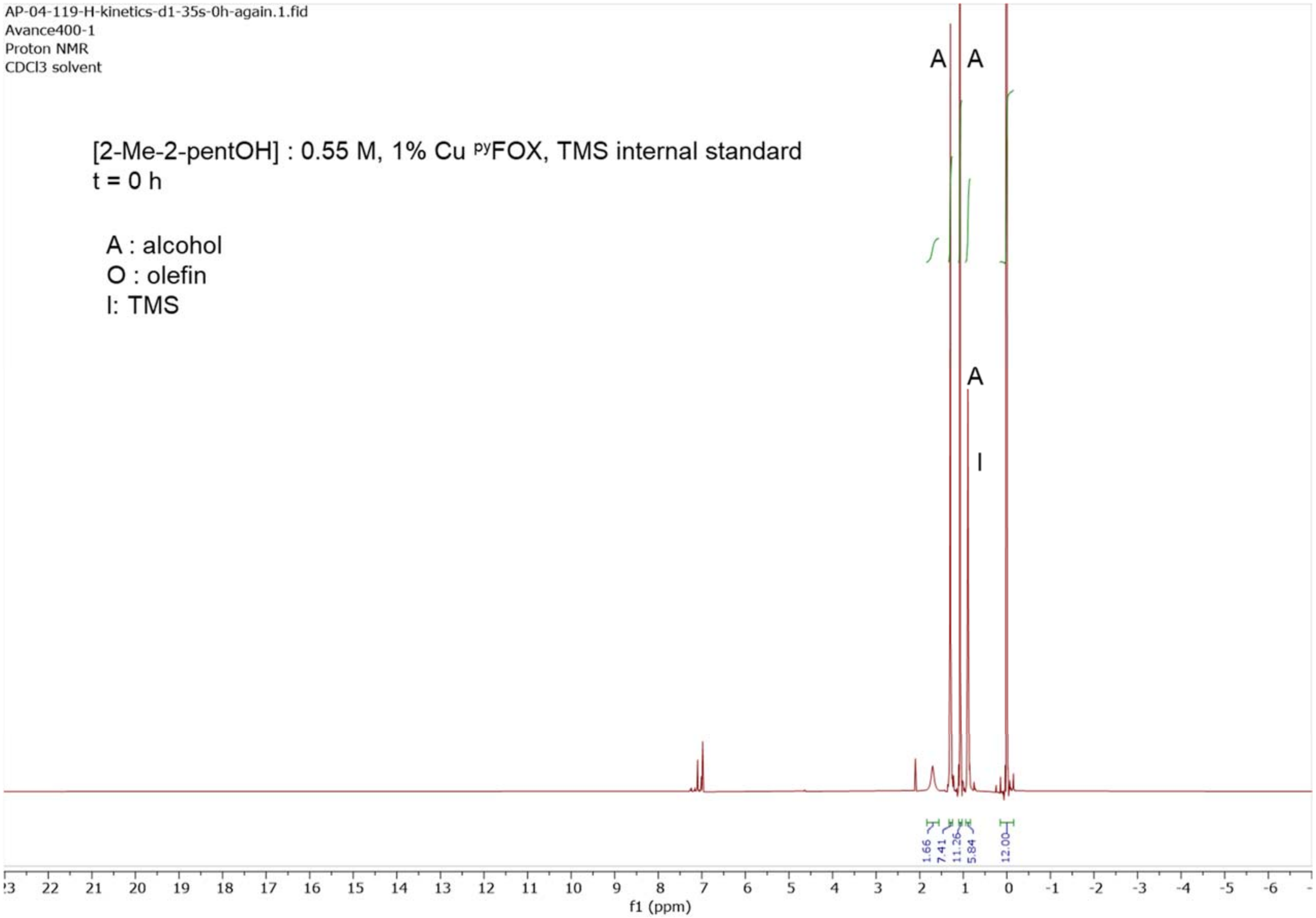

Figure S-36. ^{1}H-NMR Dehydration of 2-Me-2-pentanol (0.83 M), 1% Cu^{py}FOX, toluene-d_8, 120 °C, 64 h

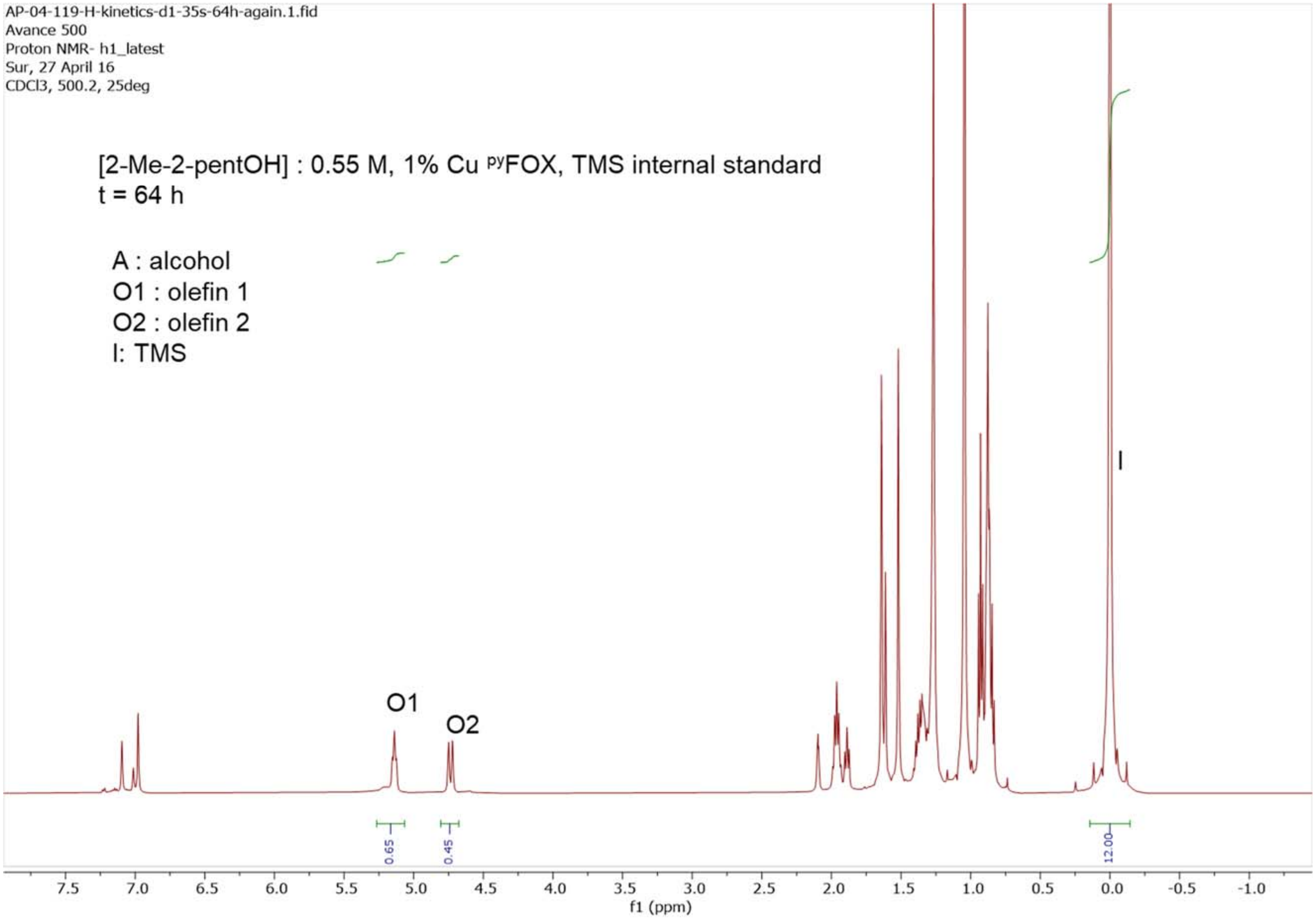

Figure S-37. ^{1}H-NMR Dehydration of 2-Me-2-pentanol (0.83 M), 1% Cu^{Q}FOX, ODCB-d_4, 120 °C, 0 h

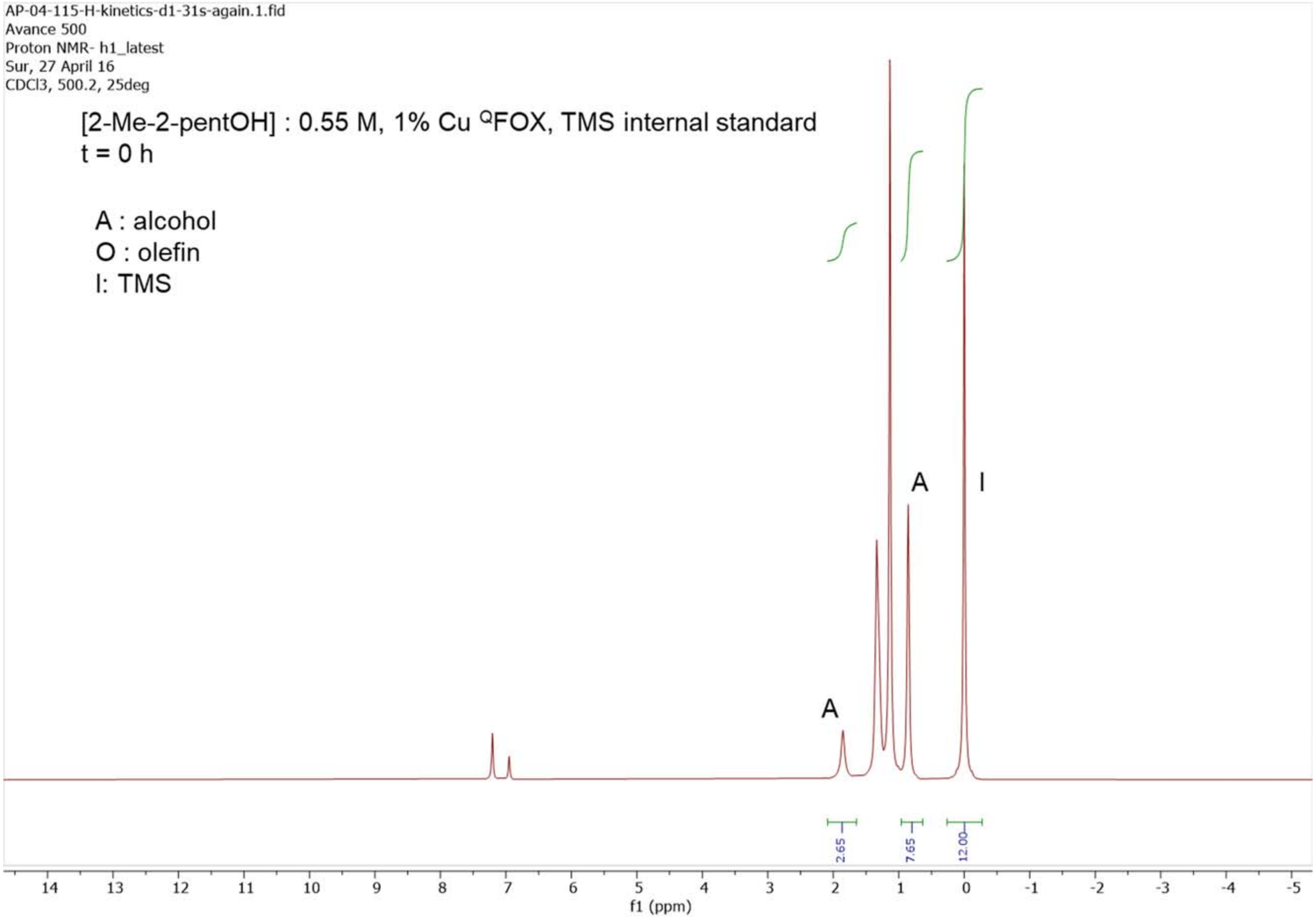

Figure S-38. ^{1}H-NMR Dehydration of 2-Me-2-pentanol (0.83 M), 1% Cu^{Q}FOX, ODCB-d_4, 120 °C, 64 h

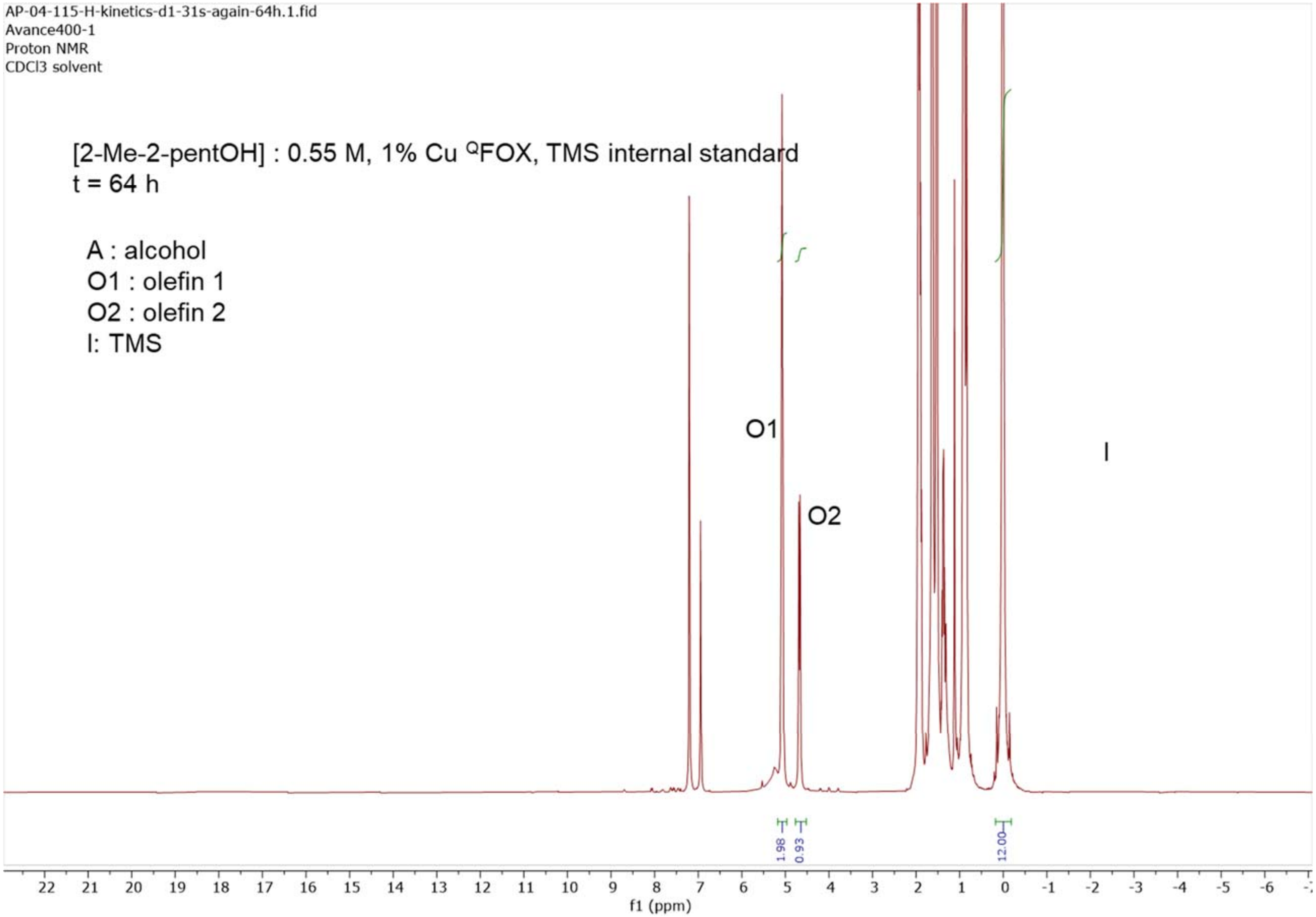

Figure S-39. ^{1}H NMR spectrum of (^{Q}FOX)$MnBr_2$ in CD_3OD

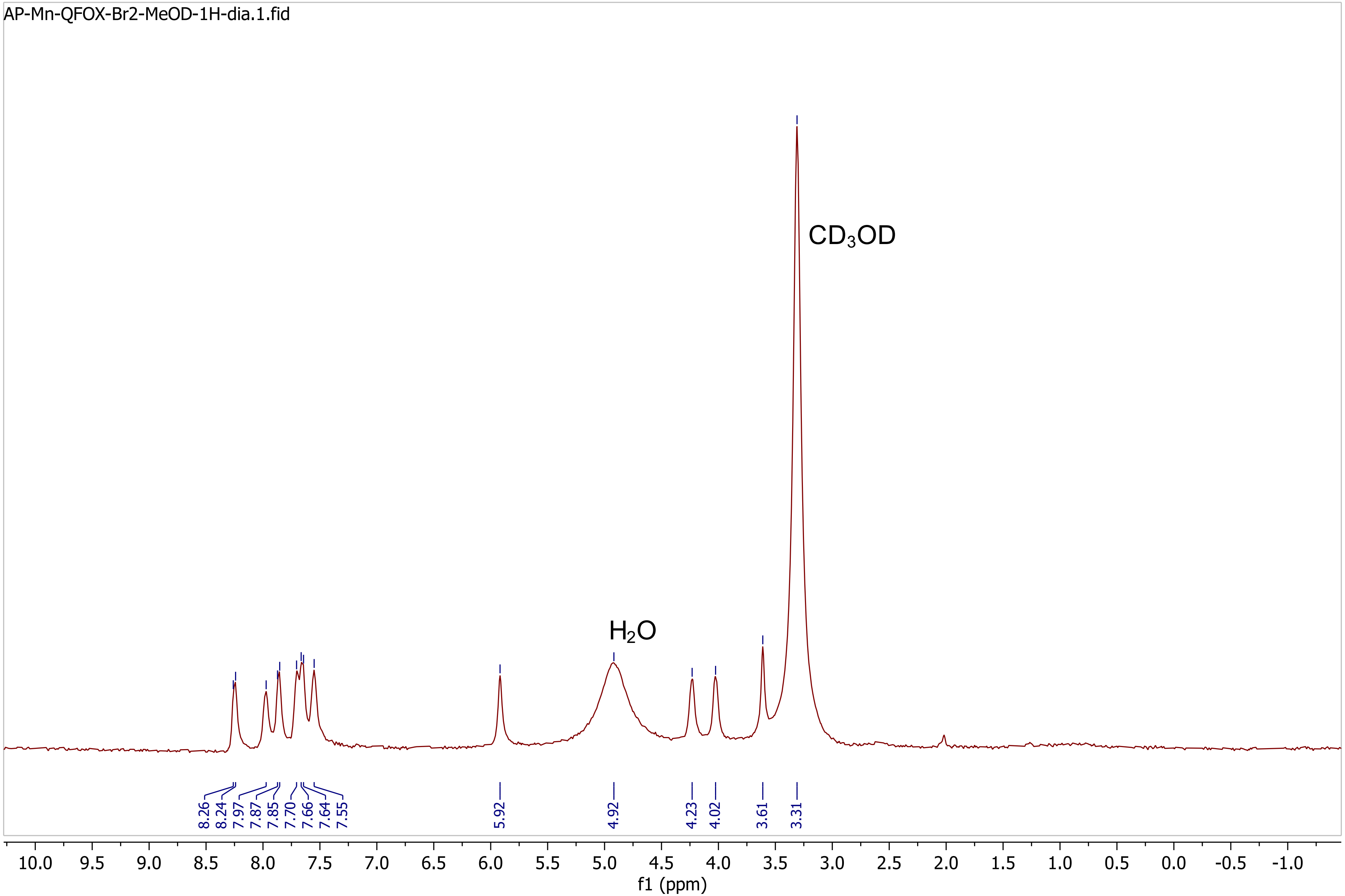

Figure S-40. ^{1}H NMR spectrum of (^{Q}FOX)$FeBr_2$ in CD_3OD

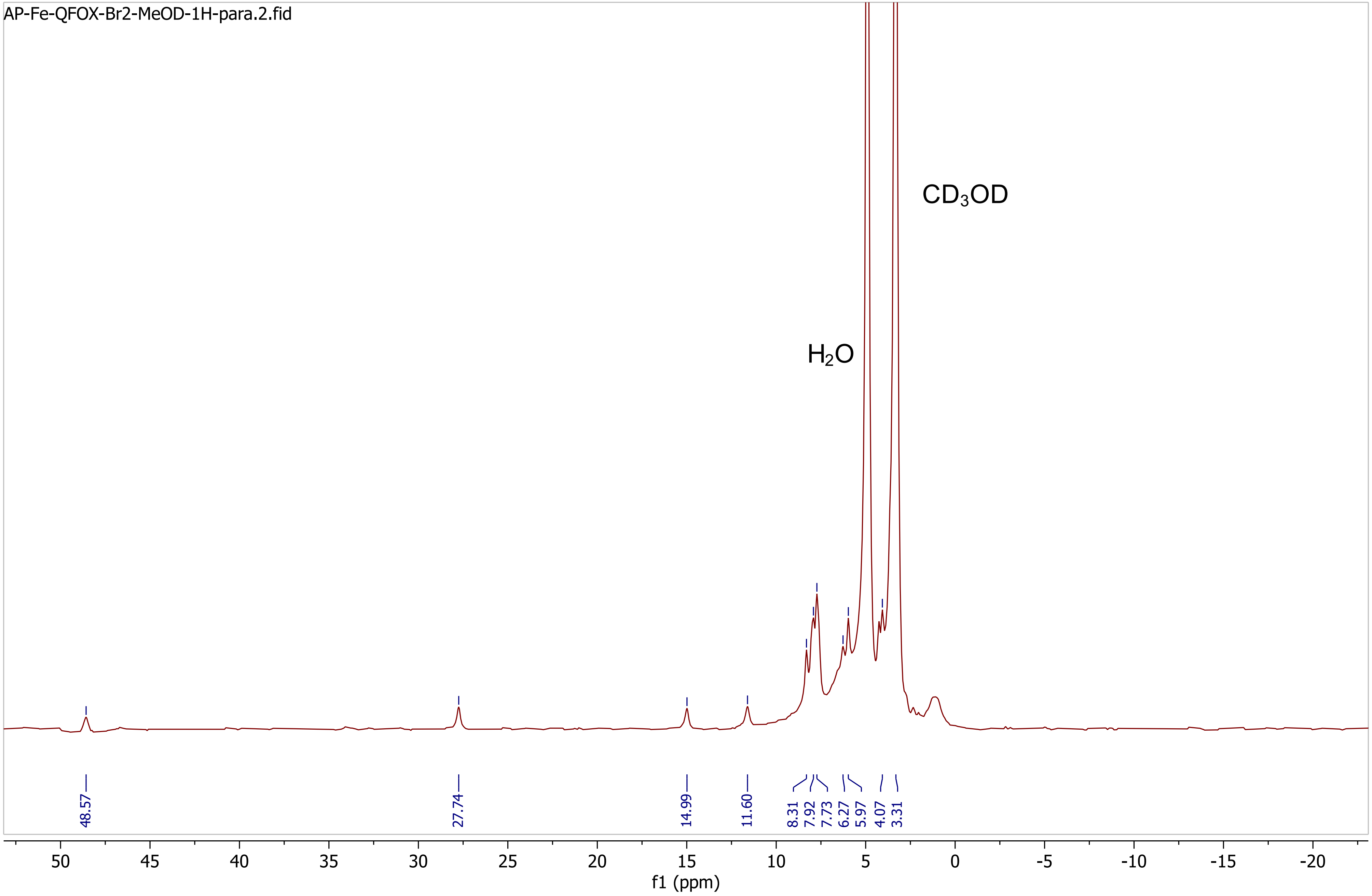

Figure S-41. ^{1}H NMR spectrum of (^{Q}FOX)$CoBr_2$ in CD_3OD

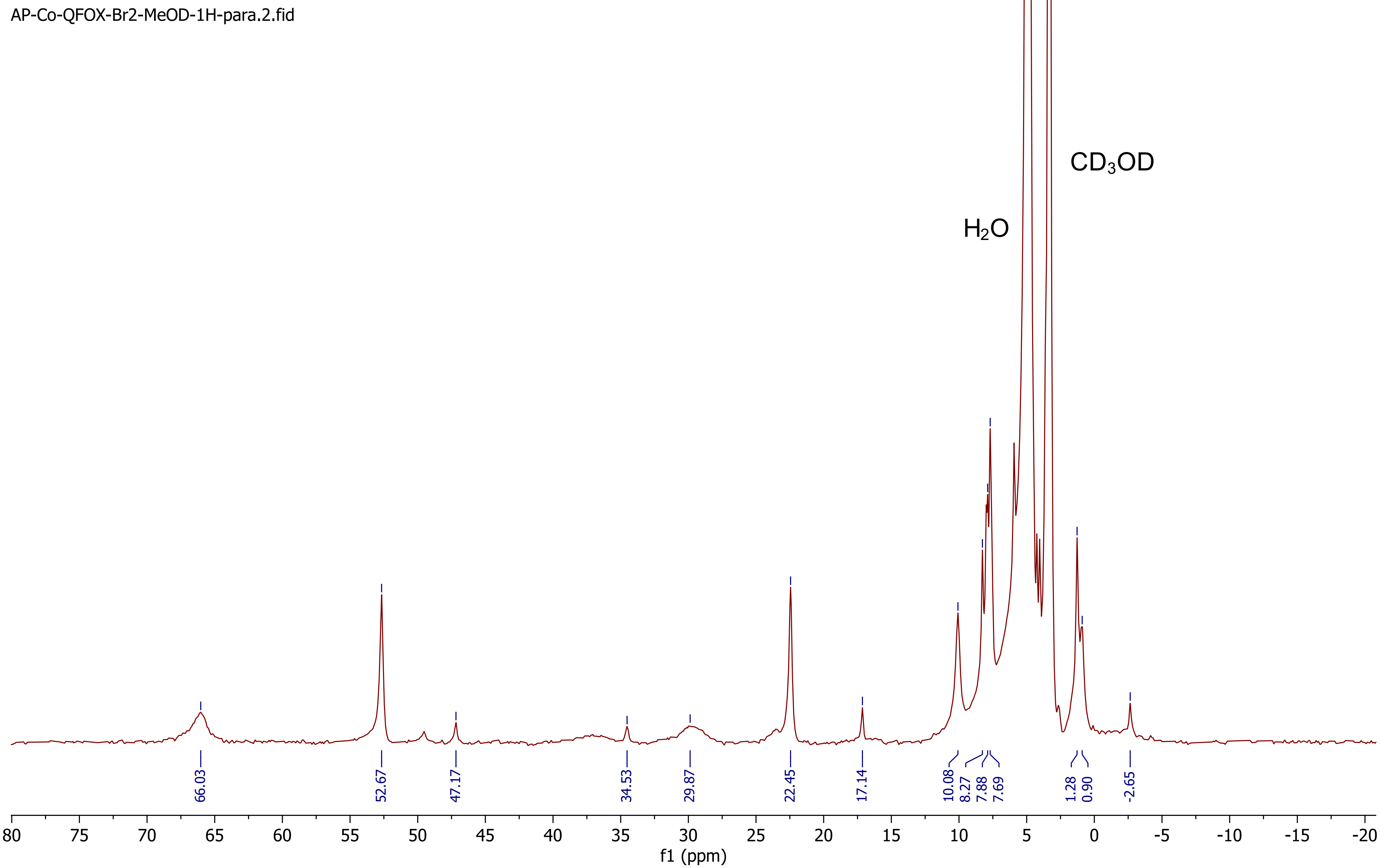

Figure S-42. ^{1}H NMR spectrum of (^{Q}FOX)NiBr$_2$ in CD_3OD

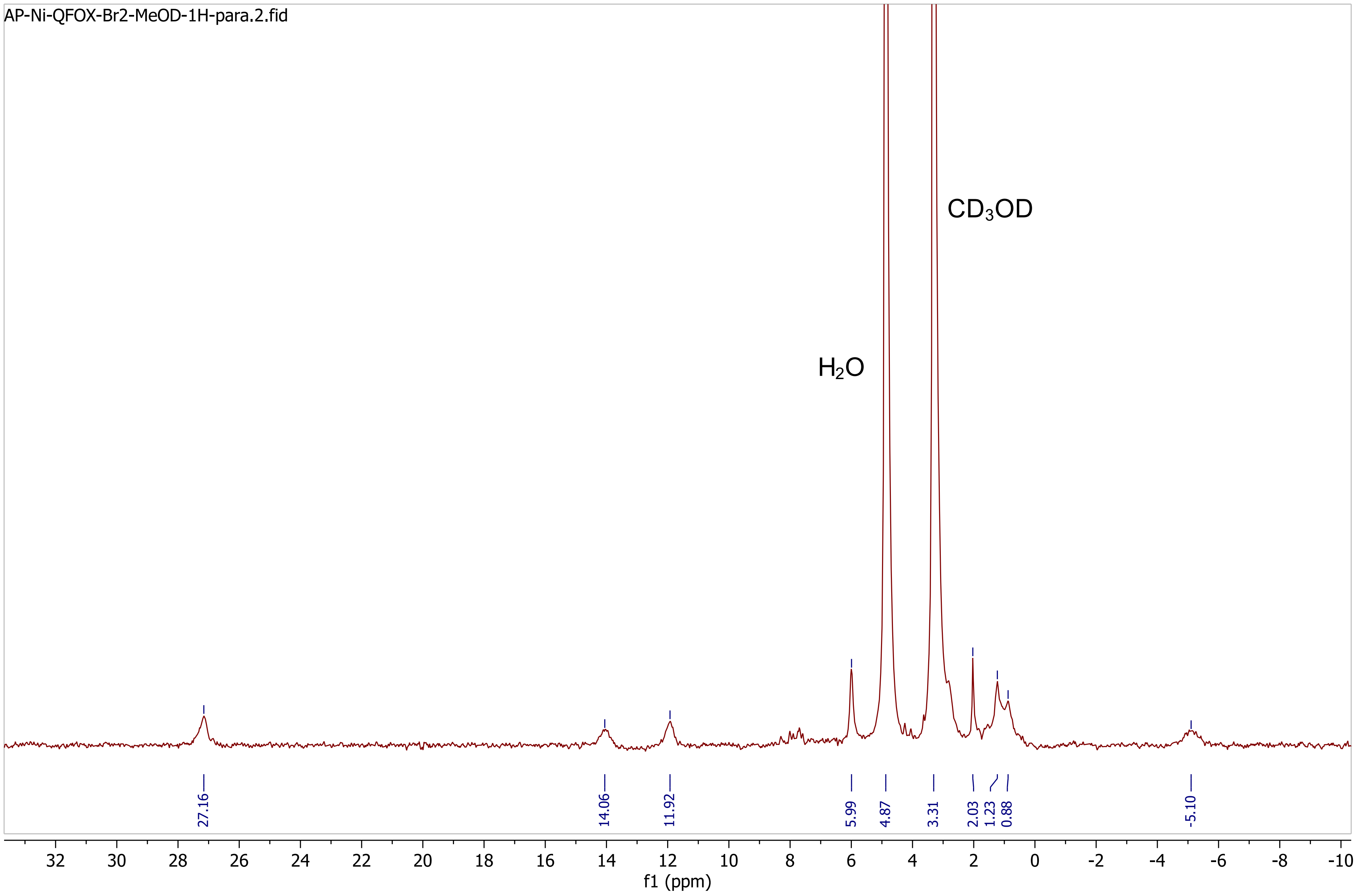

Figure S-43. ^{1}H NMR spectrum of (^{Q}FOX)$CuBr_2$ in CD_3OD

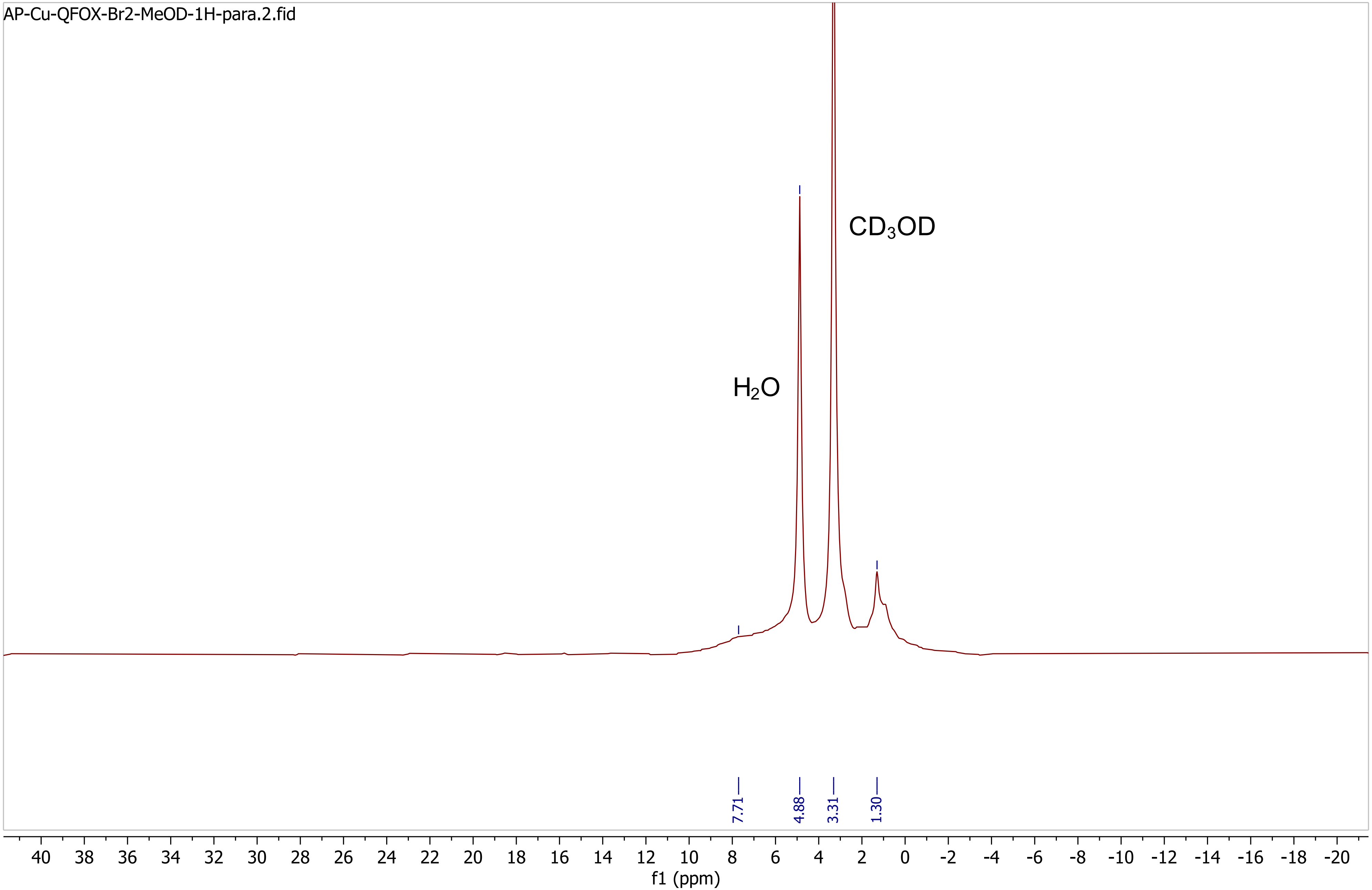

Figure S-44. ^{1}H NMR spectrum of (^{Q}FOX)Mn(OTf)$_2$ in CD_3OD

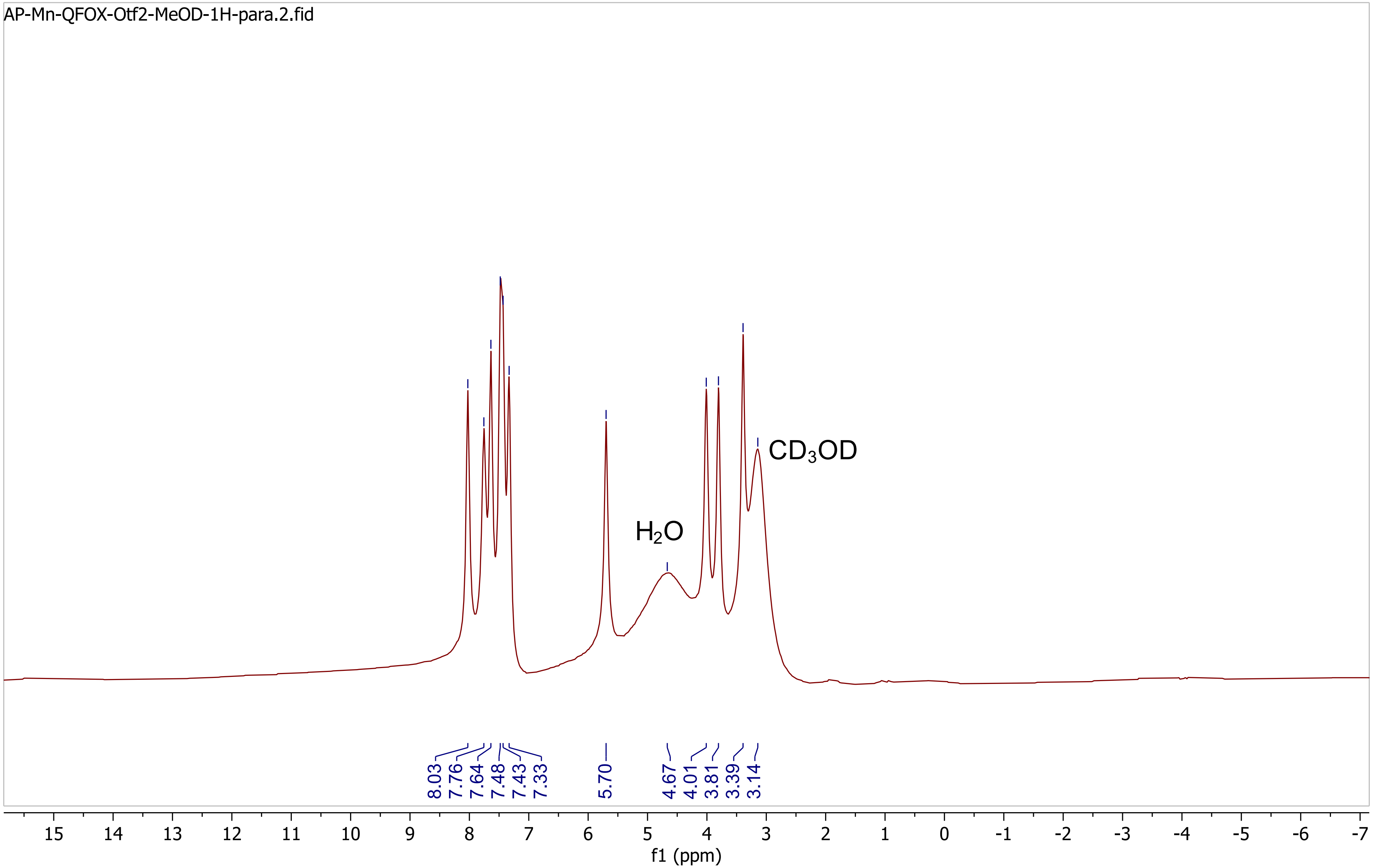

Figure S-45. ^{1}H NMR spectrum of (^{Q}FOX)$Fe(OTf)_2$ in CD_3OD

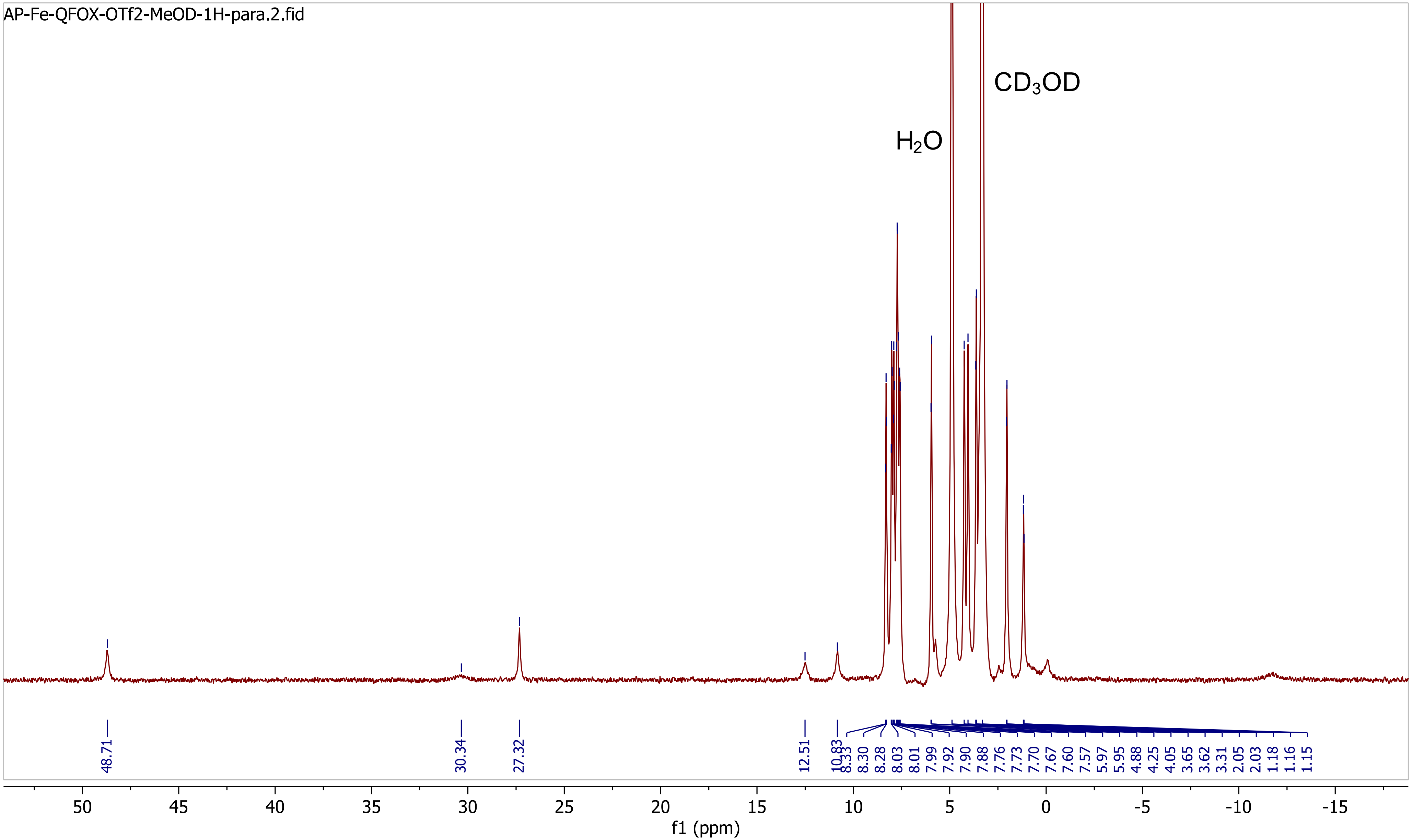

Figure S-46. ^{1}H NMR spectrum of $(^{Q}FOX)Co(OTf)_2$ in CD_3OD

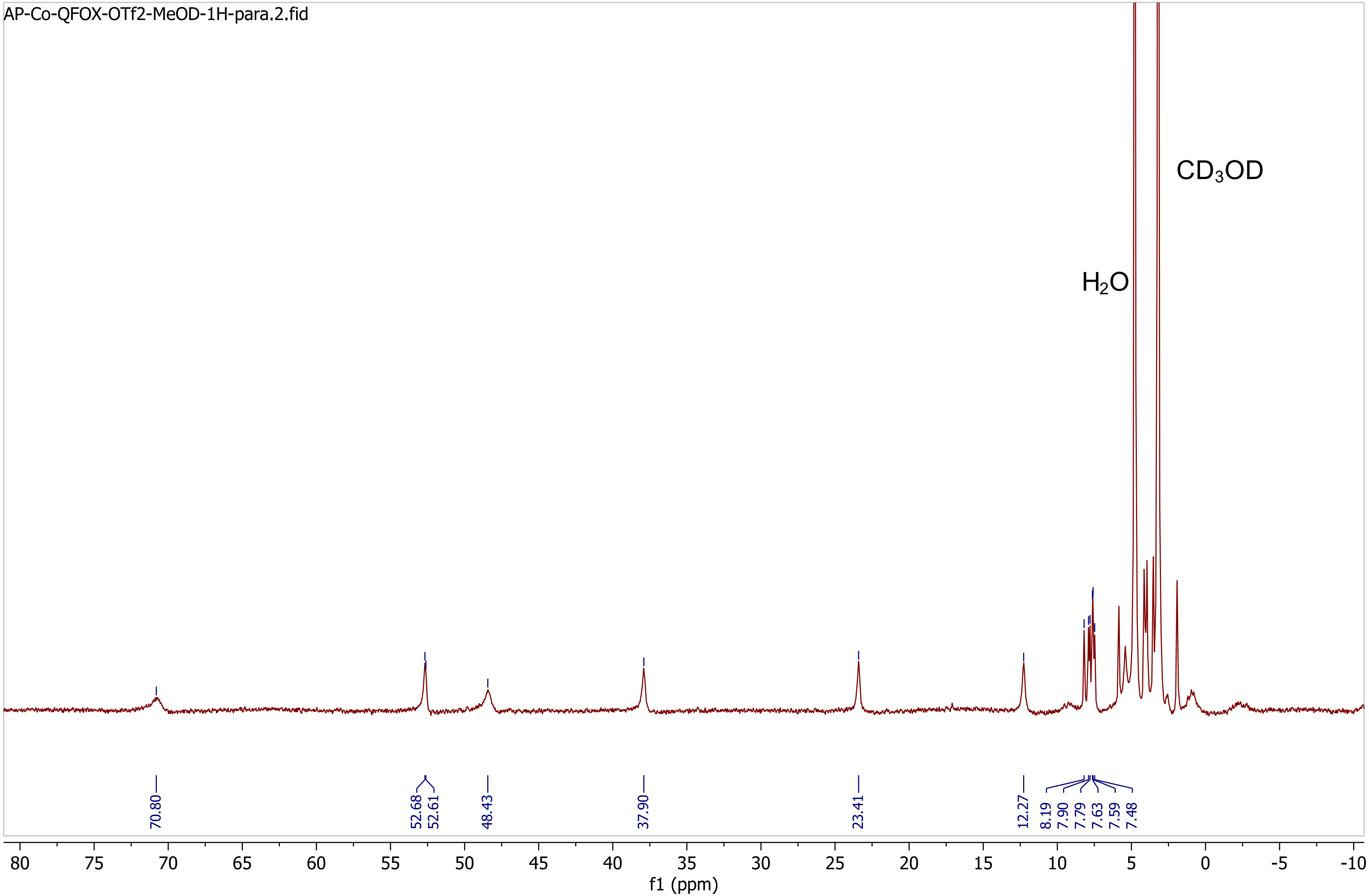

Figure S-47. ^{1}H NMR spectrum of (^{Q}FOX)Ni(OTf)$_2$ in CD_3OD

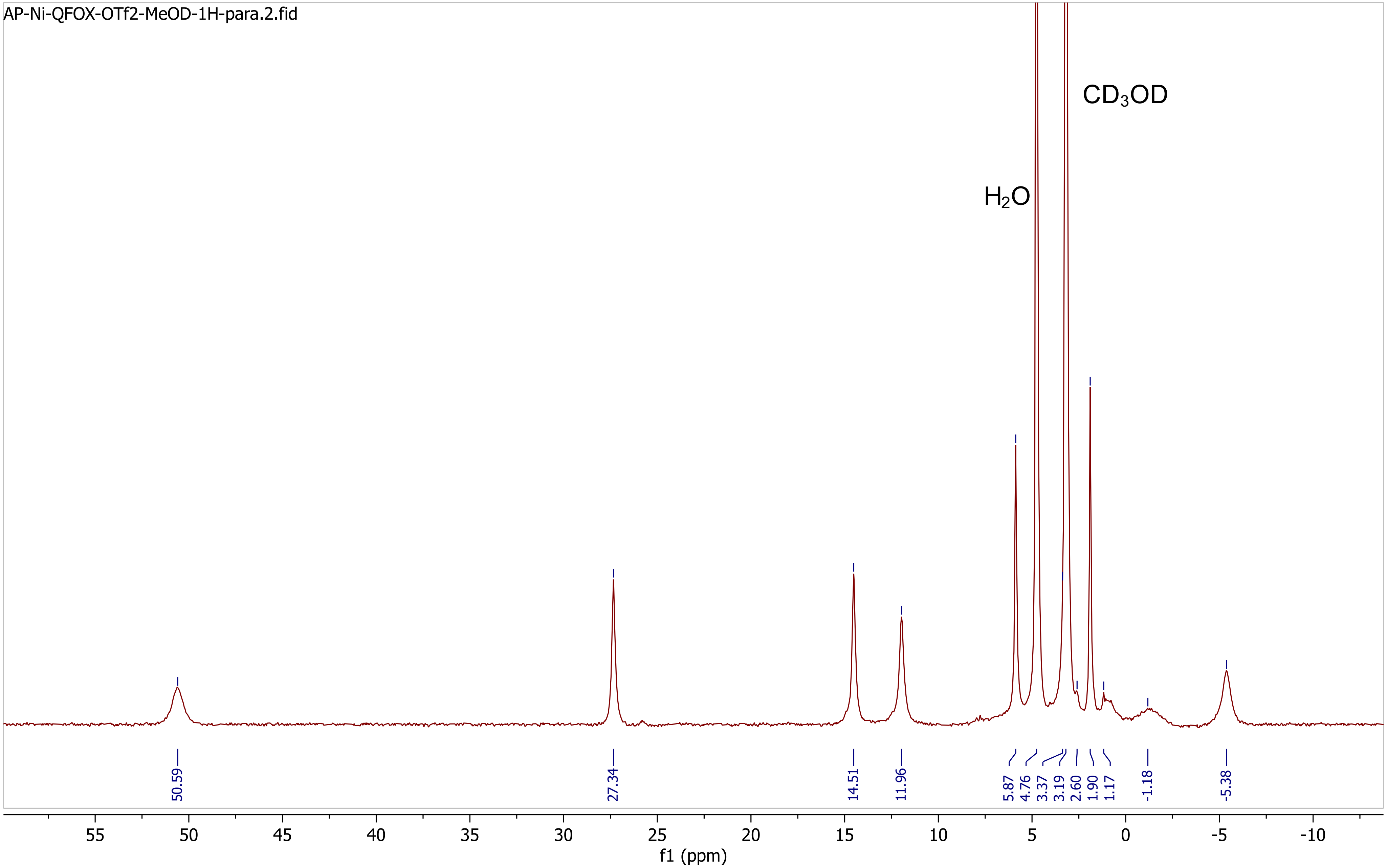

Figure S-48. ^{1}H NMR spectrum of (^{Q}FOX)Cu(OTf)$_2$ in CD_3OD

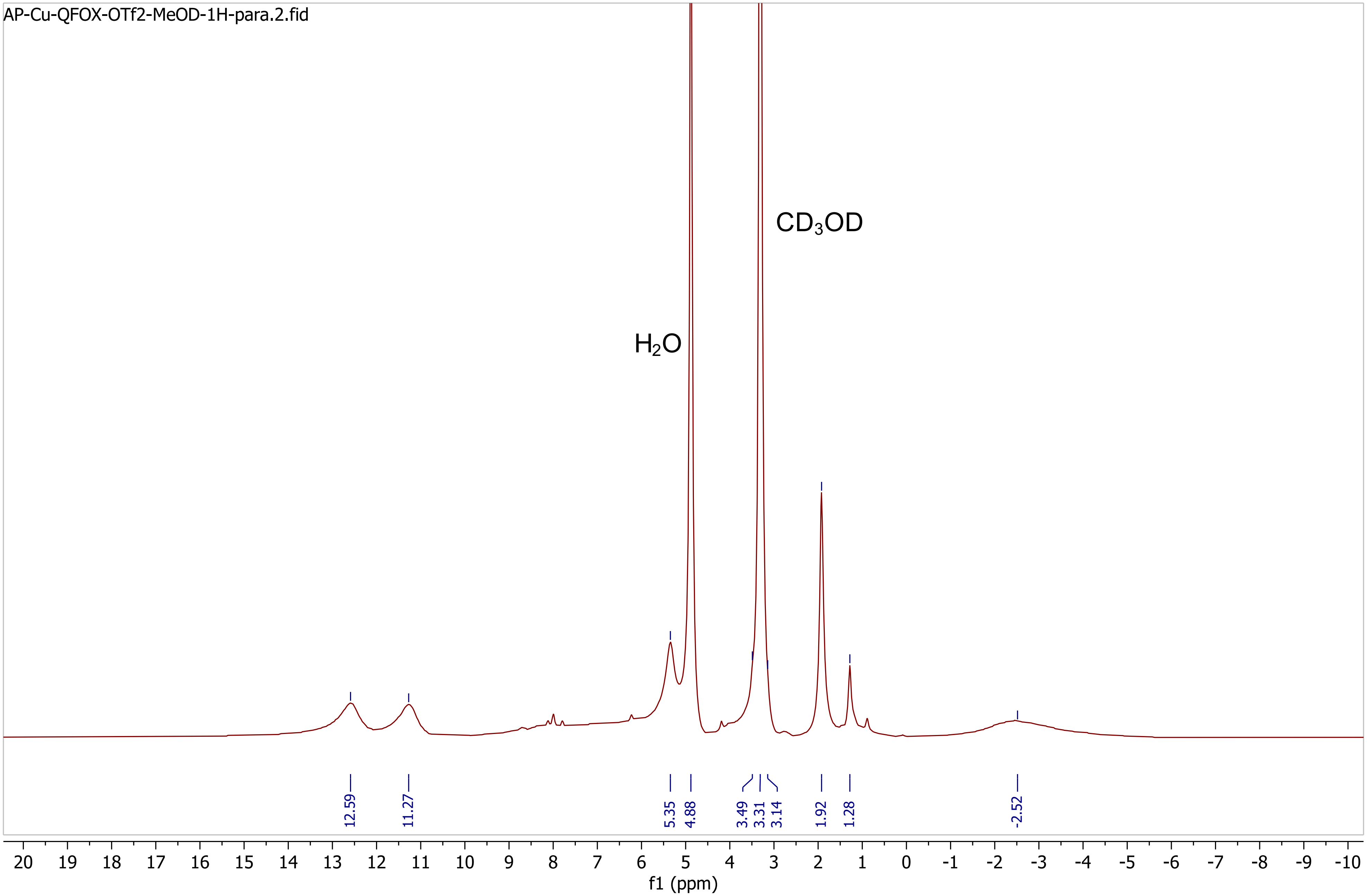

Screening Reactions - Dehydration of 1-phenylethanol catalyzed by bis-triflate complexes

In addition to the screens described in the text, a set of homogeneous reaction conditions were tested. MeCN, THF, butyronitrile (PrCN), and DMSO were tested as organic cosolvents to facilitate catalyst solvation. Moreover, in previous studies of the iron(II) FOX complex, high concentrations of **A** favored the formation of **E** over **S**. Thus, **A** is added to a lower concentration (0.17 M) in subsequent screenings. Reactions were first tested in toluene (bp. = 110.6 ^{o}C) along with MeCN or THF as cosolvents (Table S-1). Reactions were then also tested in ortho-dichlorobenzene (bp. = 180.1 ^{o}C) along with cosolvents with higher boiling point (Table S-2). In particular, **2e** was not soluble in PrCN at room temperature, so 2.5% MeCN was added into the reaction to further facilitate the solvation of the catalyst. This screening led to an identification of the reaction condition that led to nearly quantitative conversion of **A** into **S** (Table S-2, entry 2). This condition was then applied to a screening of dehydration activity of complex **C1-C5**.

Table S-1. Screening of homogenous reaction conditions with **2b** in 1-dram vials.[1]

Entry	cosolvent	Catalyst	Homogenous?	%A	%S	%E
1	5% MeCN	**2b**	Suspension	52	15	18
2	10% MeCN	**2b**	Yes	6	62	18
3	20% MeCN	**2b**	Yes	25	23	28
4	50% MeCN	**2b**	Yes	28	13	46
5	20% THF	**2b**	Suspension	72	9	9
6	50% THF	**2b**	Yes	97	2	2
7	100 % THF	**2b**	Yes	100	0	0
8	100 % 1,4-Dioxane	**2b**	Yes	100	0	0
9	100 % MeCN	**2b**	Yes	100	0	0

[1] Reaction conditions: 0.17 mmol **A** and 1.7 umol catalyst (1%) in 1 mL solvent (cosolvent + toluene) under N_2 sealed in 1-dram vial equipped with PTFE cap liner, heated at 120 ^{o}C for 18 hours. % = % added **1** converted. Reactions analyzed by gas chromatography (GC) using calibration curves with the isolated compound.

Table S-2. Screening of homogenous reaction conditions with **2e** in 1-dram vials.[1]

				% Composition		
**Entry**	**Cosolvent**	**Catalyst**	**Homogenous?**	**%A**	**%S**	**%E**
1	2.5% MeCN, 2.5% PrCN	**2e**	Yes	3	90	2
2	2.5% MeCN, 7.5% PrCN	**2e**	Yes	0	98	0
3	2.5% MeCN, 17.5% PrCN	**2e**	Yes	0	100	0
4	5% DMSO	**2e**	Yes	25	46	20
5	10% DMSO	**2e**	Yes	84	23	11
6	20% DMSO	**2e**	Yes	70	37	9
7	100 % THF	**2e**	Yes	100	0	0
8	100 % 1,4-Dioxane	**2e**	Yes	100	0	0
9	100 % MeCN	**2e**	Yes	100	0	0

[1] Reaction conditions: 0.17 mmol **A** and 1.7 umol catalyst (1%) in 1 mL solvent (cosolvent + ortho-dichlorobenzene) under N_2 sealed in 1-dram vial equipped with PTFE cap liner, heated at 120 ^{o}C for 18 hours. % Composition = % added **1** converted. Reactions analyzed by gas chromatography (GC) using calibration curves with the isolated compound.

Isolation and Structures of Dibromide Complexes.

Crystal structures of the dibromide complexes with Mn, Co, and Ni were determined by X-ray crystallography. These dihalide complexes were recrystallized from MeOH via slow evaporation (Scheme S-1). In addition, crystals of Ni dihalide complexes were also obtained from liquid-liquid diffusion involving MeCN and Et_2O (Scheme S-2). In the synthesis of these dibromide complexes, they formed as precipitates in MeCN, so it might be expected that the molecular formula of these compounds is best represented by [M(*meso*-^{Q}FOX)(NCMe)Br]Br. However, the results of elemental analysis suggested the removal of MeCN molecules upon vacuum drying, supporting the generic molecular formula [M(*meso*-^{Q}FOX)Br_2]. The structure of solvated derivative [Ni(^{Q}FOX)(NCMe)Br]Br (**1d'**) was determined.

Scheme S-1. The generic structures of [M(*meso*-^{Q}FOX)Br_2] (M = Mn, Co, Ni, **1a**, **1d**, **1d**).

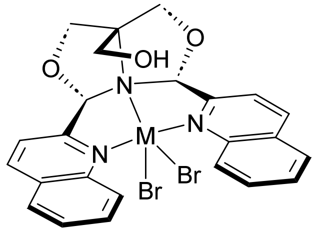

Scheme S-2. The structure of Ni(II) dihalide complex **1d'** recrystallized from Et_2O/MeCN.

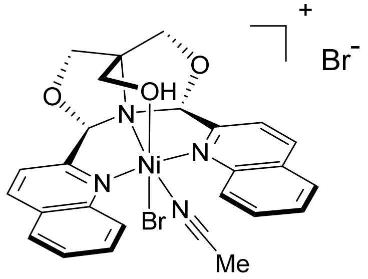

Isolation and Structures of Bis-triflate Complexes.

The structures of the metal bis-triflate complexes are presented in Scheme S-3, with their key structural features summarized in Table S-3. While Mn(II) can tolerate 2 triflate ions in the first coordination sphere, for Fe(II), Co(II), and Ni(II), one triflate ion was substituted by a linear MeCN molecule, and Cu(II) accommodated only one MeCN, likely due to the increase in effective nuclear charge and thus the decrease in ionic radius across the transition metal series.

Scheme S-3. The structures of M(^{Q}FOX) bis-triflate complexes (M = Mn, Fe, Co, Ni, Cu, **2a-e**) and [Ni(^{Q}FOX)(OTf)]$_2$(OTf)$_2$.

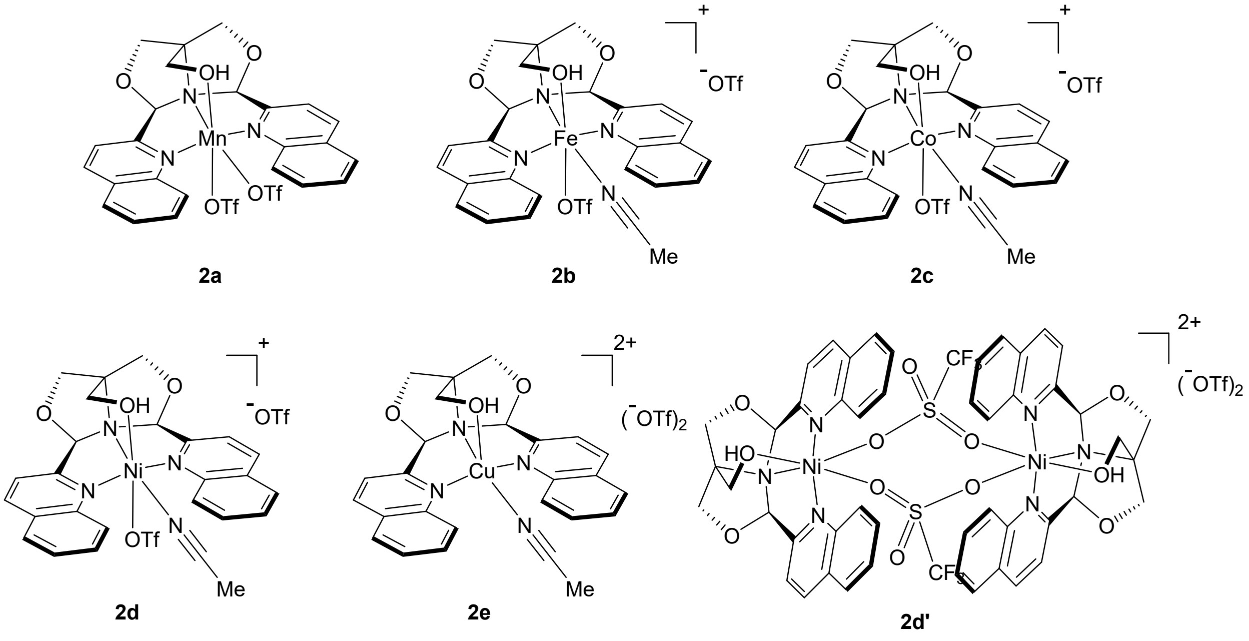

Table S-3. Some noteworthy features of the X-ray structures of bis-triflate M^{II}(^{Q}FOX) complexes

	Chemical formula	*meso*-^{Q}FOX binding mode	Other first coordination sphere ligand
**2a**	(^{Q}FOX)Mn(OTf)$_2$·MeCN	κ^4-ONNN	2 OTf$^-$
**2b**	[(^{Q}FOX)Fe(NCMe)(OTf)][OTf]·½Et$_2$O	κ^4-ONNN	1 OTf$^-$, 1 MeCN
**2c**	[(^{Q}FOX)Co(NCMe)(OTf)][OTf]	κ^4-ONNN	1 OTf$^-$, 1 MeCN
**2d**	[(^{Q}FOX)Ni(NCMe)(OTf)][OTf]·MeCN	κ^4-ONNN	1 OTf$^-$, 1 MeCN
**2e**	[(^{Q}FOX)Cu(NCMe)][OTf]$_2$·1.11MeCN	κ^4-ONNN	1 MeCN

REFERENCE NUMBER: jonap22

rac-^{Q}FOX

CRYSTAL STRUCTURE REPORT

C_{24} H_{21} N_3 O_3

Report prepared for:

C. Wood, A. Panda, Prof. W. Jones

June 25, 2023

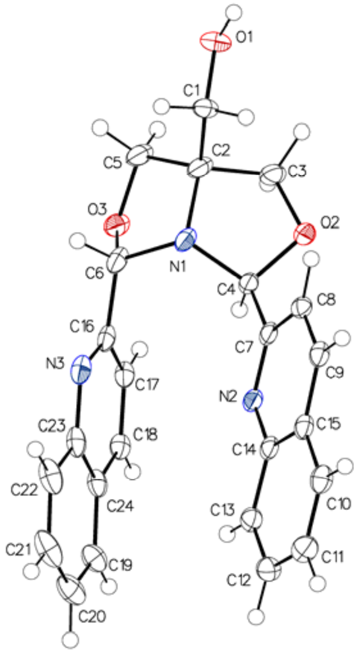

William W. Brennessel

X-ray Crystallographic Facility

Department of Chemistry, University of Rochester

120 Trustee Road

Rochester, NY 14627

Data collection

A crystal (0.236 x 0.041 x 0.033 mm^3) was placed onto a nylon loop and mounted on a Rigaku XtaLAB Synergy-S Dualflex diffractometer equipped with a HyPix-6000HE HPC area detector for data collection at 100.00(10) K. A preliminary set of cell constants and an orientation matrix were calculated from a small sampling of reflections.[1] A short pre-experiment was run, from which an optimal data collection strategy was determined. The full data collection was carried out using a PhotonJet (Cu) X-ray source with frame times of 2.00 and 8.00 seconds and a detector distance of 34.0 mm. Series of frames were collected in 0.50º steps in ω at different 2θ, κ, and ϕ settings. After the intensity data were corrected for absorption, the final cell constants were calculated from the xyz centroids of 10011 strong reflections from the actual data collection after integration.[1] See Table S-1 for additional crystal and refinement information.

Structure solution and refinement

The structure was solved using SHELXT[2] and refined using SHELXL.[3] The space group *C*2/*c* was determined based on systematic absences and intensity statistics. Most or all non-hydrogen atoms were assigned from the solution. Full-matrix least squares / difference Fourier cycles were performed which located any remaining non-hydrogen atoms. All non-hydrogen atoms were refined with anisotropic displacement parameters. The O-H hydrogen atoms were found from the difference Fourier map and then given riding models. All other hydrogen atoms were placed in ideal positions and refined as riding atoms with relative isotropic displacement parameters. The final full matrix least squares refinement converged to $R1 = 0.0370$ (F^2, $I > 2\sigma(I)$) and $wR2 = 0.0901$ (F^2, all data).

Structure description

The structure is the one suggested. The asymmetric unit contains one molecule in a general position. The $-CH_2OH$ terminus is modeled as disordered across crystallographic inversion centers; the hydrogen bonding is directional in each chain along [100], with an equal number in both directions throughout the crystal due to symmetry (see figure and Table S-7).

Structure manipulation and figure generation were performed using Olex2.[4] Unless noted otherwise all structural diagrams containing anisotropic displacement ellipsoids are drawn at the 50 % probability level.

Data collection, structure solution, and structure refinement were conducted at the X-ray Crystallographic Facility, B04 Hutchison Hall, Department of Chemistry, University of Rochester. The instrument was purchased with funding from NSF MRI program grant CHE-1725028. All publications arising from this report MUST either 1) include William W. Brennessel as a coauthor or 2) acknowledge William W. Brennessel and the X-ray Crystallographic Facility of the Department of Chemistry at the University of Rochester.

[1] *CrysAlisPro*, version 171.42.90a; Rigaku Corporation: Oxford, UK, 2023.

[2] Sheldrick, G. M. *SHELXT*, version 2018/2; *Acta. Crystallogr.* **2015**, *A71*, 3-8.

[3] Sheldrick, G. M. *SHELXL*, version 2019/2; *Acta. Crystallogr.* **2015**, *C71*, 3-8.

[4] Dolomanov, O. V.; Bourhis, L. J.; Gildea, R. J.; Howard, J. A. K.; Puschmann, H. *Olex2*, version 1.5; *J. Appl. Cryst.* **2009**, *42*, 339-341.

Some equations of interest:

$$R_{\mathrm{int}} = \Sigma\, |F_o^2 - \langle F_o^2 \rangle| \,/\, \Sigma|F_o^2|$$

$$R1 = \Sigma||F_o|-|F_c|| \,/\, \Sigma|F_o|$$

$$wR2 = [\Sigma[w(F_o^2-F_c^2)^2] \,/\, \Sigma\, [w(F_o^2)^2]]^{1/2}$$

where $w = 1 / [\sigma^2 (F_o^2) + (aP)^2 + bP]$ and

$$P = 1/3 \max(0, F_o^2) + 2/3\, F_c^2$$

$$\mathrm{GOF} = S = [\Sigma[w(F_o^2-F_c^2)^2] \,/\, (m-n)]^{1/2}$$

where m = number of reflections and n = number of parameters

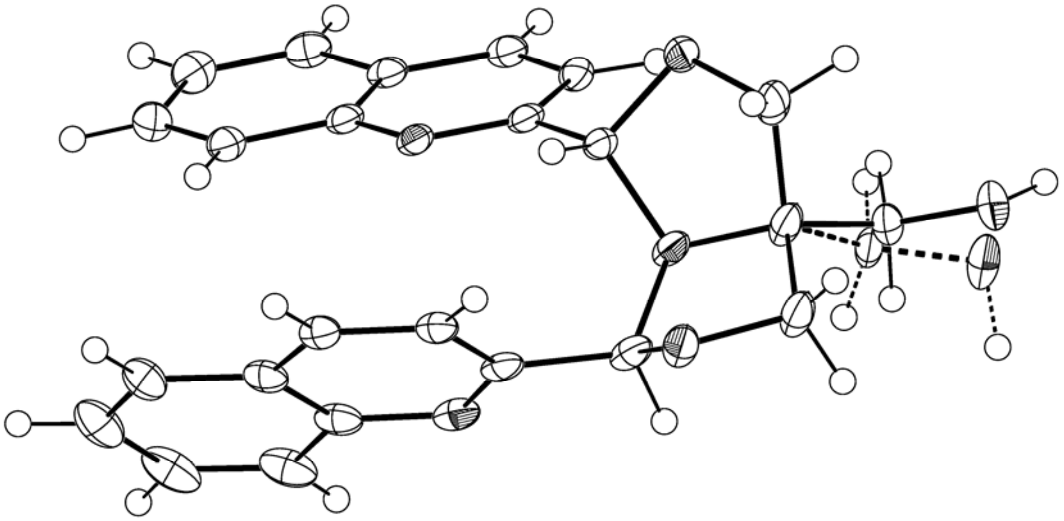

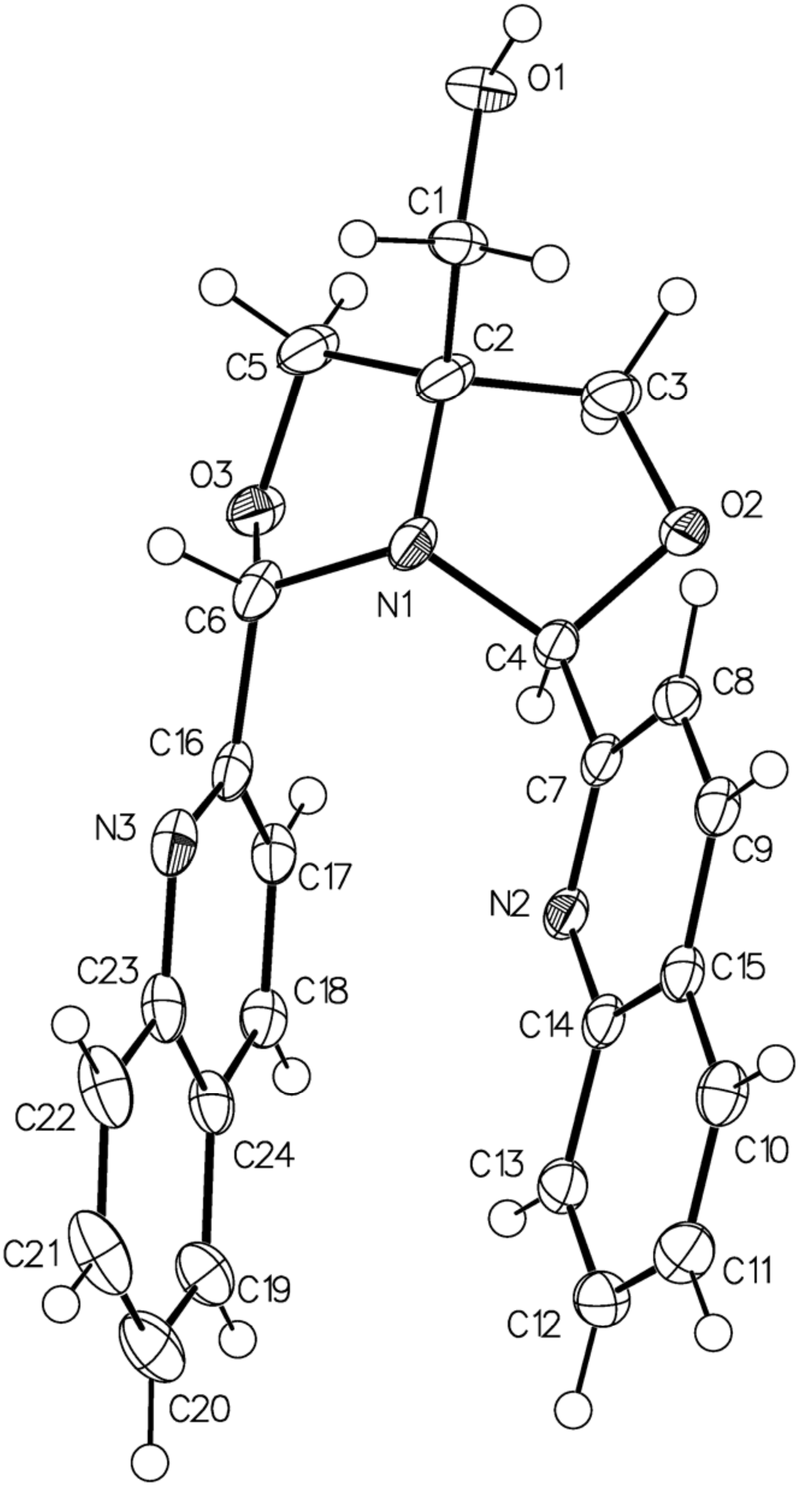
O1
C1
C2
C5
C3
O3
O2
N1
C6
C4
C8
C16
C7
C9
N3
C17
N2
C15
C23
C18
C14
C22
C24
C10
C13
C21
C19
C11
C12
C20

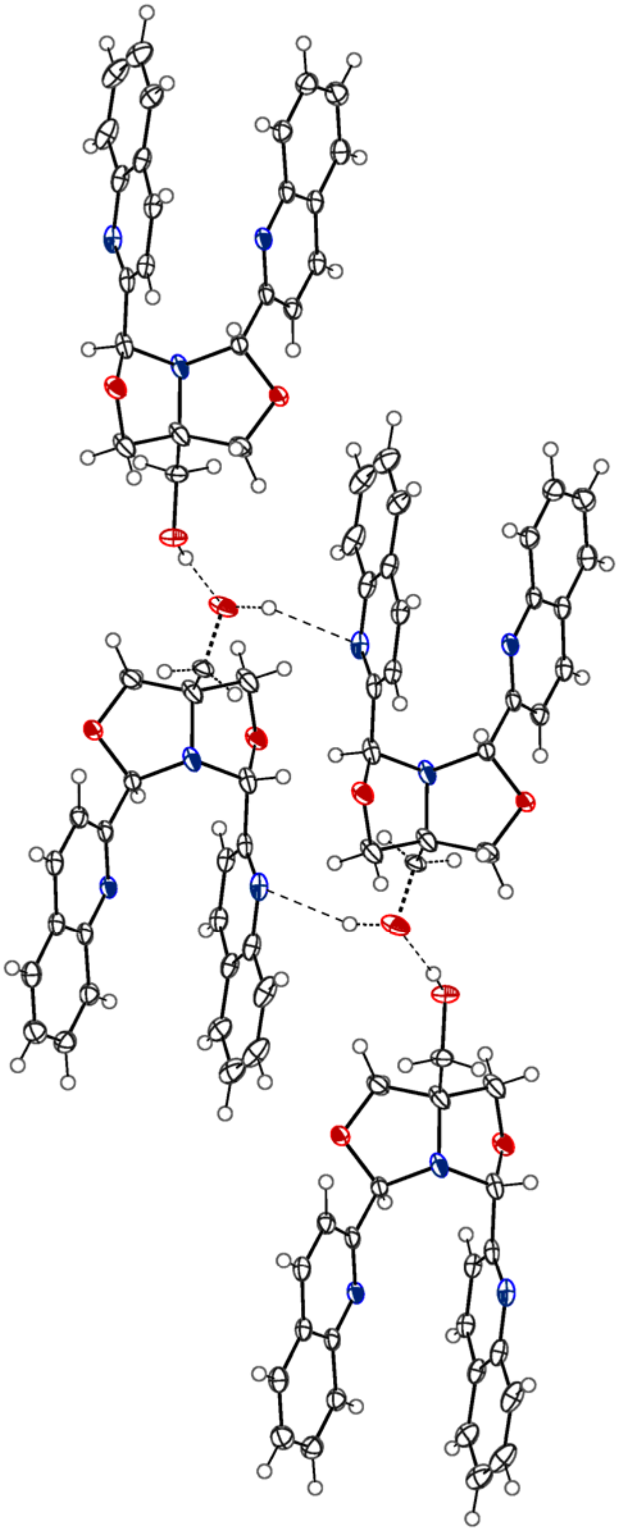

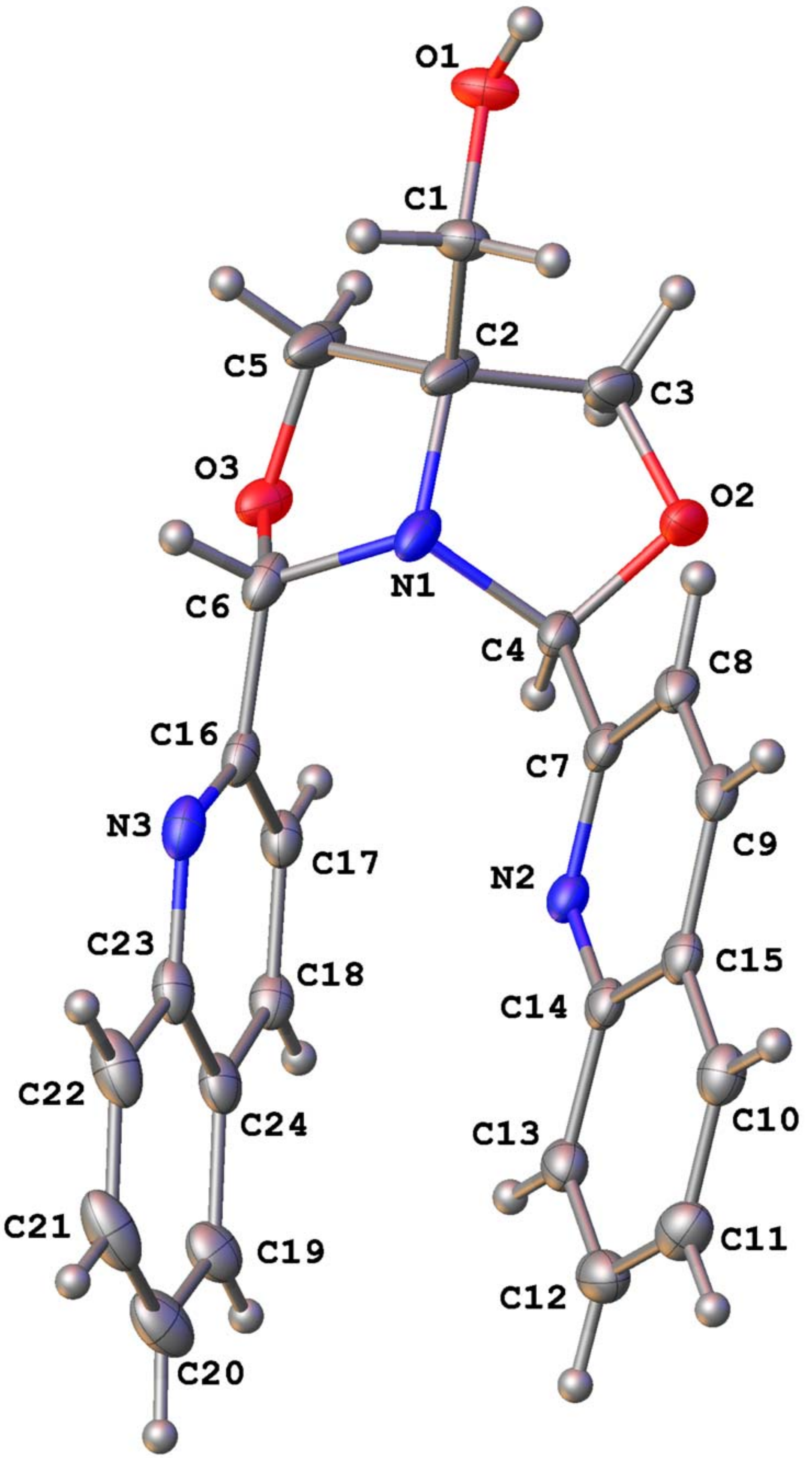
O1
C1
C2
C5
C3
O3
O2
N1
C6
C4
C8
C16
C7
N3
C17
C9
N2
C23
C18
C15
C14
C22
C24
C10
C13
C21
C19
C11
C12
C20

Table S-1. Crystal data and structure refinement for jonap22.

Identification code	jonap22	
Empirical formula	C24 H21 N3 O3	
Formula weight	399.44	
Temperature	100.00(10) K	
Wavelength	1.54184 Å	
Crystal system	monoclinic	
Space group	*C*2/*c*	
Unit cell dimensions	*a* = 20.2712(3) Å	α = 90°
	b = 5.99790(10) Å	β = 96.1670(10)°
	c = 30.7309(3) Å	γ = 90°
Volume	3714.78(9) Å^3	
Z	8	
Density (calculated)	1.428 Mg/m^3	
Absorption coefficient	0.776 mm^{-1}	
F(000)	1680	
Crystal color, morphology	colourless, needle	
Crystal size	0.236 x 0.041 x 0.033 mm^3	
Theta range for data collection	4.388 to 79.838°	
Index ranges	$-25 \le h \le 25$, $-7 \le k \le 7$, $-32 \le l \le 39$	
Reflections collected	22398	
Independent reflections	3964 [*R*(int) = 0.0384]	
Observed reflections	3332	
Completeness to theta = 74.504°	99.6%	
Absorption correction	Multi-scan	
Max. and min. transmission	1.00000 and 0.65163	
Refinement method	Full-matrix least-squares on F^2	
Data / restraints / parameters	3964 / 33 / 289	
Goodness-of-fit on F^2	1.047	
Final *R* indices [*I*>2sigma(*I*)]	*R*1 = 0.0370, *wR*2 = 0.0856	
R indices (all data)	*R*1 = 0.0461, *wR*2 = 0.0901	
Largest diff. peak and hole	0.209 and -0.235 e.Å^{-3}	

Table S-2. Atomic coordinates (x 10^4) and equivalent isotropic displacement parameters ($Å^2$x 10^3) for jonap22. U_{eq} is defined as one third of the trace of the orthogonalized U_{ij} tensor.

	x	y	z	U_{eq}
O1	243(1)	2301(3)	5016(1)	24(1)
O2	1083(1)	4545(2)	6178(1)	24(1)
O3	1852(1)	6936(2)	5212(1)	25(1)
N1	1848(1)	3948(2)	5688(1)	22(1)
N2	2705(1)	4549(2)	6672(1)	18(1)
N3	3388(1)	4944(2)	5753(1)	22(1)
C1	864(2)	1967(8)	5274(2)	21(1)
C2	1180(1)	4064(2)	5433(1)	29(1)
C3	744(1)	5053(3)	5758(1)	28(1)
C4	1765(1)	4846(2)	6131(1)	19(1)
C5	1278(1)	5665(3)	5053(1)	34(1)
C6	2275(1)	5285(2)	5425(1)	22(1)
C7	2171(1)	3547(2)	6485(1)	18(1)
C8	1972(1)	1365(2)	6596(1)	20(1)
C9	2344(1)	226(2)	6918(1)	20(1)
C10	3336(1)	201(2)	7476(1)	23(1)
C11	3890(1)	1266(2)	7666(1)	26(1)
C12	4065(1)	3393(2)	7518(1)	25(1)
C13	3674(1)	4442(2)	7189(1)	21(1)
C14	3091(1)	3406(2)	6992(1)	18(1)
C15	2923(1)	1238(2)	7132(1)	19(1)
C16	2874(1)	6292(2)	5683(1)	20(1)
C17	2864(1)	8506(2)	5846(1)	20(1)
C18	3397(1)	9249(2)	6114(1)	20(1)
C19	4510(1)	8404(2)	6515(1)	25(1)
C20	5023(1)	6935(2)	6599(1)	31(1)
C21	5009(1)	4840(2)	6391(1)	33(1)
C22	4473(1)	4226(2)	6108(1)	30(1)
C23	3926(1)	5682(2)	6022(1)	23(1)
C24	3947(1)	7824(2)	6221(1)	21(1)
O1'	559(1)	1446(3)	4931(1)	29(1)
C1'	1078(2)	1511(7)	5286(2)	19(1)

Table S-3. Bond lengths [Å] and angles [°] for jonap22.

O(1)-H(1)	0.8913	C(10)-C(15)	1.4180(17)
O(1)-C(1)	1.429(4)	C(11)-H(11)	0.9500
O(2)-C(3)	1.4282(14)	C(11)-C(12)	1.4129(19)
O(2)-C(4)	1.4158(14)	C(12)-H(12)	0.9500
O(3)-C(5)	1.4326(15)	C(12)-C(13)	1.3685(18)
O(3)-C(6)	1.4233(16)	C(13)-H(13)	0.9500
N(1)-C(2)	1.4912(17)	C(13)-C(14)	1.4117(17)
N(1)-C(4)	1.4899(14)	C(14)-C(15)	1.4223(17)
N(1)-C(6)	1.4815(15)	C(16)-C(17)	1.4199(16)
N(2)-C(7)	1.3147(15)	C(17)-H(17)	0.9500
N(2)-C(14)	1.3732(15)	C(17)-C(18)	1.3604(17)
N(3)-C(16)	1.3173(17)	C(18)-H(18)	0.9500
N(3)-C(23)	1.3693(17)	C(18)-C(24)	1.4160(18)
C(1)-H(1A)	0.9900	C(19)-H(19)	0.9500
C(1)-H(1B)	0.9900	C(19)-C(20)	1.3669(19)
C(1)-C(2)	1.471(5)	C(19)-C(24)	1.4188(18)
C(2)-C(3)	1.524(2)	C(20)-H(20)	0.9500
C(2)-C(5)	1.5423(17)	C(20)-C(21)	1.409(2)
C(2)-C(1')	1.604(5)	C(21)-H(21)	0.9500
C(3)-H(3A)	0.9900	C(21)-C(22)	1.367(2)
C(3)-H(3B)	0.9900	C(22)-H(22)	0.9500
C(4)-H(4)	1.0000	C(22)-C(23)	1.4148(19)
C(4)-C(7)	1.5103(17)	C(23)-C(24)	1.4227(17)
C(5)-H(5A)	0.9900	O(1')-H(1')	1.0069
C(5)-H(5B)	0.9900	O(1')-C(1')	1.432(4)
C(6)-H(6)	1.0000	C(1')-H(1'A)	0.9900
C(6)-C(16)	1.5034(17)	C(1')-H(1'B)	0.9900
C(7)-C(8)	1.4214(17)	C(1)-O(1)-H(1)	106.6
C(8)-H(8)	0.9500	C(4)-O(2)-C(3)	105.16(9)
C(8)-C(9)	1.3630(17)	C(6)-O(3)-C(5)	102.41(10)
C(9)-H(9)	0.9500	C(4)-N(1)-C(2)	106.07(10)
C(9)-C(15)	1.4185(17)	C(6)-N(1)-C(2)	103.86(9)
C(10)-H(10)	0.9500	C(6)-N(1)-C(4)	115.63(9)
C(10)-C(11)	1.3675(19)	C(7)-N(2)-C(14)	117.65(10)

C(16)-N(3)-C(23)	117.63(11)	O(3)-C(6)-C(16)	112.12(10)
O(1)-C(1)-H(1A)	109.0	N(1)-C(6)-H(6)	108.1
O(1)-C(1)-H(1B)	109.0	N(1)-C(6)-C(16)	114.58(9)
O(1)-C(1)-C(2)	113.0(3)	C(16)-C(6)-H(6)	108.1
H(1A)-C(1)-H(1B)	107.8	N(2)-C(7)-C(4)	116.03(10)
C(2)-C(1)-H(1A)	109.0	N(2)-C(7)-C(8)	123.93(11)
C(2)-C(1)-H(1B)	109.0	C(8)-C(7)-C(4)	120.04(11)
N(1)-C(2)-C(3)	103.46(9)	C(7)-C(8)-H(8)	120.5
N(1)-C(2)-C(5)	104.11(10)	C(9)-C(8)-C(7)	119.06(11)
N(1)-C(2)-C(1')	100.70(17)	C(9)-C(8)-H(8)	120.5
C(1)-C(2)-N(1)	118.23(18)	C(8)-C(9)-H(9)	120.4
C(1)-C(2)-C(3)	106.8(2)	C(8)-C(9)-C(15)	119.23(11)
C(1)-C(2)-C(5)	111.5(2)	C(15)-C(9)-H(9)	120.4
C(3)-C(2)-C(5)	112.68(13)	C(11)-C(10)-H(10)	119.8
C(3)-C(2)-C(1')	119.36(19)	C(11)-C(10)-C(15)	120.36(12)
C(5)-C(2)-C(1')	113.8(2)	C(15)-C(10)-H(10)	119.8
O(2)-C(3)-C(2)	104.55(11)	C(10)-C(11)-H(11)	119.8
O(2)-C(3)-H(3A)	110.8	C(10)-C(11)-C(12)	120.43(12)
O(2)-C(3)-H(3B)	110.8	C(12)-C(11)-H(11)	119.8
C(2)-C(3)-H(3A)	110.8	C(11)-C(12)-H(12)	119.7
C(2)-C(3)-H(3B)	110.8	C(13)-C(12)-C(11)	120.55(12)
H(3A)-C(3)-H(3B)	108.9	C(13)-C(12)-H(12)	119.7
O(2)-C(4)-N(1)	104.77(9)	C(12)-C(13)-H(13)	119.8
O(2)-C(4)-H(4)	110.5	C(12)-C(13)-C(14)	120.32(12)
O(2)-C(4)-C(7)	108.85(9)	C(14)-C(13)-H(13)	119.8
N(1)-C(4)-H(4)	110.5	N(2)-C(14)-C(13)	118.26(11)
N(1)-C(4)-C(7)	111.45(10)	N(2)-C(14)-C(15)	122.41(11)
C(7)-C(4)-H(4)	110.5	C(13)-C(14)-C(15)	119.32(11)
O(3)-C(5)-C(2)	103.77(10)	C(9)-C(15)-C(14)	117.68(11)
O(3)-C(5)-H(5A)	111.0	C(10)-C(15)-C(9)	123.33(11)
O(3)-C(5)-H(5B)	111.0	C(10)-C(15)-C(14)	118.99(11)
C(2)-C(5)-H(5A)	111.0	N(3)-C(16)-C(6)	114.72(11)
C(2)-C(5)-H(5B)	111.0	N(3)-C(16)-C(17)	123.89(12)
H(5A)-C(5)-H(5B)	109.0	C(17)-C(16)-C(6)	121.37(11)
O(3)-C(6)-N(1)	105.59(10)	C(16)-C(17)-H(17)	120.7
O(3)-C(6)-H(6)	108.1	C(18)-C(17)-C(16)	118.65(12)

C(18)-C(17)-H(17)	120.7	C(23)-C(22)-H(22)	119.7
C(17)-C(18)-H(18)	120.1	N(3)-C(23)-C(22)	118.21(12)
C(17)-C(18)-C(24)	119.83(11)	N(3)-C(23)-C(24)	122.54(12)
C(24)-C(18)-H(18)	120.1	C(22)-C(23)-C(24)	119.24(12)
C(20)-C(19)-H(19)	119.8	C(18)-C(24)-C(19)	124.01(12)
C(20)-C(19)-C(24)	120.40(13)	C(18)-C(24)-C(23)	117.23(12)
C(24)-C(19)-H(19)	119.8	C(19)-C(24)-C(23)	118.76(12)
C(19)-C(20)-H(20)	119.6	C(1')-O(1')-H(1')	105.7
C(19)-C(20)-C(21)	120.75(14)	C(2)-C(1')-H(1'A)	110.2
C(21)-C(20)-H(20)	119.6	C(2)-C(1')-H(1'B)	110.2
C(20)-C(21)-H(21)	119.9	O(1')-C(1')-C(2)	107.6(3)
C(22)-C(21)-C(20)	120.27(13)	O(1')-C(1')-H(1'A)	110.2
C(22)-C(21)-H(21)	119.9	O(1')-C(1')-H(1'B)	110.2
C(21)-C(22)-H(22)	119.7	H(1'A)-C(1')-H(1'B)	108.5
C(21)-C(22)-C(23)	120.54(13)		

Table S-4. Anisotropic displacement parameters ($Å^2$x 10^3) for jonap22. The anisotropic displacement factor exponent takes the form: $-2\pi^2[h^2 a^{*2}U_{11} + ... + 2 h k a^* b^* U_{12}]$

	U_{11}	U_{22}	U_{33}	U_{23}	U_{13}	U_{12}
O1	18(1)	23(1)	29(1)	-2(1)	-7(1)	-2(1)
O2	22(1)	36(1)	14(1)	4(1)	0(1)	-8(1)
O3	32(1)	26(1)	17(1)	3(1)	-1(1)	-13(1)
N1	33(1)	21(1)	12(1)	-2(1)	5(1)	-11(1)
N2	23(1)	19(1)	14(1)	-2(1)	5(1)	-4(1)
N3	32(1)	17(1)	19(1)	-1(1)	12(1)	-2(1)
C1	16(2)	25(2)	21(2)	-2(1)	-3(2)	-2(1)
C2	40(1)	32(1)	13(1)	1(1)	-1(1)	-20(1)
C3	28(1)	39(1)	17(1)	6(1)	-4(1)	-12(1)
C4	22(1)	22(1)	13(1)	-2(1)	4(1)	-7(1)
C5	45(1)	41(1)	14(1)	2(1)	-2(1)	-26(1)
C6	36(1)	18(1)	14(1)	-2(1)	8(1)	-6(1)
C7	23(1)	20(1)	12(1)	-4(1)	6(1)	-3(1)
C8	23(1)	20(1)	16(1)	-5(1)	5(1)	-6(1)
C9	27(1)	17(1)	18(1)	-3(1)	9(1)	-4(1)
C10	28(1)	20(1)	22(1)	0(1)	8(1)	2(1)
C11	27(1)	28(1)	23(1)	0(1)	2(1)	6(1)
C12	21(1)	29(1)	25(1)	-5(1)	4(1)	-1(1)
C13	23(1)	22(1)	20(1)	-3(1)	6(1)	-3(1)
C14	22(1)	19(1)	14(1)	-4(1)	7(1)	-1(1)
C15	23(1)	18(1)	16(1)	-3(1)	7(1)	0(1)
C16	29(1)	17(1)	15(1)	0(1)	10(1)	-4(1)
C17	24(1)	17(1)	20(1)	-1(1)	9(1)	-2(1)
C18	26(1)	14(1)	21(1)	-1(1)	9(1)	-3(1)
C19	24(1)	20(1)	32(1)	4(1)	6(1)	-6(1)
C20	24(1)	27(1)	42(1)	12(1)	6(1)	-5(1)
C21	24(1)	25(1)	53(1)	12(1)	16(1)	3(1)
C22	30(1)	19(1)	43(1)	3(1)	19(1)	2(1)
C23	27(1)	18(1)	25(1)	2(1)	14(1)	-2(1)
C24	23(1)	18(1)	24(1)	2(1)	11(1)	-3(1)
O1'	33(1)	26(1)	24(1)	0(1)	-11(1)	-7(1)
C1'	20(2)	22(2)	16(1)	-2(1)	-3(2)	-6(2)

Table S-5. Hydrogen coordinates (x 10^4) and isotropic displacement parameters ($Å^2$ x 10^3) for jonap22.

	x	y	z	U(eq)
H1	25	1010	5015	36
H1A	1168	1166	5096	26
H1B	794	1014	5528	26
H3A	697	6684	5717	34
H3B	297	4366	5723	34
H4	1884	6464	6149	23
H5A	1355	4828	4785	40
H5B	886	6643	4987	40
H6	2432	4293	5196	27
H8	1585	709	6447	24
H9	2217	-1228	6999	24
H10	3227	-1241	7574	27
H11	4158	571	7900	31
H12	4457	4101	7647	30
H13	3796	5873	7092	26
H17	2494	9449	5770	24
H18	3400	10722	6228	24
H19	4529	9821	6654	30
H20	5393	7331	6801	37
H21	5374	3849	6448	40
H22	4468	2812	5969	36
H1'	788	1313	4658	43
H1'A	1494	906	5191	23
H1'B	956	600	5534	23

Table S-6. Torsion angles [°] for jonap22.

O1-C1-C2-N1	-177.0(2)	C4-N1-C2-C1'	-124.0(2)
O1-C1-C2-C3	67.1(4)	C4-N1-C6-O3	-85.45(12)
O1-C1-C2-C5	-56.4(4)	C4-N1-C6-C16	38.42(15)
O2-C4-C7-N2	-138.64(10)	C4-C7-C8-C9	-179.66(10)
O2-C4-C7-C8	41.99(14)	C5-O3-C6-N1	-45.40(11)
O3-C6-C16-N3	-156.39(10)	C5-O3-C6-C16	-170.81(10)
O3-C6-C16-C17	25.39(15)	C5-C2-C3-O2	-135.04(11)
N1-C2-C3-O2	-23.22(13)	C5-C2-C1'-O1'	-51.9(3)
N1-C2-C5-O3	-22.36(15)	C6-O3-C5-C2	41.23(13)
N1-C2-C1'-O1'	-162.6(2)	C6-N1-C2-C1	119.9(3)
N1-C4-C7-N2	106.30(11)	C6-N1-C2-C3	-122.37(10)
N1-C4-C7-C8	-73.07(13)	C6-N1-C2-C5	-4.40(13)
N1-C6-C16-N3	83.28(13)	C6-N1-C2-C1'	113.7(2)
N1-C6-C16-C17	-94.94(13)	C6-N1-C4-O2	138.04(11)
N2-C7-C8-C9	1.02(17)	C6-N1-C4-C7	-104.41(12)
N2-C14-C15-C9	2.04(16)	C6-C16-C17-C18	173.54(10)
N2-C14-C15-C10	-177.72(10)	C7-N2-C14-C13	178.58(10)
N3-C16-C17-C18	-4.51(17)	C7-N2-C14-C15	-1.73(16)
N3-C23-C24-C18	-3.28(17)	C7-C8-C9-C15	-0.65(17)
N3-C23-C24-C19	176.46(11)	C8-C9-C15-C10	178.97(11)
C1-C2-C3-O2	102.2(2)	C8-C9-C15-C14	-0.78(16)
C1-C2-C5-O3	-150.89(19)	C10-C11-C12-C13	1.62(19)
C2-N1-C4-O2	23.54(12)	C11-C10-C15-C9	179.69(11)
C2-N1-C4-C7	141.09(10)	C11-C10-C15-C14	-0.55(17)
C2-N1-C6-O3	30.31(12)	C11-C12-C13-C14	-0.17(18)
C2-N1-C6-C16	154.18(11)	C12-C13-C14-N2	178.09(11)
C3-O2-C4-N1	-39.19(12)	C12-C13-C14-C15	-1.62(17)
C3-O2-C4-C7	-158.51(10)	C13-C14-C15-C9	-178.26(10)
C3-C2-C5-O3	89.06(14)	C13-C14-C15-C10	1.97(16)
C3-C2-C1'-O1'	85.1(3)	C14-N2-C7-C4	-179.17(10)
C4-O2-C3-C2	39.13(13)	C14-N2-C7-C8	0.17(16)
C4-N1-C2-C1	-117.8(3)	C15-C10-C11-C12	-1.24(18)
C4-N1-C2-C3	-0.04(12)	C16-N3-C23-C22	178.29(11)
C4-N1-C2-C5	117.93(11)	C16-N3-C23-C24	-0.42(17)

C16-C17-C18-C24	0.44(17)	C21-C22-C23-C24	1.95(18)
C17-C18-C24-C19	-176.59(11)	C22-C23-C24-C18	178.02(11)
C17-C18-C24-C23	3.13(16)	C22-C23-C24-C19	-2.24(17)
C19-C20-C21-C22	-1.5(2)	C23-N3-C16-C6	-173.76(10)
C20-C19-C24-C18	-179.57(12)	C23-N3-C16-C17	4.41(17)
C20-C19-C24-C23	0.71(18)	C24-C19-C20-C21	1.2(2)
C20-C21-C22-C23	-0.1(2)	C1'-C2-C3-O2	87.5(2)
C21-C22-C23-N3	-176.80(12)	C1'-C2-C5-O3	-131.02(19)

Table S-7. Hydrogen bonds and close contacts for jonap22 [Å and °].

D-H...A	d(D-H)	d(H...A)	d(D...A)	<(DHA)
O1-H1...O1'#1	0.89	1.91	2.789(3)	169.2
O1'-H1'...N3#2	1.01	2.32	3.262(3)	154.4

Symmetry transformations used to generate equivalent atoms:

#1 -x,-y,-z+1 #2 -x+1/2,-y+1/2,-z+1

REFERENCE NUMBER: jonap24

meso-^{Q}FOX

CRYSTAL STRUCTURE REPORT

$C_{24} H_{21} N_3 O_3$

Report prepared for:

R. Zhang, A. Panda, Prof. W. Jones

September 18, 2023

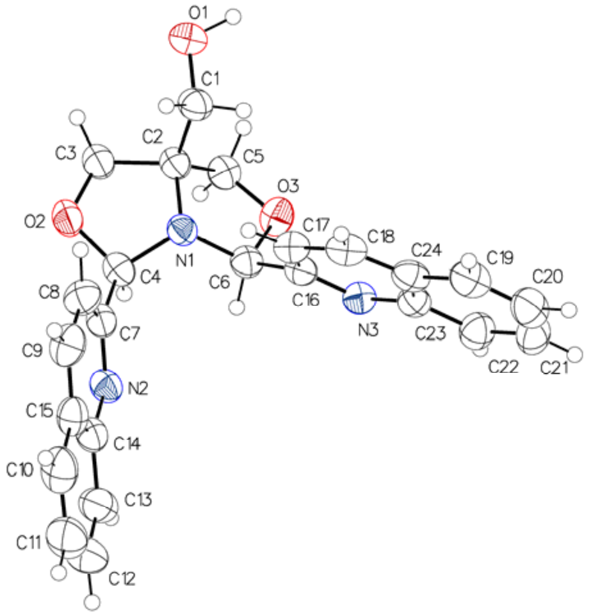

William W. Brennessel

X-ray Crystallographic Facility

Department of Chemistry, University of Rochester

120 Trustee Road

Rochester, NY 14627

Data collection

A crystal (0.158 x 0.063 x 0.015 mm^3) was placed onto a nylon loop and mounted on a Rigaku XtaLAB Synergy-S Dualflex diffractometer equipped with a HyPix-6000HE HPC area detector for data collection at 222.99(10) K. A preliminary set of cell constants and an orientation matrix were calculated from a small sampling of reflections.[1] A short pre-experiment was run, from which an optimal data collection strategy was determined. The full data collection was carried out using a PhotonJet (Cu) X-ray source with frame times of 2.71 and 10.85 seconds and a detector distance of 34.0 mm. Series of frames were collected in 0.50º steps in ω at different 2θ, κ, and ϕ settings. After the intensity data were corrected for absorption, the final cell constants were calculated from the xyz centroids of 26951 strong reflections from the actual data collection after integration.[1] See Table S-1 for additional crystal and refinement information.

Structure solution and refinement

The structure was solved using SHELXT[2] and refined using SHELXL.[3] The space group $P2_1$ was determined based on systematic absences and intensity statistics. Most or all non-hydrogen atoms were assigned from the solution. Full-matrix least squares / difference Fourier cycles were performed which located any remaining non-hydrogen atoms. All non-hydrogen atoms were refined with anisotropic displacement parameters. The O-H hydrogen atoms were found from the difference Fourier map and refined freely. All other hydrogen atoms were placed in ideal positions and refined as riding atoms with relative isotropic displacement parameters. The final full matrix least squares refinement converged to $R1 = 0.0314$ (F^2, $I > 2\sigma(I)$) and $wR2 = 0.0826$ (F^2, all data).

Structure description

The structure is the one suggested. The asymmetric unit contains two molecules in general positions. Molecules are linked in one dimension along [010] via O-H…N hydrogen bonding (see figure and Table S-7).

Structure manipulation and figure generation were performed using Olex2.[4] Unless noted otherwise all structural diagrams containing anisotropic displacement ellipsoids are drawn at the 50 % probability level.

Data collection, structure solution, and structure refinement were conducted at the X-ray Crystallographic Facility, B04 Hutchison Hall, Department of Chemistry, University of Rochester. The instrument was purchased with funding from NSF MRI program grant CHE-1725028. All publications arising from this report MUST either 1) include William W. Brennessel as a coauthor or 2) acknowledge William W. Brennessel and the X-ray Crystallographic Facility of the Department of Chemistry at the University of Rochester.

[1] *CrysAlisPro*, version 171.42.101a; Rigaku Corporation: Oxford, UK, 2023.

[2] Sheldrick, G. M. *SHELXT*, version 2018/2; *Acta. Crystallogr.* **2015**, *A71*, 3-8.

[3] Sheldrick, G. M. *SHELXL*, version 2019/2; *Acta. Crystallogr.* **2015**, *C71*, 3-8.

[4] Dolomanov, O. V.; Bourhis, L. J.; Gildea, R. J.; Howard, J. A. K.; Puschmann, H. *Olex2*, version 1.5; *J. Appl. Cryst.* **2009**, *42*, 339-341.

Some equations of interest:

$$R_{int} = \Sigma |F_o^2 - \langle F_o^2 \rangle| / \Sigma|F_o^2|$$

$$R1 = \Sigma||F_o|-|F_c|| / \Sigma|F_o|$$

$$wR2 = [\Sigma[w(F_o^2-F_c^2)^2] / \Sigma [w(F_o^2)^2]]^{1/2}$$

$$\text{where } w = 1 / [\sigma^2 (F_o^2) + (aP)^2 + bP] \text{ and}$$

$$P = 1/3 \max (0, F_o^2) + 2/3 F_c^2$$

$$\text{GOF} = S = [\Sigma[w(F_o^2-F_c^2)^2] / (m-n)]^{1/2}$$

where m = number of reflections and n = number of parameters

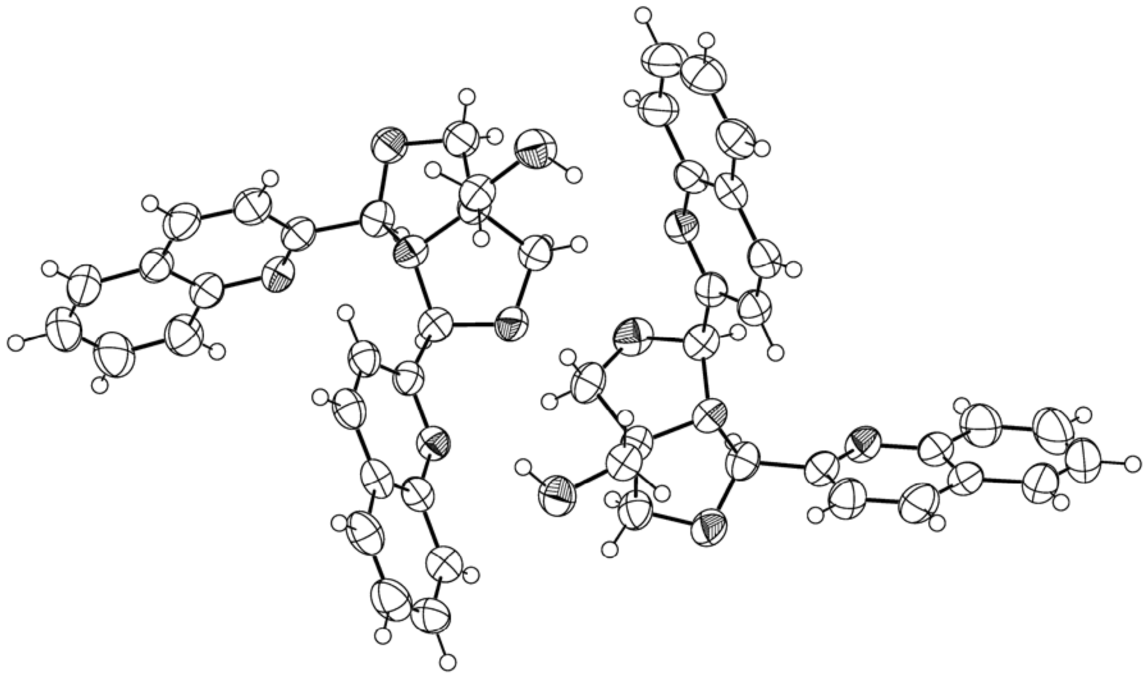

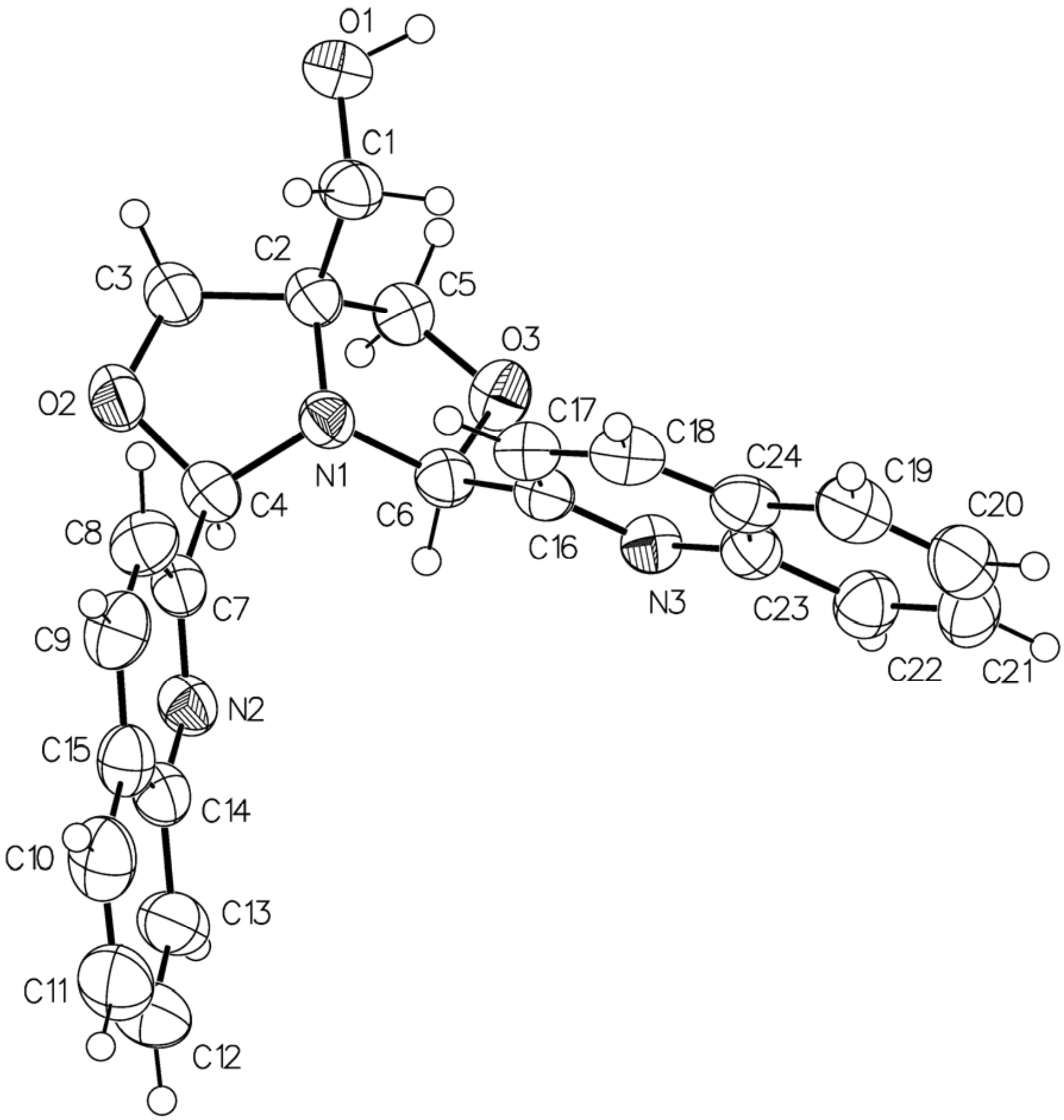
O1
C1
C2
C3
C5
O3
O2
N1
C17
C18
C24
C19
C20
C4
C6
C8
C16
N3
C23
C7
C9
C22
C21
N2
C15
C14
C10
C13
C11
C12

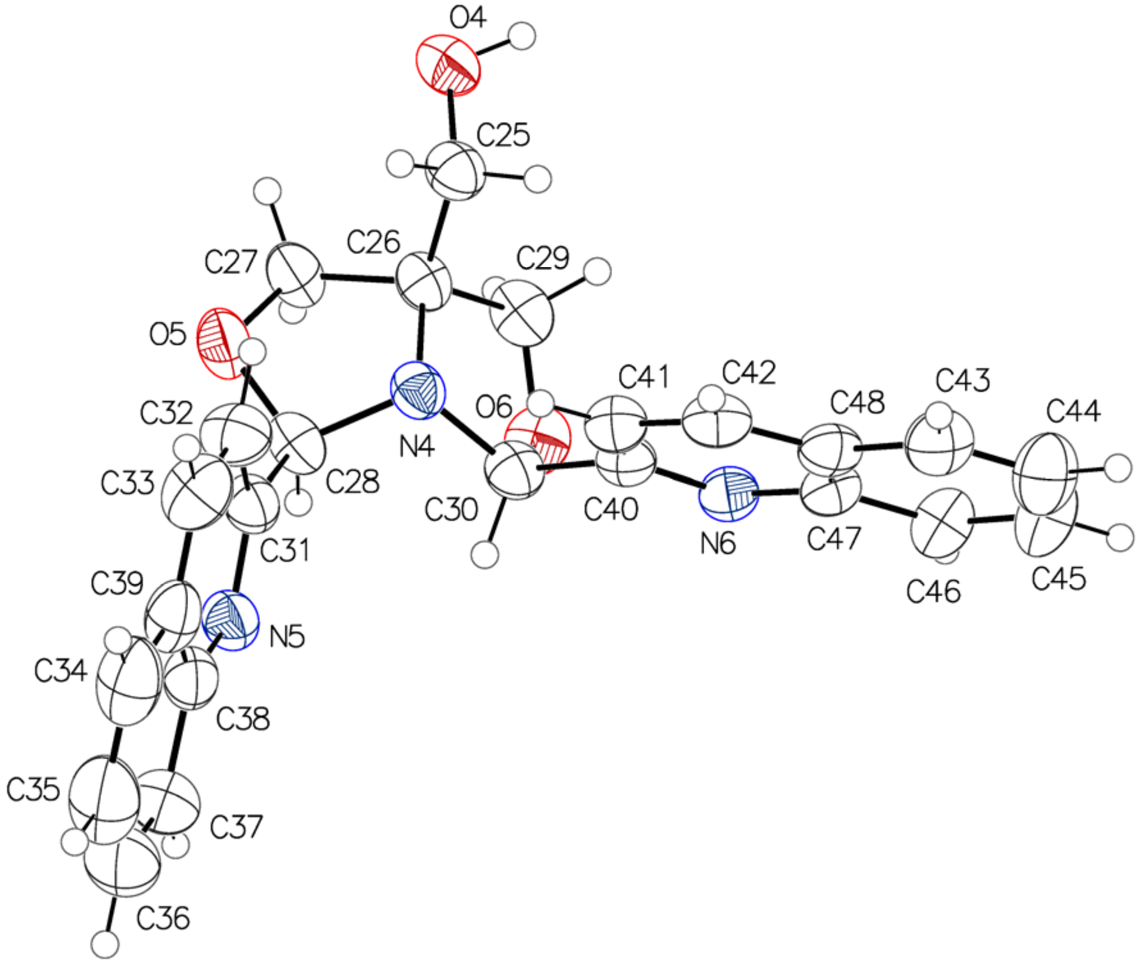

O4
C25
C27
C26
C29
O5
C41
C42
C43
O6
C48
C44
C32
N4
C28
C33
C30
C40
N6
C47
C31
C46
C45
C39
N5
C34
C38
C35
C37
C36

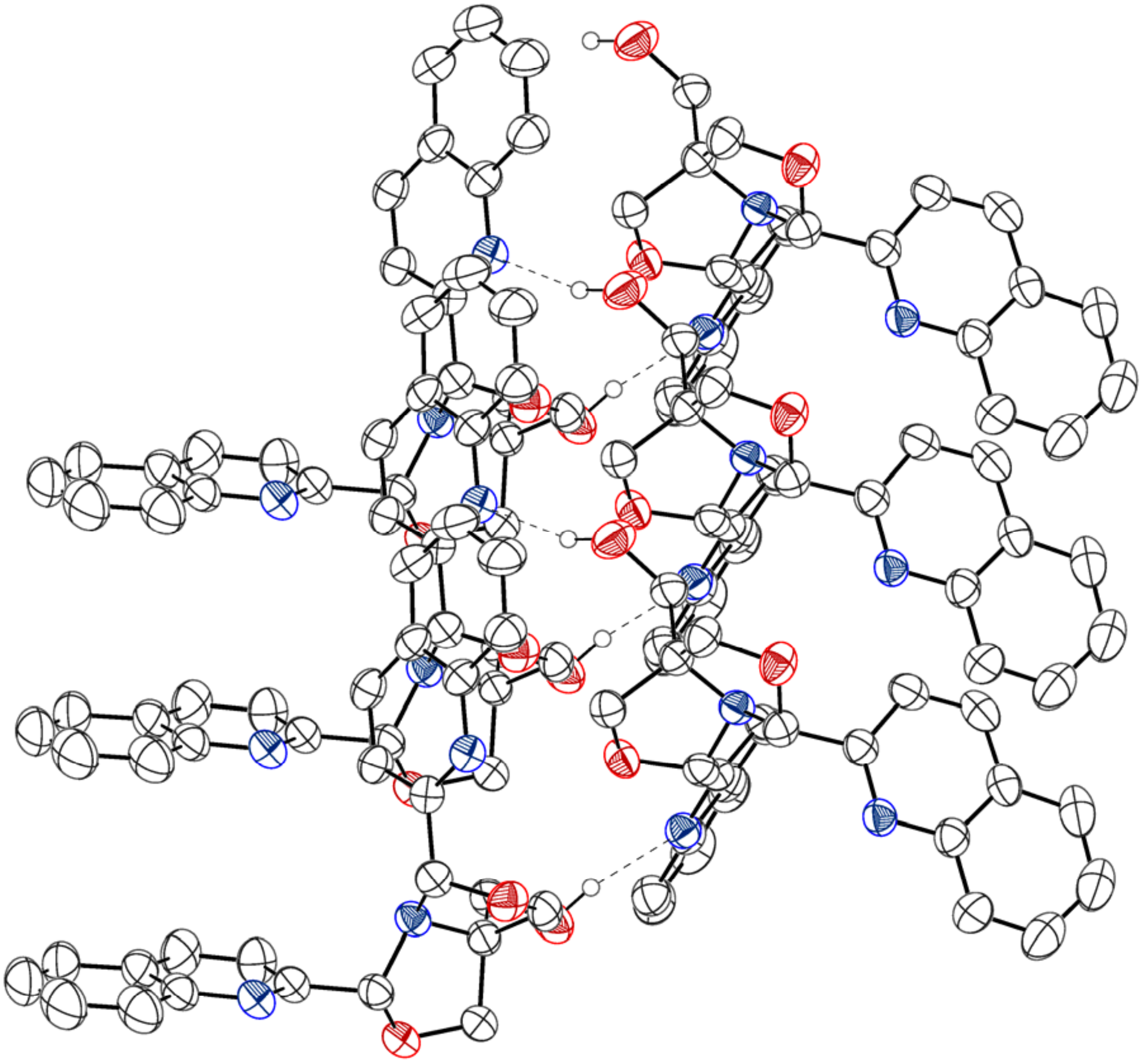

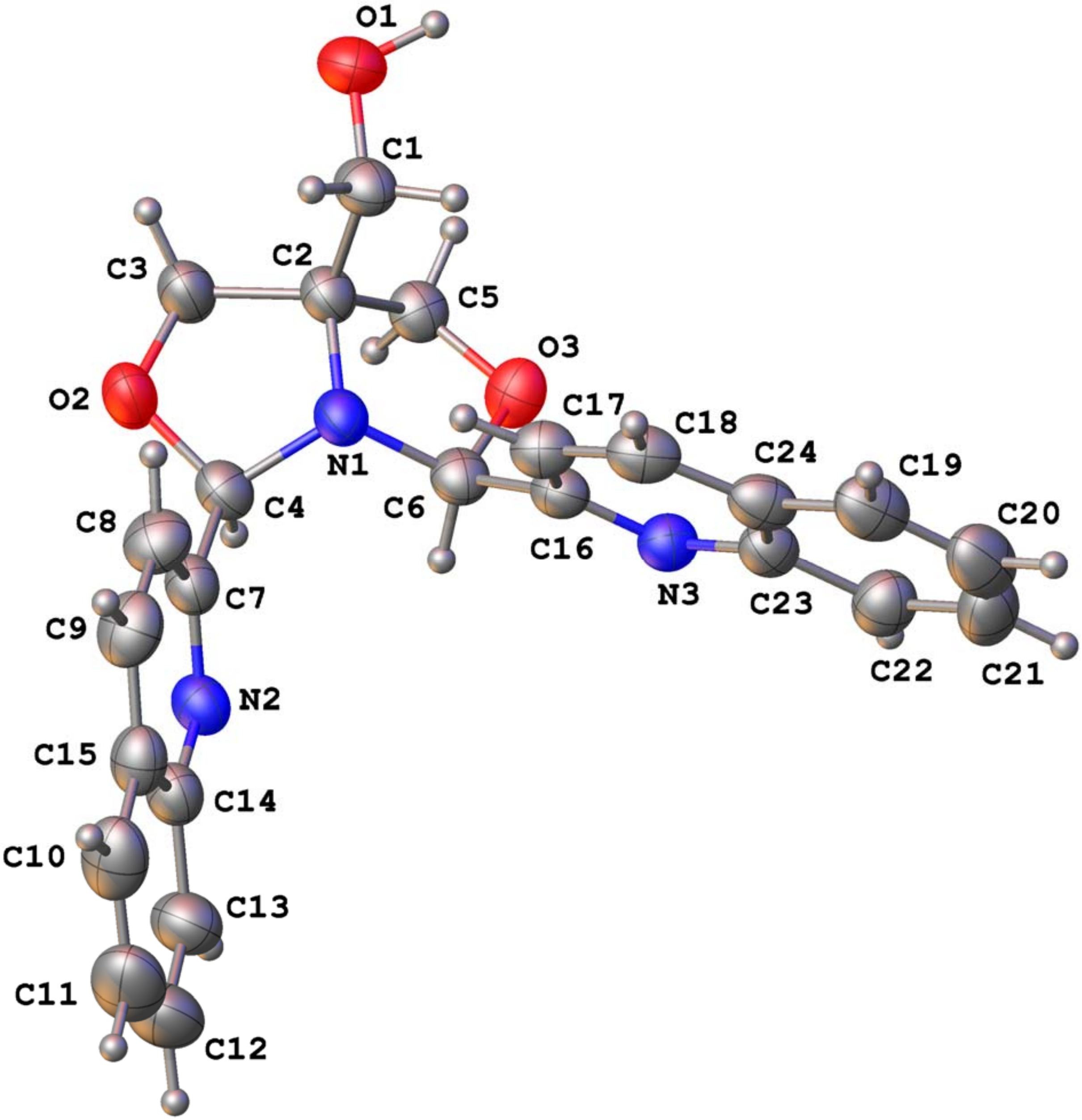
O1
C1
C2
C3
C5
O3
O2
N1
C17
C18
C24
C19
C20
C4
C6
C8
C16
N3
C23
C7
C9
C22
C21
N2
C15
C14
C10
C13
C11
C12

Table S-1. Crystal data and structure refinement for jonap24.

Identification code	jonap24	
Empirical formula	C24 H21 N3 O3	
Formula weight	399.44	
Temperature	222.99(10) K	
Wavelength	1.54184 Å	
Crystal system	monoclinic	
Space group	$P2_1$	
Unit cell dimensions	a = 11.92869(7) Å	α = 90°
	b = 6.22289(4) Å	β = 98.5135(6)°
	c = 26.83826(15) Å	γ = 90°
Volume	1970.28(2) $Å^3$	
Z	4	
Density (calculated)	1.347 Mg/m^3	
Absorption coefficient	0.731 mm^{-1}	
$F(000)$	840	
Crystal color, morphology	colourless, needle	
Crystal size	0.158 x 0.063 x 0.015 mm^3	
Theta range for data collection	3.330 to 80.404°	
Index ranges	$-15 \le h \le 15$, $-7 \le k \le 7$, $-34 \le l \le 33$	
Reflections collected	49230	
Independent reflections	8282 [R(int) = 0.0314]	
Observed reflections	7597	
Completeness to theta = 74.504°	100.0%	
Absorption correction	Multi-scan	
Max. and min. transmission	1.00000 and 0.83401	
Refinement method	Full-matrix least-squares on F^2	
Data / restraints / parameters	8282 / 1 / 549	
Goodness-of-fit on F^2	1.057	
Final R indices [I>2sigma(I)]	$R1$ = 0.0314, $wR2$ = 0.0804	
R indices (all data)	$R1$ = 0.0351, $wR2$ = 0.0826	
Absolute structure parameter	-0.17(6)	
Largest diff. peak and hole	0.256 and -0.139 e.$Å^{-3}$	

Table S-2. Atomic coordinates (x 10^4) and equivalent isotropic displacement parameters ($Å^2$x 10^3) for jonap24. U_{eq} is defined as one third of the trace of the orthogonalized U_{ij} tensor.

	x	y	z	U_{eq}
O1	4245(1)	10855(3)	1505(1)	61(1)
O2	2178(1)	6237(3)	698(1)	52(1)
O3	2408(1)	7043(3)	2252(1)	46(1)
N1	1632(1)	7530(3)	1413(1)	39(1)
N2	-434(1)	4245(3)	846(1)	42(1)
N3	391(1)	8061(3)	2580(1)	41(1)
C1	3080(2)	10344(4)	1468(1)	46(1)
C2	2870(2)	7938(3)	1467(1)	40(1)
C3	3223(2)	6812(4)	1006(1)	50(1)
C4	1407(2)	5810(4)	1033(1)	43(1)
C5	3312(2)	6854(4)	1966(1)	42(1)
C6	1364(2)	6994(3)	1911(1)	40(1)
C7	209(2)	5876(3)	762(1)	42(1)
C8	-160(2)	7640(4)	451(1)	52(1)
C9	-1249(2)	7665(4)	217(1)	55(1)
C10	-3143(2)	5878(6)	74(1)	66(1)
C11	-3814(2)	4210(6)	178(1)	74(1)
C12	-3365(2)	2548(6)	498(1)	71(1)
C13	-2253(2)	2558(5)	708(1)	58(1)
C14	-1539(2)	4270(4)	614(1)	46(1)
C15	-1988(2)	5970(4)	294(1)	48(1)
C16	591(2)	8563(3)	2123(1)	38(1)
C17	186(2)	10456(3)	1871(1)	43(1)
C18	-398(2)	11910(4)	2115(1)	48(1)
C19	-1168(2)	12919(4)	2894(1)	57(1)
C20	-1368(2)	12352(5)	3364(1)	65(1)
C21	-1003(2)	10373(5)	3576(1)	61(1)
C22	-427(2)	8957(5)	3317(1)	53(1)
C23	-205(2)	9493(4)	2826(1)	42(1)
C24	-594(2)	11482(4)	2612(1)	45(1)
O4	1593(1)	5161(3)	3350(1)	60(1)

O5	3304(1)	361(3)	4080(1)	54(1)
O6	3834(1)	722(3)	2702(1)	52(1)
N4	4263(1)	1998(3)	3515(1)	40(1)
N5	6055(2)	-1314(3)	4276(1)	47(1)
N6	5451(1)	3354(3)	2386(1)	41(1)
C25	2773(2)	4781(4)	3457(1)	46(1)
C26	3044(2)	2466(3)	3352(1)	40(1)
C27	2454(2)	890(4)	3666(1)	50(1)
C28	4305(2)	159(4)	3865(1)	45(1)
C29	2871(2)	1960(4)	2784(1)	49(1)
C30	4737(2)	1489(3)	3060(1)	43(1)
C31	5328(2)	276(4)	4266(1)	43(1)
C32	5468(2)	2052(4)	4595(1)	56(1)
C33	6411(2)	2145(5)	4946(1)	62(1)
C34	8199(2)	447(7)	5341(1)	77(1)
C35	8933(2)	-1212(9)	5364(1)	93(1)
C36	8726(3)	-2912(8)	5022(1)	93(1)
C37	7785(2)	-2938(6)	4663(1)	73(1)
C38	7008(2)	-1231(4)	4634(1)	51(1)
C39	7214(2)	489(5)	4978(1)	55(1)
C40	5342(2)	3388(3)	2869(1)	40(1)
C41	5816(2)	5006(4)	3207(1)	43(1)
C42	6389(2)	6652(4)	3028(1)	45(1)
C43	7013(2)	8450(4)	2293(1)	56(1)
C44	7089(2)	8431(5)	1793(1)	69(1)
C45	6649(2)	6706(6)	1490(1)	70(1)
C46	6122(2)	5027(5)	1684(1)	56(1)
C47	6019(2)	5013(4)	2204(1)	42(1)
C48	6484(2)	6742(4)	2510(1)	44(1)

Table S-3. Bond lengths [Å] and angles [°] for jonap24.

O(1)-C(1)	1.414(3)	C(11)-H(11)	0.9400
O(1)-H(1)	0.99(5)	C(11)-C(12)	1.400(5)
O(2)-C(3)	1.435(2)	C(12)-H(12)	0.9400
O(2)-C(4)	1.405(3)	C(12)-C(13)	1.362(4)
O(3)-C(5)	1.418(2)	C(13)-H(13)	0.9400
O(3)-C(6)	1.433(2)	C(13)-C(14)	1.410(3)
N(1)-C(2)	1.484(3)	C(14)-C(15)	1.418(3)
N(1)-C(4)	1.474(3)	C(16)-C(17)	1.408(3)
N(1)-C(6)	1.458(2)	C(17)-H(17)	0.9400
N(2)-C(7)	1.311(3)	C(17)-C(18)	1.366(3)
N(2)-C(14)	1.372(3)	C(18)-H(18)	0.9400
N(3)-C(16)	1.322(2)	C(18)-C(24)	1.415(3)
N(3)-C(23)	1.368(3)	C(19)-H(19)	0.9400
C(1)-H(1A)	0.9800	C(19)-C(20)	1.365(4)
C(1)-H(1B)	0.9800	C(19)-C(24)	1.413(3)
C(1)-C(2)	1.518(3)	C(20)-H(20)	0.9400
C(2)-C(3)	1.535(3)	C(20)-C(21)	1.399(4)
C(2)-C(5)	1.523(3)	C(21)-H(21)	0.9400
C(3)-H(3A)	0.9800	C(21)-C(22)	1.370(4)
C(3)-H(3B)	0.9800	C(22)-H(22)	0.9400
C(4)-H(4)	0.9900	C(22)-C(23)	1.421(3)
C(4)-C(7)	1.506(3)	C(23)-C(24)	1.413(3)
C(5)-H(5A)	0.9800	O(4)-C(25)	1.415(3)
C(5)-H(5B)	0.9800	O(4)-H(4A)	0.91(4)
C(6)-H(6)	0.9900	O(5)-C(27)	1.427(2)
C(6)-C(16)	1.512(3)	O(5)-C(28)	1.407(3)
C(7)-C(8)	1.409(3)	O(6)-C(29)	1.427(3)
C(8)-H(8)	0.9400	O(6)-C(30)	1.417(2)
C(8)-C(9)	1.358(3)	N(4)-C(26)	1.484(3)
C(9)-H(9)	0.9400	N(4)-C(28)	1.477(3)
C(9)-C(15)	1.408(4)	N(4)-C(30)	1.454(3)
C(10)-H(10)	0.9400	N(5)-C(31)	1.313(3)
C(10)-C(11)	1.364(5)	N(5)-C(38)	1.376(3)
C(10)-C(15)	1.417(3)	N(6)-C(40)	1.321(2)

N(6)-C(47)	1.365(3)	C(43)-C(48)	1.405(3)
C(25)-H(25A)	0.9800	C(44)-H(44)	0.9400
C(25)-H(25B)	0.9800	C(44)-C(45)	1.400(4)
C(25)-C(26)	1.512(3)	C(45)-H(45)	0.9400
C(26)-C(27)	1.532(3)	C(45)-C(46)	1.362(4)
C(26)-C(29)	1.538(3)	C(46)-H(46)	0.9400
C(27)-H(27A)	0.9800	C(46)-C(47)	1.418(3)
C(27)-H(27B)	0.9800	C(47)-C(48)	1.415(3)
C(28)-H(28)	0.9900	C(1)-O(1)-H(1)	113(3)
C(28)-C(31)	1.505(3)	C(4)-O(2)-C(3)	105.80(14)
C(29)-H(29A)	0.9800	C(5)-O(3)-C(6)	108.07(14)
C(29)-H(29B)	0.9800	C(4)-N(1)-C(2)	105.58(15)
C(30)-H(30)	0.9900	C(6)-N(1)-C(2)	107.40(15)
C(30)-C(40)	1.513(3)	C(6)-N(1)-C(4)	115.15(16)
C(31)-C(32)	1.408(3)	C(7)-N(2)-C(14)	117.41(18)
C(32)-H(32)	0.9400	C(16)-N(3)-C(23)	118.15(17)
C(32)-C(33)	1.359(3)	O(1)-C(1)-H(1A)	109.1
C(33)-H(33)	0.9400	O(1)-C(1)-H(1B)	109.1
C(33)-C(39)	1.401(4)	O(1)-C(1)-C(2)	112.45(19)
C(34)-H(34)	0.9400	H(1A)-C(1)-H(1B)	107.8
C(34)-C(35)	1.349(6)	C(2)-C(1)-H(1A)	109.1
C(34)-C(39)	1.412(3)	C(2)-C(1)-H(1B)	109.1
C(35)-H(35)	0.9400	N(1)-C(2)-C(1)	109.31(17)
C(35)-C(36)	1.398(6)	N(1)-C(2)-C(3)	103.32(15)
C(36)-H(36)	0.9400	N(1)-C(2)-C(5)	102.72(16)
C(36)-C(37)	1.367(4)	C(1)-C(2)-C(3)	112.79(18)
C(37)-H(37)	0.9400	C(1)-C(2)-C(5)	113.55(17)
C(37)-C(38)	1.404(4)	C(5)-C(2)-C(3)	114.01(18)
C(38)-C(39)	1.412(4)	O(2)-C(3)-C(2)	105.10(16)
C(40)-C(41)	1.416(3)	O(2)-C(3)-H(3A)	110.7
C(41)-H(41)	0.9400	O(2)-C(3)-H(3B)	110.7
C(41)-C(42)	1.357(3)	C(2)-C(3)-H(3A)	110.7
C(42)-H(42)	0.9400	C(2)-C(3)-H(3B)	110.7
C(42)-C(48)	1.415(3)	H(3A)-C(3)-H(3B)	108.8
C(43)-H(43)	0.9400	O(2)-C(4)-N(1)	103.67(16)
C(43)-C(44)	1.360(4)	O(2)-C(4)-H(4)	110.1

O(2)-C(4)-C(7)	110.41(16)	C(14)-C(13)-H(13)	119.8
N(1)-C(4)-H(4)	110.1	N(2)-C(14)-C(13)	118.4(2)
N(1)-C(4)-C(7)	112.15(16)	N(2)-C(14)-C(15)	122.3(2)
C(7)-C(4)-H(4)	110.1	C(13)-C(14)-C(15)	119.3(2)
O(3)-C(5)-C(2)	104.33(15)	C(9)-C(15)-C(10)	124.0(2)
O(3)-C(5)-H(5A)	110.9	C(9)-C(15)-C(14)	117.38(19)
O(3)-C(5)-H(5B)	110.9	C(10)-C(15)-C(14)	118.6(2)
C(2)-C(5)-H(5A)	110.9	N(3)-C(16)-C(6)	113.71(17)
C(2)-C(5)-H(5B)	110.9	N(3)-C(16)-C(17)	123.22(18)
H(5A)-C(5)-H(5B)	108.9	C(17)-C(16)-C(6)	122.94(17)
O(3)-C(6)-N(1)	107.11(15)	C(16)-C(17)-H(17)	120.4
O(3)-C(6)-H(6)	109.6	C(18)-C(17)-C(16)	119.11(19)
O(3)-C(6)-C(16)	105.41(15)	C(18)-C(17)-H(17)	120.4
N(1)-C(6)-H(6)	109.6	C(17)-C(18)-H(18)	120.2
N(1)-C(6)-C(16)	115.27(16)	C(17)-C(18)-C(24)	119.6(2)
C(16)-C(6)-H(6)	109.6	C(24)-C(18)-H(18)	120.2
N(2)-C(7)-C(4)	115.65(18)	C(20)-C(19)-H(19)	120.1
N(2)-C(7)-C(8)	124.44(19)	C(20)-C(19)-C(24)	119.8(2)
C(8)-C(7)-C(4)	119.90(19)	C(24)-C(19)-H(19)	120.1
C(7)-C(8)-H(8)	120.8	C(19)-C(20)-H(20)	119.4
C(9)-C(8)-C(7)	118.5(2)	C(19)-C(20)-C(21)	121.1(2)
C(9)-C(8)-H(8)	120.8	C(21)-C(20)-H(20)	119.4
C(8)-C(9)-H(9)	120.0	C(20)-C(21)-H(21)	119.7
C(8)-C(9)-C(15)	120.0(2)	C(22)-C(21)-C(20)	120.6(2)
C(15)-C(9)-H(9)	120.0	C(22)-C(21)-H(21)	119.7
C(11)-C(10)-H(10)	119.7	C(21)-C(22)-H(22)	120.1
C(11)-C(10)-C(15)	120.6(3)	C(21)-C(22)-C(23)	119.8(2)
C(15)-C(10)-H(10)	119.7	C(23)-C(22)-H(22)	120.1
C(10)-C(11)-H(11)	119.8	N(3)-C(23)-C(22)	118.4(2)
C(10)-C(11)-C(12)	120.3(2)	N(3)-C(23)-C(24)	122.48(18)
C(12)-C(11)-H(11)	119.8	C(24)-C(23)-C(22)	119.2(2)
C(11)-C(12)-H(12)	119.6	C(19)-C(24)-C(18)	123.2(2)
C(13)-C(12)-C(11)	120.7(3)	C(19)-C(24)-C(23)	119.5(2)
C(13)-C(12)-H(12)	119.6	C(23)-C(24)-C(18)	117.27(19)
C(12)-C(13)-H(13)	119.8	C(25)-O(4)-H(4A)	114(2)
C(12)-C(13)-C(14)	120.4(3)	C(28)-O(5)-C(27)	104.39(15)

C(30)-O(6)-C(29)	105.17(15)	O(6)-C(30)-N(4)	106.94(16)
C(28)-N(4)-C(26)	106.25(15)	O(6)-C(30)-H(30)	108.3
C(30)-N(4)-C(26)	106.34(15)	O(6)-C(30)-C(40)	112.67(16)
C(30)-N(4)-C(28)	112.60(17)	N(4)-C(30)-H(30)	108.3
C(31)-N(5)-C(38)	117.47(19)	N(4)-C(30)-C(40)	112.27(16)
C(40)-N(6)-C(47)	117.70(17)	C(40)-C(30)-H(30)	108.3
O(4)-C(25)-H(25A)	109.5	N(5)-C(31)-C(28)	116.17(18)
O(4)-C(25)-H(25B)	109.5	N(5)-C(31)-C(32)	124.21(19)
O(4)-C(25)-C(26)	110.82(18)	C(32)-C(31)-C(28)	119.6(2)
H(25A)-C(25)-H(25B)	108.1	C(31)-C(32)-H(32)	120.7
C(26)-C(25)-H(25A)	109.5	C(33)-C(32)-C(31)	118.5(2)
C(26)-C(25)-H(25B)	109.5	C(33)-C(32)-H(32)	120.7
N(4)-C(26)-C(25)	111.05(17)	C(32)-C(33)-H(33)	120.0
N(4)-C(26)-C(27)	102.78(15)	C(32)-C(33)-C(39)	119.9(2)
N(4)-C(26)-C(29)	103.53(16)	C(39)-C(33)-H(33)	120.0
C(25)-C(26)-C(27)	112.27(18)	C(35)-C(34)-H(34)	119.6
C(25)-C(26)-C(29)	112.27(17)	C(35)-C(34)-C(39)	120.9(3)
C(27)-C(26)-C(29)	114.15(18)	C(39)-C(34)-H(34)	119.6
O(5)-C(27)-C(26)	104.22(17)	C(34)-C(35)-H(35)	120.0
O(5)-C(27)-H(27A)	110.9	C(34)-C(35)-C(36)	120.0(3)
O(5)-C(27)-H(27B)	110.9	C(36)-C(35)-H(35)	120.0
C(26)-C(27)-H(27A)	110.9	C(35)-C(36)-H(36)	119.4
C(26)-C(27)-H(27B)	110.9	C(37)-C(36)-C(35)	121.2(3)
H(27A)-C(27)-H(27B)	108.9	C(37)-C(36)-H(36)	119.4
O(5)-C(28)-N(4)	103.95(16)	C(36)-C(37)-H(37)	120.1
O(5)-C(28)-H(28)	110.3	C(36)-C(37)-C(38)	119.8(3)
O(5)-C(28)-C(31)	110.43(16)	C(38)-C(37)-H(37)	120.1
N(4)-C(28)-H(28)	110.3	N(5)-C(38)-C(37)	118.8(2)
N(4)-C(28)-C(31)	111.43(17)	N(5)-C(38)-C(39)	122.0(2)
C(31)-C(28)-H(28)	110.3	C(37)-C(38)-C(39)	119.2(2)
O(6)-C(29)-C(26)	105.72(16)	C(33)-C(39)-C(34)	123.2(3)
O(6)-C(29)-H(29A)	110.6	C(33)-C(39)-C(38)	117.9(2)
O(6)-C(29)-H(29B)	110.6	C(38)-C(39)-C(34)	119.0(3)
C(26)-C(29)-H(29A)	110.6	N(6)-C(40)-C(30)	116.18(17)
C(26)-C(29)-H(29B)	110.6	N(6)-C(40)-C(41)	123.47(19)
H(29A)-C(29)-H(29B)	108.7	C(41)-C(40)-C(30)	120.22(17)

C(40)-C(41)-H(41)	120.5	C(44)-C(45)-H(45)	119.3
C(42)-C(41)-C(40)	119.00(18)	C(46)-C(45)-C(44)	121.4(2)
C(42)-C(41)-H(41)	120.5	C(46)-C(45)-H(45)	119.3
C(41)-C(42)-H(42)	120.2	C(45)-C(46)-H(46)	120.2
C(41)-C(42)-C(48)	119.69(19)	C(45)-C(46)-C(47)	119.5(2)
C(48)-C(42)-H(42)	120.2	C(47)-C(46)-H(46)	120.2
C(44)-C(43)-H(43)	120.0	N(6)-C(47)-C(46)	118.4(2)
C(44)-C(43)-C(48)	120.1(2)	N(6)-C(47)-C(48)	122.74(18)
C(48)-C(43)-H(43)	120.0	C(48)-C(47)-C(46)	118.8(2)
C(43)-C(44)-H(44)	119.8	C(42)-C(48)-C(47)	117.28(19)
C(43)-C(44)-C(45)	120.3(2)	C(43)-C(48)-C(42)	122.9(2)
C(45)-C(44)-H(44)	119.8	C(43)-C(48)-C(47)	119.8(2)

Table S-4. Anisotropic displacement parameters (Å^{2}x 10^3) for jonap24. The anisotropic displacement factor exponent takes the form: $-2\pi^2[h^2 a^{*2}U_{11} + ... + 2 h k a^* b^* U_{12}]$

	U_{11}	U_{22}	U_{33}	U_{23}	U_{13}	U_{12}
O1	61(1)	63(1)	60(1)	-9(1)	12(1)	-22(1)
O2	49(1)	64(1)	42(1)	-16(1)	6(1)	-6(1)
O3	46(1)	53(1)	40(1)	8(1)	5(1)	10(1)
N1	47(1)	35(1)	36(1)	-2(1)	6(1)	-3(1)
N2	46(1)	40(1)	38(1)	0(1)	0(1)	-1(1)
N3	41(1)	38(1)	43(1)	1(1)	6(1)	1(1)
C1	52(1)	41(1)	45(1)	1(1)	4(1)	-6(1)
C2	44(1)	37(1)	38(1)	-3(1)	4(1)	-1(1)
C3	47(1)	57(1)	45(1)	-12(1)	6(1)	-5(1)
C4	47(1)	38(1)	42(1)	-5(1)	3(1)	-1(1)
C5	44(1)	38(1)	44(1)	0(1)	5(1)	4(1)
C6	46(1)	33(1)	41(1)	2(1)	6(1)	-1(1)
C7	49(1)	40(1)	36(1)	-3(1)	5(1)	0(1)
C8	56(1)	49(1)	50(1)	11(1)	8(1)	0(1)
C9	63(1)	58(1)	44(1)	13(1)	7(1)	12(1)
C10	55(1)	91(2)	47(1)	0(1)	-3(1)	14(1)
C11	48(1)	104(3)	66(2)	-13(2)	-2(1)	-4(2)
C12	58(2)	79(2)	75(2)	-13(2)	4(1)	-18(1)
C13	57(1)	57(2)	59(1)	-3(1)	3(1)	-10(1)
C14	47(1)	50(1)	39(1)	-4(1)	4(1)	-2(1)
C15	49(1)	57(1)	36(1)	0(1)	4(1)	8(1)
C16	38(1)	36(1)	39(1)	1(1)	2(1)	-4(1)
C17	39(1)	40(1)	48(1)	6(1)	2(1)	1(1)
C18	39(1)	39(1)	63(1)	5(1)	2(1)	2(1)
C19	43(1)	49(1)	78(2)	-12(1)	7(1)	6(1)
C20	50(1)	70(2)	76(2)	-24(1)	13(1)	6(1)
C21	50(1)	82(2)	53(1)	-12(1)	15(1)	2(1)
C22	47(1)	60(2)	51(1)	0(1)	9(1)	2(1)
C23	35(1)	44(1)	47(1)	-5(1)	6(1)	0(1)
C24	33(1)	41(1)	60(1)	-4(1)	3(1)	1(1)
O4	54(1)	62(1)	64(1)	19(1)	8(1)	15(1)

O5	54(1)	59(1)	47(1)	18(1)	3(1)	-4(1)
O6	63(1)	46(1)	47(1)	-14(1)	7(1)	-12(1)
N4	49(1)	35(1)	37(1)	0(1)	5(1)	1(1)
N5	55(1)	45(1)	40(1)	3(1)	5(1)	5(1)
N6	41(1)	40(1)	42(1)	-5(1)	6(1)	1(1)
C25	51(1)	39(1)	45(1)	-1(1)	5(1)	2(1)
C26	45(1)	36(1)	39(1)	1(1)	1(1)	-2(1)
C27	49(1)	46(1)	53(1)	12(1)	0(1)	-2(1)
C28	52(1)	37(1)	44(1)	4(1)	-1(1)	0(1)
C29	57(1)	43(1)	42(1)	-5(1)	-4(1)	-4(1)
C30	52(1)	36(1)	42(1)	-4(1)	5(1)	0(1)
C31	50(1)	41(1)	38(1)	2(1)	4(1)	2(1)
C32	60(1)	56(1)	51(1)	-11(1)	3(1)	6(1)
C33	69(2)	72(2)	45(1)	-18(1)	5(1)	-7(1)
C34	57(1)	120(3)	51(1)	12(2)	-2(1)	-17(2)
C35	51(2)	150(4)	75(2)	37(2)	-4(1)	-5(2)
C36	57(2)	124(3)	99(2)	42(2)	13(2)	26(2)
C37	64(2)	81(2)	76(2)	18(2)	14(1)	21(2)
C38	48(1)	62(1)	44(1)	11(1)	9(1)	4(1)
C39	50(1)	77(2)	38(1)	5(1)	6(1)	-7(1)
C40	39(1)	38(1)	41(1)	-5(1)	4(1)	4(1)
C41	43(1)	43(1)	44(1)	-7(1)	4(1)	0(1)
C42	39(1)	42(1)	53(1)	-11(1)	2(1)	1(1)
C43	47(1)	54(1)	68(1)	-2(1)	11(1)	-9(1)
C44	64(2)	71(2)	76(2)	8(1)	20(1)	-21(1)
C45	71(2)	85(2)	58(1)	1(1)	25(1)	-17(2)
C46	55(1)	63(2)	51(1)	-4(1)	15(1)	-8(1)
C47	36(1)	44(1)	47(1)	-3(1)	10(1)	2(1)
C48	31(1)	44(1)	56(1)	-2(1)	6(1)	1(1)

Table S-5. Hydrogen coordinates (x 10^4) and isotropic displacement parameters ($Å^2$ x 10^3) for jonap24.

	x	y	z	U(eq)
H1A	2674	10967	1157	56
H1B	2775	10992	1753	56
H3A	3667	7782	824	60
H3B	3677	5530	1107	60
H4	1569	4388	1194	51
H5A	3493	5341	1915	50
H5B	3993	7582	2135	50
H6	1034	5533	1905	48
H8	337	8773	407	62
H9	-1511	8813	3	66
H10	-3450	6978	-145	79
H11	-4581	4176	33	88
H12	-3836	1412	569	86
H13	-1960	1416	917	70
H17	315	10716	1539	51
H18	-668	13188	1953	57
H19	-1411	14261	2759	68
H20	-1757	13309	3549	78
H21	-1156	10010	3900	73
H22	-178	7635	3463	63
H25A	3042	5112	3811	55
H25B	3170	5729	3249	55
H27A	1798	1564	3784	60
H27B	2201	-396	3470	60
H28	4302	-1212	3677	54
H29A	2171	1139	2688	59
H29B	2826	3289	2586	59
H30	5293	311	3139	52
H32	4920	3149	4571	68
H33	6524	3319	5169	75

H34	8346	1590	5571	92
H35	9582	-1226	5610	112
H36	9245	-4057	5039	112
H37	7658	-4096	4437	88
H41	5736	4941	3550	52
H42	6721	7726	3248	54
H43	7315	9607	2495	67
H44	7438	9582	1649	83
H45	6718	6706	1146	84
H46	5828	3886	1476	67
H4A	1380(30)	5890(70)	3057(13)	91(11)
H1	4570(40)	11460(90)	1838(18)	146(17)

Table S-6. Torsion angles [°] for jonap24.

O1-C1-C2-N1	-178.40(15)	C4-C7-C8-C9	-179.1(2)
O1-C1-C2-C3	-64.1(2)	C5-O3-C6-N1	-19.6(2)
O1-C1-C2-C5	67.6(2)	C5-O3-C6-C16	-142.88(16)
O2-C4-C7-N2	131.55(19)	C5-C2-C3-O2	120.6(2)
O2-C4-C7-C8	-49.8(3)	C6-O3-C5-C2	31.5(2)
O3-C6-C16-N3	-60.4(2)	C6-N1-C2-C1	-101.73(18)
O3-C6-C16-C17	115.6(2)	C6-N1-C2-C3	137.96(17)
N1-C2-C3-O2	9.9(2)	C6-N1-C2-C5	19.1(2)
N1-C2-C5-O3	-30.7(2)	C6-N1-C4-O2	-153.03(16)
N1-C4-C7-N2	-113.4(2)	C6-N1-C4-C7	87.9(2)
N1-C4-C7-C8	65.3(2)	C6-C16-C17-C18	-172.31(19)
N1-C6-C16-N3	-178.28(17)	C7-N2-C14-C13	-178.54(19)
N1-C6-C16-C17	-2.3(3)	C7-N2-C14-C15	0.2(3)
N2-C7-C8-C9	-0.6(3)	C7-C8-C9-C15	1.0(3)
N2-C14-C15-C9	0.2(3)	C8-C9-C15-C10	178.6(2)
N2-C14-C15-C10	-179.3(2)	C8-C9-C15-C14	-0.9(3)
N3-C16-C17-C18	3.3(3)	C10-C11-C12-C13	-0.6(4)
N3-C23-C24-C18	3.4(3)	C11-C10-C15-C9	-178.2(2)
N3-C23-C24-C19	-177.7(2)	C11-C10-C15-C14	1.3(4)
C1-C2-C3-O2	-108.0(2)	C11-C12-C13-C14	1.3(4)
C1-C2-C5-O3	87.2(2)	C12-C13-C14-N2	178.1(2)
C2-N1-C4-O2	-34.74(19)	C12-C13-C14-C15	-0.7(3)
C2-N1-C4-C7	-153.85(16)	C13-C14-C15-C9	179.0(2)
C2-N1-C6-O3	-0.9(2)	C13-C14-C15-C10	-0.6(3)
C2-N1-C6-C16	116.02(18)	C14-N2-C7-C4	178.55(17)
C3-O2-C4-N1	41.8(2)	C14-N2-C7-C8	0.0(3)
C3-O2-C4-C7	162.10(18)	C15-C10-C11-C12	-0.8(4)
C3-C2-C5-O3	-141.76(17)	C16-N3-C23-C22	179.77(19)
C4-O2-C3-C2	-32.2(2)	C16-N3-C23-C24	-1.0(3)
C4-N1-C2-C1	134.92(17)	C16-C17-C18-C24	-0.6(3)
C4-N1-C2-C3	14.6(2)	C17-C18-C24-C19	178.6(2)
C4-N1-C2-C5	-104.21(17)	C17-C18-C24-C23	-2.5(3)
C4-N1-C6-O3	116.37(18)	C19-C20-C21-C22	0.5(4)
C4-N1-C6-C16	-126.71(18)	C20-C19-C24-C18	177.2(2)

C20-C19-C24-C23	-1.7(3)	C27-C26-C29-O6	-94.9(2)
C20-C21-C22-C23	-0.6(4)	C28-O5-C27-C26	-40.2(2)
C21-C22-C23-N3	178.8(2)	C28-N4-C26-C25	124.46(17)
C21-C22-C23-C24	-0.5(3)	C28-N4-C26-C27	4.2(2)
C22-C23-C24-C18	-177.32(19)	C28-N4-C26-C29	-114.86(17)
C22-C23-C24-C19	1.6(3)	C28-N4-C30-O6	90.65(19)
C23-N3-C16-C6	173.50(16)	C28-N4-C30-C40	-145.28(17)
C23-N3-C16-C17	-2.5(3)	C28-C31-C32-C33	-178.6(2)
C24-C19-C20-C21	0.6(4)	C29-O6-C30-N4	36.1(2)
O4-C25-C26-N4	-173.83(16)	C29-O6-C30-C40	-87.76(19)
O4-C25-C26-C27	-59.4(2)	C29-C26-C27-O5	132.59(19)
O4-C25-C26-C29	70.8(2)	C30-O6-C29-C26	-31.9(2)
O5-C28-C31-N5	126.7(2)	C30-N4-C26-C25	-115.36(18)
O5-C28-C31-C32	-54.6(3)	C30-N4-C26-C27	124.40(18)
O6-C30-C40-N6	-36.5(2)	C30-N4-C26-C29	5.3(2)
O6-C30-C40-C41	147.47(18)	C30-N4-C28-O5	-144.70(17)
N4-C26-C27-O5	21.2(2)	C30-N4-C28-C31	96.4(2)
N4-C26-C29-O6	16.0(2)	C30-C40-C41-C42	177.65(19)
N4-C28-C31-N5	-118.3(2)	C31-N5-C38-C37	178.2(2)
N4-C28-C31-C32	60.4(3)	C31-N5-C38-C39	-0.2(3)
N4-C30-C40-N6	-157.32(17)	C31-C32-C33-C39	-0.4(4)
N4-C30-C40-C41	26.6(3)	C32-C33-C39-C34	-178.3(2)
N5-C31-C32-C33	0.0(4)	C32-C33-C39-C38	0.4(4)
N5-C38-C39-C33	-0.1(3)	C34-C35-C36-C37	-0.4(5)
N5-C38-C39-C34	178.7(2)	C35-C34-C39-C33	178.3(3)
N6-C40-C41-C42	1.9(3)	C35-C34-C39-C38	-0.4(4)
N6-C47-C48-C42	3.1(3)	C35-C36-C37-C38	0.2(5)
N6-C47-C48-C43	-177.0(2)	C36-C37-C38-N5	-178.6(3)
C25-C26-C27-O5	-98.2(2)	C36-C37-C38-C39	-0.2(4)
C25-C26-C29-O6	135.88(18)	C37-C38-C39-C33	-178.6(2)
C26-N4-C28-O5	-28.67(19)	C37-C38-C39-C34	0.3(3)
C26-N4-C28-C31	-147.62(17)	C38-N5-C31-C28	178.90(18)
C26-N4-C30-O6	-25.3(2)	C38-N5-C31-C32	0.3(3)
C26-N4-C30-C40	98.74(19)	C39-C34-C35-C36	0.5(5)
C27-O5-C28-N4	43.0(2)	C40-N6-C47-C46	-178.57(19)
C27-O5-C28-C31	162.64(18)	C40-N6-C47-C48	-0.2(3)

C40-C41-C42-C48	1.1(3)	C45-C46-C47-N6	177.6(2)
C41-C42-C48-C43	176.7(2)	C45-C46-C47-C48	-0.8(3)
C41-C42-C48-C47	-3.4(3)	C46-C47-C48-C42	-178.6(2)
C43-C44-C45-C46	1.0(5)	C46-C47-C48-C43	1.3(3)
C44-C43-C48-C42	179.2(2)	C47-N6-C40-C30	-178.24(17)
C44-C43-C48-C47	-0.7(3)	C47-N6-C40-C41	-2.3(3)
C44-C45-C46-C47	-0.3(4)	C48-C43-C44-C45	-0.5(4)

Table S-7. Hydrogen bonds and close contacts for jonap24 [Å and °].

D-H...A	d(D-H)	d(H...A)	d(D...A)	<(DHA)
O4-H4A...N3	0.91(4)	2.10(4)	2.950(2)	155(3)
O1-H1...N6#1	0.99(5)	2.05(5)	3.012(2)	163(4)

Symmetry transformations used to generate equivalent atoms:

#1 x,y+1,z

REFERENCE NUMBER: jonap32

1a

CRYSTAL STRUCTURE REPORT

$C_{24}H_{21}Br_2MnN_3O_3$

or

$(\kappa^3\text{-}L^q)MnBr_2$

Report prepared for:

R. Zhang, A. Panda, Prof. W. Jones

November 30, 2023

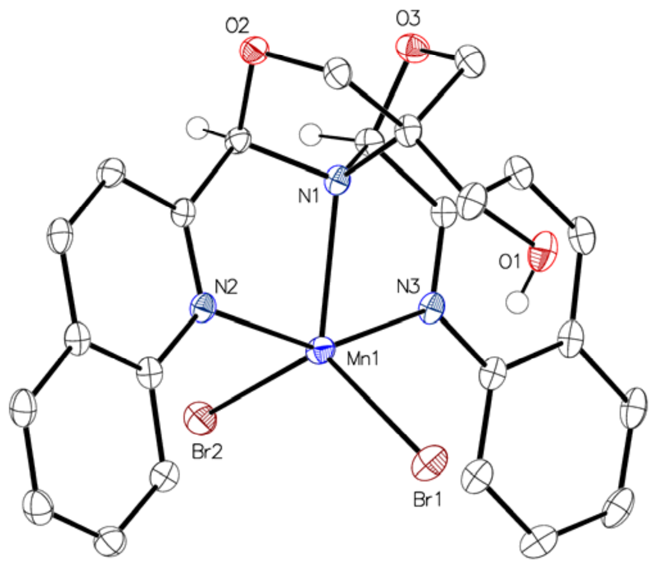

William W. Brennessel
X-ray Crystallographic Facility
Department of Chemistry, University of Rochester
120 Trustee Road
Rochester, NY 14627

Data collection

A crystal (0.13 x 0.062 x 0.026 mm^3) was placed onto a nylon loop and mounted on a Rigaku XtaLAB Synergy-S Dualflex diffractometer equipped with a HyPix-6000HE HPC area detector for data collection at 100.00(10) K. A preliminary set of cell constants and an orientation matrix were calculated from a small sampling of reflections.[1] A short pre-experiment was run, from which an optimal data collection strategy was determined. The full data collection was carried out using a PhotonJet (Cu) X-ray source with frame times of 0.12 and 0.49 seconds and a detector distance of 34.0 mm. Series of frames were collected in 0.50º steps in ω at different 2θ, κ, and ϕ settings. After the intensity data were corrected for absorption, the final cell constants were calculated from the xyz centroids of 26354 strong reflections from the actual data collection after integration.[1] See Table S-1 for additional crystal and refinement information.

Structure solution and refinement

The structure was solved using SHELXT[2] and refined using SHELXL.[3] The space group *P*-1 was determined based on intensity statistics. Most or all non-hydrogen atoms were assigned from the solution. Full-matrix least squares / difference Fourier cycles were performed which located any remaining non-hydrogen atoms. All non-hydrogen atoms were refined with anisotropic displacement parameters. The O-H hydrogen atom was found from the difference Fourier map and then given a riding model. All other hydrogen atoms were placed in ideal positions and refined as riding atoms with relative isotropic displacement parameters. The final full matrix least squares refinement converged to $R1 = 0.0270$ (F^2, $I > 2\sigma(I)$) and $wR2 = 0.0744$ (F^2, all data).

Structure description

The structure is the one suggested. The asymmetric unit contains one molecule in a general position.

Structure manipulation and figure generation were performed using Olex2.[4] Unless noted otherwise all structural diagrams containing anisotropic displacement ellipsoids are drawn at the 50 % probability level.

Data collection, structure solution, and structure refinement were conducted at the X-ray Crystallographic Facility, B04 Hutchison Hall, Department of Chemistry, University of Rochester. The instrument was purchased with funding from NSF MRI program grant CHE-1725028. All publications arising from this report MUST either 1) include William W. Brennessel as a coauthor or 2) acknowledge William W. Brennessel and the X-ray Crystallographic Facility of the Department of Chemistry at the University of Rochester.

[1] *CrysAlisPro*, version 171.42.101a; Rigaku Corporation: Oxford, UK, 2023.

[2] Sheldrick, G. M. *SHELXT*, version 2018/2; *Acta. Crystallogr.* **2015**, *A71*, 3-8.

[3] Sheldrick, G. M. *SHELXL*, version 2019/2; *Acta. Crystallogr.* **2015**, *C71*, 3-8.

[4] Dolomanov, O. V.; Bourhis, L. J.; Gildea, R. J.; Howard, J. A. K.; Puschmann, H. *Olex2*, version 1.5; *J. Appl. Cryst.* **2009**, *42*, 339-341.

Some equations of interest:

$$R_{int} = \Sigma |F_o^2 - \langle F_o^2 \rangle| / \Sigma|F_o^2|$$

$$R1 = \Sigma||F_o|-|F_c|| / \Sigma|F_o|$$

$$wR2 = [\Sigma[w(F_o^2-F_c^2)^2] / \Sigma [w(F_o^2)^2]]^{1/2}$$

where $w = 1 / [\sigma^2 (F_o^2) + (aP)^2 + bP]$ and

$$P = 1/3 \max (0, F_o^2) + 2/3 F_c^2$$

$$GOF = S = [\Sigma[w(F_o^2-F_c^2)^2] / (m-n)]^{1/2}$$

where m = number of reflections and n = number of parameters

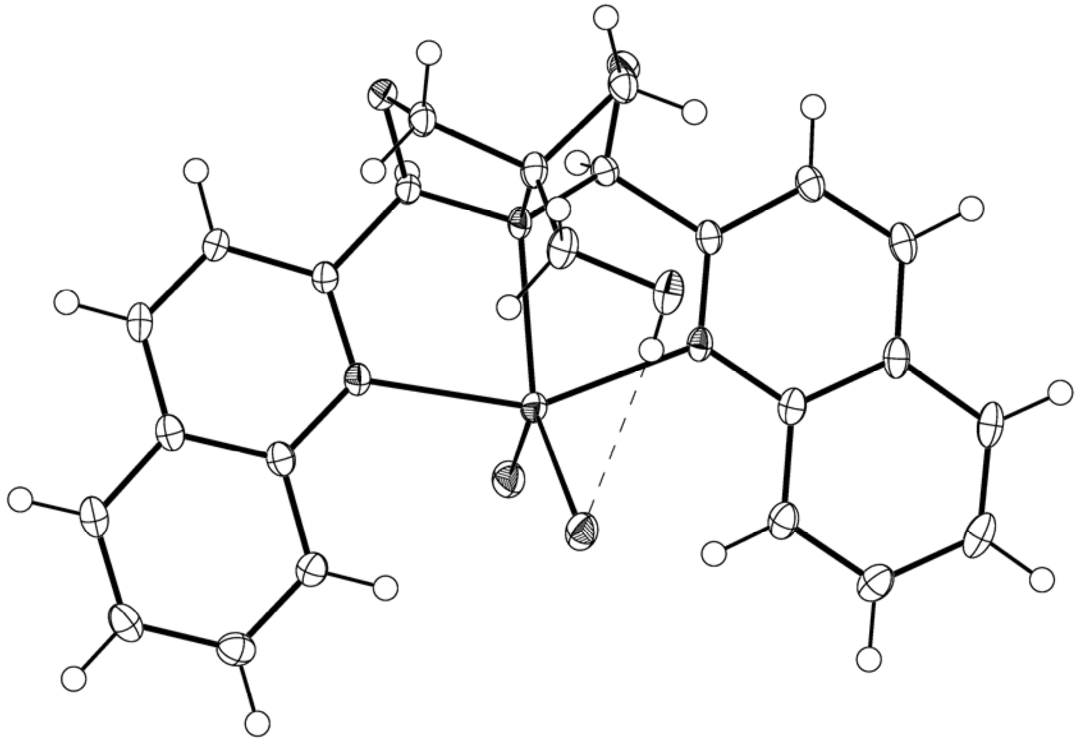

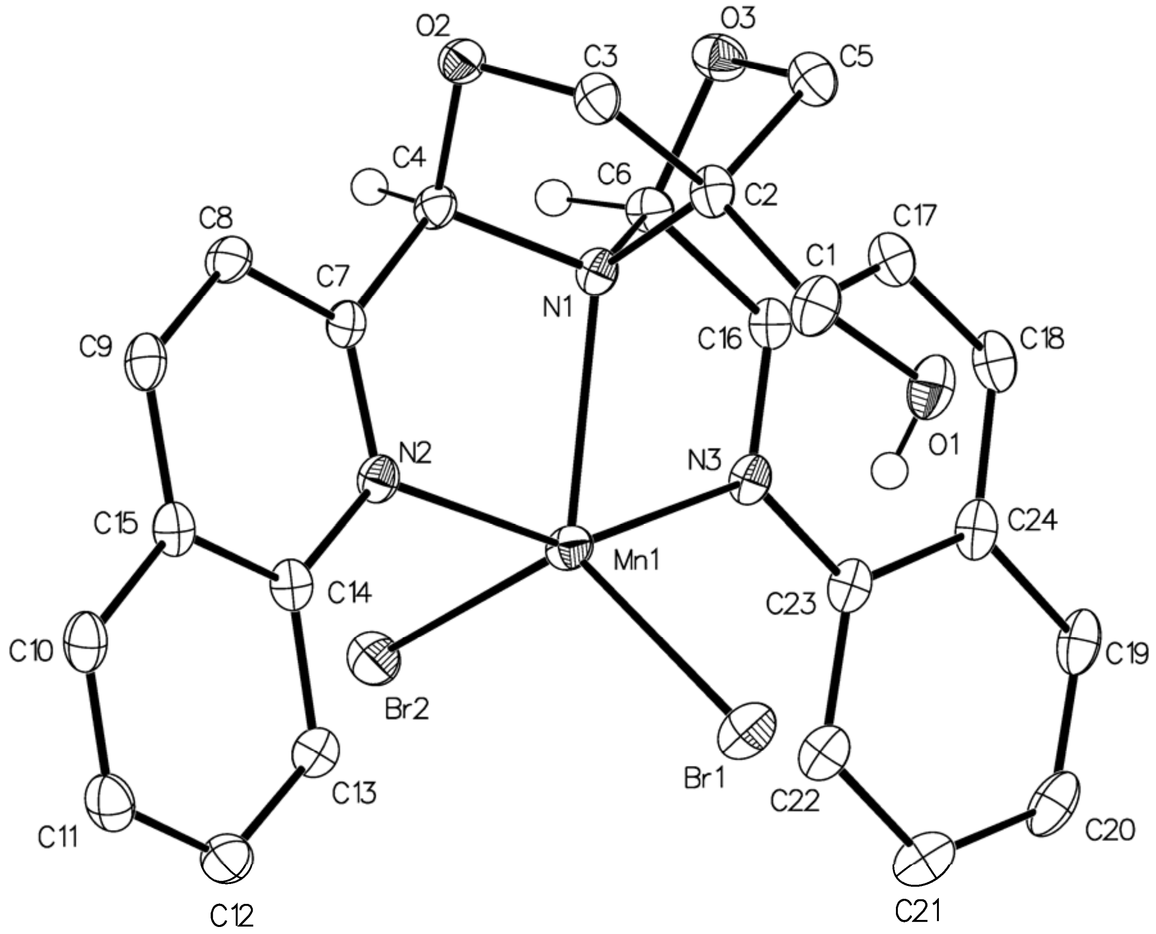
O2
C3
O3
C5
C4
C6
C2
C8
C17
C7
C1
N1
C9
C16
C18
N2
N3
O1
C15
C24
Mn1
C14
C23
C10
C19
Br2
Br1
C13
C22
C11
C20
C12
C21

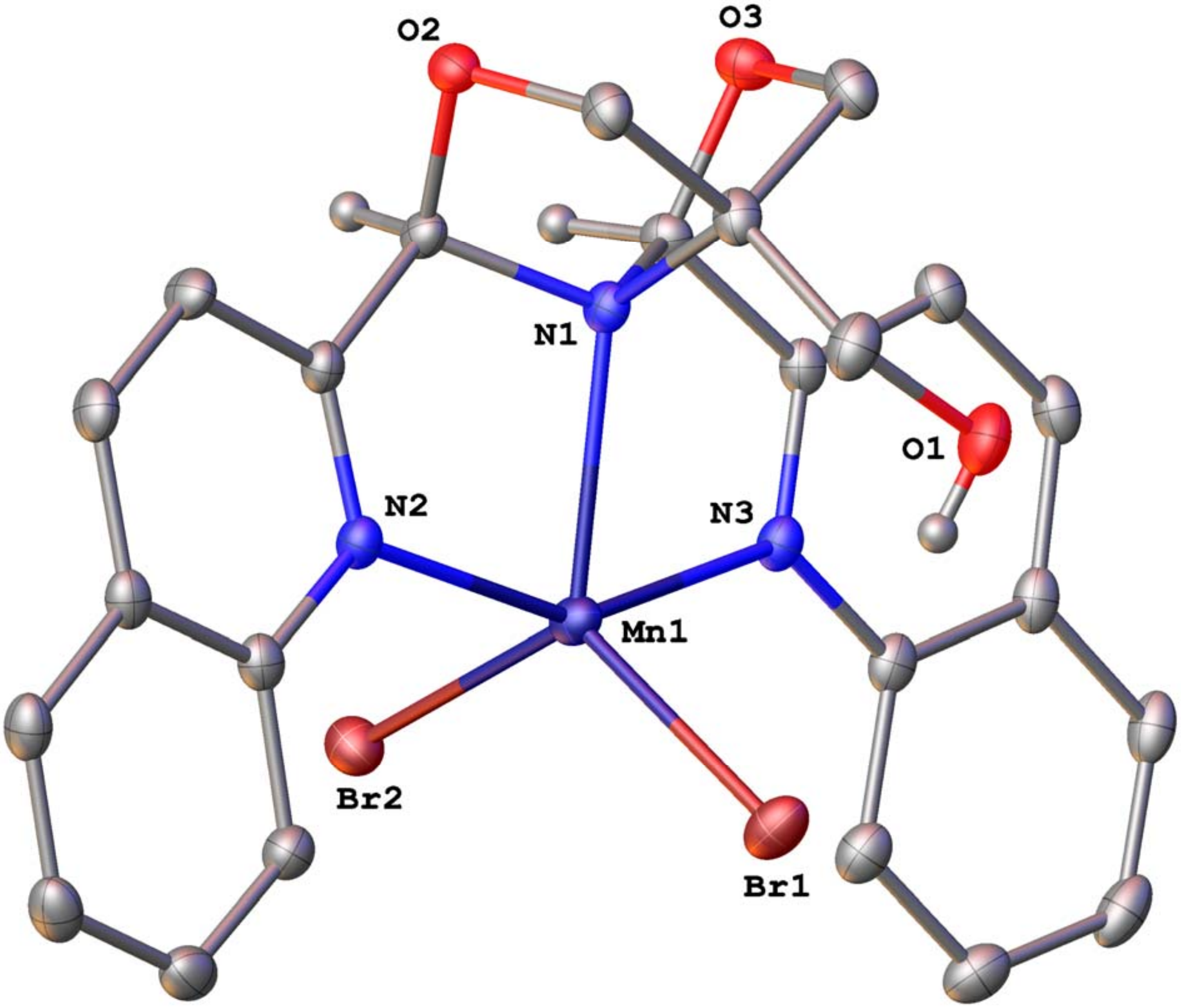
O2
O3
N1
O1
N2
N3
Mn1
Br2
Br1

Table S-1. Crystal data and structure refinement for jonap32.

Identification code	jonap32	
Empirical formula	C24 H21 Br2 Mn N3 O3	
Formula weight	614.20	
Temperature	100.00(10) K	
Wavelength	1.54184 Å	
Crystal system	triclinic	
Space group	*P*-1	
Unit cell dimensions	*a* = 9.3062(2) Å	α = 97.9050(10)°
	b = 10.01440(10) Å	β = 104.983(2)°
	c = 12.6039(2) Å	γ = 95.8840(10)°
Volume	1112.15(3) $Å^3$	
Z	2	
Density (calculated)	1.834 Mg/m^3	
Absorption coefficient	9.316 mm^{-1}	
F(000)	610	
Crystal color, morphology	colourless, block	
Crystal size	0.13 x 0.062 x 0.026 mm^3	
Theta range for data collection	3.684 to 80.322°	
Index ranges	$-11 \le h \le 10$, $-12 \le k \le 12$, $-16 \le l \le 16$	
Reflections collected	37015	
Independent reflections	4760 [*R*(int) = 0.0479]	
Observed reflections	4524	
Completeness to theta = 74.504°	99.8%	
Absorption correction	Multi-scan	
Max. and min. transmission	1.00000 and 0.68926	
Refinement method	Full-matrix least-squares on F^2	
Data / restraints / parameters	4760 / 0 / 299	
Goodness-of-fit on F^2	1.042	
Final *R* indices [*I*>2sigma(*I*)]	*R*1 = 0.0270, *wR*2 = 0.0733	
R indices (all data)	*R*1 = 0.0285, *wR*2 = 0.0744	
Largest diff. peak and hole	0.562 and -0.523 e.$Å^{-3}$	

Table S-2. Atomic coordinates (x 10^4) and equivalent isotropic displacement parameters ($Å^2$x 10^3) for jonap32. U_{eq} is defined as one third of the trace of the orthogonalized U_{ij} tensor.

	x	y	z	U_{eq}
Br1	2275(1)	3491(1)	4368(1)	22(1)
Br2	-504(1)	4354(1)	1200(1)	20(1)
Mn1	1981(1)	3980(1)	2406(1)	15(1)
O1	4919(2)	2207(2)	3518(2)	24(1)
O2	5854(2)	5612(2)	1414(1)	16(1)
O3	4998(2)	2573(2)	512(1)	18(1)
N1	4054(2)	4001(2)	1702(1)	13(1)
N2	2977(2)	6167(2)	2731(2)	14(1)
N3	1791(2)	1849(2)	1535(1)	14(1)
C1	5730(3)	3492(2)	3526(2)	20(1)
C2	5614(2)	3781(2)	2360(2)	15(1)
C3	6588(3)	5139(2)	2415(2)	16(1)
C4	4319(2)	5368(2)	1383(2)	14(1)
C5	5974(3)	2597(2)	1601(2)	18(1)
C6	3640(2)	2892(2)	719(2)	14(1)
C7	3975(2)	6485(2)	2197(2)	14(1)
C8	4722(3)	7825(2)	2312(2)	16(1)
C9	4416(3)	8844(2)	3021(2)	17(1)
C10	2908(3)	9581(2)	4291(2)	19(1)
C11	1795(3)	9274(2)	4779(2)	21(1)
C12	1072(3)	7915(2)	4606(2)	20(1)
C13	1471(3)	6898(2)	3945(2)	17(1)
C14	2613(2)	7193(2)	3420(2)	15(1)
C15	3334(2)	8556(2)	3594(2)	15(1)
C16	2737(2)	1643(2)	928(2)	13(1)
C17	2883(3)	332(2)	419(2)	16(1)
C18	2022(3)	-779(2)	560(2)	17(1)
C19	49(3)	-1720(2)	1366(2)	19(1)
C20	-967(3)	-1489(2)	1966(2)	21(1)
C21	-1102(3)	-150(2)	2396(2)	21(1)
C22	-198(3)	952(2)	2259(2)	18(1)
C23	879(2)	744(2)	1664(2)	14(1)
C24	985(3)	-602(2)	1197(2)	16(1)

Table S-3. Bond lengths [Å] and angles [°] for jonap32.

Br(1)-Mn(1)	2.5383(4)	C(9)-H(9)	0.9500
Br(2)-Mn(1)	2.5127(4)	C(9)-C(15)	1.412(3)
Mn(1)-N(1)	2.3213(18)	C(10)-H(10)	0.9500
Mn(1)-N(2)	2.2278(17)	C(10)-C(11)	1.365(3)
Mn(1)-N(3)	2.2290(17)	C(10)-C(15)	1.414(3)
O(1)-H(1)	0.9360	C(11)-H(11)	0.9500
O(1)-C(1)	1.421(3)	C(11)-C(12)	1.417(3)
O(2)-C(3)	1.438(3)	C(12)-H(12)	0.9500
O(2)-C(4)	1.413(3)	C(12)-C(13)	1.369(3)
O(3)-C(5)	1.432(3)	C(13)-H(13)	0.9500
O(3)-C(6)	1.412(3)	C(13)-C(14)	1.416(3)
N(1)-C(2)	1.528(3)	C(14)-C(15)	1.420(3)
N(1)-C(4)	1.495(3)	C(16)-C(17)	1.415(3)
N(1)-C(6)	1.485(3)	C(17)-H(17)	0.9500
N(2)-C(7)	1.319(3)	C(17)-C(18)	1.363(3)
N(2)-C(14)	1.379(3)	C(18)-H(18)	0.9500
N(3)-C(16)	1.321(3)	C(18)-C(24)	1.416(3)
N(3)-C(23)	1.379(3)	C(19)-H(19)	0.9500
C(1)-H(1A)	0.9900	C(19)-C(20)	1.373(4)
C(1)-H(1B)	0.9900	C(19)-C(24)	1.421(3)
C(1)-C(2)	1.516(3)	C(20)-H(20)	0.9500
C(2)-C(3)	1.538(3)	C(20)-C(21)	1.406(3)
C(2)-C(5)	1.538(3)	C(21)-H(21)	0.9500
C(3)-H(3A)	0.9900	C(21)-C(22)	1.376(3)
C(3)-H(3B)	0.9900	C(22)-H(22)	0.9500
C(4)-H(4)	1.0000	C(22)-C(23)	1.414(3)
C(4)-C(7)	1.528(3)	C(23)-C(24)	1.419(3)
C(5)-H(5A)	0.9900		
C(5)-H(5B)	0.9900	Br(2)-Mn(1)-Br(1)	121.609(16)
C(6)-H(6)	1.0000	N(1)-Mn(1)-Br(1)	118.54(5)
C(6)-C(16)	1.526(3)	N(1)-Mn(1)-Br(2)	119.81(5)
C(7)-C(8)	1.417(3)	N(2)-Mn(1)-Br(1)	101.82(5)
C(8)-H(8)	0.9500	N(2)-Mn(1)-Br(2)	94.29(5)
C(8)-C(9)	1.364(3)	N(2)-Mn(1)-N(1)	75.36(6)

N(2)-Mn(1)-N(3)	150.28(7)	H(3A)-C(3)-H(3B)	109.0
N(3)-Mn(1)-Br(1)	98.36(5)	O(2)-C(4)-N(1)	106.97(16)
N(3)-Mn(1)-Br(2)	93.45(5)	O(2)-C(4)-H(4)	109.7
N(3)-Mn(1)-N(1)	75.89(6)	O(2)-C(4)-C(7)	109.38(16)
C(1)-O(1)-H(1)	102.2	N(1)-C(4)-H(4)	109.7
C(4)-O(2)-C(3)	102.77(16)	N(1)-C(4)-C(7)	111.23(16)
C(6)-O(3)-C(5)	103.81(16)	C(7)-C(4)-H(4)	109.7
C(2)-N(1)-Mn(1)	124.17(13)	O(3)-C(5)-C(2)	105.64(17)
C(4)-N(1)-Mn(1)	107.00(12)	O(3)-C(5)-H(5A)	110.6
C(4)-N(1)-C(2)	103.80(15)	O(3)-C(5)-H(5B)	110.6
C(6)-N(1)-Mn(1)	106.03(12)	C(2)-C(5)-H(5A)	110.6
C(6)-N(1)-C(2)	103.98(16)	C(2)-C(5)-H(5B)	110.6
C(6)-N(1)-C(4)	111.79(16)	H(5A)-C(5)-H(5B)	108.7
C(7)-N(2)-Mn(1)	116.07(14)	O(3)-C(6)-N(1)	106.83(16)
C(7)-N(2)-C(14)	118.55(18)	O(3)-C(6)-H(6)	109.2
C(14)-N(2)-Mn(1)	125.36(14)	O(3)-C(6)-C(16)	110.99(17)
C(16)-N(3)-Mn(1)	115.63(13)	N(1)-C(6)-H(6)	109.2
C(16)-N(3)-C(23)	118.83(18)	N(1)-C(6)-C(16)	111.35(17)
C(23)-N(3)-Mn(1)	125.26(14)	C(16)-C(6)-H(6)	109.2
O(1)-C(1)-H(1A)	109.1	N(2)-C(7)-C(4)	118.82(18)
O(1)-C(1)-H(1B)	109.1	N(2)-C(7)-C(8)	123.4(2)
O(1)-C(1)-C(2)	112.28(18)	C(8)-C(7)-C(4)	117.72(19)
H(1A)-C(1)-H(1B)	107.9	C(7)-C(8)-H(8)	120.6
C(2)-C(1)-H(1A)	109.1	C(9)-C(8)-C(7)	118.8(2)
C(2)-C(1)-H(1B)	109.1	C(9)-C(8)-H(8)	120.6
N(1)-C(2)-C(3)	102.77(16)	C(8)-C(9)-H(9)	120.2
N(1)-C(2)-C(5)	103.08(16)	C(8)-C(9)-C(15)	119.7(2)
C(1)-C(2)-N(1)	115.18(18)	C(15)-C(9)-H(9)	120.2
C(1)-C(2)-C(3)	110.11(18)	C(11)-C(10)-H(10)	119.7
C(1)-C(2)-C(5)	112.75(18)	C(11)-C(10)-C(15)	120.5(2)
C(3)-C(2)-C(5)	112.44(18)	C(15)-C(10)-H(10)	119.7
O(2)-C(3)-C(2)	103.81(17)	C(10)-C(11)-H(11)	119.9
O(2)-C(3)-H(3A)	111.0	C(10)-C(11)-C(12)	120.2(2)
O(2)-C(3)-H(3B)	111.0	C(12)-C(11)-H(11)	119.9
C(2)-C(3)-H(3A)	111.0	C(11)-C(12)-H(12)	119.7
C(2)-C(3)-H(3B)	111.0	C(13)-C(12)-C(11)	120.6(2)

C(13)-C(12)-H(12)	119.7	C(20)-C(19)-H(19)	120.1
C(12)-C(13)-H(13)	119.8	C(20)-C(19)-C(24)	119.8(2)
C(12)-C(13)-C(14)	120.3(2)	C(24)-C(19)-H(19)	120.1
C(14)-C(13)-H(13)	119.8	C(19)-C(20)-H(20)	119.9
N(2)-C(14)-C(13)	119.83(19)	C(19)-C(20)-C(21)	120.3(2)
N(2)-C(14)-C(15)	121.2(2)	C(21)-C(20)-H(20)	119.9
C(13)-C(14)-C(15)	118.9(2)	C(20)-C(21)-H(21)	119.4
C(9)-C(15)-C(10)	122.3(2)	C(22)-C(21)-C(20)	121.3(2)
C(9)-C(15)-C(14)	118.2(2)	C(22)-C(21)-H(21)	119.4
C(10)-C(15)-C(14)	119.5(2)	C(21)-C(22)-H(22)	120.1
N(3)-C(16)-C(6)	117.72(18)	C(21)-C(22)-C(23)	119.8(2)
N(3)-C(16)-C(17)	123.06(19)	C(23)-C(22)-H(22)	120.1
C(17)-C(16)-C(6)	119.12(19)	N(3)-C(23)-C(22)	119.65(19)
C(16)-C(17)-H(17)	120.4	N(3)-C(23)-C(24)	121.2(2)
C(18)-C(17)-C(16)	119.2(2)	C(22)-C(23)-C(24)	119.1(2)
C(18)-C(17)-H(17)	120.4	C(18)-C(24)-C(19)	122.1(2)
C(17)-C(18)-H(18)	120.2	C(18)-C(24)-C(23)	118.11(19)
C(17)-C(18)-C(24)	119.6(2)	C(23)-C(24)-C(19)	119.7(2)
C(24)-C(18)-H(18)	120.2		

Table S-4. Anisotropic displacement parameters ($Å^2$x 10^3) for jonap32. The anisotropic displacement factor exponent takes the form: $-2\pi^2$[$h^2 a^{*2}U_{11}$ + ... + 2 h k a* b* U_{12}]

	U_{11}	U_{22}	U_{33}	U_{23}	U_{13}	U_{12}
Br1	30(1)	17(1)	16(1)	0(1)	7(1)	-3(1)
Br2	18(1)	17(1)	24(1)	1(1)	5(1)	1(1)
Mn1	17(1)	11(1)	16(1)	0(1)	6(1)	-1(1)
O1	30(1)	15(1)	28(1)	7(1)	6(1)	0(1)
O2	15(1)	14(1)	20(1)	3(1)	6(1)	1(1)
O3	18(1)	16(1)	19(1)	-1(1)	7(1)	1(1)
N1	14(1)	9(1)	13(1)	0(1)	2(1)	-1(1)
N2	16(1)	11(1)	14(1)	2(1)	3(1)	1(1)
N3	16(1)	10(1)	12(1)	1(1)	1(1)	-1(1)
C1	21(1)	16(1)	19(1)	3(1)	0(1)	-2(1)
C2	14(1)	11(1)	18(1)	1(1)	1(1)	-1(1)
C3	16(1)	12(1)	19(1)	1(1)	3(1)	0(1)
C4	16(1)	10(1)	14(1)	1(1)	5(1)	0(1)
C5	15(1)	14(1)	23(1)	0(1)	3(1)	1(1)
C6	16(1)	12(1)	14(1)	-1(1)	4(1)	0(1)
C7	16(1)	11(1)	14(1)	3(1)	3(1)	2(1)
C8	18(1)	11(1)	21(1)	2(1)	8(1)	-1(1)
C9	19(1)	10(1)	20(1)	2(1)	2(1)	1(1)
C10	25(1)	12(1)	17(1)	1(1)	2(1)	4(1)
C11	28(1)	18(1)	16(1)	0(1)	5(1)	8(1)
C12	21(1)	24(1)	17(1)	6(1)	7(1)	6(1)
C13	18(1)	16(1)	17(1)	4(1)	5(1)	2(1)
C14	16(1)	13(1)	13(1)	1(1)	1(1)	3(1)
C15	18(1)	13(1)	13(1)	1(1)	0(1)	3(1)
C16	15(1)	11(1)	12(1)	0(1)	0(1)	0(1)
C17	16(1)	13(1)	16(1)	-1(1)	1(1)	2(1)
C18	18(1)	12(1)	17(1)	-1(1)	-2(1)	2(1)
C19	21(1)	12(1)	19(1)	3(1)	-3(1)	-3(1)
C20	23(1)	18(1)	17(1)	6(1)	-2(1)	-7(1)
C21	22(1)	22(1)	15(1)	2(1)	4(1)	-5(1)
C22	20(1)	15(1)	14(1)	1(1)	2(1)	-2(1)
C23	15(1)	13(1)	12(1)	2(1)	-2(1)	-1(1)
C24	18(1)	11(1)	14(1)	1(1)	-3(1)	-1(1)

Table S-5. Hydrogen coordinates (x 10^4) and isotropic displacement parameters ($Å^2$ x 10^3) for jonap32.

	x	y	z	U(eq)
H1	4043	2452	3677	41(10)
H1A	6801	3513	3925	24
H1B	5335	4216	3937	24
H3A	6613	5795	3085	19
H3B	7630	4999	2426	19
H4	3678	5364	611	16
H5A	7040	2751	1596	22
H5B	5782	1725	1862	22
H6	3028	3228	60	17
H8	5423	8010	1902	20
H9	4929	9744	3128	21
H10	3401	10492	4421	23
H11	1503	9973	5235	25
H12	303	7708	4953	24
H13	980	5990	3838	20
H17	3574	229	-15	19
H18	2116	-1667	232	21
H19	127	-2624	1066	23
H20	-1582	-2236	2089	26
H21	-1831	-4	2790	25
H22	-298	1849	2562	21

Table S-6. Torsion angles [°] for jonap32.

Mn1-N1-C2-C1	2.0(2)	C1-C2-C3-O2	-152.48(18)
Mn1-N1-C2-C3	-117.72(15)	C1-C2-C5-O3	144.75(18)
Mn1-N1-C2-C5	125.25(15)	C2-N1-C4-O2	22.7(2)
Mn1-N1-C4-O2	155.55(12)	C2-N1-C4-C7	-96.68(19)
Mn1-N1-C4-C7	36.17(18)	C2-N1-C6-O3	-28.0(2)
Mn1-N1-C6-O3	-160.35(12)	C2-N1-C6-C16	93.35(19)
Mn1-N1-C6-C16	-39.02(19)	C3-O2-C4-N1	-42.23(19)
Mn1-N2-C7-C4	2.2(2)	C3-O2-C4-C7	78.34(18)
Mn1-N2-C7-C8	-179.68(16)	C3-C2-C5-O3	-90.0(2)
Mn1-N2-C14-C13	-3.0(3)	C4-O2-C3-C2	44.12(19)
Mn1-N2-C14-C15	178.66(15)	C4-N1-C2-C1	124.09(18)
Mn1-N3-C16-C6	-10.7(2)	C4-N1-C2-C3	4.3(2)
Mn1-N3-C16-C17	172.95(16)	C4-N1-C2-C5	-112.69(17)
Mn1-N3-C23-C22	9.8(3)	C4-N1-C6-O3	83.4(2)
Mn1-N3-C23-C24	-171.10(15)	C4-N1-C6-C16	-155.28(17)
O1-C1-C2-N1	67.9(2)	C4-C7-C8-C9	179.0(2)
O1-C1-C2-C3	-176.50(18)	C5-O3-C6-N1	41.6(2)
O1-C1-C2-C5	-50.0(3)	C5-O3-C6-C16	-80.00(19)
O2-C4-C7-N2	-145.52(19)	C5-C2-C3-O2	80.9(2)
O2-C4-C7-C8	36.3(2)	C6-O3-C5-C2	-37.8(2)
O3-C6-C16-N3	154.36(18)	C6-N1-C2-C1	-118.84(19)
O3-C6-C16-C17	-29.1(3)	C6-N1-C2-C3	121.42(17)
N1-C2-C3-O2	-29.3(2)	C6-N1-C2-C5	4.4(2)
N1-C2-C5-O3	19.9(2)	C6-N1-C4-O2	-88.77(19)
N1-C4-C7-N2	-27.6(3)	C6-N1-C4-C7	151.84(17)
N1-C4-C7-C8	154.20(19)	C6-C16-C17-C18	-176.74(19)
N1-C6-C16-N3	35.5(3)	C7-N2-C14-C13	175.7(2)
N1-C6-C16-C17	-147.98(19)	C7-N2-C14-C15	-2.6(3)
N2-C7-C8-C9	0.9(3)	C7-C8-C9-C15	-2.1(3)
N2-C14-C15-C9	1.4(3)	C8-C9-C15-C10	-176.4(2)
N2-C14-C15-C10	178.9(2)	C8-C9-C15-C14	1.0(3)
N3-C16-C17-C18	-0.4(3)	C10-C11-C12-C13	-0.5(4)
N3-C23-C24-C18	-2.1(3)	C11-C10-C15-C9	176.1(2)
N3-C23-C24-C19	178.66(19)	C11-C10-C15-C14	-1.2(3)

C11-C12-C13-C14	-0.2(3)	C17-C18-C24-C23	0.3(3)
C12-C13-C14-N2	-178.2(2)	C19-C20-C21-C22	-1.9(4)
C12-C13-C14-C15	0.1(3)	C20-C19-C24-C18	-178.2(2)
C13-C14-C15-C9	-176.9(2)	C20-C19-C24-C23	1.0(3)
C13-C14-C15-C10	0.5(3)	C20-C21-C22-C23	0.7(3)
C14-N2-C7-C4	-176.62(18)	C21-C22-C23-N3	-179.5(2)
C14-N2-C7-C8	1.5(3)	C21-C22-C23-C24	1.4(3)
C15-C10-C11-C12	1.2(3)	C22-C23-C24-C18	177.0(2)
C16-N3-C23-C22	-176.53(19)	C22-C23-C24-C19	-2.2(3)
C16-N3-C23-C24	2.6(3)	C23-N3-C16-C6	175.07(18)
C16-C17-C18-C24	0.9(3)	C23-N3-C16-C17	-1.3(3)
C17-C18-C24-C19	179.6(2)	C24-C19-C20-C21	1.0(3)

Table S-7. Hydrogen bonds and close contacts for jonap32 [Å and °].

D-H...A	d(D-H)	d(H...A)	d(D...A)	<(DHA)
O1-H1...Br1	0.94	2.32	3.2383(19)	166.1

REFERENCE NUMBER: jonap33

1c

CRYSTAL STRUCTURE REPORT

$C_{24} H_{21} Br_2 Co N_3 O_3$

or

$(\kappa^3\text{-}L^q)CoBr_2$

Report prepared for:

R. Zhang, A. Panda, Prof. W. Jones

November 30, 2023

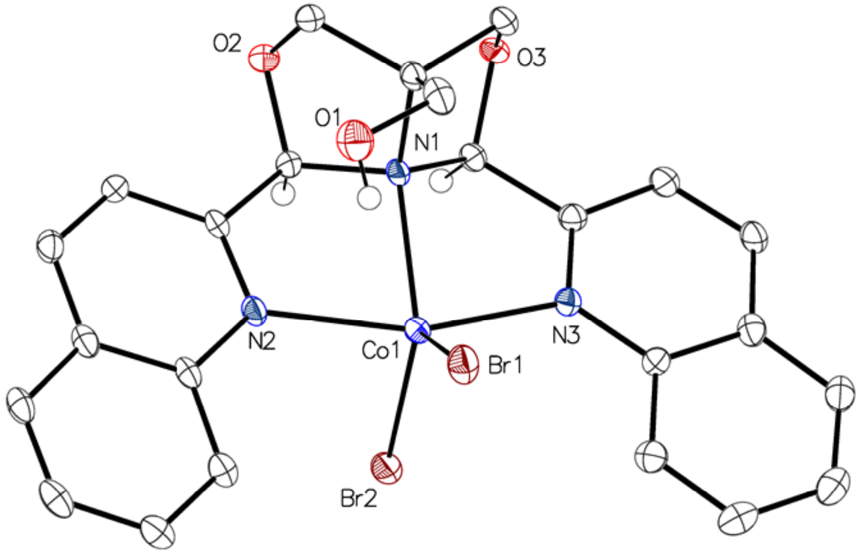

William W. Brennessel

X-ray Crystallographic Facility

Department of Chemistry, University of Rochester

120 Trustee Road

Rochester, NY 14627

Data collection

A crystal (0.28 x 0.135 x 0.026 mm^3) was placed onto a nylon loop and mounted on a Rigaku XtaLAB Synergy-S Dualflex diffractometer equipped with a HyPix-6000HE HPC area detector for data collection at 100.00(10) K. A preliminary set of cell constants and an orientation matrix were calculated from a small sampling of reflections.[1] A short pre-experiment was run, from which an optimal data collection strategy was determined. The full data collection was carried out using a PhotonJet (Cu) X-ray source with frame times of 0.19 and 0.76 seconds and a detector distance of 34.0 mm. Series of frames were collected in 0.50º steps in ω at different 2θ, κ, and ϕ settings. After the intensity data were corrected for absorption, the final cell constants were calculated from the xyz centroids of 20513 strong reflections from the actual data collection after integration.[1] See Table S-1 for additional crystal and refinement information.

Structure solution and refinement

The structure was solved using SHELXT[2] and refined using SHELXL.[3] The space group *P*-1 was determined based on intensity statistics. Most or all non-hydrogen atoms were assigned from the solution. Full-matrix least squares / difference Fourier cycles were performed which located any remaining non-hydrogen atoms. All non-hydrogen atoms were refined with anisotropic displacement parameters. The O-H hydrogen atom was found from the difference Fourier map and refined freely. All other hydrogen atoms were placed in ideal positions and refined as riding atoms with relative isotropic displacement parameters. The final full matrix least squares refinement converged to $R1 = 0.0341$ (F^2, $I > 2\sigma(I)$) and $wR2 = 0.0895$ (F^2, all data).

Structure description

The structure is the one suggested. The asymmetric unit contains one molecule in a general position.

Structure manipulation and figure generation were performed using Olex2.[4] Unless noted otherwise all structural diagrams containing anisotropic displacement ellipsoids are drawn at the 50 % probability level.

Data collection, structure solution, and structure refinement were conducted at the X-ray Crystallographic Facility, B04 Hutchison Hall, Department of Chemistry, University of Rochester. The instrument was purchased with funding from NSF MRI program grant CHE-1725028. All publications arising from this report MUST either 1) include William W. Brennessel as a coauthor or 2) acknowledge William W. Brennessel and the X-ray Crystallographic Facility of the Department of Chemistry at the University of Rochester.

1 *CrysAlisPro*, version 171.42.101a; Rigaku Corporation: Oxford, UK, 2023.

2 Sheldrick, G. M. *SHELXT*, version 2018/2; *Acta. Crystallogr.* **2015**, *A71*, 3-8.

3 Sheldrick, G. M. *SHELXL*, version 2019/2; *Acta. Crystallogr.* **2015**, *C71*, 3-8.

4 Dolomanov, O. V.; Bourhis, L. J.; Gildea, R. J.; Howard, J. A. K.; Puschmann, H. *Olex2*, version 1.5; *J. Appl. Cryst.* **2009**, *42*, 339-341.

Some equations of interest:

$$R_{int} = \Sigma |F_o^2 - \langle F_o^2 \rangle| / \Sigma |F_o^2|$$

$$R1 = \Sigma ||F_o| - |F_c|| / \Sigma |F_o|$$

$$wR2 = [\Sigma[w(F_o^2 - F_c^2)^2] / \Sigma [w(F_o^2)^2]]^{1/2}$$

where $w = 1 / [\sigma^2 (F_o^2) + (aP)^2 + bP]$ and

$$P = 1/3 \max (0, F_o^2) + 2/3 F_c^2$$

$$\text{GOF} = S = [\Sigma[w(F_o^2 - F_c^2)^2] / (m-n)]^{1/2}$$

where m = number of reflections and n = number of parameters

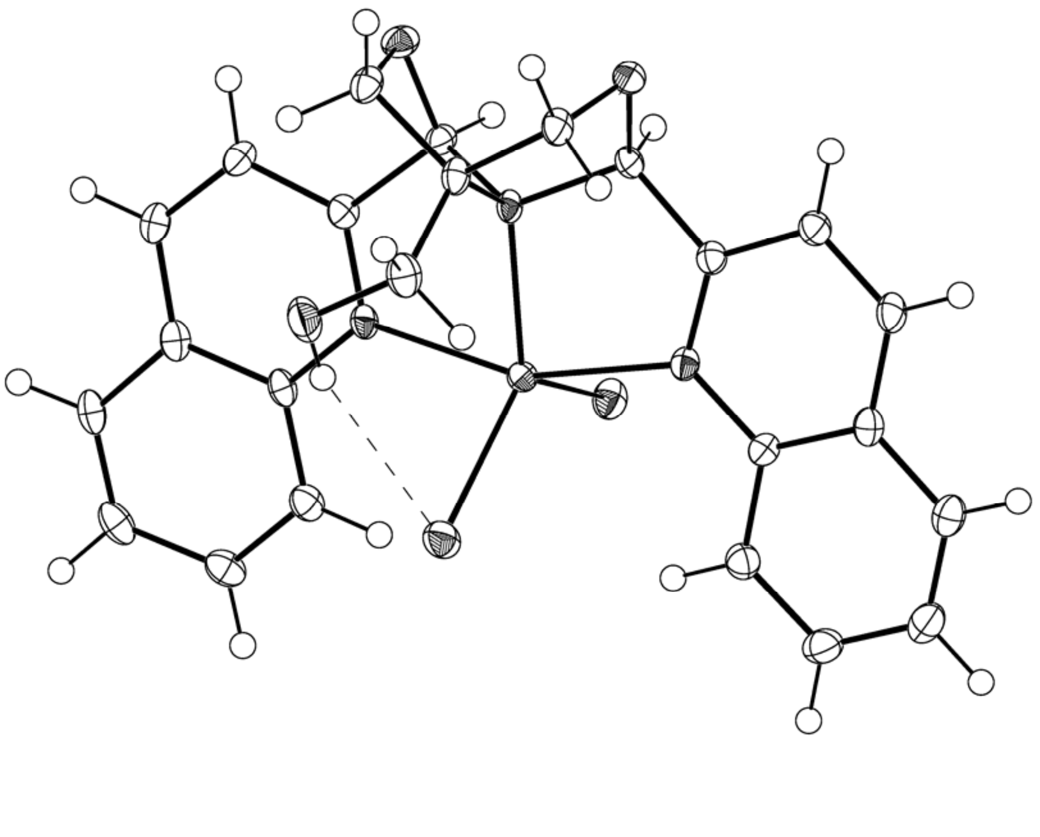

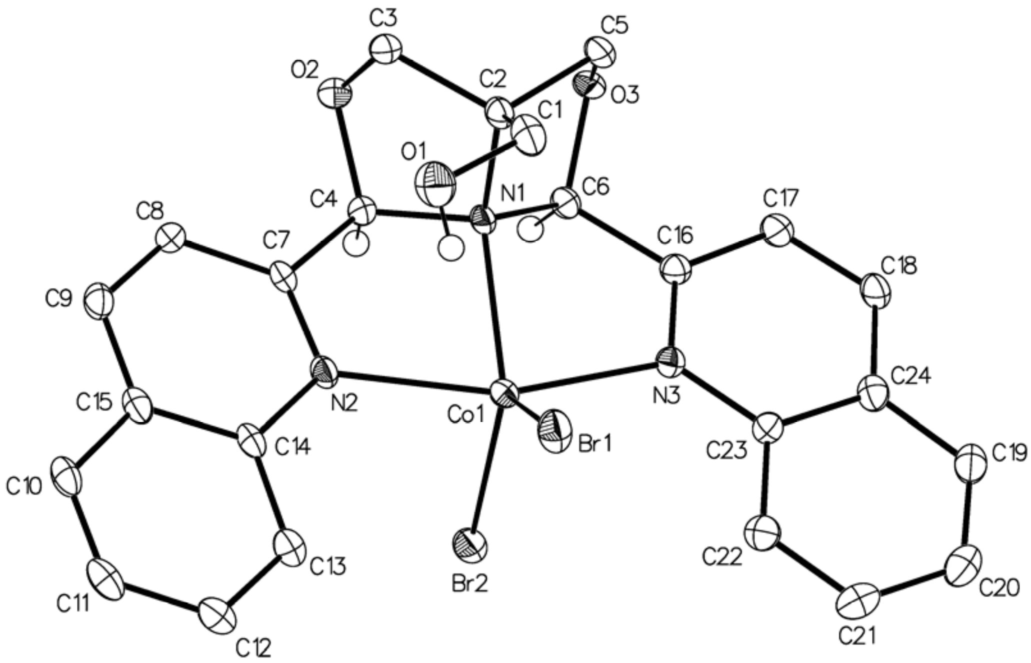
C3
C5
O2
C2
O3
C1
O1
N1
C4
C6
C8
C17
C7
C16
C18
C9
N2
N3
C24
C15
Co1
Br1
C14
C23
C19
C10
C13
C22
Br2
C11
C20
C12
C21

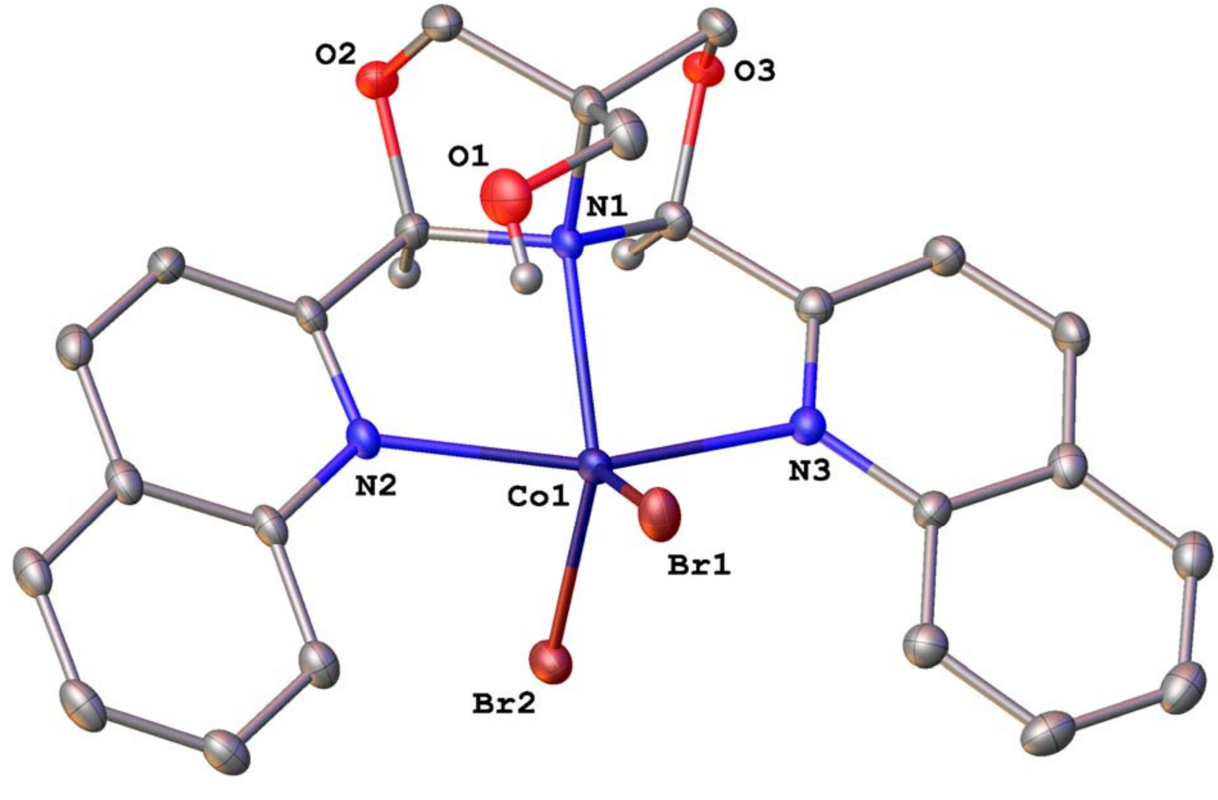
O2
O3
O1
N1
N2
Co1
N3
Br1
Br2

Table S-1. Crystal data and structure refinement for jonap33.

Identification code	jonap33	
Empirical formula	C24 H21 Br2 Co N3 O3	
Formula weight	618.19	
Temperature	100.00(10) K	
Wavelength	1.54184 Å	
Crystal system	triclinic	
Space group	*P*-1	
Unit cell dimensions	*a* = 9.14455(15) Å	α = 97.9974(13)°
	b = 9.99922(14) Å	β = 104.8311(15)°
	c = 12.6688(2) Å	γ = 96.5679(13)°
Volume	1095.24(3) $Å^3$	
Z	2	
Density (calculated)	1.875 Mg/m^3	
Absorption coefficient	10.701 mm^{-1}	
F(000)	614	
Crystal color, morphology	violet, plate	
Crystal size	0.28 x 0.135 x 0.026 mm^3	
Theta range for data collection	3.666 to 80.052°	
Index ranges	$-11 \le h \le 10$, $-12 \le k \le 12$, $-16 \le l \le 16$	
Reflections collected	33878	
Independent reflections	4671 [*R*(int) = 0.0580]	
Observed reflections	4509	
Completeness to theta = 74.504°	99.6%	
Absorption correction	Multi-scan	
Max. and min. transmission	1.00000 and 0.35317	
Refinement method	Full-matrix least-squares on F^2	
Data / restraints / parameters	4671 / 0 / 302	
Goodness-of-fit on F^2	1.045	
Final *R* indices [*I*>2sigma(*I*)]	*R*1 = 0.0341, *wR*2 = 0.0888	
R indices (all data)	*R*1 = 0.0352, *wR*2 = 0.0895	
Largest diff. peak and hole	1.032 and -0.579 e.$Å^{-3}$	

Table S-2. Atomic coordinates (x 10^4) and equivalent isotropic displacement parameters (Å^{2}x 10^3) for jonap33. U_{eq} is defined as one third of the trace of the orthogonalized U_{ij} tensor.

	x	y	z	U_{eq}
Br1	7518(1)	6526(1)	5712(1)	22(1)
Br2	10239(1)	5705(1)	8787(1)	20(1)
Co1	7810(1)	6056(1)	7612(1)	14(1)
O1	4878(3)	7868(2)	6527(2)	26(1)
O2	4979(2)	7436(2)	9536(2)	18(1)
O3	4062(2)	4382(2)	8594(2)	17(1)
N1	5874(2)	6027(2)	8295(2)	13(1)
N2	8157(3)	8150(2)	8413(2)	14(1)
N3	6970(3)	3919(2)	7243(2)	14(1)
C1	4075(3)	6561(3)	6519(2)	21(1)
C2	4260(3)	6244(3)	7680(2)	15(1)
C3	3937(3)	7414(3)	8473(2)	18(1)
C4	6340(3)	7133(3)	9286(2)	14(1)
C5	3278(3)	4870(3)	7623(2)	17(1)
C6	5617(3)	4646(3)	8604(2)	15(1)
C7	7243(3)	8383(3)	9054(2)	14(1)
C8	7135(3)	9700(3)	9567(2)	16(1)
C9	8020(3)	10802(3)	9400(2)	17(1)
C10	10026(3)	11703(3)	8570(2)	18(1)
C11	11055(3)	11453(3)	7983(2)	20(1)
C12	11161(3)	10101(3)	7557(2)	19(1)
C13	10205(3)	9011(3)	7686(2)	18(1)
C14	9118(3)	9245(3)	8280(2)	14(1)
C15	9050(3)	10597(3)	8753(2)	16(1)
C16	5963(3)	3554(3)	7778(2)	14(1)
C17	5236(3)	2190(3)	7660(2)	17(1)
C18	5579(3)	1191(3)	6955(2)	18(1)
C19	7168(3)	513(3)	5717(2)	19(1)
C20	8317(4)	869(3)	5251(2)	22(1)
C21	9023(3)	2244(3)	5426(2)	22(1)
C22	8562(3)	3249(3)	6065(2)	18(1)
C23	7388(3)	2911(3)	6567(2)	14(1)
C24	6688(3)	1525(3)	6394(2)	16(1)

Table S-3. Bond lengths [Å] and angles [°] for jonap33.

Br(1)-Co(1)	2.4703(5)	C(9)-H(9)	0.9500
Br(2)-Co(1)	2.4291(5)	C(9)-C(15)	1.412(4)
Co(1)-N(1)	2.159(2)	C(10)-H(10)	0.9500
Co(1)-N(2)	2.146(2)	C(10)-C(11)	1.364(4)
Co(1)-N(3)	2.129(2)	C(10)-C(15)	1.423(4)
O(1)-C(1)	1.421(4)	C(11)-H(11)	0.9500
O(1)-H(1)	0.77(4)	C(11)-C(12)	1.408(4)
O(2)-C(3)	1.433(3)	C(12)-H(12)	0.9500
O(2)-C(4)	1.414(3)	C(12)-C(13)	1.373(4)
O(3)-C(5)	1.436(3)	C(13)-H(13)	0.9500
O(3)-C(6)	1.413(3)	C(13)-C(14)	1.413(4)
N(1)-C(2)	1.537(3)	C(14)-C(15)	1.415(4)
N(1)-C(4)	1.488(3)	C(16)-C(17)	1.417(4)
N(1)-C(6)	1.496(3)	C(17)-H(17)	0.9500
N(2)-C(7)	1.324(3)	C(17)-C(18)	1.365(4)
N(2)-C(14)	1.381(3)	C(18)-H(18)	0.9500
N(3)-C(16)	1.323(3)	C(18)-C(24)	1.415(4)
N(3)-C(23)	1.382(3)	C(19)-H(19)	0.9500
C(1)-H(1A)	0.9900	C(19)-C(20)	1.368(4)
C(1)-H(1B)	0.9900	C(19)-C(24)	1.415(4)
C(1)-C(2)	1.518(4)	C(20)-H(20)	0.9500
C(2)-C(3)	1.539(4)	C(20)-C(21)	1.413(4)
C(2)-C(5)	1.535(4)	C(21)-H(21)	0.9500
C(3)-H(3A)	0.9900	C(21)-C(22)	1.375(4)
C(3)-H(3B)	0.9900	C(22)-H(22)	0.9500
C(4)-H(4)	1.0000	C(22)-C(23)	1.413(4)
C(4)-C(7)	1.523(4)	C(23)-C(24)	1.422(4)
C(5)-H(5A)	0.9900	Br(2)-Co(1)-Br(1)	122.37(2)
C(5)-H(5B)	0.9900	N(1)-Co(1)-Br(1)	119.49(6)
C(6)-H(6)	1.0000	N(1)-Co(1)-Br(2)	118.10(6)
C(6)-C(16)	1.523(4)	N(2)-Co(1)-Br(1)	96.44(6)
C(7)-C(8)	1.411(4)	N(2)-Co(1)-Br(2)	91.31(6)
C(8)-H(8)	0.9500	N(2)-Co(1)-N(1)	79.79(8)
C(8)-C(9)	1.365(4)	N(3)-Co(1)-Br(1)	99.72(6)

N(3)-Co(1)-Br(2)	91.82(6)	O(2)-C(4)-N(1)	106.8(2)
N(3)-Co(1)-N(1)	80.05(8)	O(2)-C(4)-H(4)	109.3
N(3)-Co(1)-N(2)	158.58(9)	O(2)-C(4)-C(7)	111.7(2)
C(1)-O(1)-H(1)	106(3)	N(1)-C(4)-H(4)	109.3
C(4)-O(2)-C(3)	103.94(19)	N(1)-C(4)-C(7)	110.5(2)
C(6)-O(3)-C(5)	103.40(19)	C(7)-C(4)-H(4)	109.3
C(2)-N(1)-Co(1)	125.28(16)	O(3)-C(5)-C(2)	103.9(2)
C(4)-N(1)-Co(1)	105.74(15)	O(3)-C(5)-H(5A)	111.0
C(4)-N(1)-C(2)	103.90(19)	O(3)-C(5)-H(5B)	111.0
C(4)-N(1)-C(6)	111.7(2)	C(2)-C(5)-H(5A)	111.0
C(6)-N(1)-Co(1)	106.37(15)	C(2)-C(5)-H(5B)	111.0
C(6)-N(1)-C(2)	103.72(19)	H(5A)-C(5)-H(5B)	109.0
C(7)-N(2)-Co(1)	113.22(17)	O(3)-C(6)-N(1)	106.9(2)
C(7)-N(2)-C(14)	118.5(2)	O(3)-C(6)-H(6)	109.7
C(14)-N(2)-Co(1)	128.17(18)	O(3)-C(6)-C(16)	110.0(2)
C(16)-N(3)-Co(1)	113.29(17)	N(1)-C(6)-H(6)	109.7
C(16)-N(3)-C(23)	118.4(2)	N(1)-C(6)-C(16)	110.8(2)
C(23)-N(3)-Co(1)	128.26(18)	C(16)-C(6)-H(6)	109.7
O(1)-C(1)-H(1A)	109.1	N(2)-C(7)-C(4)	116.7(2)
O(1)-C(1)-H(1B)	109.1	N(2)-C(7)-C(8)	123.7(2)
O(1)-C(1)-C(2)	112.3(2)	C(8)-C(7)-C(4)	119.5(2)
H(1A)-C(1)-H(1B)	107.9	C(7)-C(8)-H(8)	120.7
C(2)-C(1)-H(1A)	109.1	C(9)-C(8)-C(7)	118.7(2)
C(2)-C(1)-H(1B)	109.1	C(9)-C(8)-H(8)	120.7
N(1)-C(2)-C(3)	102.9(2)	C(8)-C(9)-H(9)	120.2
C(1)-C(2)-N(1)	115.6(2)	C(8)-C(9)-C(15)	119.5(3)
C(1)-C(2)-C(3)	112.5(2)	C(15)-C(9)-H(9)	120.2
C(1)-C(2)-C(5)	109.8(2)	C(11)-C(10)-H(10)	120.0
C(5)-C(2)-N(1)	102.8(2)	C(11)-C(10)-C(15)	120.1(3)
C(5)-C(2)-C(3)	112.7(2)	C(15)-C(10)-H(10)	120.0
O(2)-C(3)-C(2)	105.9(2)	C(10)-C(11)-H(11)	119.8
O(2)-C(3)-H(3A)	110.6	C(10)-C(11)-C(12)	120.3(3)
O(2)-C(3)-H(3B)	110.6	C(12)-C(11)-H(11)	119.8
C(2)-C(3)-H(3A)	110.6	C(11)-C(12)-H(12)	119.4
C(2)-C(3)-H(3B)	110.6	C(13)-C(12)-C(11)	121.2(3)
H(3A)-C(3)-H(3B)	108.7	C(13)-C(12)-H(12)	119.4

C(12)-C(13)-H(13)	120.2	C(20)-C(19)-H(19)	120.0
C(12)-C(13)-C(14)	119.5(3)	C(20)-C(19)-C(24)	120.0(3)
C(14)-C(13)-H(13)	120.2	C(24)-C(19)-H(19)	120.0
N(2)-C(14)-C(13)	119.6(2)	C(19)-C(20)-H(20)	119.7
N(2)-C(14)-C(15)	120.8(2)	C(19)-C(20)-C(21)	120.6(3)
C(13)-C(14)-C(15)	119.6(2)	C(21)-C(20)-H(20)	119.7
C(9)-C(15)-C(10)	122.1(3)	C(20)-C(21)-H(21)	119.7
C(9)-C(15)-C(14)	118.7(3)	C(22)-C(21)-C(20)	120.5(3)
C(14)-C(15)-C(10)	119.2(3)	C(22)-C(21)-H(21)	119.7
N(3)-C(16)-C(6)	118.3(2)	C(21)-C(22)-H(22)	119.9
N(3)-C(16)-C(17)	123.7(2)	C(21)-C(22)-C(23)	120.2(3)
C(17)-C(16)-C(6)	118.0(2)	C(23)-C(22)-H(22)	119.9
C(16)-C(17)-H(17)	120.6	N(3)-C(23)-C(22)	119.8(2)
C(18)-C(17)-C(16)	118.7(3)	N(3)-C(23)-C(24)	121.1(2)
C(18)-C(17)-H(17)	120.6	C(22)-C(23)-C(24)	119.1(2)
C(17)-C(18)-H(18)	120.2	C(18)-C(24)-C(19)	122.0(3)
C(17)-C(18)-C(24)	119.7(3)	C(18)-C(24)-C(23)	118.4(2)
C(24)-C(18)-H(18)	120.2	C(19)-C(24)-C(23)	119.6(3)

Table S-4. Anisotropic displacement parameters ($Å^2$x 10^3) for jonap33. The anisotropic displacement factor exponent takes the form: $-2\pi^2[h^2 a^{*2}U_{11} + ... + 2 h k a^* b^* U_{12}]$

	U_{11}	U_{22}	U_{33}	U_{23}	U_{13}	U_{12}
Br1	27(1)	18(1)	19(1)	1(1)	8(1)	-2(1)
Br2	16(1)	16(1)	26(1)	2(1)	4(1)	1(1)
Co1	13(1)	12(1)	18(1)	1(1)	6(1)	-1(1)
O1	30(1)	17(1)	33(1)	9(1)	10(1)	2(1)
O2	16(1)	18(1)	22(1)	0(1)	9(1)	0(1)
O3	13(1)	14(1)	25(1)	2(1)	9(1)	-1(1)
N1	12(1)	8(1)	17(1)	1(1)	3(1)	0(1)
N2	14(1)	10(1)	17(1)	2(1)	3(1)	-1(1)
N3	13(1)	11(1)	17(1)	2(1)	4(1)	2(1)
C1	21(1)	17(1)	22(1)	3(1)	1(1)	0(1)
C2	13(1)	11(1)	21(1)	2(1)	3(1)	1(1)
C3	13(1)	14(1)	25(1)	1(1)	6(1)	2(1)
C4	14(1)	12(1)	18(1)	0(1)	7(1)	0(1)
C5	13(1)	12(1)	25(1)	1(1)	6(1)	-1(1)
C6	15(1)	11(1)	19(1)	3(1)	7(1)	0(1)
C7	12(1)	12(1)	15(1)	-1(1)	1(1)	-2(1)
C8	13(1)	14(1)	20(1)	-1(1)	5(1)	2(1)
C9	17(1)	12(1)	20(1)	1(1)	1(1)	3(1)
C10	18(1)	11(1)	21(1)	4(1)	-1(1)	-1(1)
C11	19(1)	18(1)	20(1)	6(1)	1(1)	-5(1)
C12	16(1)	22(1)	18(1)	4(1)	4(1)	-3(1)
C13	18(1)	15(1)	18(1)	2(1)	4(1)	-2(1)
C14	14(1)	11(1)	16(1)	3(1)	0(1)	-2(1)
C15	14(1)	12(1)	17(1)	2(1)	-1(1)	0(1)
C16	13(1)	13(1)	16(1)	3(1)	4(1)	2(1)
C17	16(1)	14(1)	22(1)	3(1)	7(1)	0(1)
C18	16(1)	11(1)	26(1)	2(1)	5(1)	-1(1)
C19	21(1)	16(1)	19(1)	0(1)	1(1)	4(1)
C20	26(2)	21(1)	19(1)	2(1)	5(1)	10(1)
C21	22(1)	27(2)	21(1)	6(1)	9(1)	10(1)
C22	18(1)	17(1)	19(1)	4(1)	5(1)	3(1)
C23	14(1)	14(1)	15(1)	1(1)	4(1)	3(1)
C24	17(1)	14(1)	17(1)	3(1)	1(1)	3(1)

Table S-5. Hydrogen coordinates (x 10^4) and isotropic displacement parameters (Å^2 x 10^3) for jonap33.

	x	y	z	U(eq)
H1A	4459	5852	6081	25
H1B	2974	6526	6152	25
H3A	4113	8297	8220	21
H3B	2864	7245	8508	21
H4	6991	6794	9924	17
H5A	2226	4996	7641	21
H5B	3224	4227	6940	21
H6	6286	4652	9365	18
H8	6459	9817	10019	19
H9	7944	11701	9717	21
H10	9961	12618	8857	22
H11	11703	12194	7862	24
H12	11907	9941	7172	23
H13	10274	8106	7379	21
H17	4524	1976	8065	21
H18	5076	276	6841	22
H19	6691	-412	5587	23
H20	8643	184	4805	27
H21	9824	2476	5100	26
H22	9033	4173	6170	21
H1	5580(50)	7730(40)	6320(30)	25(10)

Table S-6. Torsion angles [°] for jonap33.

Co1-N1-C2-C1	-2.7(3)	C1-C2-C3-O2	-144.7(2)
Co1-N1-C2-C3	-125.83(19)	C1-C2-C5-O3	153.0(2)
Co1-N1-C2-C5	116.9(2)	C2-N1-C4-O2	28.1(3)
Co1-N1-C4-O2	161.56(16)	C2-N1-C4-C7	-93.6(2)
Co1-N1-C4-C7	39.9(2)	C2-N1-C6-O3	-21.7(3)
Co1-N1-C6-O3	-155.44(16)	C2-N1-C6-C16	98.2(2)
Co1-N1-C6-C16	-35.5(2)	C3-O2-C4-N1	-41.5(2)
Co1-N2-C7-C4	10.9(3)	C3-O2-C4-C7	79.4(3)
Co1-N2-C7-C8	-172.6(2)	C3-C2-C5-O3	-80.7(3)
Co1-N2-C14-C13	-10.6(4)	C4-O2-C3-C2	37.6(3)
Co1-N2-C14-C15	170.88(19)	C4-N1-C2-C1	118.4(2)
Co1-N3-C16-C6	-3.3(3)	C4-N1-C2-C3	-4.7(2)
Co1-N3-C16-C17	179.5(2)	C4-N1-C2-C5	-121.9(2)
Co1-N3-C23-C22	3.1(4)	C4-N1-C6-O3	89.6(2)
Co1-N3-C23-C24	-178.86(19)	C4-N1-C6-C16	-150.5(2)
O1-C1-C2-N1	-68.4(3)	C4-C7-C8-C9	176.2(2)
O1-C1-C2-C3	49.5(3)	C5-O3-C6-N1	41.5(2)
O1-C1-C2-C5	175.9(2)	C5-O3-C6-C16	-78.9(2)
O2-C4-C7-N2	-154.3(2)	C5-C2-C3-O2	90.4(3)
O2-C4-C7-C8	29.0(3)	C6-O3-C5-C2	-43.9(2)
O3-C6-C16-N3	145.5(2)	C6-N1-C2-C1	-124.6(2)
O3-C6-C16-C17	-37.1(3)	C6-N1-C2-C3	112.3(2)
N1-C2-C3-O2	-19.6(3)	C6-N1-C2-C5	-4.9(2)
N1-C2-C5-O3	29.3(2)	C6-N1-C4-O2	-83.1(2)
N1-C4-C7-N2	-35.6(3)	C6-N1-C4-C7	155.2(2)
N1-C4-C7-C8	147.8(2)	C6-C16-C17-C18	-177.6(2)
N1-C6-C16-N3	27.4(3)	C7-N2-C14-C13	174.1(2)
N1-C6-C16-C17	-155.2(2)	C7-N2-C14-C15	-4.4(4)
N2-C7-C8-C9	-0.2(4)	C7-C8-C9-C15	-2.0(4)
N2-C14-C15-C9	2.4(4)	C8-C9-C15-C10	-178.6(3)
N2-C14-C15-C10	-178.1(2)	C8-C9-C15-C14	0.9(4)
N3-C16-C17-C18	-0.3(4)	C10-C11-C12-C13	2.2(4)
N3-C23-C24-C18	-1.7(4)	C11-C10-C15-C9	176.9(3)
N3-C23-C24-C19	-178.5(2)	C11-C10-C15-C14	-2.6(4)

C11-C12-C13-C14	-1.5(4)	C17-C18-C24-C23	-1.5(4)
C12-C13-C14-N2	-179.9(3)	C19-C20-C21-C22	-0.3(4)
C12-C13-C14-C15	-1.3(4)	C20-C19-C24-C18	-175.5(3)
C13-C14-C15-C9	-176.2(2)	C20-C19-C24-C23	1.2(4)
C13-C14-C15-C10	3.3(4)	C20-C21-C22-C23	0.9(4)
C14-N2-C7-C4	-173.1(2)	C21-C22-C23-N3	177.5(3)
C14-N2-C7-C8	3.4(4)	C21-C22-C23-C24	-0.5(4)
C15-C10-C11-C12	-0.1(4)	C22-C23-C24-C18	176.3(2)
C16-N3-C23-C22	-174.2(2)	C22-C23-C24-C19	-0.5(4)
C16-N3-C23-C24	3.8(4)	C23-N3-C16-C6	174.4(2)
C16-C17-C18-C24	2.5(4)	C23-N3-C16-C17	-2.8(4)
C17-C18-C24-C19	175.3(3)	C24-C19-C20-C21	-0.8(4)

Table S-7. Hydrogen bonds and close contacts for jonap33 [Å and °].

D-H...A	d(D-H)	d(H...A)	d(D...A)	<(DHA)
O1-H1...Br1	0.77(4)	2.47(4)	3.213(3)	161(4)

REFERENCE NUMBER: jonap36

1d

CRYSTAL STRUCTURE REPORT

C_{24} H_{21} Br_2 N_3 Ni O_3

or

(κ^3-L^q)$NiBr_2$

Report prepared for:

R. Zhang, A. Panda, Prof. W. Jones

February 19, 2024

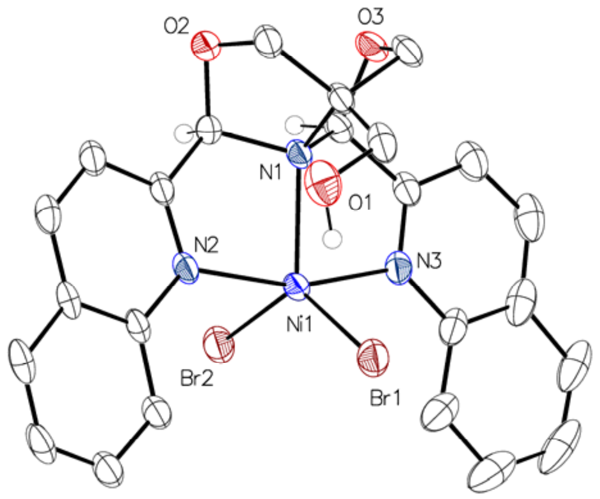

William W. Brennessel
X-ray Crystallographic Facility
Department of Chemistry, University of Rochester
120 Trustee Road
Rochester, NY 14627

Data collection

A crystal (0.116 x 0.045 x 0.045 mm^3) was placed onto a nylon loop and mounted on a Rigaku XtaLAB Synergy-S Dualflex diffractometer equipped with a HyPix-6000HE HPC area detector for data collection at 100.00(10) K. A preliminary set of cell constants and an orientation matrix were calculated from a small sampling of reflections.[1] A short pre-experiment was run, from which an optimal data collection strategy was determined. The full data collection was carried out using a PhotonJet (Cu) X-ray source with frame times of 0.27 and 1.06 seconds and a detector distance of 34.0 mm. Series of frames were collected in 0.50º steps in ω at different 2θ, κ, and ϕ settings. After the intensity data were corrected for absorption, the final cell constants were calculated from the xyz centroids of 17902 strong reflections from the actual data collection after integration.[1] See Table S-1 for additional crystal and refinement information.

Structure solution and refinement

The structure was solved using SHELXT[2] and refined using SHELXL.[3] The space group *P*-1 was determined based on intensity statistics. Most or all non-hydrogen atoms were assigned from the solution. Full-matrix least squares / difference Fourier cycles were performed which located any remaining non-hydrogen atoms. All non-hydrogen atoms were refined with anisotropic displacement parameters. All hydrogen atoms were placed in ideal positions and refined as riding atoms with relative isotropic displacement parameters. The final full matrix least squares refinement converged to $R1 = 0.0455$ (F^2, $I > 2\sigma(I)$) and $wR2 = 0.1174$ (F^2, all data).

Structure description

The structure is the one suggested. The asymmetric unit contains one molecule in a general position.

Structure manipulation and figure generation were performed using Olex2.[4] Unless noted otherwise all structural diagrams containing anisotropic displacement ellipsoids are drawn at the 50 % probability level.

Data collection, structure solution, and structure refinement were conducted at the X-ray Crystallographic Facility, B04 Hutchison Hall, Department of Chemistry, University of Rochester. The instrument was purchased with funding from NSF MRI program grant CHE-1725028. All publications arising from this report MUST either 1) include William W. Brennessel as a coauthor or 2) acknowledge William W. Brennessel and the X-ray Crystallographic Facility of the Department of Chemistry at the University of Rochester.

[1] *CrysAlisPro*, version 171.43.104a; Rigaku Corporation: Oxford, UK, 2024.

[2] Sheldrick, G. M. *SHELXT*, version 2018/2; *Acta. Crystallogr.* **2015**, *A71*, 3-8.

[3] Sheldrick, G. M. *SHELXL*, version 2019/2; *Acta. Crystallogr.* **2015**, *C71*, 3-8.

[4] Dolomanov, O. V.; Bourhis, L. J.; Gildea, R. J.; Howard, J. A. K.; Puschmann, H. *Olex2*, version 1.5; *J. Appl. Cryst.* **2009**, *42*, 339-341.

Some equations of interest:

$$R_{int} = \Sigma |F_o^2 - \langle F_o^2 \rangle| / \Sigma|F_o^2|$$

$$R1 = \Sigma||F_o|-|F_c|| / \Sigma|F_o|$$

$$wR2 = [\Sigma[w(F_o^2-F_c^2)^2] / \Sigma [w(F_o^2)^2]]^{1/2}$$

$$\text{where } w = 1 / [\sigma^2 (F_o^2) + (aP)^2 + bP] \text{ and}$$

$$P = 1/3 \max (0, F_o^2) + 2/3 F_c^2$$

$$GOF = S = [\Sigma[w(F_o^2-F_c^2)^2] / (m-n)]^{1/2}$$

where *m* = number of reflections and *n* = number of parameters

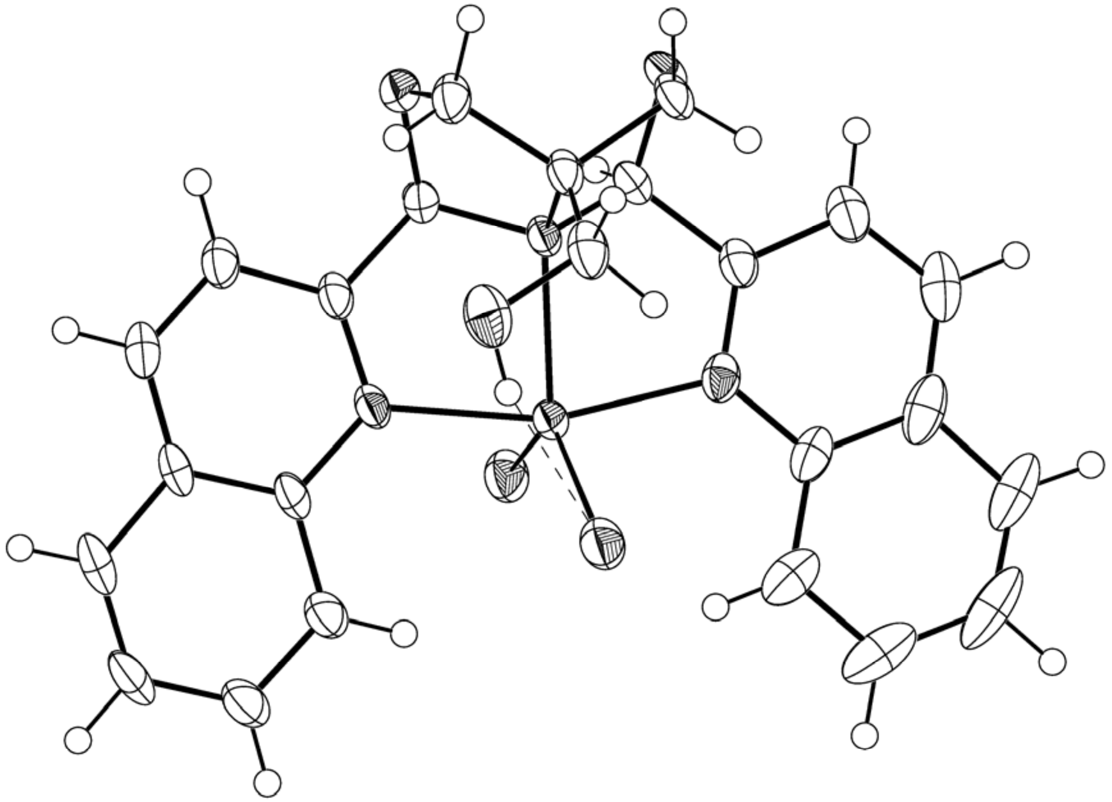

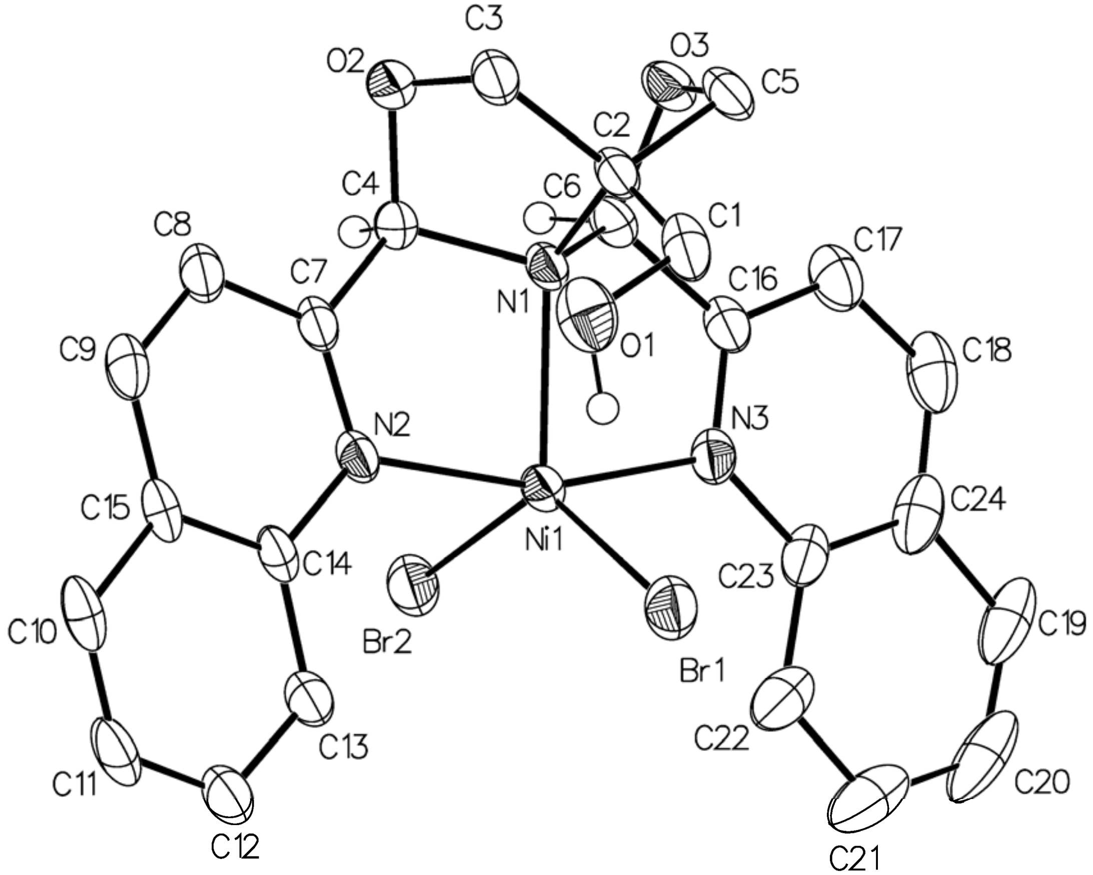

C3
O2
O3
C5
C2
C4
C6
C1
C8
C7
N1
C16
C17
C9
O1
C18
N2
N3
C15
C24
C14
Ni1
C23
C10
Br2
C19
Br1
C22
C13
C20
C11
C12
C21

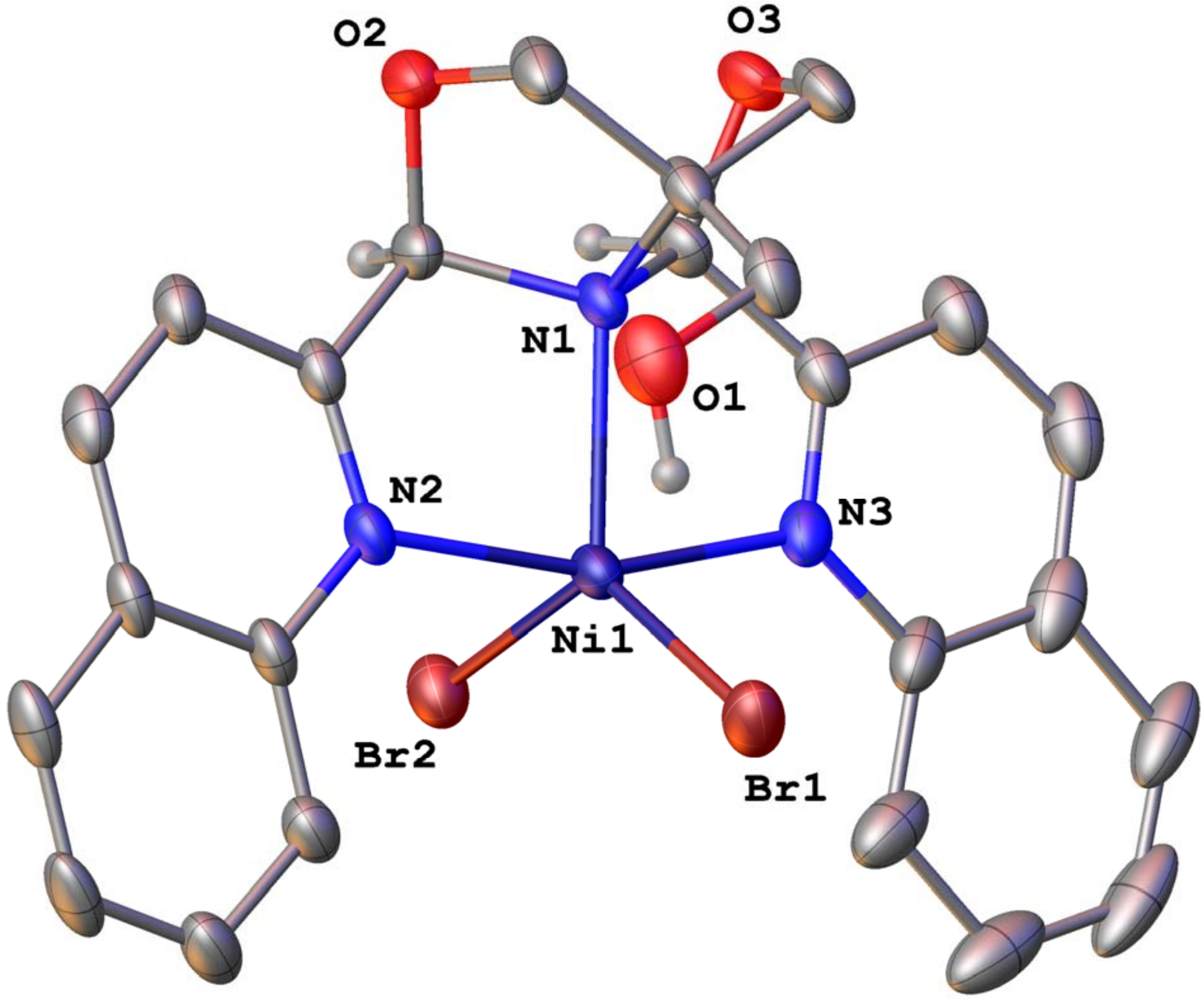
O2
O3
N1
O1
N2
N3
Ni1
Br2
Br1

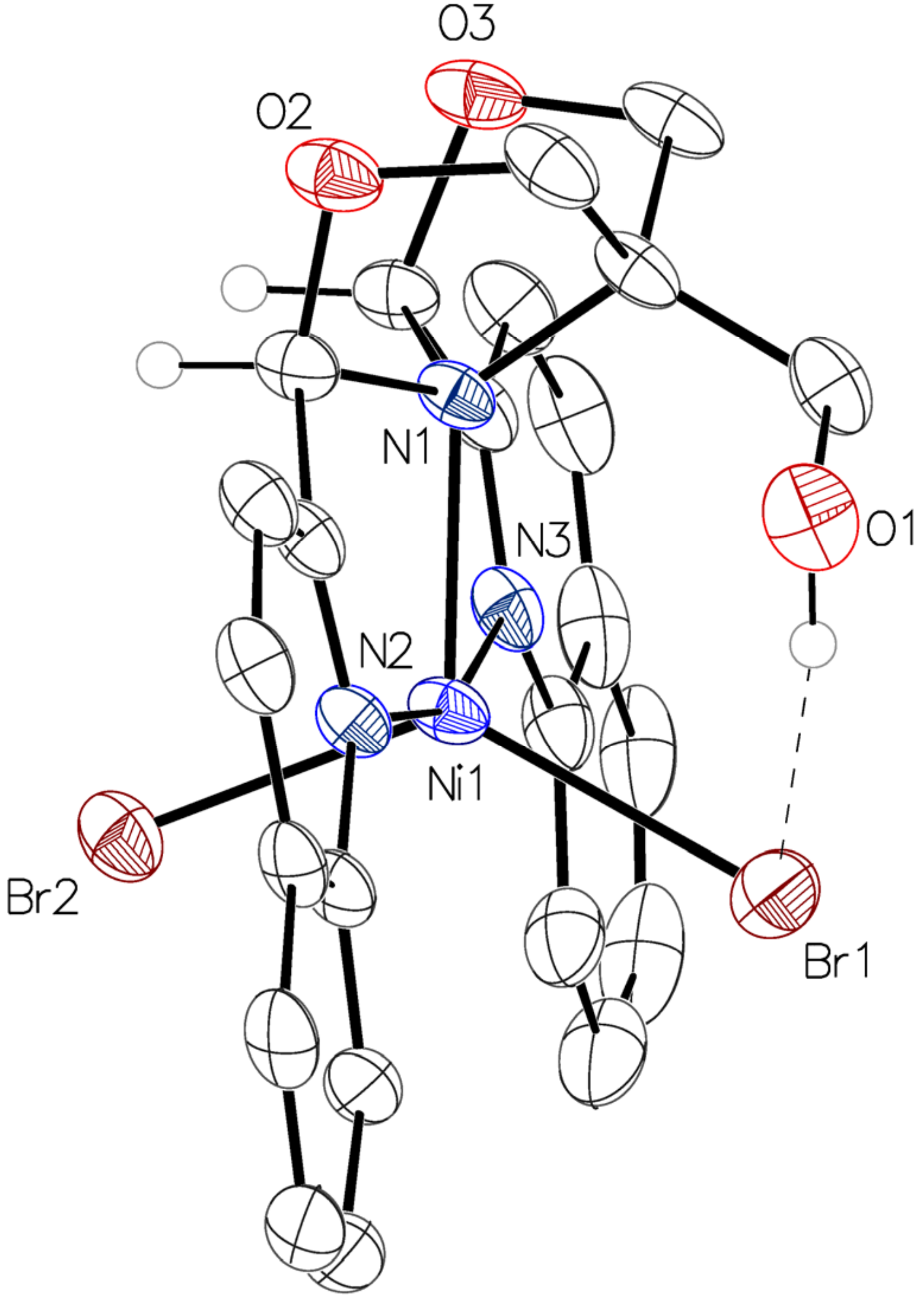
O3
O2
N1
O1
N3
N2
Ni1
Br2
Br1

Table S-1. Crystal data and structure refinement for jonap36.

Identification code	jonap36	
Empirical formula	C24 H21 Br2 N3 Ni O3	
Formula weight	617.97	
Temperature	100.00(10) K	
Wavelength	1.54184 Å	
Crystal system	triclinic	
Space group	*P*-1	
Unit cell dimensions	a = 9.09690(10) Å	α = 96.9440(10)°
	b = 10.00070(10) Å	β = 103.9700(10)°
	c = 12.6571(2) Å	γ = 97.4700(10)°
Volume	1093.98(2) $Å^3$	
Z	2	
Density (calculated)	1.876 Mg/m^3	
Absorption coefficient	5.843 mm^{-1}	
F(000)	616	
Crystal color, morphology	yellow-orange, block	
Crystal size	0.116 x 0.045 x 0.045 mm^3	
Theta range for data collection	3.645 to 79.944°	
Index ranges	$-9 \le h \le 11$, $-12 \le k \le 12$, $-15 \le l \le 16$	
Reflections collected	26989	
Independent reflections	4681 [R(int) = 0.0358]	
Observed reflections	4295	
Completeness to theta = 74.504°	99.7%	
Absorption correction	Multi-scan	
Max. and min. transmission	1.00000 and 0.86311	
Refinement method	Full-matrix least-squares on F^2	
Data / restraints / parameters	4681 / 0 / 298	
Goodness-of-fit on F^2	1.049	
Final R indices [I>2sigma(I)]	$R1$ = 0.0455, $wR2$ = 0.1149	
R indices (all data)	$R1$ = 0.0491, $wR2$ = 0.1174	
Largest diff. peak and hole	2.252 and -1.012 e.$Å^{-3}$	

Table S-2. Atomic coordinates (x 10^4) and equivalent isotropic displacement parameters (Å^{2}x 10^3) for jonap36. U_{eq} is defined as one third of the trace of the orthogonalized U_{ij} tensor.

	x	y	z	U_{eq}
Br1	7217(1)	6684(1)	5666(1)	37(1)
Br2	10046(1)	5808(1)	8813(1)	33(1)
Ni1	7708(1)	6198(1)	7585(1)	24(1)
O1	4717(4)	8036(3)	6587(3)	40(1)
O2	4983(3)	7435(3)	9576(2)	33(1)
O3	4026(3)	4396(3)	8586(2)	32(1)
N1	5800(3)	6100(3)	8263(2)	22(1)
N2	8094(3)	8210(3)	8347(3)	24(1)
N3	6962(3)	4113(3)	7198(3)	26(1)
C1	3925(5)	6713(4)	6577(4)	34(1)
C2	4179(4)	6320(4)	7715(3)	27(1)
C3	3893(4)	7451(4)	8552(4)	33(1)
C4	6312(4)	7162(4)	9269(3)	26(1)
C5	3206(4)	4925(4)	7661(4)	32(1)
C6	5576(4)	4698(4)	8557(3)	26(1)
C7	7208(4)	8423(4)	9016(3)	24(1)
C8	7150(4)	9733(4)	9554(3)	28(1)
C9	8060(4)	10837(4)	9373(3)	29(1)
C10	10097(5)	11754(4)	8533(3)	33(1)
C11	11124(5)	11504(4)	7945(3)	38(1)
C12	11177(5)	10161(5)	7489(3)	37(1)
C13	10168(4)	9076(4)	7598(3)	30(1)
C14	9086(4)	9306(4)	8209(3)	26(1)
C15	9071(4)	10651(3)	8704(3)	26(1)
C16	5942(4)	3666(4)	7720(3)	26(1)
C17	5269(5)	2284(4)	7584(4)	35(1)
C18	5683(5)	1347(4)	6892(4)	40(1)
C19	7407(6)	825(5)	5705(4)	48(1)
C20	8569(7)	1266(6)	5289(4)	58(2)
C21	9242(6)	2663(6)	5479(4)	53(1)
C22	8678(5)	3612(5)	6082(3)	40(1)
C23	7466(4)	3177(4)	6537(3)	32(1)
C24	6811(5)	1766(4)	6360(3)	39(1)

Table S-3. Bond lengths [Å] and angles [°] for jonap36.

Br(1)-Ni(1)	2.4804(8)	C(9)-H(9)	0.9500
Br(2)-Ni(1)	2.4203(7)	C(9)-C(15)	1.408(6)
Ni(1)-N(1)	2.110(3)	C(10)-H(10)	0.9500
Ni(1)-N(2)	2.070(3)	C(10)-C(11)	1.357(7)
Ni(1)-N(3)	2.067(3)	C(10)-C(15)	1.422(5)
O(1)-H(1)	0.9153	C(11)-H(11)	0.9500
O(1)-C(1)	1.419(5)	C(11)-C(12)	1.408(6)
O(2)-C(3)	1.432(5)	C(12)-H(12)	0.9500
O(2)-C(4)	1.405(4)	C(12)-C(13)	1.371(5)
O(3)-C(5)	1.427(5)	C(13)-H(13)	0.9500
O(3)-C(6)	1.413(4)	C(13)-C(14)	1.415(5)
N(1)-C(2)	1.527(4)	C(14)-C(15)	1.417(5)
N(1)-C(4)	1.491(4)	C(16)-C(17)	1.409(5)
N(1)-C(6)	1.495(4)	C(17)-H(17)	0.9500
N(2)-C(7)	1.320(5)	C(17)-C(18)	1.356(6)
N(2)-C(14)	1.383(4)	C(18)-H(18)	0.9500
N(3)-C(16)	1.325(5)	C(18)-C(24)	1.404(7)
N(3)-C(23)	1.377(5)	C(19)-H(19)	0.9500
C(1)-H(1A)	0.9900	C(19)-C(20)	1.338(9)
C(1)-H(1B)	0.9900	C(19)-C(24)	1.425(6)
C(1)-C(2)	1.511(6)	C(20)-H(20)	0.9500
C(2)-C(3)	1.546(5)	C(20)-C(21)	1.417(9)
C(2)-C(5)	1.536(5)	C(21)-H(21)	0.9500
C(3)-H(3A)	0.9900	C(21)-C(22)	1.373(6)
C(3)-H(3B)	0.9900	C(22)-H(22)	0.9500
C(4)-H(4)	1.0000	C(22)-C(23)	1.407(7)
C(4)-C(7)	1.521(5)	C(23)-C(24)	1.427(6)
C(5)-H(5A)	0.9900	Br(2)-Ni(1)-Br(1)	130.06(3)
C(5)-H(5B)	0.9900	N(1)-Ni(1)-Br(1)	115.54(9)
C(6)-H(6)	1.0000	N(1)-Ni(1)-Br(2)	114.41(9)
C(6)-C(16)	1.515(5)	N(2)-Ni(1)-Br(1)	96.52(9)
C(7)-C(8)	1.414(5)	N(2)-Ni(1)-Br(2)	90.40(9)
C(8)-H(8)	0.9500	N(2)-Ni(1)-N(1)	81.92(11)
C(8)-C(9)	1.369(5)	N(3)-Ni(1)-Br(1)	97.11(9)

N(3)-Ni(1)-Br(2)	89.70(9)	O(2)-C(4)-N(1)	106.9(3)
N(3)-Ni(1)-N(1)	81.72(12)	O(2)-C(4)-H(4)	109.4
N(3)-Ni(1)-N(2)	162.07(13)	O(2)-C(4)-C(7)	112.4(3)
C(1)-O(1)-H(1)	103.3	N(1)-C(4)-H(4)	109.4
C(4)-O(2)-C(3)	104.3(3)	N(1)-C(4)-C(7)	109.4(3)
C(6)-O(3)-C(5)	103.8(3)	C(7)-C(4)-H(4)	109.4
C(2)-N(1)-Ni(1)	127.4(2)	O(3)-C(5)-C(2)	103.9(3)
C(4)-N(1)-Ni(1)	104.1(2)	O(3)-C(5)-H(5A)	111.0
C(4)-N(1)-C(2)	104.2(3)	O(3)-C(5)-H(5B)	111.0
C(4)-N(1)-C(6)	111.2(3)	C(2)-C(5)-H(5A)	111.0
C(6)-N(1)-Ni(1)	105.4(2)	C(2)-C(5)-H(5B)	111.0
C(6)-N(1)-C(2)	104.1(3)	H(5A)-C(5)-H(5B)	109.0
C(7)-N(2)-Ni(1)	112.6(2)	O(3)-C(6)-N(1)	106.7(3)
C(7)-N(2)-C(14)	118.8(3)	O(3)-C(6)-H(6)	109.4
C(14)-N(2)-Ni(1)	128.6(3)	O(3)-C(6)-C(16)	111.1(3)
C(16)-N(3)-Ni(1)	113.4(2)	N(1)-C(6)-H(6)	109.4
C(16)-N(3)-C(23)	118.7(3)	N(1)-C(6)-C(16)	110.8(3)
C(23)-N(3)-Ni(1)	127.8(3)	C(16)-C(6)-H(6)	109.4
O(1)-C(1)-H(1A)	109.1	N(2)-C(7)-C(4)	116.6(3)
O(1)-C(1)-H(1B)	109.1	N(2)-C(7)-C(8)	123.4(3)
O(1)-C(1)-C(2)	112.5(3)	C(8)-C(7)-C(4)	119.8(3)
H(1A)-C(1)-H(1B)	107.8	C(7)-C(8)-H(8)	120.8
C(2)-C(1)-H(1A)	109.1	C(9)-C(8)-C(7)	118.4(4)
C(2)-C(1)-H(1B)	109.1	C(9)-C(8)-H(8)	120.8
N(1)-C(2)-C(3)	102.8(3)	C(8)-C(9)-H(9)	120.0
N(1)-C(2)-C(5)	102.7(3)	C(8)-C(9)-C(15)	120.1(3)
C(1)-C(2)-N(1)	116.4(3)	C(15)-C(9)-H(9)	120.0
C(1)-C(2)-C(3)	111.6(3)	C(11)-C(10)-H(10)	120.0
C(1)-C(2)-C(5)	110.0(3)	C(11)-C(10)-C(15)	119.9(4)
C(5)-C(2)-C(3)	113.0(3)	C(15)-C(10)-H(10)	120.0
O(2)-C(3)-C(2)	105.8(3)	C(10)-C(11)-H(11)	119.6
O(2)-C(3)-H(3A)	110.6	C(10)-C(11)-C(12)	120.7(4)
O(2)-C(3)-H(3B)	110.6	C(12)-C(11)-H(11)	119.6
C(2)-C(3)-H(3A)	110.6	C(11)-C(12)-H(12)	119.4
C(2)-C(3)-H(3B)	110.6	C(13)-C(12)-C(11)	121.1(4)
H(3A)-C(3)-H(3B)	108.7	C(13)-C(12)-H(12)	119.4

C(12)-C(13)-H(13)	120.3	C(20)-C(19)-H(19)	119.9
C(12)-C(13)-C(14)	119.4(4)	C(20)-C(19)-C(24)	120.2(5)
C(14)-C(13)-H(13)	120.3	C(24)-C(19)-H(19)	119.9
N(2)-C(14)-C(13)	119.6(3)	C(19)-C(20)-H(20)	119.1
N(2)-C(14)-C(15)	120.8(3)	C(19)-C(20)-C(21)	121.9(4)
C(13)-C(14)-C(15)	119.5(3)	C(21)-C(20)-H(20)	119.1
C(9)-C(15)-C(10)	122.4(4)	C(20)-C(21)-H(21)	120.0
C(9)-C(15)-C(14)	118.3(3)	C(22)-C(21)-C(20)	120.0(5)
C(14)-C(15)-C(10)	119.2(4)	C(22)-C(21)-H(21)	120.0
N(3)-C(16)-C(6)	117.2(3)	C(21)-C(22)-H(22)	120.3
N(3)-C(16)-C(17)	123.5(4)	C(21)-C(22)-C(23)	119.4(5)
C(17)-C(16)-C(6)	119.2(3)	C(23)-C(22)-H(22)	120.3
C(16)-C(17)-H(17)	120.5	N(3)-C(23)-C(22)	119.5(4)
C(18)-C(17)-C(16)	119.1(4)	N(3)-C(23)-C(24)	120.0(4)
C(18)-C(17)-H(17)	120.5	C(22)-C(23)-C(24)	120.4(4)
C(17)-C(18)-H(18)	120.3	C(18)-C(24)-C(19)	122.6(5)
C(17)-C(18)-C(24)	119.4(4)	C(18)-C(24)-C(23)	119.2(4)
C(24)-C(18)-H(18)	120.3	C(19)-C(24)-C(23)	118.1(5)

Table S-4. Anisotropic displacement parameters ($Å^2 x 10^3$) for jonap36. The anisotropic displacement factor exponent takes the form: $-2\pi^2[h^2 a^{*2}U_{11} + ... + 2 h k a^* b^* U_{12}]$

	U_{11}	U_{22}	U_{33}	U_{23}	U_{13}	U_{12}
Br1	43(1)	33(1)	31(1)	0(1)	10(1)	-7(1)
Br2	27(1)	31(1)	36(1)	-2(1)	3(1)	2(1)
Ni1	20(1)	20(1)	30(1)	-3(1)	8(1)	-2(1)
O1	42(2)	26(1)	48(2)	6(1)	4(1)	4(1)
O2	26(1)	30(1)	39(2)	-8(1)	14(1)	-3(1)
O3	26(1)	25(1)	45(2)	-1(1)	15(1)	-6(1)
N1	18(1)	16(1)	29(2)	-1(1)	5(1)	-1(1)
N2	20(1)	19(1)	28(2)	0(1)	3(1)	-3(1)
N3	21(1)	24(1)	28(2)	-4(1)	1(1)	5(1)
C1	27(2)	27(2)	39(2)	-3(2)	-2(2)	1(2)
C2	16(2)	21(2)	39(2)	-5(1)	4(1)	0(1)
C3	21(2)	28(2)	45(2)	-11(2)	6(2)	1(1)
C4	24(2)	21(2)	29(2)	-5(1)	9(1)	-2(1)
C5	18(2)	22(2)	52(2)	-4(2)	9(2)	-4(1)
C6	25(2)	20(2)	33(2)	2(1)	9(1)	-4(1)
C7	19(2)	20(2)	28(2)	-3(1)	0(1)	-1(1)
C8	23(2)	24(2)	32(2)	-5(1)	2(1)	1(1)
C9	28(2)	18(2)	34(2)	-4(1)	-1(2)	2(1)
C10	35(2)	20(2)	34(2)	4(2)	-6(2)	-9(2)
C11	36(2)	34(2)	32(2)	7(2)	0(2)	-20(2)
C12	32(2)	41(2)	31(2)	2(2)	6(2)	-13(2)
C13	26(2)	31(2)	28(2)	1(2)	5(1)	-6(2)
C14	22(2)	21(2)	28(2)	-1(1)	1(1)	-6(1)
C15	24(2)	18(2)	28(2)	1(1)	-3(1)	-5(1)
C16	20(2)	22(2)	33(2)	0(1)	1(1)	1(1)
C17	30(2)	24(2)	46(2)	0(2)	4(2)	1(2)
C18	38(2)	23(2)	47(2)	-3(2)	-7(2)	6(2)
C19	59(3)	47(3)	31(2)	-12(2)	-7(2)	33(2)
C20	79(4)	64(3)	30(2)	-9(2)	1(2)	53(3)
C21	53(3)	84(4)	31(2)	5(2)	13(2)	41(3)
C22	41(2)	54(3)	27(2)	-1(2)	7(2)	22(2)
C23	29(2)	33(2)	27(2)	-7(2)	-1(2)	11(2)
C24	43(2)	33(2)	32(2)	-7(2)	-7(2)	21(2)

Table S-5. Hydrogen coordinates (x 10^4) and isotropic displacement parameters (Å^2 x 10^3) for jonap36.

	x	y	z	U(eq)
H1	5474	7862	6252	61
H1A	4277	6037	6096	41
H1B	2812	6684	6260	41
H3A	4050	8352	8311	40
H3B	2831	7261	8629	40
H4	6987	6795	9875	31
H5A	2161	5029	7722	39
H5B	3125	4319	6963	39
H6	6262	4693	9303	32
H8	6495	9842	10029	34
H9	8008	11731	9699	35
H10	10064	12665	8832	40
H11	11814	12244	7839	46
H12	11926	10005	7099	44
H13	10194	8178	7266	36
H17	4536	2013	7971	42
H18	5215	416	6769	48
H19	6979	-117	5562	58
H20	8953	625	4854	69
H21	10083	2942	5190	64
H22	9103	4553	6190	48

Table S-6. Torsion angles [°] for jonap36.

Ni1-N1-C2-C1	-2.9(4)	C1-C2-C3-O2	-144.8(3)
Ni1-N1-C2-C3	-125.2(3)	C1-C2-C5-O3	153.8(3)
Ni1-N1-C2-C5	117.3(3)	C2-N1-C4-O2	27.6(4)
Ni1-N1-C4-O2	162.9(2)	C2-N1-C4-C7	-94.3(3)
Ni1-N1-C4-C7	41.0(3)	C2-N1-C6-O3	-20.9(4)
Ni1-N1-C6-O3	-156.9(2)	C2-N1-C6-C16	100.2(3)
Ni1-N1-C6-C16	-35.8(3)	C3-O2-C4-N1	-40.9(4)
Ni1-N2-C7-C4	10.8(4)	C3-O2-C4-C7	79.1(3)
Ni1-N2-C7-C8	-173.9(3)	C3-C2-C5-O3	-80.7(4)
Ni1-N2-C14-C13	-9.9(5)	C4-O2-C3-C2	37.2(4)
Ni1-N2-C14-C15	172.4(3)	C4-N1-C2-C1	117.9(3)
Ni1-N3-C16-C6	-4.3(4)	C4-N1-C2-C3	-4.4(4)
Ni1-N3-C16-C17	179.8(3)	C4-N1-C2-C5	-122.0(3)
Ni1-N3-C23-C22	3.1(5)	C4-N1-C6-O3	90.8(3)
Ni1-N3-C23-C24	-179.9(3)	C4-N1-C6-C16	-148.1(3)
O1-C1-C2-N1	-67.7(4)	C4-C7-C8-C9	175.1(3)
O1-C1-C2-C3	49.9(4)	C5-O3-C6-N1	40.6(3)
O1-C1-C2-C5	176.1(3)	C5-O3-C6-C16	-80.2(3)
O2-C4-C7-N2	-155.2(3)	C5-C2-C3-O2	90.6(4)
O2-C4-C7-C8	29.3(5)	C6-O3-C5-C2	-43.4(3)
O3-C6-C16-N3	146.8(3)	C6-N1-C2-C1	-125.5(3)
O3-C6-C16-C17	-37.1(5)	C6-N1-C2-C3	112.2(3)
N1-C2-C3-O2	-19.4(4)	C6-N1-C2-C5	-5.3(4)
N1-C2-C5-O3	29.3(4)	C6-N1-C4-O2	-84.0(3)
N1-C4-C7-N2	-36.6(4)	C6-N1-C4-C7	154.1(3)
N1-C4-C7-C8	147.9(3)	C6-C16-C17-C18	-175.3(4)
N1-C6-C16-N3	28.3(4)	C7-N2-C14-C13	172.8(3)
N1-C6-C16-C17	-155.6(3)	C7-N2-C14-C15	-4.9(5)
N2-C7-C8-C9	-0.1(6)	C7-C8-C9-C15	-2.7(6)
N2-C14-C15-C9	2.3(5)	C8-C9-C15-C10	-176.7(4)
N2-C14-C15-C10	-179.4(3)	C8-C9-C15-C14	1.6(5)
N3-C16-C17-C18	0.5(6)	C10-C11-C12-C13	2.0(7)
N3-C23-C24-C18	-1.8(6)	C11-C10-C15-C9	175.3(4)
N3-C23-C24-C19	-177.6(4)	C11-C10-C15-C14	-3.0(6)

C11-C12-C13-C14	-2.0(6)	C17-C18-C24-C23	-1.7(6)
C12-C13-C14-N2	-178.2(4)	C19-C20-C21-C22	-1.7(7)
C12-C13-C14-C15	-0.4(6)	C20-C19-C24-C18	-174.5(4)
C13-C14-C15-C9	-175.4(3)	C20-C19-C24-C23	1.2(6)
C13-C14-C15-C10	2.9(5)	C20-C21-C22-C23	2.1(7)
C14-N2-C7-C4	-171.4(3)	C21-C22-C23-N3	176.0(4)
C14-N2-C7-C8	3.9(5)	C21-C22-C23-C24	-1.0(6)
C15-C10-C11-C12	0.6(6)	C22-C23-C24-C18	175.2(4)
C16-N3-C23-C22	-172.5(4)	C22-C23-C24-C19	-0.7(6)
C16-N3-C23-C24	4.5(5)	C23-N3-C16-C6	171.9(3)
C16-C17-C18-C24	2.3(6)	C23-N3-C16-C17	-3.9(5)
C17-C18-C24-C19	174.0(4)	C24-C19-C20-C21	-0.1(7)

Table S-7. Hydrogen bonds and close contacts for jonap36 [Å and °].

D-H...A	d(D-H)	d(H...A)	d(D...A)	<(DHA)
O1-H1...Br1	0.92	2.30	3.171(3)	158.7

REFERENCE NUMBER: jonap28

2a

CRYSTAL STRUCTURE REPORT

C_{28} H_{24} F_6 Mn N_4 O_9 S_2

or

(κ^4-L^q)Mn$(OTf)_2$ · MeCN

Report prepared for:

R. Zhang, A. Panda, Prof. W. Jones

October 03, 2023

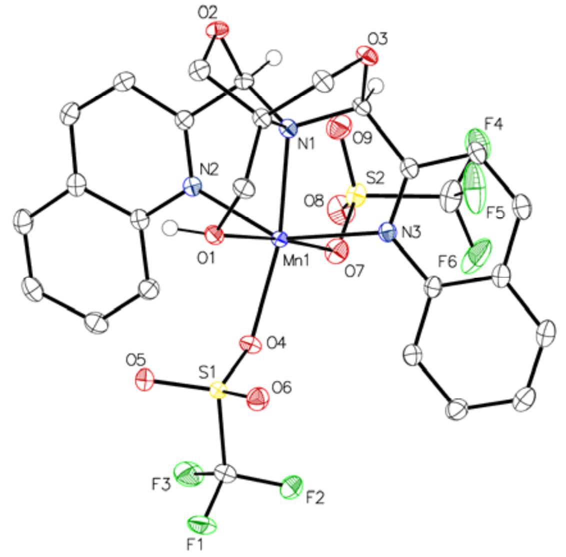

William W. Brennessel
X-ray Crystallographic Facility
Department of Chemistry, University of Rochester
120 Trustee Road
Rochester, NY 14627

Data collection

A crystal (0.115 x 0.089 x 0.073 mm^3) was placed onto a nylon loop and mounted on a Rigaku XtaLAB Synergy-S Dualflex diffractometer equipped with a HyPix-6000HE HPC area detector for data collection at 100.01(10) K. A preliminary set of cell constants and an orientation matrix were calculated from a small sampling of reflections.[1] A short pre-experiment was run, from which an optimal data collection strategy was determined. The full data collection was carried out using a PhotonJet (Cu) X-ray source with frame times of 0.09 and 0.37 seconds and a detector distance of 34.0 mm. Series of frames were collected in 0.50º steps in ω at different 2θ, κ, and ϕ settings. After the intensity data were corrected for absorption, the final cell constants were calculated from the xyz centroids of 35128 strong reflections from the actual data collection after integration.[1] See Table S-1 for additional crystal and refinement information.

Structure solution and refinement

The structure was solved using SHELXT[2] and refined using SHELXL.[3] The space group *P*-1 was determined based on intensity statistics. Most or all non-hydrogen atoms were assigned from the solution. Full-matrix least squares / difference Fourier cycles were performed which located any remaining non-hydrogen atoms. All non-hydrogen atoms were refined with anisotropic displacement parameters. The O-H hydrogen atom was found from the difference Fourier map and refined freely. All other hydrogen atoms were placed in ideal positions and refined as riding atoms with relative isotropic displacement parameters. The final full matrix least squares refinement converged to $R1 = 0.0267$ (F^2, $I > 2\sigma(I)$) and $wR2 = 0.0676$ (F^2, all data).

Structure description

The structure is the one suggested. The asymmetric unit contains one Mn complex and one acetonitrile solvent molecule of crystallization in general positions. Mn complexes are linked pairwise by O-H…O hydrogen bonding (see figure and Table S-7).

Structure manipulation and figure generation were performed using Olex2.[4] Unless noted otherwise all structural diagrams containing anisotropic displacement ellipsoids are drawn at the 50 % probability level.

Data collection, structure solution, and structure refinement were conducted at the X-ray Crystallographic Facility, B04 Hutchison Hall, Department of Chemistry, University of Rochester. The instrument was purchased with funding from NSF MRI program grant CHE-1725028. All publications arising from this report MUST either 1) include William W. Brennessel as a coauthor or 2) acknowledge William W. Brennessel and the X-ray Crystallographic Facility of the Department of Chemistry at the University of Rochester.

[1] *CrysAlisPro*, version 171.42.101a; Rigaku Corporation: Oxford, UK, 2023.

[2] Sheldrick, G. M. *SHELXT*, version 2018/2; *Acta. Crystallogr.* **2015**, *A71*, 3-8.

[3] Sheldrick, G. M. *SHELXL*, version 2019/2; *Acta. Crystallogr.* **2015**, *C71*, 3-8.

[4] Dolomanov, O. V.; Bourhis, L. J.; Gildea, R. J.; Howard, J. A. K.; Puschmann, H. *Olex2*, version 1.5; *J. Appl. Cryst.* **2009**, *42*, 339-341.

Some equations of interest:

$$R_{int} = \Sigma |F_o^2 - \langle F_o^2 \rangle| / \Sigma|F_o^2|$$

$$R1 = \Sigma||F_o|-|F_c|| / \Sigma|F_o|$$

$$wR2 = [\Sigma[w(F_o^2-F_c^2)^2] / \Sigma [w(F_o^2)^2]]^{1/2}$$

where $w = 1 / [\sigma^2 (F_o^2) + (aP)^2 + bP]$ and

$$P = 1/3 \max (0, F_o^2) + 2/3 F_c^2$$

$$\mathrm{GOF} = S = [\Sigma[w(F_o^2-F_c^2)^2] / (m-n)]^{1/2}$$

where m = number of reflections and n = number of parameters

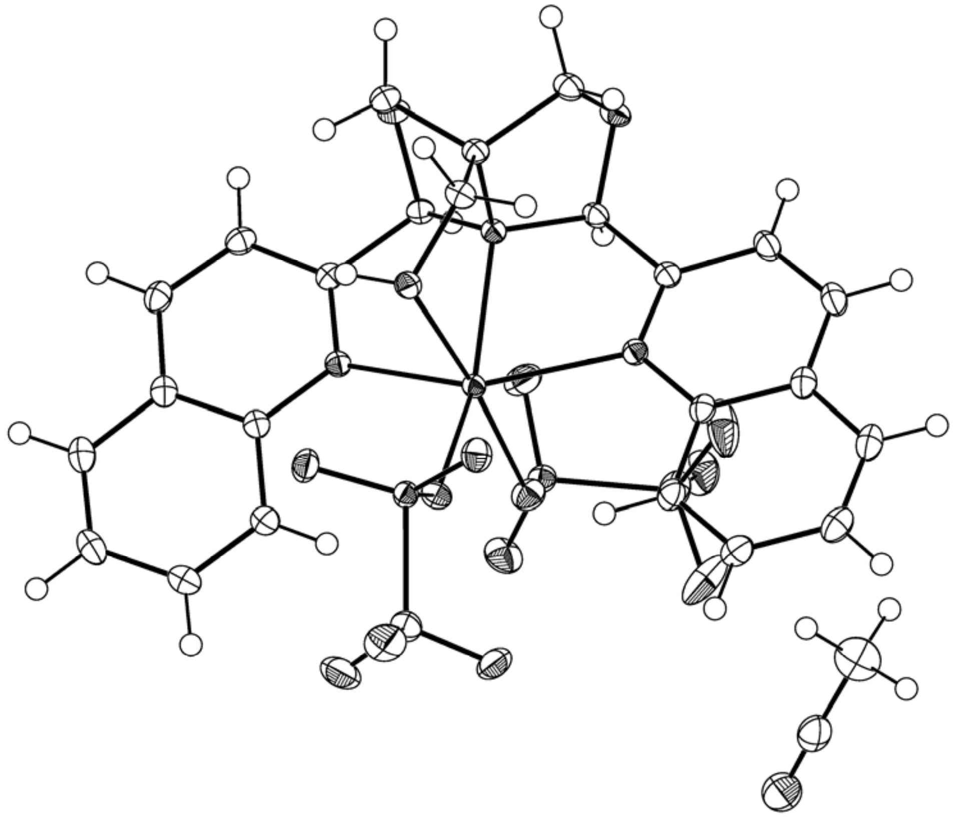

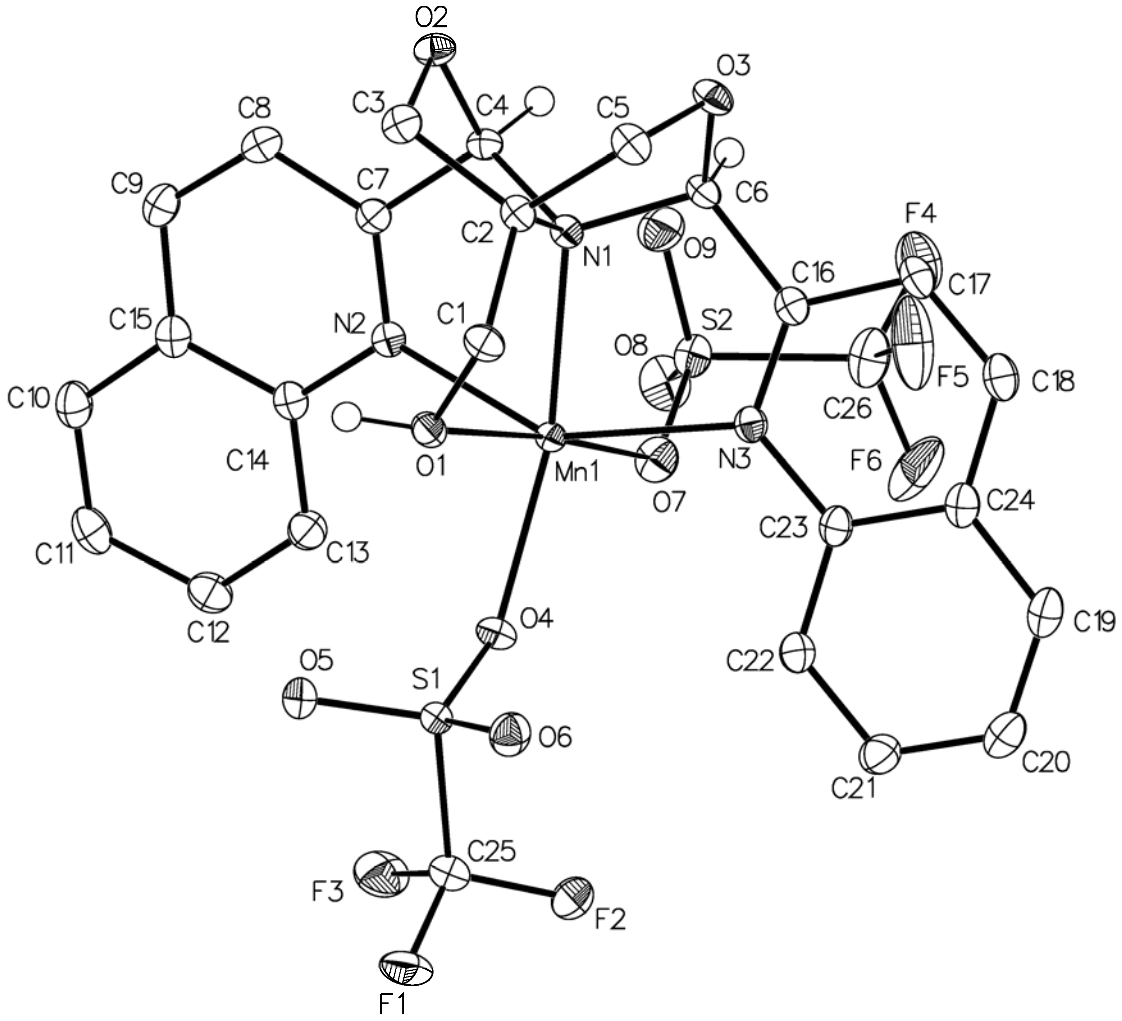
O2
O3
C3
C4
C5
C8
C9
C7
C6
C2
N1
F4
O9
C16
C17
C15
N2
C1
S2
O8
C10
F5
C18
C26
O1
Mn1
N3
C14
O7
F6
C24
C23
C11
C13
C19
C12
O4
C22
O5
S1
O6
C20
C21
C25
F3
F2
F1

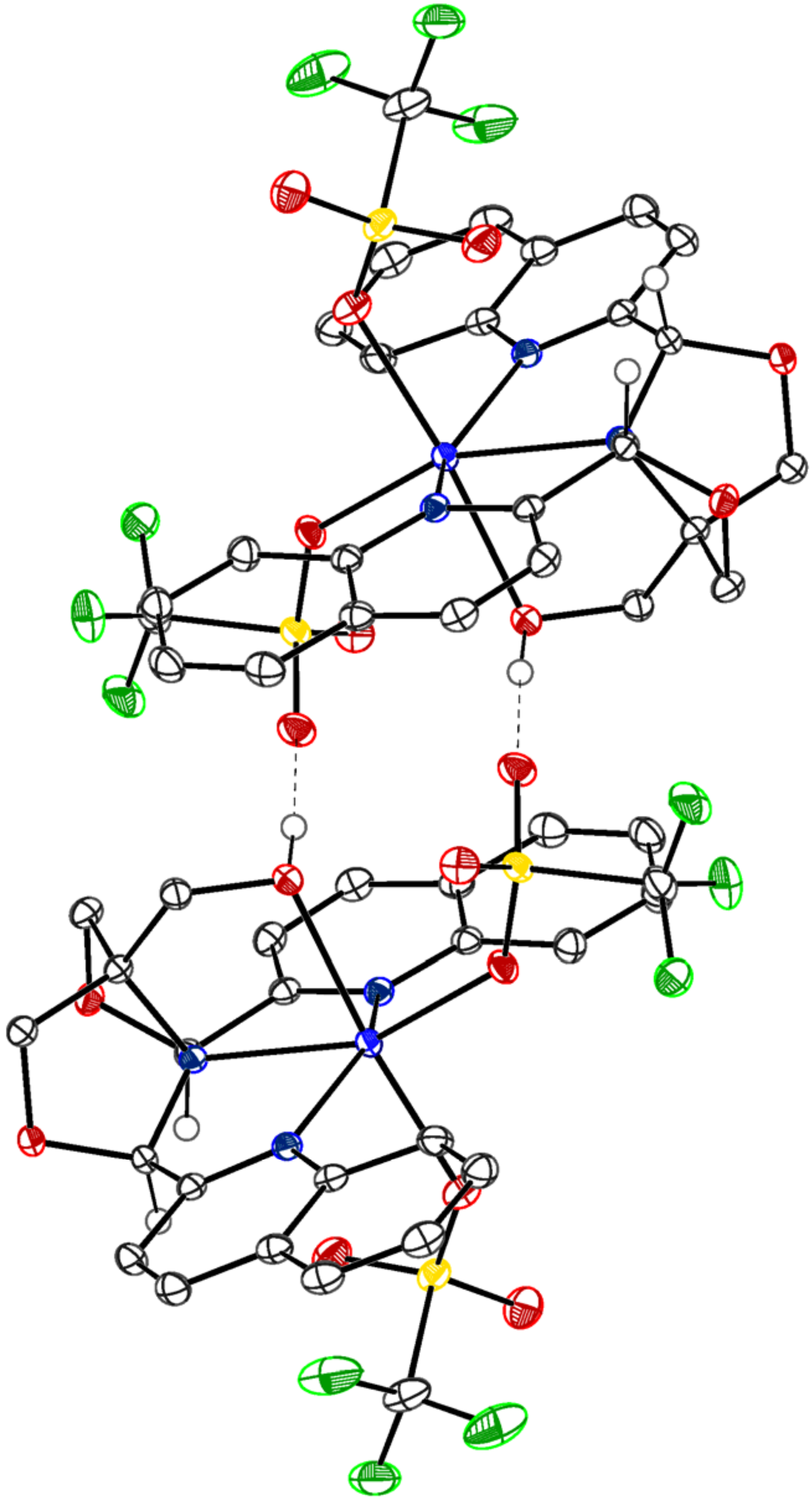

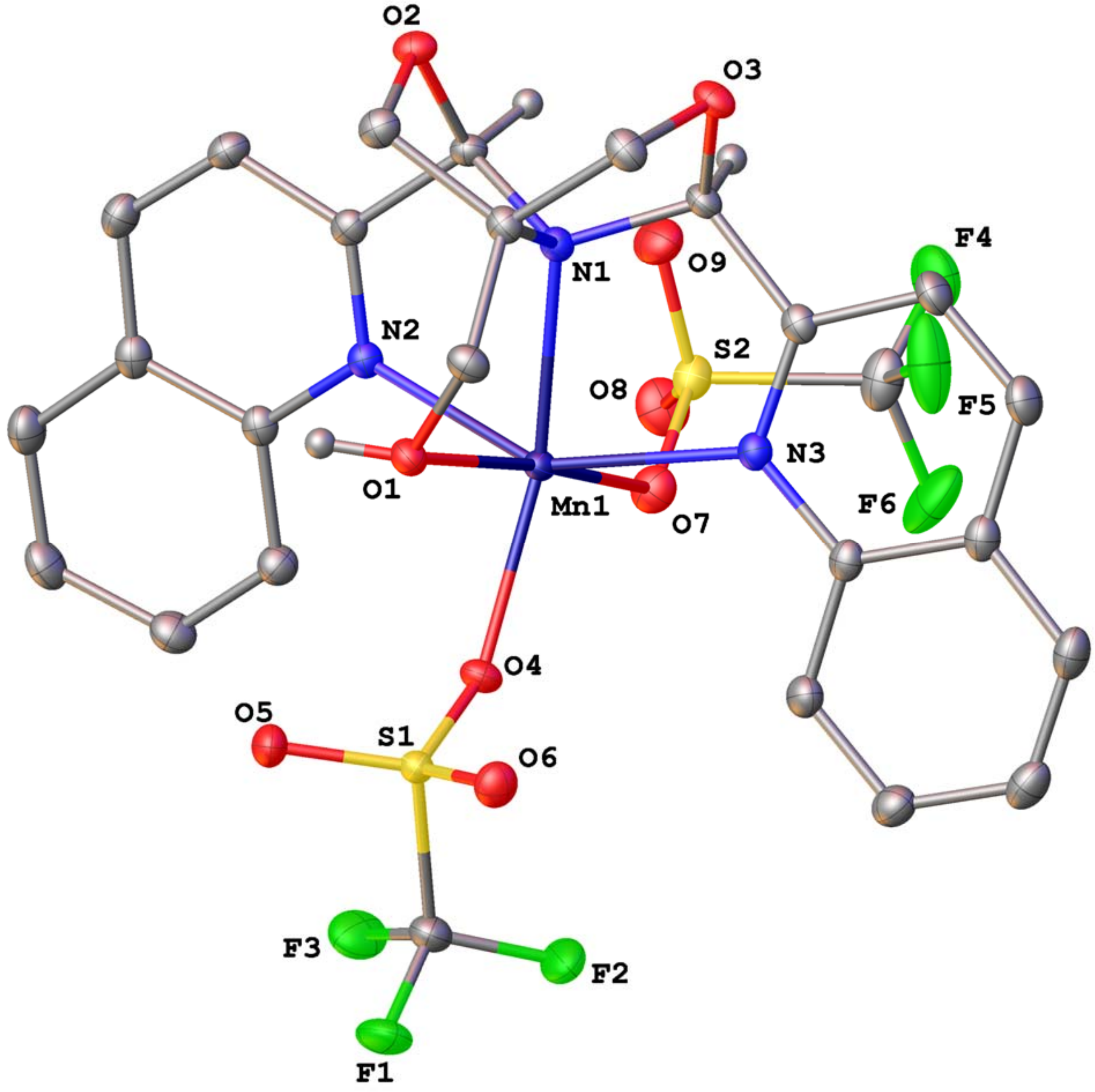
O2
O3
N1
O9
F4
N2
S2
O8
F5
N3
O1
Mn1
O7
F6
O4
O5
S1
O6
F3
F2
F1

Table S-1. Crystal data and structure refinement for jonap28.

Identification code	jonap28	
Empirical formula	C28 H24 F6 Mn N4 O9 S2	
Formula weight	793.57	
Temperature	100.01(10) K	
Wavelength	1.54184 Å	
Crystal system	triclinic	
Space group	*P*-1	
Unit cell dimensions	*a* = 10.31340(10) Å	α = 83.9410(10)°
	b = 10.54870(10) Å	β = 84.7860(10)°
	c = 14.46490(10) Å	γ = 81.0050(10)°
Volume	1541.21(2) $Å^3$	
Z	2	
Density (calculated)	1.710 Mg/m^3	
Absorption coefficient	5.643 mm^{-1}	
F(000)	806	
Crystal color, morphology	colourless, block	
Crystal size	0.115 x 0.089 x 0.073 mm^3	
Theta range for data collection	4.262 to 80.119°	
Index ranges	$-11 \leq h \leq 12$, $-13 \leq k \leq 13$, $-18 \leq l \leq 18$	
Reflections collected	52185	
Independent reflections	6635 [*R*(int) = 0.0365]	
Observed reflections	6444	
Completeness to theta = 74.504°	99.7%	
Absorption correction	Multi-scan	
Max. and min. transmission	1.00000 and 0.91521	
Refinement method	Full-matrix least-squares on F^2	
Data / restraints / parameters	6635 / 0 / 456	
Goodness-of-fit on F^2	1.052	
Final *R* indices [*I*>2sigma(*I*)]	*R*1 = 0.0267, *wR*2 = 0.0672	
R indices (all data)	*R*1 = 0.0274, *wR*2 = 0.0676	
Largest diff. peak and hole	0.328 and -0.422 e.$Å^{-3}$	

Table S-2. Atomic coordinates (x 10^4) and equivalent isotropic displacement parameters ($Å^2$x 10^3) for jonap28. U_{eq} is defined as one third of the trace of the orthogonalized U_{ij} tensor.

	x	y	z	U_{eq}
Mn1	1804(1)	3830(1)	7054(1)	11(1)
S1	1805(1)	2655(1)	5022(1)	13(1)
S2	4253(1)	4393(1)	8188(1)	16(1)
F1	2877(1)	1496(1)	3571(1)	30(1)
F2	4030(1)	1130(1)	4768(1)	28(1)
F3	3933(1)	2959(1)	3935(1)	30(1)
F4	5188(1)	3568(1)	9801(1)	32(1)
F5	3888(1)	2376(1)	9359(1)	46(1)
F6	5857(1)	2318(1)	8720(1)	42(1)
O1	-215(1)	3987(1)	6484(1)	15(1)
O2	-1018(1)	6502(1)	8487(1)	15(1)
O3	-668(1)	3842(1)	9636(1)	16(1)
O4	2457(1)	3099(1)	5762(1)	16(1)
O5	1116(1)	3683(1)	4440(1)	21(1)
O6	1154(1)	1563(1)	5308(1)	20(1)
O7	3834(1)	3702(1)	7467(1)	20(1)
O8	5402(1)	4985(1)	7906(1)	26(1)
O9	3184(1)	5141(1)	8687(1)	25(1)
N1	303(1)	4560(1)	8203(1)	12(1)
N2	1654(1)	5952(1)	6749(1)	13(1)
N3	1698(1)	2108(1)	8125(1)	13(1)
C1	-1190(1)	3700(1)	7213(1)	16(1)
C2	-1112(1)	4447(1)	8054(1)	13(1)
C3	-1753(1)	5859(1)	7936(1)	16(1)
C4	305(1)	5965(1)	8245(1)	12(1)
C5	-1607(1)	3746(1)	8983(1)	16(1)
C6	556(1)	3787(1)	9097(1)	13(1)
C7	799(1)	6627(1)	7314(1)	13(1)
C8	356(1)	7958(1)	7124(1)	16(1)
C9	787(2)	8583(1)	6306(1)	17(1)
C10	2217(2)	8497(1)	4832(1)	20(1)

C11	3148(2)	7814(2)	4268(1)	21(1)
C12	3609(2)	6506(2)	4535(1)	20(1)
C13	3127(1)	5900(1)	5349(1)	17(1)
C14	2152(1)	6582(1)	5939(1)	14(1)
C15	1708(1)	7905(1)	5683(1)	16(1)
C16	1116(1)	2385(1)	8946(1)	13(1)
C17	1037(2)	1459(1)	9725(1)	17(1)
C18	1571(2)	204(1)	9626(1)	18(1)
C19	2806(2)	-1420(1)	8626(1)	20(1)
C20	3455(2)	-1700(1)	7788(1)	21(1)
C21	3537(2)	-708(1)	7059(1)	20(1)
C22	2967(2)	538(1)	7173(1)	18(1)
C23	2278(1)	848(1)	8024(1)	14(1)
C24	2211(1)	-141(1)	8767(1)	16(1)
C25	3247(2)	2033(1)	4283(1)	20(1)
C26	4823(2)	3094(2)	9063(1)	25(1)
N4	8351(2)	488(1)	7991(1)	34(1)
C27	7463(2)	-29(2)	8201(1)	26(1)
C28	6342(2)	-691(2)	8493(1)	36(1)

Table S-3. Bond lengths [Å] and angles [°] for jonap28.

Mn(1)-O(1)	2.2841(10)	C(1)-H(1B)	0.9900
Mn(1)-O(4)	2.1098(10)	C(1)-C(2)	1.5317(18)
Mn(1)-O(7)	2.2089(11)	C(2)-C(3)	1.5314(19)
Mn(1)-N(1)	2.2771(11)	C(2)-C(5)	1.5471(19)
Mn(1)-N(2)	2.2196(11)	C(3)-H(3A)	0.9900
Mn(1)-N(3)	2.2699(11)	C(3)-H(3B)	0.9900
S(1)-O(4)	1.4669(10)	C(4)-H(4)	1.0000
S(1)-O(5)	1.4372(10)	C(4)-C(7)	1.5331(18)
S(1)-O(6)	1.4279(11)	C(5)-H(5A)	0.9900
S(1)-C(25)	1.8281(15)	C(5)-H(5B)	0.9900
S(2)-O(7)	1.4640(11)	C(6)-H(6)	1.0000
S(2)-O(8)	1.4318(11)	C(6)-C(16)	1.5315(19)
S(2)-O(9)	1.4361(11)	C(7)-C(8)	1.4128(19)
S(2)-C(26)	1.8280(16)	C(8)-H(8)	0.9500
F(1)-C(25)	1.3386(18)	C(8)-C(9)	1.365(2)
F(2)-C(25)	1.3315(17)	C(9)-H(9)	0.9500
F(3)-C(25)	1.3248(19)	C(9)-C(15)	1.411(2)
F(4)-C(26)	1.333(2)	C(10)-H(10)	0.9500
F(5)-C(26)	1.3310(19)	C(10)-C(11)	1.368(2)
F(6)-C(26)	1.330(2)	C(10)-C(15)	1.416(2)
O(1)-C(1)	1.4321(17)	C(11)-H(11)	0.9500
O(1)-H(1)	0.86(3)	C(11)-C(12)	1.412(2)
O(2)-C(3)	1.4338(16)	C(12)-H(12)	0.9500
O(2)-C(4)	1.4202(16)	C(12)-C(13)	1.370(2)
O(3)-C(5)	1.4345(17)	C(13)-H(13)	0.9500
O(3)-C(6)	1.4181(16)	C(13)-C(14)	1.416(2)
N(1)-C(2)	1.5189(17)	C(14)-C(15)	1.4206(19)
N(1)-C(4)	1.4904(17)	C(16)-C(17)	1.4157(19)
N(1)-C(6)	1.4753(16)	C(17)-H(17)	0.9500
N(2)-C(7)	1.3221(18)	C(17)-C(18)	1.369(2)
N(2)-C(14)	1.3821(18)	C(18)-H(18)	0.9500
N(3)-C(16)	1.3211(18)	C(18)-C(24)	1.408(2)
N(3)-C(23)	1.3863(17)	C(19)-H(19)	0.9500
C(1)-H(1A)	0.9900	C(19)-C(20)	1.367(2)

C(19)-C(24)	1.420(2)	O(8)-S(2)-O(9)	117.09(7)
C(20)-H(20)	0.9500	O(8)-S(2)-C(26)	103.59(7)
C(20)-C(21)	1.411(2)	O(9)-S(2)-O(7)	113.67(7)
C(21)-H(21)	0.9500	O(9)-S(2)-C(26)	103.09(8)
C(21)-C(22)	1.374(2)	Mn(1)-O(1)-H(1)	119.4(17)
C(22)-H(22)	0.9500	C(1)-O(1)-Mn(1)	110.93(8)
C(22)-C(23)	1.408(2)	C(1)-O(1)-H(1)	110.1(17)
C(23)-C(24)	1.4215(19)	C(4)-O(2)-C(3)	102.83(10)
N(4)-C(27)	1.140(2)	C(6)-O(3)-C(5)	104.72(10)
C(27)-C(28)	1.451(3)	S(1)-O(4)-Mn(1)	134.46(6)
C(28)-H(28A)	0.9800	S(2)-O(7)-Mn(1)	126.27(6)
C(28)-H(28B)	0.9800	C(2)-N(1)-Mn(1)	114.49(8)
C(28)-H(28C)	0.9800	C(4)-N(1)-Mn(1)	109.59(8)
O(4)-Mn(1)-O(1)	82.64(4)	C(4)-N(1)-C(2)	104.71(10)
O(4)-Mn(1)-O(7)	92.63(4)	C(6)-N(1)-Mn(1)	110.29(8)
O(4)-Mn(1)-N(1)	156.24(4)	C(6)-N(1)-C(2)	104.33(10)
O(4)-Mn(1)-N(2)	104.32(4)	C(6)-N(1)-C(4)	113.33(10)
O(4)-Mn(1)-N(3)	107.03(4)	C(7)-N(2)-Mn(1)	114.75(9)
O(7)-Mn(1)-O(1)	174.62(4)	C(7)-N(2)-C(14)	118.46(12)
O(7)-Mn(1)-N(1)	111.06(4)	C(14)-N(2)-Mn(1)	125.35(9)
O(7)-Mn(1)-N(2)	91.14(4)	C(16)-N(3)-Mn(1)	114.94(9)
O(7)-Mn(1)-N(3)	84.26(4)	C(16)-N(3)-C(23)	118.31(12)
N(1)-Mn(1)-O(1)	73.76(4)	C(23)-N(3)-Mn(1)	126.46(9)
N(2)-Mn(1)-O(1)	87.55(4)	O(1)-C(1)-H(1A)	109.5
N(2)-Mn(1)-N(1)	77.82(4)	O(1)-C(1)-H(1B)	109.5
N(2)-Mn(1)-N(3)	148.48(4)	O(1)-C(1)-C(2)	110.88(11)
N(3)-Mn(1)-O(1)	99.55(4)	H(1A)-C(1)-H(1B)	108.1
N(3)-Mn(1)-N(1)	74.89(4)	C(2)-C(1)-H(1A)	109.5
O(4)-S(1)-C(25)	99.84(7)	C(2)-C(1)-H(1B)	109.5
O(5)-S(1)-O(4)	113.75(6)	N(1)-C(2)-C(1)	111.47(11)
O(5)-S(1)-C(25)	104.33(7)	N(1)-C(2)-C(3)	102.41(10)
O(6)-S(1)-O(4)	114.52(6)	N(1)-C(2)-C(5)	103.63(10)
O(6)-S(1)-O(5)	117.04(7)	C(1)-C(2)-C(5)	112.56(11)
O(6)-S(1)-C(25)	104.64(7)	C(3)-C(2)-C(1)	113.94(11)
O(7)-S(2)-C(26)	103.01(7)	C(3)-C(2)-C(5)	111.88(11)
O(8)-S(2)-O(7)	114.00(7)	O(2)-C(3)-C(2)	104.04(10)

O(2)-C(3)-H(3A)	110.9	C(10)-C(11)-C(12)	119.90(14)
O(2)-C(3)-H(3B)	110.9	C(12)-C(11)-H(11)	120.1
C(2)-C(3)-H(3A)	110.9	C(11)-C(12)-H(12)	119.5
C(2)-C(3)-H(3B)	110.9	C(13)-C(12)-C(11)	121.01(14)
H(3A)-C(3)-H(3B)	109.0	C(13)-C(12)-H(12)	119.5
O(2)-C(4)-N(1)	106.25(10)	C(12)-C(13)-H(13)	119.9
O(2)-C(4)-H(4)	109.4	C(12)-C(13)-C(14)	120.18(13)
O(2)-C(4)-C(7)	110.03(11)	C(14)-C(13)-H(13)	119.9
N(1)-C(4)-H(4)	109.4	N(2)-C(14)-C(13)	119.64(12)
N(1)-C(4)-C(7)	112.34(10)	N(2)-C(14)-C(15)	121.39(12)
C(7)-C(4)-H(4)	109.4	C(13)-C(14)-C(15)	118.97(13)
O(3)-C(5)-C(2)	105.53(11)	C(9)-C(15)-C(10)	122.72(13)
O(3)-C(5)-H(5A)	110.6	C(9)-C(15)-C(14)	117.98(13)
O(3)-C(5)-H(5B)	110.6	C(10)-C(15)-C(14)	119.29(13)
C(2)-C(5)-H(5A)	110.6	N(3)-C(16)-C(6)	119.31(12)
C(2)-C(5)-H(5B)	110.6	N(3)-C(16)-C(17)	123.43(13)
H(5A)-C(5)-H(5B)	108.8	C(17)-C(16)-C(6)	117.18(12)
O(3)-C(6)-N(1)	106.80(10)	C(16)-C(17)-H(17)	120.6
O(3)-C(6)-H(6)	109.5	C(18)-C(17)-C(16)	118.84(13)
O(3)-C(6)-C(16)	110.08(11)	C(18)-C(17)-H(17)	120.6
N(1)-C(6)-H(6)	109.5	C(17)-C(18)-H(18)	120.0
N(1)-C(6)-C(16)	111.32(10)	C(17)-C(18)-C(24)	119.99(13)
C(16)-C(6)-H(6)	109.5	C(24)-C(18)-H(18)	120.0
N(2)-C(7)-C(4)	119.16(12)	C(20)-C(19)-H(19)	119.7
N(2)-C(7)-C(8)	123.17(13)	C(20)-C(19)-C(24)	120.56(14)
C(8)-C(7)-C(4)	117.60(12)	C(24)-C(19)-H(19)	119.7
C(7)-C(8)-H(8)	120.4	C(19)-C(20)-H(20)	120.2
C(9)-C(8)-C(7)	119.22(13)	C(19)-C(20)-C(21)	119.69(14)
C(9)-C(8)-H(8)	120.4	C(21)-C(20)-H(20)	120.2
C(8)-C(9)-H(9)	120.2	C(20)-C(21)-H(21)	119.4
C(8)-C(9)-C(15)	119.66(13)	C(22)-C(21)-C(20)	121.18(14)
C(15)-C(9)-H(9)	120.2	C(22)-C(21)-H(21)	119.4
C(11)-C(10)-H(10)	119.7	C(21)-C(22)-H(22)	119.9
C(11)-C(10)-C(15)	120.63(14)	C(21)-C(22)-C(23)	120.21(14)
C(15)-C(10)-H(10)	119.7	C(23)-C(22)-H(22)	119.9
C(10)-C(11)-H(11)	120.1	N(3)-C(23)-C(22)	119.57(12)

N(3)-C(23)-C(24)	121.45(13)	F(5)-C(26)-S(2)	111.68(11)
C(22)-C(23)-C(24)	118.98(13)	F(5)-C(26)-F(4)	107.93(15)
C(18)-C(24)-C(19)	122.69(13)	F(6)-C(26)-S(2)	111.08(12)
C(18)-C(24)-C(23)	117.94(13)	F(6)-C(26)-F(4)	107.56(13)
C(19)-C(24)-C(23)	119.36(13)	F(6)-C(26)-F(5)	107.60(14)
F(1)-C(25)-S(1)	110.23(11)	N(4)-C(27)-C(28)	178.47(19)
F(2)-C(25)-S(1)	110.36(10)	C(27)-C(28)-H(28A)	109.5
F(2)-C(25)-F(1)	107.77(12)	C(27)-C(28)-H(28B)	109.5
F(3)-C(25)-S(1)	111.65(10)	C(27)-C(28)-H(28C)	109.5
F(3)-C(25)-F(1)	108.11(12)	H(28A)-C(28)-H(28B)	109.5
F(3)-C(25)-F(2)	108.61(13)	H(28A)-C(28)-H(28C)	109.5
F(4)-C(26)-S(2)	110.82(11)	H(28B)-C(28)-H(28C)	109.5

Table S-4. Anisotropic displacement parameters ($Å^2x\ 10^3$) for jonap28. The anisotropic displacement factor exponent takes the form: $-2\pi^2[\ h^2\ a^{*2}U_{11} + ... + 2\ h\ k\ a^*\ b^*\ U_{12}\ ]$

	U_{11}	U_{22}	U_{33}	U_{23}	U_{13}	U_{12}
Mn1	12(1)	11(1)	10(1)	-1(1)	0(1)	-1(1)
S1	16(1)	13(1)	11(1)	-1(1)	-1(1)	0(1)
S2	12(1)	16(1)	20(1)	1(1)	-2(1)	-3(1)
F1	37(1)	33(1)	18(1)	-13(1)	-2(1)	4(1)
F2	24(1)	30(1)	23(1)	-2(1)	-1(1)	12(1)
F3	30(1)	32(1)	26(1)	-2(1)	11(1)	-7(1)
F4	31(1)	40(1)	29(1)	3(1)	-14(1)	-7(1)
F5	39(1)	48(1)	54(1)	31(1)	-27(1)	-28(1)
F6	34(1)	26(1)	65(1)	-8(1)	-21(1)	12(1)
O1	15(1)	17(1)	12(1)	-1(1)	-1(1)	-1(1)
O2	12(1)	17(1)	17(1)	-5(1)	0(1)	-2(1)
O3	13(1)	22(1)	11(1)	-2(1)	1(1)	-4(1)
O4	18(1)	18(1)	12(1)	-4(1)	1(1)	-3(1)
O5	24(1)	18(1)	16(1)	1(1)	-3(1)	3(1)
O6	22(1)	16(1)	22(1)	-1(1)	-3(1)	-4(1)
O7	16(1)	21(1)	24(1)	-2(1)	-4(1)	-3(1)
O8	21(1)	30(1)	29(1)	1(1)	-2(1)	-13(1)
O9	19(1)	28(1)	26(1)	-5(1)	-3(1)	4(1)
N1	11(1)	13(1)	11(1)	-1(1)	-1(1)	-3(1)
N2	12(1)	13(1)	13(1)	-1(1)	-2(1)	-3(1)
N3	12(1)	14(1)	13(1)	0(1)	-2(1)	-2(1)
C1	15(1)	19(1)	14(1)	-3(1)	-1(1)	-5(1)
C2	10(1)	16(1)	13(1)	-1(1)	-2(1)	-3(1)
C3	13(1)	17(1)	18(1)	-3(1)	-3(1)	-2(1)
C4	12(1)	13(1)	12(1)	-3(1)	-1(1)	-2(1)
C5	13(1)	22(1)	15(1)	-1(1)	-1(1)	-5(1)
C6	12(1)	17(1)	10(1)	-1(1)	0(1)	-3(1)
C7	12(1)	14(1)	14(1)	-2(1)	-3(1)	-2(1)
C8	15(1)	14(1)	19(1)	-4(1)	-1(1)	-1(1)
C9	18(1)	13(1)	22(1)	0(1)	-4(1)	-2(1)
C10	24(1)	16(1)	20(1)	3(1)	-4(1)	-5(1)

C11	24(1)	24(1)	15(1)	3(1)	-1(1)	-8(1)
C12	18(1)	23(1)	17(1)	-2(1)	2(1)	-5(1)
C13	16(1)	17(1)	17(1)	-1(1)	0(1)	-3(1)
C14	13(1)	15(1)	14(1)	-1(1)	-3(1)	-4(1)
C15	16(1)	15(1)	17(1)	1(1)	-4(1)	-5(1)
C16	11(1)	16(1)	13(1)	0(1)	-2(1)	-3(1)
C17	18(1)	20(1)	13(1)	2(1)	-1(1)	-4(1)
C18	19(1)	19(1)	17(1)	4(1)	-3(1)	-6(1)
C19	21(1)	15(1)	25(1)	3(1)	-7(1)	-3(1)
C20	20(1)	15(1)	29(1)	-3(1)	-7(1)	-1(1)
C21	19(1)	20(1)	21(1)	-4(1)	-3(1)	1(1)
C22	18(1)	18(1)	16(1)	-1(1)	-4(1)	0(1)
C23	13(1)	14(1)	16(1)	0(1)	-4(1)	-2(1)
C24	16(1)	15(1)	19(1)	2(1)	-5(1)	-4(1)
C25	24(1)	21(1)	14(1)	-3(1)	1(1)	0(1)
C26	19(1)	23(1)	34(1)	4(1)	-12(1)	-6(1)
N4	39(1)	25(1)	35(1)	-4(1)	6(1)	1(1)
C27	34(1)	22(1)	21(1)	-5(1)	-1(1)	5(1)
C28	38(1)	38(1)	34(1)	-8(1)	-2(1)	-9(1)

Table S-5. Hydrogen coordinates (x 10^4) and isotropic displacement parameters ($Å^2$ x 10^3) for jonap28.

	x	y	z	U(eq)
H1A	-1055	2764	7408	19
H1B	-2076	3932	6978	19
H3A	-1684	6208	7274	19
H3B	-2694	5953	8168	19
H4	873	6091	8743	15
H5A	-2494	4165	9197	20
H5B	-1648	2832	8907	20
H6	1189	4171	9427	15
H8	-236	8413	7560	19
H9	469	9469	6156	21
H10	1909	9376	4650	24
H11	3484	8219	3698	25
H12	4263	6040	4144	24
H13	3448	5019	5518	20
H17	620	1704	10305	21
H18	1510	-434	10137	22
H19	2752	-2087	9117	24
H20	3848	-2558	7697	25
H21	3995	-906	6480	24
H22	3037	1193	6675	21
H28A	5621	-351	8097	54
H28B	6054	-554	9144	54
H28C	6595	-1616	8435	54
H1	-510(30)	4670(30)	6139(18)	50(7)

Table S-6. Torsion angles [°] for jonap28.

Mn1-O1-C1-C2	48.50(12)	O7-S2-C26-F4	-176.99(11)
Mn1-N1-C2-C1	7.23(13)	O7-S2-C26-F5	-56.63(14)
Mn1-N1-C2-C3	-114.98(9)	O7-S2-C26-F6	63.50(12)
Mn1-N1-C2-C5	128.53(9)	O8-S2-O7-Mn1	-133.46(8)
Mn1-N1-C4-O2	145.05(8)	O8-S2-C26-F4	63.97(13)
Mn1-N1-C4-C7	24.68(12)	O8-S2-C26-F5	-175.66(13)
Mn1-N1-C6-O3	-152.79(8)	O8-S2-C26-F6	-55.53(13)
Mn1-N1-C6-C16	-32.61(12)	O9-S2-O7-Mn1	4.23(10)
Mn1-N2-C7-C4	16.94(15)	O9-S2-C26-F4	-58.52(13)
Mn1-N2-C7-C8	-166.16(11)	O9-S2-C26-F5	61.85(14)
Mn1-N2-C14-C13	-18.22(18)	O9-S2-C26-F6	-178.02(11)
Mn1-N2-C14-C15	161.96(10)	N1-C2-C3-O2	-29.90(13)
Mn1-N3-C16-C6	1.29(15)	N1-C2-C5-O3	15.48(13)
Mn1-N3-C16-C17	-175.31(11)	N1-C4-C7-N2	-29.02(17)
Mn1-N3-C23-C22	-3.01(18)	N1-C4-C7-C8	153.91(12)
Mn1-N3-C23-C24	176.07(10)	N1-C6-C16-N3	21.72(17)
O1-C1-C2-N1	-36.42(15)	N1-C6-C16-C17	-161.47(12)
O1-C1-C2-C3	78.87(14)	N2-C7-C8-C9	2.3(2)
O1-C1-C2-C5	-152.37(11)	N2-C14-C15-C9	3.1(2)
O2-C4-C7-N2	-147.17(12)	N2-C14-C15-C10	-177.99(13)
O2-C4-C7-C8	35.76(16)	N3-C16-C17-C18	-0.9(2)
O3-C6-C16-N3	139.95(12)	N3-C23-C24-C18	-2.0(2)
O3-C6-C16-C17	-43.24(16)	N3-C23-C24-C19	179.13(13)
O4-S1-C25-F1	176.36(10)	C1-C2-C3-O2	-150.41(11)
O4-S1-C25-F2	57.43(12)	C1-C2-C5-O3	136.05(12)
O4-S1-C25-F3	-63.47(11)	C2-N1-C4-O2	21.79(12)
O5-S1-O4-Mn1	76.26(10)	C2-N1-C4-C7	-98.57(12)
O5-S1-C25-F1	-65.85(12)	C2-N1-C6-O3	-29.39(13)
O5-S1-C25-F2	175.23(10)	C2-N1-C6-C16	90.80(12)
O5-S1-C25-F3	54.32(12)	C3-O2-C4-N1	-41.51(12)
O6-S1-O4-Mn1	-62.04(10)	C3-O2-C4-C7	80.34(12)
O6-S1-C25-F1	57.63(12)	C3-C2-C5-O3	-94.13(13)
O6-S1-C25-F2	-61.30(12)	C4-O2-C3-C2	44.32(12)
O6-S1-C25-F3	177.80(10)	C4-N1-C2-C1	127.26(11)

C4-N1-C2-C3	5.05(12)	C12-C13-C14-C15	-1.5(2)
C4-N1-C2-C5	-111.43(11)	C13-C14-C15-C9	-176.73(13)
C4-N1-C6-O3	83.92(13)	C13-C14-C15-C10	2.2(2)
C4-N1-C6-C16	-155.90(11)	C14-N2-C7-C4	-176.06(12)
C4-C7-C8-C9	179.23(13)	C14-N2-C7-C8	0.8(2)
C5-O3-C6-N1	40.25(13)	C15-C10-C11-C12	0.0(2)
C5-O3-C6-C16	-80.73(12)	C16-N3-C23-C22	-176.56(13)
C5-C2-C3-O2	80.48(13)	C16-N3-C23-C24	2.52(19)
C6-O3-C5-C2	-34.00(13)	C16-C17-C18-C24	1.4(2)
C6-N1-C2-C1	-113.41(12)	C17-C18-C24-C19	178.82(14)
C6-N1-C2-C3	124.38(11)	C17-C18-C24-C23	0.0(2)
C6-N1-C2-C5	7.89(13)	C19-C20-C21-C22	-0.5(2)
C6-N1-C4-O2	-91.28(12)	C20-C19-C24-C18	-177.81(14)
C6-N1-C4-C7	148.35(11)	C20-C19-C24-C23	1.0(2)
C6-C16-C17-C18	-177.59(13)	C20-C21-C22-C23	-0.3(2)
C7-N2-C14-C13	176.28(13)	C21-C22-C23-N3	-179.42(13)
C7-N2-C14-C15	-3.54(19)	C21-C22-C23-C24	1.5(2)
C7-C8-C9-C15	-2.7(2)	C22-C23-C24-C18	177.05(13)
C8-C9-C15-C10	-178.79(14)	C22-C23-C24-C19	-1.8(2)
C8-C9-C15-C14	0.1(2)	C23-N3-C16-C6	175.57(12)
C10-C11-C12-C13	0.8(2)	C23-N3-C16-C17	-1.0(2)
C11-C10-C15-C9	177.38(14)	C24-C19-C20-C21	0.2(2)
C11-C10-C15-C14	-1.5(2)	C25-S1-O4-Mn1	-173.21(8)
C11-C12-C13-C14	0.0(2)	C26-S2-O7-Mn1	115.02(8)
C12-C13-C14-N2	178.73(13)		

Table S-7. Hydrogen bonds and close contacts for jonap28 [Å and °].

D-H...A	d(D-H)	d(H...A)	d(D...A)	<(DHA)
O1-H1...O5#1	0.86(3)	1.89(3)	2.7400(14)	170(2)

Symmetry transformations used to generate equivalent atoms:

#1 -x,-y+1,-z+1

REFERENCE NUMBER: jonap25

2b

CRYSTAL STRUCTURE REPORT

C_{30} H_{29} F_6 Fe N_4 $O_{9.5}$ S_2

or

[(κ^4-L^q)Fe(OTf)](NCMe)[OTf] · ½ Et_2O

Report prepared for:

R. Zhang, A. Panda, Prof. W. Jones

September 27, 2023

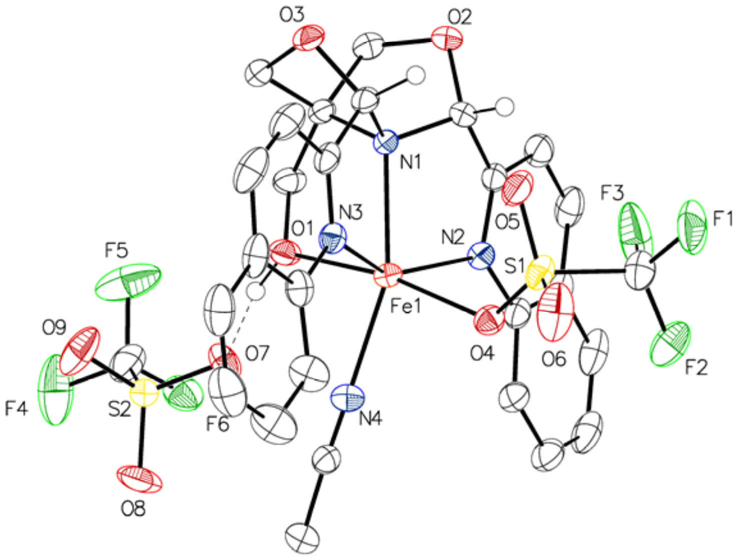

William W. Brennessel

X-ray Crystallographic Facility

Department of Chemistry, University of Rochester

120 Trustee Road

Rochester, NY 14627

Data collection

A crystal (0.209 x 0.182 x 0.141 mm^3) was placed onto a nylon loop and mounted on a Rigaku XtaLAB Synergy-S Dualflex diffractometer equipped with a HyPix-6000HE HPC area detector for data collection at 100.01(10) K. A preliminary set of cell constants and an orientation matrix were calculated from a small sampling of reflections.[1] A short pre-experiment was run, from which an optimal data collection strategy was determined. The full data collection was carried out using a PhotonJet (Cu) X-ray source with frame times of 0.11 and 0.43 seconds and a detector distance of 34.0 mm. Series of frames were collected in 0.50º steps in ω at different 2θ, κ, and ϕ settings. After the intensity data were corrected for absorption, the final cell constants were calculated from the xyz centroids of 33556 strong reflections from the actual data collection after integration.[1] See Table S-1 for additional crystal and refinement information.

Structure solution and refinement

The structure was solved using SHELXT[2] and refined using SHELXL.[3] The space group *C*2/*c* was determined based on systematic absences and intensity statistics. Most or all non-hydrogen atoms were assigned from the solution. Full-matrix least squares / difference Fourier cycles were performed which located any remaining non-hydrogen atoms. All non-hydrogen atoms were refined with anisotropic displacement parameters. The O-H hydrogen atom was found from the difference Fourier map and refined freely. All other hydrogen atoms were placed in ideal positions and refined as riding atoms with relative isotropic displacement parameters. The final full matrix least squares refinement converged to $R1 = 0.0339$ (F^2, $I > 2\sigma(I)$) and $wR2 = 0.0890$ (F^2, all data).

Structure description

The structure is the one suggested. The asymmetric unit contains one monocationic iron complex and one triflate anion in general positions and one-half of a diethyl ether solvent molecule on a crystallographic two-fold axis. The triflate counterion is modeled as disordered over three positions (0.56:0.33:11). The solvent is modeled as disordered over the two-fold axis (0.50:0.50).

Structure manipulation and figure generation were performed using Olex2.[4] Unless noted otherwise all structural diagrams containing anisotropic displacement ellipsoids are drawn at the 50 % probability level.

Data collection, structure solution, and structure refinement were conducted at the X-ray Crystallographic Facility, B04 Hutchison Hall, Department of Chemistry, University of Rochester. The instrument was purchased with funding from NSF MRI program grant CHE-1725028. All publications arising from this report MUST either 1) include William W. Brennessel as a coauthor or 2) acknowledge William W. Brennessel and the X-ray Crystallographic Facility of the Department of Chemistry at the University of Rochester.

1 *CrysAlisPro*, version 171.42.101a; Rigaku Corporation: Oxford, UK, 2023.

2 Sheldrick, G. M. *SHELXT*, version 2018/2; *Acta. Crystallogr.* **2015**, *A71*, 3-8.

3 Sheldrick, G. M. *SHELXL*, version 2019/2; *Acta. Crystallogr.* **2015**, *C71*, 3-8.

4 Dolomanov, O. V.; Bourhis, L. J.; Gildea, R. J.; Howard, J. A. K.; Puschmann, H. *Olex2*, version 1.5; *J. Appl. Cryst.* **2009**, *42*, 339-341.

Some equations of interest:

$$R_{\mathrm{int}} = \Sigma\,|F_o^2 - \langle F_o^2 \rangle| \,/\, \Sigma|F_o^2|$$

$$R1 = \Sigma||F_o|-|F_c|| \,/\, \Sigma|F_o|$$

$$wR2 = [\Sigma[w(F_o^2-F_c^2)^2] \,/\, \Sigma\,[w(F_o^2)^2]]^{1/2}$$

$$\text{where } w = 1 \,/\, [\sigma^2(F_o^2) + (aP)^2 + bP] \text{ and}$$

$$P = 1/3 \max(0, F_o^2) + 2/3\, F_c^2$$

$$\mathrm{GOF} = S = [\Sigma[w(F_o^2-F_c^2)^2] \,/\, (m-n)]^{1/2}$$

where m = number of reflections and n = number of parameters

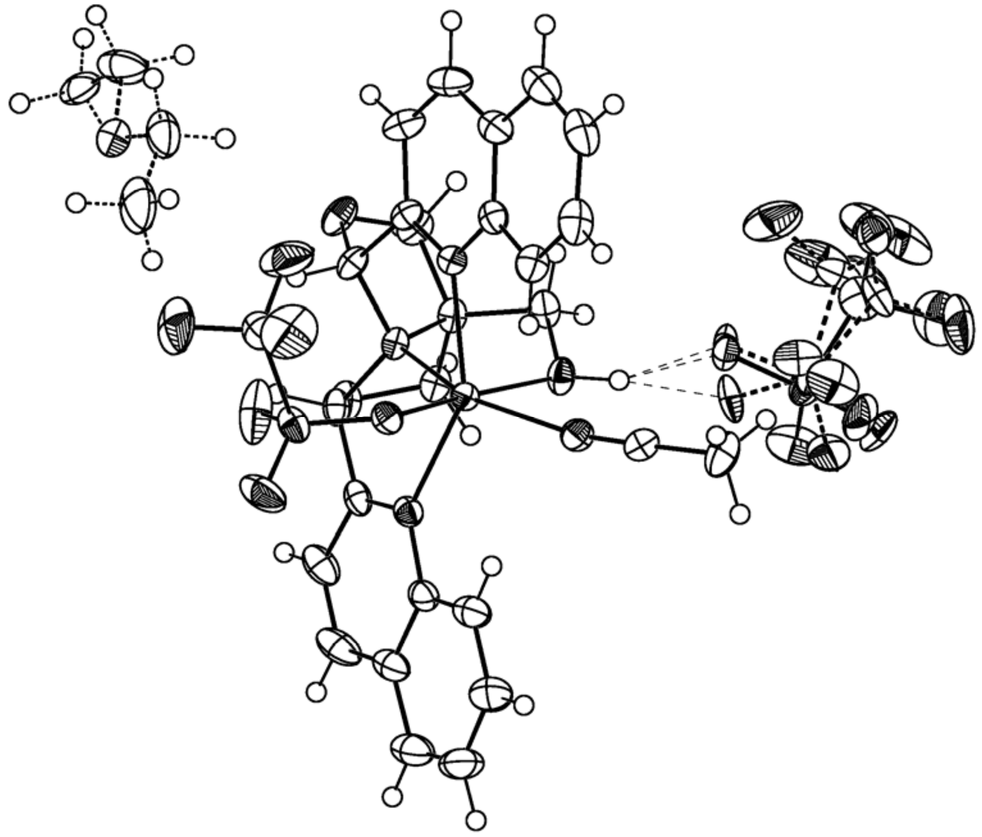

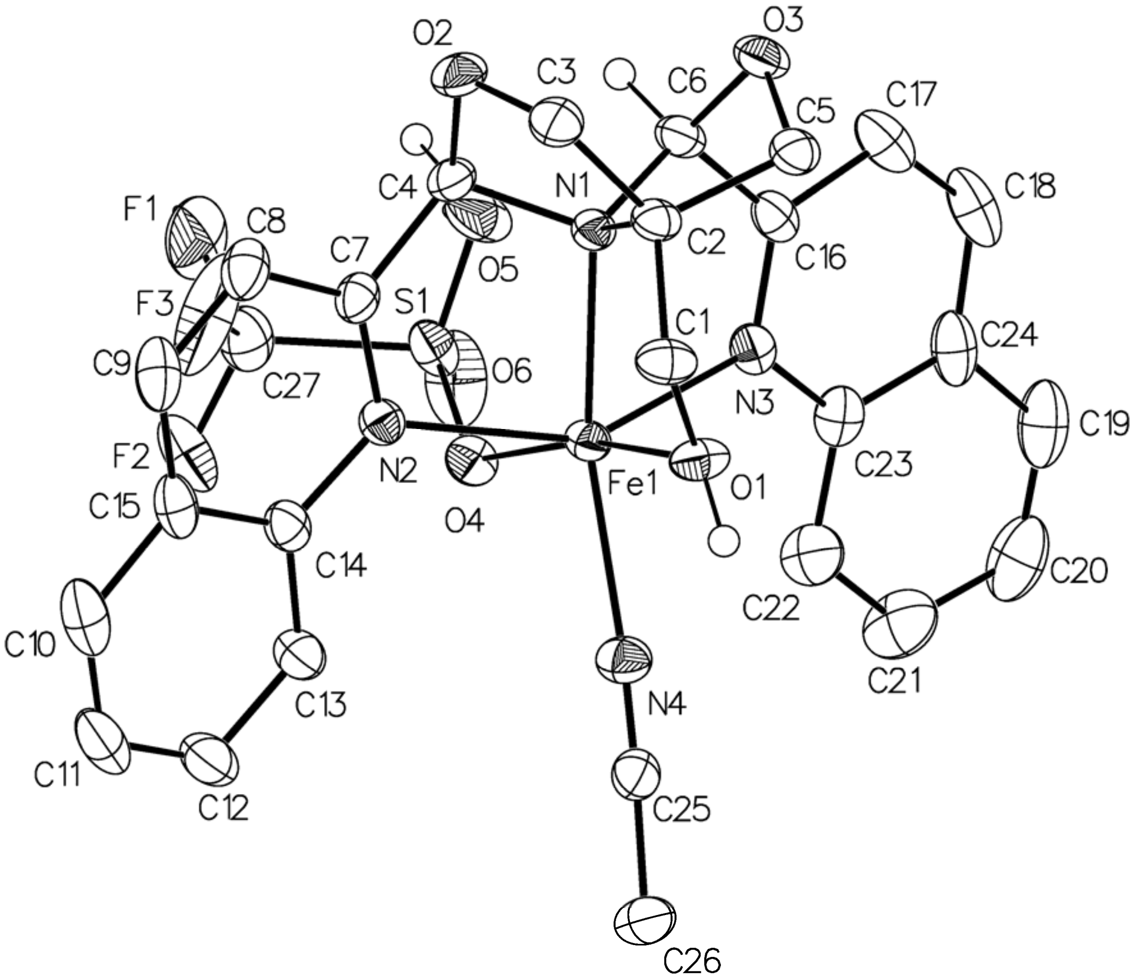
O2
O3
C3
C6
C5
C17
C4
N1
C18
F1
C8
C2
C16
C7
O5
S1
F3
C1
C24
C9
O6
N3
C27
C19
N2
Fe1
F2
O1
C23
C15
O4
C14
C20
C22
C10
C21
N4
C13
C11
C12
C25
C26

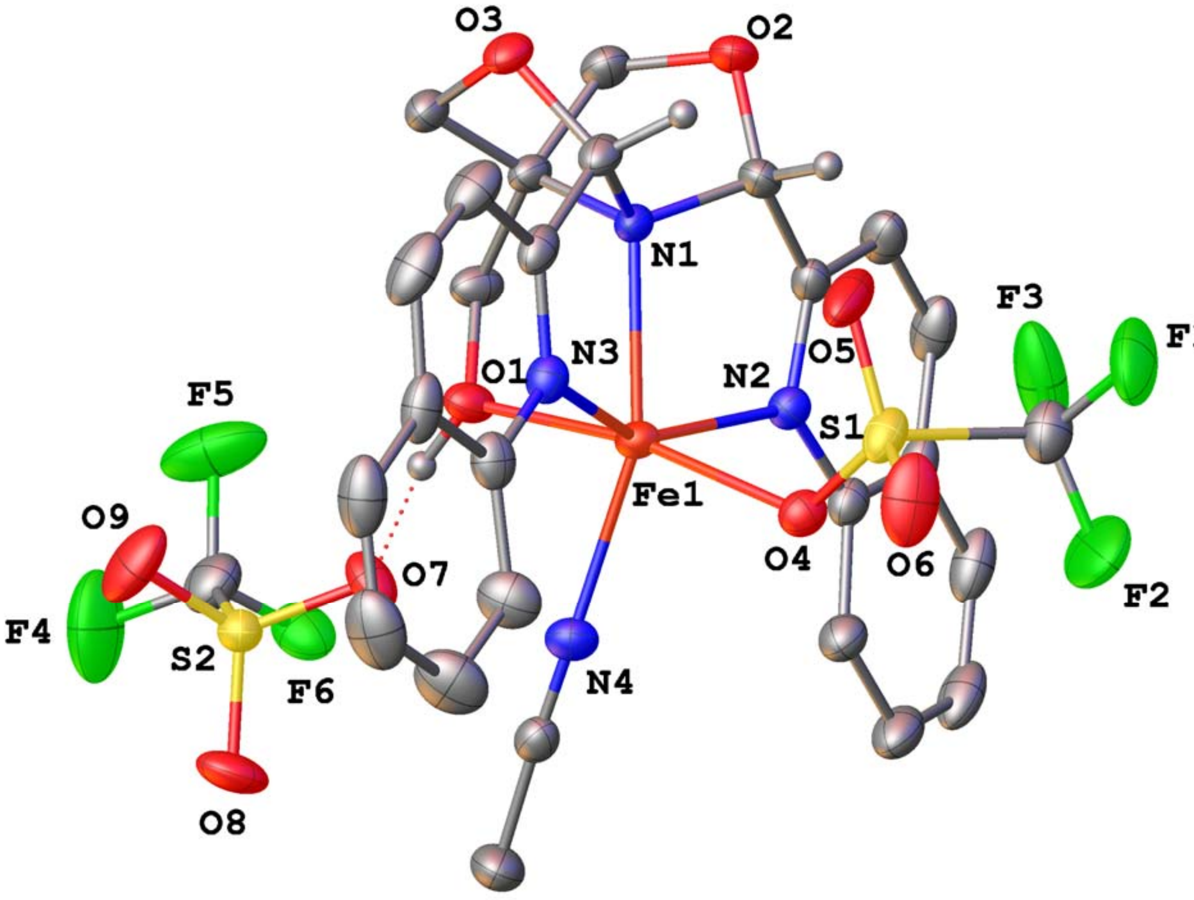
O3
O2
N1
F3
F1
O5
N3
O1
N2
F5
S1
Fe1
O9
O7
O4
O6
F2
F4
S2
F6
N4
O8

Table S-1. Crystal data and structure refinement for jonap25.

Identification code	jonap25	
Empirical formula	C30 H29 F6 Fe N4 O9.50 S2	
Formula weight	831.54	
Temperature	100.01(10) K	
Wavelength	1.54184 Å	
Crystal system	monoclinic	
Space group	*C*2/*c*	
Unit cell dimensions	*a* = 33.8633(2) Å	α = 90°
	b = 10.60010(10) Å	β = 95.5490(10)°
	c = 19.21380(10) Å	γ = 90°
Volume	6864.56(9) $Å^3$	
Z	8	
Density (calculated)	1.609 Mg/m^3	
Absorption coefficient	5.504 mm^{-1}	
F(000)	3400	
Crystal color, morphology	yellow, block	
Crystal size	0.209 x 0.182 x 0.141 mm^3	
Theta range for data collection	4.372 to 80.384°	
Index ranges	$-42 \le h \le 43$, $-13 \le k \le 13$, $-20 \le l \le 24$	
Reflections collected	58765	
Independent reflections	7376 [*R*(int) = 0.0370]	
Observed reflections	6916	
Completeness to theta = 74.504°	99.7%	
Absorption correction	Multi-scan	
Max. and min. transmission	1.00000 and 0.87540	
Refinement method	Full-matrix least-squares on F^2	
Data / restraints / parameters	7376 / 366 / 650	
Goodness-of-fit on F^2	1.061	
Final *R* indices [*I*>2sigma(*I*)]	*R*1 = 0.0339, *wR*2 = 0.0877	
R indices (all data)	*R*1 = 0.0362, *wR*2 = 0.0890	
Largest diff. peak and hole	0.733 and -0.505 e.$Å^{-3}$	

Table S-2. Atomic coordinates (x 10^4) and equivalent isotropic displacement parameters ($Å^2$x 10^3) for jonap25. U_{eq} is defined as one third of the trace of the orthogonalized U_{ij} tensor.

	x	y	z	U_{eq}
Fe1	3625(1)	4108(1)	3315(1)	19(1)
S1	2949(1)	5402(1)	4155(1)	27(1)
F1	2579(1)	4815(2)	5237(1)	61(1)
F2	2348(1)	3926(2)	4300(1)	68(1)
F3	2892(1)	3286(2)	4833(1)	76(1)
O1	4151(1)	3399(1)	2920(1)	26(1)
O2	4290(1)	3043(1)	5211(1)	31(1)
O3	4594(1)	5543(1)	4564(1)	28(1)
O4	3042(1)	4673(1)	3547(1)	24(1)
O5	3291(1)	5696(2)	4622(1)	54(1)
O6	2671(1)	6392(2)	3988(1)	52(1)
N1	4075(1)	4166(1)	4216(1)	20(1)
N2	3491(1)	2376(1)	3886(1)	21(1)
N3	3782(1)	6110(1)	3336(1)	23(1)
N4	3403(1)	3666(2)	2257(1)	26(1)
C1	4427(1)	2787(2)	3417(1)	26(1)
C2	4469(1)	3579(2)	4079(1)	22(1)
C3	4583(1)	2775(2)	4744(1)	28(1)
C4	3945(1)	3382(2)	4784(1)	24(1)
C5	4734(1)	4728(2)	4045(1)	25(1)
C6	4177(1)	5487(2)	4430(1)	24(1)
C7	3716(1)	2234(2)	4485(1)	23(1)
C8	3738(1)	1099(2)	4871(1)	32(1)
C9	3522(1)	88(2)	4616(1)	34(1)
C10	3021(1)	-796(2)	3726(1)	38(1)
C11	2763(1)	-620(2)	3148(1)	40(1)
C12	2729(1)	570(2)	2823(1)	37(1)
C13	2964(1)	1555(2)	3070(1)	29(1)
C14	3246(1)	1386(2)	3654(1)	24(1)
C15	3265(1)	207(2)	3999(1)	28(1)
C16	4043(1)	6438(2)	3863(1)	24(1)

C17	4208(1)	7664(2)	3937(1)	34(1)
C18	4105(1)	8541(2)	3441(1)	39(1)
C19	3704(1)	9121(2)	2336(1)	43(1)
C20	3426(1)	8800(2)	1810(1)	48(1)
C21	3250(1)	7594(2)	1798(1)	47(1)
C22	3362(1)	6720(2)	2304(1)	36(1)
C23	3660(1)	7018(2)	2844(1)	27(1)
C24	3825(1)	8247(2)	2873(1)	32(1)
C25	3312(1)	3264(2)	1716(1)	26(1)
C26	3186(1)	2725(2)	1040(1)	38(1)
C27	2677(1)	4295(2)	4656(1)	35(1)
S2	4342(2)	1831(7)	1189(3)	29(1)
F4	4832(2)	194(6)	710(3)	82(2)
F5	4902(1)	410(4)	1828(3)	72(1)
F6	4415(4)	-635(11)	1328(4)	40(2)
O7	4144(1)	1863(4)	1819(2)	34(1)
O8	4086(1)	1674(4)	570(2)	46(1)
O9	4643(2)	2801(6)	1166(3)	52(1)
C28	4635(3)	403(10)	1271(5)	46(3)
S2'	4356(3)	1728(11)	1209(4)	22(2)
F4'	4668(2)	35(10)	476(5)	65(2)
F5'	4998(3)	373(18)	1455(9)	61(3)
F6'	4454(6)	-680(20)	1435(8)	64(4)
O7'	4350(3)	1858(7)	1945(3)	50(2)
O8'	3977(2)	1369(8)	863(5)	55(2)
O9'	4567(3)	2623(8)	862(4)	49(2)
C28'	4632(4)	271(14)	1151(7)	29(3)
S2"	4378(5)	1695(18)	1239(8)	28(4)
F4"	4585(4)	34(16)	2186(7)	67(4)
F5"	5017(9)	460(50)	1460(20)	56(8)
F6"	4516(8)	-720(20)	1134(13)	72(7)
O7"	4506(5)	1925(19)	561(7)	55(4)
O8"	3970(4)	1310(20)	1210(9)	35(4)
O9"	4502(4)	2612(15)	1757(7)	38(3)
C28"	4637(7)	263(19)	1524(9)	35(4)
C29	4285(3)	4399(9)	6708(5)	69(3)

C30	4604(3)	3448(8)	6939(4)	53(2)
O10	4922(2)	4029(3)	7416(5)	50(2)
C31	5221(2)	3200(7)	7754(5)	68(2)
C32	5565(2)	3964(8)	8125(4)	47(2)

Table S-3. Bond lengths [Å] and angles [°] for jonap25.

Fe(1)-O(1)	2.1395(13)	C(4)-H(4)	1.0000
Fe(1)-O(4)	2.1490(12)	C(4)-C(7)	1.525(3)
Fe(1)-N(1)	2.1946(14)	C(5)-H(5A)	0.9900
Fe(1)-N(2)	2.2079(14)	C(5)-H(5B)	0.9900
Fe(1)-N(3)	2.1875(15)	C(6)-H(6)	1.0000
Fe(1)-N(4)	2.1509(15)	C(6)-C(16)	1.522(3)
S(1)-O(4)	1.4605(13)	C(7)-C(8)	1.411(3)
S(1)-O(5)	1.4321(16)	C(8)-H(8)	0.9500
S(1)-O(6)	1.4235(17)	C(8)-C(9)	1.360(3)
S(1)-C(27)	1.823(2)	C(9)-H(9)	0.9500
F(1)-C(27)	1.317(2)	C(9)-C(15)	1.407(3)
F(2)-C(27)	1.309(3)	C(10)-H(10)	0.9500
F(3)-C(27)	1.321(3)	C(10)-C(11)	1.358(4)
O(1)-H(1)	0.90(3)	C(10)-C(15)	1.416(3)
O(1)-C(1)	1.425(2)	C(11)-H(11)	0.9500
O(2)-C(3)	1.428(2)	C(11)-C(12)	1.408(3)
O(2)-C(4)	1.409(2)	C(12)-H(12)	0.9500
O(3)-C(5)	1.434(2)	C(12)-C(13)	1.369(3)
O(3)-C(6)	1.413(2)	C(13)-H(13)	0.9500
N(1)-C(2)	1.518(2)	C(13)-C(14)	1.413(3)
N(1)-C(4)	1.472(2)	C(14)-C(15)	1.414(3)
N(1)-C(6)	1.491(2)	C(16)-C(17)	1.416(3)
N(2)-C(7)	1.326(2)	C(17)-H(17)	0.9500
N(2)-C(14)	1.384(2)	C(17)-C(18)	1.353(3)
N(3)-C(16)	1.322(2)	C(18)-H(18)	0.9500
N(3)-C(23)	1.384(2)	C(18)-C(24)	1.408(3)
N(4)-C(25)	1.137(2)	C(19)-H(19)	0.9500
C(1)-H(1A)	0.9900	C(19)-C(20)	1.355(4)
C(1)-H(1B)	0.9900	C(19)-C(24)	1.418(3)
C(1)-C(2)	1.520(2)	C(20)-H(20)	0.9500
C(2)-C(3)	1.553(2)	C(20)-C(21)	1.409(4)
C(2)-C(5)	1.519(2)	C(21)-H(21)	0.9500
C(3)-H(3A)	0.9900	C(21)-C(22)	1.370(3)
C(3)-H(3B)	0.9900	C(22)-H(22)	0.9500

C(22)-C(23)	1.410(3)	C(31)-H(31B)	0.9900
C(23)-C(24)	1.418(3)	C(31)-C(32)	1.536(11)
C(25)-C(26)	1.444(3)	C(32)-H(32A)	0.9800
C(26)-H(26A)	0.9800	C(32)-H(32B)	0.9800
C(26)-H(26B)	0.9800	C(32)-H(32C)	0.9800
C(26)-H(26C)	0.9800	O(1)-Fe(1)-O(4)	169.77(5)
S(2)-O(7)	1.440(6)	O(1)-Fe(1)-N(1)	75.57(5)
S(2)-O(8)	1.412(6)	O(1)-Fe(1)-N(2)	95.81(5)
S(2)-O(9)	1.453(7)	O(1)-Fe(1)-N(3)	97.91(5)
S(2)-C(28)	1.809(9)	O(1)-Fe(1)-N(4)	79.34(5)
F(4)-C(28)	1.340(9)	O(4)-Fe(1)-N(1)	114.21(5)
F(5)-C(28)	1.333(9)	O(4)-Fe(1)-N(2)	83.83(5)
F(6)-C(28)	1.339(9)	O(4)-Fe(1)-N(3)	87.31(5)
S(2')-O(7')	1.424(7)	O(4)-Fe(1)-N(4)	90.74(5)
S(2')-O(8')	1.437(9)	N(1)-Fe(1)-N(2)	77.96(5)
S(2')-O(9')	1.396(10)	N(3)-Fe(1)-N(1)	78.98(6)
S(2')-C(28')	1.814(12)	N(3)-Fe(1)-N(2)	149.21(5)
F(4')-C(28')	1.338(12)	N(4)-Fe(1)-N(1)	154.82(6)
F(5')-C(28')	1.323(12)	N(4)-Fe(1)-N(2)	102.62(6)
F(6')-C(28')	1.319(12)	N(4)-Fe(1)-N(3)	106.93(6)
S(2")-O(7")	1.433(13)	O(4)-S(1)-C(27)	103.94(9)
S(2")-O(8")	1.437(12)	O(5)-S(1)-O(4)	113.01(8)
S(2")-O(9")	1.425(13)	O(5)-S(1)-C(27)	103.25(11)
S(2")-C(28")	1.812(16)	O(6)-S(1)-O(4)	113.57(10)
F(4")-C(28")	1.323(14)	O(6)-S(1)-O(5)	117.29(12)
F(5")-C(28")	1.323(14)	O(6)-S(1)-C(27)	103.70(10)
F(6")-C(28")	1.329(14)	Fe(1)-O(1)-H(1)	125(2)
C(29)-H(29A)	0.9800	C(1)-O(1)-Fe(1)	115.96(10)
C(29)-H(29B)	0.9800	C(1)-O(1)-H(1)	109(2)
C(29)-H(29C)	0.9800	C(4)-O(2)-C(3)	105.83(13)
C(29)-C(30)	1.512(10)	C(6)-O(3)-C(5)	103.89(12)
C(30)-H(30A)	0.9900	S(1)-O(4)-Fe(1)	126.01(7)
C(30)-H(30B)	0.9900	C(2)-N(1)-Fe(1)	113.96(10)
C(30)-O(10)	1.480(9)	C(4)-N(1)-Fe(1)	109.50(10)
O(10)-C(31)	1.446(8)	C(4)-N(1)-C(2)	103.21(13)
C(31)-H(31A)	0.9900	C(4)-N(1)-C(6)	113.86(13)

C(6)-N(1)-Fe(1)	111.63(10)	C(2)-C(5)-H(5A)	111.3
C(6)-N(1)-C(2)	104.41(12)	C(2)-C(5)-H(5B)	111.3
C(7)-N(2)-Fe(1)	113.46(11)	H(5A)-C(5)-H(5B)	109.2
C(7)-N(2)-C(14)	117.97(15)	O(3)-C(6)-N(1)	106.91(14)
C(14)-N(2)-Fe(1)	128.00(11)	O(3)-C(6)-H(6)	109.5
C(16)-N(3)-Fe(1)	114.36(12)	O(3)-C(6)-C(16)	109.11(14)
C(16)-N(3)-C(23)	118.25(16)	N(1)-C(6)-H(6)	109.5
C(23)-N(3)-Fe(1)	127.25(12)	N(1)-C(6)-C(16)	112.30(13)
C(25)-N(4)-Fe(1)	169.86(15)	C(16)-C(6)-H(6)	109.5
O(1)-C(1)-H(1A)	110.1	N(2)-C(7)-C(4)	117.66(15)
O(1)-C(1)-H(1B)	110.1	N(2)-C(7)-C(8)	123.31(17)
O(1)-C(1)-C(2)	108.09(14)	C(8)-C(7)-C(4)	118.96(16)
H(1A)-C(1)-H(1B)	108.4	C(7)-C(8)-H(8)	120.5
C(2)-C(1)-H(1A)	110.1	C(9)-C(8)-C(7)	118.92(18)
C(2)-C(1)-H(1B)	110.1	C(9)-C(8)-H(8)	120.5
N(1)-C(2)-C(1)	111.12(13)	C(8)-C(9)-H(9)	120.0
N(1)-C(2)-C(3)	103.59(13)	C(8)-C(9)-C(15)	119.98(18)
N(1)-C(2)-C(5)	102.28(14)	C(15)-C(9)-H(9)	120.0
C(1)-C(2)-C(3)	112.41(16)	C(11)-C(10)-H(10)	119.8
C(5)-C(2)-C(1)	114.49(15)	C(11)-C(10)-C(15)	120.4(2)
C(5)-C(2)-C(3)	111.93(14)	C(15)-C(10)-H(10)	119.8
O(2)-C(3)-C(2)	105.97(14)	C(10)-C(11)-H(11)	119.9
O(2)-C(3)-H(3A)	110.5	C(10)-C(11)-C(12)	120.28(19)
O(2)-C(3)-H(3B)	110.5	C(12)-C(11)-H(11)	119.9
C(2)-C(3)-H(3A)	110.5	C(11)-C(12)-H(12)	119.7
C(2)-C(3)-H(3B)	110.5	C(13)-C(12)-C(11)	120.7(2)
H(3A)-C(3)-H(3B)	108.7	C(13)-C(12)-H(12)	119.7
O(2)-C(4)-N(1)	106.48(13)	C(12)-C(13)-H(13)	119.9
O(2)-C(4)-H(4)	109.2	C(12)-C(13)-C(14)	120.3(2)
O(2)-C(4)-C(7)	112.25(15)	C(14)-C(13)-H(13)	119.9
N(1)-C(4)-H(4)	109.2	N(2)-C(14)-C(13)	119.84(16)
N(1)-C(4)-C(7)	110.45(13)	N(2)-C(14)-C(15)	121.42(16)
C(7)-C(4)-H(4)	109.2	C(13)-C(14)-C(15)	118.72(17)
O(3)-C(5)-C(2)	102.49(13)	C(9)-C(15)-C(10)	122.43(19)
O(3)-C(5)-H(5A)	111.3	C(9)-C(15)-C(14)	118.03(17)
O(3)-C(5)-H(5B)	111.3	C(14)-C(15)-C(10)	119.52(19)

N(3)-C(16)-C(6)	120.33(15)	F(2)-C(27)-S(1)	111.42(16)
N(3)-C(16)-C(17)	123.10(18)	F(2)-C(27)-F(1)	107.21(18)
C(17)-C(16)-C(6)	116.57(16)	F(2)-C(27)-F(3)	108.3(2)
C(16)-C(17)-H(17)	120.4	F(3)-C(27)-S(1)	111.32(14)
C(18)-C(17)-C(16)	119.10(19)	O(7)-S(2)-O(9)	113.2(5)
C(18)-C(17)-H(17)	120.4	O(7)-S(2)-C(28)	104.1(5)
C(17)-C(18)-H(18)	119.9	O(8)-S(2)-O(7)	114.5(5)
C(17)-C(18)-C(24)	120.18(18)	O(8)-S(2)-O(9)	115.7(5)
C(24)-C(18)-H(18)	119.9	O(8)-S(2)-C(28)	105.1(5)
C(20)-C(19)-H(19)	119.7	O(9)-S(2)-C(28)	102.3(6)
C(20)-C(19)-C(24)	120.7(2)	F(4)-C(28)-S(2)	112.4(7)
C(24)-C(19)-H(19)	119.7	F(5)-C(28)-S(2)	112.9(7)
C(19)-C(20)-H(20)	119.9	F(5)-C(28)-F(4)	107.1(8)
C(19)-C(20)-C(21)	120.2(2)	F(5)-C(28)-F(6)	106.1(8)
C(21)-C(20)-H(20)	119.9	F(6)-C(28)-S(2)	112.9(9)
C(20)-C(21)-H(21)	119.6	F(6)-C(28)-F(4)	104.8(9)
C(22)-C(21)-C(20)	120.9(2)	O(7')-S(2')-O(8')	112.8(8)
C(22)-C(21)-H(21)	119.6	O(7')-S(2')-C(28')	101.6(8)
C(21)-C(22)-H(22)	120.0	O(8')-S(2')-C(28')	100.9(7)
C(21)-C(22)-C(23)	120.1(2)	O(9')-S(2')-O(7')	117.7(7)
C(23)-C(22)-H(22)	120.0	O(9')-S(2')-O(8')	115.8(9)
N(3)-C(23)-C(22)	119.55(17)	O(9')-S(2')-C(28')	105.0(9)
N(3)-C(23)-C(24)	121.40(18)	F(4')-C(28')-S(2')	108.3(9)
C(22)-C(23)-C(24)	119.05(18)	F(5')-C(28')-S(2')	111.7(13)
C(18)-C(24)-C(19)	123.1(2)	F(5')-C(28')-F(4')	106.0(11)
C(18)-C(24)-C(23)	117.83(19)	F(6')-C(28')-S(2')	111.3(14)
C(23)-C(24)-C(19)	119.1(2)	F(6')-C(28')-F(4')	110.3(12)
N(4)-C(25)-C(26)	177.8(2)	F(6')-C(28')-F(5')	109.1(13)
C(25)-C(26)-H(26A)	109.5	O(7")-S(2")-O(8")	112.8(14)
C(25)-C(26)-H(26B)	109.5	O(7")-S(2")-C(28")	103.7(13)
C(25)-C(26)-H(26C)	109.5	O(8")-S(2")-C(28")	102.0(15)
H(26A)-C(26)-H(26B)	109.5	O(9")-S(2")-O(7")	115.1(13)
H(26A)-C(26)-H(26C)	109.5	O(9")-S(2")-O(8")	116.1(14)
H(26B)-C(26)-H(26C)	109.5	O(9")-S(2")-C(28")	105.0(14)
F(1)-C(27)-S(1)	111.09(15)	F(4")-C(28")-S(2")	109.8(15)
F(1)-C(27)-F(3)	107.3(2)	F(4")-C(28")-F(6")	109.6(18)

F(5")-C(28")-S(2")	107(3)	O(10)-C(30)-H(30B)	109.4
F(5")-C(28")-F(4")	110(2)	C(31)-O(10)-C(30)	117.5(7)
F(5")-C(28")-F(6")	109(2)	O(10)-C(31)-H(31A)	109.5
F(6")-C(28")-S(2")	112.0(18)	O(10)-C(31)-H(31B)	109.5
H(29A)-C(29)-H(29B)	109.5	O(10)-C(31)-C(32)	110.8(6)
H(29A)-C(29)-H(29C)	109.5	H(31A)-C(31)-H(31B)	108.1
H(29B)-C(29)-H(29C)	109.5	C(32)-C(31)-H(31A)	109.5
C(30)-C(29)-H(29A)	109.5	C(32)-C(31)-H(31B)	109.5
C(30)-C(29)-H(29B)	109.5	C(31)-C(32)-H(32A)	109.5
C(30)-C(29)-H(29C)	109.5	C(31)-C(32)-H(32B)	109.5
C(29)-C(30)-H(30A)	109.4	C(31)-C(32)-H(32C)	109.5
C(29)-C(30)-H(30B)	109.4	H(32A)-C(32)-H(32B)	109.5
H(30A)-C(30)-H(30B)	108.0	H(32A)-C(32)-H(32C)	109.5
O(10)-C(30)-C(29)	111.1(7)	H(32B)-C(32)-H(32C)	109.5
O(10)-C(30)-H(30A)	109.4		

Table S-4. Anisotropic displacement parameters ($Å^2 x 10^3$) for jonap25. The anisotropic displacement factor exponent takes the form: $-2\pi^2[h^2 a^{*2}U_{11} + ... + 2 h k a^* b^* U_{12}]$

	U_{11}	U_{22}	U_{33}	U_{23}	U_{13}	U_{12}
Fe1	18(1)	19(1)	20(1)	-1(1)	-1(1)	-1(1)
S1	22(1)	24(1)	37(1)	-8(1)	9(1)	-4(1)
F1	59(1)	71(1)	60(1)	-18(1)	40(1)	-14(1)
F2	56(1)	76(1)	72(1)	-1(1)	5(1)	-45(1)
F3	85(1)	69(1)	83(1)	46(1)	57(1)	39(1)
O1	24(1)	35(1)	19(1)	-6(1)	-1(1)	4(1)
O2	23(1)	49(1)	18(1)	4(1)	-4(1)	-3(1)
O3	21(1)	36(1)	26(1)	-9(1)	-1(1)	-6(1)
O4	22(1)	24(1)	27(1)	-2(1)	2(1)	1(1)
O5	29(1)	91(1)	43(1)	-34(1)	15(1)	-25(1)
O6	51(1)	27(1)	84(1)	6(1)	34(1)	12(1)
N1	16(1)	26(1)	18(1)	-2(1)	2(1)	-2(1)
N2	19(1)	21(1)	22(1)	0(1)	2(1)	0(1)
N3	22(1)	21(1)	26(1)	-2(1)	6(1)	-1(1)
N4	26(1)	28(1)	24(1)	0(1)	-3(1)	1(1)
C1	21(1)	32(1)	25(1)	-6(1)	-2(1)	5(1)
C2	15(1)	29(1)	21(1)	-3(1)	0(1)	0(1)
C3	20(1)	37(1)	27(1)	3(1)	-2(1)	-1(1)
C4	22(1)	34(1)	16(1)	3(1)	0(1)	-2(1)
C5	19(1)	33(1)	23(1)	-3(1)	2(1)	-3(1)
C6	20(1)	29(1)	23(1)	-9(1)	3(1)	-5(1)
C7	18(1)	28(1)	22(1)	4(1)	3(1)	1(1)
C8	24(1)	38(1)	33(1)	15(1)	2(1)	1(1)
C9	26(1)	29(1)	49(1)	16(1)	10(1)	2(1)
C10	32(1)	26(1)	58(1)	-4(1)	22(1)	-5(1)
C11	32(1)	36(1)	54(1)	-19(1)	18(1)	-16(1)
C12	29(1)	47(1)	37(1)	-13(1)	5(1)	-14(1)
C13	26(1)	33(1)	28(1)	-4(1)	2(1)	-7(1)
C14	20(1)	25(1)	27(1)	-4(1)	6(1)	-2(1)
C15	22(1)	24(1)	40(1)	0(1)	13(1)	-2(1)
C16	19(1)	24(1)	30(1)	-8(1)	8(1)	-3(1)

C17	26(1)	26(1)	51(1)	-11(1)	5(1)	-5(1)
C18	28(1)	21(1)	71(2)	-4(1)	13(1)	-5(1)
C19	44(1)	28(1)	61(2)	12(1)	26(1)	7(1)
C20	61(2)	36(1)	50(1)	19(1)	18(1)	15(1)
C21	61(2)	37(1)	40(1)	8(1)	-1(1)	12(1)
C22	46(1)	25(1)	36(1)	3(1)	0(1)	5(1)
C23	29(1)	22(1)	31(1)	0(1)	11(1)	4(1)
C24	29(1)	22(1)	50(1)	4(1)	20(1)	3(1)
C25	21(1)	30(1)	25(1)	4(1)	1(1)	-1(1)
C26	36(1)	56(1)	22(1)	-4(1)	1(1)	-9(1)
C27	33(1)	33(1)	40(1)	-2(1)	12(1)	-2(1)
S2	27(2)	34(1)	26(2)	2(1)	5(1)	-1(1)
F4	72(3)	73(3)	110(5)	7(3)	62(3)	16(3)
F5	57(3)	51(2)	99(3)	5(2)	-43(2)	2(2)
F6	56(4)	33(3)	32(2)	-7(2)	2(2)	-10(2)
O7	51(2)	30(2)	22(2)	-5(1)	11(2)	-4(2)
O8	53(2)	64(3)	18(2)	-5(2)	-11(1)	7(2)
O9	29(2)	40(2)	88(4)	12(3)	6(2)	-11(2)
C28	36(4)	48(5)	54(4)	-3(4)	8(4)	-15(3)
S2'	19(3)	38(3)	7(2)	-8(2)	-4(2)	-5(2)
F4'	65(5)	67(4)	67(4)	-29(3)	38(4)	0(4)
F5'	31(4)	39(5)	111(9)	-22(6)	1(5)	-3(3)
F6'	54(6)	34(5)	110(11)	-2(7)	36(7)	-9(4)
O7'	89(6)	43(4)	17(3)	-7(2)	6(3)	26(5)
O8'	48(4)	50(4)	61(7)	-3(6)	-26(5)	-4(3)
O9'	57(5)	33(4)	60(5)	4(4)	31(4)	-16(3)
C28'	19(4)	24(4)	43(5)	-11(4)	2(4)	3(3)
S2"	24(5)	30(6)	30(7)	7(5)	4(4)	-22(4)
F4"	58(9)	81(10)	61(7)	34(7)	-4(6)	15(8)
F5"	30(8)	56(16)	80(18)	0(15)	-6(10)	-8(8)
F6"	69(13)	35(7)	104(14)	-17(10)	-42(11)	-11(8)
O7"	80(8)	58(8)	30(6)	9(5)	25(6)	-6(7)
O8"	15(6)	40(8)	47(11)	12(9)	-7(6)	-8(5)
O9"	39(7)	37(7)	39(7)	-25(7)	4(6)	-4(6)
C28"	26(7)	22(7)	55(7)	4(6)	-10(7)	-4(6)
C29	84(6)	65(5)	68(5)	-22(4)	52(4)	-19(4)

C30	67(6)	44(4)	53(4)	-17(4)	35(4)	-18(4)
O10	83(6)	40(2)	30(4)	2(2)	16(3)	13(2)
C31	76(5)	44(4)	95(7)	33(4)	57(5)	34(4)
C32	55(5)	49(5)	41(3)	24(4)	30(3)	23(3)

Table S-5. Hydrogen coordinates (x 10^4) and isotropic displacement parameters ($Å^2$ x 10^3) for jonap25.

	x	y	z	U(eq)
H1	4163(9)	3040(30)	2498(16)	63(9)
H1A	4687	2705	3227	32
H1B	4331	1932	3520	32
H3A	4582	1866	4626	34
H3B	4850	3009	4959	34
H4	3769	3894	5064	29
H5A	5017	4506	4162	30
H5B	4702	5123	3576	30
H6	4052	5696	4867	29
H8	3900	1041	5301	38
H9	3545	-697	4855	41
H10	3038	-1597	3949	45
H11	2604	-1303	2962	48
H12	2541	692	2429	45
H13	2938	2355	2847	35
H17	4390	7867	4329	41
H18	4221	9357	3476	47
H19	3818	9941	2345	52
H20	3350	9389	1449	58
H21	3051	7384	1433	56
H22	3240	5914	2291	43
H26A	3189	3378	680	58
H26B	2916	2392	1043	58
H26C	3367	2041	940	58
H29A	4180	4767	7120	104
H29B	4071	3978	6418	104
H29C	4399	5067	6436	104
H30A	4485	2738	7180	63
H30B	4719	3106	6523	63
H31A	5324	2640	7401	82

H31B	5101	2665	8099	82
H32A	5698	4441	7778	70
H32B	5755	3388	8376	70
H32C	5461	4550	8457	70

Table S-6. Torsion angles [°] for jonap25.

Fe1-O1-C1-C2	-44.09(17)	N1-C2-C3-O2	-5.53(18)
Fe1-N1-C2-C1	-14.91(17)	N1-C2-C5-O3	36.27(16)
Fe1-N1-C2-C3	-135.82(11)	N1-C4-C7-N2	-34.7(2)
Fe1-N1-C2-C5	107.70(12)	N1-C4-C7-C8	148.20(16)
Fe1-N1-C4-O2	156.96(11)	N1-C6-C16-N3	15.6(2)
Fe1-N1-C4-C7	34.83(16)	N1-C6-C16-C17	-164.77(15)
Fe1-N1-C6-O3	-136.38(11)	N2-C7-C8-C9	0.7(3)
Fe1-N1-C6-C16	-16.74(16)	N2-C14-C15-C9	4.1(3)
Fe1-N2-C7-C4	15.58(19)	N2-C14-C15-C10	-177.29(16)
Fe1-N2-C7-C8	-167.49(14)	N3-C16-C17-C18	-1.5(3)
Fe1-N2-C14-C13	-17.7(2)	N3-C23-C24-C18	-3.4(3)
Fe1-N2-C14-C15	163.79(12)	N3-C23-C24-C19	176.84(17)
Fe1-N3-C16-C6	-5.92(19)	C1-C2-C3-O2	-125.57(15)
Fe1-N3-C16-C17	174.47(14)	C1-C2-C5-O3	156.56(14)
Fe1-N3-C23-C22	8.9(2)	C2-N1-C4-O2	35.24(17)
Fe1-N3-C23-C24	-171.44(13)	C2-N1-C4-C7	-86.88(15)
O1-C1-C2-N1	36.5(2)	C2-N1-C6-O3	-12.81(17)
O1-C1-C2-C3	152.10(14)	C2-N1-C6-C16	106.83(15)
O1-C1-C2-C5	-78.72(18)	C3-O2-C4-N1	-40.03(18)
O2-C4-C7-N2	-153.40(15)	C3-O2-C4-C7	80.94(17)
O2-C4-C7-C8	29.5(2)	C3-C2-C5-O3	-74.02(17)
O3-C6-C16-N3	133.94(16)	C4-O2-C3-C2	27.51(19)
O3-C6-C16-C17	-46.4(2)	C4-N1-C2-C1	103.75(16)
O4-S1-C27-F1	178.44(15)	C4-N1-C2-C3	-17.17(16)
O4-S1-C27-F2	-62.10(17)	C4-N1-C2-C5	-133.65(13)
O4-S1-C27-F3	58.92(19)	C4-N1-C6-O3	99.00(16)
O5-S1-O4-Fe1	-1.97(14)	C4-N1-C6-C16	-141.36(14)
O5-S1-C27-F1	60.25(18)	C4-C7-C8-C9	177.59(17)
O5-S1-C27-F2	179.72(16)	C5-O3-C6-N1	36.78(17)
O5-S1-C27-F3	-59.26(19)	C5-O3-C6-C16	-84.90(16)
O6-S1-O4-Fe1	134.81(10)	C5-C2-C3-O2	103.93(16)
O6-S1-C27-F1	-62.58(19)	C6-O3-C5-C2	-45.54(17)
O6-S1-C27-F2	56.89(19)	C6-N1-C2-C1	-136.96(15)
O6-S1-C27-F3	177.91(18)	C6-N1-C2-C3	102.12(15)

C6-N1-C2-C5	-14.36(16)	C23-N3-C16-C6	178.03(15)
C6-N1-C4-O2	-77.29(17)	C23-N3-C16-C17	-1.6(3)
C6-N1-C4-C7	160.58(14)	C24-C19-C20-C21	1.0(4)
C6-C16-C17-C18	178.83(17)	C27-S1-O4-Fe1	-113.20(11)
C7-N2-C14-C13	171.57(16)	O7-S2-C28-F4	177.8(6)
C7-N2-C14-C15	-6.9(2)	O7-S2-C28-F5	-60.8(8)
C7-C8-C9-C15	-3.6(3)	O7-S2-C28-F6	59.6(8)
C8-C9-C15-C10	-177.29(19)	O8-S2-C28-F4	57.2(8)
C8-C9-C15-C14	1.3(3)	O8-S2-C28-F5	178.5(7)
C10-C11-C12-C13	2.2(3)	O8-S2-C28-F6	-61.1(8)
C11-C10-C15-C9	176.76(19)	O9-S2-C28-F4	-64.1(8)
C11-C10-C15-C14	-1.8(3)	O9-S2-C28-F5	57.3(8)
C11-C12-C13-C14	0.3(3)	O9-S2-C28-F6	177.6(7)
C12-C13-C14-N2	177.99(17)	O7'-S2'-C28'-F4'	-179.0(9)
C12-C13-C14-C15	-3.5(3)	O7'-S2'-C28'-F5'	-62.6(13)
C13-C14-C15-C9	-174.42(17)	O7'-S2'-C28'-F6'	59.6(12)
C13-C14-C15-C10	4.2(3)	O8'-S2'-C28'-F4'	64.8(11)
C14-N2-C7-C4	-172.38(14)	O8'-S2'-C28'-F5'	-178.8(11)
C14-N2-C7-C8	4.5(3)	O8'-S2'-C28'-F6'	-56.6(12)
C15-C10-C11-C12	-1.4(3)	O9'-S2'-C28'-F4'	-55.8(11)
C16-N3-C23-C22	-175.66(17)	O9'-S2'-C28'-F5'	60.5(12)
C16-N3-C23-C24	4.0(2)	O9'-S2'-C28'-F6'	-177.3(10)
C16-C17-C18-C24	2.2(3)	O7"-S2"-C28"-F4"	171.8(15)
C17-C18-C24-C19	180.0(2)	O7"-S2"-C28"-F5"	52(2)
C17-C18-C24-C23	0.2(3)	O7"-S2"-C28"-F6"	-66(2)
C19-C20-C21-C22	-1.5(4)	O8"-S2"-C28"-F4"	-70.8(17)
C20-C19-C24-C18	-178.3(2)	O8"-S2"-C28"-F5"	170(2)
C20-C19-C24-C23	1.5(3)	O8"-S2"-C28"-F6"	51(2)
C20-C21-C22-C23	-0.5(4)	O9"-S2"-C28"-F4"	50.7(17)
C21-C22-C23-N3	-177.3(2)	O9"-S2"-C28"-F5"	-69(2)
C21-C22-C23-C24	3.0(3)	O9"-S2"-C28"-F6"	172.6(17)
C22-C23-C24-C18	176.33(18)	C29-C30-O10-C31	172.1(8)
C22-C23-C24-C19	-3.5(3)	C30-O10-C31-C32	170.1(7)

Table S-7. Hydrogen bonds and close contacts for jonap25 [Å and °].

D-H...A	d(D-H)	d(H...A)	d(D...A)	<(DHA)
O1-H1...O7	0.90(3)	1.80(3)	2.668(4)	161(3)
O1-H1...O7'	0.90(3)	1.79(3)	2.622(6)	151(3)
O1-H1...O9"	0.90(3)	1.97(3)	2.761(13)	146(3)

REFERENCE NUMBER: jonap27

2c

CRYSTAL STRUCTURE REPORT

$C_{28}H_{24}CoF_6N_4O_9S_2$

or

[(κ^4-L^q)Co(NCMe)(OTf)][OTf]

Report prepared for:

R. Zhang, A. Panda, Prof. W. Jones

October 03, 2023

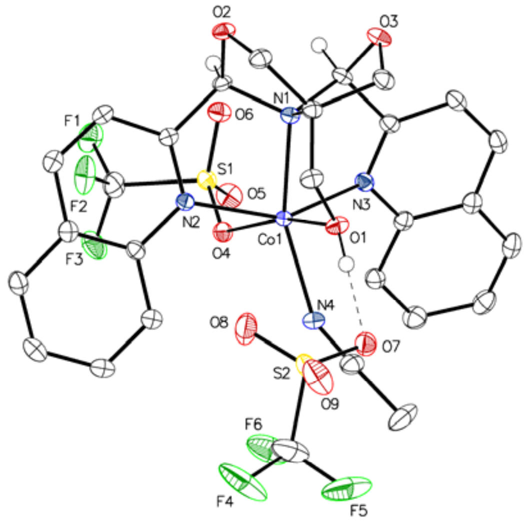

William W. Brennessel
X-ray Crystallographic Facility
Department of Chemistry, University of Rochester
120 Trustee Road
Rochester, NY 14627

Data collection

A crystal (0.395 x 0.222 x 0.073 mm^3) was placed onto a nylon loop and mounted on a Rigaku XtaLAB Synergy-S Dualflex diffractometer equipped with a HyPix-6000HE HPC area detector for data collection at 100.00(10) K. A preliminary set of cell constants and an orientation matrix were calculated from a small sampling of reflections.[1] A short pre-experiment was run, from which an optimal data collection strategy was determined. The full data collection was carried out using a PhotonJet (Cu) X-ray source with frame times of 0.09 and 0.37 seconds and a detector distance of 34.0 mm. Series of frames were collected in 0.50º steps in ω at different 2θ, κ, and ϕ settings. After the intensity data were corrected for absorption, the final cell constants were calculated from the xyz centroids of 33064 strong reflections from the actual data collection after integration.[1] See Table S-1 for additional crystal and refinement information.

Structure solution and refinement

The structure was solved using SHELXT[2] and refined using SHELXL.[3] The space group $P2_1/c$ was determined based on systematic absences. Most or all non-hydrogen atoms were assigned from the solution. Full-matrix least squares / difference Fourier cycles were performed which located any remaining non-hydrogen atoms. All non-hydrogen atoms were refined with anisotropic displacement parameters. The O-H hydrogen atom was found form the difference Fourier map and refined freely. All other hydrogen atoms were placed in ideal positions and refined as riding atoms with relative isotropic displacement parameters. The final full matrix least squares refinement converged to $R1 = 0.0310$ (F^2, $I > 2\sigma(I)$) and $wR2 = 0.0787$ (F^2, all data).

Structure description

The structure is the one suggested. The asymmetric unit contains one monocationic Co complex and one triflate anion in general positions. The cation and anion are linked via hydrogen bonding (see figure and Table S-7).

Structure manipulation and figure generation were performed using Olex2.[4] Unless noted otherwise all structural diagrams containing anisotropic displacement ellipsoids are drawn at the 50 % probability level.

Data collection, structure solution, and structure refinement were conducted at the X-ray Crystallographic Facility, B04 Hutchison Hall, Department of Chemistry, University of Rochester. The instrument was purchased with funding from NSF MRI program grant CHE-1725028. All publications arising from this report MUST either 1) include William W. Brennessel as a coauthor or 2) acknowledge William W. Brennessel and the X-ray Crystallographic Facility of the Department of Chemistry at the University of Rochester.

[1] *CrysAlisPro*, version 171.42.101a; Rigaku Corporation: Oxford, UK, 2023.

[2] Sheldrick, G. M. *SHELXT*, version 2018/2; *Acta. Crystallogr.* **2015**, *A71*, 3-8.

[3] Sheldrick, G. M. *SHELXL*, version 2019/2; *Acta. Crystallogr.* **2015**, *C71*, 3-8.

[4] Dolomanov, O. V.; Bourhis, L. J.; Gildea, R. J.; Howard, J. A. K.; Puschmann, H. *Olex2*, version 1.5; *J. Appl. Cryst.* **2009**, *42*, 339-341.

Some equations of interest:

$$R_{int} = \Sigma |F_o^2 - \langle F_o^2 \rangle| / \Sigma|F_o^2|$$

$$R1 = \Sigma||F_o|-|F_c|| / \Sigma|F_o|$$

$$wR2 = [\Sigma[w(F_o^2-F_c^2)^2] / \Sigma [w(F_o^2)^2]]^{1/2}$$

$$\text{where } w = 1 / [\sigma^2 (F_o^2) + (aP)^2 + bP] \text{ and}$$

$$P = 1/3 \max (0, F_o^2) + 2/3 F_c^2$$

$$\text{GOF} = S = [\Sigma[w(F_o^2-F_c^2)^2] / (m-n)]^{1/2}$$

where m = number of reflections and n = number of parameters

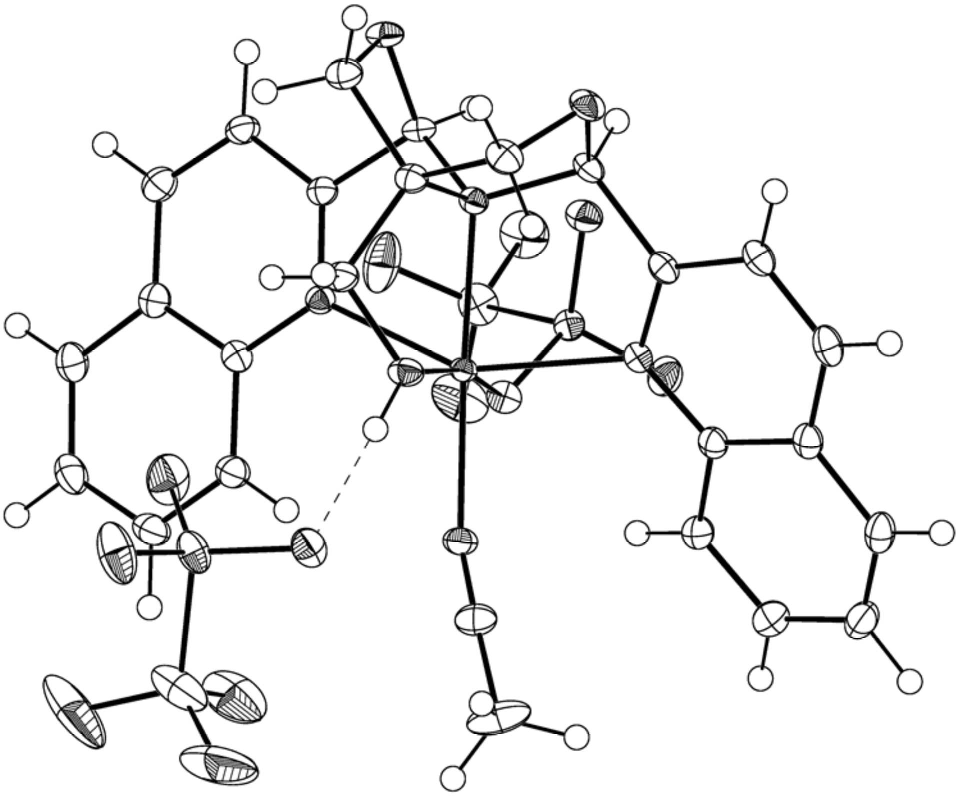

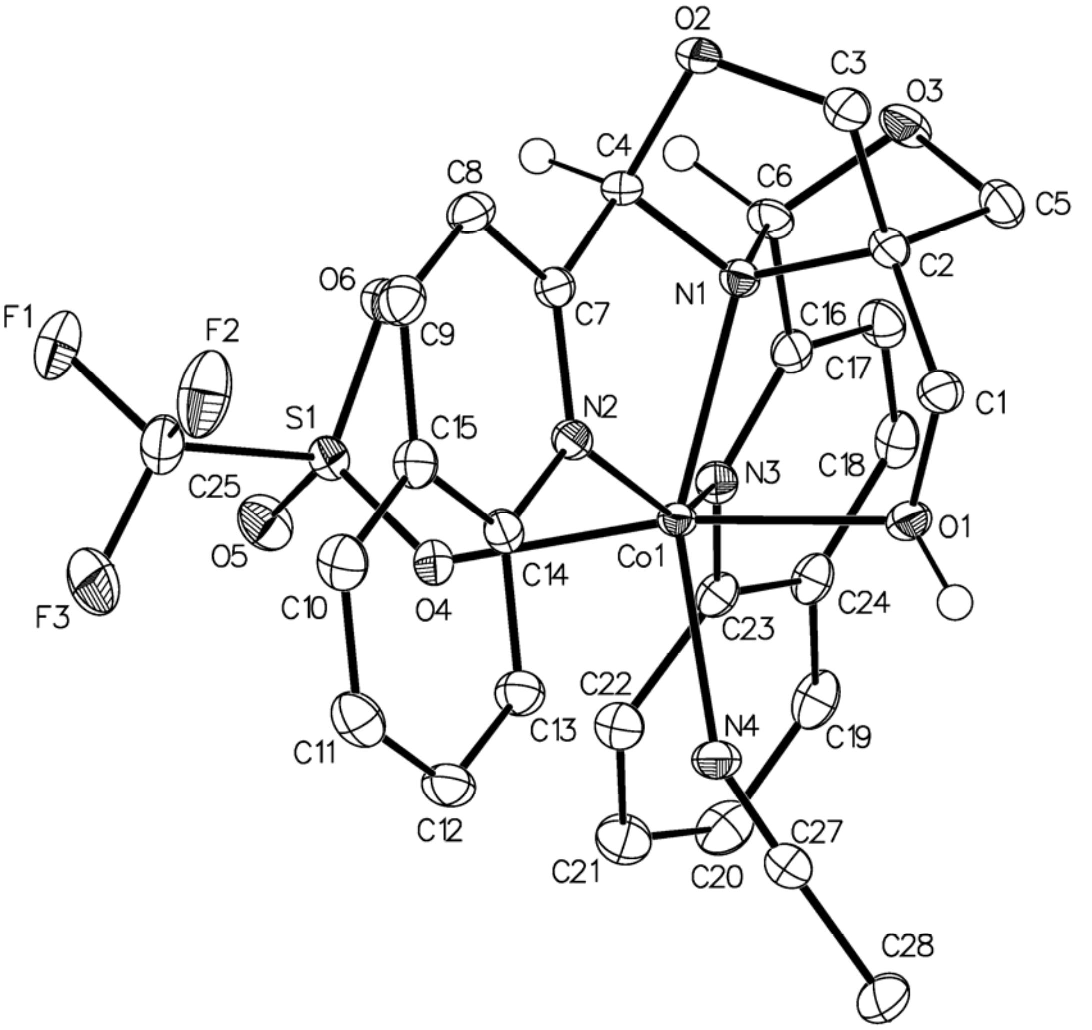
O2
C3
O3
C4
C6
C5
C8
C2
N1
O6
C7
F1
C16
C9
F2
C17
C1
N2
S1
C15
N3
C18
C25
O1
Co1
O5
C14
C10
C24
O4
F3
C23
C22
C19
N4
C13
C11
C27
C12
C21
C20
C28

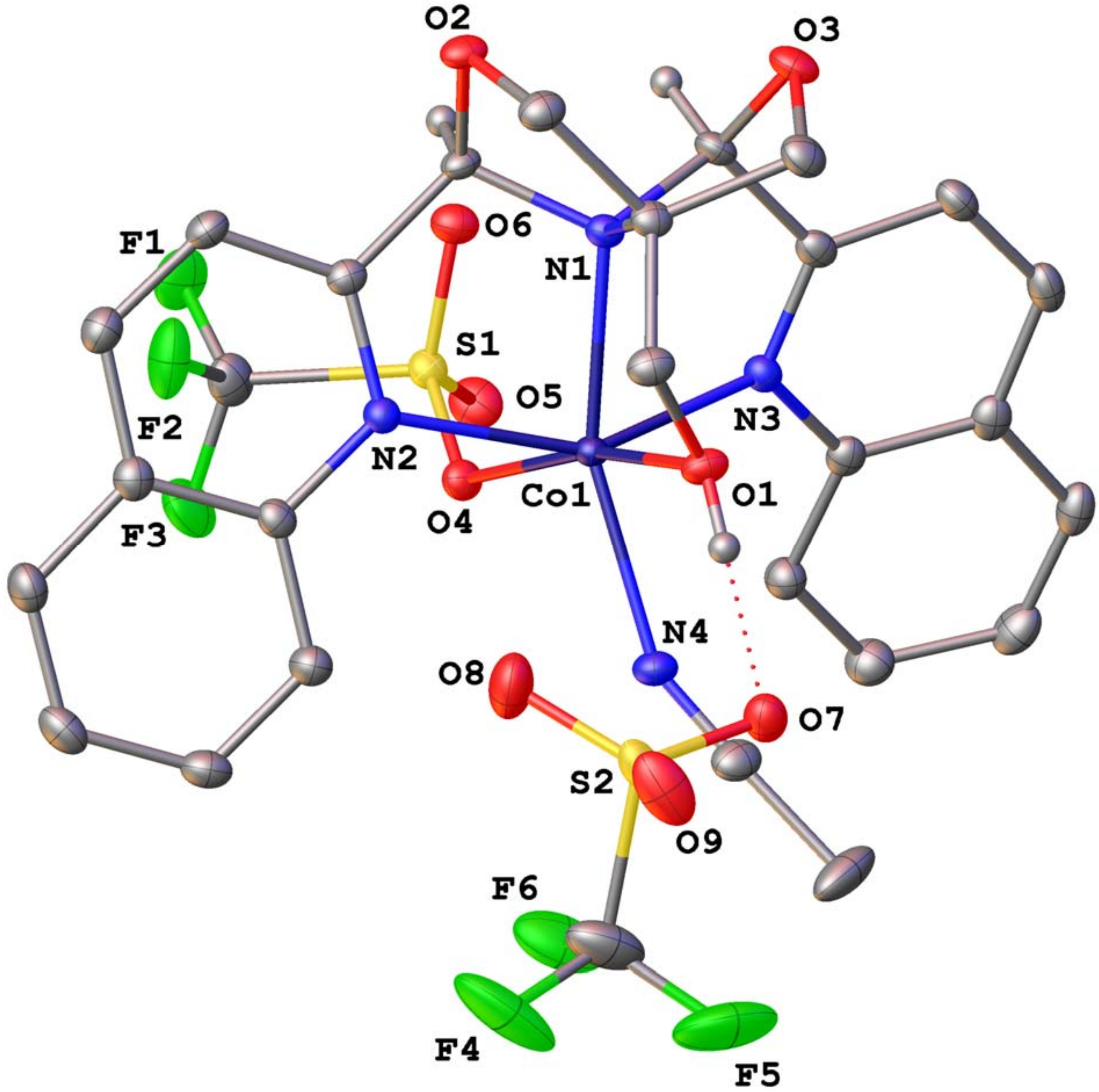
O2
O3
F1
O6
N1
S1
O5
F2
N3
N2
Co1
O1
F3
O4
N4
O8
O7
S2
O9
F6
F4
F5

Table S-1. Crystal data and structure refinement for jonap27.

Identification code	jonap27	
Empirical formula	C28 H24 Co F6 N4 O9 S2	
Formula weight	797.56	
Temperature	100.00(10) K	
Wavelength	1.54184 Å	
Crystal system	monoclinic	
Space group	$P2_{1/c}$	
Unit cell dimensions	a = 16.21480(10) Å	α = 90°
	b = 10.89330(10) Å	β = 100.2390(10)°
	c = 18.22090(10) Å	γ = 90°
Volume	3167.15(4) $Å^3$	
Z	4	
Density (calculated)	1.673 Mg/m^3	
Absorption coefficient	6.351 mm^{-1}	
F(000)	1620	
Crystal color, morphology	violet, plate	
Crystal size	0.395 x 0.222 x 0.073 mm^3	
Theta range for data collection	4.750 to 80.383°	
Index ranges	$-20 \le h \le 20$, $-13 \le k \le 10$, $-23 \le l \le 22$	
Reflections collected	55218	
Independent reflections	6832 [R(int) = 0.0430]	
Observed reflections	6546	
Completeness to theta = 74.504°	100.0%	
Absorption correction	Multi-scan	
Max. and min. transmission	1.00000 and 0.38099	
Refinement method	Full-matrix least-squares on F^2	
Data / restraints / parameters	6832 / 0 / 456	
Goodness-of-fit on F^2	1.016	
Final R indices [I>2sigma(I)]	R1 = 0.0310, wR2 = 0.0779	
R indices (all data)	R1 = 0.0324, wR2 = 0.0787	
Largest diff. peak and hole	0.348 and -0.508 e.$Å^{-3}$	

Table S-2. Atomic coordinates (x 10^4) and equivalent isotropic displacement parameters ($Å^2$x 10^3) for jonap27. U_{eq} is defined as one third of the trace of the orthogonalized U_{ij} tensor.

	x	y	z	U_{eq}
Co1	7225(1)	2820(1)	5397(1)	11(1)
S1	6164(1)	2849(1)	3692(1)	14(1)
F1	6352(1)	3090(1)	2305(1)	30(1)
F2	7412(1)	3708(1)	3102(1)	34(1)
F3	6287(1)	4804(1)	2882(1)	37(1)
O1	7939(1)	2487(1)	6463(1)	16(1)
O2	8876(1)	218(1)	4760(1)	16(1)
O3	7622(1)	-896(1)	5721(1)	18(1)
O4	6509(1)	3550(1)	4360(1)	16(1)
O5	5272(1)	2950(1)	3472(1)	22(1)
O6	6506(1)	1629(1)	3678(1)	18(1)
N1	7807(1)	1077(1)	5276(1)	12(1)
N2	8240(1)	3309(1)	4854(1)	13(1)
N3	6180(1)	1613(1)	5442(1)	13(1)
N4	6741(1)	4248(1)	5972(1)	16(1)
C1	8722(1)	1907(2)	6418(1)	16(1)
C2	8511(1)	788(1)	5922(1)	14(1)
C3	9235(1)	360(2)	5533(1)	18(1)
C4	8227(1)	1095(1)	4622(1)	13(1)
C5	8139(1)	-253(2)	6317(1)	18(1)
C6	7206(1)	48(1)	5269(1)	15(1)
C7	8558(1)	2383(2)	4523(1)	13(1)
C8	9158(1)	2531(2)	4055(1)	16(1)
C9	9421(1)	3686(2)	3926(1)	18(1)
C10	9320(1)	5933(2)	4126(1)	20(1)
C11	8981(1)	6888(2)	4456(1)	22(1)
C12	8408(1)	6662(2)	4939(1)	20(1)
C13	8171(1)	5487(2)	5073(1)	18(1)
C14	8499(1)	4484(2)	4730(1)	14(1)
C15	9092(1)	4704(2)	4258(1)	16(1)
C16	6417(1)	454(2)	5552(1)	14(1)

C17	5959(1)	-435(2)	5874(1)	18(1)
C18	5239(1)	-85(2)	6104(1)	18(1)
C19	4164(1)	1519(2)	6148(1)	20(1)
C20	3861(1)	2667(2)	5958(1)	22(1)
C21	4310(1)	3462(2)	5560(1)	21(1)
C22	5067(1)	3120(2)	5384(1)	17(1)
C23	5407(1)	1948(2)	5599(1)	14(1)
C24	4933(1)	1122(2)	5961(1)	16(1)
C25	6579(1)	3663(2)	2958(1)	22(1)
C27	6486(1)	4647(2)	6464(1)	20(1)
C28	6156(1)	5113(2)	7099(1)	34(1)
S2	8893(1)	4974(1)	7463(1)	20(1)
F4	9000(1)	7362(1)	7452(1)	68(1)
F5	8059(1)	6663(2)	8043(1)	65(1)
F6	7836(1)	6656(1)	6839(1)	50(1)
O7	8216(1)	4163(1)	7577(1)	21(1)
O8	9108(1)	4855(1)	6736(1)	33(1)
O9	9584(1)	5042(1)	8076(1)	36(1)
C26	8424(2)	6492(2)	7447(1)	40(1)

Table S-3. Bond lengths [Å] and angles [°] for jonap27.

Co(1)-O(1)	2.1080(12)	C(4)-H(4)	1.0000
Co(1)-O(4)	2.1854(11)	C(4)-C(7)	1.524(2)
Co(1)-N(1)	2.1497(13)	C(5)-H(5A)	0.9900
Co(1)-N(2)	2.1332(13)	C(5)-H(5B)	0.9900
Co(1)-N(3)	2.1589(13)	C(6)-H(6)	1.0000
Co(1)-N(4)	2.1035(14)	C(6)-C(16)	1.527(2)
S(1)-O(4)	1.4612(12)	C(7)-C(8)	1.413(2)
S(1)-O(5)	1.4348(12)	C(8)-H(8)	0.9500
S(1)-O(6)	1.4421(12)	C(8)-C(9)	1.363(2)
S(1)-C(25)	1.8306(18)	C(9)-H(9)	0.9500
F(1)-C(25)	1.336(2)	C(9)-C(15)	1.412(2)
F(2)-C(25)	1.329(2)	C(10)-H(10)	0.9500
F(3)-C(25)	1.329(2)	C(10)-C(11)	1.366(3)
O(1)-C(1)	1.4328(19)	C(10)-C(15)	1.420(2)
O(1)-H(1)	0.87(3)	C(11)-H(11)	0.9500
O(2)-C(3)	1.4329(19)	C(11)-C(12)	1.410(3)
O(2)-C(4)	1.4104(18)	C(12)-H(12)	0.9500
O(3)-C(5)	1.431(2)	C(12)-C(13)	1.372(2)
O(3)-C(6)	1.4121(19)	C(13)-H(13)	0.9500
N(1)-C(2)	1.5205(19)	C(13)-C(14)	1.408(2)
N(1)-C(4)	1.4739(19)	C(14)-C(15)	1.421(2)
N(1)-C(6)	1.484(2)	C(16)-C(17)	1.410(2)
N(2)-C(7)	1.325(2)	C(17)-H(17)	0.9500
N(2)-C(14)	1.378(2)	C(17)-C(18)	1.364(2)
N(3)-C(16)	1.325(2)	C(18)-H(18)	0.9500
N(3)-C(23)	1.382(2)	C(18)-C(24)	1.413(2)
N(4)-C(27)	1.137(2)	C(19)-H(19)	0.9500
C(1)-H(1A)	0.9900	C(19)-C(20)	1.365(3)
C(1)-H(1B)	0.9900	C(19)-C(24)	1.417(2)
C(1)-C(2)	1.520(2)	C(20)-H(20)	0.9500
C(2)-C(3)	1.548(2)	C(20)-C(21)	1.412(3)
C(2)-C(5)	1.524(2)	C(21)-H(21)	0.9500
C(3)-H(3A)	0.9900	C(21)-C(22)	1.374(2)
C(3)-H(3B)	0.9900	C(22)-H(22)	0.9500

C(22)-C(23)	1.418(2)	C(1)-O(1)-H(1)	108.8(17)
C(23)-C(24)	1.420(2)	C(4)-O(2)-C(3)	105.21(11)
C(27)-C(28)	1.451(2)	C(6)-O(3)-C(5)	103.91(12)
C(28)-H(28A)	0.9800	S(1)-O(4)-Co(1)	126.64(7)
C(28)-H(28B)	0.9800	C(2)-N(1)-Co(1)	112.69(9)
C(28)-H(28C)	0.9800	C(4)-N(1)-Co(1)	110.22(9)
S(2)-O(7)	1.4530(13)	C(4)-N(1)-C(2)	103.82(11)
S(2)-O(8)	1.4349(14)	C(4)-N(1)-C(6)	113.61(12)
S(2)-O(9)	1.4354(14)	C(6)-N(1)-Co(1)	111.63(9)
S(2)-C(26)	1.818(2)	C(6)-N(1)-C(2)	104.55(12)
F(4)-C(26)	1.329(3)	C(7)-N(2)-Co(1)	114.56(11)
F(5)-C(26)	1.338(3)	C(7)-N(2)-C(14)	118.60(14)
F(6)-C(26)	1.339(3)	C(14)-N(2)-Co(1)	126.14(10)
O(1)-Co(1)-O(4)	168.59(5)	C(16)-N(3)-Co(1)	112.12(10)
O(1)-Co(1)-N(1)	76.31(5)	C(16)-N(3)-C(23)	118.11(14)
O(1)-Co(1)-N(2)	97.37(5)	C(23)-N(3)-Co(1)	126.40(11)
O(1)-Co(1)-N(3)	99.58(5)	C(27)-N(4)-Co(1)	153.50(14)
N(1)-Co(1)-O(4)	114.04(5)	O(1)-C(1)-H(1A)	110.5
N(1)-Co(1)-N(3)	80.08(5)	O(1)-C(1)-H(1B)	110.5
N(2)-Co(1)-O(4)	80.93(5)	O(1)-C(1)-C(2)	106.17(13)
N(2)-Co(1)-N(1)	77.51(5)	H(1A)-C(1)-H(1B)	108.7
N(2)-Co(1)-N(3)	147.70(5)	C(2)-C(1)-H(1A)	110.5
N(3)-Co(1)-O(4)	87.37(5)	C(2)-C(1)-H(1B)	110.5
N(4)-Co(1)-O(1)	82.04(5)	N(1)-C(2)-C(3)	103.44(12)
N(4)-Co(1)-O(4)	88.52(5)	N(1)-C(2)-C(5)	102.37(12)
N(4)-Co(1)-N(1)	156.08(5)	C(1)-C(2)-N(1)	110.70(12)
N(4)-Co(1)-N(2)	115.72(5)	C(1)-C(2)-C(3)	113.89(13)
N(4)-Co(1)-N(3)	93.79(5)	C(1)-C(2)-C(5)	112.52(14)
O(4)-S(1)-C(25)	102.65(7)	C(5)-C(2)-C(3)	112.87(13)
O(5)-S(1)-O(4)	114.22(7)	O(2)-C(3)-C(2)	105.94(12)
O(5)-S(1)-O(6)	116.02(7)	O(2)-C(3)-H(3A)	110.5
O(5)-S(1)-C(25)	104.05(8)	O(2)-C(3)-H(3B)	110.5
O(6)-S(1)-O(4)	113.84(7)	C(2)-C(3)-H(3A)	110.5
O(6)-S(1)-C(25)	103.91(8)	C(2)-C(3)-H(3B)	110.5
Co(1)-O(1)-H(1)	123.6(17)	H(3A)-C(3)-H(3B)	108.7
C(1)-O(1)-Co(1)	111.77(9)	O(2)-C(4)-N(1)	106.45(12)

O(2)-C(4)-H(4)	109.6	C(12)-C(13)-C(14)	120.36(16)
O(2)-C(4)-C(7)	112.18(13)	C(14)-C(13)-H(13)	119.8
N(1)-C(4)-H(4)	109.6	N(2)-C(14)-C(13)	119.89(14)
N(1)-C(4)-C(7)	109.30(12)	N(2)-C(14)-C(15)	120.94(14)
C(7)-C(4)-H(4)	109.6	C(13)-C(14)-C(15)	119.17(15)
O(3)-C(5)-C(2)	103.57(13)	C(9)-C(15)-C(10)	122.55(15)
O(3)-C(5)-H(5A)	111.0	C(9)-C(15)-C(14)	118.35(15)
O(3)-C(5)-H(5B)	111.0	C(10)-C(15)-C(14)	119.09(15)
C(2)-C(5)-H(5A)	111.0	N(3)-C(16)-C(6)	117.71(14)
C(2)-C(5)-H(5B)	111.0	N(3)-C(16)-C(17)	123.78(15)
H(5A)-C(5)-H(5B)	109.0	C(17)-C(16)-C(6)	118.41(14)
O(3)-C(6)-N(1)	107.51(12)	C(16)-C(17)-H(17)	120.7
O(3)-C(6)-H(6)	109.0	C(18)-C(17)-C(16)	118.68(15)
O(3)-C(6)-C(16)	110.86(13)	C(18)-C(17)-H(17)	120.7
N(1)-C(6)-H(6)	109.0	C(17)-C(18)-H(18)	120.2
N(1)-C(6)-C(16)	111.49(12)	C(17)-C(18)-C(24)	119.64(15)
C(16)-C(6)-H(6)	109.0	C(24)-C(18)-H(18)	120.2
N(2)-C(7)-C(4)	118.27(14)	C(20)-C(19)-H(19)	119.6
N(2)-C(7)-C(8)	123.58(15)	C(20)-C(19)-C(24)	120.78(16)
C(8)-C(7)-C(4)	118.09(14)	C(24)-C(19)-H(19)	119.6
C(7)-C(8)-H(8)	120.7	C(19)-C(20)-H(20)	120.1
C(9)-C(8)-C(7)	118.66(15)	C(19)-C(20)-C(21)	119.73(16)
C(9)-C(8)-H(8)	120.7	C(21)-C(20)-H(20)	120.1
C(8)-C(9)-H(9)	120.1	C(20)-C(21)-H(21)	119.4
C(8)-C(9)-C(15)	119.86(15)	C(22)-C(21)-C(20)	121.14(17)
C(15)-C(9)-H(9)	120.1	C(22)-C(21)-H(21)	119.4
C(11)-C(10)-H(10)	119.8	C(21)-C(22)-H(22)	120.0
C(11)-C(10)-C(15)	120.44(16)	C(21)-C(22)-C(23)	119.96(16)
C(15)-C(10)-H(10)	119.8	C(23)-C(22)-H(22)	120.0
C(10)-C(11)-H(11)	119.9	N(3)-C(23)-C(22)	120.22(15)
C(10)-C(11)-C(12)	120.20(16)	N(3)-C(23)-C(24)	120.91(15)
C(12)-C(11)-H(11)	119.9	C(22)-C(23)-C(24)	118.86(15)
C(11)-C(12)-H(12)	119.6	C(18)-C(24)-C(19)	122.33(16)
C(13)-C(12)-C(11)	120.72(16)	C(18)-C(24)-C(23)	118.31(15)
C(13)-C(12)-H(12)	119.6	C(19)-C(24)-C(23)	119.34(16)
C(12)-C(13)-H(13)	119.8	F(1)-C(25)-S(1)	110.17(13)

F(2)-C(25)-S(1)	111.47(12)	O(7)-S(2)-C(26)	103.36(10)
F(2)-C(25)-F(1)	107.69(15)	O(8)-S(2)-O(7)	113.01(8)
F(2)-C(25)-F(3)	108.48(16)	O(8)-S(2)-O(9)	115.97(10)
F(3)-C(25)-S(1)	110.98(13)	O(8)-S(2)-C(26)	103.72(11)
F(3)-C(25)-F(1)	107.92(14)	O(9)-S(2)-O(7)	115.18(8)
N(4)-C(27)-C(28)	178.0(2)	O(9)-S(2)-C(26)	103.43(10)
C(27)-C(28)-H(28A)	109.5	F(4)-C(26)-S(2)	110.89(18)
C(27)-C(28)-H(28B)	109.5	F(4)-C(26)-F(5)	107.90(19)
C(27)-C(28)-H(28C)	109.5	F(4)-C(26)-F(6)	107.86(19)
H(28A)-C(28)-H(28B)	109.5	F(5)-C(26)-S(2)	110.93(16)
H(28A)-C(28)-H(28C)	109.5	F(5)-C(26)-F(6)	107.4(2)
H(28B)-C(28)-H(28C)	109.5	F(6)-C(26)-S(2)	111.65(14)

Table S-4. Anisotropic displacement parameters ($Å^2$x 10^3) for jonap27. The anisotropic displacement factor exponent takes the form: $-2\pi^2[h^2 a^{*2}U_{11} + ... + 2 h k a^* b^* U_{12}]$

	U_{11}	U_{22}	U_{33}	U_{23}	U_{13}	U_{12}
Co1	12(1)	9(1)	12(1)	-1(1)	3(1)	0(1)
S1	14(1)	14(1)	14(1)	2(1)	1(1)	1(1)
F1	36(1)	39(1)	13(1)	0(1)	1(1)	-1(1)
F2	25(1)	56(1)	22(1)	7(1)	6(1)	-12(1)
F3	59(1)	23(1)	28(1)	11(1)	9(1)	2(1)
O1	18(1)	16(1)	14(1)	-4(1)	1(1)	3(1)
O2	18(1)	14(1)	16(1)	-2(1)	2(1)	6(1)
O3	21(1)	10(1)	24(1)	1(1)	1(1)	1(1)
O4	19(1)	15(1)	13(1)	2(1)	2(1)	1(1)
O5	15(1)	24(1)	26(1)	1(1)	-1(1)	1(1)
O6	20(1)	15(1)	19(1)	-1(1)	2(1)	2(1)
N1	13(1)	11(1)	12(1)	-1(1)	1(1)	1(1)
N2	13(1)	12(1)	13(1)	1(1)	2(1)	1(1)
N3	14(1)	12(1)	14(1)	0(1)	2(1)	-1(1)
N4	18(1)	13(1)	18(1)	-2(1)	4(1)	2(1)
C1	15(1)	17(1)	16(1)	-2(1)	0(1)	3(1)
C2	15(1)	13(1)	14(1)	1(1)	1(1)	3(1)
C3	17(1)	18(1)	17(1)	-1(1)	2(1)	4(1)
C4	15(1)	12(1)	13(1)	-1(1)	3(1)	4(1)
C5	22(1)	14(1)	18(1)	3(1)	1(1)	2(1)
C6	18(1)	9(1)	17(1)	-1(1)	2(1)	-1(1)
C7	13(1)	14(1)	12(1)	-1(1)	1(1)	2(1)
C8	16(1)	18(1)	16(1)	-3(1)	5(1)	2(1)
C9	18(1)	22(1)	17(1)	0(1)	5(1)	1(1)
C10	21(1)	20(1)	21(1)	2(1)	5(1)	-4(1)
C11	22(1)	15(1)	26(1)	3(1)	1(1)	-4(1)
C12	22(1)	14(1)	22(1)	-2(1)	2(1)	2(1)
C13	19(1)	16(1)	19(1)	-1(1)	4(1)	0(1)
C14	15(1)	13(1)	14(1)	2(1)	1(1)	0(1)
C15	16(1)	18(1)	15(1)	1(1)	2(1)	-1(1)
C16	16(1)	13(1)	13(1)	-1(1)	0(1)	-2(1)

C17	21(1)	12(1)	18(1)	2(1)	2(1)	-3(1)
C18	21(1)	19(1)	15(1)	2(1)	2(1)	-7(1)
C19	17(1)	27(1)	15(1)	-1(1)	4(1)	-6(1)
C20	15(1)	30(1)	22(1)	-5(1)	6(1)	-1(1)
C21	18(1)	22(1)	24(1)	0(1)	4(1)	2(1)
C22	17(1)	16(1)	19(1)	0(1)	4(1)	-1(1)
C23	15(1)	16(1)	12(1)	-2(1)	2(1)	-2(1)
C24	16(1)	20(1)	12(1)	-2(1)	1(1)	-3(1)
C25	26(1)	25(1)	15(1)	2(1)	1(1)	-1(1)
C27	22(1)	18(1)	19(1)	1(1)	2(1)	7(1)
C28	43(1)	44(1)	19(1)	1(1)	11(1)	24(1)
S2	24(1)	18(1)	16(1)	3(1)	-1(1)	-6(1)
F4	90(1)	21(1)	79(1)	9(1)	-27(1)	-19(1)
F5	87(1)	50(1)	56(1)	-33(1)	4(1)	18(1)
F6	61(1)	27(1)	52(1)	3(1)	-18(1)	10(1)
O7	25(1)	21(1)	18(1)	-2(1)	5(1)	-5(1)
O8	38(1)	39(1)	24(1)	0(1)	12(1)	-10(1)
O9	35(1)	34(1)	30(1)	9(1)	-13(1)	-14(1)
C26	55(1)	20(1)	38(1)	-6(1)	-8(1)	1(1)

Table S-5. Hydrogen coordinates (x 10^4) and isotropic displacement parameters ($Å^2$ x 10^3) for jonap27.

	x	y	z	U(eq)
H1A	9014	1659	6921	20
H1B	9090	2475	6200	20
H3A	9689	980	5595	21
H3B	9469	-428	5745	21
H4	7818	857	4166	16
H5A	8584	-794	6584	22
H5B	7802	70	6677	22
H6	7044	-264	4747	18
H8	9375	1839	3835	20
H9	9824	3807	3613	22
H10	9712	6091	3807	24
H11	9131	7708	4360	26
H12	8183	7332	5173	24
H13	7783	5348	5398	21
H17	6149	-1260	5929	21
H18	4944	-650	6359	22
H19	3856	980	6408	23
H20	3350	2930	6094	27
H21	4085	4247	5412	26
H22	5362	3668	5118	21
H28A	5565	4881	7052	51
H28B	6206	6009	7116	51
H28C	6474	4763	7559	51
H1	8021(16)	3030(20)	6817(15)	36(7)

Table S-6. Torsion angles [°] for jonap27.

Co1-O1-C1-C2	-52.63(14)	N1-C2-C3-O2	-10.09(16)
Co1-N1-C2-C1	-10.10(15)	N1-C2-C5-O3	33.21(15)
Co1-N1-C2-C3	-132.48(10)	N1-C4-C7-N2	-18.87(19)
Co1-N1-C2-C5	110.03(11)	N1-C4-C7-C8	164.05(13)
Co1-N1-C4-O2	154.06(9)	N1-C6-C16-N3	29.69(19)
Co1-N1-C4-C7	32.70(14)	N1-C6-C16-C17	-153.87(14)
Co1-N1-C6-O3	-136.70(10)	N2-C7-C8-C9	-0.9(2)
Co1-N1-C6-C16	-15.00(15)	N2-C14-C15-C9	-1.0(2)
Co1-N2-C7-C4	-5.11(17)	N2-C14-C15-C10	177.92(14)
Co1-N2-C7-C8	171.79(12)	N3-C16-C17-C18	-1.6(2)
Co1-N2-C14-C13	10.0(2)	N3-C23-C24-C18	-4.9(2)
Co1-N2-C14-C15	-169.67(11)	N3-C23-C24-C19	176.73(14)
Co1-N3-C16-C6	-28.37(17)	C1-C2-C3-O2	-130.32(14)
Co1-N3-C16-C17	155.41(13)	C1-C2-C5-O3	152.07(13)
Co1-N3-C23-C22	32.4(2)	C2-N1-C4-O2	33.14(15)
Co1-N3-C23-C24	-149.14(12)	C2-N1-C4-C7	-88.22(14)
O1-C1-C2-N1	39.20(17)	C2-N1-C6-O3	-14.59(15)
O1-C1-C2-C3	155.25(13)	C2-N1-C6-C16	107.11(14)
O1-C1-C2-C5	-74.67(16)	C3-O2-C4-N1	-40.66(15)
O2-C4-C7-N2	-136.69(14)	C3-O2-C4-C7	78.84(15)
O2-C4-C7-C8	46.23(19)	C3-C2-C5-O3	-77.32(15)
O3-C6-C16-N3	149.43(14)	C4-O2-C3-C2	30.88(16)
O3-C6-C16-C17	-34.13(19)	C4-N1-C2-C1	109.14(14)
O4-S1-C25-F1	176.84(12)	C4-N1-C2-C3	-13.24(15)
O4-S1-C25-F2	57.35(14)	C4-N1-C2-C5	-130.72(13)
O4-S1-C25-F3	-63.69(14)	C4-N1-C6-O3	97.91(14)
O5-S1-O4-Co1	121.19(9)	C4-N1-C6-C16	-140.39(13)
O5-S1-C25-F1	-63.85(14)	C4-C7-C8-C9	176.01(14)
O5-S1-C25-F2	176.66(13)	C5-O3-C6-N1	36.54(15)
O5-S1-C25-F3	55.62(14)	C5-O3-C6-C16	-85.55(15)
O6-S1-O4-Co1	-15.24(11)	C5-C2-C3-O2	99.78(15)
O6-S1-C25-F1	58.01(14)	C6-O3-C5-C2	-43.35(15)
O6-S1-C25-F2	-61.48(14)	C6-N1-C2-C1	-131.52(13)
O6-S1-C25-F3	177.48(12)	C6-N1-C2-C3	106.10(13)

C6-N1-C2-C5	-11.39(15)	C17-C18-C24-C19	176.31(16)
C6-N1-C4-O2	-79.80(15)	C17-C18-C24-C23	-2.0(2)
C6-N1-C4-C7	158.84(12)	C19-C20-C21-C22	-2.6(3)
C6-C16-C17-C18	-177.81(15)	C20-C19-C24-C18	-175.76(16)
C7-N2-C14-C13	179.86(15)	C20-C19-C24-C23	2.5(2)
C7-N2-C14-C15	0.2(2)	C20-C21-C22-C23	0.3(3)
C7-C8-C9-C15	0.0(2)	C21-C22-C23-N3	-178.12(15)
C8-C9-C15-C10	-178.00(16)	C21-C22-C23-C24	3.3(2)
C8-C9-C15-C14	0.9(2)	C22-C23-C24-C18	173.62(15)
C10-C11-C12-C13	-1.2(3)	C22-C23-C24-C19	-4.7(2)
C11-C10-C15-C9	179.54(17)	C23-N3-C16-C6	171.02(13)
C11-C10-C15-C14	0.7(2)	C23-N3-C16-C17	-5.2(2)
C11-C12-C13-C14	0.1(3)	C24-C19-C20-C21	1.1(3)
C12-C13-C14-N2	-178.30(15)	C25-S1-O4-Co1	-126.87(9)
C12-C13-C14-C15	1.4(2)	O7-S2-C26-F4	171.18(16)
C13-C14-C15-C9	179.31(15)	O7-S2-C26-F5	51.30(18)
C13-C14-C15-C10	-1.8(2)	O7-S2-C26-F6	-68.5(2)
C14-N2-C7-C4	-176.11(13)	O8-S2-C26-F4	-70.69(18)
C14-N2-C7-C8	0.8(2)	O8-S2-C26-F5	169.44(16)
C15-C10-C11-C12	0.8(3)	O8-S2-C26-F6	49.6(2)
C16-N3-C23-C22	-170.11(14)	O9-S2-C26-F4	50.76(19)
C16-N3-C23-C24	8.4(2)	O9-S2-C26-F5	-69.11(18)
C16-C17-C18-C24	5.2(2)	O9-S2-C26-F6	171.07(18)

REFERENCE NUMBER: jonap34

2d

CRYSTAL STRUCTURE REPORT

$C_{30}\ H_{27}\ F_6\ N_5\ Ni\ O_9\ S_2$

or

[(κ^4-L^q)Ni(NCMe)(OTf)][OTf] · MeCN

Report prepared for:

R. Zhang, A. Panda, Prof. W. Jones

December 07, 2023

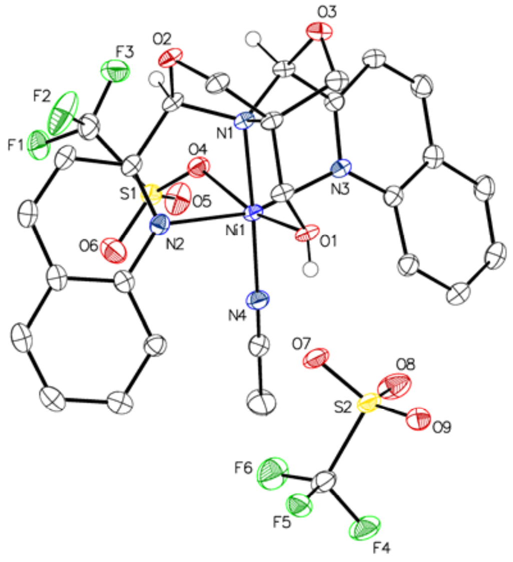

William W. Brennessel

X-ray Crystallographic Facility

Department of Chemistry, University of Rochester

120 Trustee Road

Rochester, NY 14627

Data collection

A crystal (0.151 x 0.105 x 0.018 mm^3) was placed onto a nylon loop and mounted on a Rigaku XtaLAB Synergy-S Dualflex diffractometer equipped with a HyPix-6000HE HPC area detector for data collection at 100.00(10) K. A preliminary set of cell constants and an orientation matrix were calculated from a small sampling of reflections.[1] A short pre-experiment was run, from which an optimal data collection strategy was determined. The full data collection was carried out using a PhotonJet (Cu) X-ray source with frame times of 0.47 and 1.90 seconds and a detector distance of 34.0 mm. Series of frames were collected in 0.50º steps in ω at different 2θ, κ, and ϕ settings. After the intensity data were corrected for absorption, the final cell constants were calculated from the xyz centroids of 24100 strong reflections from the actual data collection after integration.[1] See Table S-1 for additional crystal and refinement information.

Structure solution and refinement

The structure was solved using SHELXT[2] and refined using SHELXL.[3] The space group *P*-1 was determined based on intensity statistics. Most or all non-hydrogen atoms were assigned from the solution. Full-matrix least squares / difference Fourier cycles were performed which located any remaining non-hydrogen atoms. All non-hydrogen atoms were refined with anisotropic displacement parameters. The O-H hydrogen atom was found from the difference Fourier map and refined freely. All other hydrogen atoms were placed in ideal positions and refined as riding atoms with relative isotropic displacement parameters. The final full matrix least squares refinement converged to $R1 = 0.0507$ (F^2, $I > 2\sigma(I)$) and $wR2 = 0.1509$ (F^2, all data).

Structure description

The structure is the one suggested. The asymmetric unit contains one monocationic Ni complex, one triflate anion, and one acetonitrile solvent molecule, all in general positions. The cation and anion are linked via hydrogen bonding (see figure and Table S-7).

Structure manipulation and figure generation were performed using Olex2.[4] Unless noted otherwise all structural diagrams containing anisotropic displacement ellipsoids are drawn at the 50 % probability level.

Data collection, structure solution, and structure refinement were conducted at the X-ray Crystallographic Facility, B04 Hutchison Hall, Department of Chemistry, University of Rochester. The instrument was purchased with funding from NSF MRI program grant CHE-1725028. All publications arising from this report MUST either 1) include William W. Brennessel as a coauthor or 2) acknowledge William W. Brennessel and the X-ray Crystallographic Facility of the Department of Chemistry at the University of Rochester.

[1] *CrysAlisPro*, version 171.42.101a; Rigaku Corporation: Oxford, UK, 2023.

[2] Sheldrick, G. M. *SHELXT*, version 2018/2; *Acta. Crystallogr.* **2015**, *A71*, 3-8.

[3] Sheldrick, G. M. *SHELXL*, version 2019/2; *Acta. Crystallogr.* **2015**, *C71*, 3-8.

[4] Dolomanov, O. V.; Bourhis, L. J.; Gildea, R. J.; Howard, J. A. K.; Puschmann, H. *Olex2*, version 1.5; *J. Appl. Cryst.* **2009**, *42*, 339-341.

Some equations of interest:

$$R_{int} = \Sigma |F_o^2 - \langle F_o^2 \rangle| / \Sigma|F_o^2|$$

$$R1 = \Sigma||F_o|-|F_c|| / \Sigma|F_o|$$

$$wR2 = [\Sigma[w(F_o^2-F_c^2)^2] / \Sigma [w(F_o^2)^2]]^{1/2}$$

where $w = 1 / [\sigma^2 (F_o^2) + (aP)^2 + bP]$ and

$$P = 1/3 \max (0, F_o^2) + 2/3 F_c^2$$

$$\mathrm{GOF} = S = [\Sigma[w(F_o^2-F_c^2)^2] / (m-n)]^{1/2}$$

where m = number of reflections and n = number of parameters

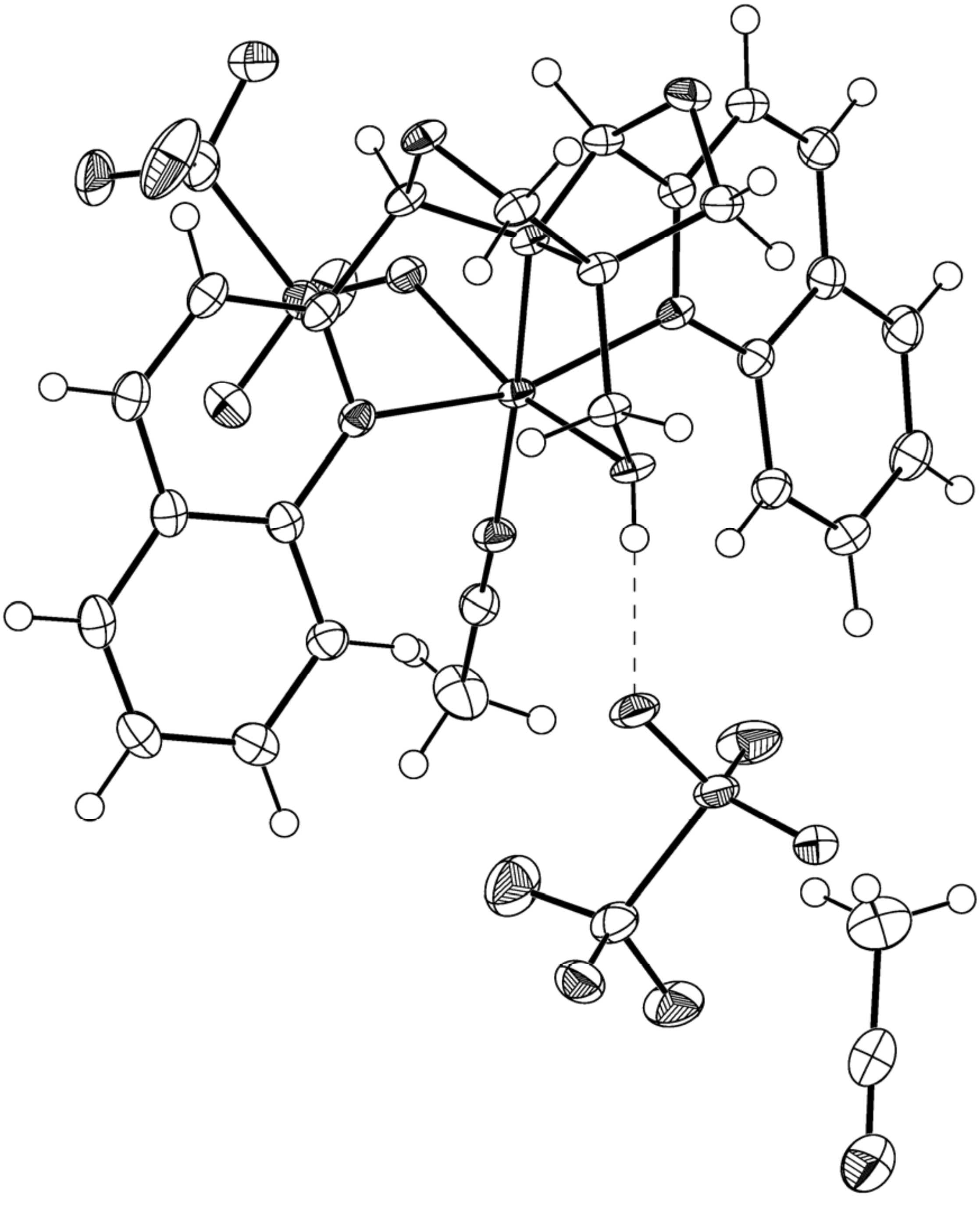

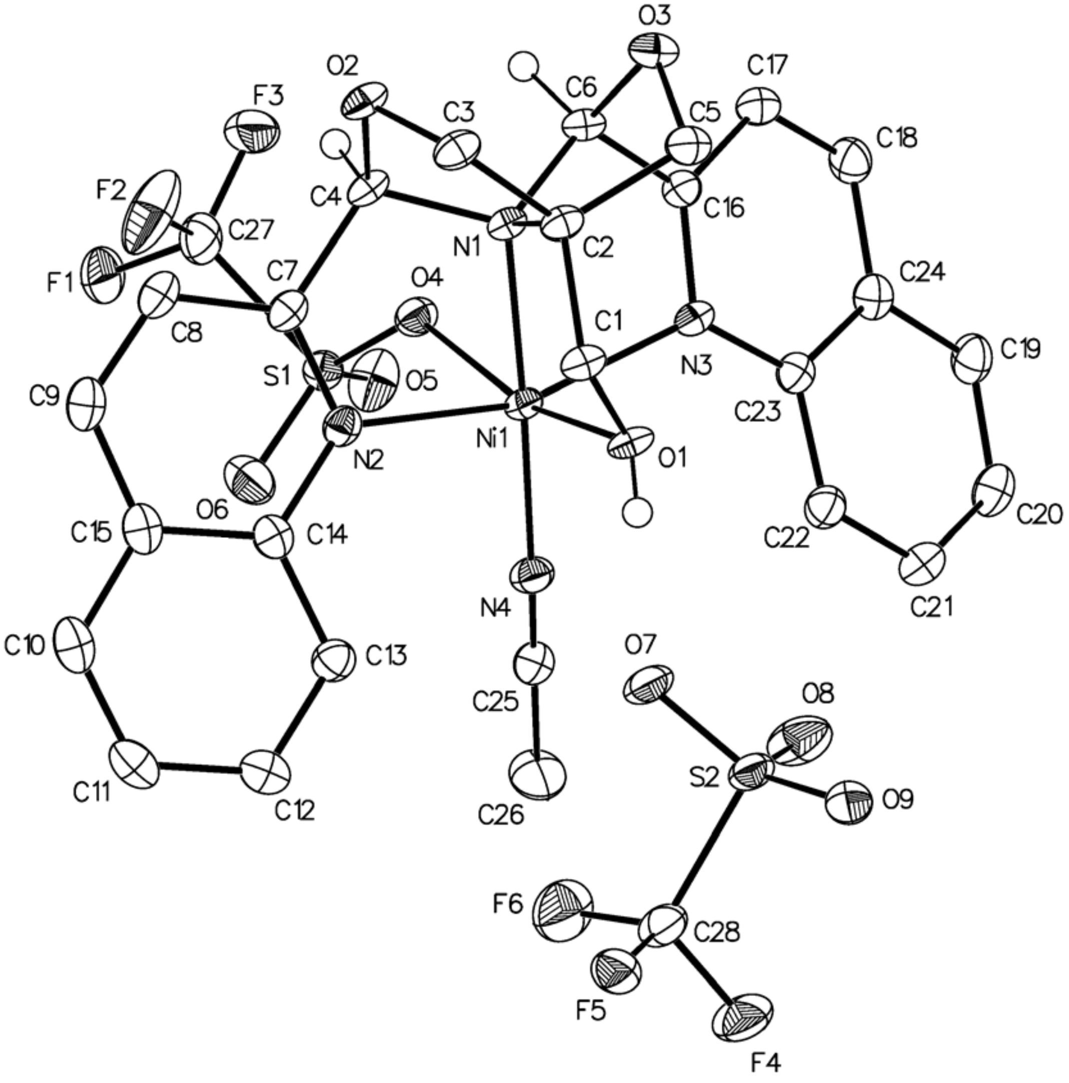
O3
O2
C17
F3
C6
C3
C5
C18
F2
C4
C16
C27
N1
C2
F1
C7
O4
C24
C8
C1
N3
C19
S1
O5
C9
C23
Ni1
N2
O1
O6
C22
C20
C15
C14
N4
C21
C10
C13
O7
C25
O8
S2
O9
C11
C12
C26
F6
C28
F5
F4

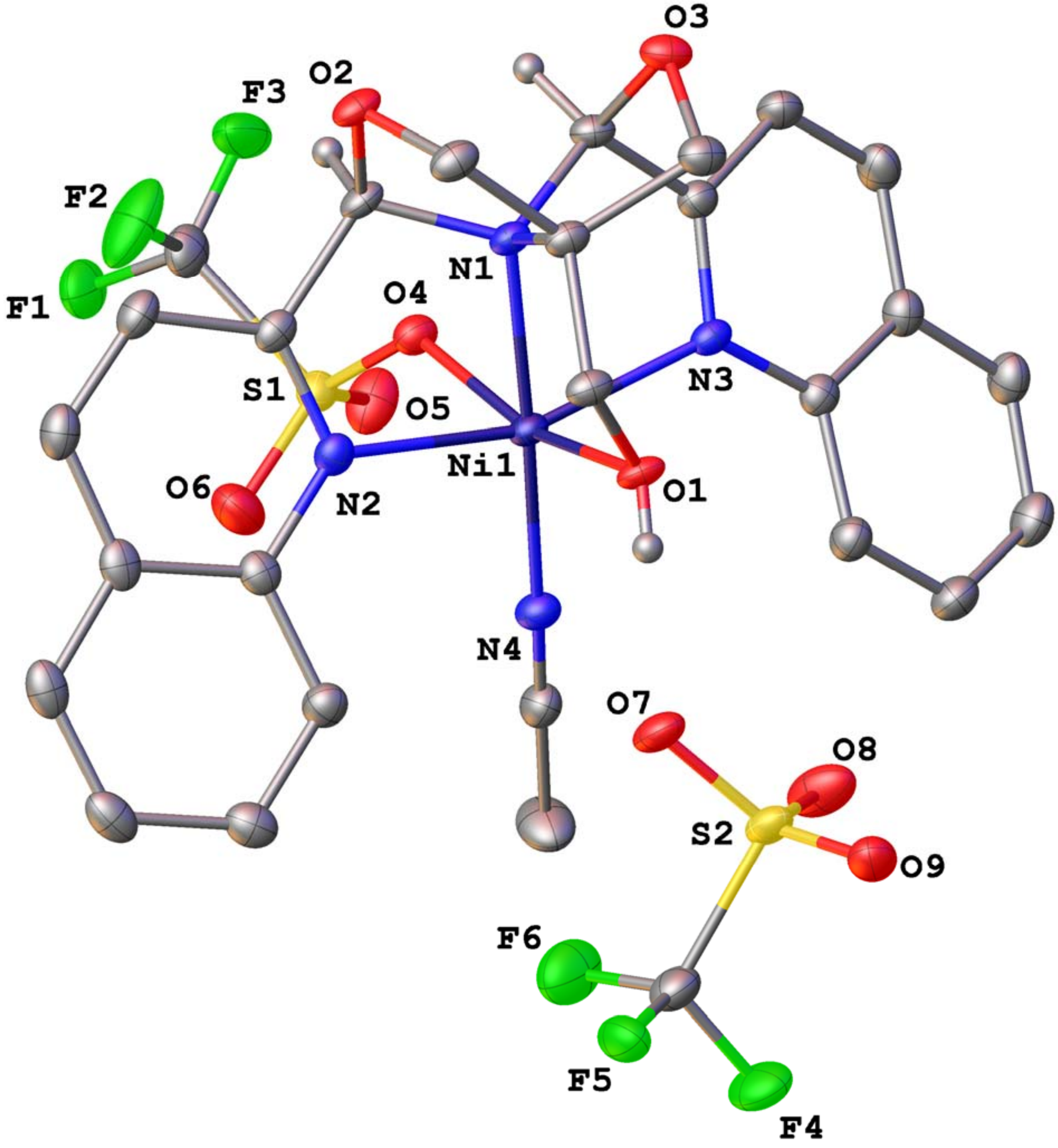
O3
O2
F3
F2
F1
N1
O4
S1
O5
N3
Ni1
O6
N2
O1
N4
O7
O8
S2
O9
F6
F5
F4

Table S-1. Crystal data and structure refinement for jonap34.

Identification code	jonap34	
Empirical formula	C30 H27 F6 N5 Ni O9 S2	
Formula weight	838.39	
Temperature	100.00(10) K	
Wavelength	1.54184 Å	
Crystal system	triclinic	
Space group	*P*-1	
Unit cell dimensions	*a* = 10.99534(17) Å	α = 106.9792(13)°
	b = 11.79005(19) Å	β = 102.6852(13)°
	c = 14.25756(16) Å	γ = 95.8656(13)°
Volume	1696.95(4) $Å^3$	
Z	2	
Density (calculated)	1.641 Mg/m^3	
Absorption coefficient	2.829 mm^{-1}	
F(000)	856	
Crystal color, morphology	colourless, block	
Crystal size	0.151 x 0.105 x 0.018 mm^3	
Theta range for data collection	3.359 to 80.425°	
Index ranges	$-13 \leq h \leq 12$, $-15 \leq k \leq 15$, $-18 \leq l \leq 18$	
Reflections collected	42063	
Independent reflections	7264 [*R*(int) = 0.0456]	
Observed reflections	6486	
Completeness to theta = 74.504°	99.8%	
Absorption correction	Multi-scan	
Max. and min. transmission	1.00000 and 0.88759	
Refinement method	Full-matrix least-squares on F^2	
Data / restraints / parameters	7264 / 0 / 484	
Goodness-of-fit on F^2	1.044	
Final *R* indices [*I*>2sigma(*I*)]	*R*1 = 0.0507, *wR*2 = 0.1479	
R indices (all data)	*R*1 = 0.0549, *wR*2 = 0.1509	
Largest diff. peak and hole	1.897 and -0.533 e.$Å^{-3}$	

Table S-2. Atomic coordinates (x 10^4) and equivalent isotropic displacement parameters ($Å^2$x 10^3) for jonap34. U_{eq} is defined as one third of the trace of the orthogonalized U_{ij} tensor.

	x	y	z	U_{eq}
Ni1	3145(1)	6268(1)	7795(1)	14(1)
S1	604(1)	7535(1)	7689(1)	18(1)
F1	-622(2)	9063(2)	8655(2)	33(1)
F2	1253(2)	9105(2)	9525(2)	50(1)
F3	989(2)	9900(2)	8337(2)	43(1)
O1	4632(2)	5342(2)	7970(1)	17(1)
O2	5243(2)	8856(2)	10420(1)	18(1)
O3	6188(2)	8815(2)	8463(2)	20(1)
O4	1962(2)	7592(2)	7791(1)	19(1)
O5	-94(2)	7592(2)	6735(2)	30(1)
O6	53(2)	6663(2)	8066(2)	35(1)
N1	4500(2)	7699(2)	8733(2)	14(1)
N2	2878(2)	6295(2)	9246(2)	16(1)
N3	3840(2)	6764(2)	6679(2)	15(1)
N4	1706(2)	4957(2)	6858(2)	19(1)
C1	5504(2)	5935(2)	8945(2)	17(1)
C2	5718(2)	7297(2)	9133(2)	16(1)
C3	6108(2)	8026(2)	10278(2)	19(1)
C4	4105(2)	8306(2)	9648(2)	16(1)
C5	6572(3)	7713(2)	8542(2)	20(1)
C6	4838(2)	8530(2)	8181(2)	16(1)
C7	3317(2)	7378(2)	9929(2)	16(1)
C8	3103(3)	7724(3)	10908(2)	21(1)
C9	2406(3)	6893(3)	11171(2)	22(1)
C10	1301(3)	4799(3)	10740(2)	22(1)
C11	937(3)	3650(3)	10068(2)	24(1)
C12	1231(3)	3360(3)	9121(2)	22(1)
C13	1871(3)	4231(2)	8850(2)	20(1)
C14	2237(2)	5430(2)	9522(2)	17(1)
C15	1971(2)	5711(3)	10488(2)	19(1)
C16	4373(2)	7925(2)	7037(2)	16(1)

C17	4588(3)	8621(2)	6417(2)	20(1)
C18	4230(3)	8093(3)	5390(2)	22(1)
C19	3389(3)	6224(3)	3912(2)	23(1)
C20	2959(3)	5005(3)	3549(2)	25(1)
C21	2899(3)	4352(3)	4226(2)	24(1)
C22	3204(3)	4923(2)	5255(2)	20(1)
C23	3586(2)	6192(2)	5648(2)	16(1)
C24	3733(3)	6845(2)	4975(2)	20(1)
C25	829(3)	4320(2)	6297(2)	21(1)
C26	-286(3)	3519(3)	5571(2)	36(1)
C27	565(3)	8990(3)	8607(2)	25(1)
S2	3923(1)	2115(1)	6740(1)	22(1)
F4	2209(2)	156(2)	5839(1)	36(1)
F5	2932(2)	524(2)	7450(1)	30(1)
F6	1620(2)	1567(2)	6897(2)	45(1)
O7	4160(2)	3022(2)	7739(2)	25(1)
O8	3426(3)	2529(2)	5910(2)	36(1)
O9	4913(2)	1422(2)	6629(2)	29(1)
C28	2599(3)	1038(3)	6736(2)	26(1)
N5	1429(4)	-1689(3)	3244(3)	46(1)
C29	1746(3)	-686(3)	3391(2)	32(1)
C30	2159(4)	583(3)	3570(3)	38(1)

Table S-3. Bond lengths [Å] and angles [°] for jonap34.

Ni(1)-O(1)	2.0660(19)	C(4)-H(4)	1.0000
Ni(1)-O(4)	2.1320(19)	C(4)-C(7)	1.525(4)
Ni(1)-N(1)	2.037(2)	C(5)-H(5A)	0.9900
Ni(1)-N(2)	2.145(2)	C(5)-H(5B)	0.9900
Ni(1)-N(3)	2.108(2)	C(6)-H(6)	1.0000
Ni(1)-N(4)	2.011(2)	C(6)-C(16)	1.520(3)
S(1)-O(4)	1.4608(19)	C(7)-C(8)	1.414(4)
S(1)-O(5)	1.433(2)	C(8)-H(8)	0.9500
S(1)-O(6)	1.433(2)	C(8)-C(9)	1.370(4)
S(1)-C(27)	1.839(3)	C(9)-H(9)	0.9500
F(1)-C(27)	1.333(3)	C(9)-C(15)	1.410(4)
F(2)-C(27)	1.321(4)	C(10)-H(10)	0.9500
F(3)-C(27)	1.316(4)	C(10)-C(11)	1.370(4)
O(1)-H(1)	0.76(4)	C(10)-C(15)	1.416(4)
O(1)-C(1)	1.432(3)	C(11)-H(11)	0.9500
O(2)-C(3)	1.436(3)	C(11)-C(12)	1.411(4)
O(2)-C(4)	1.418(3)	C(12)-H(12)	0.9500
O(3)-C(5)	1.434(3)	C(12)-C(13)	1.380(4)
O(3)-C(6)	1.427(3)	C(13)-H(13)	0.9500
N(1)-C(2)	1.514(3)	C(13)-C(14)	1.415(4)
N(1)-C(4)	1.472(3)	C(14)-C(15)	1.422(4)
N(1)-C(6)	1.490(3)	C(16)-C(17)	1.410(4)
N(2)-C(7)	1.324(3)	C(17)-H(17)	0.9500
N(2)-C(14)	1.378(3)	C(17)-C(18)	1.360(4)
N(3)-C(16)	1.332(3)	C(18)-H(18)	0.9500
N(3)-C(23)	1.377(3)	C(18)-C(24)	1.414(4)
N(4)-C(25)	1.138(4)	C(19)-H(19)	0.9500
C(1)-H(1A)	0.9900	C(19)-C(20)	1.369(4)
C(1)-H(1B)	0.9900	C(19)-C(24)	1.421(4)
C(1)-C(2)	1.534(3)	C(20)-H(20)	0.9500
C(2)-C(3)	1.544(3)	C(20)-C(21)	1.408(4)
C(2)-C(5)	1.526(4)	C(21)-H(21)	0.9500
C(3)-H(3A)	0.9900	C(21)-C(22)	1.370(4)
C(3)-H(3B)	0.9900	C(22)-H(22)	0.9500

C(22)-C(23)	1.417(4)	O(5)-S(1)-C(27)	103.91(14)
C(23)-C(24)	1.419(4)	O(6)-S(1)-O(4)	114.26(13)
C(25)-C(26)	1.450(4)	O(6)-S(1)-C(27)	103.86(14)
C(26)-H(26A)	0.9800	Ni(1)-O(1)-H(1)	119(3)
C(26)-H(26B)	0.9800	C(1)-O(1)-Ni(1)	109.41(14)
C(26)-H(26C)	0.9800	C(1)-O(1)-H(1)	110(3)
S(2)-O(7)	1.460(2)	C(4)-O(2)-C(3)	106.33(18)
S(2)-O(8)	1.431(2)	C(6)-O(3)-C(5)	103.72(19)
S(2)-O(9)	1.435(2)	S(1)-O(4)-Ni(1)	132.14(12)
S(2)-C(28)	1.829(3)	C(2)-N(1)-Ni(1)	111.39(15)
F(4)-C(28)	1.339(3)	C(4)-N(1)-Ni(1)	110.48(15)
F(5)-C(28)	1.333(4)	C(4)-N(1)-C(2)	104.66(19)
F(6)-C(28)	1.328(4)	C(4)-N(1)-C(6)	113.05(19)
N(5)-C(29)	1.141(5)	C(6)-N(1)-Ni(1)	111.10(15)
C(29)-C(30)	1.449(5)	C(6)-N(1)-C(2)	105.91(19)
C(30)-H(30A)	0.9800	C(7)-N(2)-Ni(1)	110.87(17)
C(30)-H(30B)	0.9800	C(7)-N(2)-C(14)	118.3(2)
C(30)-H(30C)	0.9800	C(14)-N(2)-Ni(1)	130.46(18)
O(1)-Ni(1)-O(4)	166.25(8)	C(16)-N(3)-Ni(1)	110.06(16)
O(1)-Ni(1)-N(2)	91.39(8)	C(16)-N(3)-C(23)	117.6(2)
O(1)-Ni(1)-N(3)	87.60(8)	C(23)-N(3)-Ni(1)	130.54(17)
O(4)-Ni(1)-N(2)	88.51(8)	C(25)-N(4)-Ni(1)	171.9(2)
N(1)-Ni(1)-O(1)	82.43(8)	O(1)-C(1)-H(1A)	109.9
N(1)-Ni(1)-O(4)	84.03(8)	O(1)-C(1)-H(1B)	109.9
N(1)-Ni(1)-N(2)	79.36(9)	O(1)-C(1)-C(2)	108.9(2)
N(1)-Ni(1)-N(3)	81.60(8)	H(1A)-C(1)-H(1B)	108.3
N(3)-Ni(1)-O(4)	88.01(8)	C(2)-C(1)-H(1A)	109.9
N(3)-Ni(1)-N(2)	160.90(9)	C(2)-C(1)-H(1B)	109.9
N(4)-Ni(1)-O(1)	102.48(8)	N(1)-C(2)-C(1)	111.2(2)
N(4)-Ni(1)-O(4)	91.03(8)	N(1)-C(2)-C(3)	103.62(19)
N(4)-Ni(1)-N(1)	175.06(9)	N(1)-C(2)-C(5)	101.38(19)
N(4)-Ni(1)-N(2)	100.82(9)	C(1)-C(2)-C(3)	112.0(2)
N(4)-Ni(1)-N(3)	98.02(9)	C(5)-C(2)-C(1)	114.2(2)
O(4)-S(1)-C(27)	101.73(12)	C(5)-C(2)-C(3)	113.4(2)
O(5)-S(1)-O(4)	113.97(12)	O(2)-C(3)-C(2)	105.90(19)
O(5)-S(1)-O(6)	116.65(15)	O(2)-C(3)-H(3A)	110.6

O(2)-C(3)-H(3B)	110.6	C(12)-C(11)-H(11)	119.8
C(2)-C(3)-H(3A)	110.6	C(11)-C(12)-H(12)	119.7
C(2)-C(3)-H(3B)	110.6	C(13)-C(12)-C(11)	120.6(3)
H(3A)-C(3)-H(3B)	108.7	C(13)-C(12)-H(12)	119.7
O(2)-C(4)-N(1)	105.7(2)	C(12)-C(13)-H(13)	120.0
O(2)-C(4)-H(4)	110.1	C(12)-C(13)-C(14)	120.1(3)
O(2)-C(4)-C(7)	111.2(2)	C(14)-C(13)-H(13)	120.0
N(1)-C(4)-H(4)	110.1	N(2)-C(14)-C(13)	119.7(2)
N(1)-C(4)-C(7)	109.5(2)	N(2)-C(14)-C(15)	121.1(2)
C(7)-C(4)-H(4)	110.1	C(13)-C(14)-C(15)	119.1(2)
O(3)-C(5)-C(2)	103.5(2)	C(9)-C(15)-C(10)	122.2(3)
O(3)-C(5)-H(5A)	111.1	C(9)-C(15)-C(14)	118.4(2)
O(3)-C(5)-H(5B)	111.1	C(10)-C(15)-C(14)	119.4(3)
C(2)-C(5)-H(5A)	111.1	N(3)-C(16)-C(6)	118.5(2)
C(2)-C(5)-H(5B)	111.1	N(3)-C(16)-C(17)	123.9(2)
H(5A)-C(5)-H(5B)	109.0	C(17)-C(16)-C(6)	117.5(2)
O(3)-C(6)-N(1)	106.09(19)	C(16)-C(17)-H(17)	120.5
O(3)-C(6)-H(6)	110.1	C(18)-C(17)-C(16)	118.9(3)
O(3)-C(6)-C(16)	109.0(2)	C(18)-C(17)-H(17)	120.5
N(1)-C(6)-H(6)	110.1	C(17)-C(18)-H(18)	120.4
N(1)-C(6)-C(16)	111.3(2)	C(17)-C(18)-C(24)	119.2(3)
C(16)-C(6)-H(6)	110.1	C(24)-C(18)-H(18)	120.4
N(2)-C(7)-C(4)	118.2(2)	C(20)-C(19)-H(19)	119.9
N(2)-C(7)-C(8)	123.8(3)	C(20)-C(19)-C(24)	120.1(3)
C(8)-C(7)-C(4)	118.0(2)	C(24)-C(19)-H(19)	119.9
C(7)-C(8)-H(8)	120.8	C(19)-C(20)-H(20)	119.8
C(9)-C(8)-C(7)	118.4(3)	C(19)-C(20)-C(21)	120.3(3)
C(9)-C(8)-H(8)	120.8	C(21)-C(20)-H(20)	119.8
C(8)-C(9)-H(9)	120.1	C(20)-C(21)-H(21)	119.5
C(8)-C(9)-C(15)	119.8(2)	C(22)-C(21)-C(20)	121.0(3)
C(15)-C(9)-H(9)	120.1	C(22)-C(21)-H(21)	119.5
C(11)-C(10)-H(10)	119.9	C(21)-C(22)-H(22)	120.1
C(11)-C(10)-C(15)	120.3(3)	C(21)-C(22)-C(23)	119.8(3)
C(15)-C(10)-H(10)	119.9	C(23)-C(22)-H(22)	120.1
C(10)-C(11)-H(11)	119.8	N(3)-C(23)-C(22)	119.4(2)
C(10)-C(11)-C(12)	120.4(3)	N(3)-C(23)-C(24)	121.3(2)

C(22)-C(23)-C(24)	119.3(2)	O(8)-S(2)-O(7)	114.14(13)
C(18)-C(24)-C(19)	122.3(3)	O(8)-S(2)-O(9)	116.09(14)
C(18)-C(24)-C(23)	118.6(2)	O(8)-S(2)-C(28)	103.99(15)
C(23)-C(24)-C(19)	119.2(3)	O(9)-S(2)-O(7)	114.06(14)
N(4)-C(25)-C(26)	179.2(3)	O(9)-S(2)-C(28)	104.03(13)
C(25)-C(26)-H(26A)	109.5	F(4)-C(28)-S(2)	110.5(2)
C(25)-C(26)-H(26B)	109.5	F(5)-C(28)-S(2)	111.4(2)
C(25)-C(26)-H(26C)	109.5	F(5)-C(28)-F(4)	107.5(2)
H(26A)-C(26)-H(26B)	109.5	F(6)-C(28)-S(2)	111.3(2)
H(26A)-C(26)-H(26C)	109.5	F(6)-C(28)-F(4)	107.9(3)
H(26B)-C(26)-H(26C)	109.5	F(6)-C(28)-F(5)	108.1(2)
F(1)-C(27)-S(1)	109.8(2)	N(5)-C(29)-C(30)	179.3(4)
F(2)-C(27)-S(1)	110.9(2)	C(29)-C(30)-H(30A)	109.5
F(2)-C(27)-F(1)	108.0(2)	C(29)-C(30)-H(30B)	109.5
F(3)-C(27)-S(1)	111.4(2)	C(29)-C(30)-H(30C)	109.5
F(3)-C(27)-F(1)	107.7(2)	H(30A)-C(30)-H(30B)	109.5
F(3)-C(27)-F(2)	108.8(3)	H(30A)-C(30)-H(30C)	109.5
O(7)-S(2)-C(28)	102.29(13)	H(30B)-C(30)-H(30C)	109.5

Table S-4. Anisotropic displacement parameters ($Å^2 x 10^3$) for jonap34. The anisotropic displacement factor exponent takes the form: $-2\pi^2[h^2 a^{*2}U_{11} + ... + 2 h k a^* b^* U_{12}]$

	U_{11}	U_{22}	U_{33}	U_{23}	U_{13}	U_{12}
Ni1	16(1)	11(1)	13(1)	2(1)	2(1)	1(1)
S1	17(1)	18(1)	18(1)	4(1)	5(1)	2(1)
F1	23(1)	36(1)	42(1)	6(1)	17(1)	8(1)
F2	48(1)	66(2)	23(1)	-8(1)	1(1)	28(1)
F3	43(1)	20(1)	70(2)	7(1)	33(1)	3(1)
O1	20(1)	9(1)	18(1)	1(1)	1(1)	2(1)
O2	19(1)	13(1)	17(1)	-2(1)	-1(1)	2(1)
O3	17(1)	15(1)	25(1)	6(1)	2(1)	0(1)
O4	19(1)	16(1)	21(1)	4(1)	5(1)	4(1)
O5	25(1)	41(1)	22(1)	5(1)	1(1)	12(1)
O6	36(1)	28(1)	48(1)	15(1)	23(1)	4(1)
N1	16(1)	11(1)	14(1)	2(1)	2(1)	2(1)
N2	18(1)	16(1)	16(1)	6(1)	5(1)	6(1)
N3	14(1)	12(1)	15(1)	2(1)	2(1)	3(1)
N4	21(1)	15(1)	18(1)	3(1)	3(1)	1(1)
C1	16(1)	13(1)	18(1)	3(1)	0(1)	3(1)
C2	16(1)	12(1)	17(1)	3(1)	1(1)	4(1)
C3	18(1)	16(1)	18(1)	2(1)	-1(1)	4(1)
C4	18(1)	12(1)	13(1)	-1(1)	1(1)	3(1)
C5	18(1)	17(1)	23(1)	6(1)	4(1)	3(1)
C6	16(1)	11(1)	18(1)	3(1)	1(1)	0(1)
C7	15(1)	16(1)	16(1)	3(1)	2(1)	6(1)
C8	22(1)	21(1)	15(1)	2(1)	4(1)	7(1)
C9	22(1)	28(1)	17(1)	8(1)	7(1)	10(1)
C10	18(1)	34(2)	23(1)	16(1)	8(1)	12(1)
C11	18(1)	29(2)	32(2)	19(1)	7(1)	6(1)
C12	19(1)	22(1)	27(1)	11(1)	3(1)	3(1)
C13	22(1)	18(1)	20(1)	6(1)	5(1)	5(1)
C14	14(1)	21(1)	18(1)	8(1)	3(1)	4(1)
C15	14(1)	27(1)	19(1)	11(1)	4(1)	9(1)
C16	17(1)	15(1)	18(1)	4(1)	5(1)	3(1)

C17	24(1)	14(1)	23(1)	6(1)	7(1)	2(1)
C18	28(1)	22(1)	22(1)	11(1)	11(1)	7(1)
C19	28(1)	26(1)	17(1)	8(1)	8(1)	7(1)
C20	28(1)	28(1)	16(1)	3(1)	6(1)	7(1)
C21	27(1)	18(1)	22(1)	2(1)	7(1)	3(1)
C22	23(1)	19(1)	18(1)	5(1)	6(1)	4(1)
C23	15(1)	18(1)	16(1)	5(1)	3(1)	4(1)
C24	21(1)	22(1)	18(1)	7(1)	6(1)	6(1)
C25	24(1)	20(1)	18(1)	4(1)	7(1)	2(1)
C26	28(2)	42(2)	25(2)	0(1)	1(1)	-12(1)
C27	18(1)	29(2)	27(1)	6(1)	8(1)	6(1)
S2	34(1)	12(1)	20(1)	4(1)	9(1)	4(1)
F4	44(1)	26(1)	25(1)	-3(1)	2(1)	-4(1)
F5	40(1)	24(1)	25(1)	8(1)	11(1)	-3(1)
F6	30(1)	42(1)	56(1)	3(1)	12(1)	9(1)
O7	37(1)	13(1)	22(1)	1(1)	7(1)	2(1)
O8	59(2)	21(1)	27(1)	10(1)	5(1)	7(1)
O9	37(1)	19(1)	37(1)	9(1)	19(1)	6(1)
C28	29(2)	19(1)	23(1)	0(1)	5(1)	2(1)
N5	62(2)	37(2)	43(2)	7(1)	27(2)	11(2)
C29	32(2)	36(2)	27(2)	6(1)	9(1)	13(1)
C30	40(2)	34(2)	36(2)	9(1)	1(1)	7(1)

Table S-5. Hydrogen coordinates (x 10^4) and isotropic displacement parameters ($Å^2$ x 10^3) for jonap34.

	x	y	z	U(eq)
H1	4490(30)	4670(40)	7880(30)	18(8)
H1A	6319	5641	8965	20
H1B	5158	5754	9481	20
H3A	6044	7485	10688	22
H3B	6991	8466	10484	22
H4	3602	8932	9522	19
H5A	7476	7854	8914	24
H5B	6437	7111	7861	24
H6	4475	9279	8391	19
H8	3435	8514	11371	25
H9	2217	7113	11812	26
H10	1103	4986	11378	27
H11	484	3045	10241	28
H12	985	2557	8665	27
H13	2067	4026	8211	24
H17	4977	9446	6711	24
H18	4312	8558	4956	27
H19	3457	6656	3452	27
H20	2700	4599	2837	30
H21	2642	3501	3966	28
H22	3161	4470	5702	24
H26A	-71	3115	4939	55
H26B	-951	3985	5430	55
H26C	-590	2914	5853	55
H30A	2547	678	3035	58
H30B	2780	936	4232	58
H30C	1428	995	3565	58

Table S-6. Torsion angles [°] for jonap34.

Ni1-O1-C1-C2	-44.2(2)	N1-C2-C3-O2	-7.2(3)
Ni1-N1-C2-C1	-14.1(2)	N1-C2-C5-O3	35.3(2)
Ni1-N1-C2-C3	-134.57(17)	N1-C4-C7-N2	-14.5(3)
Ni1-N1-C2-C5	107.70(18)	N1-C4-C7-C8	165.2(2)
Ni1-N1-C4-O2	153.01(15)	N1-C6-C16-N3	-5.0(3)
Ni1-N1-C4-C7	33.2(2)	N1-C6-C16-C17	177.8(2)
Ni1-N1-C6-O3	-134.43(16)	N2-C7-C8-C9	0.3(4)
Ni1-N1-C6-C16	-16.0(2)	N2-C14-C15-C9	-1.5(4)
Ni1-N2-C7-C4	-10.4(3)	N2-C14-C15-C10	179.5(2)
Ni1-N2-C7-C8	169.9(2)	N3-C16-C17-C18	0.4(4)
Ni1-N2-C14-C13	14.2(4)	N3-C23-C24-C18	4.1(4)
Ni1-N2-C14-C15	-167.98(18)	N3-C23-C24-C19	-176.4(2)
Ni1-N3-C16-C6	22.5(3)	C1-C2-C3-O2	-127.1(2)
Ni1-N3-C16-C17	-160.5(2)	C1-C2-C5-O3	155.0(2)
Ni1-N3-C23-C22	-26.9(3)	C2-N1-C4-O2	33.0(2)
Ni1-N3-C23-C24	155.0(2)	C2-N1-C4-C7	-86.8(2)
O1-C1-C2-N1	38.4(3)	C2-N1-C6-O3	-13.3(2)
O1-C1-C2-C3	153.8(2)	C2-N1-C6-C16	105.1(2)
O1-C1-C2-C5	-75.6(3)	C3-O2-C4-N1	-38.6(2)
O2-C4-C7-N2	-130.9(2)	C3-O2-C4-C7	80.2(2)
O2-C4-C7-C8	48.8(3)	C3-C2-C5-O3	-75.1(2)
O3-C6-C16-N3	111.7(2)	C4-O2-C3-C2	28.0(3)
O3-C6-C16-C17	-65.5(3)	C4-N1-C2-C1	105.3(2)
O4-S1-C27-F1	-176.5(2)	C4-N1-C2-C3	-15.2(2)
O4-S1-C27-F2	-57.1(2)	C4-N1-C2-C5	-132.9(2)
O4-S1-C27-F3	64.3(2)	C4-N1-C6-O3	100.7(2)
O5-S1-O4-Ni1	-108.04(17)	C4-N1-C6-C16	-140.8(2)
O5-S1-C27-F1	64.9(2)	C4-C7-C8-C9	-179.4(2)
O5-S1-C27-F2	-175.8(2)	C5-O3-C6-N1	36.4(2)
O5-S1-C27-F3	-54.4(2)	C5-O3-C6-C16	-83.5(2)
O6-S1-O4-Ni1	29.6(2)	C5-C2-C3-O2	101.9(2)
O6-S1-C27-F1	-57.6(2)	C6-O3-C5-C2	-45.1(2)
O6-S1-C27-F2	61.8(2)	C6-N1-C2-C1	-135.0(2)
O6-S1-C27-F3	-176.8(2)	C6-N1-C2-C3	104.5(2)

C6-N1-C2-C5	-13.2(2)	C17-C18-C24-C19	-177.4(3)
C6-N1-C4-O2	-81.8(2)	C17-C18-C24-C23	2.1(4)
C6-N1-C4-C7	158.4(2)	C19-C20-C21-C22	3.4(4)
C6-C16-C17-C18	177.4(2)	C20-C19-C24-C18	177.4(3)
C7-N2-C14-C13	-173.4(2)	C20-C19-C24-C23	-2.0(4)
C7-N2-C14-C15	4.4(4)	C20-C21-C22-C23	0.1(4)
C7-C8-C9-C15	2.8(4)	C21-C22-C23-N3	177.3(2)
C8-C9-C15-C10	176.8(2)	C21-C22-C23-C24	-4.5(4)
C8-C9-C15-C14	-2.2(4)	C22-C23-C24-C18	-174.0(2)
C10-C11-C12-C13	-1.0(4)	C22-C23-C24-C19	5.5(4)
C11-C10-C15-C9	-177.5(3)	C23-N3-C16-C6	-171.3(2)
C11-C10-C15-C14	1.5(4)	C23-N3-C16-C17	5.7(4)
C11-C12-C13-C14	-0.2(4)	C24-C19-C20-C21	-2.4(4)
C12-C13-C14-N2	179.9(2)	C27-S1-O4-Ni1	140.80(16)
C12-C13-C14-C15	2.0(4)	O7-S2-C28-F4	177.0(2)
C13-C14-C15-C9	176.4(2)	O7-S2-C28-F5	-63.7(2)
C13-C14-C15-C10	-2.6(4)	O7-S2-C28-F6	57.1(2)
C14-N2-C7-C4	175.7(2)	O8-S2-C28-F4	57.9(2)
C14-N2-C7-C8	-3.9(4)	O8-S2-C28-F5	177.30(19)
C15-C10-C11-C12	0.4(4)	O8-S2-C28-F6	-61.9(2)
C16-N3-C23-C22	170.3(2)	O9-S2-C28-F4	-64.0(2)
C16-N3-C23-C24	-7.8(4)	O9-S2-C28-F5	55.3(2)
C16-C17-C18-C24	-4.3(4)	O9-S2-C28-F6	176.1(2)

Table S-7. Hydrogen bonds and close contacts for jonap34 [Å and °].

D-H...A	d(D-H)	d(H...A)	d(D...A)	<(DHA)
O1-H1...O7	0.76(4)	1.88(4)	2.640(3)	177(4)

REFERENCE NUMBER: jonap26

2e

CRYSTAL STRUCTURE REPORT

$C_{30.23} H_{27.33} Cu F_6 N_{5.11} O_9 S_2$

or

$[(\kappa^4\text{-}L^q)Cu(NCMe)][OTf]_2 \cdot 1.11\ MeCN$

Report prepared for:

R. Zhang, A. Panda, Prof. W. Jones

October 03, 2023

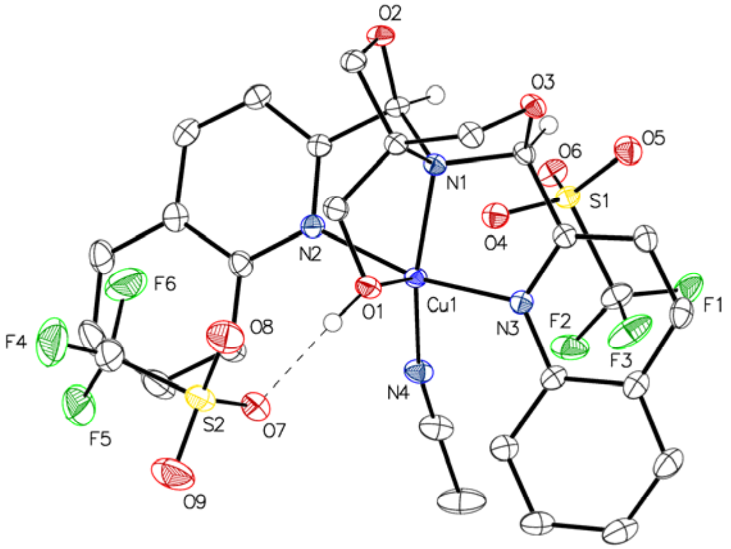

William W. Brennessel
X-ray Crystallographic Facility
Department of Chemistry, University of Rochester
120 Trustee Road
Rochester, NY 14627

Data collection

A crystal (0.366 x 0.11 x 0.03 mm^3) was placed onto a nylon loop and mounted on a Rigaku XtaLAB Synergy-S Dualflex diffractometer equipped with a HyPix-6000HE HPC area detector for data collection at 100.00(10) K. A preliminary set of cell constants and an orientation matrix were calculated from a small sampling of reflections.[1] A short pre-experiment was run, from which an optimal data collection strategy was determined. The full data collection was carried out using a PhotonJet (Cu) X-ray source with frame times of 0.08 and 0.32 seconds and a detector distance of 34.0 mm. Series of frames were collected in 0.50º steps in ω at different 2θ, κ, and ϕ settings. After the intensity data were corrected for absorption, the final cell constants were calculated from the xyz centroids of 35271 strong reflections from the actual data collection after integration.[1] See Table S-1 for additional crystal and refinement information.

Structure solution and refinement

The structure was solved using SHELXT[2] and refined using SHELXL.[3] The space group *P*-1 was determined based on intensity statistics. Most or all non-hydrogen atoms were assigned from the solution. Full-matrix least squares / difference Fourier cycles were performed which located any remaining non-hydrogen atoms. All non-hydrogen atoms were refined with anisotropic displacement parameters. The O-H hydrogen atom was found from the difference Fourier map and refined freely. All other hydrogen atoms were placed in ideal positions and refined as riding atoms with relative isotropic displacement parameters. The final full matrix least squares refinement converged to $R1 = 0.0312$ (F^2, $I > 2\sigma(I)$) and $wR2 = 0.0815$ (F^2, all data).

Structure description

The structure is the one suggested. The asymmetric unit contains one copper complex, two triflate anions, and one acetonitrile solvent molecule of crystallization in general positions, and one partially-occupied (0.11) acetonitrile molecule of crystallization on a crystallographic inversion center. The last is modeled as disordered over the center (0.50:0.50). The Cu1…O4 distance is 2.5788(11) Å. Hydrogen bonding (O-H…O) links the cation to the second triflate anion (see figure and Table S-7).

Structure manipulation and figure generation were performed using Olex2.[4] Unless noted otherwise all structural diagrams containing anisotropic displacement ellipsoids are drawn at the 50 % probability level.

Data collection, structure solution, and structure refinement were conducted at the X-ray Crystallographic Facility, B04 Hutchison Hall, Department of Chemistry, University of Rochester. The instrument was purchased with funding from NSF MRI program grant CHE-1725028. All publications arising from this report MUST either 1) include William W. Brennessel as a coauthor or 2) acknowledge William W. Brennessel and the X-ray Crystallographic Facility of the Department of Chemistry at the University of Rochester.

[1] *CrysAlisPro*, version 171.42.101a; Rigaku Corporation: Oxford, UK, 2023.

[2] Sheldrick, G. M. *SHELXT*, version 2018/2; *Acta. Crystallogr.* **2015**, *A71*, 3-8.

[3] Sheldrick, G. M. *SHELXL*, version 2019/2; *Acta. Crystallogr.* **2015**, *C71*, 3-8.

[4] Dolomanov, O. V.; Bourhis, L. J.; Gildea, R. J.; Howard, J. A. K.; Puschmann, H. *Olex2*, version 1.5; *J. Appl. Cryst.* **2009**, *42*, 339-341.

Some equations of interest:

$$R_{int} = \Sigma |F_o^2 - \langle F_o^2 \rangle| / \Sigma|F_o^2|$$

$$R1 = \Sigma||F_o|-|F_c|| / \Sigma|F_o|$$

$$wR2 = [\Sigma[w(F_o^2-F_c^2)^2] / \Sigma [w(F_o^2)^2]]^{1/2}$$

where $w = 1 / [\sigma^2 (F_o^2) + (aP)^2 + bP]$ and

$$P = 1/3 \max (0, F_o^2) + 2/3 F_c^2$$

$$\mathrm{GOF} = S = [\Sigma[w(F_o^2-F_c^2)^2] / (m-n)]^{1/2}$$

where m = number of reflections and n = number of parameters

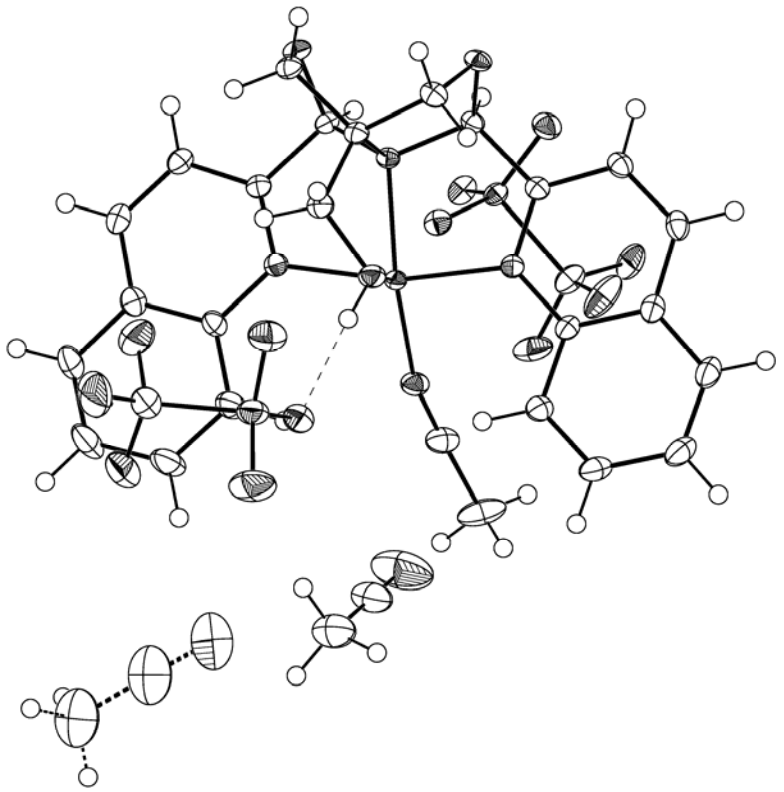

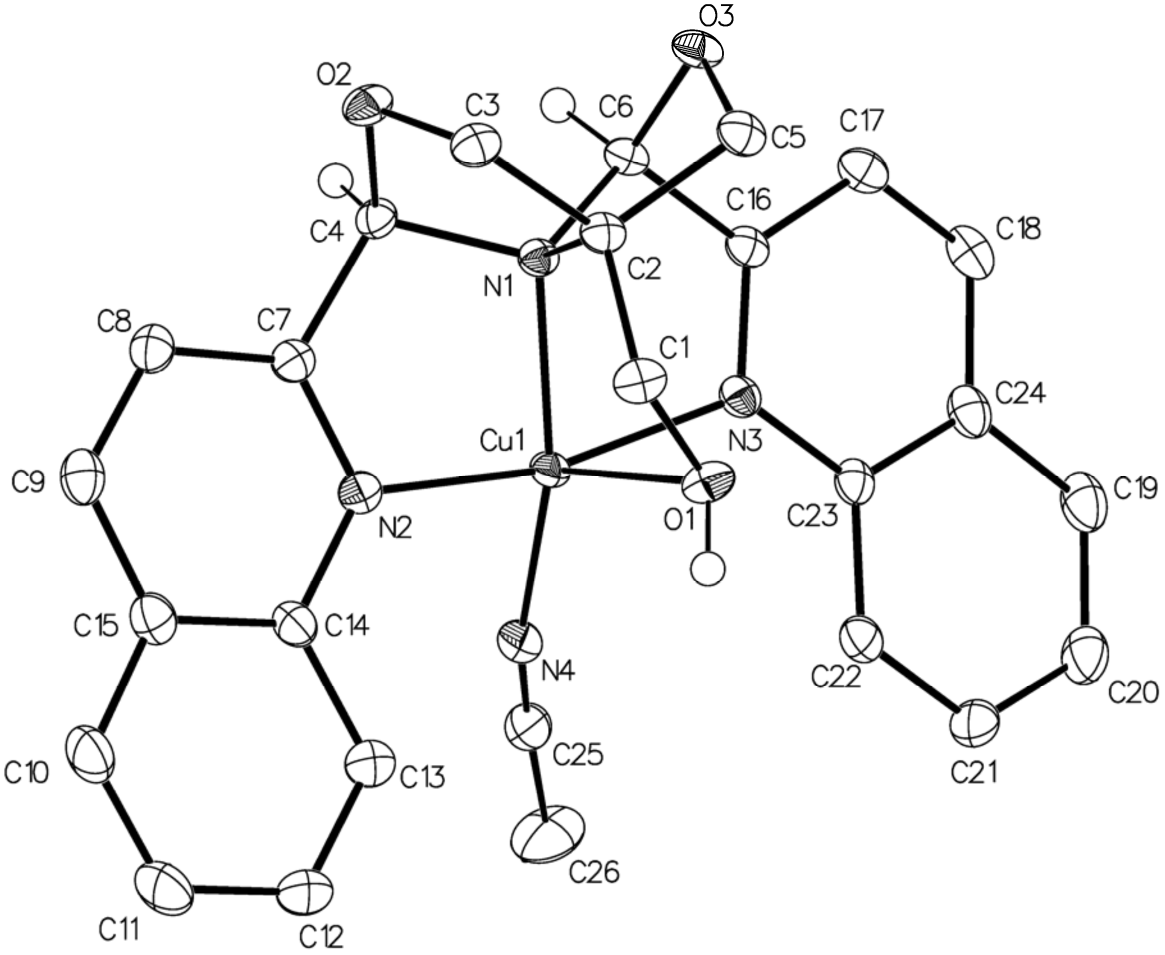
O3
O2
C3
C6
C5
C17
C16
C4
C2
C18
N1
C8
C7
C1
C24
Cu1
N3
C9
C19
N2
O1
C23
C15
C14
N4
C22
C20
C10
C13
C25
C21
C26
C11
C12

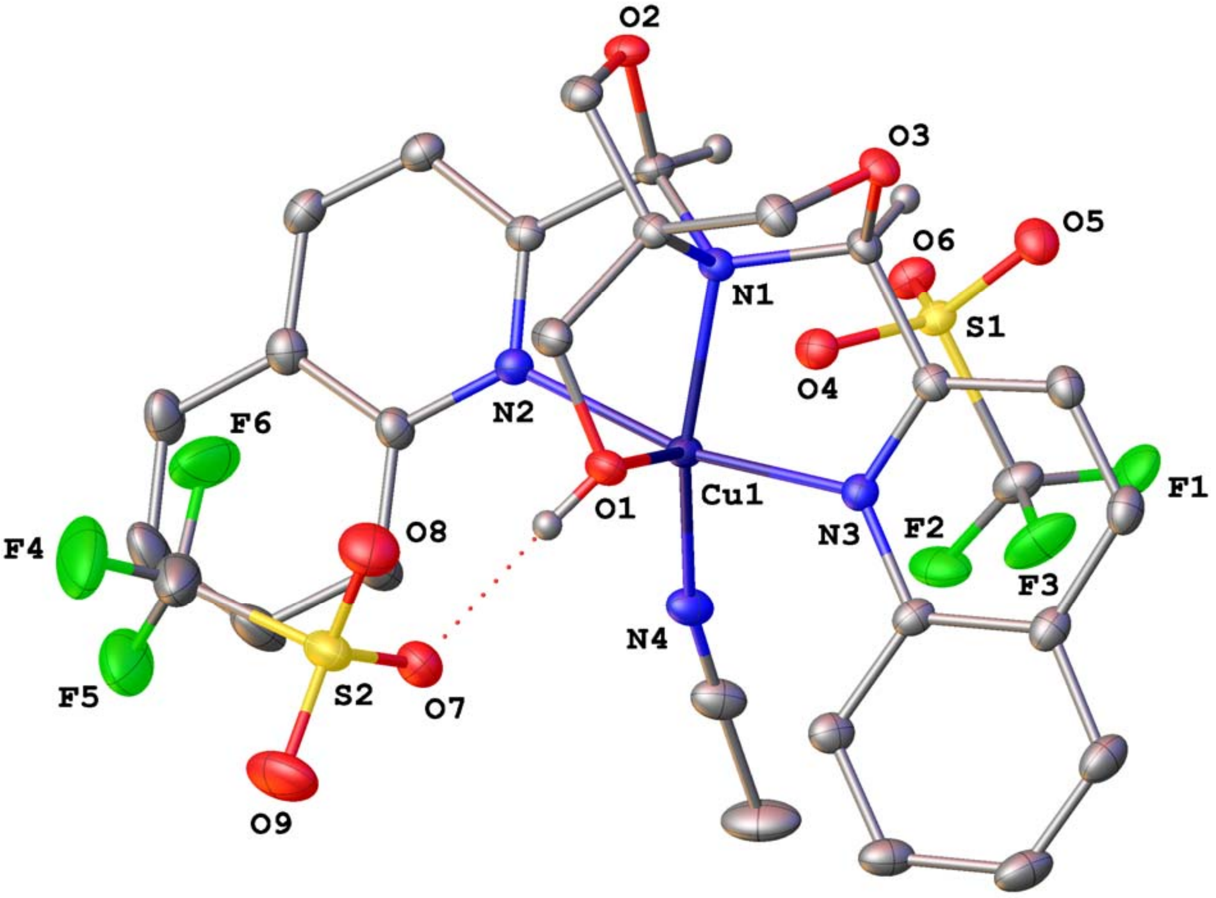
O2
O3
O5
O6
N1
S1
O4
F6
N2
F1
O1
Cu1
N3
F2
F4
O8
F3
N4
F5
S2
O7
O9

Table S-1. Crystal data and structure refinement for jonap26.

Identification code	jonap26	
Empirical formula	C30.23 H27.33 Cu F6 N5.11 O9 S2	
Formula weight	847.81	
Temperature	100.00(10) K	
Wavelength	1.54184 Å	
Crystal system	triclinic	
Space group	*P*-1	
Unit cell dimensions	*a* = 8.96914(8) Å	α = 81.3678(7)°
	b = 10.25163(8) Å	β = 79.9068(7)°
	c = 21.22441(16) Å	γ = 65.0171(8)°
Volume	1735.23(3) $Å^3$	
Z	2	
Density (calculated)	1.623 Mg/m^3	
Absorption coefficient	2.853 mm^{-1}	
F(000)	863	
Crystal color, morphology	blue, needle	
Crystal size	0.366 x 0.11 x 0.03 mm^3	
Theta range for data collection	4.247 to 80.089°	
Index ranges	$-11 \le h \le 10$, $-13 \le k \le 13$, $-27 \le l \le 26$	
Reflections collected	56639	
Independent reflections	7426 [*R*(int) = 0.0487]	
Observed reflections	6887	
Completeness to theta = 74.504°	99.7%	
Absorption correction	Multi-scan	
Max. and min. transmission	1.00000 and 0.50957	
Refinement method	Full-matrix least-squares on F^2	
Data / restraints / parameters	7426 / 15 / 513	
Goodness-of-fit on F^2	1.044	
Final *R* indices [*I*>2sigma(*I*)]	*R*1 = 0.0312, *wR*2 = 0.0802	
R indices (all data)	*R*1 = 0.0336, *wR*2 = 0.0815	
Largest diff. peak and hole	0.508 and -0.663 e.$Å^{-3}$	

Table S-2. Atomic coordinates (x 10^4) and equivalent isotropic displacement parameters ($Å^2$x 10^3) for jonap26. U_{eq} is defined as one third of the trace of the orthogonalized U_{ij} tensor.

	x	y	z	U_{eq}
Cu1	3333(1)	3070(1)	2008(1)	14(1)
O1	2956(2)	908(1)	2358(1)	19(1)
O2	6893(1)	668(1)	687(1)	17(1)
O3	3207(1)	1454(1)	425(1)	16(1)
N1	4312(2)	1931(1)	1231(1)	13(1)
N2	5703(2)	2188(1)	2172(1)	15(1)
N3	1194(2)	3627(1)	1669(1)	14(1)
N4	2521(2)	4727(2)	2542(1)	20(1)
C1	4357(2)	-177(2)	2036(1)	18(1)
C2	4585(2)	351(2)	1327(1)	14(1)
C3	6395(2)	-440(2)	1015(1)	17(1)
C4	5994(2)	1897(2)	1029(1)	14(1)
C5	3315(2)	333(2)	932(1)	17(1)
C6	3157(2)	2605(2)	733(1)	14(1)
C7	6741(2)	1784(2)	1638(1)	14(1)
C8	8472(2)	1239(2)	1620(1)	18(1)
C9	9120(2)	1082(2)	2177(1)	20(1)
C10	8677(2)	1220(2)	3355(1)	26(1)
C11	7614(3)	1544(3)	3909(1)	34(1)
C12	5889(3)	2100(3)	3890(1)	34(1)
C13	5243(2)	2327(2)	3323(1)	26(1)
C14	6326(2)	2001(2)	2745(1)	18(1)
C15	8067(2)	1432(2)	2760(1)	20(1)
C16	1397(2)	3418(2)	1052(1)	14(1)
C17	58(2)	3879(2)	692(1)	17(1)
C18	-1512(2)	4617(2)	981(1)	19(1)
C19	-3390(2)	5466(2)	1980(1)	24(1)
C20	-3597(2)	5529(2)	2630(1)	27(1)
C21	-2216(2)	4908(2)	2973(1)	23(1)
C22	-634(2)	4269(2)	2662(1)	19(1)
C23	-388(2)	4245(2)	1988(1)	15(1)

C24	-1779(2)	4808(2)	1643(1)	18(1)
C25	1765(2)	5759(2)	2788(1)	23(1)
C26	713(3)	7077(2)	3098(1)	38(1)
S1	3429(1)	6197(1)	695(1)	14(1)
F1	863(1)	8689(1)	663(1)	30(1)
F2	1734(2)	7964(1)	1592(1)	30(1)
F3	356(1)	6977(1)	1253(1)	38(1)
O4	3864(1)	4995(1)	1183(1)	18(1)
O5	2945(2)	5933(1)	132(1)	22(1)
O6	4515(1)	6940(1)	582(1)	19(1)
C27	1490(2)	7525(2)	1067(1)	22(1)
S2	3259(1)	-1012(1)	3896(1)	26(1)
F4	5997(2)	-3023(2)	4271(1)	73(1)
F5	5510(2)	-878(2)	4485(1)	54(1)
F6	6363(2)	-1487(2)	3516(1)	62(1)
O7	2840(2)	482(1)	3648(1)	26(1)
O8	3367(2)	-1921(2)	3420(1)	37(1)
O9	2392(2)	-1196(2)	4513(1)	45(1)
C28	5393(3)	-1632(3)	4050(1)	44(1)
N5	650(4)	4372(3)	4098(1)	70(1)
C29	639(3)	3492(3)	4492(1)	39(1)
C30	615(3)	2375(3)	4990(1)	43(1)
C32	6310(40)	4860(30)	5588(14)	67(6)
C31	5190(50)	4900(50)	5150(20)	65(6)
N6	4290(30)	5080(30)	4776(16)	57(6)

Table S-3. Bond lengths [Å] and angles [°] for jonap26.

Cu(1)-O(1)	2.3724(12)	C(8)-C(9)	1.366(2)
Cu(1)-N(1)	2.0151(13)	C(9)-H(9)	0.9500
Cu(1)-N(2)	1.9999(14)	C(9)-C(15)	1.411(2)
Cu(1)-N(3)	1.9898(13)	C(10)-H(10)	0.9500
Cu(1)-N(4)	1.9857(14)	C(10)-C(11)	1.363(3)
O(1)-H(1)	0.82(3)	C(10)-C(15)	1.414(2)
O(1)-C(1)	1.4252(19)	C(11)-H(11)	0.9500
O(2)-C(3)	1.4336(19)	C(11)-C(12)	1.411(3)
O(2)-C(4)	1.4058(18)	C(12)-H(12)	0.9500
O(3)-C(5)	1.4346(19)	C(12)-C(13)	1.373(3)
O(3)-C(6)	1.4141(18)	C(13)-H(13)	0.9500
N(1)-C(2)	1.5192(19)	C(13)-C(14)	1.412(2)
N(1)-C(4)	1.4815(19)	C(14)-C(15)	1.421(2)
N(1)-C(6)	1.493(2)	C(16)-C(17)	1.409(2)
N(2)-C(7)	1.330(2)	C(17)-H(17)	0.9500
N(2)-C(14)	1.380(2)	C(17)-C(18)	1.367(2)
N(3)-C(16)	1.326(2)	C(18)-H(18)	0.9500
N(3)-C(23)	1.381(2)	C(18)-C(24)	1.412(2)
N(4)-C(25)	1.134(2)	C(19)-H(19)	0.9500
C(1)-H(1A)	0.9900	C(19)-C(20)	1.367(3)
C(1)-H(1B)	0.9900	C(19)-C(24)	1.418(2)
C(1)-C(2)	1.530(2)	C(20)-H(20)	0.9500
C(2)-C(3)	1.550(2)	C(20)-C(21)	1.407(3)
C(2)-C(5)	1.535(2)	C(21)-H(21)	0.9500
C(3)-H(3A)	0.9900	C(21)-C(22)	1.376(2)
C(3)-H(3B)	0.9900	C(22)-H(22)	0.9500
C(4)-H(4)	1.0000	C(22)-C(23)	1.412(2)
C(4)-C(7)	1.526(2)	C(23)-C(24)	1.418(2)
C(5)-H(5A)	0.9900	C(25)-C(26)	1.458(2)
C(5)-H(5B)	0.9900	C(26)-H(26A)	0.9800
C(6)-H(6)	1.0000	C(26)-H(26B)	0.9800
C(6)-C(16)	1.527(2)	C(26)-H(26C)	0.9800
C(7)-C(8)	1.405(2)	S(1)-O(4)	1.4475(12)
C(8)-H(8)	0.9500	S(1)-O(5)	1.4386(12)

S(1)-O(6)	1.4404(12)	C(6)-O(3)-C(5)	104.43(11)
S(1)-C(27)	1.8275(17)	C(2)-N(1)-Cu(1)	116.69(9)
F(1)-C(27)	1.330(2)	C(4)-N(1)-Cu(1)	107.46(9)
F(2)-C(27)	1.347(2)	C(4)-N(1)-C(2)	104.38(11)
F(3)-C(27)	1.335(2)	C(4)-N(1)-C(6)	114.54(12)
S(2)-O(7)	1.4507(14)	C(6)-N(1)-Cu(1)	108.33(9)
S(2)-O(8)	1.4398(15)	C(6)-N(1)-C(2)	105.69(11)
S(2)-O(9)	1.4318(15)	C(7)-N(2)-Cu(1)	112.05(11)
S(2)-C(28)	1.818(2)	C(7)-N(2)-C(14)	119.61(14)
F(4)-C(28)	1.335(3)	C(14)-N(2)-Cu(1)	128.32(11)
F(5)-C(28)	1.333(3)	C(16)-N(3)-Cu(1)	112.64(11)
F(6)-C(28)	1.333(3)	C(16)-N(3)-C(23)	119.52(14)
N(5)-C(29)	1.136(3)	C(23)-N(3)-Cu(1)	127.75(11)
C(29)-C(30)	1.441(3)	C(25)-N(4)-Cu(1)	165.60(15)
C(30)-H(30A)	0.9800	O(1)-C(1)-H(1A)	109.9
C(30)-H(30B)	0.9800	O(1)-C(1)-H(1B)	109.9
C(30)-H(30C)	0.9800	O(1)-C(1)-C(2)	109.14(13)
C(32)-H(32A)	0.9800	H(1A)-C(1)-H(1B)	108.3
C(32)-H(32B)	0.9800	C(2)-C(1)-H(1A)	109.9
C(32)-H(32C)	0.9800	C(2)-C(1)-H(1B)	109.9
C(32)-C(31)	1.461(17)	N(1)-C(2)-C(1)	112.58(12)
C(31)-N(6)	1.175(17)	N(1)-C(2)-C(3)	103.45(12)
N(1)-Cu(1)-O(1)	77.42(5)	N(1)-C(2)-C(5)	101.95(12)
N(2)-Cu(1)-O(1)	93.35(5)	C(1)-C(2)-C(3)	111.44(13)
N(2)-Cu(1)-N(1)	81.93(5)	C(1)-C(2)-C(5)	114.21(13)
N(3)-Cu(1)-O(1)	81.98(5)	C(5)-C(2)-C(3)	112.35(13)
N(3)-Cu(1)-N(1)	83.94(5)	O(2)-C(3)-C(2)	105.99(12)
N(3)-Cu(1)-N(2)	165.78(5)	O(2)-C(3)-H(3A)	110.5
N(4)-Cu(1)-O(1)	123.30(5)	O(2)-C(3)-H(3B)	110.5
N(4)-Cu(1)-N(1)	159.08(6)	C(2)-C(3)-H(3A)	110.5
N(4)-Cu(1)-N(2)	98.32(6)	C(2)-C(3)-H(3B)	110.5
N(4)-Cu(1)-N(3)	95.45(6)	H(3A)-C(3)-H(3B)	108.7
Cu(1)-O(1)-H(1)	109.2(18)	O(2)-C(4)-N(1)	106.40(12)
C(1)-O(1)-Cu(1)	104.10(9)	O(2)-C(4)-H(4)	110.6
C(1)-O(1)-H(1)	110.9(18)	O(2)-C(4)-C(7)	111.37(13)
C(4)-O(2)-C(3)	106.28(11)	N(1)-C(4)-H(4)	110.6

N(1)-C(4)-C(7)	107.24(12)	N(2)-C(14)-C(15)	120.23(15)
C(7)-C(4)-H(4)	110.6	C(13)-C(14)-C(15)	119.49(15)
O(3)-C(5)-C(2)	103.88(12)	C(9)-C(15)-C(10)	122.56(16)
O(3)-C(5)-H(5A)	111.0	C(9)-C(15)-C(14)	118.22(15)
O(3)-C(5)-H(5B)	111.0	C(10)-C(15)-C(14)	119.21(16)
C(2)-C(5)-H(5A)	111.0	N(3)-C(16)-C(6)	117.95(14)
C(2)-C(5)-H(5B)	111.0	N(3)-C(16)-C(17)	122.68(15)
H(5A)-C(5)-H(5B)	109.0	C(17)-C(16)-C(6)	119.34(14)
O(3)-C(6)-N(1)	106.19(12)	C(16)-C(17)-H(17)	120.5
O(3)-C(6)-H(6)	110.5	C(18)-C(17)-C(16)	118.93(15)
O(3)-C(6)-C(16)	109.22(12)	C(18)-C(17)-H(17)	120.5
N(1)-C(6)-H(6)	110.5	C(17)-C(18)-H(18)	120.1
N(1)-C(6)-C(16)	109.89(12)	C(17)-C(18)-C(24)	119.79(15)
C(16)-C(6)-H(6)	110.5	C(24)-C(18)-H(18)	120.1
N(2)-C(7)-C(4)	117.57(14)	C(20)-C(19)-H(19)	119.8
N(2)-C(7)-C(8)	122.77(15)	C(20)-C(19)-C(24)	120.36(17)
C(8)-C(7)-C(4)	119.65(14)	C(24)-C(19)-H(19)	119.8
C(7)-C(8)-H(8)	120.6	C(19)-C(20)-H(20)	119.9
C(9)-C(8)-C(7)	118.77(15)	C(19)-C(20)-C(21)	120.21(16)
C(9)-C(8)-H(8)	120.6	C(21)-C(20)-H(20)	119.9
C(8)-C(9)-H(9)	119.9	C(20)-C(21)-H(21)	119.5
C(8)-C(9)-C(15)	120.27(15)	C(22)-C(21)-C(20)	121.07(17)
C(15)-C(9)-H(9)	119.9	C(22)-C(21)-H(21)	119.5
C(11)-C(10)-H(10)	119.7	C(21)-C(22)-H(22)	120.2
C(11)-C(10)-C(15)	120.58(17)	C(21)-C(22)-C(23)	119.57(16)
C(15)-C(10)-H(10)	119.7	C(23)-C(22)-H(22)	120.2
C(10)-C(11)-H(11)	120.1	N(3)-C(23)-C(22)	120.22(15)
C(10)-C(11)-C(12)	119.90(17)	N(3)-C(23)-C(24)	120.16(14)
C(12)-C(11)-H(11)	120.1	C(22)-C(23)-C(24)	119.57(15)
C(11)-C(12)-H(12)	119.3	C(18)-C(24)-C(19)	122.37(16)
C(13)-C(12)-C(11)	121.45(18)	C(18)-C(24)-C(23)	118.45(15)
C(13)-C(12)-H(12)	119.3	C(23)-C(24)-C(19)	119.08(16)
C(12)-C(13)-H(13)	120.3	N(4)-C(25)-C(26)	176.9(2)
C(12)-C(13)-C(14)	119.38(17)	C(25)-C(26)-H(26A)	109.5
C(14)-C(13)-H(13)	120.3	C(25)-C(26)-H(26B)	109.5
N(2)-C(14)-C(13)	120.24(15)	C(25)-C(26)-H(26C)	109.5

H(26A)-C(26)-H(26B)	109.5	F(4)-C(28)-S(2)	111.1(2)
H(26A)-C(26)-H(26C)	109.5	F(5)-C(28)-S(2)	111.16(15)
H(26B)-C(26)-H(26C)	109.5	F(5)-C(28)-F(4)	107.84(18)
O(4)-S(1)-C(27)	103.26(7)	F(6)-C(28)-S(2)	111.08(15)
O(5)-S(1)-O(4)	114.79(7)	F(6)-C(28)-F(4)	107.8(2)
O(5)-S(1)-O(6)	115.94(7)	F(6)-C(28)-F(5)	107.7(2)
O(5)-S(1)-C(27)	102.69(8)	N(5)-C(29)-C(30)	179.6(3)
O(6)-S(1)-O(4)	114.52(7)	C(29)-C(30)-H(30A)	109.5
O(6)-S(1)-C(27)	103.09(7)	C(29)-C(30)-H(30B)	109.5
F(1)-C(27)-S(1)	111.17(12)	C(29)-C(30)-H(30C)	109.5
F(1)-C(27)-F(2)	107.49(13)	H(30A)-C(30)-H(30B)	109.5
F(1)-C(27)-F(3)	108.36(15)	H(30A)-C(30)-H(30C)	109.5
F(2)-C(27)-S(1)	110.89(12)	H(30B)-C(30)-H(30C)	109.5
F(3)-C(27)-S(1)	111.60(11)	H(32A)-C(32)-H(32B)	109.5
F(3)-C(27)-F(2)	107.14(14)	H(32A)-C(32)-H(32C)	109.5
O(7)-S(2)-C(28)	103.09(11)	H(32B)-C(32)-H(32C)	109.5
O(8)-S(2)-O(7)	113.22(9)	C(31)-C(32)-H(32A)	109.5
O(8)-S(2)-C(28)	103.60(10)	C(31)-C(32)-H(32B)	109.5
O(9)-S(2)-O(7)	114.41(9)	C(31)-C(32)-H(32C)	109.5
O(9)-S(2)-O(8)	116.51(10)	N(6)-C(31)-C(32)	172(3)
O(9)-S(2)-C(28)	103.81(11)		

Table S-4. Anisotropic displacement parameters ($Å^2$x 10^3) for jonap26. The anisotropic displacement factor exponent takes the form: $-2\pi^2[h^2 a^{*2}U_{11} + ... + 2 h k a^* b^* U_{12}]$

	U_{11}	U_{22}	U_{33}	U_{23}	U_{13}	U_{12}
Cu1	12(1)	16(1)	13(1)	-5(1)	-1(1)	-5(1)
O1	20(1)	20(1)	13(1)	-1(1)	1(1)	-6(1)
O2	18(1)	15(1)	15(1)	-5(1)	3(1)	-6(1)
O3	23(1)	14(1)	13(1)	-4(1)	-3(1)	-8(1)
N1	15(1)	12(1)	12(1)	-2(1)	-1(1)	-6(1)
N2	15(1)	16(1)	15(1)	-2(1)	-1(1)	-6(1)
N3	15(1)	12(1)	15(1)	-2(1)	-2(1)	-6(1)
N4	19(1)	25(1)	18(1)	-8(1)	0(1)	-11(1)
C1	21(1)	14(1)	16(1)	-1(1)	-1(1)	-5(1)
C2	18(1)	11(1)	15(1)	-2(1)	-2(1)	-6(1)
C3	19(1)	13(1)	17(1)	-3(1)	0(1)	-5(1)
C4	13(1)	14(1)	15(1)	-4(1)	1(1)	-5(1)
C5	22(1)	14(1)	17(1)	-1(1)	-4(1)	-9(1)
C6	17(1)	14(1)	12(1)	-2(1)	-3(1)	-6(1)
C7	17(1)	12(1)	16(1)	-2(1)	-1(1)	-6(1)
C8	16(1)	16(1)	21(1)	-4(1)	0(1)	-7(1)
C9	15(1)	17(1)	27(1)	-2(1)	-3(1)	-6(1)
C10	24(1)	34(1)	25(1)	3(1)	-11(1)	-15(1)
C11	36(1)	52(1)	21(1)	4(1)	-13(1)	-24(1)
C12	32(1)	60(1)	15(1)	-4(1)	-1(1)	-25(1)
C13	23(1)	40(1)	18(1)	-4(1)	-1(1)	-16(1)
C14	18(1)	21(1)	17(1)	-2(1)	-4(1)	-10(1)
C15	21(1)	19(1)	21(1)	0(1)	-5(1)	-10(1)
C16	17(1)	11(1)	15(1)	-2(1)	-3(1)	-7(1)
C17	22(1)	15(1)	17(1)	-1(1)	-6(1)	-7(1)
C18	19(1)	16(1)	25(1)	-2(1)	-9(1)	-6(1)
C19	15(1)	23(1)	35(1)	-10(1)	-4(1)	-5(1)
C20	16(1)	30(1)	36(1)	-16(1)	4(1)	-9(1)
C21	22(1)	28(1)	24(1)	-12(1)	5(1)	-12(1)
C22	18(1)	21(1)	21(1)	-6(1)	-1(1)	-10(1)
C23	14(1)	13(1)	21(1)	-5(1)	-1(1)	-6(1)

C24	16(1)	15(1)	25(1)	-4(1)	-5(1)	-6(1)
C25	25(1)	28(1)	22(1)	-9(1)	3(1)	-17(1)
C26	47(1)	30(1)	39(1)	-22(1)	16(1)	-19(1)
S1	14(1)	11(1)	17(1)	-3(1)	0(1)	-5(1)
F1	24(1)	16(1)	39(1)	-2(1)	-4(1)	1(1)
F2	38(1)	22(1)	26(1)	-11(1)	6(1)	-9(1)
F3	19(1)	26(1)	64(1)	-8(1)	10(1)	-10(1)
O4	20(1)	14(1)	19(1)	-1(1)	-1(1)	-7(1)
O5	27(1)	20(1)	21(1)	-4(1)	-6(1)	-8(1)
O6	17(1)	16(1)	25(1)	-3(1)	1(1)	-8(1)
C27	19(1)	16(1)	28(1)	-5(1)	2(1)	-7(1)
S2	30(1)	27(1)	16(1)	-3(1)	0(1)	-7(1)
F4	76(1)	47(1)	65(1)	-14(1)	-40(1)	21(1)
F5	50(1)	63(1)	42(1)	-20(1)	-24(1)	-4(1)
F6	24(1)	102(1)	51(1)	-32(1)	1(1)	-9(1)
O7	28(1)	29(1)	19(1)	0(1)	-3(1)	-9(1)
O8	42(1)	37(1)	32(1)	-12(1)	-4(1)	-13(1)
O9	59(1)	41(1)	24(1)	1(1)	10(1)	-19(1)
C28	37(1)	47(1)	32(1)	-15(1)	-14(1)	4(1)
N5	119(2)	58(2)	34(1)	-1(1)	0(1)	-40(2)
C29	48(1)	44(1)	26(1)	-10(1)	-2(1)	-18(1)
C30	50(1)	43(1)	32(1)	-6(1)	2(1)	-18(1)
C32	67(16)	43(7)	89(14)	18(9)	-23(10)	-23(10)
C31	65(17)	43(7)	87(13)	18(8)	-24(10)	-25(10)
N6	60(17)	38(7)	85(13)	15(8)	-27(10)	-32(10)

Table S-5. Hydrogen coordinates (x 10^4) and isotropic displacement parameters (Å^2 x 10^3) for jonap26.

	x	y	z	U(eq)
H1	2960(30)	760(30)	2748(13)	37(7)
H1A	4186	-1081	2075	21
H1B	5362	-382	2234	21
H3A	7123	-1005	1348	20
H3B	6454	-1105	709	20
H4	5936	2791	750	17
H5A	3702	-614	757	20
H5B	2228	542	1196	20
H6	3523	3272	416	17
H8	9177	985	1228	21
H9	10286	735	2171	24
H10	9838	848	3368	31
H11	8034	1395	4306	40
H12	5158	2323	4279	40
H13	4078	2700	3320	31
H17	244	3681	255	21
H18	-2421	5000	737	23
H19	-4329	5864	1753	29
H20	-4676	5994	2851	32
H21	-2379	4929	3427	28
H22	286	3847	2900	23
H26A	263	7872	2771	58
H26B	1369	7313	3350	58
H26C	-201	6933	3383	58
H30A	1339	1428	4836	64
H30B	-520	2439	5102	64
H30C	1009	2500	5370	64
H32A	7036	5321	5365	100
H32B	6985	3851	5726	100
H32C	5659	5372	5964	100

Table S-6. Torsion angles [°] for jonap26.

Cu1-O1-C1-C2	-46.97(13)	N3-C23-C24-C19	-178.66(15)
Cu1-N1-C2-C1	-10.57(16)	C1-C2-C3-O2	-130.61(13)
Cu1-N1-C2-C3	-131.00(10)	C1-C2-C5-O3	152.44(12)
Cu1-N1-C2-C5	112.24(11)	C2-N1-C4-O2	31.35(15)
Cu1-N1-C4-O2	155.87(9)	C2-N1-C4-C7	-87.93(13)
Cu1-N1-C4-C7	36.59(13)	C2-N1-C6-O3	-17.42(15)
Cu1-N1-C6-O3	-143.19(9)	C2-N1-C6-C16	100.59(13)
Cu1-N1-C6-C16	-25.18(13)	C3-O2-C4-N1	-38.48(15)
Cu1-N2-C7-C4	-6.38(17)	C3-O2-C4-C7	78.06(15)
Cu1-N2-C7-C8	174.95(12)	C3-C2-C5-O3	-79.39(15)
Cu1-N2-C14-C13	6.3(2)	C4-O2-C3-C2	29.39(15)
Cu1-N2-C14-C15	-175.89(11)	C4-N1-C2-C1	107.83(14)
Cu1-N3-C16-C6	8.88(17)	C4-N1-C2-C3	-12.60(14)
Cu1-N3-C16-C17	-172.90(12)	C4-N1-C2-C5	-129.36(12)
Cu1-N3-C23-C22	-13.8(2)	C4-N1-C6-O3	96.89(14)
Cu1-N3-C23-C24	168.74(11)	C4-N1-C6-C16	-145.10(12)
O1-C1-C2-N1	41.40(17)	C4-C7-C8-C9	-176.92(14)
O1-C1-C2-C3	157.12(13)	C5-O3-C6-N1	37.96(15)
O1-C1-C2-C5	-74.25(16)	C5-O3-C6-C16	-80.49(14)
O2-C4-C7-N2	-136.73(14)	C5-C2-C3-O2	99.77(14)
O2-C4-C7-C8	41.98(19)	C6-O3-C5-C2	-43.10(15)
O3-C6-C16-N3	127.57(14)	C6-N1-C2-C1	-131.03(13)
O3-C6-C16-C17	-50.71(18)	C6-N1-C2-C3	108.55(13)
N1-C2-C3-O2	-9.41(15)	C6-N1-C2-C5	-8.21(14)
N1-C2-C5-O3	30.74(14)	C6-N1-C4-O2	-83.73(15)
N1-C4-C7-N2	-20.70(18)	C6-N1-C4-C7	156.99(12)
N1-C4-C7-C8	158.01(13)	C6-C16-C17-C18	-179.45(14)
N1-C6-C16-N3	11.46(18)	C7-N2-C14-C13	-175.42(16)
N1-C6-C16-C17	-166.82(13)	C7-N2-C14-C15	2.4(2)
N2-C7-C8-C9	1.7(2)	C7-C8-C9-C15	1.4(2)
N2-C14-C15-C9	0.5(2)	C8-C9-C15-C10	176.69(16)
N2-C14-C15-C10	-178.61(15)	C8-C9-C15-C14	-2.4(2)
N3-C16-C17-C18	2.4(2)	C10-C11-C12-C13	0.0(3)
N3-C23-C24-C18	5.0(2)	C11-C10-C15-C9	-178.55(18)

C11-C10-C15-C14	0.6(3)	C23-N3-C16-C6	-174.38(13)
C11-C12-C13-C14	-0.2(3)	C23-N3-C16-C17	3.8(2)
C12-C13-C14-N2	178.42(18)	C24-C19-C20-C21	-1.7(3)
C12-C13-C14-C15	0.5(3)	O4-S1-C27-F1	-176.73(12)
C13-C14-C15-C9	178.42(16)	O4-S1-C27-F2	63.74(13)
C13-C14-C15-C10	-0.7(2)	O4-S1-C27-F3	-55.62(15)
C14-N2-C7-C4	175.03(13)	O5-S1-C27-F1	-57.08(13)
C14-N2-C7-C8	-3.6(2)	O5-S1-C27-F2	-176.61(11)
C15-C10-C11-C12	-0.2(3)	O5-S1-C27-F3	64.03(15)
C16-N3-C23-C22	170.03(14)	O6-S1-C27-F1	63.76(13)
C16-N3-C23-C24	-7.5(2)	O6-S1-C27-F2	-55.77(13)
C16-C17-C18-C24	-4.8(2)	O6-S1-C27-F3	-175.13(13)
C17-C18-C24-C19	-175.06(16)	O7-S2-C28-F4	-178.60(15)
C17-C18-C24-C23	1.2(2)	O7-S2-C28-F5	61.3(2)
C19-C20-C21-C22	2.2(3)	O7-S2-C28-F6	-58.57(19)
C20-C19-C24-C18	174.97(17)	O8-S2-C28-F4	-60.39(18)
C20-C19-C24-C23	-1.3(3)	O8-S2-C28-F5	179.53(18)
C20-C21-C22-C23	0.5(3)	O8-S2-C28-F6	59.6(2)
C21-C22-C23-N3	179.07(15)	O9-S2-C28-F4	61.78(17)
C21-C22-C23-C24	-3.4(2)	O9-S2-C28-F5	-58.3(2)
C22-C23-C24-C18	-172.55(15)	O9-S2-C28-F6	-178.19(17)
C22-C23-C24-C19	3.8(2)		

Table S-7. Hydrogen bonds and close contacts for jonap26 [Å and °].

D-H...A	d(D-H)	d(H...A)	d(D...A)	<(DHA)
O1-H1...O7	0.82(3)	1.88(3)	2.6978(17)	177(3)

REFERENCE NUMBER: jonap49

2f

CRYSTAL STRUCTURE REPORT

$C_{26}\ H_{23}\ Cu\ F_6\ N_3\ O_{10}\ S_2$

or

$[(\kappa^4\text{-}L^q)Cu(OH_2)][OTf]_2$

Report prepared for:

A. Panda, Prof. W. Jones

October 07, 2025

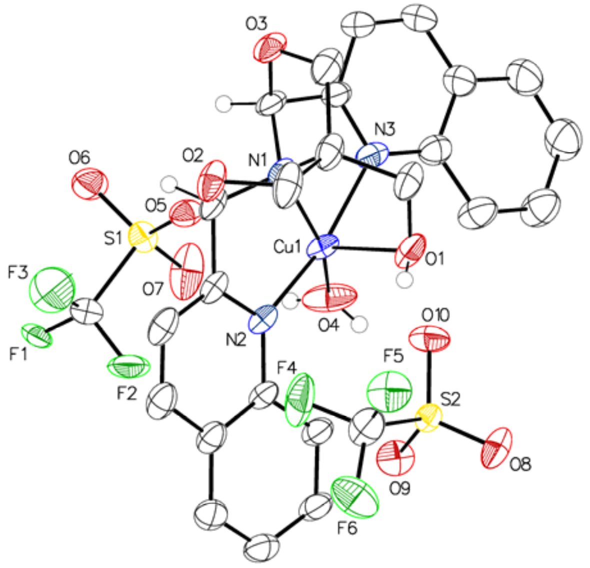

William W. Brennessel
X-ray Crystallographic Facility
Department of Chemistry, University of Rochester
120 Trustee Road
Rochester, NY 14627

Data collection

A crystal (0.12 x 0.079 x 0.035 mm^3) was placed onto a nylon loop and mounted on a Rigaku XtaLAB Synergy-S Dualflex diffractometer equipped with a HyPix-6000HE HPC area detector for data collection at 100.00(10) K. A preliminary set of cell constants and an orientation matrix were calculated from a small sampling of reflections.[1] A short pre-experiment was run, from which an optimal data collection strategy was determined. The full data collection was carried out using a PhotonJet (Cu) X-ray source with frame times of 1.11 and 4.44 seconds and a detector distance of 34.0 mm. Series of frames were collected in 0.50º steps in ω at different 2θ, κ, and ϕ settings. After the intensity data were corrected for absorption, the final cell constants were calculated from the xyz centroids of 27255 strong reflections from the actual data collection after integration.[1] See Table S-1 for additional crystal and refinement information.

Structure solution and refinement

The structure was solved using SHELXT[2] and refined using SHELXL.[3] The space group *I*2/*a* was determined based on systematic absences and intensity statistics. Most or all non-hydrogen atoms were assigned from the solution. Refinement proceeded in an iterative fashion, with each stage including full-matrix least squares cycles, followed by a difference Fourier synthesis, which located any remaining electron density. All non-hydrogen atoms were refined with anisotropic displacement parameters. The O-H hydrogen atoms were found from the difference Fourier map and refined freely. All other hydrogen atoms were placed in ideal positions and refined as riding atoms with relative isotropic displacement parameters. The final full matrix least squares refinement converged to $R1 = 0.0343$ (F^2, $I > 2\sigma(I)$) and $wR2 = 0.0887$ (F^2, all data).

Structure description

The structure is the one suggested. The asymmetric unit contains dicationic copper complex and two triflate anions in general positions. The triflate anion containing S1 is modeled as disordered over two positions (0.51:0.49); the triflate anion containing S2 is modeled as disordered over three positions (0.36:0.39:0.25). The Cu1…O5 and Cu1…O5a distances are 2.700(8) and 2.474(8), respectively. The cation and anions are linked via O-H…O hydrogen bonding (see figures and Table S-7).

Structure manipulation and figure generation were performed using Olex2.[4] Unless noted otherwise all structural diagrams containing anisotropic displacement ellipsoids are drawn at the 50 % probability level.

Data collection, structure solution, and structure refinement were conducted at the X-ray Crystallographic Facility, B04 Hutchison Hall, Department of Chemistry, University of Rochester. The instrument was purchased with funding from NSF MRI program grant CHE-1725028. All publications arising from this report MUST either 1) include William W. Brennessel as a coauthor or 2) acknowledge William W. Brennessel and the X-ray Crystallographic Facility of the Department of Chemistry at the University of Rochester.

[1] *CrysAlisPro*, version 171.44.114a; Rigaku Corporation: Oxford, UK, 2025.

[2] Sheldrick, G. M. *SHELXT*, version 2018/2; *Acta. Crystallogr.* **2015**, *A71*, 3-8.

[3] Sheldrick, G. M. *SHELXL*, version 2025/1; *Acta. Crystallogr.* **2015**, *C71*, 3-8.

[4] Dolomanov, O. V.; Bourhis, L. J.; Gildea, R. J.; Howard, J. A. K.; Puschmann, H. *Olex2*, version 1.5; *J. Appl. Cryst.* **2009**, *42*, 339-341.

Some equations of interest:

$$R_{int} = \Sigma |F_o^2 - < F_o^2 >| / \Sigma|F_o^2|$$

$$R1 = \Sigma||F_o|-|F_c|| / \Sigma|F_o|$$

$$wR2 = [\Sigma[w(F_o^2-F_c^2)^2] / \Sigma [w(F_o^2)^2]]^{1/2}$$

where $w = 1 / [\sigma^2 (F_o^2) + (aP)^2 + bP]$ and

$$P = 1/3 \max (0, F_o^2) + 2/3 F_c^2$$

$$GOF = S = [\Sigma[w(F_o^2-F_c^2)^2] / (m-n)]^{1/2}$$

where m = number of reflections and n = number of parameters

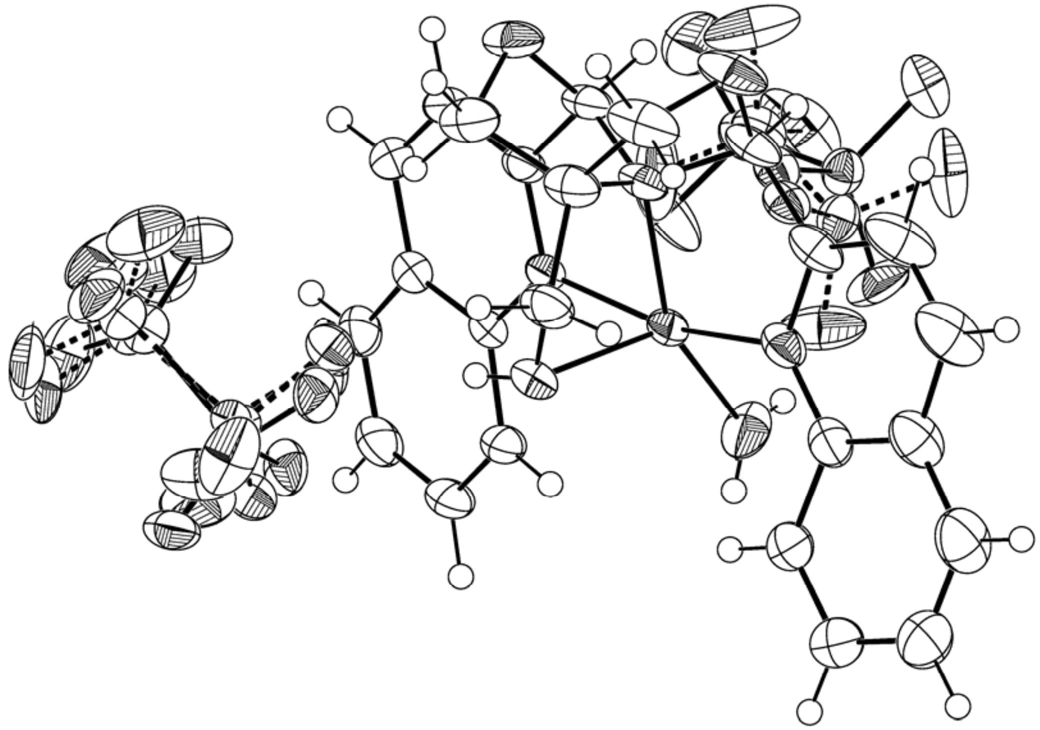

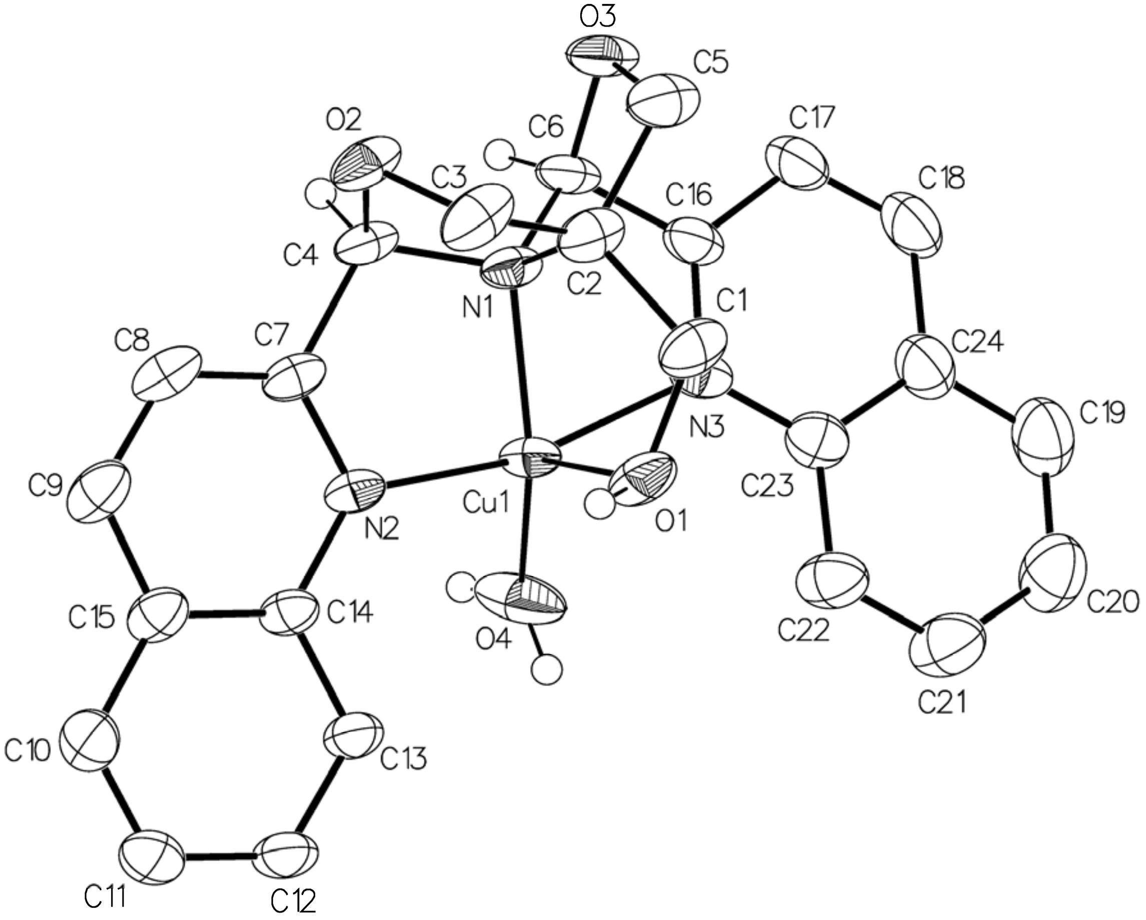
O3
C5
O2
C6
C17
C3
C16
C18
C4
C2
N1
C1
C8
C7
C24
C19
N3
C9
C23
N2
Cu1
O1
C15
C14
C20
C22
O4
C21
C10
C13
C11
C12

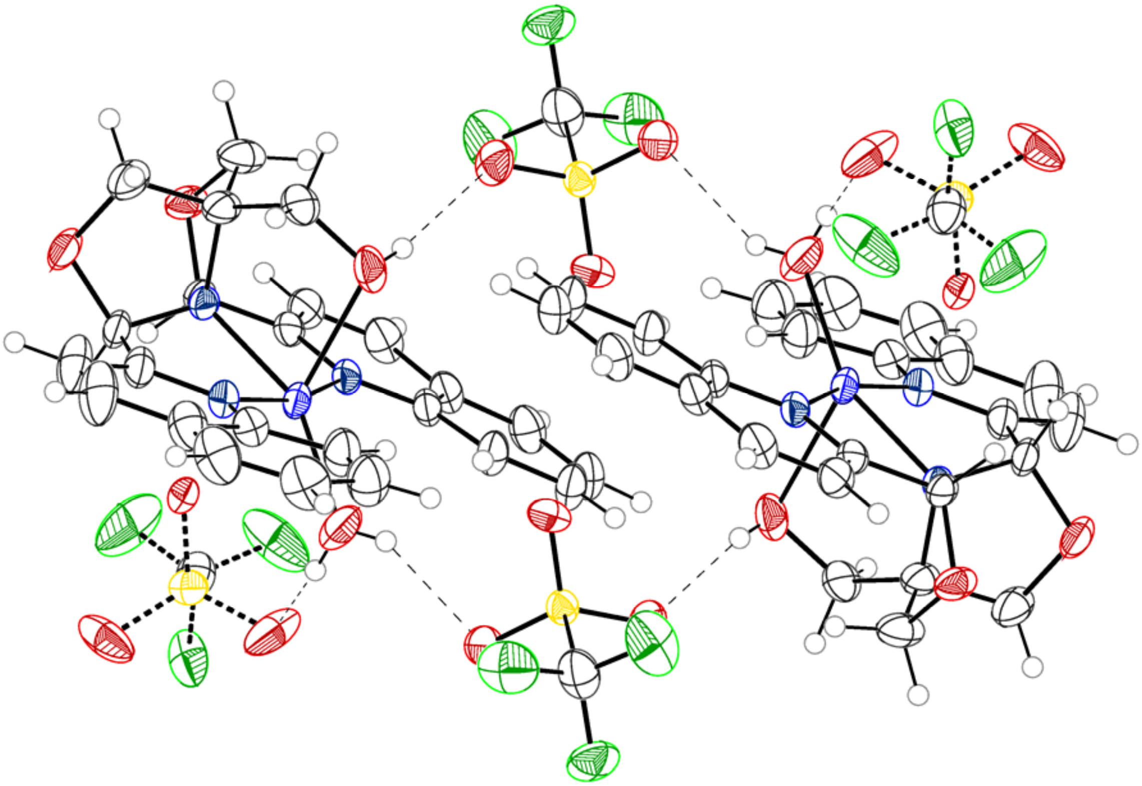

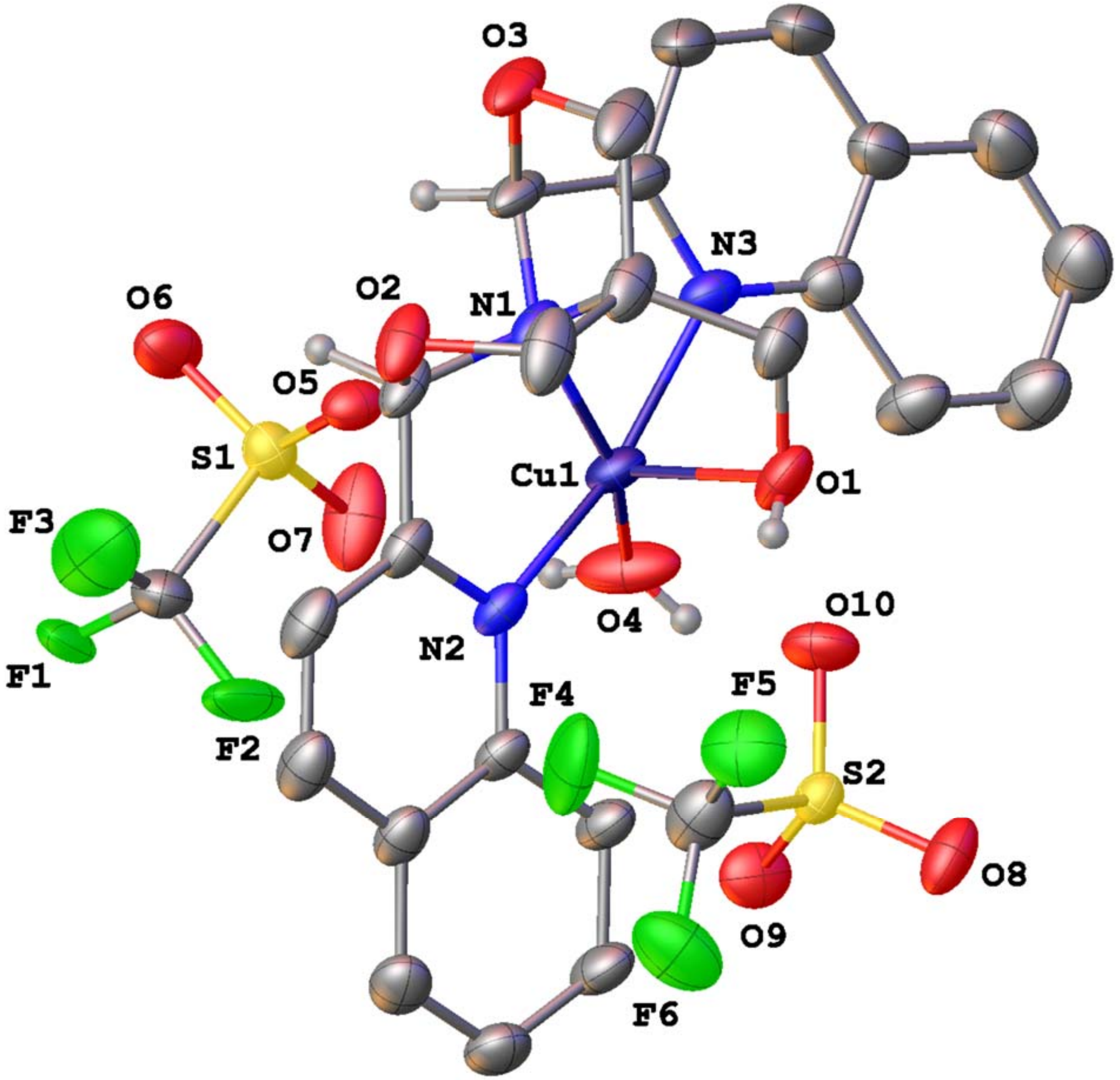
O3
N3
O6
O2
N1
O5
S1
Cu1
O1
F3
O7
O4
O10
N2
F1
F5
F4
F2
S2
O8
O9
F6

Table S-1. Crystal data and structure refinement for jonap49.

Identification code	jonap49	
Empirical formula	C26 H23 Cu F6 N3 O10 S2	
Formula weight	779.13	
Temperature	100.00(10) K	
Wavelength	1.54184 Å	
Crystal system	monoclinic	
Space group	*I*2/*a*	
Unit cell dimensions	*a* = 19.48740(10) Å	α = 90°
	b = 12.17970(10) Å	β = 101.4740(10)°
	c = 25.5882(2) Å	γ = 90°
Volume	5952.00(8) $Å^3$	
Z	8	
Density (calculated)	1.739 Mg/m^3	
Absorption coefficient	3.267 mm^{-1}	
F(000)	3160	
Crystal color, morphology	blue, block	
Crystal size	0.12 x 0.079 x 0.035 mm^3	
Theta range for data collection	3.525 to 80.213°	
Index ranges	$-24 \le h \le 24$, $-15 \le k \le 13$, $-30 \le l \le 32$	
Reflections collected	51833	
Independent reflections	6452 [*R*(int) = 0.0363]	
Observed reflections	5829	
Completeness to theta = 74.504°	100.0%	
Absorption correction	Multi-scan	
Max. and min. transmission	1.00000 and 0.88730	
Refinement method	Full-matrix least-squares on F^2	
Data / restraints / parameters	6452 / 473 / 665	
Goodness-of-fit on F^2	1.052	
Final *R* indices [*I*>2sigma(*I*)]	*R*1 = 0.0343, *wR*2 = 0.0865	
R indices (all data)	*R*1 = 0.0381, *wR*2 = 0.0887	
Largest diff. peak and hole	0.349 and -0.469 e.$Å^{-3}$	

Table S-2. Atomic coordinates (x 10^4) and equivalent isotropic displacement parameters ($Å^2$x 10^3) for jonap49. U_{eq} is defined as one third of the trace of the orthogonalized U_{ij} tensor.

	x	y	z	U_{eq}
Cu1	3712(1)	4997(1)	3570(1)	28(1)
O1	3266(1)	4659(1)	4329(1)	39(1)
O2	2643(1)	2173(1)	3231(1)	41(1)
O3	1756(1)	4246(2)	2767(1)	45(1)
O4	4502(1)	6002(2)	3524(1)	56(1)
N1	2869(1)	4048(1)	3272(1)	30(1)
N2	4218(1)	3558(1)	3686(1)	27(1)
N3	2990(1)	6191(1)	3379(1)	31(1)
C1	2536(1)	4476(2)	4155(1)	41(1)
C2	2370(1)	3790(2)	3640(1)	38(1)
C3	2481(1)	2555(2)	3724(1)	47(1)
C4	3117(1)	2969(2)	3112(1)	31(1)
C5	1643(1)	4072(2)	3299(1)	50(1)
C6	2434(1)	4680(2)	2833(1)	34(1)
C7	3853(1)	2735(2)	3424(1)	30(1)
C8	4110(1)	1664(2)	3417(1)	37(1)
C9	4773(1)	1447(2)	3678(1)	39(1)
C10	5888(1)	2120(2)	4222(1)	39(1)
C11	6261(1)	2948(2)	4501(1)	40(1)
C12	5950(1)	3976(2)	4536(1)	38(1)
C13	5274(1)	4180(2)	4277(1)	33(1)
C14	4886(1)	3348(2)	3967(1)	28(1)
C15	5186(1)	2294(2)	3956(1)	33(1)
C16	2452(1)	5879(2)	3004(1)	35(1)
C17	1904(1)	6589(2)	2789(1)	53(1)
C18	1911(1)	7634(3)	2973(1)	59(1)
C19	2498(2)	9070(2)	3596(1)	55(1)
C20	3029(2)	9362(2)	4000(1)	55(1)
C21	3546(1)	8595(2)	4216(1)	52(1)
C22	3533(1)	7552(2)	4014(1)	44(1)
C23	3003(1)	7237(2)	3583(1)	35(1)

C24	2466(1)	7998(2)	3378(1)	44(1)
S1	4313(1)	4760(2)	2235(1)	30(1)
F1	5378(6)	3641(12)	2001(6)	52(2)
F2	5247(3)	3534(7)	2816(3)	65(2)
F3	4559(3)	2688(3)	2217(2)	82(2)
O5	3808(5)	4484(7)	2561(3)	31(1)
O6	4031(2)	4776(4)	1674(1)	44(1)
O7	4754(3)	5673(3)	2446(2)	58(1)
C25	4914(5)	3606(8)	2315(5)	39(1)
S1A	4458(1)	5030(2)	2400(1)	30(1)
F1A	5290(7)	3858(12)	1988(7)	50(2)
F2A	5101(5)	3215(7)	2730(4)	79(2)
F3A	4322(3)	3051(5)	2001(3)	86(2)
O5A	3871(5)	4782(7)	2640(4)	30(1)
O6A	4272(2)	5489(5)	1876(2)	61(2)
O7A	5026(2)	5535(4)	2759(3)	61(2)
C25A	4801(6)	3702(8)	2278(5)	40(1)
S2	4220(4)	2796(8)	5418(3)	26(1)
F4	4046(4)	1061(4)	4818(2)	68(2)
F5	3552(4)	971(6)	5495(3)	52(2)
F6	4668(4)	780(8)	5592(3)	64(2)
O8	4282(3)	2981(4)	5977(2)	43(1)
O9	4839(4)	3057(7)	5222(2)	40(2)
O10	3576(2)	3193(4)	5095(2)	41(1)
C26	4110(6)	1339(10)	5335(4)	46(2)
S2A	4158(5)	2684(7)	5350(4)	25(1)
F4A	4543(6)	854(6)	4961(3)	91(3)
F5A	3648(7)	681(9)	5336(5)	98(4)
F6A	4676(6)	885(8)	5808(3)	82(3)
O8A	3800(6)	2936(13)	5762(4)	58(3)
O9A	4867(3)	3042(6)	5448(3)	33(1)
O10A	3780(3)	2849(8)	4821(3)	42(2)
C26A	4248(7)	1200(9)	5362(4)	48(2)
S2B	4143(7)	2584(12)	5371(5)	26(1)
F4B	4427(6)	595(8)	5155(4)	63(2)
F5B	3343(6)	908(12)	5125(5)	93(4)

F6B	4058(8)	820(6)	5891(3)	96(4)
O8B	3628(10)	3050(20)	5625(7)	62(6)
O9B	4841(6)	2724(10)	5658(6)	85(5)
O10B	4036(8)	2823(15)	4812(5)	66(4)
C26B	3984(7)	1132(13)	5389(5)	46(2)

Table S-3. Bond lengths [Å] and angles [°] for jonap49.

Cu(1)-O(1)	2.3163(16)	C(8)-H(8)	0.9500
Cu(1)-O(4)	1.9890(17)	C(8)-C(9)	1.356(3)
Cu(1)-N(1)	2.0291(15)	C(9)-H(9)	0.9500
Cu(1)-N(2)	2.0035(16)	C(9)-C(15)	1.411(3)
Cu(1)-N(3)	2.0154(16)	C(10)-H(10)	0.9500
O(1)-H(1)	0.74(3)	C(10)-C(11)	1.360(3)
O(1)-C(1)	1.422(3)	C(10)-C(15)	1.417(3)
O(2)-C(3)	1.438(2)	C(11)-H(11)	0.9500
O(2)-C(4)	1.414(2)	C(11)-C(12)	1.402(3)
O(3)-C(5)	1.437(3)	C(12)-H(12)	0.9500
O(3)-C(6)	1.402(2)	C(12)-C(13)	1.373(3)
O(4)-H(4A)	0.86(6)	C(13)-H(13)	0.9500
O(4)-H(4B)	0.76(4)	C(13)-C(14)	1.410(2)
N(1)-C(2)	1.514(2)	C(14)-C(15)	1.414(3)
N(1)-C(4)	1.486(3)	C(16)-C(17)	1.400(3)
N(1)-C(6)	1.481(2)	C(17)-H(17)	0.9500
N(2)-C(7)	1.332(2)	C(17)-C(18)	1.356(4)
N(2)-C(14)	1.380(2)	C(18)-H(18)	0.9500
N(3)-C(16)	1.327(2)	C(18)-C(24)	1.412(3)
N(3)-C(23)	1.376(3)	C(19)-H(19)	0.9500
C(1)-H(1A)	0.9900	C(19)-C(20)	1.357(4)
C(1)-H(1B)	0.9900	C(19)-C(24)	1.416(4)
C(1)-C(2)	1.540(3)	C(20)-H(20)	0.9500
C(2)-C(3)	1.529(3)	C(20)-C(21)	1.405(4)
C(2)-C(5)	1.549(3)	C(21)-H(21)	0.9500
C(3)-H(3A)	0.9900	C(21)-C(22)	1.369(3)
C(3)-H(3B)	0.9900	C(22)-H(22)	0.9500
C(4)-H(4)	1.0000	C(22)-C(23)	1.407(3)
C(4)-C(7)	1.523(3)	C(23)-C(24)	1.418(3)
C(5)-H(5A)	0.9900	S(1)-O(5)	1.449(7)
C(5)-H(5B)	0.9900	S(1)-O(6)	1.432(3)
C(6)-H(6)	1.0000	S(1)-O(7)	1.443(4)
C(6)-C(16)	1.524(3)	S(1)-C(25)	1.815(9)
C(7)-C(8)	1.398(3)	F(1)-C(25)	1.322(9)

F(2)-C(25)	1.319(9)	N(2)-Cu(1)-N(1)	84.04(6)
F(3)-C(25)	1.313(10)	N(2)-Cu(1)-N(3)	165.16(6)
S(1A)-O(5A)	1.433(8)	N(3)-Cu(1)-O(1)	88.37(6)
S(1A)-O(6A)	1.431(4)	N(3)-Cu(1)-N(1)	81.12(7)
S(1A)-O(7A)	1.429(4)	Cu(1)-O(1)-H(1)	115(2)
S(1A)-C(25A)	1.802(10)	C(1)-O(1)-Cu(1)	106.53(11)
F(1A)-C(25A)	1.333(10)	C(1)-O(1)-H(1)	109(2)
F(2A)-C(25A)	1.328(10)	C(4)-O(2)-C(3)	103.59(14)
F(3A)-C(25A)	1.318(10)	C(6)-O(3)-C(5)	105.02(14)
S(2)-O(8)	1.430(8)	Cu(1)-O(4)-H(4A)	118(4)
S(2)-O(9)	1.430(9)	Cu(1)-O(4)-H(4B)	126(3)
S(2)-O(10)	1.443(9)	H(4A)-O(4)-H(4B)	114(5)
S(2)-C(26)	1.794(11)	C(2)-N(1)-Cu(1)	117.14(11)
F(4)-C(26)	1.349(11)	C(4)-N(1)-Cu(1)	108.84(11)
F(5)-C(26)	1.315(11)	C(4)-N(1)-C(2)	105.83(15)
F(6)-C(26)	1.339(11)	C(6)-N(1)-Cu(1)	106.73(12)
S(2A)-O(8A)	1.407(9)	C(6)-N(1)-C(2)	103.78(15)
S(2A)-O(9A)	1.422(8)	C(6)-N(1)-C(4)	114.76(14)
S(2A)-O(10A)	1.420(9)	C(7)-N(2)-Cu(1)	112.70(12)
S(2A)-C(26A)	1.816(10)	C(7)-N(2)-C(14)	118.49(16)
F(4A)-C(26A)	1.339(11)	C(14)-N(2)-Cu(1)	128.69(12)
F(5A)-C(26A)	1.319(11)	C(16)-N(3)-Cu(1)	112.43(14)
F(6A)-C(26A)	1.328(11)	C(16)-N(3)-C(23)	119.44(17)
S(2B)-O(8B)	1.419(12)	C(23)-N(3)-Cu(1)	128.12(12)
S(2B)-O(9B)	1.423(12)	O(1)-C(1)-H(1A)	109.1
S(2B)-O(10B)	1.434(12)	O(1)-C(1)-H(1B)	109.1
S(2B)-C(26B)	1.798(13)	O(1)-C(1)-C(2)	112.29(17)
F(4B)-C(26B)	1.317(12)	H(1A)-C(1)-H(1B)	107.9
F(5B)-C(26B)	1.326(12)	C(2)-C(1)-H(1A)	109.1
F(6B)-C(26B)	1.317(12)	C(2)-C(1)-H(1B)	109.1
O(4)-Cu(1)-O(1)	125.62(8)	N(1)-C(2)-C(1)	111.65(16)
O(4)-Cu(1)-N(1)	155.06(8)	N(1)-C(2)-C(3)	101.67(16)
O(4)-Cu(1)-N(2)	100.52(7)	N(1)-C(2)-C(5)	103.42(16)
O(4)-Cu(1)-N(3)	93.01(7)	C(1)-C(2)-C(5)	111.99(19)
N(1)-Cu(1)-O(1)	78.68(5)	C(3)-C(2)-C(1)	114.37(18)
N(2)-Cu(1)-O(1)	88.66(6)	C(3)-C(2)-C(5)	112.72(19)

O(2)-C(3)-C(2)	104.05(17)	C(10)-C(11)-H(11)	119.9
O(2)-C(3)-H(3A)	110.9	C(10)-C(11)-C(12)	120.15(19)
O(2)-C(3)-H(3B)	110.9	C(12)-C(11)-H(11)	119.9
C(2)-C(3)-H(3A)	110.9	C(11)-C(12)-H(12)	119.4
C(2)-C(3)-H(3B)	110.9	C(13)-C(12)-C(11)	121.11(19)
H(3A)-C(3)-H(3B)	109.0	C(13)-C(12)-H(12)	119.4
O(2)-C(4)-N(1)	106.47(14)	C(12)-C(13)-H(13)	120.1
O(2)-C(4)-H(4)	110.0	C(12)-C(13)-C(14)	119.77(18)
O(2)-C(4)-C(7)	110.22(16)	C(14)-C(13)-H(13)	120.1
N(1)-C(4)-H(4)	110.0	N(2)-C(14)-C(13)	120.32(17)
N(1)-C(4)-C(7)	110.23(14)	N(2)-C(14)-C(15)	120.59(16)
C(7)-C(4)-H(4)	110.0	C(13)-C(14)-C(15)	119.09(17)
O(3)-C(5)-C(2)	105.73(16)	C(9)-C(15)-C(10)	122.15(19)
O(3)-C(5)-H(5A)	110.6	C(9)-C(15)-C(14)	118.53(18)
O(3)-C(5)-H(5B)	110.6	C(14)-C(15)-C(10)	119.33(17)
C(2)-C(5)-H(5A)	110.6	N(3)-C(16)-C(6)	116.88(17)
C(2)-C(5)-H(5B)	110.6	N(3)-C(16)-C(17)	122.6(2)
H(5A)-C(5)-H(5B)	108.7	C(17)-C(16)-C(6)	120.43(18)
O(3)-C(6)-N(1)	106.22(17)	C(16)-C(17)-H(17)	120.4
O(3)-C(6)-H(6)	110.5	C(18)-C(17)-C(16)	119.2(2)
O(3)-C(6)-C(16)	111.35(16)	C(18)-C(17)-H(17)	120.4
N(1)-C(6)-H(6)	110.5	C(17)-C(18)-H(18)	119.9
N(1)-C(6)-C(16)	107.62(14)	C(17)-C(18)-C(24)	120.3(2)
C(16)-C(6)-H(6)	110.5	C(24)-C(18)-H(18)	119.9
N(2)-C(7)-C(4)	118.42(17)	C(20)-C(19)-H(19)	119.7
N(2)-C(7)-C(8)	123.46(18)	C(20)-C(19)-C(24)	120.5(2)
C(8)-C(7)-C(4)	118.12(16)	C(24)-C(19)-H(19)	119.7
C(7)-C(8)-H(8)	120.5	C(19)-C(20)-H(20)	119.8
C(9)-C(8)-C(7)	119.01(18)	C(19)-C(20)-C(21)	120.3(2)
C(9)-C(8)-H(8)	120.5	C(21)-C(20)-H(20)	119.8
C(8)-C(9)-H(9)	120.1	C(20)-C(21)-H(21)	119.6
C(8)-C(9)-C(15)	119.81(19)	C(22)-C(21)-C(20)	120.7(2)
C(15)-C(9)-H(9)	120.1	C(22)-C(21)-H(21)	119.6
C(11)-C(10)-H(10)	119.8	C(21)-C(22)-H(22)	119.9
C(11)-C(10)-C(15)	120.37(19)	C(21)-C(22)-C(23)	120.2(2)
C(15)-C(10)-H(10)	119.8	C(23)-C(22)-H(22)	119.9

N(3)-C(23)-C(22)	120.34(18)	O(9)-S(2)-O(10)	115.0(7)
N(3)-C(23)-C(24)	120.58(18)	O(9)-S(2)-C(26)	105.5(7)
C(22)-C(23)-C(24)	119.0(2)	O(10)-S(2)-C(26)	101.2(6)
C(18)-C(24)-C(19)	123.1(2)	F(4)-C(26)-S(2)	110.5(8)
C(18)-C(24)-C(23)	117.8(2)	F(5)-C(26)-S(2)	112.8(8)
C(19)-C(24)-C(23)	119.1(2)	F(5)-C(26)-F(4)	107.4(9)
O(5)-S(1)-C(25)	104.7(6)	F(5)-C(26)-F(6)	108.4(10)
O(6)-S(1)-O(5)	114.2(4)	F(6)-C(26)-S(2)	112.2(9)
O(6)-S(1)-O(7)	116.5(3)	F(6)-C(26)-F(4)	105.1(9)
O(6)-S(1)-C(25)	103.5(4)	O(8A)-S(2A)-O(9A)	114.1(8)
O(7)-S(1)-O(5)	112.7(3)	O(8A)-S(2A)-O(10A)	116.2(8)
O(7)-S(1)-C(25)	103.4(4)	O(8A)-S(2A)-C(26A)	105.4(9)
F(1)-C(25)-S(1)	114.3(8)	O(9A)-S(2A)-C(26A)	102.4(7)
F(2)-C(25)-S(1)	110.0(7)	O(10A)-S(2A)-O(9A)	115.2(7)
F(2)-C(25)-F(1)	109.2(10)	O(10A)-S(2A)-C(26A)	100.8(7)
F(3)-C(25)-S(1)	109.5(6)	F(4A)-C(26A)-S(2A)	110.8(8)
F(3)-C(25)-F(1)	108.0(10)	F(5A)-C(26A)-S(2A)	113.2(9)
F(3)-C(25)-F(2)	105.5(8)	F(5A)-C(26A)-F(4A)	108.8(11)
O(5A)-S(1A)-C(25A)	103.9(5)	F(5A)-C(26A)-F(6A)	107.6(10)
O(6A)-S(1A)-O(5A)	114.2(5)	F(6A)-C(26A)-S(2A)	110.1(9)
O(6A)-S(1A)-C(25A)	102.8(5)	F(6A)-C(26A)-F(4A)	106.0(9)
O(7A)-S(1A)-O(5A)	113.4(4)	O(8B)-S(2B)-O(9B)	113.9(13)
O(7A)-S(1A)-O(6A)	116.9(3)	O(8B)-S(2B)-O(10B)	113.6(12)
O(7A)-S(1A)-C(25A)	103.3(4)	O(8B)-S(2B)-C(26B)	104.1(14)
F(1A)-C(25A)-S(1A)	107.4(9)	O(9B)-S(2B)-O(10B)	115.1(12)
F(2A)-C(25A)-S(1A)	111.4(8)	O(9B)-S(2B)-C(26B)	104.8(10)
F(2A)-C(25A)-F(1A)	108.0(10)	O(10B)-S(2B)-C(26B)	103.6(11)
F(3A)-C(25A)-S(1A)	112.7(7)	F(4B)-C(26B)-S(2B)	110.2(11)
F(3A)-C(25A)-F(1A)	107.1(11)	F(4B)-C(26B)-F(5B)	107.6(13)
F(3A)-C(25A)-F(2A)	110.0(9)	F(4B)-C(26B)-F(6B)	110.2(11)
O(8)-S(2)-O(9)	114.0(6)	F(5B)-C(26B)-S(2B)	109.8(11)
O(8)-S(2)-O(10)	114.2(7)	F(6B)-C(26B)-S(2B)	108.9(10)
O(8)-S(2)-C(26)	105.0(7)	F(6B)-C(26B)-F(5B)	110.3(13)

Table S-4. Anisotropic displacement parameters ($Å^2 x 10^3$) for jonap49. The anisotropic displacement factor exponent takes the form: $-2\pi^2[h^2 a^{*2}U_{11} + ... + 2 h k a^* b^* U_{12}]$

	U_{11}	U_{22}	U_{33}	U_{23}	U_{13}	U_{12}
Cu1	25(1)	30(1)	24(1)	1(1)	-5(1)	-6(1)
O1	52(1)	37(1)	22(1)	-4(1)	-4(1)	-4(1)
O2	44(1)	50(1)	33(1)	-17(1)	16(1)	-26(1)
O3	22(1)	83(1)	30(1)	-17(1)	3(1)	-14(1)
O4	33(1)	42(1)	79(1)	21(1)	-18(1)	-14(1)
N1	26(1)	44(1)	19(1)	-6(1)	4(1)	-11(1)
N2	31(1)	28(1)	19(1)	0(1)	-1(1)	-7(1)
N3	26(1)	41(1)	25(1)	3(1)	-1(1)	-2(1)
C1	49(1)	49(1)	24(1)	-7(1)	10(1)	-10(1)
C2	37(1)	52(1)	27(1)	-10(1)	14(1)	-17(1)
C3	58(1)	52(1)	36(1)	-14(1)	25(1)	-26(1)
C4	30(1)	41(1)	22(1)	-9(1)	5(1)	-15(1)
C5	33(1)	83(2)	36(1)	-19(1)	14(1)	-18(1)
C6	19(1)	61(1)	21(1)	-6(1)	-1(1)	-6(1)
C7	36(1)	34(1)	18(1)	-3(1)	3(1)	-11(1)
C8	53(1)	30(1)	27(1)	-5(1)	3(1)	-14(1)
C9	58(1)	26(1)	30(1)	-2(1)	2(1)	-2(1)
C10	48(1)	33(1)	32(1)	4(1)	0(1)	9(1)
C11	38(1)	43(1)	33(1)	3(1)	-6(1)	6(1)
C12	37(1)	37(1)	32(1)	-4(1)	-10(1)	-2(1)
C13	35(1)	28(1)	31(1)	-3(1)	-7(1)	0(1)
C14	33(1)	28(1)	20(1)	2(1)	-3(1)	-3(1)
C15	45(1)	28(1)	23(1)	1(1)	0(1)	-1(1)
C16	23(1)	57(1)	24(1)	-3(1)	1(1)	3(1)
C17	30(1)	83(2)	41(1)	-16(1)	-6(1)	17(1)
C18	42(1)	82(2)	47(1)	-10(1)	-5(1)	31(1)
C19	62(2)	55(2)	48(1)	3(1)	8(1)	23(1)
C20	71(2)	40(1)	53(1)	-1(1)	9(1)	7(1)
C21	61(2)	39(1)	49(1)	-1(1)	-5(1)	-4(1)
C22	48(1)	35(1)	42(1)	4(1)	-9(1)	-2(1)
C23	35(1)	39(1)	30(1)	5(1)	3(1)	0(1)

C24	40(1)	56(1)	35(1)	0(1)	5(1)	14(1)
S1	32(1)	28(1)	30(1)	2(1)	10(1)	1(1)
F1	32(3)	90(5)	33(2)	-6(3)	3(2)	38(3)
F2	34(2)	123(7)	34(2)	11(3)	-3(1)	34(3)
F3	108(4)	43(2)	84(3)	-5(2)	-11(2)	18(2)
O5	23(2)	41(4)	28(3)	3(2)	3(2)	-3(2)
O6	41(2)	58(3)	31(2)	10(2)	8(1)	12(2)
O7	90(3)	39(2)	57(3)	-16(2)	39(3)	-40(2)
C25	28(3)	48(2)	39(2)	-4(2)	0(2)	11(2)
S1A	28(1)	27(1)	34(1)	3(1)	9(1)	2(1)
F1A	37(3)	77(5)	36(2)	1(3)	7(2)	36(3)
F2A	110(6)	66(4)	73(5)	41(3)	44(4)	54(4)
F3A	75(3)	66(3)	116(4)	-50(3)	15(3)	-32(3)
O5A	21(2)	41(4)	28(3)	0(2)	0(2)	-4(2)
O6A	61(3)	73(4)	57(3)	41(3)	31(2)	35(3)
O7A	37(2)	76(3)	74(3)	-42(3)	24(2)	-24(2)
C25A	32(3)	48(2)	39(2)	-3(2)	3(2)	6(2)
S2	30(1)	23(2)	23(1)	-1(1)	2(1)	-2(1)
F4	117(5)	39(3)	47(3)	-20(2)	12(3)	1(3)
F5	50(3)	36(3)	69(5)	4(3)	9(3)	-18(2)
F6	63(4)	47(3)	86(7)	17(5)	22(4)	17(3)
O8	52(3)	50(3)	28(2)	-12(2)	9(2)	-20(2)
O9	39(3)	45(3)	37(4)	12(3)	13(3)	-6(2)
O10	31(2)	38(2)	49(3)	10(2)	-4(2)	6(2)
C26	64(5)	32(3)	39(2)	2(2)	0(2)	-1(3)
S2A	27(1)	23(2)	23(1)	-1(1)	4(1)	-2(1)
F4A	168(8)	47(4)	58(5)	-13(3)	25(5)	29(5)
F5A	149(10)	52(6)	87(7)	2(4)	12(6)	-59(6)
F6A	137(7)	40(3)	57(5)	24(3)	-10(5)	11(4)
O8A	48(5)	77(9)	57(5)	-30(5)	31(4)	-16(4)
O9A	23(2)	40(3)	35(4)	-1(3)	2(3)	1(2)
O10A	41(3)	41(3)	37(4)	2(2)	-10(3)	-3(3)
C26A	66(5)	31(3)	40(2)	2(2)	-1(3)	-4(3)
S2B	28(1)	25(2)	23(1)	0(1)	2(1)	-2(1)
F4B	106(6)	33(4)	52(6)	-17(4)	20(5)	15(4)
F5B	91(7)	71(7)	105(10)	-6(6)	-9(6)	-47(6)

F6B	216(13)	32(3)	46(4)	20(3)	38(7)	15(5)
O8B	75(11)	44(5)	85(12)	8(8)	57(10)	16(8)
O9B	49(6)	57(7)	121(12)	-8(6)	-48(7)	1(5)
O10B	113(12)	62(6)	32(5)	25(4)	36(7)	23(9)
C26B	63(5)	31(3)	39(2)	0(2)	1(3)	0(3)

Table S-5. Hydrogen coordinates (x 10^4) and isotropic displacement parameters ($Å^2$ x 10^3) for jonap49.

	x	y	z	U(eq)
H1	3421(16)	4180(30)	4490(12)	57(9)
H4A	4690(30)	5960(60)	3250(30)	230(40)
H4B	4730(20)	6300(30)	3756(19)	115(16)
H1A	2293	5192	4093	49
H1B	2354	4090	4440	49
H3A	2873	2405	4027	56
H3B	2052	2197	3794	56
H4	3115	2967	2720	37
H5A	1310	3460	3306	60
H5B	1451	4743	3435	60
H6	2618	4598	2497	41
H8	3825	1098	3233	45
H9	4959	727	3673	47
H10	6098	1420	4206	47
H11	6735	2830	4672	48
H12	6210	4540	4742	45
H13	5069	4879	4307	40
H17	1531	6342	2517	64
H18	1542	8123	2829	71
H19	2144	9590	3458	66
H20	3051	10088	4137	66
H21	3910	8802	4505	62
H22	3883	7038	4167	53

Table S-6. Torsion angles [°] for jonap49.

Cu1-O1-C1-C2	39.9(2)	N3-C23-C24-C19	-179.6(2)
Cu1-N1-C2-C1	9.9(2)	C1-C2-C3-O2	-152.21(17)
Cu1-N1-C2-C3	-112.50(15)	C1-C2-C5-O3	130.92(19)
Cu1-N1-C2-C5	130.45(15)	C2-N1-C4-O2	17.14(18)
Cu1-N1-C4-O2	143.85(11)	C2-N1-C4-C7	-102.41(16)
Cu1-N1-C4-C7	24.30(16)	C2-N1-C6-O3	-33.72(19)
Cu1-N1-C6-O3	-158.06(12)	C2-N1-C6-C16	85.63(17)
Cu1-N1-C6-C16	-38.71(16)	C3-O2-C4-N1	-38.0(2)
Cu1-N2-C7-C4	-3.3(2)	C3-O2-C4-C7	81.6(2)
Cu1-N2-C7-C8	176.33(15)	C3-C2-C5-O3	-98.4(2)
Cu1-N2-C14-C13	8.3(3)	C4-O2-C3-C2	43.7(2)
Cu1-N2-C14-C15	-172.85(13)	C4-N1-C2-C1	131.37(18)
Cu1-N3-C16-C6	6.3(2)	C4-N1-C2-C3	9.00(18)
Cu1-N3-C16-C17	-176.73(18)	C4-N1-C2-C5	-108.04(18)
Cu1-N3-C23-C22	-6.7(3)	C4-N1-C6-O3	81.27(18)
Cu1-N3-C23-C24	176.22(15)	C4-N1-C6-C16	-159.38(14)
O1-C1-C2-N1	-35.0(3)	C4-C7-C8-C9	177.76(18)
O1-C1-C2-C3	79.7(2)	C5-O3-C6-N1	41.5(2)
O1-C1-C2-C5	-150.48(19)	C5-O3-C6-C16	-75.4(2)
O2-C4-C7-N2	-131.81(17)	C5-C2-C3-O2	78.3(2)
O2-C4-C7-C8	48.5(2)	C6-O3-C5-C2	-31.8(2)
O3-C6-C16-N3	138.31(17)	C6-N1-C2-C1	-107.45(19)
O3-C6-C16-C17	-38.7(3)	C6-N1-C2-C3	130.19(17)
N1-C2-C3-O2	-31.7(2)	C6-N1-C2-C5	13.1(2)
N1-C2-C5-O3	10.6(2)	C6-N1-C4-O2	-96.66(17)
N1-C4-C7-N2	-14.6(2)	C6-N1-C4-C7	143.80(15)
N1-C4-C7-C8	165.75(16)	C6-C16-C17-C18	175.8(2)
N1-C6-C16-N3	22.3(2)	C7-N2-C14-C13	-176.01(17)
N1-C6-C16-C17	-154.76(19)	C7-N2-C14-C15	2.9(3)
N2-C7-C8-C9	-1.9(3)	C7-C8-C9-C15	1.0(3)
N2-C14-C15-C9	-3.7(3)	C8-C9-C15-C10	-178.2(2)
N2-C14-C15-C10	176.22(17)	C8-C9-C15-C14	1.7(3)
N3-C16-C17-C18	-1.1(4)	C10-C11-C12-C13	-2.2(3)
N3-C23-C24-C18	1.9(3)	C11-C10-C15-C9	-177.8(2)

C11-C10-C15-C14	2.2(3)	O5A-S1A-C25A-F2A	-70.3(9)
C11-C12-C13-C14	-0.5(3)	O5A-S1A-C25A-F3A	53.9(10)
C12-C13-C14-N2	-177.08(18)	O6A-S1A-C25A-F1A	52.4(10)
C12-C13-C14-C15	4.0(3)	O6A-S1A-C25A-F2A	170.5(8)
C13-C14-C15-C9	175.19(19)	O6A-S1A-C25A-F3A	-65.4(9)
C13-C14-C15-C10	-4.9(3)	O7A-S1A-C25A-F1A	-69.7(10)
C14-N2-C7-C4	-179.72(15)	O7A-S1A-C25A-F2A	48.4(9)
C14-N2-C7-C8	-0.1(3)	O7A-S1A-C25A-F3A	172.5(8)
C15-C10-C11-C12	1.3(3)	O8-S2-C26-F4	-179.9(7)
C16-N3-C23-C22	174.0(2)	O8-S2-C26-F5	59.9(10)
C16-N3-C23-C24	-3.1(3)	O8-S2-C26-F6	-62.9(10)
C16-C17-C18-C24	-0.1(4)	O9-S2-C26-F4	-59.2(9)
C17-C18-C24-C19	-178.7(3)	O9-S2-C26-F5	-179.4(8)
C17-C18-C24-C23	-0.3(4)	O9-S2-C26-F6	57.8(10)
C19-C20-C21-C22	1.6(4)	O10-S2-C26-F4	61.0(9)
C20-C19-C24-C18	177.4(3)	O10-S2-C26-F5	-59.2(10)
C20-C19-C24-C23	-1.0(4)	O10-S2-C26-F6	178.0(8)
C20-C21-C22-C23	0.7(4)	O8A-S2A-C26A-F4A	175.9(10)
C21-C22-C23-N3	179.8(2)	O8A-S2A-C26A-F5A	53.4(11)
C21-C22-C23-C24	-3.1(4)	O8A-S2A-C26A-F6A	-67.1(11)
C22-C23-C24-C18	-175.2(2)	O9A-S2A-C26A-F4A	-64.5(11)
C22-C23-C24-C19	3.2(3)	O9A-S2A-C26A-F5A	173.0(9)
C23-N3-C16-C6	-174.26(17)	O9A-S2A-C26A-F6A	52.5(11)
C23-N3-C16-C17	2.7(3)	O10A-S2A-C26A-F4A	54.6(11)
C24-C19-C20-C21	-1.4(4)	O10A-S2A-C26A-F5A	-67.9(10)
O5-S1-C25-F1	173.0(10)	O10A-S2A-C26A-F6A	171.6(9)
O5-S1-C25-F2	-63.8(8)	O8B-S2B-C26B-F4B	179.0(12)
O5-S1-C25-F3	51.7(8)	O8B-S2B-C26B-F5B	60.7(15)
O6-S1-C25-F1	53.1(10)	O8B-S2B-C26B-F6B	-60.1(15)
O6-S1-C25-F2	176.3(7)	O9B-S2B-C26B-F4B	-61.0(14)
O6-S1-C25-F3	-68.2(8)	O9B-S2B-C26B-F5B	-179.4(13)
O7-S1-C25-F1	-68.8(10)	O9B-S2B-C26B-F6B	59.9(15)
O7-S1-C25-F2	54.4(8)	O10B-S2B-C26B-F4B	60.0(13)
O7-S1-C25-F3	169.9(7)	O10B-S2B-C26B-F5B	-58.3(14)
O5A-S1A-C25A-F1A	171.6(10)	O10B-S2B-C26B-F6B	-179.1(12)

Table S-7. Hydrogen bonds and close contacts for jonap49 [Å and °].

D-H...A	d(D-H)	d(H...A)	d(D...A)	<(DHA)
O1-H1...O10	0.74(3)	1.94(3)	2.632(4)	156(3)
O1-H1...O10A	0.74(3)	1.90(3)	2.636(9)	173(3)
O1-H1...O10B	0.74(3)	2.11(3)	2.836(15)	165(3)
O4-H4A...O7	0.86(6)	2.11(7)	2.923(5)	159(6)
O4-H4A...O7A	0.86(6)	1.61(7)	2.448(5)	165(7)
O4-H4B...O8#1	0.76(4)	2.10(4)	2.752(5)	144(5)
O4-H4B...O9A#1	0.76(4)	2.19(5)	2.915(7)	162(4)
O4-H4B...O9B#1	0.76(4)	1.97(4)	2.713(13)	168(5)

Symmetry transformations used to generate equivalent atoms:

#1 -x+1,-y+1,-z+1

REFERENCE NUMBER: jonap30

3 (1st polymorph)

CRYSTAL STRUCTURE REPORT

C_{27} H_{24} Cu F_3 N_4 O_6 S

or

[(κ^3-L^q)Cu(NCMe)][OTf]

Report prepared for:

R. Zhang, A. Panda, Prof. W. Jones

November 06, 2023

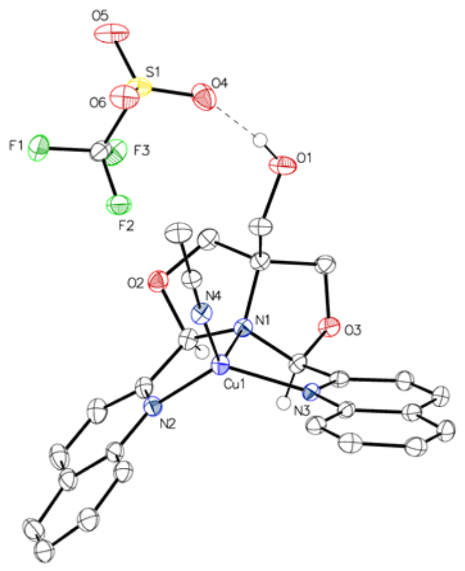

William W. Brennessel

X-ray Crystallographic Facility

Department of Chemistry, University of Rochester

120 Trustee Road

Rochester, NY 14627

Data collection

A crystal (0.218 x 0.114 x 0.029 mm^3) was placed onto a nylon loop and mounted on a Rigaku XtaLAB Synergy-S Dualflex diffractometer equipped with a HyPix-6000HE HPC area detector for data collection at 100.00(10) K. A preliminary set of cell constants and an orientation matrix were calculated from a small sampling of reflections.[1] A short pre-experiment was run, from which an optimal data collection strategy was determined. The full data collection was carried out using a PhotonJet (Cu) X-ray source with frame times of 0.43 and 1.71 seconds and a detector distance of 34.0 mm. Series of frames were collected in 0.50º steps in ω at different 2θ, κ, and ϕ settings. After the intensity data were corrected for absorption, the final cell constants were calculated from the xyz centroids of 22483 strong reflections from the actual data collection after integration.[1] See Table S-1 for additional crystal and refinement information.

Structure solution and refinement

The structure was solved using SHELXT[2] and refined using SHELXL.[3] The space group $Pna2_1$ was determined based on systematic absences and intensity statistics. Most or all non-hydrogen atoms were assigned from the solution. Full-matrix least squares / difference Fourier cycles were performed which located any remaining non-hydrogen atoms. All non-hydrogen atoms were refined with anisotropic displacement parameters. The O-H hydrogen atom was found from the difference Fourier map and refined freely. All other hydrogen atoms were placed in ideal positions and refined as riding atoms with relative isotropic displacement parameters. The final full matrix least squares refinement converged to $R1 = 0.0269$ (F^2, $I > 2\sigma(I)$) and $wR2 = 0.0674$ (F^2, all data).

Structure description

The structure is the one suggested. The asymmetric unit contains one monocationic Cu complex and one triflate anion in general positions. The cation and anion are linked via hydrogen bonding (see figures and Table S-7).

Structure manipulation and figure generation were performed using Olex2.[4] Unless noted otherwise all structural diagrams containing anisotropic displacement ellipsoids are drawn at the 50 % probability level.

Data collection, structure solution, and structure refinement were conducted at the X-ray Crystallographic Facility, B04 Hutchison Hall, Department of Chemistry, University of Rochester. The instrument was purchased with funding from NSF MRI program grant CHE-1725028. All publications arising from this report MUST either 1) include William W. Brennessel as a coauthor or 2) acknowledge William W. Brennessel and the X-ray Crystallographic Facility of the Department of Chemistry at the University of Rochester.

1 *CrysAlisPro*, version 171.42.101a; Rigaku Corporation: Oxford, UK, 2023.

2 Sheldrick, G. M. *SHELXT*, version 2018/2; *Acta. Crystallogr.* **2015**, *A71*, 3-8.

3 Sheldrick, G. M. *SHELXL*, version 2019/2; *Acta. Crystallogr.* **2015**, *C71*, 3-8.

4 Dolomanov, O. V.; Bourhis, L. J.; Gildea, R. J.; Howard, J. A. K.; Puschmann, H. *Olex2*, version 1.5; *J. Appl. Cryst.* **2009**, *42*, 339-341.

Some equations of interest:

$$R_{int} = \Sigma |F_o^2 - < F_o^2 >| / \Sigma|F_o^2|$$

$$R1 = \Sigma||F_o|-|F_c|| / \Sigma|F_o|$$

$$wR2 = [\Sigma[w(F_o^2-F_c^2)^2] / \Sigma [w(F_o^2)^2]]^{1/2}$$

where $w = 1 / [\sigma^2 (F_o^2) + (aP)^2 + bP]$ and

$$P = 1/3 \max (0, F_o^2) + 2/3 F_c^2$$

$$\text{GOF} = S = [\Sigma[w(F_o^2-F_c^2)^2] / (m-n)]^{1/2}$$

where m = number of reflections and n = number of parameters

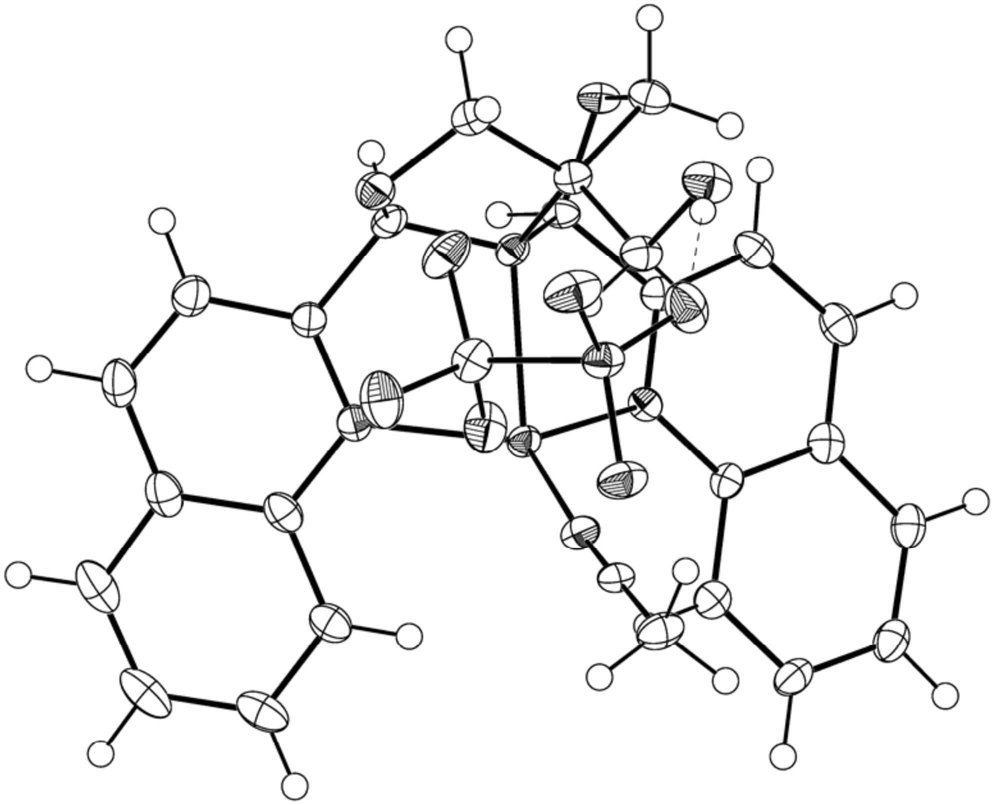

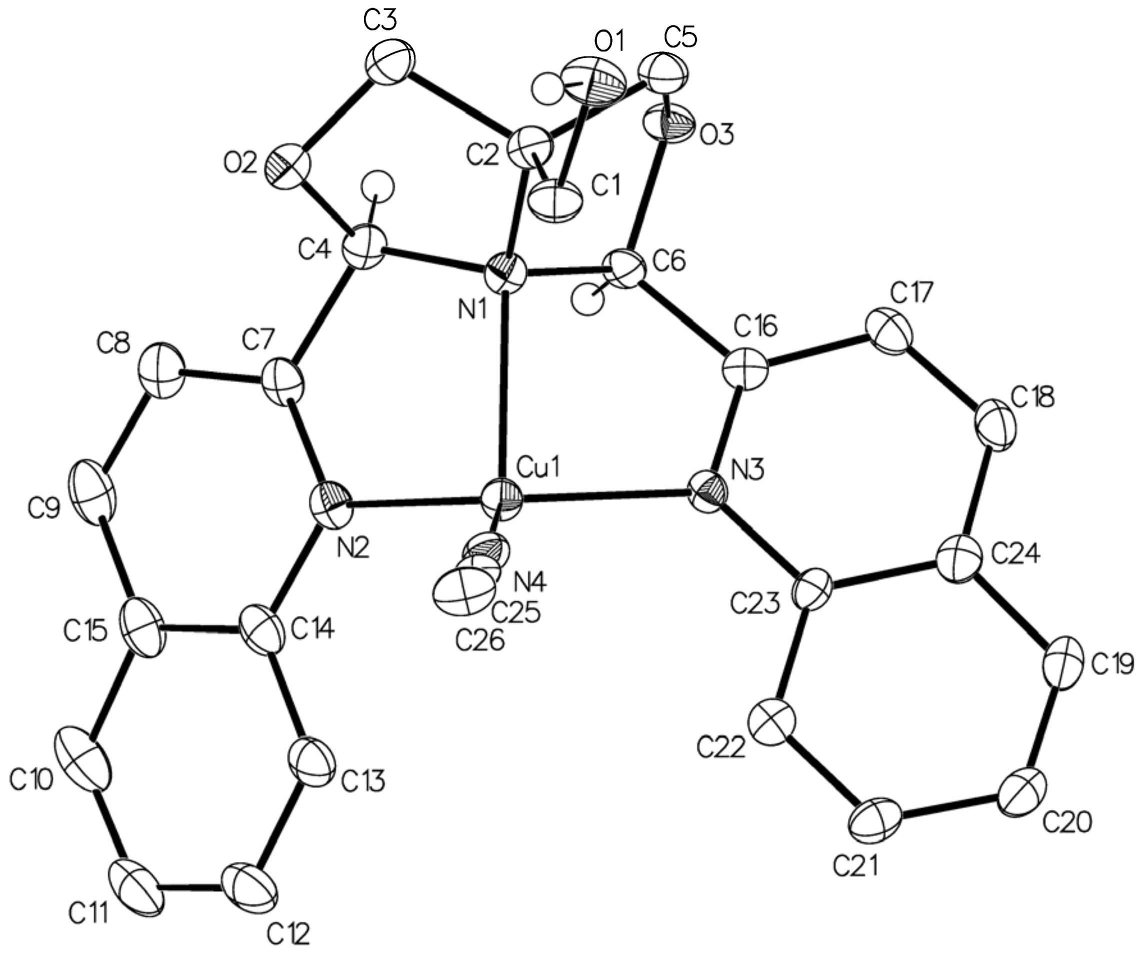

C3
O1
C5
O3
O2
C2
C1
C4
C6
N1
C17
C8
C7
C16
C18
Cu1
N3
C9
N2
N4
C24
C25
C23
C15
C14
C26
C19
C22
C10
C13
C20
C21
C11
C12

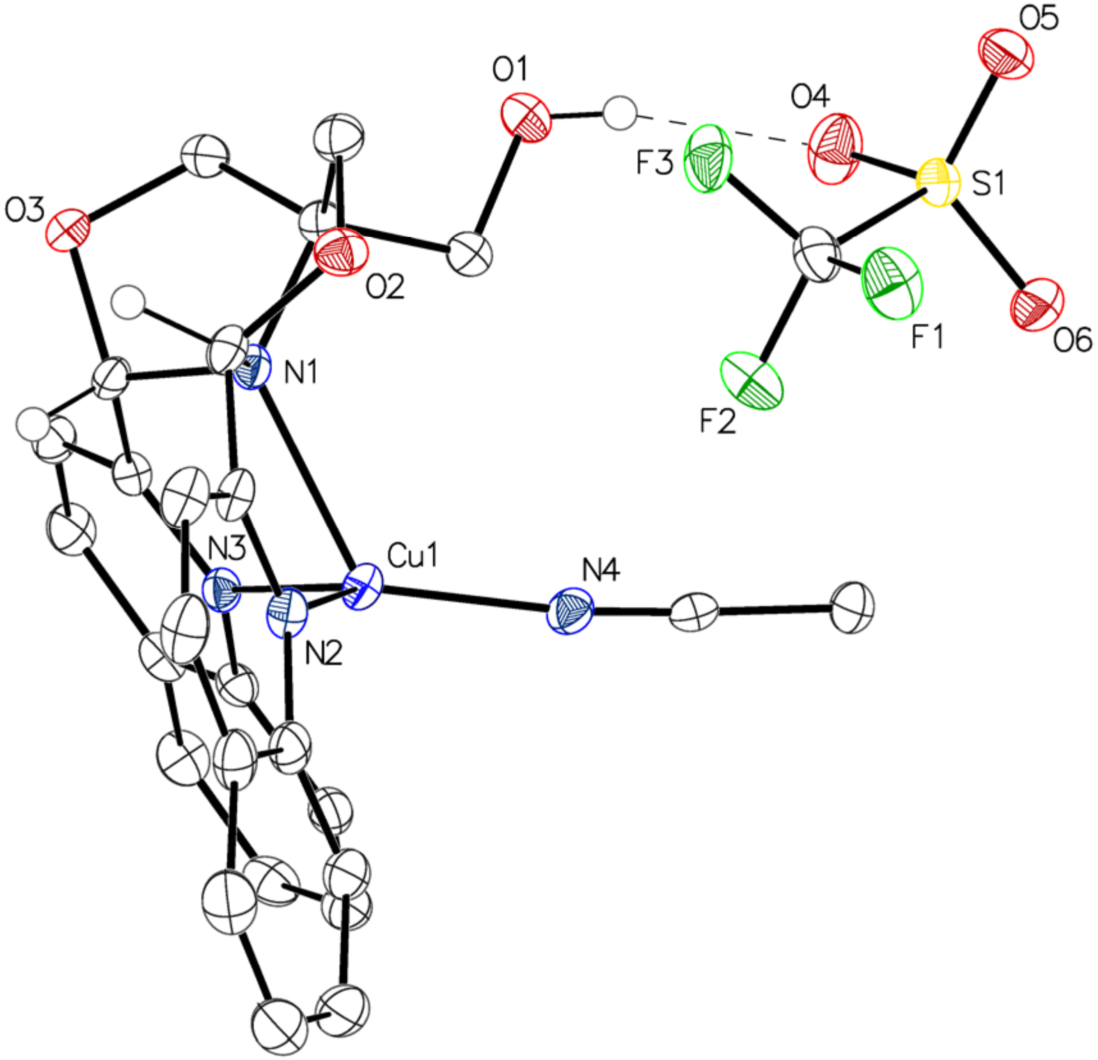
O5
O1
O4
F3
S1
O3
O2
F1
O6
N1
F2
Cu1
N3
N4
N2

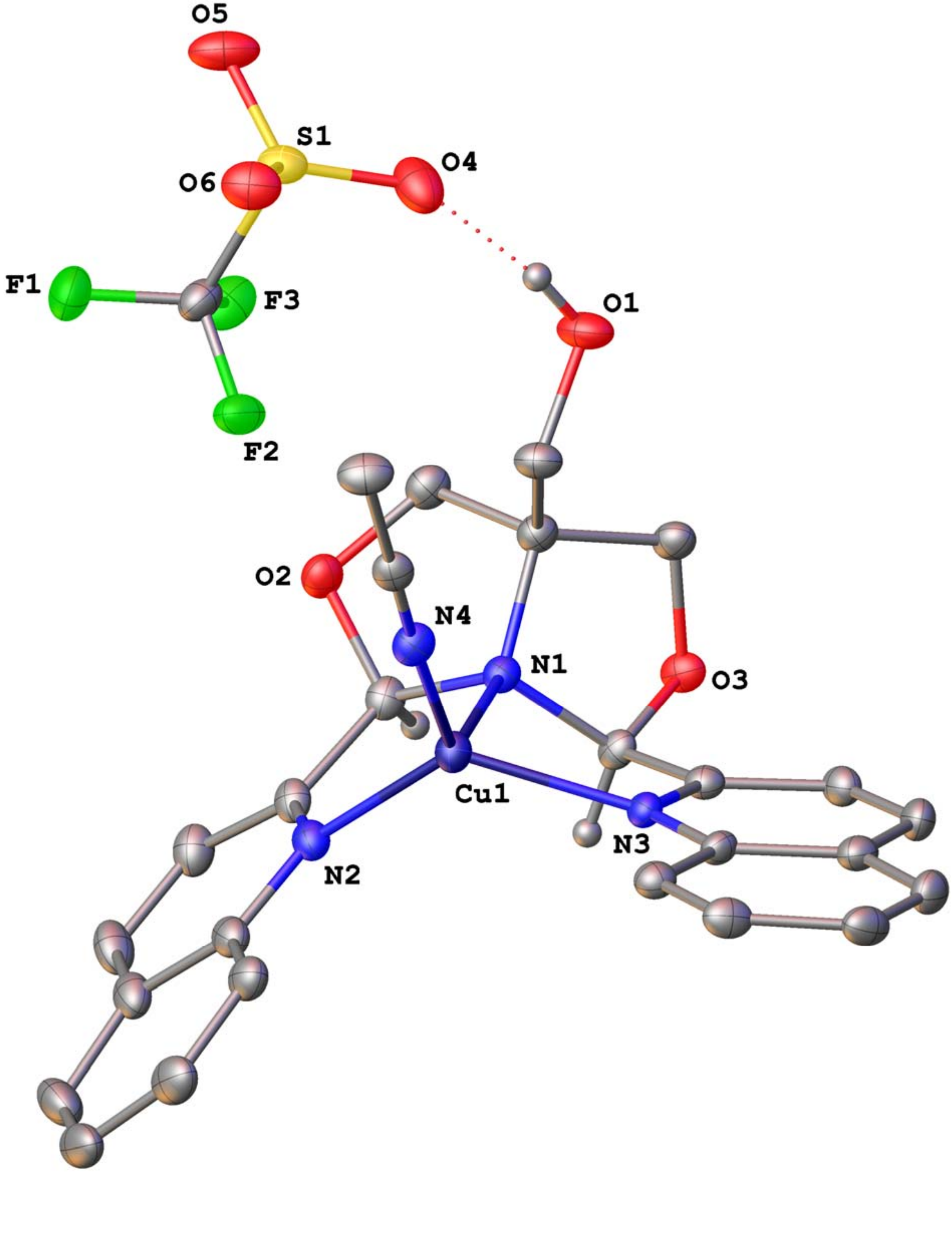
O5
S1
O4
O6
F1
F3
O1
F2
O2
N4
N1
O3
Cu1
N3
N2

Table S-1. Crystal data and structure refinement for jonap30.

Identification code	jonap30	
Empirical formula	C27 H24 Cu F3 N4 O6 S	
Formula weight	653.10	
Temperature	100.00(10) K	
Wavelength	1.54184 Å	
Crystal system	orthorhombic	
Space group	$Pna2_1$	
Unit cell dimensions	a = 15.51580(10) Å	α = 90°
	b = 10.25970(10) Å	β = 90°
	c = 16.85380(10) Å	γ = 90°
Volume	2682.91(4) Å^3	
Z	4	
Density (calculated)	1.617 Mg/m^3	
Absorption coefficient	2.519 mm^{-1}	
F(000)	1336	
Crystal color, morphology	yellow-blue, plate	
Crystal size	0.218 x 0.114 x 0.029 mm^3	
Theta range for data collection	5.047 to 80.060°	
Index ranges	$-19 \leq h \leq 19$, $-10 \leq k \leq 12$, $-21 \leq l \leq 21$	
Reflections collected	31805	
Independent reflections	5682 [R(int) = 0.0411]	
Observed reflections	5539	
Completeness to theta = 74.504°	100.0%	
Absorption correction	Multi-scan	
Max. and min. transmission	1.00000 and 0.90736	
Refinement method	Full-matrix least-squares on F^2	
Data / restraints / parameters	5682 / 1 / 384	
Goodness-of-fit on F^2	1.029	
Final R indices [I>2sigma(I)]	$R1$ = 0.0227, $wR2$ = 0.0572	
R indices (all data)	$R1$ = 0.0235, $wR2$ = 0.0577	
Absolute structure parameter	-0.002(7)	
Largest diff. peak and hole	0.347 and -0.203 e.Å^{-3}	

Table S-2. Atomic coordinates (x 10^4) and equivalent isotropic displacement parameters ($Å^2$x 10^3) for jonap30. U_{eq} is defined as one third of the trace of the orthogonalized U_{ij} tensor.

	x	y	z	U_{eq}
Cu1	5363(1)	5930(1)	4988(1)	14(1)
O1	8070(1)	6406(2)	6155(1)	25(1)
O2	7039(1)	8154(2)	4197(1)	19(1)
O3	6131(1)	9354(2)	6186(1)	17(1)
N1	6100(1)	7755(2)	5214(1)	13(1)
N2	5104(1)	6889(2)	3973(1)	15(1)
N3	4819(1)	6457(2)	6042(1)	13(1)
N4	5866(1)	4216(2)	4985(2)	16(1)
C1	7263(2)	6376(2)	5747(2)	18(1)
C2	6992(2)	7758(2)	5567(2)	15(1)
C3	7534(2)	8382(2)	4902(2)	18(1)
C4	6188(2)	8419(2)	4441(1)	16(1)
C5	6900(2)	8599(2)	6318(2)	18(1)
C6	5566(2)	8491(2)	5780(1)	14(1)
C7	5552(2)	7952(2)	3820(2)	16(1)
C8	5460(2)	8685(2)	3118(2)	21(1)
C9	4857(2)	8306(3)	2570(2)	24(1)
C10	3671(2)	6795(3)	2212(2)	29(1)
C11	3189(2)	5705(3)	2387(2)	29(1)
C12	3386(2)	4959(3)	3065(2)	27(1)
C13	4026(2)	5336(3)	3578(2)	20(1)
C14	4502(2)	6480(2)	3425(2)	18(1)
C15	4344(2)	7200(3)	2721(2)	21(1)
C16	5096(2)	7568(2)	6345(1)	14(1)
C17	4955(2)	7938(2)	7140(2)	17(1)
C18	4510(2)	7113(3)	7629(2)	19(1)
C19	3697(2)	5038(2)	7789(2)	20(1)
C20	3382(2)	3912(3)	7457(2)	20(1)
C21	3562(2)	3616(2)	6655(2)	18(1)
C22	4033(2)	4450(2)	6192(2)	16(1)
C23	4348(1)	5629(2)	6517(1)	14(1)

C24	4182(2)	5930(2)	7322(2)	17(1)
C25	6229(1)	3250(2)	5006(2)	16(1)
C26	6727(2)	2047(2)	5026(2)	22(1)
S1	4134(1)	1343(1)	4703(1)	19(1)
F1	3635(1)	754(2)	3253(1)	31(1)
F2	2642(1)	613(2)	4144(1)	26(1)
F3	3622(1)	-865(2)	4071(1)	28(1)
O4	3914(2)	765(2)	5458(1)	31(1)
O5	5003(1)	1092(2)	4442(2)	30(1)
O6	3831(1)	2655(2)	4595(1)	26(1)
C27	3479(2)	414(2)	4005(2)	20(1)

Table S-3. Bond lengths [Å] and angles [°] for jonap30.

Cu(1)-N(1)	2.2272(19)	C(9)-H(9)	0.9500
Cu(1)-N(2)	2.014(2)	C(9)-C(15)	1.409(4)
Cu(1)-N(3)	2.041(2)	C(10)-H(10)	0.9500
Cu(1)-N(4)	1.9240(18)	C(10)-C(11)	1.377(5)
O(1)-C(1)	1.428(3)	C(10)-C(15)	1.414(4)
O(1)-H(1)	0.81(5)	C(11)-H(11)	0.9500
O(2)-C(3)	1.434(3)	C(11)-C(12)	1.409(4)
O(2)-C(4)	1.410(3)	C(12)-H(12)	0.9500
O(3)-C(5)	1.440(3)	C(12)-C(13)	1.372(4)
O(3)-C(6)	1.421(3)	C(13)-H(13)	0.9500
N(1)-C(2)	1.506(3)	C(13)-C(14)	1.411(4)
N(1)-C(4)	1.477(3)	C(14)-C(15)	1.419(4)
N(1)-C(6)	1.472(3)	C(16)-C(17)	1.410(3)
N(2)-C(7)	1.319(3)	C(17)-H(17)	0.9500
N(2)-C(14)	1.379(3)	C(17)-C(18)	1.369(4)
N(3)-C(16)	1.321(3)	C(18)-H(18)	0.9500
N(3)-C(23)	1.377(3)	C(18)-C(24)	1.414(4)
N(4)-C(25)	1.140(3)	C(19)-H(19)	0.9500
C(1)-H(1A)	0.9900	C(19)-C(20)	1.374(4)
C(1)-H(1B)	0.9900	C(19)-C(24)	1.422(4)
C(1)-C(2)	1.510(3)	C(20)-H(20)	0.9500
C(2)-C(3)	1.539(3)	C(20)-C(21)	1.413(4)
C(2)-C(5)	1.538(3)	C(21)-H(21)	0.9500
C(3)-H(3A)	0.9900	C(21)-C(22)	1.370(3)
C(3)-H(3B)	0.9900	C(22)-H(22)	0.9500
C(4)-H(4)	1.0000	C(22)-C(23)	1.414(3)
C(4)-C(7)	1.517(3)	C(23)-C(24)	1.415(3)
C(5)-H(5A)	0.9900	C(25)-C(26)	1.457(3)
C(5)-H(5B)	0.9900	C(26)-H(26A)	0.9800
C(6)-H(6)	1.0000	C(26)-H(26B)	0.9800
C(6)-C(16)	1.528(3)	C(26)-H(26C)	0.9800
C(7)-C(8)	1.409(3)	S(1)-O(4)	1.444(2)
C(8)-H(8)	0.9500	S(1)-O(5)	1.441(2)
C(8)-C(9)	1.370(4)	S(1)-O(6)	1.4372(18)

S(1)-C(27)	1.823(3)
F(1)-C(27)	1.336(3)
F(2)-C(27)	1.336(3)
F(3)-C(27)	1.336(3)
N(2)-Cu(1)-N(1)	80.63(8)
N(2)-Cu(1)-N(3)	121.83(8)
N(3)-Cu(1)-N(1)	80.79(7)
N(4)-Cu(1)-N(1)	124.11(7)
N(4)-Cu(1)-N(2)	121.71(9)
N(4)-Cu(1)-N(3)	114.35(9)
C(1)-O(1)-H(1)	110(4)
C(4)-O(2)-C(3)	103.16(18)
C(6)-O(3)-C(5)	104.47(16)
C(2)-N(1)-Cu(1)	122.78(14)
C(4)-N(1)-Cu(1)	106.48(13)
C(4)-N(1)-C(2)	105.16(18)
C(6)-N(1)-Cu(1)	104.70(13)
C(6)-N(1)-C(2)	105.14(18)
C(6)-N(1)-C(4)	112.76(17)
C(7)-N(2)-Cu(1)	117.75(17)
C(7)-N(2)-C(14)	118.5(2)
C(14)-N(2)-Cu(1)	123.74(17)
C(16)-N(3)-Cu(1)	115.49(16)
C(16)-N(3)-C(23)	118.8(2)
C(23)-N(3)-Cu(1)	124.18(15)
C(25)-N(4)-Cu(1)	173.98(19)
O(1)-C(1)-H(1A)	109.9
O(1)-C(1)-H(1B)	109.9
O(1)-C(1)-C(2)	108.76(19)
H(1A)-C(1)-H(1B)	108.3
C(2)-C(1)-H(1A)	109.9
C(2)-C(1)-H(1B)	109.9
N(1)-C(2)-C(1)	109.49(19)
N(1)-C(2)-C(3)	102.49(19)
N(1)-C(2)-C(5)	103.89(18)
C(1)-C(2)-C(3)	112.6(2)
C(1)-C(2)-C(5)	112.7(2)
C(5)-C(2)-C(3)	114.6(2)
O(2)-C(3)-C(2)	104.08(18)
O(2)-C(3)-H(3A)	110.9
O(2)-C(3)-H(3B)	110.9
C(2)-C(3)-H(3A)	110.9
C(2)-C(3)-H(3B)	110.9
H(3A)-C(3)-H(3B)	109.0
O(2)-C(4)-N(1)	104.79(18)
O(2)-C(4)-H(4)	109.3
O(2)-C(4)-C(7)	110.4(2)
N(1)-C(4)-H(4)	109.3
N(1)-C(4)-C(7)	113.80(19)
C(7)-C(4)-H(4)	109.3
O(3)-C(5)-C(2)	104.60(19)
O(3)-C(5)-H(5A)	110.8
O(3)-C(5)-H(5B)	110.8
C(2)-C(5)-H(5A)	110.8
C(2)-C(5)-H(5B)	110.8
H(5A)-C(5)-H(5B)	108.9
O(3)-C(6)-N(1)	106.52(18)
O(3)-C(6)-H(6)	109.1
O(3)-C(6)-C(16)	112.33(19)
N(1)-C(6)-H(6)	109.1
N(1)-C(6)-C(16)	110.71(18)
C(16)-C(6)-H(6)	109.1
N(2)-C(7)-C(4)	117.9(2)
N(2)-C(7)-C(8)	123.6(2)
C(8)-C(7)-C(4)	118.5(2)
C(7)-C(8)-H(8)	120.5
C(9)-C(8)-C(7)	118.9(2)
C(9)-C(8)-H(8)	120.5
C(8)-C(9)-H(9)	120.3
C(8)-C(9)-C(15)	119.5(2)
C(15)-C(9)-H(9)	120.3
C(11)-C(10)-H(10)	119.7

C(11)-C(10)-C(15)	120.7(3)	C(20)-C(21)-H(21)	119.5
C(15)-C(10)-H(10)	119.7	C(22)-C(21)-C(20)	121.0(2)
C(10)-C(11)-H(11)	120.1	C(22)-C(21)-H(21)	119.5
C(10)-C(11)-C(12)	119.8(3)	C(21)-C(22)-H(22)	120.1
C(12)-C(11)-H(11)	120.1	C(21)-C(22)-C(23)	119.9(2)
C(11)-C(12)-H(12)	119.5	C(23)-C(22)-H(22)	120.1
C(13)-C(12)-C(11)	121.0(3)	N(3)-C(23)-C(22)	119.1(2)
C(13)-C(12)-H(12)	119.5	N(3)-C(23)-C(24)	121.2(2)
C(12)-C(13)-H(13)	120.1	C(22)-C(23)-C(24)	119.7(2)
C(12)-C(13)-C(14)	119.8(3)	C(18)-C(24)-C(19)	122.7(2)
C(14)-C(13)-H(13)	120.1	C(18)-C(24)-C(23)	118.2(2)
N(2)-C(14)-C(13)	119.0(2)	C(23)-C(24)-C(19)	119.1(2)
N(2)-C(14)-C(15)	121.2(2)	N(4)-C(25)-C(26)	177.6(2)
C(13)-C(14)-C(15)	119.8(2)	C(25)-C(26)-H(26A)	109.5
C(9)-C(15)-C(10)	123.0(3)	C(25)-C(26)-H(26B)	109.5
C(9)-C(15)-C(14)	118.2(2)	C(25)-C(26)-H(26C)	109.5
C(10)-C(15)-C(14)	118.8(3)	H(26A)-C(26)-H(26B)	109.5
N(3)-C(16)-C(6)	116.7(2)	H(26A)-C(26)-H(26C)	109.5
N(3)-C(16)-C(17)	123.3(2)	H(26B)-C(26)-H(26C)	109.5
C(17)-C(16)-C(6)	120.0(2)	O(4)-S(1)-C(27)	102.85(12)
C(16)-C(17)-H(17)	120.5	O(5)-S(1)-O(4)	114.59(14)
C(18)-C(17)-C(16)	119.0(2)	O(5)-S(1)-C(27)	103.34(12)
C(18)-C(17)-H(17)	120.5	O(6)-S(1)-O(4)	114.76(13)
C(17)-C(18)-H(18)	120.3	O(6)-S(1)-O(5)	115.74(12)
C(17)-C(18)-C(24)	119.5(2)	O(6)-S(1)-C(27)	103.09(12)
C(24)-C(18)-H(18)	120.3	F(1)-C(27)-S(1)	112.03(17)
C(20)-C(19)-H(19)	119.8	F(2)-C(27)-S(1)	110.41(17)
C(20)-C(19)-C(24)	120.3(2)	F(2)-C(27)-F(1)	107.6(2)
C(24)-C(19)-H(19)	119.8	F(3)-C(27)-S(1)	111.60(19)
C(19)-C(20)-H(20)	120.0	F(3)-C(27)-F(1)	107.8(2)
C(19)-C(20)-C(21)	120.0(2)	F(3)-C(27)-F(2)	107.2(2)
C(21)-C(20)-H(20)	120.0		

Table S-4. Anisotropic displacement parameters ($Å^2$x 10^3) for jonap30. The anisotropic displacement factor exponent takes the form: $-2\pi^2[h^2 a^{*2}U_{11} + ... + 2 h k a^* b^* U_{12}]$

	U_{11}	U_{22}	U_{33}	U_{23}	U_{13}	U_{12}
Cu1	17(1)	10(1)	14(1)	0(1)	1(1)	0(1)
O1	16(1)	23(1)	35(1)	-3(1)	-7(1)	4(1)
O2	18(1)	20(1)	18(1)	0(1)	3(1)	-3(1)
O3	17(1)	12(1)	22(1)	-4(1)	-1(1)	-1(1)
N1	14(1)	12(1)	13(1)	0(1)	1(1)	1(1)
N2	19(1)	13(1)	14(1)	-1(1)	0(1)	3(1)
N3	12(1)	13(1)	14(1)	-1(1)	1(1)	2(1)
N4	18(1)	12(1)	19(1)	-1(1)	2(1)	-1(1)
C1	15(1)	15(1)	23(1)	0(1)	-3(1)	1(1)
C2	14(1)	15(1)	17(1)	-1(1)	1(1)	-1(1)
C3	18(1)	17(1)	20(1)	1(1)	3(1)	-3(1)
C4	20(1)	11(1)	15(1)	1(1)	2(1)	0(1)
C5	15(1)	18(1)	20(1)	-4(1)	-1(1)	-2(1)
C6	14(1)	11(1)	17(1)	-3(1)	2(1)	0(1)
C7	22(1)	10(1)	14(1)	-2(1)	1(1)	3(1)
C8	30(1)	15(1)	17(1)	1(1)	2(1)	3(1)
C9	38(1)	20(1)	15(1)	0(1)	-2(1)	10(1)
C10	32(2)	34(2)	20(1)	-8(1)	-9(1)	12(1)
C11	22(1)	42(2)	24(1)	-18(1)	-5(1)	5(1)
C12	22(1)	30(1)	28(1)	-15(1)	0(1)	-1(1)
C13	20(1)	22(1)	19(1)	-6(1)	2(1)	2(1)
C14	18(1)	18(1)	17(1)	-6(1)	0(1)	4(1)
C15	27(1)	22(1)	14(1)	-5(1)	-2(1)	8(1)
C16	13(1)	13(1)	15(1)	0(1)	0(1)	2(1)
C17	16(1)	19(1)	17(1)	-6(1)	-2(1)	2(1)
C18	19(1)	25(1)	13(1)	-4(1)	1(1)	3(1)
C19	19(1)	25(1)	15(1)	3(1)	3(1)	2(1)
C20	15(1)	24(1)	21(1)	9(1)	1(1)	-1(1)
C21	16(1)	15(1)	24(1)	5(1)	-1(1)	-1(1)
C22	15(1)	16(1)	18(1)	1(1)	0(1)	1(1)
C23	11(1)	16(1)	14(1)	2(1)	2(1)	1(1)

C24	12(1)	21(1)	17(1)	2(1)	0(1)	3(1)
C25	15(1)	15(1)	18(1)	0(1)	0(1)	-5(1)
C26	18(1)	13(1)	34(1)	2(1)	-1(1)	2(1)
S1	16(1)	14(1)	26(1)	-1(1)	-3(1)	-2(1)
F1	33(1)	40(1)	20(1)	2(1)	6(1)	-8(1)
F2	16(1)	37(1)	26(1)	-6(1)	0(1)	-3(1)
F3	32(1)	17(1)	34(1)	-7(1)	8(1)	-7(1)
O4	41(1)	26(1)	25(1)	2(1)	-8(1)	-10(1)
O5	16(1)	22(1)	53(1)	-4(1)	-4(1)	-1(1)
O6	24(1)	16(1)	37(1)	-2(1)	-3(1)	1(1)
C27	18(1)	21(1)	21(1)	-2(1)	4(1)	-4(1)

Table S-5. Hydrogen coordinates (x 10^4) and isotropic displacement parameters ($Å^2$ x 10^3) for jonap30.

	x	y	z	U(eq)
H1A	6823	5946	6081	21
H1B	7321	5876	5248	21
H3A	7611	9327	4996	22
H3B	8107	7964	4865	22
H4	6118	9379	4518	19
H5A	6839	8045	6795	21
H5B	7407	9174	6386	21
H6	5131	9014	5479	17
H8	5811	9428	3025	25
H9	4786	8785	2092	29
H10	3549	7280	1745	35
H11	2725	5457	2052	35
H12	3071	4183	3168	32
H13	4148	4827	4036	24
H17	5166	8748	7333	21
H18	4422	7333	8171	23
H19	3591	5222	8332	24
H20	3042	3333	7767	24
H21	3352	2825	6434	22
H22	4149	4237	5654	20
H26A	6464	1439	5403	33
H26B	7319	2238	5191	33
H26C	6733	1653	4496	33
H1	8390(40)	5860(50)	5980(30)	60(15)

Table S-6. Torsion angles [°] for jonap30.

Cu1-N1-C2-C1	-1.5(3)	C1-C2-C3-O2	94.4(2)
Cu1-N1-C2-C3	118.21(16)	C1-C2-C5-O3	-138.67(19)
Cu1-N1-C2-C5	-122.20(17)	C2-N1-C4-O2	29.5(2)
Cu1-N1-C4-O2	-102.23(16)	C2-N1-C4-C7	150.10(19)
Cu1-N1-C4-C7	18.4(2)	C2-N1-C6-O3	25.9(2)
Cu1-N1-C6-O3	156.55(14)	C2-N1-C6-C16	-96.5(2)
Cu1-N1-C6-C16	34.2(2)	C3-O2-C4-N1	-45.1(2)
Cu1-N2-C7-C4	-3.7(3)	C3-O2-C4-C7	-167.95(18)
Cu1-N2-C7-C8	177.33(19)	C3-C2-C5-O3	90.8(2)
Cu1-N2-C14-C13	0.9(3)	C4-O2-C3-C2	42.3(2)
Cu1-N2-C14-C15	179.74(18)	C4-N1-C2-C1	-123.1(2)
Cu1-N3-C16-C6	17.5(3)	C4-N1-C2-C3	-3.4(2)
Cu1-N3-C16-C17	-164.55(18)	C4-N1-C2-C5	116.19(19)
Cu1-N3-C23-C22	-16.2(3)	C4-N1-C6-O3	-88.1(2)
Cu1-N3-C23-C24	163.10(17)	C4-N1-C6-C16	149.50(19)
O1-C1-C2-N1	-174.03(19)	C4-C7-C8-C9	-176.9(2)
O1-C1-C2-C3	72.6(3)	C5-O3-C6-N1	-39.7(2)
O1-C1-C2-C5	-58.9(3)	C5-O3-C6-C16	81.7(2)
O2-C4-C7-N2	106.0(2)	C5-C2-C3-O2	-134.9(2)
O2-C4-C7-C8	-75.0(3)	C6-O3-C5-C2	36.7(2)
O3-C6-C16-N3	-155.79(19)	C6-N1-C2-C1	117.6(2)
O3-C6-C16-C17	26.2(3)	C6-N1-C2-C3	-122.65(18)
N1-C2-C3-O2	-23.1(2)	C6-N1-C2-C5	-3.1(2)
N1-C2-C5-O3	-20.2(2)	C6-N1-C4-O2	143.50(18)
N1-C4-C7-N2	-11.5(3)	C6-N1-C4-C7	-95.9(2)
N1-C4-C7-C8	167.6(2)	C6-C16-C17-C18	178.0(2)
N1-C6-C16-N3	-36.8(3)	C7-N2-C14-C13	179.3(2)
N1-C6-C16-C17	145.1(2)	C7-N2-C14-C15	-1.9(3)
N2-C7-C8-C9	2.1(4)	C7-C8-C9-C15	0.1(4)
N2-C14-C15-C9	3.9(4)	C8-C9-C15-C10	175.7(3)
N2-C14-C15-C10	-174.8(2)	C8-C9-C15-C14	-2.9(4)
N3-C16-C17-C18	0.1(4)	C10-C11-C12-C13	3.2(4)
N3-C23-C24-C18	0.3(3)	C11-C10-C15-C9	179.9(3)
N3-C23-C24-C19	-179.6(2)	C11-C10-C15-C14	-1.5(4)

C11-C12-C13-C14	-0.6(4)	C21-C22-C23-N3	-179.8(2)
C12-C13-C14-N2	175.8(2)	C21-C22-C23-C24	0.8(3)
C12-C13-C14-C15	-3.0(4)	C22-C23-C24-C18	179.7(2)
C13-C14-C15-C9	-177.3(2)	C22-C23-C24-C19	-0.3(3)
C13-C14-C15-C10	4.0(4)	C23-N3-C16-C6	-176.2(2)
C14-N2-C7-C4	177.8(2)	C23-N3-C16-C17	1.8(3)
C14-N2-C7-C8	-1.1(3)	C24-C19-C20-C21	2.0(4)
C15-C10-C11-C12	-2.1(4)	O4-S1-C27-F1	-176.02(19)
C16-N3-C23-C22	178.7(2)	O4-S1-C27-F2	64.1(2)
C16-N3-C23-C24	-2.0(3)	O4-S1-C27-F3	-55.0(2)
C16-C17-C18-C24	-1.8(4)	O5-S1-C27-F1	-56.5(2)
C17-C18-C24-C19	-178.5(2)	O5-S1-C27-F2	-176.36(18)
C17-C18-C24-C23	1.6(3)	O5-S1-C27-F3	64.5(2)
C19-C20-C21-C22	-1.5(4)	O6-S1-C27-F1	64.4(2)
C20-C19-C24-C18	178.9(2)	O6-S1-C27-F2	-55.5(2)
C20-C19-C24-C23	-1.2(4)	O6-S1-C27-F3	-174.63(18)
C20-C21-C22-C23	0.0(4)		

Table S-7. Hydrogen bonds and close contacts for jonap30 [Å and °].

D-H...A	d(D-H)	d(H...A)	d(D...A)	<(DHA)
O1-H1...O4#1	0.81(5)	2.05(5)	2.839(3)	165(5)

Symmetry transformations used to generate equivalent atoms:

#1 x+1/2,-y+1/2,z

REFERENCE NUMBER: jonap31

3 (2nd polymorph)

CRYSTAL STRUCTURE REPORT

$C_{27}H_{24}CuF_3N_4O_6S$

or

[(κ^3-L^q)Cu(NCMe)][OTf]

Report prepared for:

R. Zhang, A. Panda, Prof. W. Jones

November 06, 2023

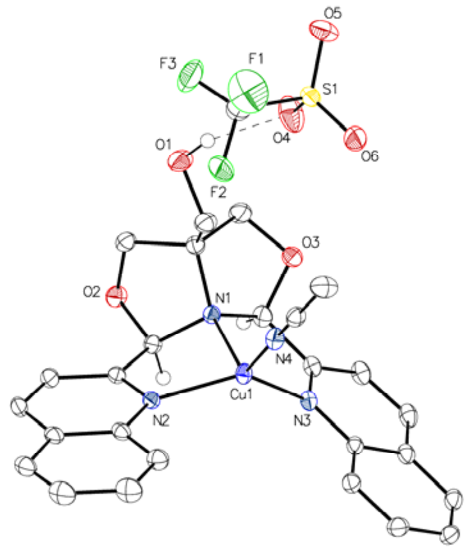

William W. Brennessel

X-ray Crystallographic Facility

Department of Chemistry, University of Rochester

120 Trustee Road

Rochester, NY 14627

Data collection

A crystal (0.212 x 0.069 x 0.044 mm^3) was placed onto a nylon loop and mounted on a Rigaku XtaLAB Synergy-S Dualflex diffractometer equipped with a HyPix-6000HE HPC area detector for data collection at 100.01(10) K. A preliminary set of cell constants and an orientation matrix were calculated from a small sampling of reflections.[1] A short pre-experiment was run, from which an optimal data collection strategy was determined. The full data collection was carried out using a PhotonJet (Cu) X-ray source with frame times of 0.57 and 2.30 seconds and a detector distance of 34.0 mm. Series of frames were collected in 0.50° steps in ω at different 2θ, κ, and ϕ settings. After the intensity data were corrected for absorption, the final cell constants were calculated from the xyz centroids of 18225 strong reflections from the actual data collection after integration.[1] See Table S-1 for additional crystal and refinement information.

Structure solution and refinement

The structure was solved using SHELXT[2] and refined using SHELXL.[3] The space group *I*2/*a* was determined based on systematic absences and intensity statistics. Most or all non-hydrogen atoms were assigned from the solution. Full-matrix least squares / difference Fourier cycles were performed which located any remaining non-hydrogen atoms. All non-hydrogen atoms were refined with anisotropic displacement parameters. The O-H hydrogen bond was found from the difference Fourier map and refined freely. All other hydrogen atoms were placed in ideal positions and refined as riding atoms with relative isotropic displacement parameters. The final full matrix least squares refinement converged to $R1 = 0.0306$ (F^2, $I > 2\sigma(I)$) and $wR2 = 0.0789$ (F^2, all data).

Structure description

The structure is the one suggested. The asymmetric unit contains one monocationic Cu complex and one triflate anion in general positions. The cation and anion are linked via hydrogen bonding (see figures and Table S-7).

Structure manipulation and figure generation were performed using Olex2.[4] Unless noted otherwise all structural diagrams containing anisotropic displacement ellipsoids are drawn at the 50 % probability level.

Data collection, structure solution, and structure refinement were conducted at the X-ray Crystallographic Facility, B04 Hutchison Hall, Department of Chemistry, University of Rochester. The instrument was purchased with funding from NSF MRI program grant CHE-1725028. All publications arising from this report MUST either 1) include William W. Brennessel as a coauthor or 2) acknowledge William W. Brennessel and the X-ray Crystallographic Facility of the Department of Chemistry at the University of Rochester.

[1] *CrysAlisPro*, version 171.42.101a; Rigaku Corporation: Oxford, UK, 2023.

[2] Sheldrick, G. M. *SHELXT*, version 2018/2; *Acta. Crystallogr.* **2015**, *A71*, 3-8.

[3] Sheldrick, G. M. *SHELXL*, version 2019/2; *Acta. Crystallogr.* **2015**, *C71*, 3-8.

[4] Dolomanov, O. V.; Bourhis, L. J.; Gildea, R. J.; Howard, J. A. K.; Puschmann, H. *Olex2*, version 1.5; *J. Appl. Cryst.* **2009**, *42*, 339-341.

Some equations of interest:

$$R_{int} = \Sigma |F_o^2 - \langle F_o^2 \rangle| / \Sigma |F_o^2|$$

$$R1 = \Sigma ||F_o| - |F_c|| / \Sigma |F_o|$$

$$wR2 = [\Sigma[w(F_o^2 - F_c^2)^2] / \Sigma [w(F_o^2)^2]]^{1/2}$$

where $w = 1 / [\sigma^2 (F_o^2) + (aP)^2 + bP]$ and

$$P = 1/3 \max (0, F_o^2) + 2/3 F_c^2$$

$$GOF = S = [\Sigma[w(F_o^2 - F_c^2)^2] / (m-n)]^{1/2}$$

where m = number of reflections and n = number of parameters

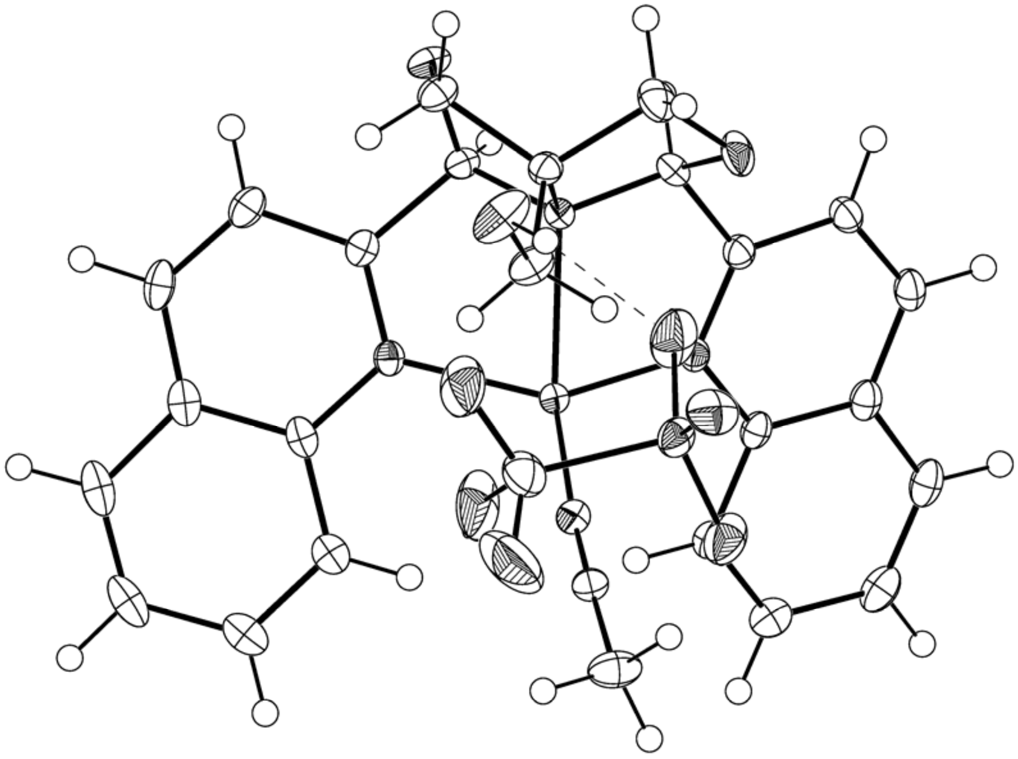

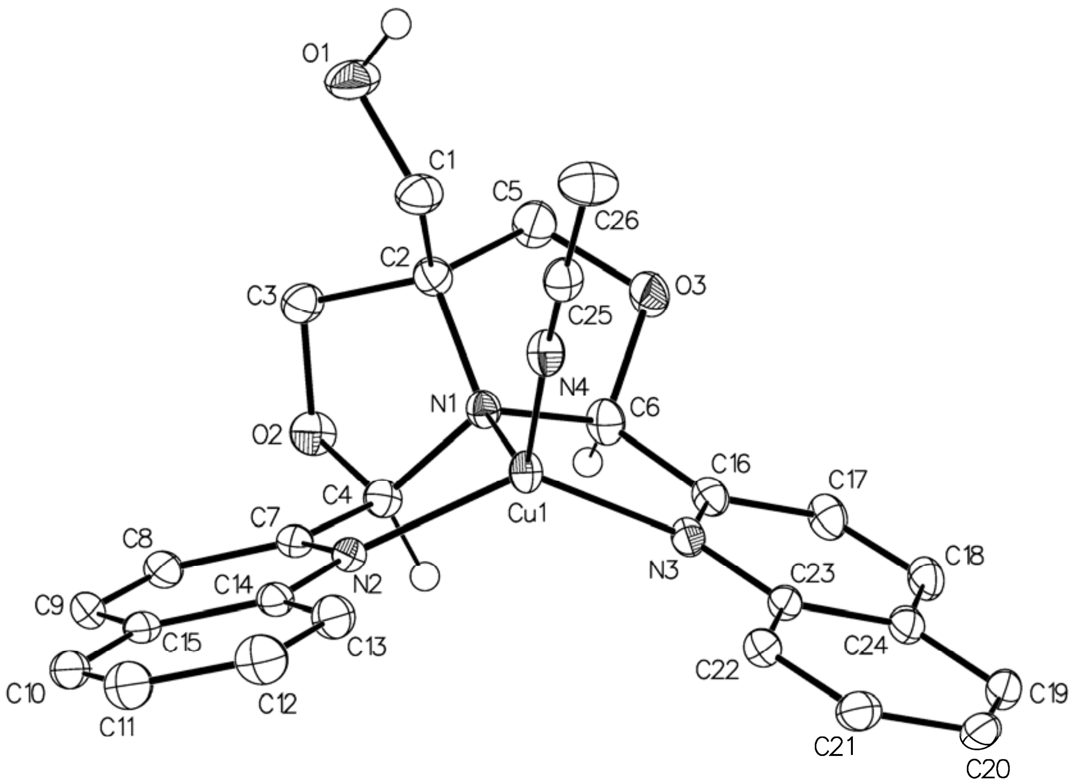
O1
C1
C5
C26
C2
C3
O3
C25
N4
N1
C6
O2
C16
C17
C4
Cu1
C7
C8
N3
C18
N2
C23
C14
C9
C15
C13
C24
C22
C10
C12
C19
C11
C21
C20

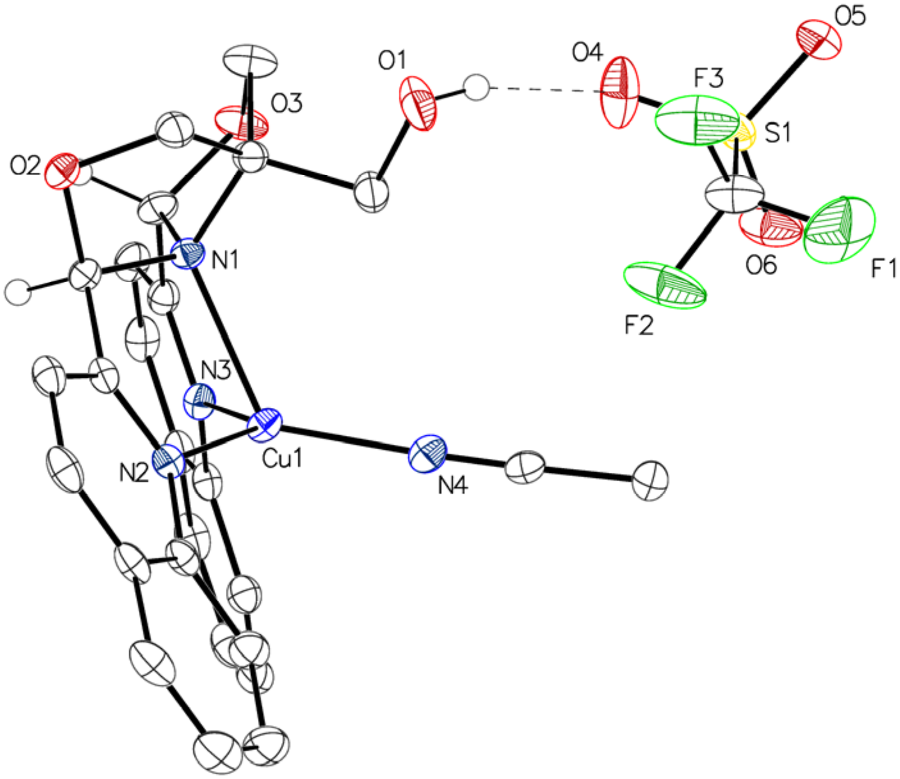
O5
O1
O4
F3
O3
S1
O2
N1
O6
F1
F2
N3
Cu1
N2
N4

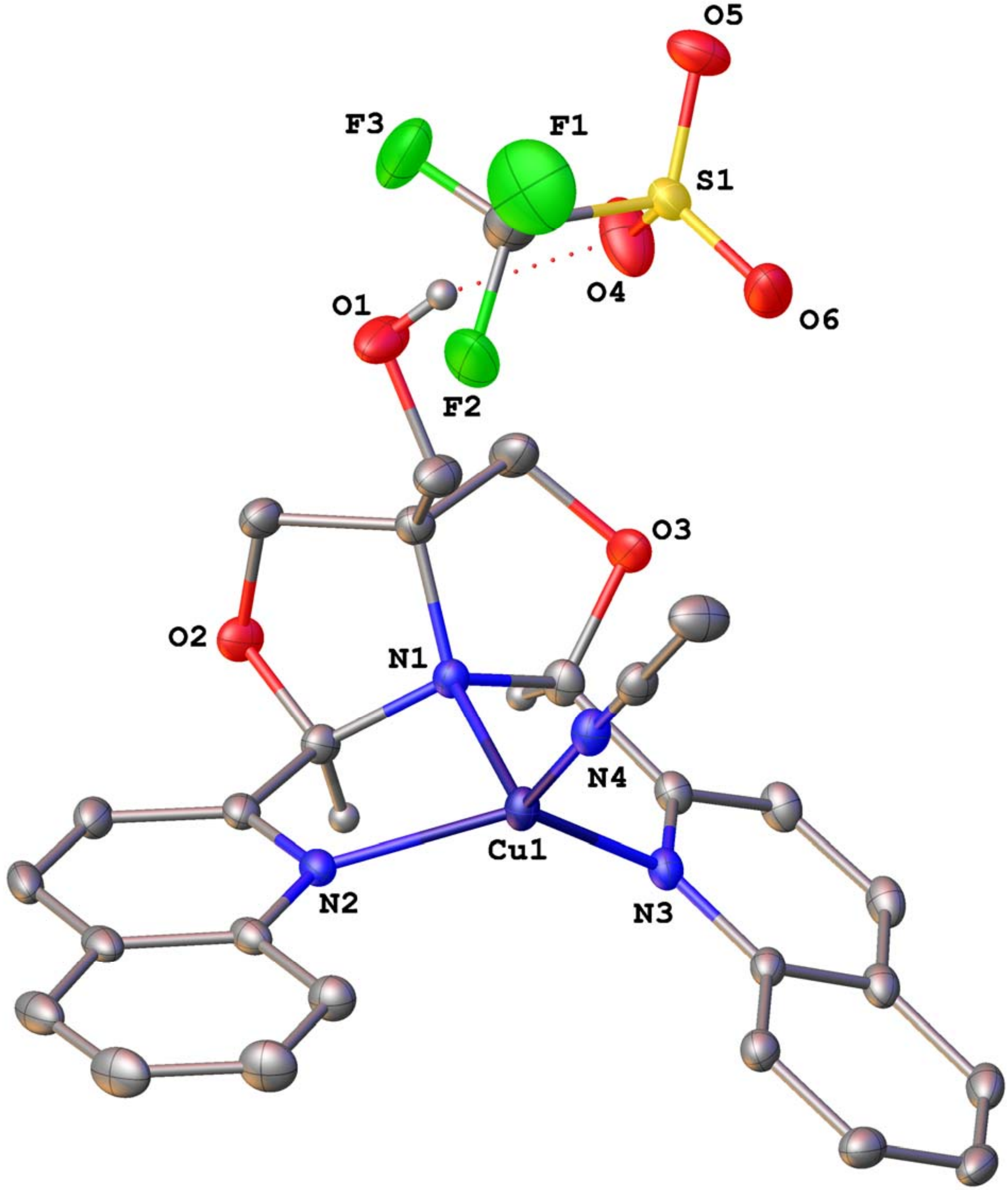
O5
F3
F1
S1
O1
O4
O6
F2
O3
O2
N1
N4
Cu1
N2
N3

Table S-1. Crystal data and structure refinement for jonap31.

Identification code	jonap31	
Empirical formula	C27 H24 Cu F3 N4 O6 S	
Formula weight	653.10	
Temperature	100.01(10) K	
Wavelength	1.54184 Å	
Crystal system	monoclinic	
Space group	*I*2/*a*	
Unit cell dimensions	*a* = 16.5237(2) Å	α = 90°
	b = 21.3215(2) Å	β = 114.840(2)°
	c = 17.0080(2) Å	γ = 90°
Volume	5437.73(13) $Å^3$	
Z	8	
Density (calculated)	1.596 Mg/m^3	
Absorption coefficient	2.485 mm^{-1}	
F(000)	2672	
Crystal color, morphology	yellow, needle	
Crystal size	0.212 x 0.069 x 0.044 mm^3	
Theta range for data collection	3.535 to 80.022°	
Index ranges	$-18 \le h \le 21$, $-26 \le k \le 27$, $-21 \le l \le 19$	
Reflections collected	32968	
Independent reflections	5817 [*R*(int) = 0.0304]	
Observed reflections	5263	
Completeness to theta = 74.504°	99.8%	
Absorption correction	Multi-scan	
Max. and min. transmission	1.00000 and 0.80210	
Refinement method	Full-matrix least-squares on F^2	
Data / restraints / parameters	5817 / 0 / 384	
Goodness-of-fit on F^2	1.039	
Final *R* indices [*I*>2sigma(*I*)]	*R*1 = 0.0306, *wR*2 = 0.0769	
R indices (all data)	*R*1 = 0.0346, *wR*2 = 0.0789	
Largest diff. peak and hole	0.379 and -0.372 e.$Å^{-3}$	

Table S-2. Atomic coordinates (x 10^4) and equivalent isotropic displacement parameters ($Å^2$x 10^3) for jonap31. U_{eq} is defined as one third of the trace of the orthogonalized U_{ij} tensor.

	x	y	z	U_{eq}
Cu1	4211(1)	3571(1)	2915(1)	15(1)
O1	6418(1)	2755(1)	2148(1)	34(1)
O2	4964(1)	1677(1)	3187(1)	21(1)
O3	6440(1)	3004(1)	4536(1)	24(1)
N1	5083(1)	2748(1)	3445(1)	14(1)
N2	3357(1)	2897(1)	2186(1)	15(1)
N3	4536(1)	3718(1)	4187(1)	15(1)
N4	4480(1)	4232(1)	2290(1)	19(1)
C1	5839(1)	3013(1)	2485(1)	22(1)
C2	5794(1)	2561(1)	3154(1)	17(1)
C3	5512(1)	1905(1)	2778(1)	20(1)
C4	4468(1)	2210(1)	3222(1)	16(1)
C5	6642(1)	2568(1)	3999(1)	26(1)
C6	5562(1)	2846(1)	4397(1)	18(1)
C7	3656(1)	2315(1)	2363(1)	15(1)
C8	3238(1)	1799(1)	1823(1)	20(1)
C9	2498(1)	1905(1)	1070(1)	22(1)
C10	1383(1)	2664(1)	89(1)	25(1)
C11	1077(1)	3267(1)	-76(1)	29(1)
C12	1516(1)	3752(1)	517(1)	27(1)
C13	2262(1)	3627(1)	1259(1)	21(1)
C14	2597(1)	3010(1)	1441(1)	17(1)
C15	2151(1)	2517(1)	856(1)	19(1)
C16	5195(1)	3375(1)	4743(1)	16(1)
C17	5592(1)	3483(1)	5646(1)	19(1)
C18	5301(1)	3979(1)	5968(1)	20(1)
C19	4255(1)	4877(1)	5684(1)	22(1)
C20	3574(1)	5230(1)	5107(1)	23(1)
C21	3192(1)	5075(1)	4218(1)	21(1)
C22	3508(1)	4575(1)	3920(1)	18(1)
C23	4218(1)	4208(1)	4503(1)	15(1)

C24	4593(1)	4354(1)	5401(1)	18(1)
C25	4680(1)	4628(1)	1958(1)	18(1)
C26	4962(1)	5135(1)	1556(1)	28(1)
S1	2452(1)	5633(1)	1893(1)	21(1)
F1	1702(1)	5476(1)	221(1)	67(1)
F2	857(1)	5922(1)	723(1)	56(1)
F3	1905(1)	6445(1)	619(1)	53(1)
O4	2397(1)	6136(1)	2433(1)	43(1)
O5	3298(1)	5591(1)	1853(1)	32(1)
O6	2079(1)	5048(1)	1994(1)	38(1)
C27	1694(1)	5884(1)	807(1)	32(1)

Table S-3. Bond lengths [Å] and angles [°] for jonap31.

Cu(1)-N(1)	2.2082(13)	C(9)-H(9)	0.9500
Cu(1)-N(2)	2.0320(13)	C(9)-C(15)	1.409(3)
Cu(1)-N(3)	2.0213(13)	C(10)-H(10)	0.9500
Cu(1)-N(4)	1.9257(14)	C(10)-C(11)	1.367(3)
O(1)-C(1)	1.419(2)	C(10)-C(15)	1.422(2)
O(1)-H(1)	0.77(3)	C(11)-H(11)	0.9500
O(2)-C(3)	1.438(2)	C(11)-C(12)	1.412(3)
O(2)-C(4)	1.4158(19)	C(12)-H(12)	0.9500
O(3)-C(5)	1.436(2)	C(12)-C(13)	1.370(2)
O(3)-C(6)	1.410(2)	C(13)-H(13)	0.9500
N(1)-C(2)	1.508(2)	C(13)-C(14)	1.411(2)
N(1)-C(4)	1.4725(19)	C(14)-C(15)	1.421(2)
N(1)-C(6)	1.4882(19)	C(16)-C(17)	1.412(2)
N(2)-C(7)	1.322(2)	C(17)-H(17)	0.9500
N(2)-C(14)	1.380(2)	C(17)-C(18)	1.367(2)
N(3)-C(16)	1.322(2)	C(18)-H(18)	0.9500
N(3)-C(23)	1.378(2)	C(18)-C(24)	1.411(2)
N(4)-C(25)	1.140(2)	C(19)-H(19)	0.9500
C(1)-H(1A)	0.9900	C(19)-C(20)	1.366(3)
C(1)-H(1B)	0.9900	C(19)-C(24)	1.418(2)
C(1)-C(2)	1.516(2)	C(20)-H(20)	0.9500
C(2)-C(3)	1.528(2)	C(20)-C(21)	1.411(2)
C(2)-C(5)	1.528(2)	C(21)-H(21)	0.9500
C(3)-H(3A)	0.9900	C(21)-C(22)	1.374(2)
C(3)-H(3B)	0.9900	C(22)-H(22)	0.9500
C(4)-H(4)	1.0000	C(22)-C(23)	1.412(2)
C(4)-C(7)	1.528(2)	C(23)-C(24)	1.420(2)
C(5)-H(5A)	0.9900	C(25)-C(26)	1.457(2)
C(5)-H(5B)	0.9900	C(26)-H(26A)	0.9800
C(6)-H(6)	1.0000	C(26)-H(26B)	0.9800
C(6)-C(16)	1.512(2)	C(26)-H(26C)	0.9800
C(7)-C(8)	1.415(2)	S(1)-O(4)	1.4401(15)
C(8)-H(8)	0.9500	S(1)-O(5)	1.4308(13)
C(8)-C(9)	1.367(2)	S(1)-O(6)	1.4342(14)

S(1)-C(27)	1.823(2)	C(1)-C(2)-C(5)	112.69(14)
F(1)-C(27)	1.327(3)	C(5)-C(2)-C(3)	113.64(14)
F(2)-C(27)	1.332(2)	O(2)-C(3)-C(2)	104.53(12)
F(3)-C(27)	1.323(2)	O(2)-C(3)-H(3A)	110.8
N(2)-Cu(1)-N(1)	81.58(5)	O(2)-C(3)-H(3B)	110.8
N(3)-Cu(1)-N(1)	81.46(5)	C(2)-C(3)-H(3A)	110.8
N(3)-Cu(1)-N(2)	124.51(5)	C(2)-C(3)-H(3B)	110.8
N(4)-Cu(1)-N(1)	122.94(5)	H(3A)-C(3)-H(3B)	108.9
N(4)-Cu(1)-N(2)	116.25(5)	O(2)-C(4)-N(1)	106.77(12)
N(4)-Cu(1)-N(3)	117.34(5)	O(2)-C(4)-H(4)	109.0
C(1)-O(1)-H(1)	105(2)	O(2)-C(4)-C(7)	111.65(13)
C(4)-O(2)-C(3)	103.97(11)	N(1)-C(4)-H(4)	109.0
C(6)-O(3)-C(5)	103.70(12)	N(1)-C(4)-C(7)	111.43(12)
C(2)-N(1)-Cu(1)	122.76(9)	C(7)-C(4)-H(4)	109.0
C(4)-N(1)-Cu(1)	104.66(9)	O(3)-C(5)-C(2)	103.78(13)
C(4)-N(1)-C(2)	104.91(12)	O(3)-C(5)-H(5A)	111.0
C(4)-N(1)-C(6)	112.56(12)	O(3)-C(5)-H(5B)	111.0
C(6)-N(1)-Cu(1)	106.86(9)	C(2)-C(5)-H(5A)	111.0
C(6)-N(1)-C(2)	105.23(12)	C(2)-C(5)-H(5B)	111.0
C(7)-N(2)-Cu(1)	115.26(10)	H(5A)-C(5)-H(5B)	109.0
C(7)-N(2)-C(14)	118.82(14)	O(3)-C(6)-N(1)	104.76(12)
C(14)-N(2)-Cu(1)	124.65(11)	O(3)-C(6)-H(6)	109.9
C(16)-N(3)-Cu(1)	116.70(11)	O(3)-C(6)-C(16)	108.02(13)
C(16)-N(3)-C(23)	118.30(13)	N(1)-C(6)-H(6)	109.9
C(23)-N(3)-Cu(1)	124.17(11)	N(1)-C(6)-C(16)	114.28(13)
C(25)-N(4)-Cu(1)	176.40(14)	C(16)-C(6)-H(6)	109.9
O(1)-C(1)-H(1A)	110.1	N(2)-C(7)-C(4)	116.75(13)
O(1)-C(1)-H(1B)	110.1	N(2)-C(7)-C(8)	123.16(15)
O(1)-C(1)-C(2)	108.10(14)	C(8)-C(7)-C(4)	120.01(14)
H(1A)-C(1)-H(1B)	108.4	C(7)-C(8)-H(8)	120.6
C(2)-C(1)-H(1A)	110.1	C(9)-C(8)-C(7)	118.74(16)
C(2)-C(1)-H(1B)	110.1	C(9)-C(8)-H(8)	120.6
N(1)-C(2)-C(1)	111.56(13)	C(8)-C(9)-H(9)	120.0
N(1)-C(2)-C(3)	103.64(12)	C(8)-C(9)-C(15)	120.09(15)
N(1)-C(2)-C(5)	102.78(12)	C(15)-C(9)-H(9)	120.0
C(1)-C(2)-C(3)	111.80(13)	C(11)-C(10)-H(10)	119.8

C(11)-C(10)-C(15)	120.44(16)	C(20)-C(21)-H(21)	119.7
C(15)-C(10)-H(10)	119.8	C(22)-C(21)-C(20)	120.63(16)
C(10)-C(11)-H(11)	119.8	C(22)-C(21)-H(21)	119.7
C(10)-C(11)-C(12)	120.48(16)	C(21)-C(22)-H(22)	119.9
C(12)-C(11)-H(11)	119.8	C(21)-C(22)-C(23)	120.13(15)
C(11)-C(12)-H(12)	119.7	C(23)-C(22)-H(22)	119.9
C(13)-C(12)-C(11)	120.60(18)	N(3)-C(23)-C(22)	119.10(14)
C(13)-C(12)-H(12)	119.7	N(3)-C(23)-C(24)	121.39(15)
C(12)-C(13)-H(13)	120.0	C(22)-C(23)-C(24)	119.51(15)
C(12)-C(13)-C(14)	120.07(16)	C(18)-C(24)-C(19)	123.06(15)
C(14)-C(13)-H(13)	120.0	C(18)-C(24)-C(23)	118.18(15)
N(2)-C(14)-C(13)	119.04(14)	C(19)-C(24)-C(23)	118.75(15)
N(2)-C(14)-C(15)	121.19(15)	N(4)-C(25)-C(26)	178.17(19)
C(13)-C(14)-C(15)	119.76(15)	C(25)-C(26)-H(26A)	109.5
C(9)-C(15)-C(10)	123.41(16)	C(25)-C(26)-H(26B)	109.5
C(9)-C(15)-C(14)	117.96(15)	C(25)-C(26)-H(26C)	109.5
C(14)-C(15)-C(10)	118.64(16)	H(26A)-C(26)-H(26B)	109.5
N(3)-C(16)-C(6)	118.53(13)	H(26A)-C(26)-H(26C)	109.5
N(3)-C(16)-C(17)	123.71(15)	H(26B)-C(26)-H(26C)	109.5
C(17)-C(16)-C(6)	117.75(14)	O(4)-S(1)-C(27)	103.15(10)
C(16)-C(17)-H(17)	120.6	O(5)-S(1)-O(4)	113.72(10)
C(18)-C(17)-C(16)	118.73(15)	O(5)-S(1)-O(6)	115.31(9)
C(18)-C(17)-H(17)	120.6	O(5)-S(1)-C(27)	103.85(9)
C(17)-C(18)-H(18)	120.2	O(6)-S(1)-O(4)	115.28(10)
C(17)-C(18)-C(24)	119.65(15)	O(6)-S(1)-C(27)	103.30(9)
C(24)-C(18)-H(18)	120.2	F(1)-C(27)-S(1)	110.43(15)
C(20)-C(19)-H(19)	119.6	F(1)-C(27)-F(2)	107.28(18)
C(20)-C(19)-C(24)	120.82(15)	F(2)-C(27)-S(1)	111.53(14)
C(24)-C(19)-H(19)	119.6	F(3)-C(27)-S(1)	111.80(13)
C(19)-C(20)-H(20)	119.9	F(3)-C(27)-F(1)	108.98(18)
C(19)-C(20)-C(21)	120.14(16)	F(3)-C(27)-F(2)	106.63(18)
C(21)-C(20)-H(20)	119.9		

Table S-4. Anisotropic displacement parameters ($Å^2$x 10^3) for jonap31. The anisotropic displacement factor exponent takes the form: $-2\pi^2[h^2 a^{*2}U_{11} + ... + 2 h k a^* b^* U_{12}]$

	U_{11}	U_{22}	U_{33}	U_{23}	U_{13}	U_{12}
Cu1	17(1)	13(1)	13(1)	0(1)	4(1)	0(1)
O1	41(1)	22(1)	55(1)	-6(1)	38(1)	-7(1)
O2	22(1)	13(1)	28(1)	3(1)	12(1)	2(1)
O3	13(1)	32(1)	22(1)	-8(1)	1(1)	2(1)
N1	14(1)	14(1)	14(1)	0(1)	4(1)	1(1)
N2	14(1)	16(1)	14(1)	-2(1)	6(1)	-2(1)
N3	16(1)	15(1)	13(1)	-1(1)	5(1)	-3(1)
N4	21(1)	16(1)	17(1)	-1(1)	6(1)	0(1)
C1	25(1)	18(1)	30(1)	1(1)	17(1)	-1(1)
C2	15(1)	16(1)	19(1)	0(1)	6(1)	1(1)
C3	20(1)	17(1)	26(1)	-1(1)	12(1)	2(1)
C4	16(1)	13(1)	18(1)	1(1)	8(1)	0(1)
C5	17(1)	32(1)	23(1)	-5(1)	3(1)	6(1)
C6	17(1)	19(1)	14(1)	1(1)	2(1)	2(1)
C7	16(1)	16(1)	17(1)	-2(1)	9(1)	-3(1)
C8	21(1)	17(1)	24(1)	-4(1)	13(1)	-5(1)
C9	20(1)	25(1)	22(1)	-10(1)	12(1)	-11(1)
C10	17(1)	43(1)	15(1)	-7(1)	7(1)	-7(1)
C11	20(1)	48(1)	14(1)	2(1)	3(1)	0(1)
C12	22(1)	34(1)	22(1)	5(1)	5(1)	4(1)
C13	19(1)	25(1)	18(1)	0(1)	7(1)	0(1)
C14	14(1)	24(1)	13(1)	-1(1)	6(1)	-3(1)
C15	16(1)	28(1)	16(1)	-5(1)	9(1)	-6(1)
C16	17(1)	16(1)	15(1)	0(1)	5(1)	-3(1)
C17	20(1)	20(1)	14(1)	2(1)	4(1)	-2(1)
C18	24(1)	22(1)	13(1)	-2(1)	7(1)	-7(1)
C19	26(1)	23(1)	21(1)	-8(1)	14(1)	-9(1)
C20	26(1)	20(1)	30(1)	-7(1)	19(1)	-4(1)
C21	21(1)	20(1)	26(1)	0(1)	13(1)	0(1)
C22	18(1)	17(1)	18(1)	-2(1)	9(1)	-2(1)
C23	18(1)	15(1)	16(1)	-3(1)	9(1)	-4(1)

C24	21(1)	18(1)	16(1)	-3(1)	9(1)	-6(1)
C25	18(1)	16(1)	20(1)	-1(1)	6(1)	3(1)
C26	27(1)	22(1)	39(1)	9(1)	18(1)	3(1)
S1	17(1)	19(1)	26(1)	2(1)	8(1)	3(1)
F1	67(1)	83(1)	31(1)	-17(1)	1(1)	-6(1)
F2	18(1)	63(1)	73(1)	36(1)	4(1)	2(1)
F3	44(1)	55(1)	46(1)	27(1)	7(1)	-14(1)
O4	57(1)	37(1)	35(1)	1(1)	19(1)	24(1)
O5	19(1)	33(1)	43(1)	-5(1)	13(1)	-1(1)
O6	23(1)	32(1)	53(1)	17(1)	8(1)	-1(1)
C27	24(1)	36(1)	30(1)	5(1)	4(1)	-7(1)

Table S-5. Hydrogen coordinates (x 10^4) and isotropic displacement parameters ($Å^2$ x 10^3) for jonap31.

	x	y	z	U(eq)
H1A	5236	3075	2014	27
H1B	6066	3425	2757	27
H3A	5169	1923	2141	24
H3B	6038	1631	2917	24
H4	4262	2149	3691	19
H5A	6771	2146	4267	31
H5B	7161	2712	3899	31
H6	5564	2448	4709	22
H8	3466	1387	1979	23
H9	2217	1566	691	26
H10	1081	2341	-312	30
H11	565	3361	-592	34
H12	1291	4168	399	33
H13	2555	3957	1651	26
H17	6052	3217	6022	23
H18	5574	4070	6570	24
H19	4505	4983	6281	26
H20	3357	5581	5305	27
H21	2713	5318	3822	26
H22	3247	4475	3320	21
H26A	4754	5051	935	41
H26B	5615	5163	1824	41
H26C	4709	5532	1638	41
H1	6766(19)	3015(13)	2205(17)	42(7)

Table S-6. Torsion angles [°] for jonap31.

Cu1-N1-C2-C1	1.37(17)	C1-C2-C3-O2	-143.51(13)
Cu1-N1-C2-C3	-119.07(11)	C1-C2-C5-O3	94.51(16)
Cu1-N1-C2-C5	122.36(12)	C2-N1-C4-O2	24.24(15)
Cu1-N1-C4-O2	154.67(9)	C2-N1-C4-C7	-97.92(14)
Cu1-N1-C4-C7	32.51(13)	C2-N1-C6-O3	26.05(15)
Cu1-N1-C6-O3	-105.91(11)	C2-N1-C6-C16	144.07(13)
Cu1-N1-C6-C16	12.11(15)	C3-O2-C4-N1	-39.81(15)
Cu1-N2-C7-C4	15.74(17)	C3-O2-C4-C7	82.21(15)
Cu1-N2-C7-C8	-167.39(12)	C3-C2-C5-O3	-137.00(15)
Cu1-N2-C14-C13	-15.0(2)	C4-O2-C3-C2	38.76(15)
Cu1-N2-C14-C15	164.70(11)	C4-N1-C2-C1	120.25(14)
Cu1-N3-C16-C6	-8.52(19)	C4-N1-C2-C3	-0.20(15)
Cu1-N3-C16-C17	170.10(12)	C4-N1-C2-C5	-118.76(13)
Cu1-N3-C23-C22	11.4(2)	C4-N1-C6-O3	139.74(13)
Cu1-N3-C23-C24	-168.07(11)	C4-N1-C6-C16	-102.24(15)
O1-C1-C2-N1	-171.05(13)	C4-C7-C8-C9	178.22(14)
O1-C1-C2-C3	-55.51(18)	C5-O3-C6-N1	-43.38(16)
O1-C1-C2-C5	73.93(18)	C5-O3-C6-C16	-165.58(13)
O2-C4-C7-N2	-153.81(13)	C5-C2-C3-O2	87.54(16)
O2-C4-C7-C8	29.21(19)	C6-O3-C5-C2	43.19(17)
O3-C6-C16-N3	112.55(15)	C6-N1-C2-C1	-120.81(14)
O3-C6-C16-C17	-66.15(18)	C6-N1-C2-C3	118.74(13)
N1-C2-C3-O2	-23.23(15)	C6-N1-C2-C5	0.18(16)
N1-C2-C5-O3	-25.71(17)	C6-N1-C4-O2	-89.65(14)
N1-C4-C7-N2	-34.52(18)	C6-N1-C4-C7	148.19(13)
N1-C4-C7-C8	148.50(14)	C6-C16-C17-C18	176.73(15)
N1-C6-C16-N3	-3.6(2)	C7-N2-C14-C13	178.52(14)
N1-C6-C16-C17	177.71(14)	C7-N2-C14-C15	-1.8(2)
N2-C7-C8-C9	1.4(2)	C7-C8-C9-C15	-1.7(2)
N2-C14-C15-C9	1.4(2)	C8-C9-C15-C10	-179.69(15)
N2-C14-C15-C10	-178.50(14)	C8-C9-C15-C14	0.4(2)
N3-C16-C17-C18	-1.9(2)	C10-C11-C12-C13	0.9(3)
N3-C23-C24-C18	-0.6(2)	C11-C10-C15-C9	179.36(16)
N3-C23-C24-C19	178.03(14)	C11-C10-C15-C14	-0.7(2)

C11-C12-C13-C14	-0.4(3)	C21-C22-C23-N3	-178.55(14)
C12-C13-C14-N2	179.07(15)	C21-C22-C23-C24	1.0(2)
C12-C13-C14-C15	-0.6(2)	C22-C23-C24-C18	179.92(14)
C13-C14-C15-C9	-178.87(15)	C22-C23-C24-C19	-1.5(2)
C13-C14-C15-C10	1.2(2)	C23-N3-C16-C6	-178.57(13)
C14-N2-C7-C4	-176.53(13)	C23-N3-C16-C17	0.0(2)
C14-N2-C7-C8	0.3(2)	C24-C19-C20-C21	0.5(2)
C15-C10-C11-C12	-0.3(3)	O4-S1-C27-F1	177.08(15)
C16-N3-C23-C22	-179.30(14)	O4-S1-C27-F2	-63.73(17)
C16-N3-C23-C24	1.2(2)	O4-S1-C27-F3	55.56(18)
C16-C17-C18-C24	2.5(2)	O5-S1-C27-F1	58.19(16)
C17-C18-C24-C19	-179.83(15)	O5-S1-C27-F2	177.39(15)
C17-C18-C24-C23	-1.3(2)	O5-S1-C27-F3	-63.33(17)
C19-C20-C21-C22	-1.0(3)	O6-S1-C27-F1	-62.52(17)
C20-C19-C24-C18	179.28(16)	O6-S1-C27-F2	56.67(18)
C20-C19-C24-C23	0.8(2)	O6-S1-C27-F3	175.95(16)
C20-C21-C22-C23	0.3(2)		

Table S-7. Hydrogen bonds and close contacts for jonap31 [Å and °].

D-H...A	d(D-H)	d(H...A)	d(D...A)	<(DHA)
O1-H1...O4#1	0.77(3)	2.04(3)	2.788(2)	162(3)

Symmetry transformations used to generate equivalent atoms:

#1 x+1/2,-y+1,z

REFERENCE NUMBER: jonap37

1d’

CRYSTAL STRUCTURE REPORT

C_{26} H_{24} Br_2 N_4 Ni O_3

or

[(κ^4-L^q)Ni(Br)(NCMe)][Br]

Report prepared for:

R. Zhang, A. Panda, Prof. W. Jones

February 19, 2024

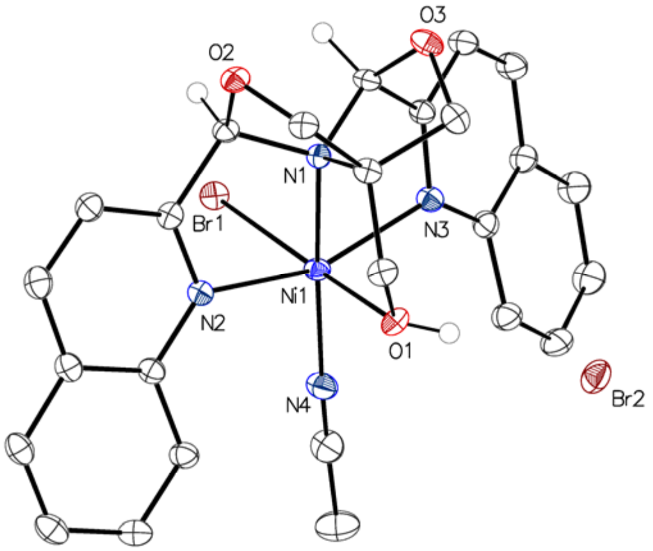

William W. Brennessel

X-ray Crystallographic Facility

Department of Chemistry, University of Rochester

120 Trustee Road

Rochester, NY 14627

Compound Isolation

During the recrystallization of compound **1d**, a small quantity of crystals formed that appeared distinct from the major crystallization product (**1d**). One of these minor crystals was examined by single-crystal X-ray structural determination, and found to be an acetonitrile solvate of **1d**, denoted as **1d'**.

Data collection

A crystal (0.188 x 0.145 x 0.109 mm^3) was placed onto a nylon loop and mounted on a Rigaku XtaLAB Synergy-S Dualflex diffractometer equipped with a HyPix-6000HE HPC area detector for data collection at 100.00(10) K. A preliminary set of cell constants and an orientation matrix were calculated from a small sampling of reflections.[1] A short pre-experiment was run, from which an optimal data collection strategy was determined. The full data collection was carried out using a PhotonJet (Cu) X-ray source with a frame time of 0.05 seconds and a detector distance of 34.0 mm. Series of frames were collected in 0.50º steps in ω at different 2θ, κ, and ϕ settings. After the intensity data were corrected for absorption, the final cell constants were calculated from the xyz centroids of 29005 strong reflections from the actual data collection after integration.[1] See Table S-1 for additional crystal and refinement information.

Structure solution and refinement

The structure was solved using SHELXT[2] and refined using SHELXL.[3] The space group *P*-1 was determined based on intensity statistics. Most or all non-hydrogen atoms were assigned from the solution. Full-matrix least squares / difference Fourier cycles were performed which located any remaining non-hydrogen atoms. All non-hydrogen atoms were refined with anisotropic displacement parameters. The O-H hydrogen atom was found from the difference Fourier map and refined freely. All other hydrogen atoms were placed in ideal positions and refined as riding atoms with relative isotropic displacement parameters. The final full matrix least squares refinement converged to $R1 = 0.0304$ (F^2, $I > 2\sigma(I)$) and $wR2 = 0.0730$ (F^2, all data).

Structure description

The structure is the one suggested. The asymmetric unit contains one monocationic nickel complex and one bromide anion in general positions. The two are linked via hydrogen bonding (see figure and Table S-7).

Structure manipulation and figure generation were performed using Olex2.[4] Unless noted otherwise all structural diagrams containing anisotropic displacement ellipsoids are drawn at the 50 % probability level.

Data collection, structure solution, and structure refinement were conducted at the X-ray Crystallographic Facility, B04 Hutchison Hall, Department of Chemistry, University of Rochester. The instrument was purchased with funding from NSF MRI program grant CHE-1725028. All publications arising from this report MUST either 1) include William W. Brennessel as a coauthor or 2) acknowledge William W. Brennessel and the X-ray Crystallographic Facility of the Department of Chemistry at the University of Rochester.

[1] *CrysAlisPro*, version 171.43.104a; Rigaku Corporation: Oxford, UK, 2024.

[2] Sheldrick, G. M. *SHELXT*, version 2018/2; *Acta. Crystallogr.* **2015**, *A71*, 3-8.

[3] Sheldrick, G. M. *SHELXL*, version 2019/2; *Acta. Crystallogr.* **2015**, *C71*, 3-8.

[4] Dolomanov, O. V.; Bourhis, L. J.; Gildea, R. J.; Howard, J. A. K.; Puschmann, H. *Olex2*, version 1.5; *J. Appl. Cryst.* **2009**, *42*, 339-341.

Some equations of interest:

$$R_{\text{int}} = \Sigma\, |F_o^2 - \langle F_o^2 \rangle| \,/\, \Sigma |F_o^2|$$

$$R1 = \Sigma ||F_o| - |F_c|| \,/\, \Sigma |F_o|$$

$$wR2 = [\Sigma[w(F_o^2 - F_c^2)^2] \,/\, \Sigma\, [w(F_o^2)^2]]^{1/2}$$

where $w = 1 / [\sigma^2 (F_o^2) + (aP)^2 + bP]$ and

$$P = 1/3 \max(0, F_o^2) + 2/3\, F_c^2$$

$$\text{GOF} = S = [\Sigma[w(F_o^2 - F_c^2)^2] \,/\, (m-n)]^{1/2}$$

where m = number of reflections and n = number of parameters

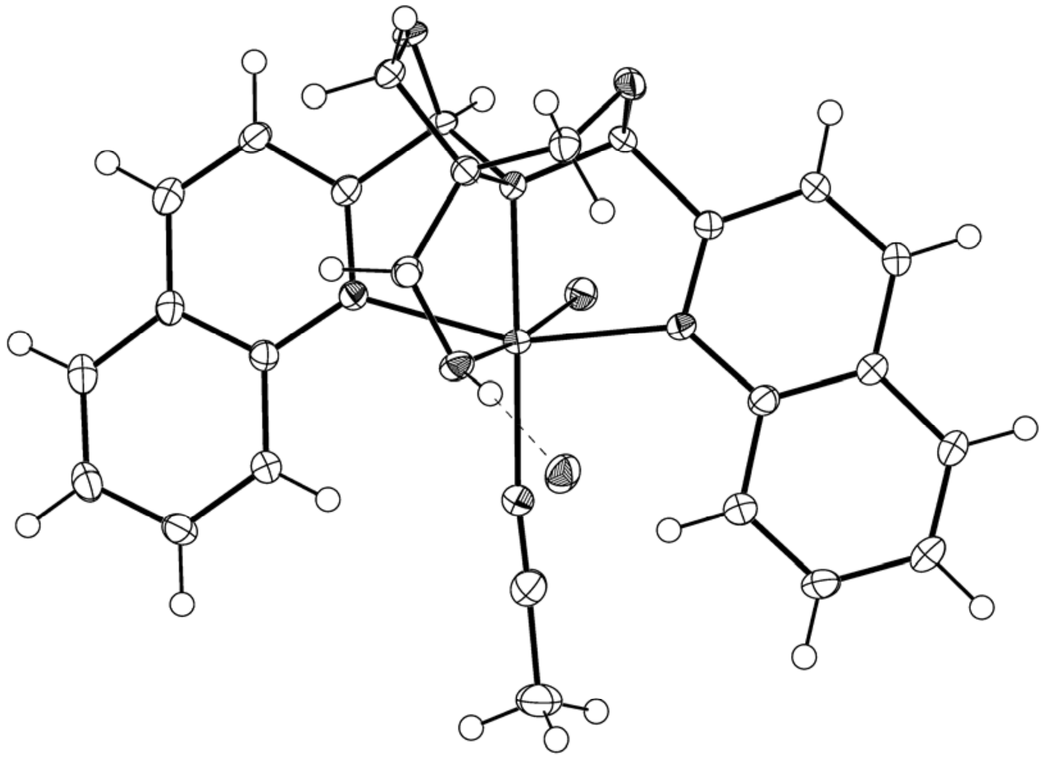

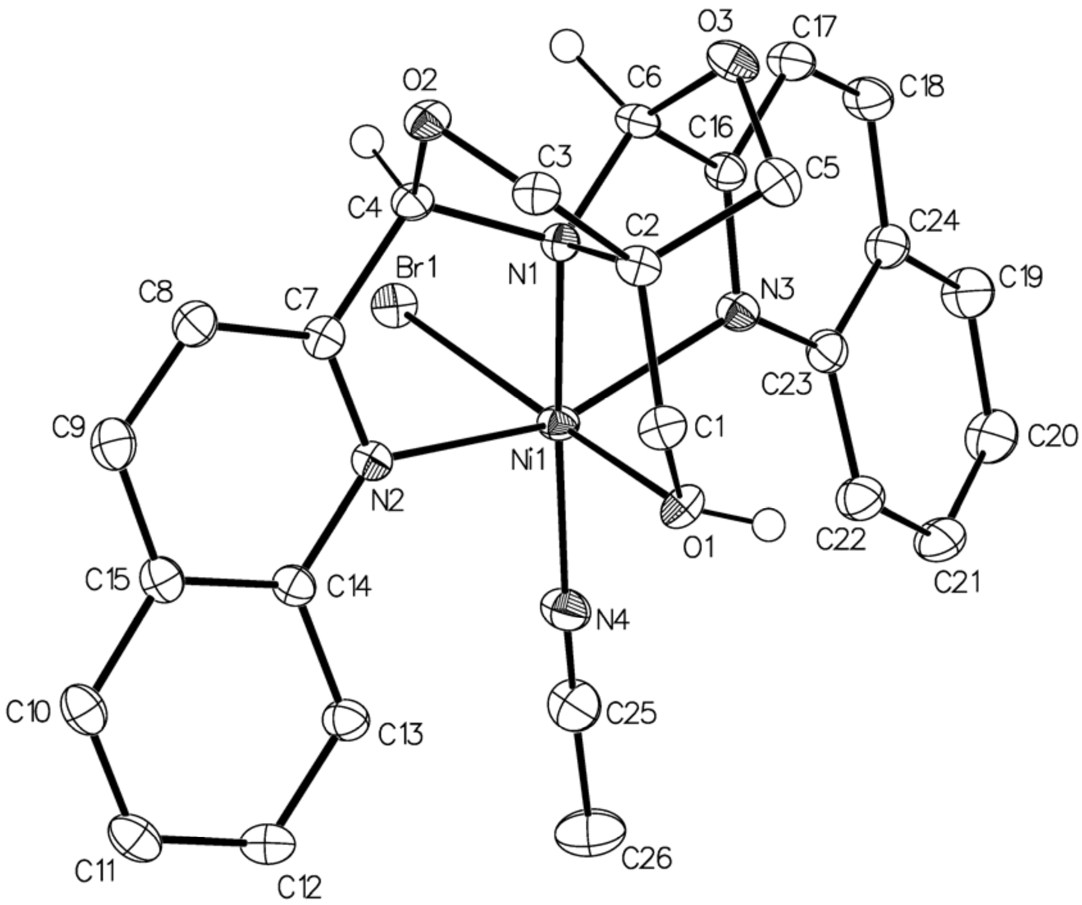
O3
C17
C6
C18
O2
C16
C3
C5
C4
C2
C24
N1
Br1
C8
C7
N3
C19
C23
C9
C1
C20
Ni1
N2
O1
C22
C21
C15
C14
N4
C25
C10
C13
C26
C11
C12

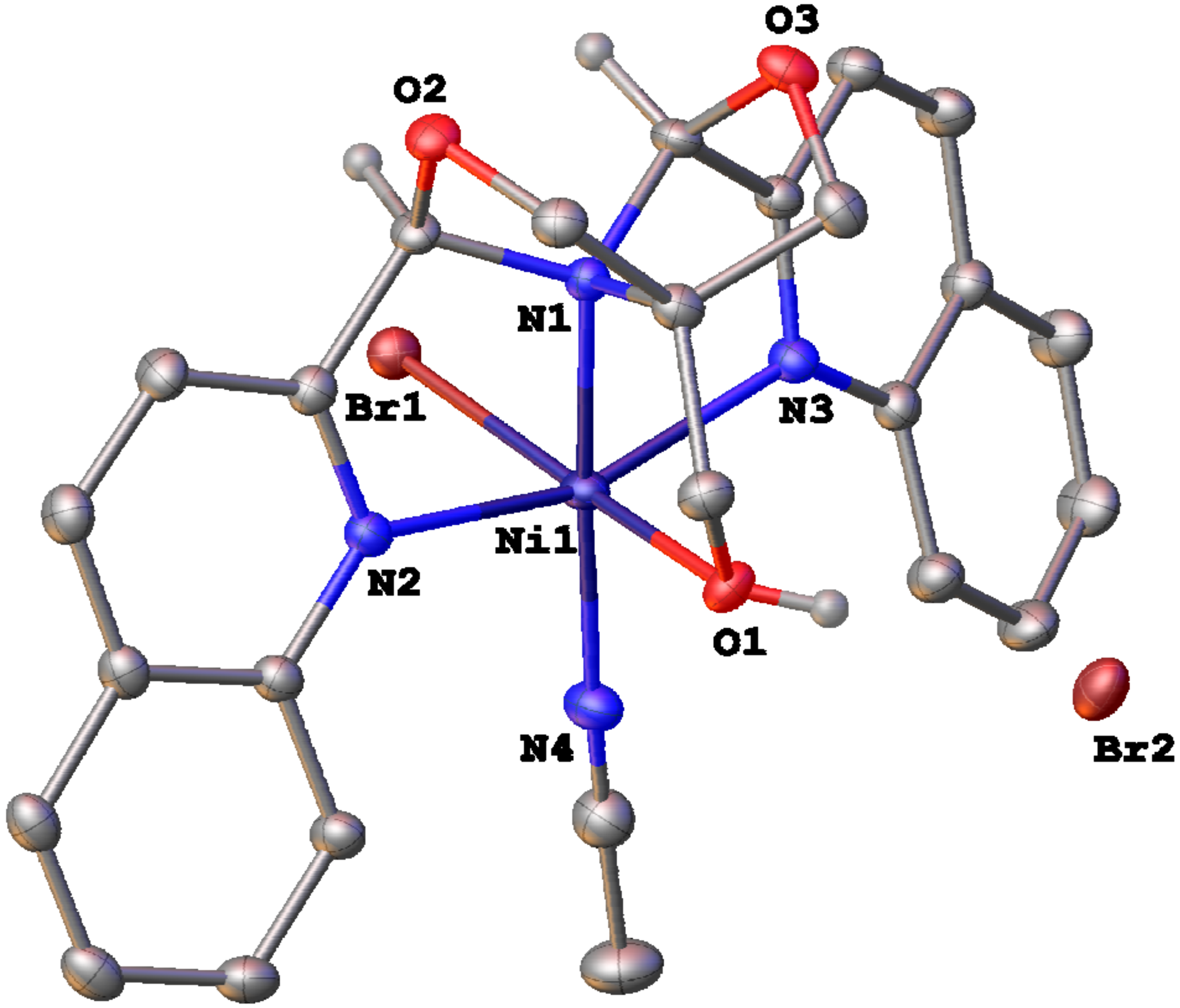

O3
O2
N1
Br1
N3
Ni1
N2
O1
N4
Br2

Table S-1. Crystal data and structure refinement for jonap37.

Identification code	jonap37	
Empirical formula	C26 H24 Br2 N4 Ni O3	
Formula weight	659.02	
Temperature	100.00(10) K	
Wavelength	1.54184 Å	
Crystal system	triclinic	
Space group	*P*-1	
Unit cell dimensions	*a* = 8.68250(10) Å	α = 76.6930(10)°
	b = 11.55700(10) Å	β = 83.5460(10)°
	c = 13.90270(10) Å	γ = 73.5020(10)°
Volume	1300.04(2) $Å^3$	
Z	2	
Density (calculated)	1.684 Mg/m^3	
Absorption coefficient	4.972 mm^{-1}	
F(000)	660	
Crystal color, morphology	green, block	
Crystal size	0.188 x 0.145 x 0.109 mm^3	
Theta range for data collection	3.271 to 80.200°	
Index ranges	$-11 \leq h \leq 10$, $-14 \leq k \leq 14$, $-17 \leq l \leq 17$	
Reflections collected	44014	
Independent reflections	5573 [*R*(int) = 0.0389]	
Observed reflections	5476	
Completeness to theta = 74.504°	99.7%	
Absorption correction	Multi-scan	
Max. and min. transmission	1.00000 and 0.82425	
Refinement method	Full-matrix least-squares on F^2	
Data / restraints / parameters	5573 / 0 / 330	
Goodness-of-fit on F^2	1.094	
Final *R* indices [*I*>2sigma(*I*)]	*R*1 = 0.0304, *wR*2 = 0.0726	
R indices (all data)	*R*1 = 0.0309, *wR*2 = 0.0730	
Largest diff. peak and hole	0.903 and -0.557 e.$Å^{-3}$	

Table S-2. Atomic coordinates (x 10^4) and equivalent isotropic displacement parameters ($Å^2$x 10^3) for jonap37. U_{eq} is defined as one third of the trace of the orthogonalized U_{ij} tensor.

	x	y	z	U_{eq}
Br1	3467(1)	6126(1)	8175(1)	17(1)
Ni1	5596(1)	7234(1)	7454(1)	13(1)
O1	7507(2)	7971(2)	6893(1)	17(1)
O2	9075(2)	4053(2)	8481(1)	16(1)
O3	8378(2)	6069(2)	9777(1)	18(1)
N1	7308(2)	5983(2)	8339(1)	12(1)
N2	6633(2)	5857(2)	6542(1)	14(1)
N3	5131(2)	8084(2)	8734(1)	15(1)
N4	4013(3)	8544(2)	6538(2)	20(1)
C1	9030(3)	7081(2)	7086(2)	17(1)
C2	8960(3)	6197(2)	8095(2)	14(1)
C3	10068(3)	4877(2)	8110(2)	16(1)
C4	7513(3)	4728(2)	8186(2)	14(1)
C5	9169(3)	6724(2)	8966(2)	18(1)
C6	6934(3)	6059(2)	9399(2)	14(1)
C7	7339(3)	4779(2)	7100(2)	15(1)
C8	7945(3)	3672(2)	6748(2)	18(1)
C9	7799(3)	3699(2)	5777(2)	20(1)
C10	6929(3)	4927(2)	4122(2)	19(1)
C11	6261(3)	6037(2)	3532(2)	21(1)
C12	5737(3)	7115(2)	3929(2)	21(1)
C13	5885(3)	7050(2)	4916(2)	18(1)
C14	6546(3)	5908(2)	5544(2)	15(1)
C15	7108(3)	4826(2)	5142(2)	17(1)
C16	5652(3)	7227(2)	9521(2)	14(1)
C17	5116(3)	7317(2)	10506(2)	17(1)
C18	3932(3)	8324(2)	10670(2)	20(1)
C19	2131(3)	10361(2)	9977(2)	23(1)
C20	1654(3)	11303(2)	9183(2)	25(1)
C21	2420(3)	11217(2)	8243(2)	24(1)
C22	3593(3)	10178(2)	8099(2)	20(1)

C23	4032(3)	9163(2)	8896(2)	16(1)
C24	3348(3)	9280(2)	9857(2)	17(1)
C25	3034(3)	9296(2)	6105(2)	23(1)
C26	1865(4)	10289(3)	5522(2)	31(1)
Br2	8060(1)	10427(1)	7050(1)	24(1)

Table S-3. Bond lengths [Å] and angles [°] for jonap37.

Br(1)-Ni(1)	2.5304(5)	C(8)-H(8)	0.9500
Ni(1)-O(1)	2.0712(17)	C(8)-C(9)	1.363(3)
Ni(1)-N(1)	2.0456(19)	C(9)-H(9)	0.9500
Ni(1)-N(2)	2.1932(19)	C(9)-C(15)	1.408(3)
Ni(1)-N(3)	2.170(2)	C(10)-H(10)	0.9500
Ni(1)-N(4)	2.032(2)	C(10)-C(11)	1.362(4)
O(1)-C(1)	1.435(3)	C(10)-C(15)	1.418(3)
O(1)-H(1)	0.82(4)	C(11)-H(11)	0.9500
O(2)-C(3)	1.434(3)	C(11)-C(12)	1.414(4)
O(2)-C(4)	1.412(3)	C(12)-H(12)	0.9500
O(3)-C(5)	1.431(3)	C(12)-C(13)	1.376(3)
O(3)-C(6)	1.416(3)	C(13)-H(13)	0.9500
N(1)-C(2)	1.513(3)	C(13)-C(14)	1.409(3)
N(1)-C(4)	1.472(3)	C(14)-C(15)	1.424(3)
N(1)-C(6)	1.490(3)	C(16)-C(17)	1.413(3)
N(2)-C(7)	1.325(3)	C(17)-H(17)	0.9500
N(2)-C(14)	1.385(3)	C(17)-C(18)	1.362(3)
N(3)-C(16)	1.320(3)	C(18)-H(18)	0.9500
N(3)-C(23)	1.386(3)	C(18)-C(24)	1.411(3)
N(4)-C(25)	1.138(3)	C(19)-H(19)	0.9500
C(1)-H(1A)	0.9900	C(19)-C(20)	1.366(4)
C(1)-H(1B)	0.9900	C(19)-C(24)	1.418(3)
C(1)-C(2)	1.539(3)	C(20)-H(20)	0.9500
C(2)-C(3)	1.553(3)	C(20)-C(21)	1.409(4)
C(2)-C(5)	1.523(3)	C(21)-H(21)	0.9500
C(3)-H(3A)	0.9900	C(21)-C(22)	1.375(3)
C(3)-H(3B)	0.9900	C(22)-H(22)	0.9500
C(4)-H(4)	1.0000	C(22)-C(23)	1.412(3)
C(4)-C(7)	1.521(3)	C(23)-C(24)	1.419(3)
C(5)-H(5A)	0.9900	C(25)-C(26)	1.449(4)
C(5)-H(5B)	0.9900	C(26)-H(26A)	0.9800
C(6)-H(6)	1.0000	C(26)-H(26B)	0.9800
C(6)-C(16)	1.517(3)	C(26)-H(26C)	0.9800
C(7)-C(8)	1.414(3)	O(1)-Ni(1)-Br(1)	174.28(5)

O(1)-Ni(1)-N(2)	86.01(7)	C(2)-C(1)-H(1A)	109.4
O(1)-Ni(1)-N(3)	94.04(7)	C(2)-C(1)-H(1B)	109.4
N(1)-Ni(1)-Br(1)	92.52(5)	N(1)-C(2)-C(1)	111.37(18)
N(1)-Ni(1)-O(1)	82.53(7)	N(1)-C(2)-C(3)	102.97(18)
N(1)-Ni(1)-N(2)	78.13(7)	N(1)-C(2)-C(5)	101.76(18)
N(1)-Ni(1)-N(3)	81.27(7)	C(1)-C(2)-C(3)	112.91(19)
N(2)-Ni(1)-Br(1)	90.16(5)	C(5)-C(2)-C(1)	113.5(2)
N(3)-Ni(1)-Br(1)	88.03(5)	C(5)-C(2)-C(3)	113.21(19)
N(3)-Ni(1)-N(2)	159.22(7)	O(2)-C(3)-C(2)	106.05(17)
N(4)-Ni(1)-Br(1)	91.48(6)	O(2)-C(3)-H(3A)	110.5
N(4)-Ni(1)-O(1)	93.48(8)	O(2)-C(3)-H(3B)	110.5
N(4)-Ni(1)-N(1)	175.99(9)	C(2)-C(3)-H(3A)	110.5
N(4)-Ni(1)-N(2)	102.07(8)	C(2)-C(3)-H(3B)	110.5
N(4)-Ni(1)-N(3)	98.68(8)	H(3A)-C(3)-H(3B)	108.7
Ni(1)-O(1)-H(1)	125(3)	O(2)-C(4)-N(1)	105.76(18)
C(1)-O(1)-Ni(1)	112.02(13)	O(2)-C(4)-H(4)	110.1
C(1)-O(1)-H(1)	107(3)	O(2)-C(4)-C(7)	111.14(18)
C(4)-O(2)-C(3)	105.33(17)	N(1)-C(4)-H(4)	110.1
C(6)-O(3)-C(5)	104.82(17)	N(1)-C(4)-C(7)	109.51(18)
C(2)-N(1)-Ni(1)	112.71(13)	C(7)-C(4)-H(4)	110.1
C(4)-N(1)-Ni(1)	110.72(13)	O(3)-C(5)-C(2)	103.27(19)
C(4)-N(1)-C(2)	104.31(17)	O(3)-C(5)-H(5A)	111.1
C(4)-N(1)-C(6)	111.93(17)	O(3)-C(5)-H(5B)	111.1
C(6)-N(1)-Ni(1)	111.35(13)	C(2)-C(5)-H(5A)	111.1
C(6)-N(1)-C(2)	105.54(17)	C(2)-C(5)-H(5B)	111.1
C(7)-N(2)-Ni(1)	110.70(15)	H(5A)-C(5)-H(5B)	109.1
C(7)-N(2)-C(14)	117.6(2)	O(3)-C(6)-N(1)	106.59(18)
C(14)-N(2)-Ni(1)	131.39(15)	O(3)-C(6)-H(6)	109.8
C(16)-N(3)-Ni(1)	108.60(15)	O(3)-C(6)-C(16)	108.79(18)
C(16)-N(3)-C(23)	117.1(2)	N(1)-C(6)-H(6)	109.8
C(23)-N(3)-Ni(1)	130.76(15)	N(1)-C(6)-C(16)	112.03(18)
C(25)-N(4)-Ni(1)	173.4(2)	C(16)-C(6)-H(6)	109.8
O(1)-C(1)-H(1A)	109.4	N(2)-C(7)-C(4)	118.0(2)
O(1)-C(1)-H(1B)	109.4	N(2)-C(7)-C(8)	124.2(2)
O(1)-C(1)-C(2)	111.01(18)	C(8)-C(7)-C(4)	117.8(2)
H(1A)-C(1)-H(1B)	108.0	C(7)-C(8)-H(8)	120.7

C(9)-C(8)-C(7)	118.7(2)	C(17)-C(18)-H(18)	120.5
C(9)-C(8)-H(8)	120.7	C(17)-C(18)-C(24)	119.0(2)
C(8)-C(9)-H(9)	120.2	C(24)-C(18)-H(18)	120.5
C(8)-C(9)-C(15)	119.6(2)	C(20)-C(19)-H(19)	119.7
C(15)-C(9)-H(9)	120.2	C(20)-C(19)-C(24)	120.7(2)
C(11)-C(10)-H(10)	119.5	C(24)-C(19)-H(19)	119.7
C(11)-C(10)-C(15)	120.9(2)	C(19)-C(20)-H(20)	120.2
C(15)-C(10)-H(10)	119.5	C(19)-C(20)-C(21)	119.6(2)
C(10)-C(11)-H(11)	119.9	C(21)-C(20)-H(20)	120.2
C(10)-C(11)-C(12)	120.3(2)	C(20)-C(21)-H(21)	119.4
C(12)-C(11)-H(11)	119.9	C(22)-C(21)-C(20)	121.2(2)
C(11)-C(12)-H(12)	119.8	C(22)-C(21)-H(21)	119.4
C(13)-C(12)-C(11)	120.3(2)	C(21)-C(22)-H(22)	120.0
C(13)-C(12)-H(12)	119.8	C(21)-C(22)-C(23)	120.0(2)
C(12)-C(13)-H(13)	119.8	C(23)-C(22)-H(22)	120.0
C(12)-C(13)-C(14)	120.4(2)	N(3)-C(23)-C(22)	119.8(2)
C(14)-C(13)-H(13)	119.8	N(3)-C(23)-C(24)	121.3(2)
N(2)-C(14)-C(13)	119.5(2)	C(22)-C(23)-C(24)	118.8(2)
N(2)-C(14)-C(15)	121.2(2)	C(18)-C(24)-C(19)	121.9(2)
C(13)-C(14)-C(15)	119.3(2)	C(18)-C(24)-C(23)	118.7(2)
C(9)-C(15)-C(10)	122.8(2)	C(19)-C(24)-C(23)	119.4(2)
C(9)-C(15)-C(14)	118.5(2)	N(4)-C(25)-C(26)	176.4(3)
C(10)-C(15)-C(14)	118.7(2)	C(25)-C(26)-H(26A)	109.5
N(3)-C(16)-C(6)	119.8(2)	C(25)-C(26)-H(26B)	109.5
N(3)-C(16)-C(17)	124.5(2)	C(25)-C(26)-H(26C)	109.5
C(17)-C(16)-C(6)	115.64(19)	H(26A)-C(26)-H(26B)	109.5
C(16)-C(17)-H(17)	120.6	H(26A)-C(26)-H(26C)	109.5
C(18)-C(17)-C(16)	118.9(2)	H(26B)-C(26)-H(26C)	109.5
C(18)-C(17)-H(17)	120.6		

Table S-4. Anisotropic displacement parameters ($Å^2$x 10^3) for jonap37. The anisotropic displacement factor exponent takes the form: $-2\pi^2[h^2 a^{*2}U_{11} + ... + 2 h k a^* b^* U_{12}]$

	U_{11}	U_{22}	U_{33}	U_{23}	U_{13}	U_{12}
Br1	15(1)	18(1)	18(1)	-3(1)	0(1)	-4(1)
Ni1	13(1)	12(1)	12(1)	-1(1)	-2(1)	0(1)
O1	17(1)	12(1)	20(1)	-1(1)	0(1)	-3(1)
O2	13(1)	13(1)	19(1)	-1(1)	-4(1)	1(1)
O3	13(1)	25(1)	14(1)	-4(1)	-2(1)	-2(1)
N1	12(1)	12(1)	12(1)	-3(1)	0(1)	-2(1)
N2	13(1)	14(1)	14(1)	-3(1)	-1(1)	-3(1)
N3	14(1)	14(1)	15(1)	-3(1)	-2(1)	0(1)
N4	22(1)	19(1)	16(1)	-4(1)	-3(1)	0(1)
C1	15(1)	17(1)	17(1)	-3(1)	1(1)	-2(1)
C2	11(1)	17(1)	14(1)	-3(1)	0(1)	-2(1)
C3	12(1)	16(1)	17(1)	-3(1)	0(1)	0(1)
C4	13(1)	12(1)	15(1)	-2(1)	-3(1)	1(1)
C5	14(1)	22(1)	19(1)	-7(1)	-1(1)	-4(1)
C6	15(1)	16(1)	10(1)	-2(1)	-2(1)	-1(1)
C7	12(1)	14(1)	17(1)	-4(1)	0(1)	-2(1)
C8	18(1)	14(1)	20(1)	-5(1)	-2(1)	0(1)
C9	19(1)	19(1)	22(1)	-9(1)	1(1)	-2(1)
C10	17(1)	25(1)	18(1)	-9(1)	2(1)	-6(1)
C11	18(1)	30(1)	15(1)	-7(1)	1(1)	-6(1)
C12	21(1)	22(1)	16(1)	0(1)	-1(1)	-3(1)
C13	20(1)	17(1)	15(1)	-3(1)	0(1)	-3(1)
C14	14(1)	17(1)	14(1)	-4(1)	0(1)	-3(1)
C15	15(1)	19(1)	16(1)	-6(1)	2(1)	-4(1)
C16	14(1)	14(1)	13(1)	-3(1)	-1(1)	-3(1)
C17	19(1)	17(1)	13(1)	-2(1)	-1(1)	-1(1)
C18	22(1)	19(1)	15(1)	-5(1)	2(1)	-1(1)
C19	26(1)	18(1)	21(1)	-7(1)	3(1)	2(1)
C20	26(1)	17(1)	27(1)	-7(1)	-1(1)	4(1)
C21	31(1)	15(1)	22(1)	-1(1)	-6(1)	1(1)
C22	24(1)	17(1)	18(1)	-3(1)	-1(1)	-2(1)

C23	17(1)	14(1)	17(1)	-4(1)	-2(1)	-2(1)
C24	17(1)	15(1)	18(1)	-4(1)	-1(1)	-1(1)
C25	22(1)	23(1)	24(1)	-6(1)	-1(1)	-2(1)
C26	28(1)	29(1)	27(1)	4(1)	-5(1)	1(1)
Br2	30(1)	16(1)	25(1)	-6(1)	4(1)	-6(1)

Table S-5. Hydrogen coordinates (x 10^4) and isotropic displacement parameters ($Å^2$ x 10^3) for jonap37.

	x	y	z	U(eq)
H1A	9866	7513	7080	20
H1B	9330	6601	6557	20
H3A	10515	4791	7435	19
H3B	10970	4698	8545	19
H4	6694	4351	8606	17
H5A	10321	6568	9082	22
H5B	8656	7622	8854	22
H6	6578	5320	9769	17
H8	8445	2925	7181	22
H9	8161	2961	5530	24
H10	7280	4210	3846	23
H11	6147	6088	2851	25
H12	5281	7887	3512	25
H13	5538	7779	5176	21
H17	5574	6684	11044	21
H18	3505	8382	11322	24
H19	1643	10431	10615	28
H20	811	12011	9265	30
H21	2120	11889	7700	29
H22	4108	10143	7462	24
H26A	1827	10100	4874	47
H26B	2177	11060	5430	47
H26C	803	10379	5866	47
H1	7600(40)	8620(30)	6980(30)	35(10)

Table S-6. Torsion angles [°] for jonap37.

Ni1-O1-C1-C2	-34.9(2)	N3-C23-C24-C19	-174.8(2)
Ni1-N1-C2-C1	-14.5(2)	C1-C2-C3-O2	-128.2(2)
Ni1-N1-C2-C3	-135.76(14)	C1-C2-C5-O3	155.05(18)
Ni1-N1-C2-C5	106.78(16)	C2-N1-C4-O2	34.9(2)
Ni1-N1-C4-O2	156.41(13)	C2-N1-C4-C7	-84.9(2)
Ni1-N1-C4-C7	36.6(2)	C2-N1-C6-O3	-10.6(2)
Ni1-N1-C6-O3	-133.21(14)	C2-N1-C6-C16	108.3(2)
Ni1-N1-C6-C16	-14.3(2)	C3-O2-C4-N1	-40.9(2)
Ni1-N2-C7-C4	-8.2(2)	C3-O2-C4-C7	77.9(2)
Ni1-N2-C7-C8	171.99(19)	C3-C2-C5-O3	-74.5(2)
Ni1-N2-C14-C13	10.0(3)	C4-O2-C3-C2	29.8(2)
Ni1-N2-C14-C15	-169.77(17)	C4-N1-C2-C1	105.7(2)
Ni1-N3-C16-C6	22.8(2)	C4-N1-C2-C3	-15.6(2)
Ni1-N3-C16-C17	-158.3(2)	C4-N1-C2-C5	-133.05(18)
Ni1-N3-C23-C22	-32.5(3)	C4-N1-C6-O3	102.3(2)
Ni1-N3-C23-C24	148.55(19)	C4-N1-C6-C16	-138.84(19)
O1-C1-C2-N1	32.2(3)	C4-C7-C8-C9	179.9(2)
O1-C1-C2-C3	147.49(19)	C5-O3-C6-N1	33.9(2)
O1-C1-C2-C5	-81.9(2)	C5-O3-C6-C16	-87.0(2)
O2-C4-C7-N2	-134.4(2)	C5-C2-C3-O2	101.1(2)
O2-C4-C7-C8	45.5(3)	C6-O3-C5-C2	-43.5(2)
O3-C6-C16-N3	110.8(2)	C6-N1-C2-C1	-136.22(19)
O3-C6-C16-C17	-68.2(3)	C6-N1-C2-C3	102.51(19)
N1-C2-C3-O2	-8.0(2)	C6-N1-C2-C5	-15.0(2)
N1-C2-C5-O3	35.3(2)	C6-N1-C4-O2	-78.7(2)
N1-C4-C7-N2	-17.9(3)	C6-N1-C4-C7	161.46(18)
N1-C4-C7-C8	162.0(2)	C6-C16-C17-C18	-178.1(2)
N1-C6-C16-N3	-6.8(3)	C7-N2-C14-C13	-176.6(2)
N1-C6-C16-C17	174.2(2)	C7-N2-C14-C15	3.7(3)
N2-C7-C8-C9	-0.2(4)	C7-C8-C9-C15	2.4(4)
N2-C14-C15-C9	-1.7(3)	C8-C9-C15-C10	179.4(2)
N2-C14-C15-C10	177.5(2)	C8-C9-C15-C14	-1.4(4)
N3-C16-C17-C18	3.0(4)	C10-C11-C12-C13	-0.5(4)
N3-C23-C24-C18	6.6(4)	C11-C10-C15-C9	-179.7(2)

C11-C10-C15-C14	1.2(4)	C17-C18-C24-C23	-0.7(4)
C11-C12-C13-C14	-0.6(4)	C19-C20-C21-C22	2.9(4)
C12-C13-C14-N2	-177.8(2)	C20-C19-C24-C18	176.2(3)
C12-C13-C14-C15	2.0(4)	C20-C19-C24-C23	-2.3(4)
C13-C14-C15-C9	178.6(2)	C20-C21-C22-C23	1.1(4)
C13-C14-C15-C10	-2.3(3)	C21-C22-C23-N3	175.4(2)
C14-N2-C7-C4	177.1(2)	C21-C22-C23-C24	-5.6(4)
C14-N2-C7-C8	-2.8(3)	C22-C23-C24-C18	-172.4(2)
C15-C10-C11-C12	0.2(4)	C22-C23-C24-C19	6.2(4)
C16-N3-C23-C22	171.5(2)	C23-N3-C16-C6	-176.1(2)
C16-N3-C23-C24	-7.5(3)	C23-N3-C16-C17	2.7(3)
C16-C17-C18-C24	-3.8(4)	C24-C19-C20-C21	-2.3(4)
C17-C18-C24-C19	-179.2(2)		

Table S-7. Hydrogen bonds and close contacts for jonap37 [Å and °].

D-H...A	d(D-H)	d(H...A)	d(D...A)	<(DHA)
O1-H1...Br2	0.82(4)	2.26(4)	3.0686(17)	173(3)

REFERENCE NUMBER: jonap35

2d’

CRYSTAL STRUCTURE REPORT

$C_{52}H_{42}F_{12}N_6Ni_2O_{18}S_4$

or

$[(\kappa^4\text{-}L^q)Ni(\mu\text{-}OTf)]_2[OTf]_2$

Report prepared for:

R. Zhang, A. Panda, Prof. W. Jones

December 07, 2023

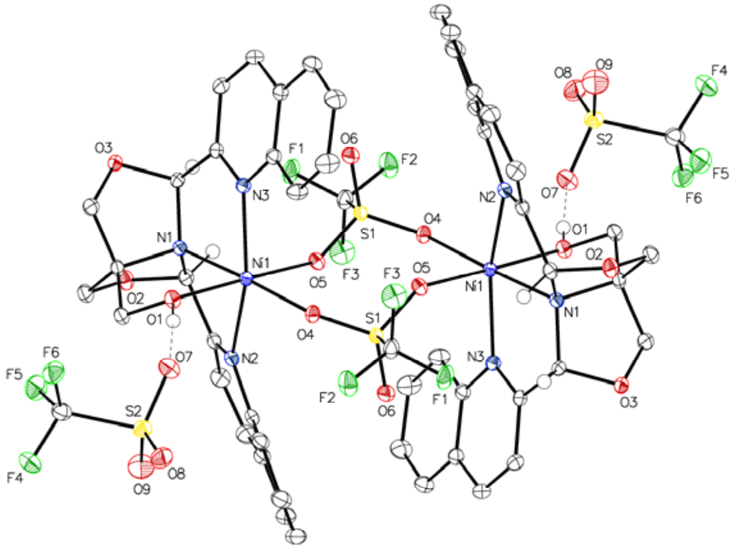

William W. Brennessel
X-ray Crystallographic Facility
Department of Chemistry, University of Rochester
120 Trustee Road
Rochester, NY 14627

Compound Isolation

During the recrystallization of compound **2d**, a small quantity of crystals formed that appeared distinct from the major crystallization product (**2d**). One of these minor crystals was examined by single-crystal X-ray structural determination, and found to be a desolvated version of **2d**, denoted as **2d'**.

Data collection

A crystal (0.346 x 0.259 x 0.243 mm^3) was placed onto a nylon loop and mounted on a Rigaku XtaLAB Synergy-S Dualflex diffractometer equipped with a HyPix-6000HE HPC area detector for data collection at 100.00(10) K. A preliminary set of cell constants and an orientation matrix were calculated from a small sampling of reflections.[1] A short pre-experiment was run, from which an optimal data collection strategy was determined. The full data collection was carried out using a PhotonJet (Cu) X-ray source with frame times of 0.05 and 0.06 seconds and a detector distance of 34.0 mm. Series of frames were collected in 0.50º steps in ω at different 2θ, κ, and ϕ settings. After the intensity data were corrected for absorption, the final cell constants were calculated from the xyz centroids of 34515 strong reflections from the actual data collection after integration.[1] See Table S-1 for additional crystal and refinement information.

Structure solution and refinement

The structure was solved using SHELXT[2] and refined using SHELXL[3] The space group $P2_1/c$ was determined based on systematic absences. Most or all non-hydrogen atoms were assigned from the solution. Full-matrix least squares / difference Fourier cycles were performed which located any remaining non-hydrogen atoms. All non-hydrogen atoms were refined with anisotropic displacement parameters. The O-H hydrogen atoms (one unique) were found from the difference Fourier map and refined freely. All other hydrogen atoms were placed in ideal positions and refined as riding atoms with relative isotropic displacement parameters. The final full matrix least squares refinement converged to $R1 = 0.0288$ (F^2, $I > 2\sigma(I)$) and $wR2 = 0.0706$ (F^2, all data).

Structure description

The structure is the one suggested. The asymmetric unit contains one-half of the dinickel salt adjacent to a crystallographic inversion center. The anions are linked to the dication by hydrogen bonding (see figures and Table S-7).

Structure manipulation and figure generation were performed using Olex2.[4] Unless noted otherwise all structural diagrams containing anisotropic displacement ellipsoids are drawn at the 50 % probability level.

Data collection, structure solution, and structure refinement were conducted at the X-ray Crystallographic Facility, B04 Hutchison Hall, Department of Chemistry, University of Rochester. The instrument was purchased with funding from NSF MRI program grant CHE-1725028. All publications arising from this report MUST either 1) include William W. Brennessel as a coauthor or 2) acknowledge William W. Brennessel and the X-ray Crystallographic Facility of the Department of Chemistry at the University of Rochester.

1 *CrysAlisPro*, version 171.42.101a; Rigaku Corporation: Oxford, UK, 2023.

2 Sheldrick, G. M. *SHELXT*, version 2018/2; *Acta. Crystallogr.* **2015**, *A71*, 3-8.

3 Sheldrick, G. M. *SHELXL*, version 2019/2; *Acta. Crystallogr.* **2015**, *C71*, 3-8.

4 Dolomanov, O. V.; Bourhis, L. J.; Gildea, R. J.; Howard, J. A. K.; Puschmann, H. *Olex2*, version 1.5; *J. Appl. Cryst.* **2009**, *42*, 339-341.

Some equations of interest:

$$R_{int} = \Sigma |F_o^2 - \langle F_o^2 \rangle| / \Sigma |F_o^2|$$

$$R1 = \Sigma||F_o|-|F_c|| / \Sigma|F_o|$$

$$wR2 = [\Sigma[w(F_o^2-F_c^2)^2] / \Sigma [w(F_o^2)^2]]^{1/2}$$

where $w = 1 / [\sigma^2 (F_o^2) + (aP)^2 + bP]$ and

$$P = 1/3 \max(0, F_o^2) + 2/3 F_c^2$$

$$GOF = S = [\Sigma[w(F_o^2-F_c^2)^2] / (m-n)]^{1/2}$$

where m = number of reflections and n = number of parameters

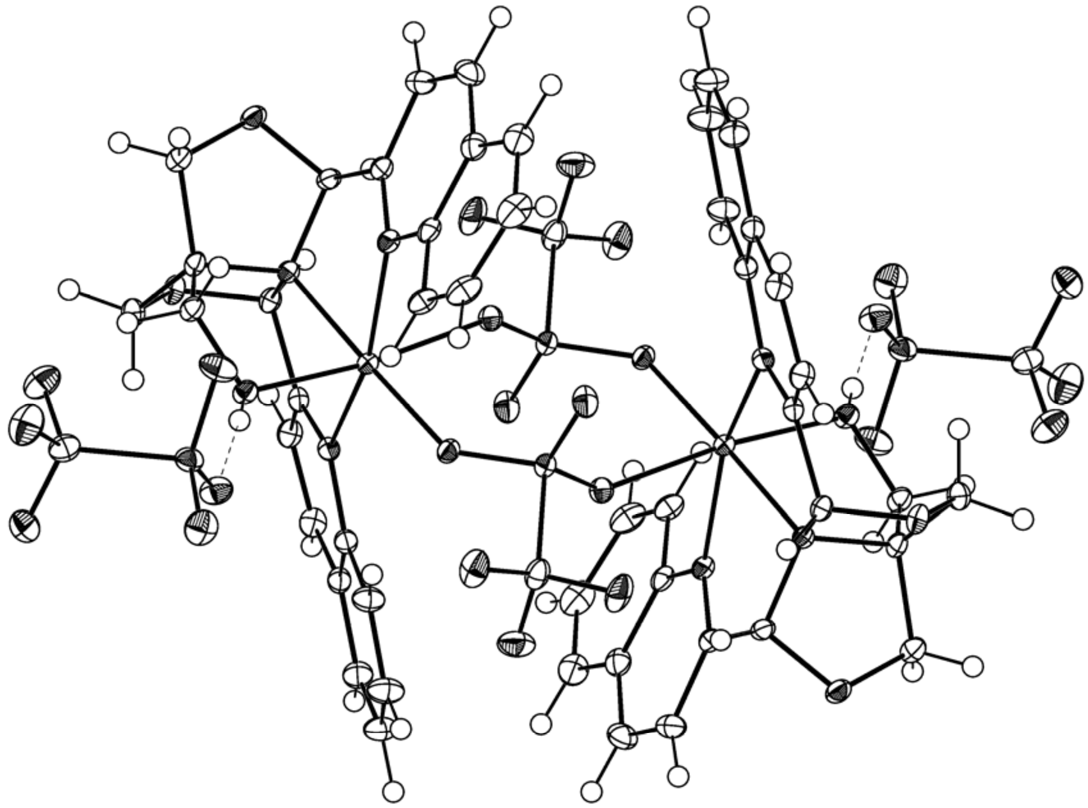

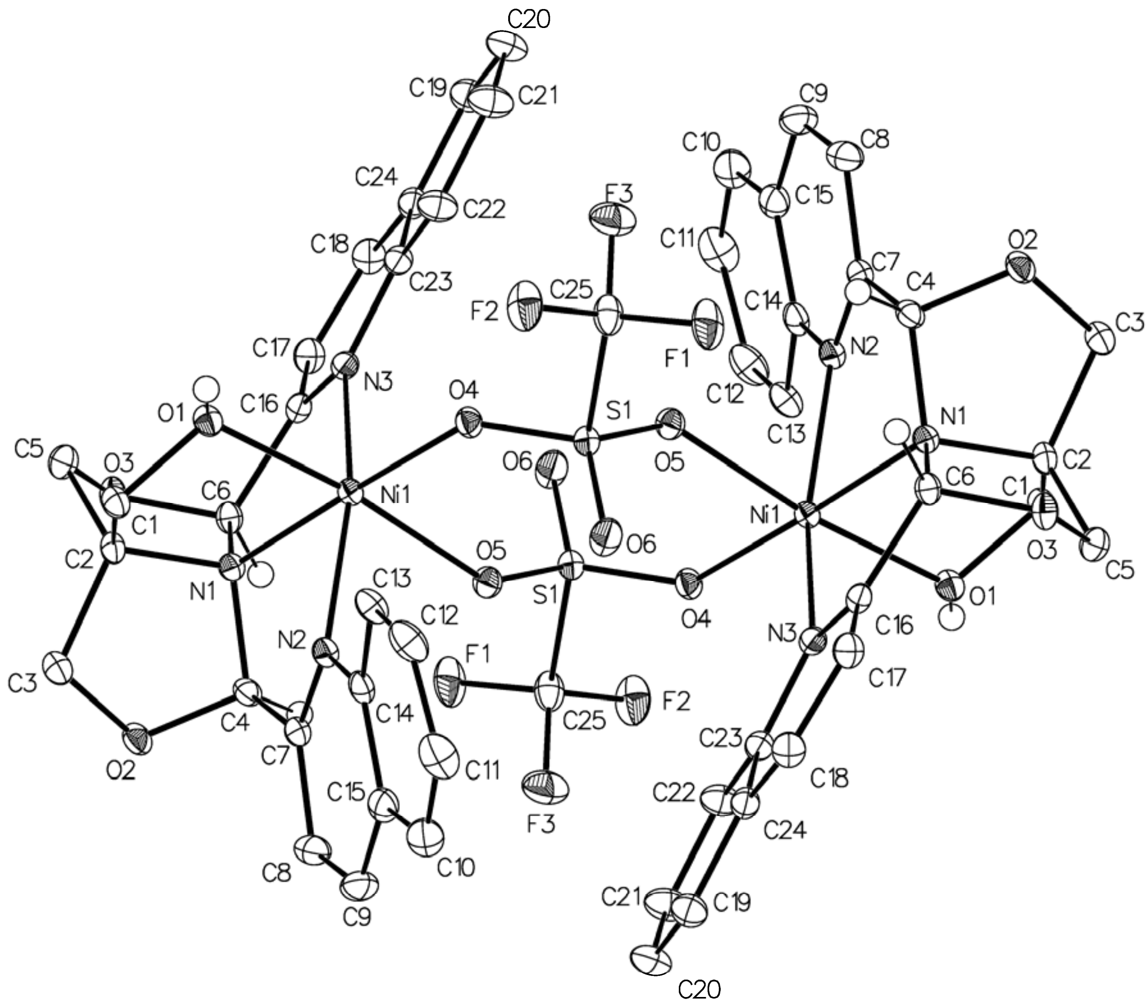
C20
C19
C21
C24
C22
C18
C23
C17
N3
C16
O1
O4
C5
O3
C6
C1
C2
N1
Ni1
O5
O6
S1
C25
F1
F2
F3
N2
C3
C4
C7
O2
C8
C9
C10
C11
C12
C13
C14
C15

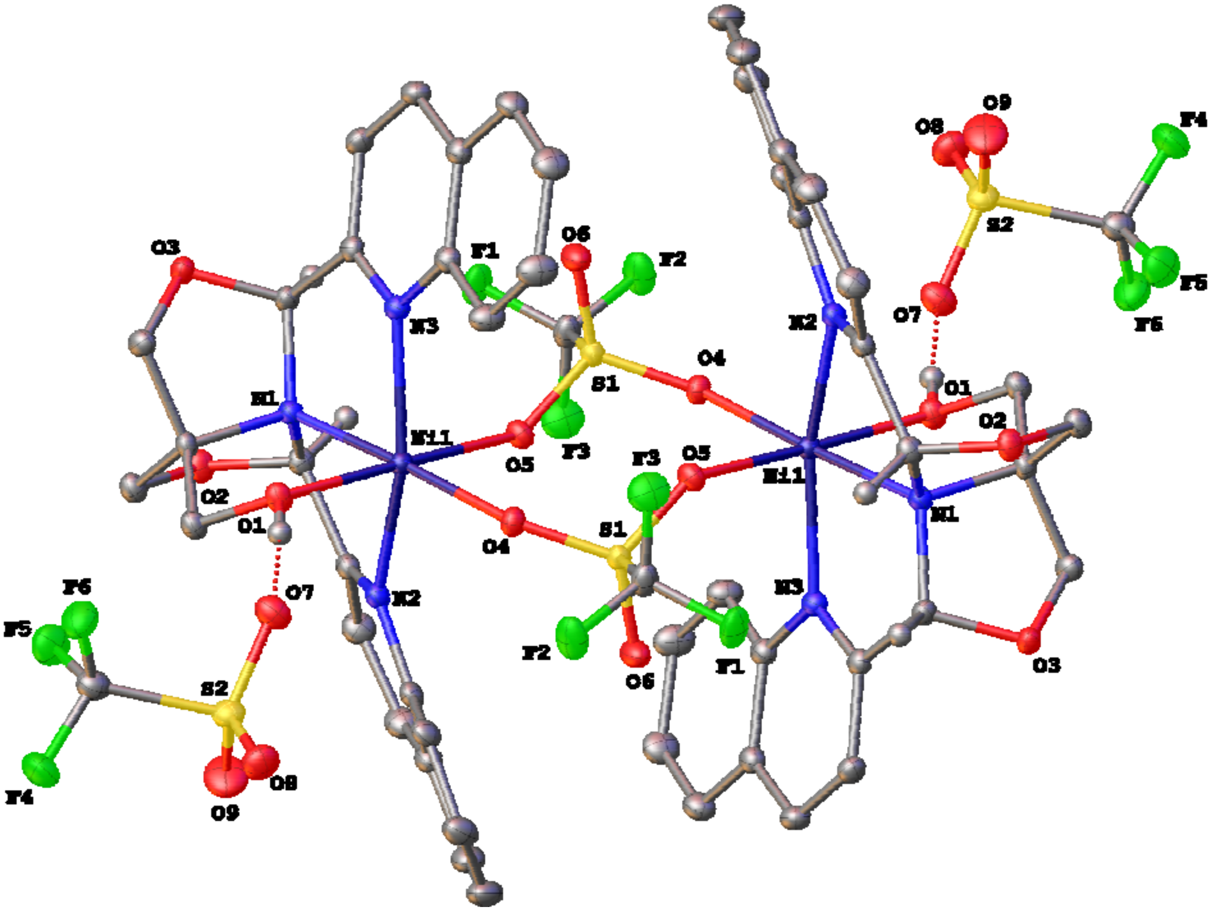
O9
O8
F4
S2
O6
F2
F1
O3
F5
F6
O7
N3
N2
O4
S1
O1
N1
Ni1
O5
F3
O2
O2
O5
Ni1
F3
O1
N1
O4
S1
O7
F6
N3
N2
F5
S2
F2
F1
O3
O6
F4
O8
O9

Table S-1. Crystal data and structure refinement for jonap35.

Identification code	jonap35	
Empirical formula	C52 H42 F12 N6 Ni2 O18 S4	
Formula weight	1512.57	
Temperature	100.00(10) K	
Wavelength	1.54184 Å	
Crystal system	monoclinic	
Space group	$P2_1/c$	
Unit cell dimensions	*a* = 12.84335(6) Å	α = 90°
	b = 18.47819(8) Å	β = 104.2006(4)°
	c = 12.38294(5) Å	γ = 90°
Volume	2848.94(2) $Å^3$	
Z	2	
Density (calculated)	1.763 Mg/m^3	
Absorption coefficient	3.271 mm^{-1}	
F(000)	1536	
Crystal color, morphology	blue-green, block	
Crystal size	0.346 x 0.259 x 0.243 mm^3	
Theta range for data collection	3.550 to 80.221°	
Index ranges	$-14 \le h \le 16$, $-22 \le k \le 23$, $-15 \le l \le 14$	
Reflections collected	48522	
Independent reflections	6153 [*R*(int) = 0.0352]	
Observed reflections	5990	
Completeness to theta = 74.504°	100.0%	
Absorption correction	Multi-scan	
Max. and min. transmission	1.00000 and 0.90277	
Refinement method	Full-matrix least-squares on F^2	
Data / restraints / parameters	6153 / 0 / 428	
Goodness-of-fit on F^2	1.083	
Final *R* indices [I>2sigma(I)]	*R*1 = 0.0288, *wR*2 = 0.0701	
R indices (all data)	*R*1 = 0.0296, *wR*2 = 0.0706	
Largest diff. peak and hole	0.390 and -0.444 e.$Å^{-3}$	

Table S-2. Atomic coordinates (x 10^4) and equivalent isotropic displacement parameters ($Å^2$x 10^3) for jonap35. U_{eq} is defined as one third of the trace of the orthogonalized U_{ij} tensor.

	x	y	z	U_{eq}
Ni1	5888(1)	4232(1)	3626(1)	10(1)
S1	5870(1)	4432(1)	6270(1)	11(1)
F1	5993(1)	3933(1)	8269(1)	24(1)
F2	6738(1)	3252(1)	7254(1)	25(1)
F3	5013(1)	3282(1)	6965(1)	24(1)
O1	6665(1)	3302(1)	3347(1)	13(1)
O2	6861(1)	5108(1)	1014(1)	14(1)
O3	4963(1)	3945(1)	327(1)	14(1)
O4	5950(1)	4040(1)	5271(1)	13(1)
O5	4811(1)	4742(1)	6186(1)	13(1)
O6	6759(1)	4879(1)	6776(1)	18(1)
N1	5900(1)	4446(1)	2016(1)	10(1)
N2	7318(1)	4869(1)	3841(1)	12(1)
N3	4377(1)	3765(1)	2828(1)	11(1)
C1	7313(1)	3468(1)	2582(1)	13(1)
C2	6618(1)	3907(1)	1623(1)	12(1)
C3	7300(1)	4390(1)	1041(1)	14(1)
C4	6384(1)	5159(1)	1930(1)	12(1)
C5	5794(1)	3442(1)	818(1)	14(1)
C6	4820(1)	4372(1)	1233(1)	12(1)
C7	7211(1)	5332(1)	3006(1)	12(1)
C8	7807(1)	5976(1)	3059(1)	17(1)
C9	8540(1)	6139(1)	4028(1)	19(1)
C10	9529(1)	5756(1)	5923(1)	21(1)
C11	9737(1)	5228(1)	6721(1)	23(1)
C12	9192(1)	4561(1)	6544(1)	20(1)
C13	8394(1)	4440(1)	5603(1)	16(1)
C14	8129(1)	4987(1)	4784(1)	13(1)
C15	8733(1)	5639(1)	4918(1)	16(1)
C16	4032(1)	4008(1)	1794(1)	12(1)
C17	2958(1)	3944(1)	1160(1)	14(1)

C18	2226(1)	3611(1)	1622(1)	16(1)
C19	1859(1)	2899(1)	3180(1)	17(1)
C20	2229(1)	2587(1)	4204(1)	20(1)
C21	3317(1)	2657(1)	4773(1)	21(1)
C22	4023(1)	3042(1)	4325(1)	17(1)
C23	3662(1)	3379(1)	3277(1)	12(1)
C24	2569(1)	3297(1)	2689(1)	14(1)
C25	5905(1)	3678(1)	7238(1)	17(1)
S2	8856(1)	2177(1)	5214(1)	15(1)
F4	10140(1)	1370(1)	4366(1)	28(1)
F5	8946(1)	1949(1)	3150(1)	27(1)
F6	8498(1)	1006(1)	3964(1)	25(1)
O7	7708(1)	2318(1)	4835(1)	20(1)
O8	9146(1)	1736(1)	6198(1)	23(1)
O9	9522(1)	2795(1)	5172(1)	31(1)
C26	9117(1)	1595(1)	4117(1)	18(1)

Table S-3. Bond lengths [Å] and angles [°] for jonap35.

Ni(1)-O(1)	2.0599(11)	C(4)-C(7)	1.520(2)
Ni(1)-O(4)	2.0498(10)	C(5)-H(5A)	0.9900
Ni(1)-O(5)#1	2.1337(11)	C(5)-H(5B)	0.9900
Ni(1)-N(1)	2.0362(12)	C(6)-H(6)	1.0000
Ni(1)-N(2)	2.1414(13)	C(6)-C(16)	1.519(2)
Ni(1)-N(3)	2.1329(12)	C(7)-C(8)	1.407(2)
S(1)-O(4)	1.4588(11)	C(8)-H(8)	0.9500
S(1)-O(5)	1.4562(11)	C(8)-C(9)	1.364(2)
S(1)-O(6)	1.4242(11)	C(9)-H(9)	0.9500
S(1)-C(25)	1.8315(16)	C(9)-C(15)	1.413(2)
F(1)-C(25)	1.3396(18)	C(10)-H(10)	0.9500
F(2)-C(25)	1.3247(18)	C(10)-C(11)	1.367(3)
F(3)-C(25)	1.331(2)	C(10)-C(15)	1.420(2)
O(1)-H(1)	0.79(3)	C(11)-H(11)	0.9500
O(1)-C(1)	1.4395(17)	C(11)-C(12)	1.408(3)
O(2)-C(3)	1.4376(18)	C(12)-H(12)	0.9500
O(2)-C(4)	1.4173(17)	C(12)-C(13)	1.369(2)
O(3)-C(5)	1.4330(18)	C(13)-H(13)	0.9500
O(3)-C(6)	1.4197(17)	C(13)-C(14)	1.412(2)
N(1)-C(2)	1.5173(18)	C(14)-C(15)	1.420(2)
N(1)-C(4)	1.4720(19)	C(16)-C(17)	1.414(2)
N(1)-C(6)	1.4904(18)	C(17)-H(17)	0.9500
N(2)-C(7)	1.3236(19)	C(17)-C(18)	1.362(2)
N(2)-C(14)	1.3788(19)	C(18)-H(18)	0.9500
N(3)-C(16)	1.3264(19)	C(18)-C(24)	1.411(2)
N(3)-C(23)	1.3823(19)	C(19)-H(19)	0.9500
C(1)-H(1A)	0.9900	C(19)-C(20)	1.368(2)
C(1)-H(1B)	0.9900	C(19)-C(24)	1.418(2)
C(1)-C(2)	1.530(2)	C(20)-H(20)	0.9500
C(2)-C(3)	1.548(2)	C(20)-C(21)	1.408(2)
C(2)-C(5)	1.528(2)	C(21)-H(21)	0.9500
C(3)-H(3A)	0.9900	C(21)-C(22)	1.373(2)
C(3)-H(3B)	0.9900	C(22)-H(22)	0.9500
C(4)-H(4)	1.0000	C(22)-C(23)	1.411(2)

C(23)-C(24)	1.422(2)	C(2)-N(1)-Ni(1)	109.69(9)
S(2)-O(7)	1.4571(12)	C(4)-N(1)-Ni(1)	110.44(9)
S(2)-O(8)	1.4373(12)	C(4)-N(1)-C(2)	105.30(11)
S(2)-O(9)	1.4357(13)	C(4)-N(1)-C(6)	112.20(11)
S(2)-C(26)	1.8270(17)	C(6)-N(1)-Ni(1)	112.97(9)
F(4)-C(26)	1.3399(19)	C(6)-N(1)-C(2)	105.83(11)
F(5)-C(26)	1.3341(19)	C(7)-N(2)-Ni(1)	109.67(9)
F(6)-C(26)	1.3323(19)	C(7)-N(2)-C(14)	118.15(13)
O(1)-Ni(1)-O(5)#1	173.71(4)	C(14)-N(2)-Ni(1)	129.70(10)
O(1)-Ni(1)-N(2)	92.48(5)	C(16)-N(3)-Ni(1)	111.15(10)
O(1)-Ni(1)-N(3)	90.44(5)	C(16)-N(3)-C(23)	117.73(12)
O(4)-Ni(1)-O(1)	96.87(4)	C(23)-N(3)-Ni(1)	129.91(10)
O(4)-Ni(1)-O(5)#1	87.71(4)	O(1)-C(1)-H(1A)	110.2
O(4)-Ni(1)-N(2)	98.58(4)	O(1)-C(1)-H(1B)	110.2
O(4)-Ni(1)-N(3)	101.44(5)	O(1)-C(1)-C(2)	107.61(11)
O(5)#1-Ni(1)-N(2)	82.53(4)	H(1A)-C(1)-H(1B)	108.5
N(1)-Ni(1)-O(1)	82.98(5)	C(2)-C(1)-H(1A)	110.2
N(1)-Ni(1)-O(4)	177.21(5)	C(2)-C(1)-H(1B)	110.2
N(1)-Ni(1)-O(5)#1	92.24(5)	N(1)-C(2)-C(1)	112.61(11)
N(1)-Ni(1)-N(2)	78.65(5)	N(1)-C(2)-C(3)	103.16(11)
N(1)-Ni(1)-N(3)	81.35(5)	N(1)-C(2)-C(5)	101.54(11)
N(3)-Ni(1)-O(5)#1	92.89(4)	C(1)-C(2)-C(3)	112.21(12)
N(3)-Ni(1)-N(2)	159.27(5)	C(5)-C(2)-C(1)	112.82(12)
O(4)-S(1)-C(25)	100.46(7)	C(5)-C(2)-C(3)	113.63(12)
O(5)-S(1)-O(4)	113.17(6)	O(2)-C(3)-C(2)	106.23(11)
O(5)-S(1)-C(25)	102.64(7)	O(2)-C(3)-H(3A)	110.5
O(6)-S(1)-O(4)	116.49(7)	O(2)-C(3)-H(3B)	110.5
O(6)-S(1)-O(5)	115.94(7)	C(2)-C(3)-H(3A)	110.5
O(6)-S(1)-C(25)	105.33(7)	C(2)-C(3)-H(3B)	110.5
Ni(1)-O(1)-H(1)	126.0(18)	H(3A)-C(3)-H(3B)	108.7
C(1)-O(1)-Ni(1)	108.28(9)	O(2)-C(4)-N(1)	105.72(11)
C(1)-O(1)-H(1)	109.6(18)	O(2)-C(4)-H(4)	109.8
C(4)-O(2)-C(3)	106.72(11)	O(2)-C(4)-C(7)	111.67(12)
C(6)-O(3)-C(5)	104.62(10)	N(1)-C(4)-H(4)	109.8
S(1)-O(4)-Ni(1)	139.70(7)	N(1)-C(4)-C(7)	109.99(12)
S(1)-O(5)-Ni(1)#1	138.81(7)	C(7)-C(4)-H(4)	109.8

O(3)-C(5)-C(2)	103.69(12)	C(9)-C(15)-C(10)	122.32(15)
O(3)-C(5)-H(5A)	111.0	C(9)-C(15)-C(14)	118.58(14)
O(3)-C(5)-H(5B)	111.0	C(14)-C(15)-C(10)	119.07(15)
C(2)-C(5)-H(5A)	111.0	N(3)-C(16)-C(6)	119.30(13)
C(2)-C(5)-H(5B)	111.0	N(3)-C(16)-C(17)	123.89(14)
H(5A)-C(5)-H(5B)	109.0	C(17)-C(16)-C(6)	116.81(13)
O(3)-C(6)-N(1)	106.57(11)	C(16)-C(17)-H(17)	120.5
O(3)-C(6)-H(6)	109.6	C(18)-C(17)-C(16)	118.91(14)
O(3)-C(6)-C(16)	110.22(12)	C(18)-C(17)-H(17)	120.5
N(1)-C(6)-H(6)	109.6	C(17)-C(18)-H(18)	120.3
N(1)-C(6)-C(16)	111.17(11)	C(17)-C(18)-C(24)	119.39(14)
C(16)-C(6)-H(6)	109.6	C(24)-C(18)-H(18)	120.3
N(2)-C(7)-C(4)	117.70(13)	C(20)-C(19)-H(19)	119.9
N(2)-C(7)-C(8)	124.01(14)	C(20)-C(19)-C(24)	120.17(14)
C(8)-C(7)-C(4)	118.29(13)	C(24)-C(19)-H(19)	119.9
C(7)-C(8)-H(8)	120.7	C(19)-C(20)-H(20)	120.0
C(9)-C(8)-C(7)	118.57(15)	C(19)-C(20)-C(21)	120.09(15)
C(9)-C(8)-H(8)	120.7	C(21)-C(20)-H(20)	120.0
C(8)-C(9)-H(9)	120.3	C(20)-C(21)-H(21)	119.4
C(8)-C(9)-C(15)	119.46(15)	C(22)-C(21)-C(20)	121.22(15)
C(15)-C(9)-H(9)	120.3	C(22)-C(21)-H(21)	119.4
C(11)-C(10)-H(10)	120.1	C(21)-C(22)-H(22)	120.0
C(11)-C(10)-C(15)	119.75(16)	C(21)-C(22)-C(23)	119.99(14)
C(15)-C(10)-H(10)	120.1	C(23)-C(22)-H(22)	120.0
C(10)-C(11)-H(11)	119.7	N(3)-C(23)-C(22)	119.79(13)
C(10)-C(11)-C(12)	120.68(15)	N(3)-C(23)-C(24)	121.24(13)
C(12)-C(11)-H(11)	119.7	C(22)-C(23)-C(24)	118.95(14)
C(11)-C(12)-H(12)	119.5	C(18)-C(24)-C(19)	121.93(14)
C(13)-C(12)-C(11)	121.08(16)	C(18)-C(24)-C(23)	118.50(14)
C(13)-C(12)-H(12)	119.5	C(19)-C(24)-C(23)	119.56(14)
C(12)-C(13)-H(13)	120.3	F(1)-C(25)-S(1)	109.80(11)
C(12)-C(13)-C(14)	119.44(15)	F(2)-C(25)-S(1)	110.65(11)
C(14)-C(13)-H(13)	120.3	F(2)-C(25)-F(1)	108.46(12)
N(2)-C(14)-C(13)	119.59(14)	F(2)-C(25)-F(3)	108.56(14)
N(2)-C(14)-C(15)	120.65(14)	F(3)-C(25)-S(1)	111.73(10)
C(13)-C(14)-C(15)	119.70(14)	F(3)-C(25)-F(1)	107.53(13)

O(7)-S(2)-C(26)	102.80(7)	F(4)-C(26)-S(2)	111.07(11)
O(8)-S(2)-O(7)	114.00(7)	F(5)-C(26)-S(2)	111.02(12)
O(8)-S(2)-C(26)	103.89(8)	F(5)-C(26)-F(4)	107.06(13)
O(9)-S(2)-O(7)	114.26(8)	F(6)-C(26)-S(2)	112.02(11)
O(9)-S(2)-O(8)	116.62(8)	F(6)-C(26)-F(4)	107.29(14)
O(9)-S(2)-C(26)	102.84(8)	F(6)-C(26)-F(5)	108.16(13)

Symmetry transformations used to generate equivalent atoms:

#1 -x+1,-y+1,-z+1

Table S-4. Anisotropic displacement parameters (Å^{2}x 10^3) for jonap35. The anisotropic displacement factor exponent takes the form: $-2\pi^2$[$h^2 a^{*2}U_{11}$ + ... + 2 h k a* b* U_{12}]

	U_{11}	U_{22}	U_{33}	U_{23}	U_{13}	U_{12}
Ni1	10(1)	11(1)	9(1)	0(1)	3(1)	0(1)
S1	11(1)	12(1)	10(1)	0(1)	3(1)	1(1)
F1	32(1)	30(1)	10(1)	2(1)	6(1)	10(1)
F2	30(1)	25(1)	21(1)	6(1)	8(1)	16(1)
F3	29(1)	21(1)	24(1)	7(1)	8(1)	-4(1)
O1	14(1)	13(1)	12(1)	3(1)	5(1)	3(1)
O2	18(1)	15(1)	13(1)	3(1)	7(1)	2(1)
O3	13(1)	18(1)	10(1)	-2(1)	3(1)	3(1)
O4	15(1)	13(1)	10(1)	0(1)	5(1)	1(1)
O5	12(1)	13(1)	15(1)	1(1)	4(1)	2(1)
O6	13(1)	20(1)	22(1)	-7(1)	4(1)	-1(1)
N1	10(1)	11(1)	11(1)	0(1)	3(1)	0(1)
N2	10(1)	13(1)	12(1)	0(1)	4(1)	0(1)
N3	11(1)	11(1)	12(1)	-1(1)	3(1)	0(1)
C1	12(1)	15(1)	14(1)	1(1)	5(1)	2(1)
C2	12(1)	13(1)	12(1)	-1(1)	5(1)	2(1)
C3	14(1)	16(1)	14(1)	2(1)	5(1)	2(1)
C4	13(1)	12(1)	12(1)	2(1)	5(1)	0(1)
C5	14(1)	14(1)	14(1)	-1(1)	2(1)	2(1)
C6	12(1)	14(1)	9(1)	0(1)	2(1)	1(1)
C7	11(1)	13(1)	14(1)	-1(1)	5(1)	0(1)
C8	18(1)	15(1)	19(1)	3(1)	6(1)	-2(1)
C9	17(1)	16(1)	25(1)	-2(1)	6(1)	-4(1)
C10	14(1)	26(1)	21(1)	-6(1)	3(1)	-4(1)
C11	13(1)	38(1)	17(1)	-3(1)	1(1)	-3(1)
C12	14(1)	31(1)	16(1)	5(1)	4(1)	1(1)
C13	12(1)	22(1)	16(1)	2(1)	6(1)	0(1)
C14	10(1)	17(1)	13(1)	-1(1)	5(1)	1(1)
C15	12(1)	19(1)	18(1)	-4(1)	5(1)	-2(1)
C16	12(1)	11(1)	12(1)	-2(1)	4(1)	1(1)
C17	14(1)	17(1)	11(1)	-1(1)	1(1)	1(1)

C18	12(1)	19(1)	15(1)	-2(1)	1(1)	0(1)
C19	13(1)	19(1)	20(1)	-1(1)	4(1)	-2(1)
C20	18(1)	21(1)	22(1)	3(1)	7(1)	-6(1)
C21	21(1)	22(1)	18(1)	7(1)	2(1)	-4(1)
C22	15(1)	18(1)	17(1)	3(1)	1(1)	-3(1)
C23	12(1)	12(1)	13(1)	-1(1)	4(1)	0(1)
C24	13(1)	14(1)	14(1)	-2(1)	4(1)	0(1)
C25	20(1)	19(1)	11(1)	2(1)	4(1)	6(1)
S2	14(1)	15(1)	17(1)	0(1)	3(1)	-3(1)
F4	16(1)	39(1)	30(1)	-2(1)	6(1)	6(1)
F5	30(1)	34(1)	18(1)	5(1)	9(1)	1(1)
F6	27(1)	22(1)	27(1)	-7(1)	9(1)	-7(1)
O7	17(1)	21(1)	21(1)	5(1)	6(1)	2(1)
O8	25(1)	28(1)	16(1)	2(1)	2(1)	3(1)
O9	30(1)	25(1)	41(1)	-6(1)	13(1)	-15(1)
C26	15(1)	22(1)	18(1)	2(1)	4(1)	-1(1)

Table S-5. Hydrogen coordinates (x 10^4) and isotropic displacement parameters ($Å^2$ x 10^3) for jonap35.

	x	y	z	U(eq)
H1	6980(20)	3030(14)	3810(20)	35(7)
H1A	7561	3017	2294	16
H1B	7951	3753	2962	16
H3A	8062	4387	1462	17
H3B	7255	4215	275	17
H4	5813	5540	1779	14
H5A	6112	3221	244	17
H5B	5513	3054	1219	17
H6	4543	4861	956	14
H8	7702	6290	2434	20
H9	8916	6586	4104	23
H10	9916	6199	6040	25
H11	10255	5313	7402	28
H12	9381	4190	7087	25
H13	8021	3991	5501	19
H17	2751	4131	423	17
H18	1492	3591	1230	19
H19	1126	2849	2796	21
H20	1751	2322	4532	24
H21	3566	2433	5480	25
H22	4755	3082	4721	21

Table S-6. Torsion angles [°] for jonap35.

Ni1-O1-C1-C2	-46.64(13)	O6-S1-C25-F2	-69.77(12)
Ni1-N1-C2-C1	-12.42(14)	O6-S1-C25-F3	169.14(10)
Ni1-N1-C2-C3	-133.61(9)	N1-C2-C3-O2	-6.84(14)
Ni1-N1-C2-C5	108.50(10)	N1-C2-C5-O3	34.37(13)
Ni1-N1-C4-O2	150.32(9)	N1-C4-C7-N2	-4.21(18)
Ni1-N1-C4-C7	29.61(13)	N1-C4-C7-C8	176.38(13)
Ni1-N1-C6-O3	-131.79(9)	N1-C6-C16-N3	-4.24(18)
Ni1-N1-C6-C16	-11.66(14)	N1-C6-C16-C17	176.23(12)
Ni1-N2-C7-C4	-21.78(15)	N2-C7-C8-C9	-0.3(2)
Ni1-N2-C7-C8	157.60(12)	N2-C14-C15-C9	-4.7(2)
Ni1-N2-C14-C13	31.4(2)	N2-C14-C15-C10	177.16(14)
Ni1-N2-C14-C15	-151.46(11)	N3-C16-C17-C18	-0.5(2)
Ni1-N3-C16-C6	17.08(16)	N3-C23-C24-C18	0.9(2)
Ni1-N3-C16-C17	-163.42(12)	N3-C23-C24-C19	-179.76(14)
Ni1-N3-C23-C22	-20.8(2)	C1-C2-C3-O2	-128.30(12)
Ni1-N3-C23-C24	160.84(11)	C1-C2-C5-O3	155.14(11)
O1-C1-C2-N1	39.23(16)	C2-N1-C4-O2	31.98(14)
O1-C1-C2-C3	155.10(12)	C2-N1-C4-C7	-88.73(13)
O1-C1-C2-C5	-75.00(15)	C2-N1-C6-O3	-11.75(14)
O2-C4-C7-N2	-121.26(14)	C2-N1-C6-C16	108.38(13)
O2-C4-C7-C8	59.32(18)	C3-O2-C4-N1	-37.22(14)
O3-C6-C16-N3	113.71(14)	C3-O2-C4-C7	82.39(14)
O3-C6-C16-C17	-65.83(16)	C3-C2-C5-O3	-75.68(14)
O4-S1-O5-Ni1#1	-126.09(9)	C4-O2-C3-C2	27.06(15)
O4-S1-C25-F1	171.32(10)	C4-N1-C2-C1	106.42(13)
O4-S1-C25-F2	51.63(12)	C4-N1-C2-C3	-14.77(13)
O4-S1-C25-F3	-69.46(12)	C4-N1-C2-C5	-132.66(11)
O5-S1-O4-Ni1	65.48(12)	C4-N1-C6-O3	102.57(13)
O5-S1-C25-F1	-71.84(12)	C4-N1-C6-C16	-137.30(12)
O5-S1-C25-F2	168.47(11)	C4-C7-C8-C9	179.08(14)
O5-S1-C25-F3	47.38(12)	C5-O3-C6-N1	34.45(14)
O6-S1-O4-Ni1	-72.68(12)	C5-O3-C6-C16	-86.30(13)
O6-S1-O5-Ni1#1	12.31(13)	C5-C2-C3-O2	102.22(14)
O6-S1-C25-F1	49.92(12)	C6-O3-C5-C2	-43.28(14)

C6-N1-C2-C1	-134.58(12)	C17-C18-C24-C19	-175.43(15)
C6-N1-C2-C3	104.23(12)	C17-C18-C24-C23	3.9(2)
C6-N1-C2-C5	-13.67(14)	C19-C20-C21-C22	0.7(3)
C6-N1-C4-O2	-82.67(13)	C20-C19-C24-C18	178.34(16)
C6-N1-C4-C7	156.62(11)	C20-C19-C24-C23	-1.0(2)
C6-C16-C17-C18	178.98(14)	C20-C21-C22-C23	0.2(3)
C7-N2-C14-C13	-168.45(13)	C21-C22-C23-N3	-179.89(15)
C7-N2-C14-C15	8.7(2)	C21-C22-C23-C24	-1.5(2)
C7-C8-C9-C15	4.4(2)	C22-C23-C24-C18	-177.49(14)
C8-C9-C15-C10	176.15(15)	C22-C23-C24-C19	1.8(2)
C8-C9-C15-C14	-1.9(2)	C23-N3-C16-C6	-174.24(13)
C10-C11-C12-C13	-4.2(3)	C23-N3-C16-C17	5.3(2)
C11-C10-C15-C9	-175.03(16)	C24-C19-C20-C21	-0.3(3)
C11-C10-C15-C14	3.0(2)	C25-S1-O4-Ni1	174.21(10)
C11-C12-C13-C14	1.4(2)	C25-S1-O5-Ni1#1	126.55(10)
C12-C13-C14-N2	-179.32(14)	O7-S2-C26-F4	-176.12(11)
C12-C13-C14-C15	3.5(2)	O7-S2-C26-F5	64.88(12)
C13-C14-C15-C9	172.39(14)	O7-S2-C26-F6	-56.16(13)
C13-C14-C15-C10	-5.7(2)	O8-S2-C26-F4	-57.05(13)
C14-N2-C7-C4	174.35(12)	O8-S2-C26-F5	-176.06(11)
C14-N2-C7-C8	-6.3(2)	O8-S2-C26-F6	62.91(13)
C15-C10-C11-C12	1.9(3)	O9-S2-C26-F4	64.93(13)
C16-N3-C23-C22	173.03(14)	O9-S2-C26-F5	-54.07(13)
C16-N3-C23-C24	-5.4(2)	O9-S2-C26-F6	-175.11(12)
C16-C17-C18-C24	-4.1(2)		

Symmetry transformations used to generate equivalent atoms:

#1 -x+1,-y+1,-z+1

Table S-7. Hydrogen bonds and close contacts for jonap35 [Å and °].

D-H...A	d(D-H)	d(H...A)	d(D...A)	<(DHA)
O1-H1...O7	0.79(3)	1.90(3)	2.6974(16)	175(3)
